# Gene-environment and protein-degradation signatures characterize genomic and phenotypic diversity in wild *Caenorhabditis elegans* populations

**DOI:** 10.1186/1741-7007-11-93

**Published:** 2013-08-19

**Authors:** Rita JM Volkers, L Basten Snoek, Caspara J van Hellenberg Hubar, Renata Coopman, Wei Chen, Wentao Yang, Mark G Sterken, Hinrich Schulenburg, Bart P Braeckman, Jan E Kammenga

**Affiliations:** 1 Laboratory of Nematology, Wageningen University, Droevendaalsesteeg 1, Wageningen 6708PB, The Netherlands; 2 Biology Department, Ghent University, Proeftuinstraat 86 N1, B-9000 Gent, Belgium; 3 Department of Evolutionary Ecology and Genetics, Zoological Institute, Christian Albrechts-Universitaet zu Kiel, Am Botanischen Garten 1-9, Kiel 24118, Germany

**Keywords:** Gene-environment interactions, Genotype-phenotype relations, Wild *C*. *elegans* strains, Transcriptomic diversity

## Abstract

**Background:**

Analyzing and understanding the relationship between genotypes and phenotypes is at the heart of genetics. Research on the nematode *Caenorhabditis elegans* has been instrumental for unraveling genotype-phenotype relations, and has important implications for understanding the biology of mammals, but almost all studies, including forward and reverse genetic screens, are limited by investigations in only one canonical genotype. This hampers the detection and functional analysis of allelic variants, which play a key role in controlling many complex traits. It is therefore essential to explore the full potential of the natural genetic variation and evolutionary context of the genotype-phenotype map in wild *C*. *elegans* populations.

**Results:**

We used multiple wild *C*. *elegans* populations freshly isolated from local sites to investigate gene sequence polymorphisms and a multitude of phenotypes including the transcriptome, fitness, and behavioral traits. The genotype, transcriptome, and a number of fitness traits showed a direct link with the original site of the strains. The separation between the isolation sites was prevalent on all chromosomes, but chromosome *V* was the largest contributor to this variation. These results were supported by a differential food preference of the wild isolates for naturally co-existing bacterial species. Comparing polymorphic genes between the populations with a set of genes extracted from 19 different studies on gene expression in *C*. *elegans* exposed to biotic and abiotic factors, such as bacteria, osmotic pressure, and temperature, revealed a significant enrichment for genes involved in gene-environment interactions and protein degradation.

**Conclusions:**

We found that wild *C. elegan*s populations are characterized by gene-environment signatures, and we have unlocked a wealth of genotype-phenotype relations for the first time. Studying natural isolates provides a treasure trove of evidence compared with that unearthed by the current research in *C. elegans*, which covers only a diminutive part of the myriad of genotype-phenotype relations that are present in the wild.

## Background

The nematode *Caenorhabditis elegans* is a widely used model species in contemporary biological research, which covers a number of disciplines including developmental biology, genetics, and evolutionary biology. Many investigations have been of paramount importance for understanding the biology of mammals, but almost all studies in *C*. *elegans*, including forward (knocking out genes by mutation) and reverse (knocking down genes using RNA interference (RNAi)) genetic screens, have been conducted in only a few strains of this organism, of which the canonical strain Bristol N2 has been the most thoroughly studied. This severely constrains the detection and functional analysis of allelic variants, which play a key role in controlling many complex traits. It is therefore essential to explore the full potential of the natural genetic variation and evolutionary context of the genotype-phenotype map in wild *C*. *elegans* populations. Moreover, the widely used strains, such as N2 and CB4856, have often been kept under controlled laboratory conditions for decades, and the handling, storage, and maintenance of worms is standardized. Such artificial regimens very likely create multiple bottlenecks that can affect the genotype-phenotype relationship. For instance, a genetic analysis of wild *C*. *elegans* strains showed that the N2 alleles of *npr*-*1* and *glb*-*5* most likely originated as an adaptation to laboratory conditions [1]. Genotype-phenotype relations have been studied in model organisms of many species, such as *Arabidopsis*[2,3], *Drosophila*[4], and *C*. *elegans*[5,6]. For the full appreciation and functional characterization of genes and the genotype-phenotype relations, it is essential to consider the natural context of the species, including analysis of natural isolates, and the interaction of the species with natural challenges. Previous studies on *C*. *elegans* have investigated the organism’s response to a wide range of different environmental factors, including exposure to different bacteria [7-9], pH [9], osmotic pressure [9,10], chemicals [11,12], and temperature [9,12-16], among others. As yet, however, these responses have not been tested in natural populations.

*C*. *elegans* is an androdioecious species with a low outcrossing rate, leading to homozygous strains in natural isolates [17]. These strains can therefore be treated as haplotypes. In this study, we investigated variation in genotype-phenotype relations for a total of 48 strains, of which 41 were freshly isolated from two different sites in France: 20 strains from a woodland area in Santeuil (S) from rotting hogweed stems, and 21 from an orchard in Orsay (O) from rotting apples. As an out-group, we used three strains freshly isolated from sites in the Netherlands and two strains previously isolated from France. Lastly, the genotypically most diverse laboratory-kept strain CB4856 and the canonical strain Bristol N2 were added (see Additional file 1, worksheet A) [9,18-22]; these two strains have been used in many studies to uncover genotype-phenotype relations both by comparing strains or by using some type of quantitative trait loci approach [1,18,23-30].

In this study, we provide insight into the genotype-phenotype relations in natural *C*. *elegans* populations through analysis of its genomic and transcriptomic variation. We found that local genetic diversity reflects site-specific signatures of environmental sensing, protein regulation, and the immune defense system. Our results indicate that exploring natural isolates in *C*. *elegans* should lead to identification of key components of genotype-phenotype relations compared with studies that are limited to the canonical strain Bristol N2.

## Results and discussion

### Local *C*. *elegans* populations are genotypically separable

Previous investigations have studied population genetics and genomic diversity in *C*. *elegans*, focusing on global [31-35] or local [17,36] populations. Cutter showed that there is a lack of geographic distribution of *C*. *elegans* genome sequences [31], and Andersen *et al*. reported that chromosome-scale selective sweeps have acted to reduce genetic variation, and have shaped the global *C*. *elegans* population structure in recent history [37]. Barrière and Félix concluded that local diversity in this organism is high [17]. In all of these papers, diversity in *C*. *elegans* was measured as genetic diversity. To date, very few papers have been published concerning phenotypic variation in wild isolates (by this we mean isolates that have not been maintained in the laboratory for a long time), and studies used only a small number of isolates [38-40]. To our knowledge, no studies have been reported on genotype-phenotype relations in wild populations.

**Figure 1:**
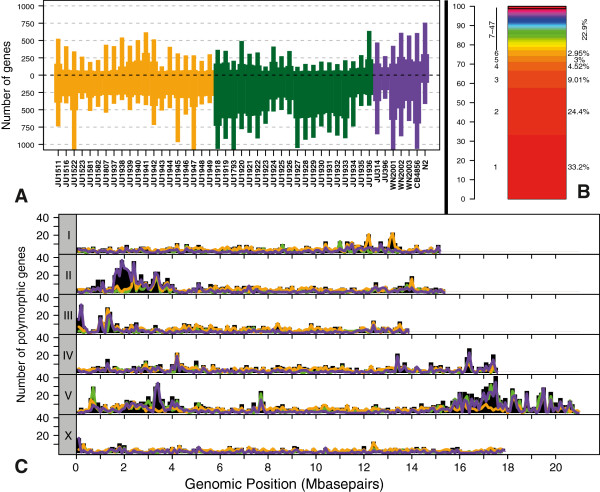
**Polymorphic genes in wild isolates of *Caenorhabditis elegans*. (A)** Number of polymorphic genes per strain. Bars above zero indicate the polymorphic genes with a positive ratio (higher intensity than the mean), while bars below zero indicate the polymorphic genes with a negative ratio. The wide part of the bars refers to the genes with a ratio of greater than 1 or less than −1, while the narrow part of the bars indicates genes with a ratio of greater than 0.5 or less than −0.5. Orsay strains are shown in orange, Santeuil strains in green, and the out-group strains in purple.** (B)** Frequency of occurrence of polymorphic genes. Number of strains is indicated on the left, and the percentage of total is shown on the right; for example, polymorphic genes only found in one strain make up 33.2% of the total number of polymorphic genes. **(C)** Distribution of the polymorphic genes in 48 different *C*. *elegans* strains. Genomic position is shown on the *x*-axis, and the number of polymorphic genes is shown on the *y*-axis. Chromosomes are shown in different panels, and chromosome names are given on the left in the gray boxes. The black bars indicate the total number of polymorphic genes per 100 kb. The lines show the number of genes with a ratio of less than −0.5 for three different groups of *C*. *elegans* strains, Orsay strains are shown in orange, Santeuil strains in green, and the out-group strains in purple; for example, the large number of polymorphic genes at the beginning (left arm) of chromosome *II* is mostly caused by the many genes that are very polymorphic in or absent from the out-group lines (purple line is high).

As mentioned above, we used wild strains from Santeuil and Orsay and a number of out-group strains. All these were genotyped based on the hybridization of genomic *C*. *elegans* DNA to microarrays (see Methods section for details), resulting in the identification of 6,368 polymorphic genes with an absolute ratio of 0.5 for the mean hybridization intensity (Figure 1A). Most of these (around 66%) were found in only one to three strains (Figure 1B), showing that between-strain variation is more abundant than between-site variation. Major hotspots of polymorphic genes were found on chromosomes *II* and *V* and minor on the other chromosomes (Figure 1C; see Additional file 1 worksheet B; see Additional files 2, 3, 4). The hotspots of polymorphic genes co-localize with the c-type lectin, nuclear hormone receptor (nhr), and math genes, and chemoreceptor gene clusters [41]. In addition, microsatellite loci were used to determine the population structure [36] of the Orsay and Santeuil strains (see Additional file 1, worksheet C; see Additional file 5, panel A).

**Figure 2:**
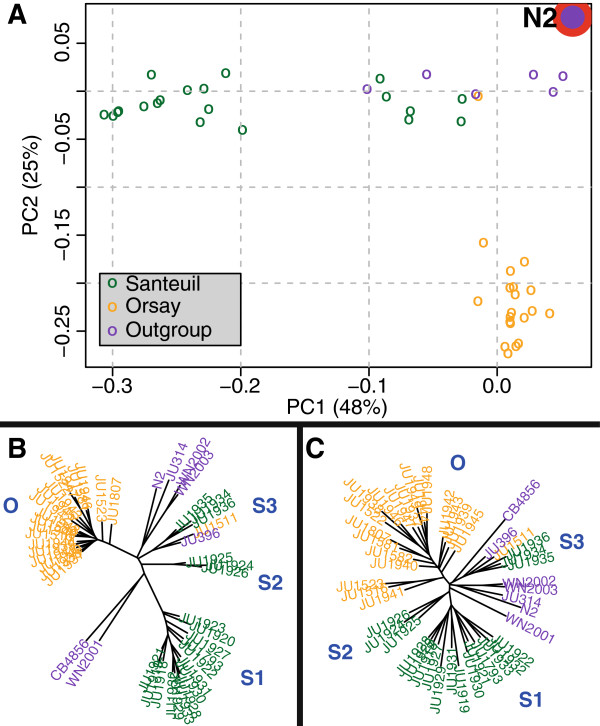
**The Orsay and Santeuil populations were found to be genotypically separable based on genomic DNA analysis with microarrays. (A)** Principal component analysis (PCA) plot. PC1 on the x-axis separates the main Santeuil group (green) from all other strains, and PC2 on the y-axis separates the Santeuil and out-group strains (purple) from the Orsay strains (yellow). **(B)** Neighbor-joining (NJ) tree created with the same genetic data as used for the PCA plot. Orsay strains are shown in orange, Santeuil strains in green, and the out-group strains in purple.** (C)** NJ tree based on the RNA hybridization data. The average log2 ratio per probe (with the mean) per genotype was used. Only probes with a maximum absolute ratio of greater than 0.5 were used. The 192 genes whose expression level was solely influenced by DNA polymorphisms were not included. Orsay strains are shown in orange, Santeuil strains in green, and the out-group strains in purple.

The two isolation sites were genotypically separable. This was shown by analyses of the presence of gene polymorphisms using principal component analysis (PCA) (Figure 2A), a distance matrix visualized by an unrooted neighbor-joining (NJ) tree (Figure 2B), and a minimum spanning network of the microsatellite data (see Additional file 5, panel A). The minimum spanning network, PCA, and NJ analyses showed a clear distinction between the Santeuil and the Orsay strains, with one large genetic group and several smaller genetic groups being identified for both isolation sites. In PCA, the first two principal components capture around 75% of the variation in DNA hybridizations. As shown in Figure 2A, N2 is in the far right top corner, indicating its genetic difference from all other strains. Moreover, the NJ tree showed that the Santeuil strains (groups S1, to S3) and Orsay strains are different from both N2 and CB4856. In both the PCA and NJ analyses, the Orsay group (group O) was seen to be genetically less diverse than the Santeuil group. Within the main Santeuil group (group S1: all Santeuil strains except JU1924, JU1925, JU1926, JU1934, JU1935, and JU1936), diversity was slightly larger. Furthermore, in the NJ tree two small genotypic groups were separate from the main Orsay and Santeuil groups (group S2: JU1924, JU1925, and JU1926; group S3: JU1511, JU1934, JU1935, and JU1936). The strains within these separate groups were all from the Santeuil site, except for JU1511, which is from the Orsay site. The strains from Santeuil in group S2 were isolated from a single hogweed stem. Similarly, those from S3 were also isolated from their own single hogweed stem (see Additional file 1, worksheet A). Other strains were found on different hogweed stems. We found that strains isolated from an individual hogweed stem grouped close together, but were not found to form their own separate genotypic groups.

The Dutch strains were isolated from two isolation sites, with WN2001 isolated from one site and WN2002 and WN2003 from the other. The latter two strains grouped together in the NJ tree. One of the French out-group strains, JU396, grouped with the Santeuil strains, whereas the other, JU314, was different from the rest of the strains. N2 and CB4856 were as diverse from the other out-group members as the whole out-group was from the Orsay or Santeuil strains. By comparing the four genetic groups (O, S1, S2, and S3) with the out-group, the genes that were polymorphic were identified (see Additional file 5, panel B). Group S1 appeared to be the most divergent from the out-group, with 3,181 genes that differed significantly (false discovery rate (FDR) = 0.05).

**Figure 3:**
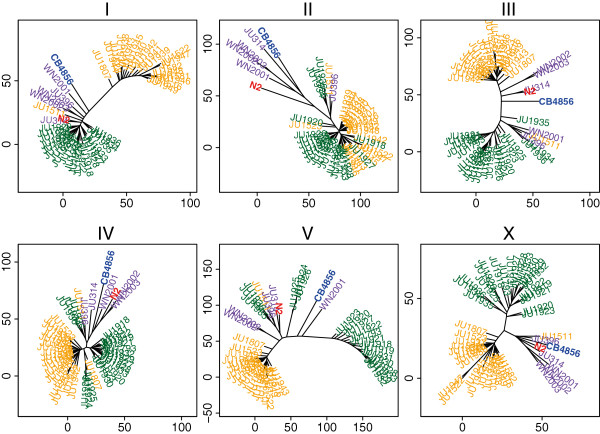
**Neighbor-joining tree of a distance matrix of the genetic polymorphisms for each chromosome.** Upper panels: chromosomes *I* to *III*; lower panels: chromosomes *IV* to *X*. Orsay strains are shown in orange, Santeuil strains in green, and the out-group strains in purple.

The genetic separation between the Orsay and Santeuil populations was prevalent on all chromosomes (Figure 3). From the scale of the axes, it can be seen that most chromosomes contributed to the separation between the two isolation sites and the out-group, except for chromosome *II*; on this chromosome the Santeuil and Orsay lines formed one group that was separate only from the out-group. Chromosome *V* was the largest contributor to the variation between Orsay and Santeuil, most likely because of the generally higher level of variation among these strains (see Additional file 4). Of the approximately 2,500 genes that were different between S1, S2, and S3, around 1,050 are located on chromosome *V*. Of all the genes on chromosome *V*, around 210 are polymorphic between S1, S2, and S3. This is a significant enrichment (*P*<1 × 10^-76^) when compared with the other chromosomes, of which 8 to 10% of the genes are polymorphic. Chromosomes *I* and *X* are under-represented for polymorphic genes (both around 8% and *P*<1 × 10^-12^).

The detected genotypic diversity between sites is in line with genotyping results from 31 markers using amplified fragment length polymorphism analysis, and with microsatellite results from two loci in other local *C*. *elegans* populations [17]. However, selective sequencing using restriction site-associated DNA tags did not reveal significant local diversity, possibly because of the limited number of different genotypes per location [37].

The four genetic groups (O, S1, S2, and S3) identified by analysis of the ratio intensities were used as input to search for all the genes linked to each isolation site. This allowed us to identify polymorphic genes by minor hybridization differences (absolute ratio (AR) <0.5), beyond those 6,386 found by major hybridization differences (AR >0.5). In this way, we identified 3,742 genes (FDR = 0.05) that were linked to each isolation site (see Additional file 6, panel A). Of these 3,742 genes, 2,403 were already identified as highly polymorphic in the initial analysis on major hybridization differences, and an additional 1,339 genes associated with isolation were found with only minor hybridization differences. Of the genes with major hybridization differences, around 62% could not be linked to an isolation site, again showing that between-strain variation is more abundant than between-site variation. Genes linked to isolation site were fond to be enriched for the gene classes fbox, math, bath, btb, C-type lectin (clec), serpentine chemoreceptor, and nhr.

To investigate whether specific types of genes are over-represented in the group of genes that could be linked to isolation site (n = 3,742) or in the polymorphic genes not linked to isolation site (n = 3,965), enrichment analyses were performed (Table 1; see Additional file 1, worksheets D and E). The linked and unlinked groups were analyzed using three types of annotations: gene class, anatomy terms and gene ontology (GO) terms, to investigate whether certain types of genes were enriched. For the gene classes, we found that fbox, math, bath, btb, clec, serpentine chemoreceptor, and nhr genes were enriched in the group of genes linked to isolation site (Table 1, see Additional file 1, worksheets F–H; see Additional file 6, panel B). Enrichment analyses were also performed for the genes that were significantly linked to the genetic groups O, S1, S2, and S3, revealing the same gene classes as above (see Additional file 1, worksheet I). The enrichment analysis of anatomy terms or GO terms did not identify a clear pattern linked to isolation site or genetic group (see Additional file 1, worksheets J and K).

**Table 1:** **Enrichment (based on DNA-array data) of gene classes**^**a**^

**Gene group**	**Gene class**	**Group size**	**Isolation site**		**Polymorphic by ratio**	
			**Overlap**	**Significance**^**b**^	**Overlap**	**Significance**
Serpentine receptors						
Superfamily Str	srh	289	118	20.3^c^	40	0.0
	str	219	68	6.4^c^	39	0.2
	sri	76	28	4.7^c^	12	0.1
	srj	45	19	4.5^c^	7	0.2
Solo	srz	104	43	8.4^c^	27	1.4
	srw	145	53	7.8^c^	20	0.0
	srbc	84	32	5.6^c^	18	0.6
	srr	10	5	2.5^c^	3	1.0
Superfamily Sra	srab	27	11	2.9^c^	5	0.4
Superfamily Srg	srt	72	29	5.8^c^	11	0.1
Others						
C-type lectins	clec	260	72	4.7^c^	44	0.1
F-box	fbxa	220	115	31.5^c^	38	0.1
	fbxb	113	43	7.1^c^	24	0.6
	fbxc	49	14	1.7	8	0.2
Math, bath, btb	math	50	41	23.6^c^	4	0.0
	bath	44	26	9.9^c^	9	0.5
	btb	21	8	2.2	4	0.4
Nuclear hormone receptor	nhr	282	71	3.2^c^	57	0.5
Pharyngeal gland toxin-related	phat	6	5	4.5^c^	0	NA
Scramblase (phospholipid scramblase)	scrm	8	6	4.4^c^	0	NA

^a^Enrichment (based on DNA-array data) of gene classes in the group of genes with variation linked to isolation site (3,742 genes) and the group of polymorphic genes that were not linked to isolation site (3,965 genes).

^b^Significance in –log10(p).

^c^Gene classes with group sizes ≥ 6, overlap ≥ 3 genes, and significance ≥ 2.5.

### Local *C*. *elegans* populations are separable on the basis of their transcriptomes

Next, the influence of natural genetic variation on gene expression was studied by measuring the transcript levels of all genes of all strains, corrected for differential hybridization. An NJ tree was constructed based on the RNA hybridization data (Figure 2C). This tree showed that the genetic groups O, S1, S2, S3, and the out-group, were also separable based on gene expression level. Again, CB4856 and N2 differed from most of the other natural strains. Isolation site and genetic group influenced the variation in RNA levels of 6,930 and 7,996 genes, respectively (see Additional file 7). Most of these genes (77% and 78%) were not influenced by DNA polymorphisms (which were the cause of variation in 2,330 genes) or genotype (affecting 773 or 1,336 genes, depending on the genetic group or isolation site that was incorporated in the model).

### Expressed genes linked to isolation site are enriched for the gene classes clec, fbxa, bath, and nhr

Enrichment analyses were performed for the genes whose RNA levels were influenced by isolation site or genetic group (see Additional file 1, worksheets L–N). The gene classes clec, fbxa, bath, and nhr were significantly enriched, thus yielding similar results to our DNA-level enrichment analyses. In addition, several nematode-specific peptide families were also enriched.

Together, these results show that at the genomic level, variation between local populations is enriched for the gene classes fbox, math, bath, btb, clec, serpentine chemoreceptor, and nhr, many of which are involved in gene-environment interactions [42-45]. Interestingly, we found that the gene classes clec, fbxa, bath, and nhr were also enriched, with variation linked to isolation site on the transcriptional level, even though the strains that originated from different sites were cultured under the same conditions. It has been shown that many of these groups of genes are differentially expressed after pathogen exposure, and thus could be involved in the immune response. For instance, C-type lectin domain-containing proteins (CTLD proteins, gene class clec) have been repeatedly proposed to contribute to nematode immunity [46]. The immune function of these genes is supported by their specific upregulation in infected *C*. *elegans*[43,44,47-50] and also by reduced immune phenotypes after RNAi knockdown of *clec*-*70*, *clec*-*17*, *clec*-*60*, or *clec*-*86*[7,51]. Furthermore, F-box proteins (gene class fbxa) are part of the protein degradation pathway [52]. In this pathway, substrates for degradation are ubiquinated to be recognizable by the 26S proteasome. Taken together, these results show that local genetic diversity reflects site-specific signatures of immune response and protein degradation pathways in *C*. *elegans*. We also found that, in addition to genotypes, transcript profiles can be used to distinguish between local *C*. *elegans* populations, and may indicate the functional importance of the identified genes or gene classes in different environments [53].

### Polymorphic genes are enriched for genes involved in gene-environment interactions

Polymorphic genes between the populations were compared using a set of genes extracted from 19 different studies on gene expression in *C*. *elegans* exposed to biotic and abiotic factors (see Additional file 1, worksheet S). In the wild, *C*. *elegans* is exposed to many different bacteria. In studies on the effect on gene expression of various bacteria, such as *Lactobacillus rhamnosus*[54], *Microbacterium*. *nematophilum*[7], *Drechmeria coniospora*[55], *Serratia marcescens*[8], *Xenorhabdu*s *nematophila*[8], and *Pseudomonas aeruginosa*[56], c-type lectins were always found to be differentially expressed, as were in most cases the F-box protein genes. Receptors that are used to sense the environment, such as nhrs and serpentine receptors are also frequently differentially expressed when *C*. *elegans* is exposed to different bacteria. In response to abiotic factors such as temperature [12], osmotic stress [10] or ions [57,58], the c-type lectins and F-box protein genes are also always differentially expressed. Furthermore, the c-type lectins, F-box protein genes, and receptor genes are differentially expressed in the presence of various other substances that can be encountered by wild *C*. *elegans* strains, including tryptophan [59], β-naphthoflavone [60], H_2_S [61], fluoranthene [62], hormones [63], sediment [64], humic substances [65], and pesticides [12,66,67]. The other gene classes (bath, math, and btb) that are importaty for the variation seen between the locations at which the wild *C*. *elegans* strains were isolated were also found to be differentially expressed in several of the aforementioned environmental studies. Altogether, the differential expression of genes in environmental studies indicates that the genes that are important for the variation between local populations of *C*. *elegans* are indeed of significance for interactions with the environment.

### Local populations are separable for some fitness traits

The next question was whether the genetic polymorphisms between strains could influence fitness trait variation. *C*. *elegans* strains varied significantly in all traits except population size on *Escherichia coli* OP50 (Table 2). As all tests were performed, under standardized laboratory conditions and the variation between strains could be attributed to the genotype, showing that most phenotypic variation has a genetic basis. A genetic determinant has been found for some of these traits [5,13]. We found a significant influence of the genetic groups on the population size of *C. elegans* on *Bacillus thuringiensis* NRRL B-18BT247 and on the length/width ratio (see Additional file 1, worksheet O). We additionally reconstructed an NJ tree using phenotypic trait variation; however, phenotypic variation did not separate the two isolation sites or any of the four genetic groups. Nevertheless, some phenotypes were specific to an isolation site or to certain genetic groups. Even though the two strains with the largest length/width ratio were from Santeuil, most worms from Santeuil were significantly shorter, had a significantly smaller length/width ratio, and so were stouter than worms from Orsay (Table 2, see Additional file 8). In addition, the generation time of worms from Santeuil was significantly shorter (Table 2) (more details can be found in Additional file 1, worksheet O).

**Table 2:** Analysis of phenotypic variation between strains (ANOVA) and between sites

**Phenotype**	**Populations per genotype. N**^**a**^	**Mean (SD)**	**ANOVA (strain)**	**Mean (SD)**		***t*-test**
				**Orsay**	**Santeuil**	
Population size						
*Escherichia coli*	6	3.136 ± 695	0.3602	3278 ± 670	2988 ± 707	0.19
DSM	6	3.385 ± 750	4.83 × 10^-4^^b^	3369 ± 702	3402 ± 815	0.89
BT247	6	44 ± 39	<2.2 × 10^-16^^b^	53 ± 49	34 ± 21	0.12^c^
Development time, days	2 to 5	1.79 ± 0.07	4.16 × 10^-4^^b^	1.79 ± 0.07	1.79 ± 0.07	0.74
Generation time, days	2 to 5	1.98 ± 0.08	3.13 × 10^-6^^b^	1.98 ± 0.077	1.97 ± 0.77	0.019^b^
Embryogenesis, hours	2 to 5	4.60± 0.87	NA	4.35 ± 0.70	4.85 ± 0.97	0.031^b^
Length, μm	2 to 6	1.089 ± 58	4.83 × 10^-5^^b^	1107 ± 33	1070 ± 72	0.023^b^
Width, μm	2 to 6	43.96 ± 2.63	1.33 × 10^-9^^b^	44.45 ± 1.99	43.46 ± 3.13	0.35
Volume, nl	2 to 6	1.67 ± 0.24	1.12 × 10^-6^^b^	1.73 ± 0.19	1.61 ± 0.28	0.99
Length/width ratio	2 to 6	24.79 ± 0.82	1.19 × 10^-6^^b^	24.94 ± 0.79	24.64 ± 0.83	1.40 × 10^-5^^b^

*Abbreviations*: *BT247* nematocidal *Bacillus thuringiensis* NRRL B-18247, *DSM* non-nematocidal *Bacillus thuringiensis* DSM-350, *NA* not applicable.

^a^Describes the number of replicate populations of worms (each population more than 100 worms) which were tested per genotype, thus the number of such replicate populations (N) per isolation site is therefore 20 times higher than the replicate populations per genotype (as we have 20 genotypes per isolation site).

^b^Significant.

^c^When all observations per genotype were used instead of the mean per genotype, the populations from the different isolations sites were significantly different (*P*<0.0014).

### Local populations are separable with regard to food preference

**Figure 4:**
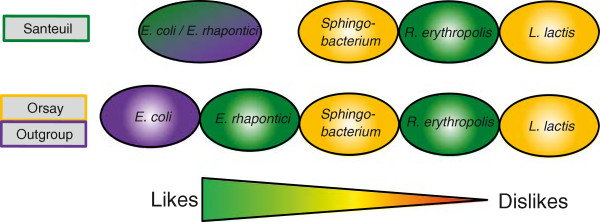
**Preference of the strains from the different origins for the different bacteria.** Strains are shown by rectangles (left), and bacteria by ellipses (right). Bacteria from Orsay are shown in orange, bacteroa frp, Santeuil strains in green, and standard laboratory food OP50 in purple.

We then investigated if the wild strains differed from each other in their food preference behavior for naturally co-existing bacteria and for *E*. *coli*. Under the laboratory conditions we used, the worms preferred *E*. *coli* OP50 over all other bacteria, followed by *Erwinia rhapontici*, *Sphingobacterium* sp., *Rhodococcus erythropolis*, and *Lactococcus lactis* (Figure 4; see Additional file 1, worksheet P). Worms from Santeuil preferred *E*. *rhapontici* (isolated from Santeuil) equally to *E*. *coli*., whereas worms from Orsay preferred *E*. *coli* over *E*. *rhapontici*. This suggests that Santeuil worms could have a slight preference for the bacterium species with which they are more likely to be familiar for an overview of the average preference of all strains, see Additional file 9B; for the complete dataset, see Additional file 1, worksheet Q (Wormcount and Choice Index) and see Additional file 1, worksheet R (Significances). The most significant differences found between the Orsay and Santeuil strains were for the bacterial combinations *E*. *coli* OP50/*E*. *rhapontici*, *E*. *coli* OP50/*R*. *erythropolis*. and *E*. *rhapontici*/*Sphingobacterium* sp. (Figure 4).

### The canonical strains Bristol N2 and CB4856 are genetic outliers

We finally investigated how far the canonical wild types Bristol N2 and CB4856 relate to the recently isolated natural strains. Both genotypic and transcriptomic analyses identified Bristol N2 and CB4856 as clearly differing from the standing genetic variation of the wild isolated strains. This also applied when we compared N2 and CB4856 with some of the other out-group strains. It is assumed that many alleles in CB4856 and N2 are laboratory-derived because both N2 and CB4856 went through multiple phenotypic and genetic bottlenecks over the past decades of laboratory maintenance [1,68]. Together with our results, this shows that the genotype-phenotypes relations in both N2 and CB4856 are likely to be obscured by a number of laboratory-derived alleles with large effect. This might impede the detection and functional analysis of many other genes and alleles that by themselves have small effects on phenotypes, but together might have a large effect.

## Conclusions

We measured a large variety of phenotypes, including the transcriptome, for multiple wild genotypes in *C*. *elegans* collected from different locally separated sites, under the same and constant laboratory conditions. The wild genotypes could be classified according to their site, based on genotypic and transcriptome analyses. These differences were also reflected in several fitness traits; however, because of the limited number of populations sampled, we were not able to associate fitness traits to the different sites. Nevertheless our data provide the basis for uncovering site-specific genotypic and phenotypic signatures. Future work should aim to provide insight into genetic drift or adaptation as the major attribute shaping *C*. *elegans* local evolution. Most likely, both processes play a role, depending on the gene or genetic element in question. However, for some gene classes, such as the chemoreceptors, it is tempting to think they are polymorphic as a result of adaptations to specific habitats.

In summary, we have unlocked a wealth of genotype-phenotype relations, indicating that the canonical wild type is a genetic outlier and that its genotype-phenotype characteristics represent a diminutive part of the myriad of interactions present in the wild.

## Methods

### Nematode and bacterial strains

The main set of strains of *C*. *elegans* comprised 41 new strains that were isolated (by M-A Félix) from two different locations in France (Orsay and Santeuil). The out-group comprised three new strains isolated in the Netherlands, two strains previously isolated in France, and the most diverse canonical strains N2 (Bristol) and CB4856 (Hawaii) [16,18-21,23-29,67] (see Additional file 1, worksheet A for details). All strains were routinely maintained on nematode growth medium (NGM) with *E. coli* OP50 as a food source [69]. *E*. *coli* OP50 was used in all experiments, except for the population growth experiment, in which *B. thuringiensis* NRRL B-18247 and *B*. *thuringiensis* DSM-350 were used next to *E. coli*[70]. In the food preference experiment, in addition to *E. coli* OP50, *E. rhapontici* and *R.s erythropolis* (both isolated from and unique for Santeuil), and *L. lactis* and *Sphingobacterium* sp. (both isolated from and unique for Orsay) were used (all bacteria were isolated and identified by M-A. Félix and B. Samuel).

### Genomic DNA analysis: worm culturing, DNA isolation, DNA microarrays, and statistical analysis

Gene expression microarrays (*C*. *elegans* (V2) Gene Expression Microarray 4X44K slides; Agilent Technologies, Santa Clara, CA, USA) were used to co-hybridize N2 versus wild-type DNA, allowing for analysis of population differences based on gene polymorphisms. Fresh populations of mixed stages were cultured for 96 hours at 20°C before sampling. All procedures were performed as recommended by the manufacturer (Agilent; Oligonucleotide Array-Based CGH for Genomic DNA Analysis; Enzymatic Labeling for Blood, Cells or Tissues (with a High Throughput Option) protocol, version 6.3). Genomic DNA isolation was performed with a commercial kit (NucleoSpin Tissue Kit; Machery-Nagel, Düren, Germany). For processing the data of the DNA microarrays, the limma package for the R software environment was used [71]. Background correction was performed using the Substract method. LOESS (locally weighted regression scatter plot smoothing) within-array normalization and scale between-array normalization were used to process the raw intensity values [72]. Genotypes were compared by calculating, per spot, the ratio of the intensities of each strain with the mean intensity over all strains. Genes with a ratio of greater than 0.5 or less than −0.5 were considered polymorphic. PCA was performed using the polymorphic genes from all strains. The unrooted NJ tree was compiled from a distance matrix made from the ratios of the polymorphic genes with the R package phangorn [73]. Linear models were used to calculate the significance of the variation in DNA hybridization intensities linked to the isolation sites and the identified genetic groups. The model used to determine linkage to isolation site was:

$$I_{x}∼L_{x}+E_{x}$$

where I is the DNA hybridization intensity of genotype x, L is the isolation site (out-group site, Orsay, or Santeuil) and E is the error. For linkage to Santeuil we took –log10(p) of 2.3 as threshold, while for linkage to Orsay we took –log10(p) of 2.7 as threshold. For linkage to genetic group the model used to determine linkage to isolation site was:

$$I_{x}∼G_{x}+E_{x}$$

where I is the DNA hybridization intensity of genotype x, G is the genetic group (out-group, S1, S2, or S3) and E is the error. The thresholds used were –log10(p) 2.5, 2.3, 3.2, and 3.3 for O, S1, S2, and S3 respectively (for the number of genes per genetic group and overlapping genes (see Additional file 5B). The significance thresholds, adjusted for multiple testing, were determined by permutation, for which the same model was used, with the spot intensities randomly distributed over the genotypes (a *P* value that gave a ratio of false positives/true positives of <0.05 was used).

### mRNA analysis: culturing, isolation, RNA-microarrays, and statistical analysis

For the mRNA microarrays, any males were discarded and only hermaphrodites grown on *E*. *coli* OP50 were used. Two independent replicates of each strain (synchronized late L4 larvae) were analyzed. For mRNA isolation, a commercial kit (RNEasy Micro Kit; Qiagen, Hilden, Germany) was used, following the manufacturer’s protocol (Purification of Total RNA from Animal and Human Tissues) provided with the kit, with modified lysing procedure (see Additional file 10). The microarrays used were as described above (*C*. *elegans* (V2) Gene Expression Microarray 4X44K slides; Agilent) following the manufacturer’s instructions. For processing of the RNA microarray data, the limma package for the R software environment was used. No background correction was performed, as recommended previously [71]. For within-array normalization, the LOESS method was used and for between-array normalization, the quantile method was used. Expression variation was determined by linear models. The variation in intensities could be explained by batch, DNA hybridization, genetic group, and genotype (see also the paragraph on statistics in the Genomic DNA Analysis section). Significance thresholds, adjusted for multiple testing, were determined by permutations of all spots on the array. In the permutations, the RNA hybridization intensities were randomly distributed over the genotypes and batches (the *P*-value that gave a ratio of false positives/true positives of <0.05 was used).

### Enrichment analysis

All enrichment analyses were performed using a hypergeometric test. The number of genes selected by a criterion in this paper (for example, linked to a genetic group) were compared with the genes with a specific annotation (for example, c-type lectin). The chance that a number of genes will be overlapping depends on the total group size, the number of genes selected, and the number of genes with a specific annotation. This chance, together with the number of overlapping genes, can be used in a hypergeometric test. Annotation groups were considered enriched when the overlap was more than three genes and the significance –log10(p) was greater than 2.5.

Polymorphic genes between populations were compared with a set of differentially expressed genes extracted from a diverse set of gene-environment interaction studies in *C*. *elegans*. All enrichment analyses were performed using a hypergeometric test.

### Phenotypic assays

#### Development time and generation time

L1 juveniles fed with *E*. *coli* OP50 were incubated at 24°C and inspected at regular time intervals. Development time was defined as the period between worm inoculation and the moment at which the first worms with open vulva were seen. Generation time was the period between inoculation and the first appearance of eggs.

#### Length and width

Analysis of length and width of young gravid worms was performed with a particle analyzer (RapidVue; Beckman Coulter Inc., Miami, FL, USA). In total, 2000 worms per strain were measured.

#### Population growth

To measure population growth, 10 single L4 worms were placed onto a bacterial lawn, and cultured at 20°C. After 96 hours, the number of worms on the plate was counted.

#### Food preference assay

To test the food preference of the worms, 5 μl drops of two different bacteria were placed on NGM in each well of a 12-well plate (see Additional file 1, worksheet P). A drop with juvenile nematodes up to he stage of L2 was then added to each well, and the plate was incubated overnight at 20°C. The worms on each bacterium were then counted and the Choice Index was calculated [74].

#### Statistics

We used ANOVA to calculate the influence of strain/genotype on the phenotypic variation, by regressing the individual measurements over the strains/genotypes. We used a two-sided *t*-test, assuming unequal variance to determine if phenotypes were significantly different between isolation sites. ANOVA was used to determine if phenotypes were significantly different between genetic groups.

### Microsatellite analysis

Population genetic differentiation was assessed using six microsatellite loci (see Additional file 1, worksheet C), which we previously identified to be highly variable in both natural and experimental *C*. *elegans* populations ([36] and see Additional file 10 for details).

### Data storage

Microarray data (both RNA and DNA) can be found at [23].

## Competing interests

The authors declare that they have no competing interests.

## Authors’ contributions

RJMV carried out the RNA and DNA arrays, and drafted the manuscript; LBS designed and carried out all statistical analyses except for the microsatellite study, and drafted the manuscript; CJHH carried out the food preference assays; RC carried out the developmental time, generation time, length, and width measurements; WC carried out the population growth measurements and generated the microsatellite data; WY helped with the enrichment analysis of the studies on gene expression; MGS helped to design the statistical analyses and to draft the manuscript; and HS carried out the statistical analysis of the microsatellite study. HS, BPB, and JEK conceived of the study and participated in its design and coordination, and helped to draft the manuscript. All authors read and approved the final manuscript.

## Supplementary Material

Additional file 1 Various supplementary datasets. Click here for file

Additional file 2 **Detailed overview of DNA hybridization differences.** Chromosome number is stated at the top of each page. Wild isolates from Orsay are shown in orange, wild isolates form Santeuil in green, and the out-group strains in purple. On the *y*-axis, the log2 ratio of the individual lines with the value of N2 per microarray probe is shown as dot, the moving average (nine probes) is shown as lines, and the threshold for the moving average is shown as horizontal red lines. Probe positions are indicated by the triangles on the *x*-axis, with the names of genes with a ratio outside the thresholds shown in the figure. The lines are drawn to the start of the gene on the genome. Click here for file

Additional file 3 **Genome-wide overview of DNA hybridization differences per chromosome.** The chromosome number is stated at the top of each page. Wild isolates from Orsay are shown in orange, wild isolates from Santeuil in green, and the out-group strains in purple. On the *y*-axis, the log2 ratio of the individual lines with N2 is shown as dots, and the thresholds for the moving averages are shown as horizontal red lines. Click here for file

Additional file 4 **Number of polymorphic genes per chromosome.** The wide part of the bars shows the number of genes with an absolute ratio greater than 1, while the narrow part shows the number of genes with an absolute ratio greater than 0.5. Total number of genes per chromosome (with percentage of polymorphic genes (ratio >0.5) in parentheses): chromosome *I*, 2,969 genes (28%); *II*: 3,588 (32%); *III*: 2,680 (28%); *IV*: 3,435 (30%); *V*: 5,400 (35%); *X*: 2,809 (24%). Click here for file

Additional file 5 **Minimum spanning network and Venn diagram. (A)** Minimum spanning network constructed using microsatellite data. The letter ‘O’ and red color refers to Orsay; S and blue to Santeuil. Circle size is proportional to the number of strains with a particular genotype. The solid lines show the main relationships among genotypes, while the dotted lines show alternative connections. Line length correlates with the inferred number of evolutionary differences. The minimum spanning network was reconstructed with the software program Arlequin. **(B)** Venn diagram of genes for which DNA hybridization intensity per genetic group was significantly different from that of the the out-group. Total number of genes for each strain: Orsay: 1,933; Santeuil 1: 3,181; Santeuil 2: 737; Santeuil 3: 567. Group S1 appeared to be the most divergent from the out-group, with 3,181 genes that differed significantly. A large part of these genes (803) was also shared with the Orsay group. The number of significantly different genes that were the same for S3 and S2 or O (4 in both cases) and for S2 and O (1 gene) was remarkably low. S2 and O also shared a small number (n =19) of the same significantly different genes. Click here for file

Additional file 6 **Polymorphic genes and genes linked or not linked to isolation sites. (A)** Schematic overview of the groups of polymorphic genes based on DNA hybridization data. **(B)** Percentage of all genes detected using hybridization of genomic *C*. *elegans* DNA on microarrays that were linked or not linked to the isolation sites. Together, the gene classes serpentine receptors, F-box, math, bath, btb, clec, and nhr composed almost 25% of the polymorphic genes significantly linked to isolation site. These same gene classes made up less than 10% of the genes that could not be linked to isolation site. Click here for file

Additional file 7 **Venn diagram of the genes that showed expression differences due to DNA polymorphisms and genotype in combination with either genetic group (O, S1, S2, and S3) or isolation site (Santeuil and Orsay).** Total number of genes: genotype (left diagram): 773; genotype (right diagram): 1,336; DNA: 2,230; genetic group: 7,996; isolation site: 6,930. Click here for file

Additional file 8 **Phenotypes of the wild isolates.** Strains from Orsay are shown in orange, strains from Santeuil in green, and the out-group strains in purple. The right panel shows the statistics, mean and standard deviation (SD) as well as the *P*-value of the *t*-test of the phenotypic difference between the Orsay and Santeuil groups with and without outliers removed. When applicable, an ANOVA on strain was performed, and the *P*-value is shown. Lastly, the heritability (H2) was calculated. Labels on the y-axis refer to the phenotypes described in Table 2. Click here for file

Additional file 9 **Food preference assay. (A)** Set-up of the food preference assay and the calculation of the Choice Index (CI). A, bacterium A; B, bacterium B. **(B)** Schematic overview of the results of the food preference assay. Green indicates bacteria isolated in Santeuil, orange indicates bacteria isolated in Orsay, purple indicates standard laboratory food OP50. All numbers are percentages and are the average of all strains in the experiment. The percentages near the bacteria indicate the fraction of worms that prefer that particular bacterium when tested together with the bacterium at the opposite end of the line. The percentages in or near the yellow circles indicate the fraction of worms that did not choose between the two bacteria. For example, when offered a choice between *Sphingobacterium* and *Lactococcus lactis*, on average 85.7% of all worms of all strains preferred *Sphingobacterium*, 5.5% preferrred *L*. *lactis*, and 8.8% did not make a choice between these bacteria. Click here for file

Additional file 10 Detailed description of Methods. Click here for file
