# Supplementary material for: Gene-environment and protein-degradation signatures characterize genomic and phenotypic diversity in wild Caenorhabditis elegans populations
Source: BMC Biol. 2013 Aug 19;11:93. doi: 10.1186/1741-7007-11-93 (PMC3846632; doi:10.1186/1741-7007-11-93)
Supplement: Additional file 2 — Detailed overview of DNA hybridization differences. Chromosome number is stated at the top of each page. Wild isolates from Orsay are shown in orange, wild isolates form Santeuil in green, and the out-group strains in purple. On the y-axis, the log2 ratio of the individual lines with the value of N2 per microarray probe is shown as dot, the moving average (nine probes) is shown as lines, and the threshold for the moving average is shown as horizontal red lines. Probe positions are indicated by the triangles on the x-axis, with the names of genes with a ratio outside the thresholds shown in the figure. The lines are drawn to the start of the gene on the genome. [file 1741-7007-11-93-S2.pdf]

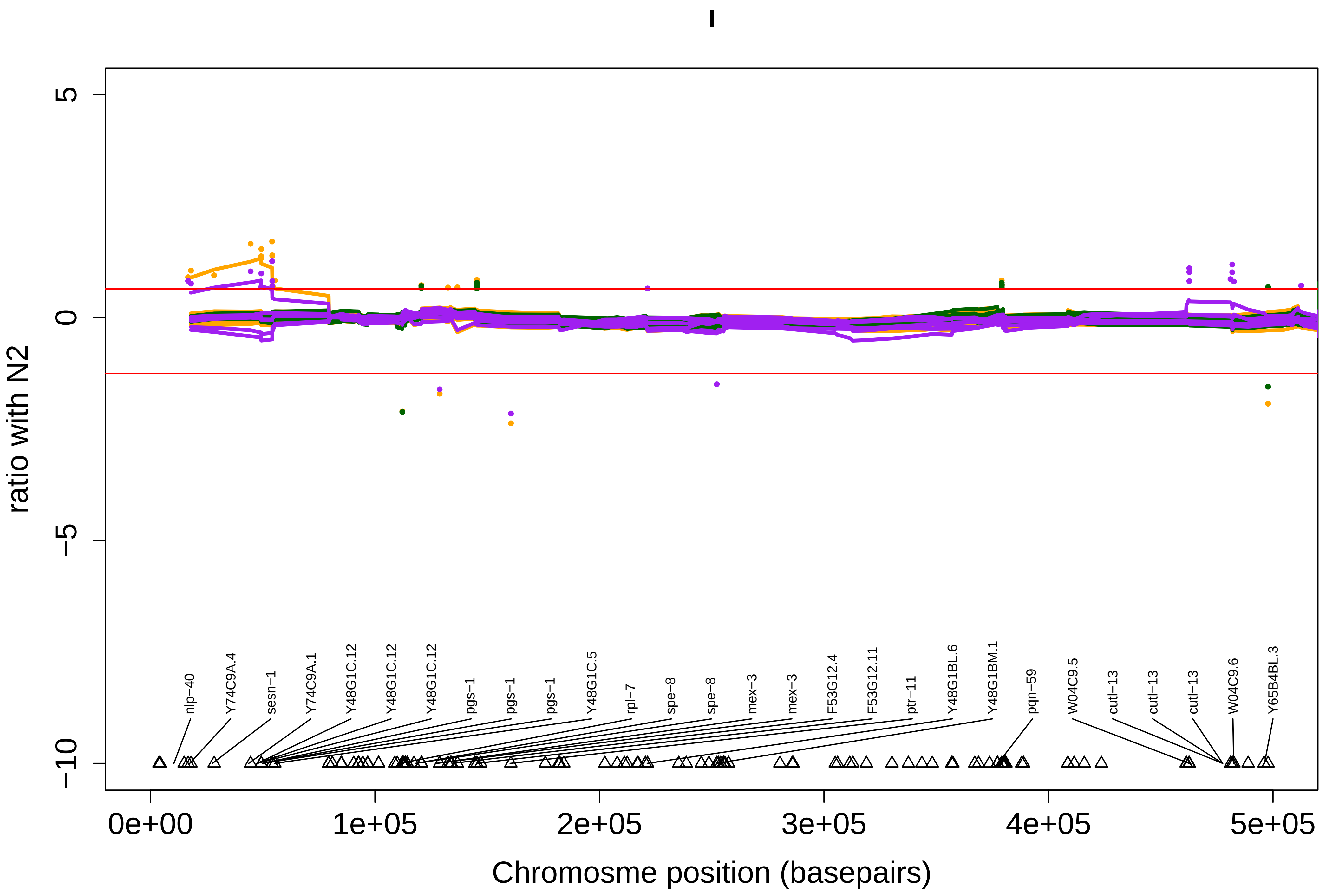
I
ratio with N2
5
0
−5
−10
0e+00
1e+05
2e+05
3e+05
4e+05
5e+05
Chromosme position (basepairs)
nlp−40
Y74C9A.4
sesn−1
Y74C9A.1
Y48G1C.12
Y48G1C.12
Y48G1C.12
pgs−1
pgs−1
pgs−1
Y48G1C.5
rpl−7
spe−8
spe−8
mex−3
mex−3
F53G12.4
F53G12.11
ptr−11
Y48G1BL.6
Y48G1BM.1
pqn−59
W04C9.5
cutl−13
cutl−13
cutl−13
W04C9.6
Y65B4BL.3

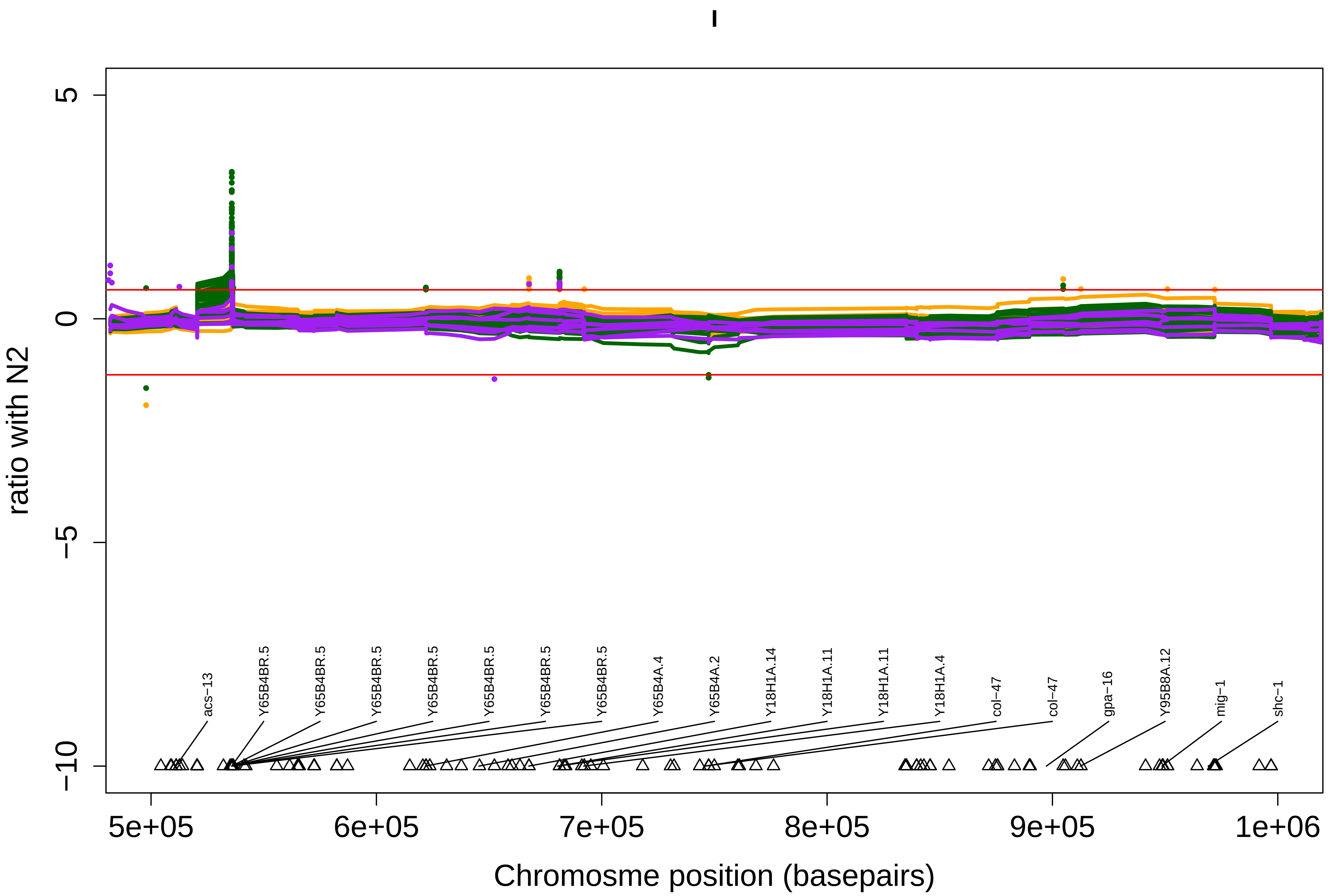
I
ratio with N2
5
0
−5
−10
5e+05
6e+05
7e+05
8e+05
9e+05
1e+06
Chromosme position (basepairs)
acs−13
Y65B4BR.5
Y65B4BR.5
Y65B4BR.5
Y65B4BR.5
Y65B4BR.5
Y65B4BR.5
Y65B4BR.5
Y65B4A.4
Y65B4A.2
Y18H1A.14
Y18H1A.11
Y18H1A.11
Y18H1A.4
col−47
col−47
gpa−16
Y95B8A.12
mig−1
shc−1

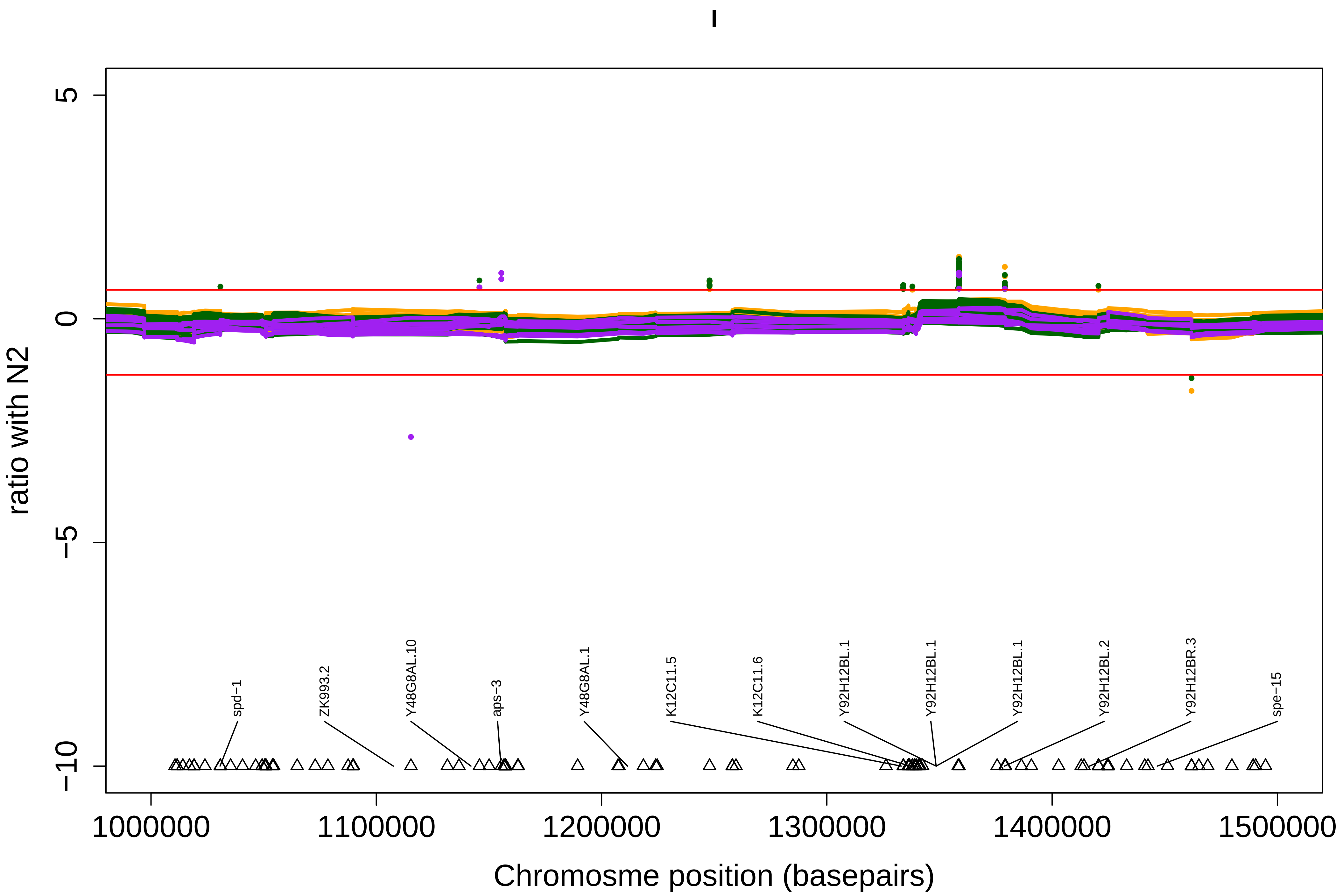

I
ratio with N2
5
0
−5
−10
spd−1
ZK993.2
Y48G8AL.10
aps−3
Y48G8AL.1
K12C11.5
K12C11.6
Y92H12BL.1
Y92H12BL.1
Y92H12BL.1
Y92H12BL.2
Y92H12BR.3
spe−15
1000000
1100000
1200000
1300000
1400000
1500000
Chromosme position (basepairs)

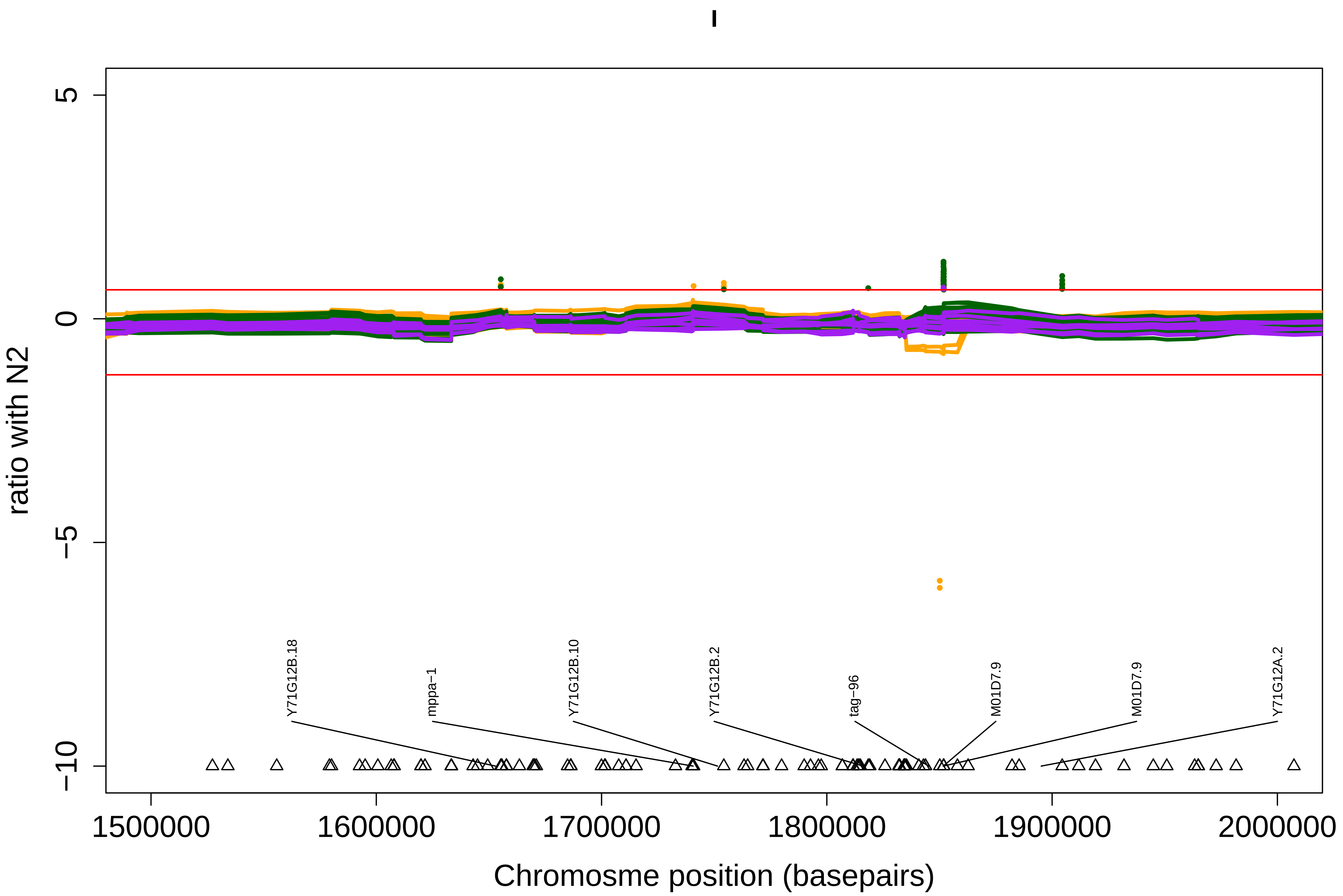
I
ratio with N2
5
0
−5
−10
Y71G12B.18
mppa−1
Y71G12B.10
Y71G12B.2
tag−96
M01D7.9
M01D7.9
Y71G12A.2
1500000
1600000
1700000
1800000
1900000
2000000
Chromosme position (basepairs)

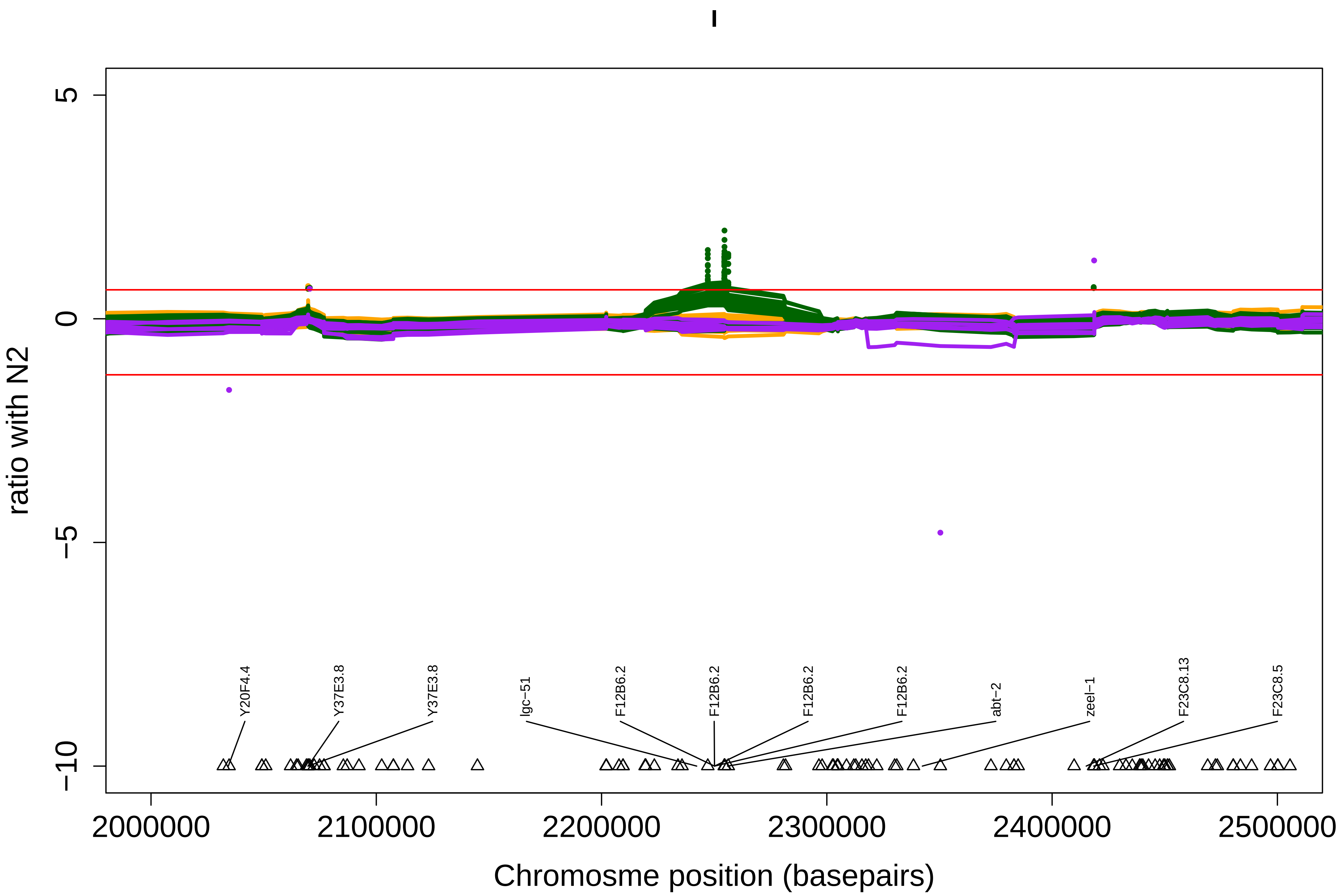
I
ratio with N2
5
0
−5
−10
Y20F4.4
Y37E3.8
Y37E3.8
lgc−51
F12B6.2
F12B6.2
F12B6.2
F12B6.2
abt−2
zeel−1
F23C8.13
F23C8.5
2000000
2100000
2200000
2300000
2400000
2500000
Chromosme position (basepairs)

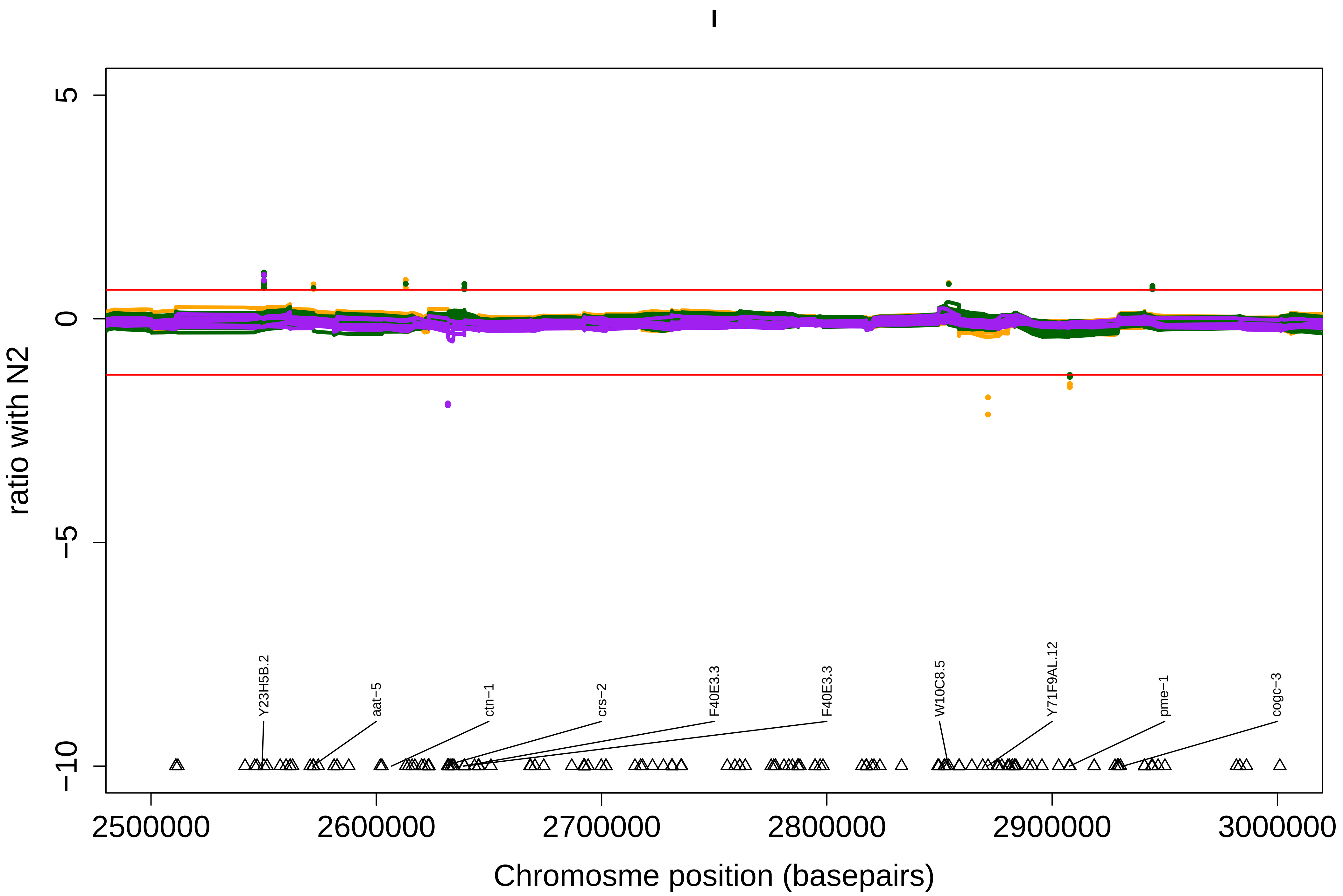
I
ratio with N2
5
0
−5
−10
Y23H5B.2
aat−5
ctn−1
crs−2
F40E3.3
F40E3.3
W10C8.5
Y71F9AL.12
pme−1
cogc−3
2500000
2600000
2700000
2800000
2900000
3000000
Chromosme position (basepairs)

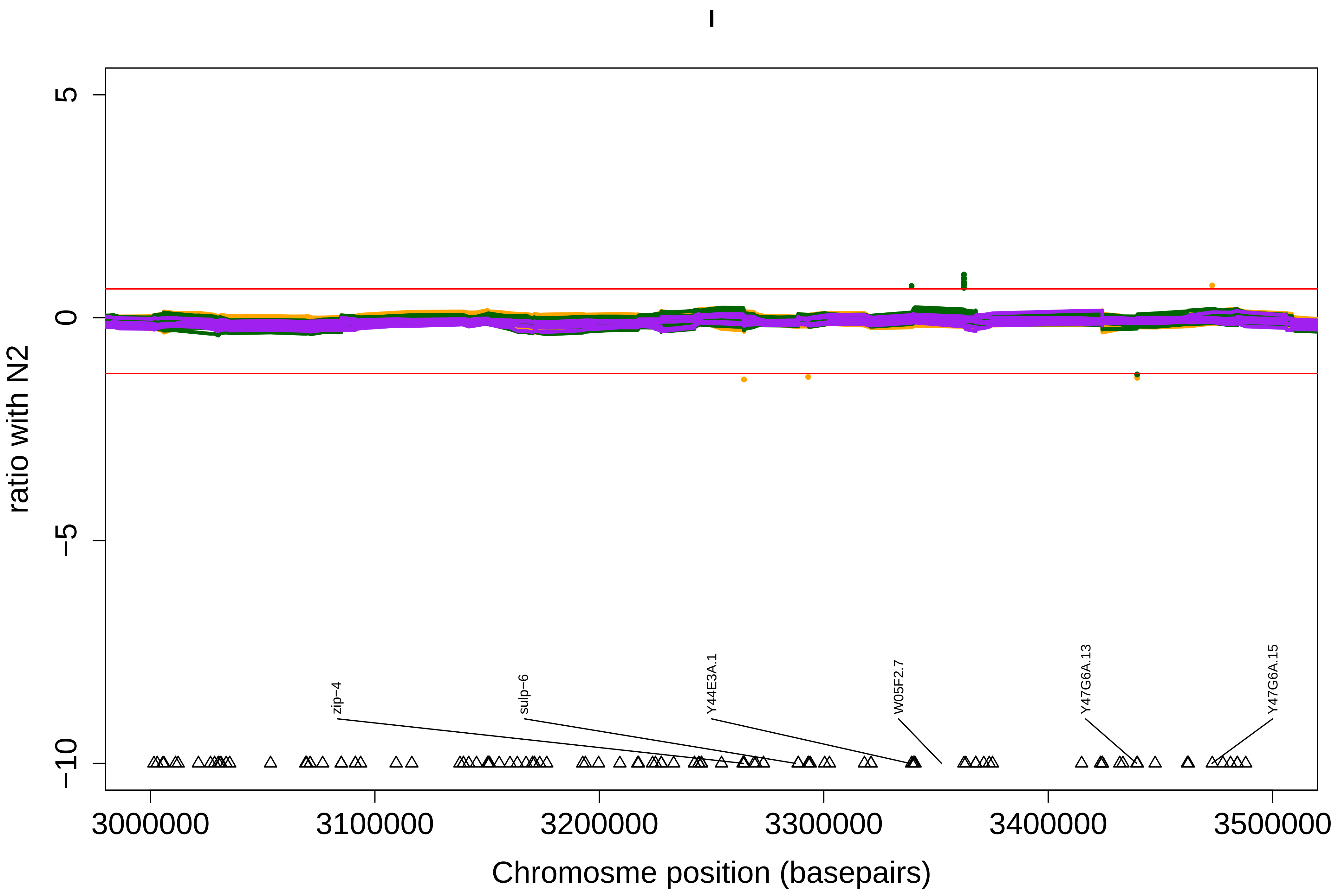
I
ratio with N2
5
0
−5
−10
Chromosme position (basepairs)
3000000
3100000
3200000
3300000
3400000
3500000
zip−4
sulp−6
Y44E3A.1
W05F2.7
Y47G6A.13
Y47G6A.15

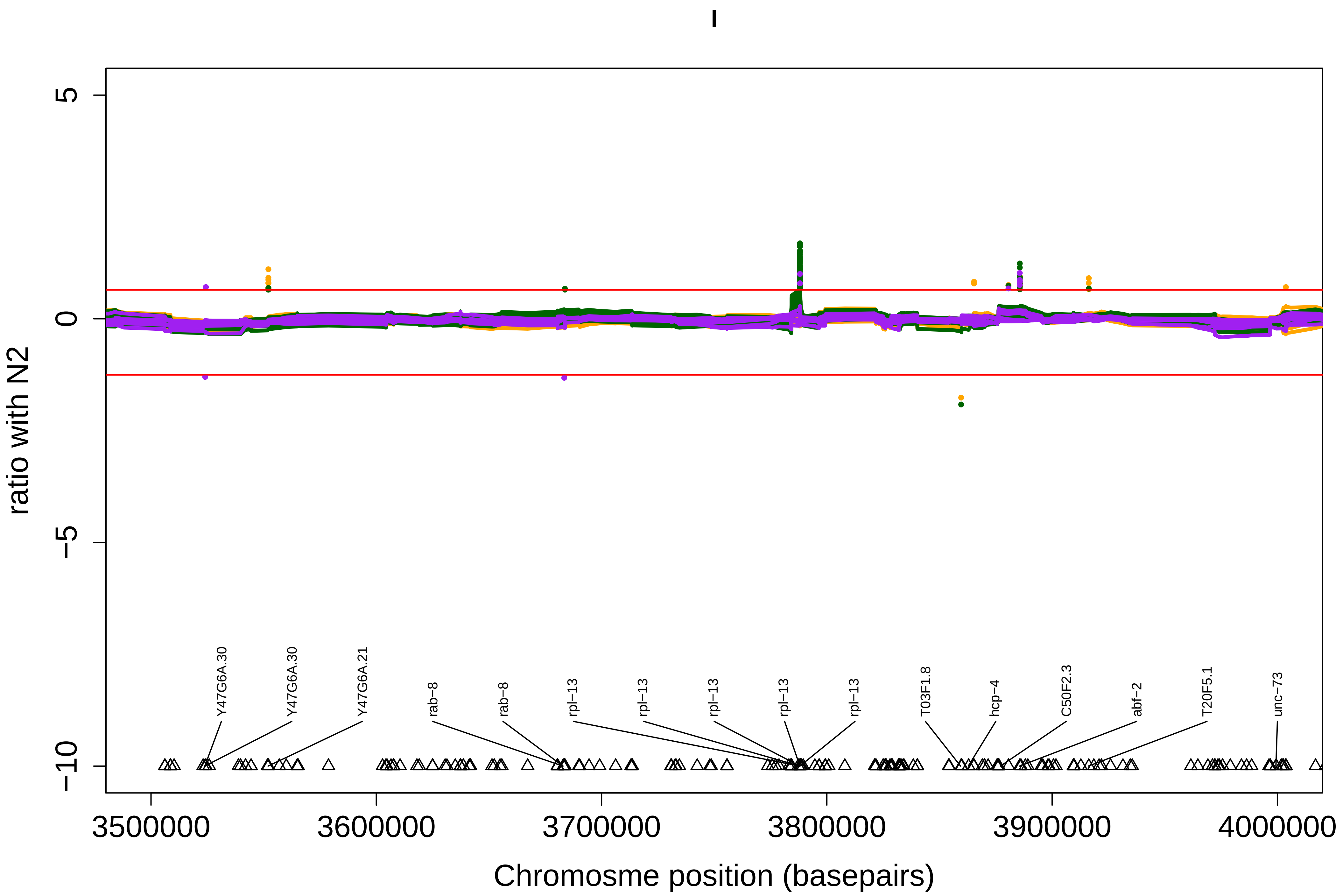
I
ratio with N2
5
0
−5
−10
Y47G6A.30
Y47G6A.30
Y47G6A.21
rab−8
rab−8
rpl−13
rpl−13
rpl−13
rpl−13
rpl−13
T03F1.8
hcp−4
C50F2.3
abf−2
T20F5.1
unc−73
3500000
3600000
3700000
3800000
3900000
4000000
Chromosme position (basepairs)

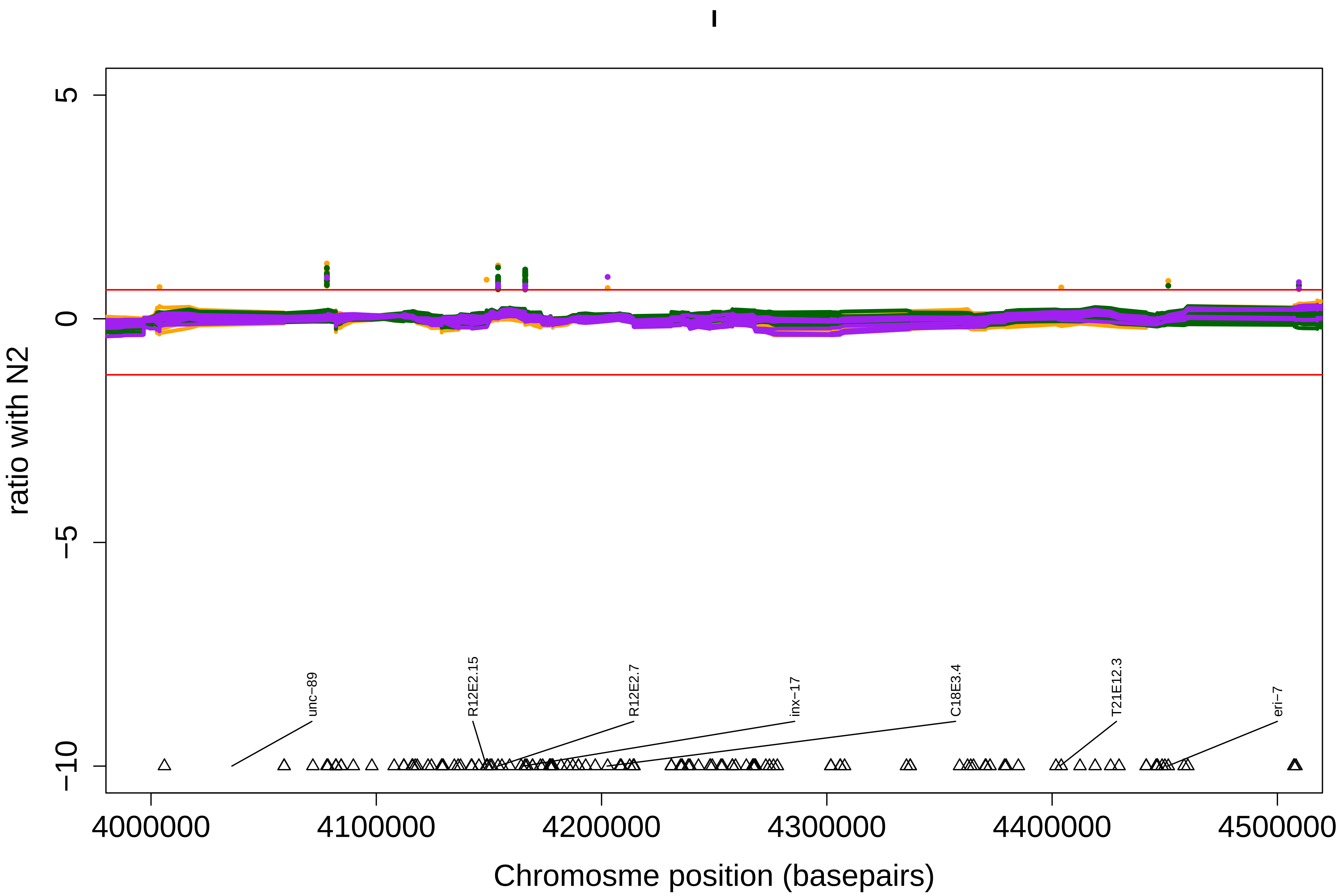
I
ratio with N2
5
0
−5
−10
unc−89
R12E2.15
R12E2.7
inx−17
C18E3.4
T21E12.3
eri−7
4000000
4100000
4200000
4300000
4400000
4500000
Chromosme position (basepairs)

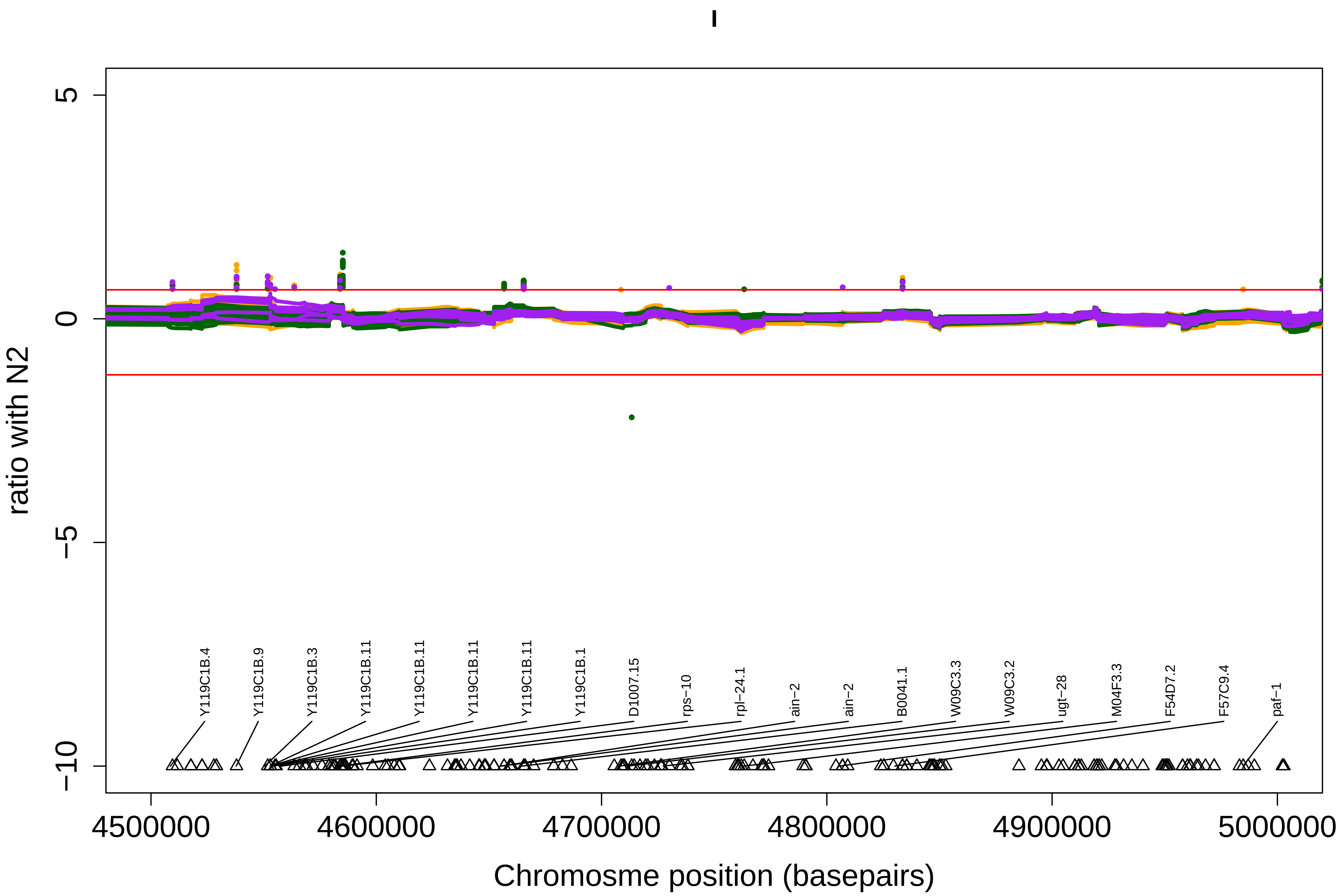

I
ratio with N2
5
0
−5
−10
Y119C1B.4
Y119C1B.9
Y119C1B.3
Y119C1B.11
Y119C1B.11
Y119C1B.11
Y119C1B.11
Y119C1B.1
D1007.15
rps−10
rpl−24.1
ain−2
ain−2
B0041.1
W09C3.3
W09C3.2
ugt−28
M04F3.3
F54D7.2
F57C9.4
paf−1
4500000
4600000
4700000
4800000
4900000
5000000
Chromosme position (basepairs)

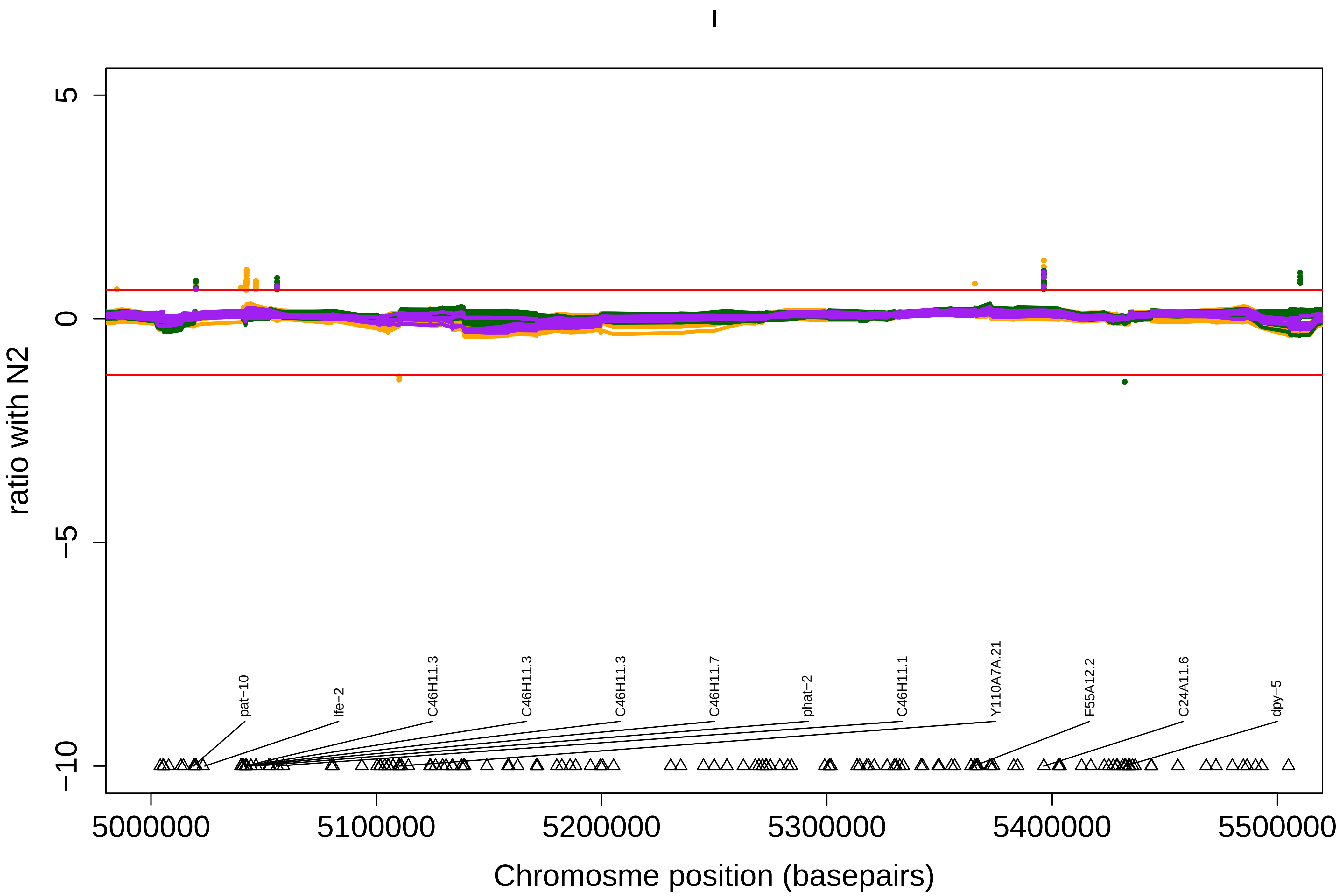

I
ratio with N2
5
0
−5
−10
pat−10
lfe−2
C46H11.3
C46H11.3
C46H11.3
C46H11.7
phat−2
C46H11.1
Y110A7A.21
F55A12.2
C24A11.6
dpy−5
5000000
5100000
5200000
5300000
5400000
5500000
Chromosme position (basepairs)

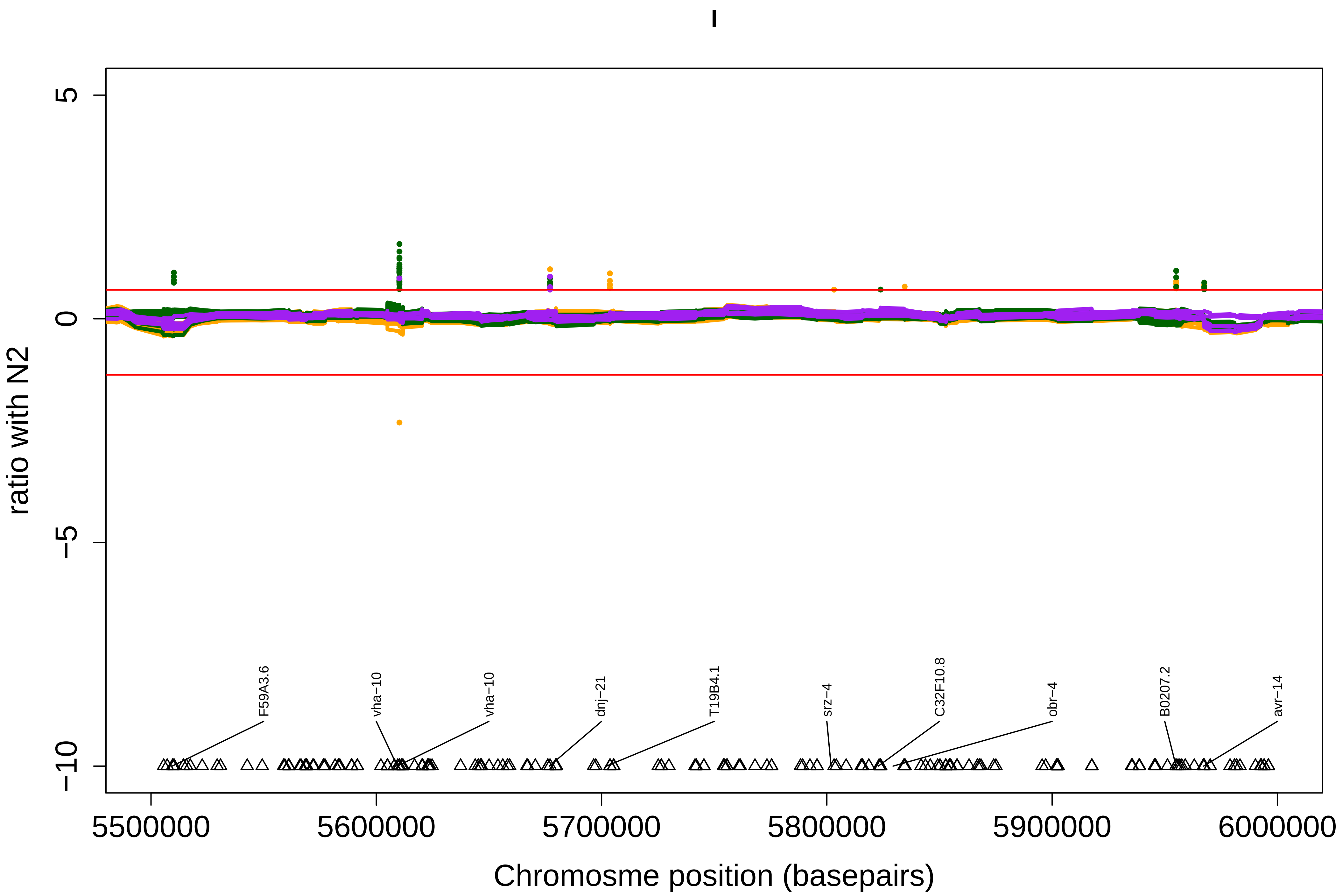
I
ratio with N2
5
0
−5
−10
F59A3.6
vha−10
vha−10
dnj−21
T19B4.1
srz−4
C32F10.8
obr−4
B0207.2
avr−14
5500000
5600000
5700000
5800000
5900000
6000000
Chromosme position (basepairs)

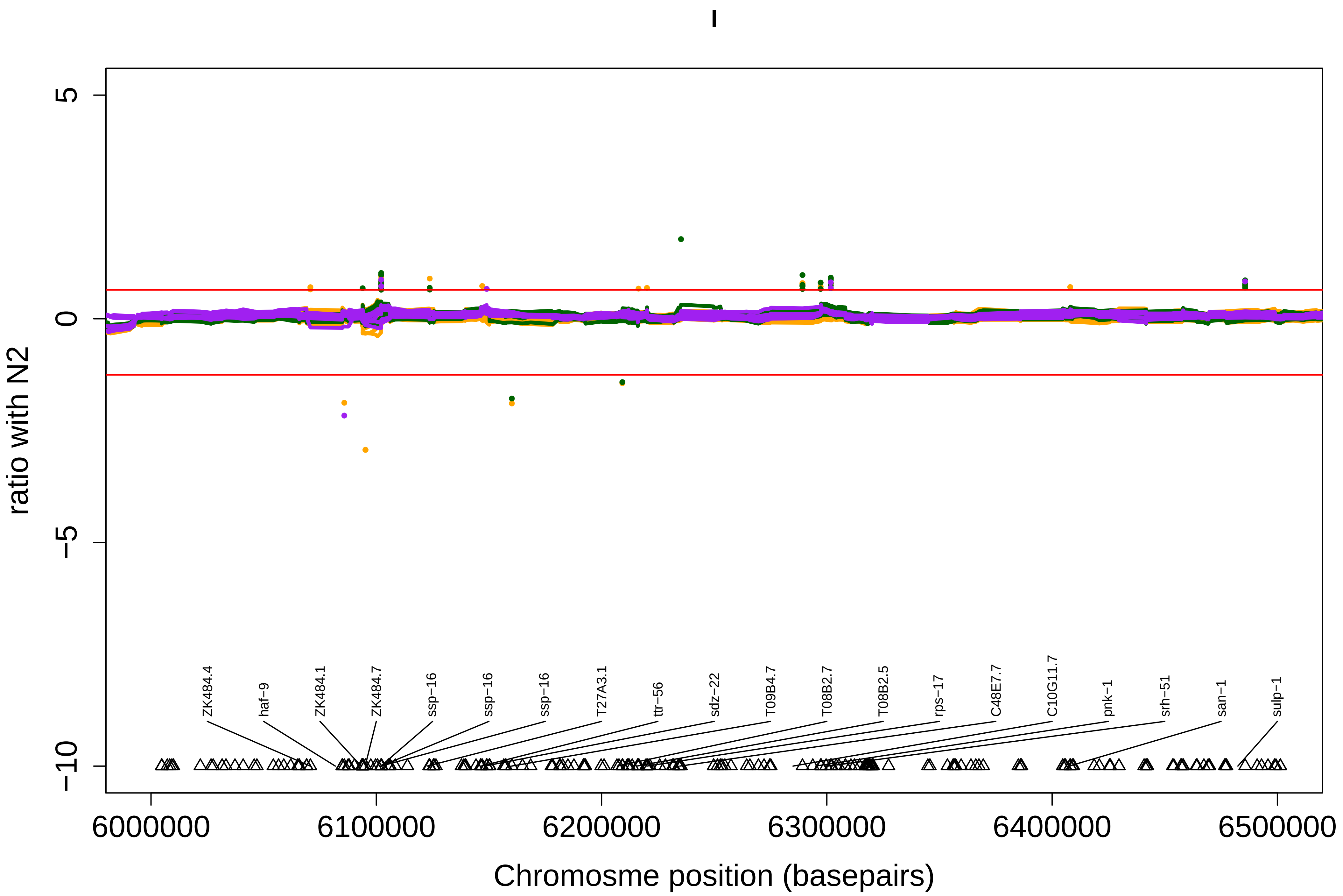

I
ratio with N2
5
0
−5
−10
ZK484.4
haf−9
ZK484.1
ZK484.7
ssp−16
ssp−16
ssp−16
T27A3.1
ttr−56
sdz−22
T09B4.7
T08B2.7
T08B2.5
rps−17
C48E7.7
C10G11.7
pnk−1
srh−51
san−1
sulp−1
6000000
6100000
6200000
6300000
6400000
6500000
Chromosme position (basepairs)

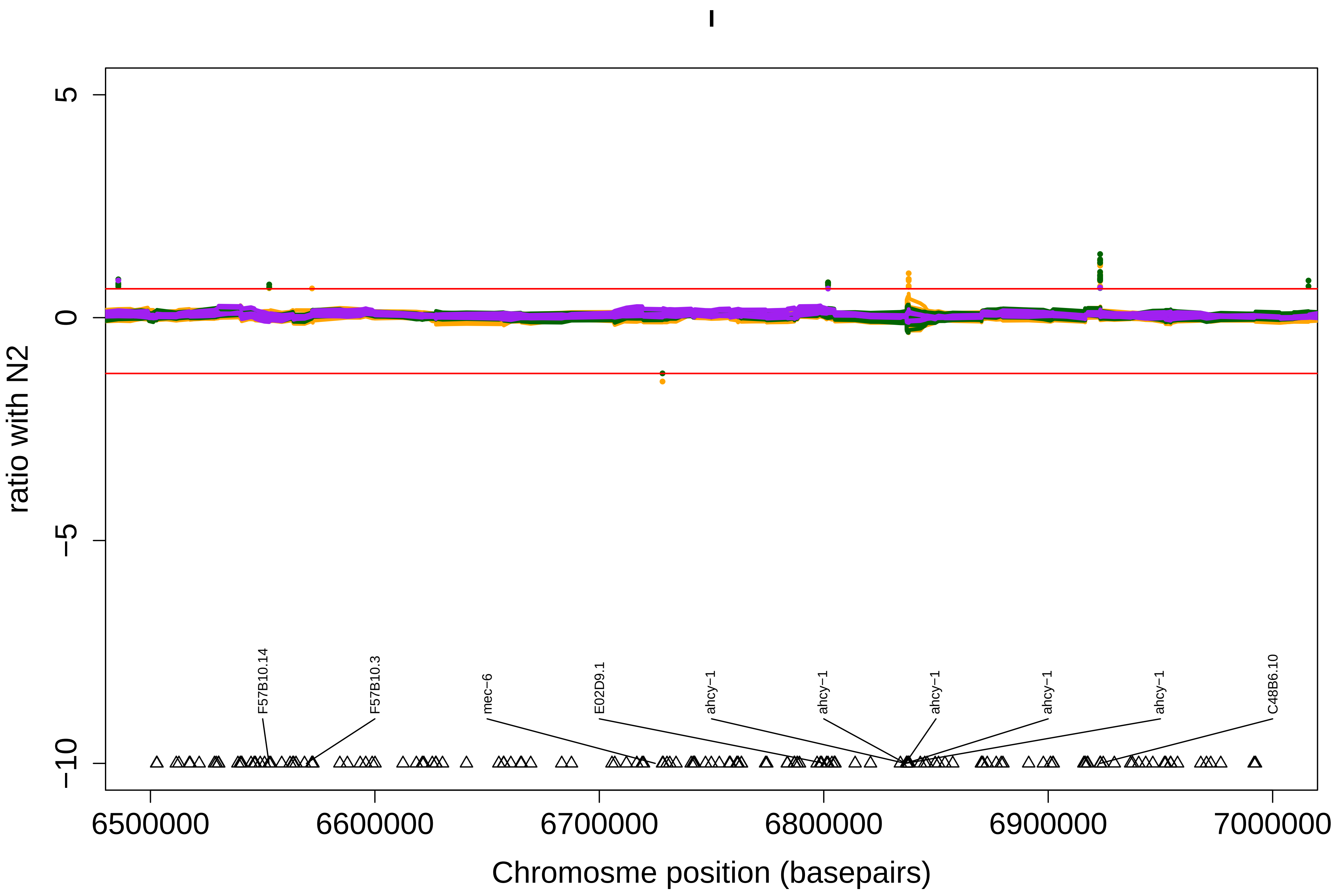
I
ratio with N2
5
0
−5
−10
F57B10.14
F57B10.3
mec−6
E02D9.1
ahcy−1
ahcy−1
ahcy−1
ahcy−1
ahcy−1
C48B6.10
6500000
6600000
6700000
6800000
6900000
7000000
Chromosme position (basepairs)

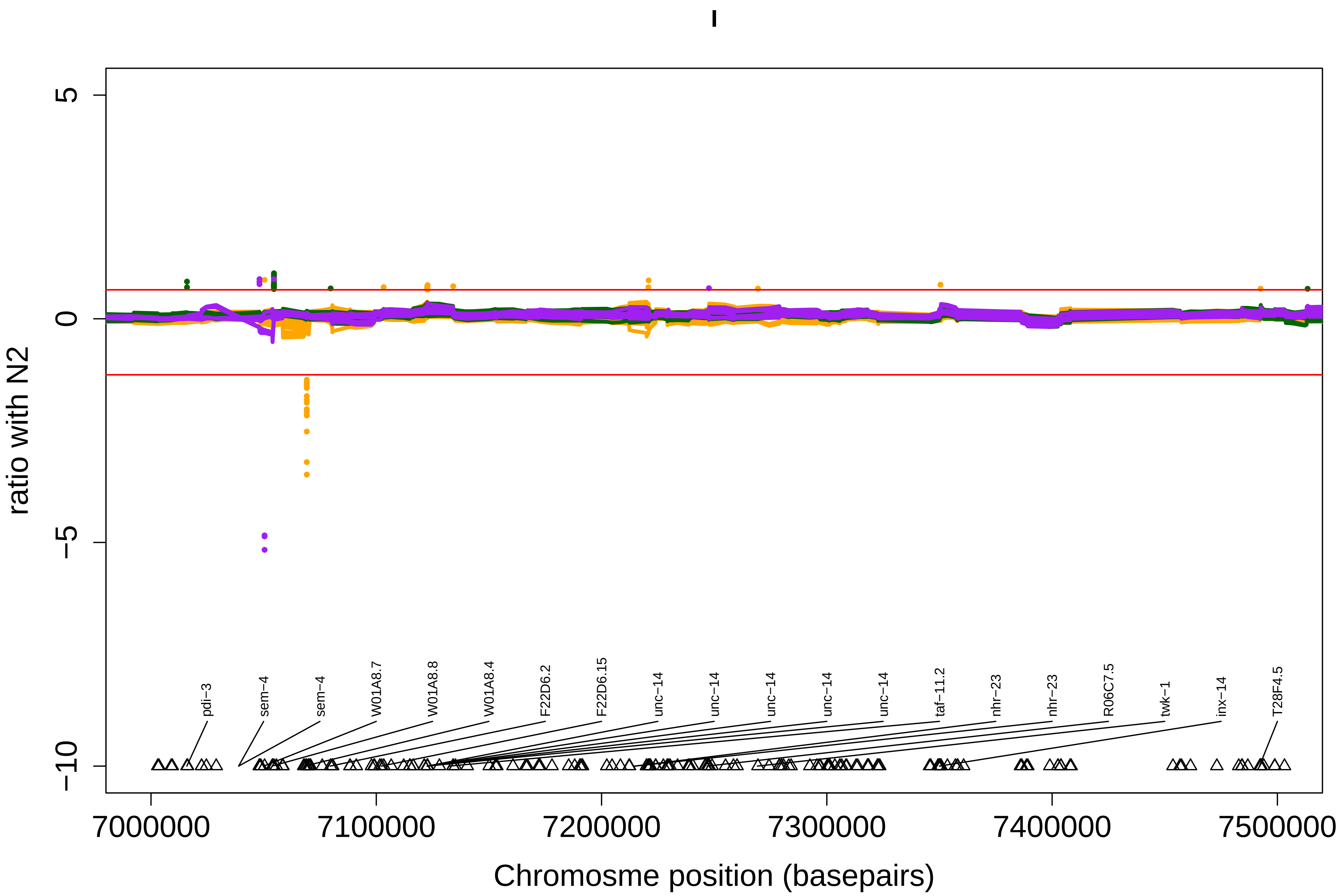
I
ratio with N2
5
0
−5
−10
pdi−3
sem−4
sem−4
W01A8.7
W01A8.8
W01A8.4
F22D6.2
F22D6.15
unc−14
unc−14
unc−14
unc−14
unc−14
taf−11.2
nhr−23
nhr−23
R06C7.5
twk−1
inx−14
T28F4.5
7000000
7100000
7200000
7300000
7400000
7500000
Chromosme position (basepairs)

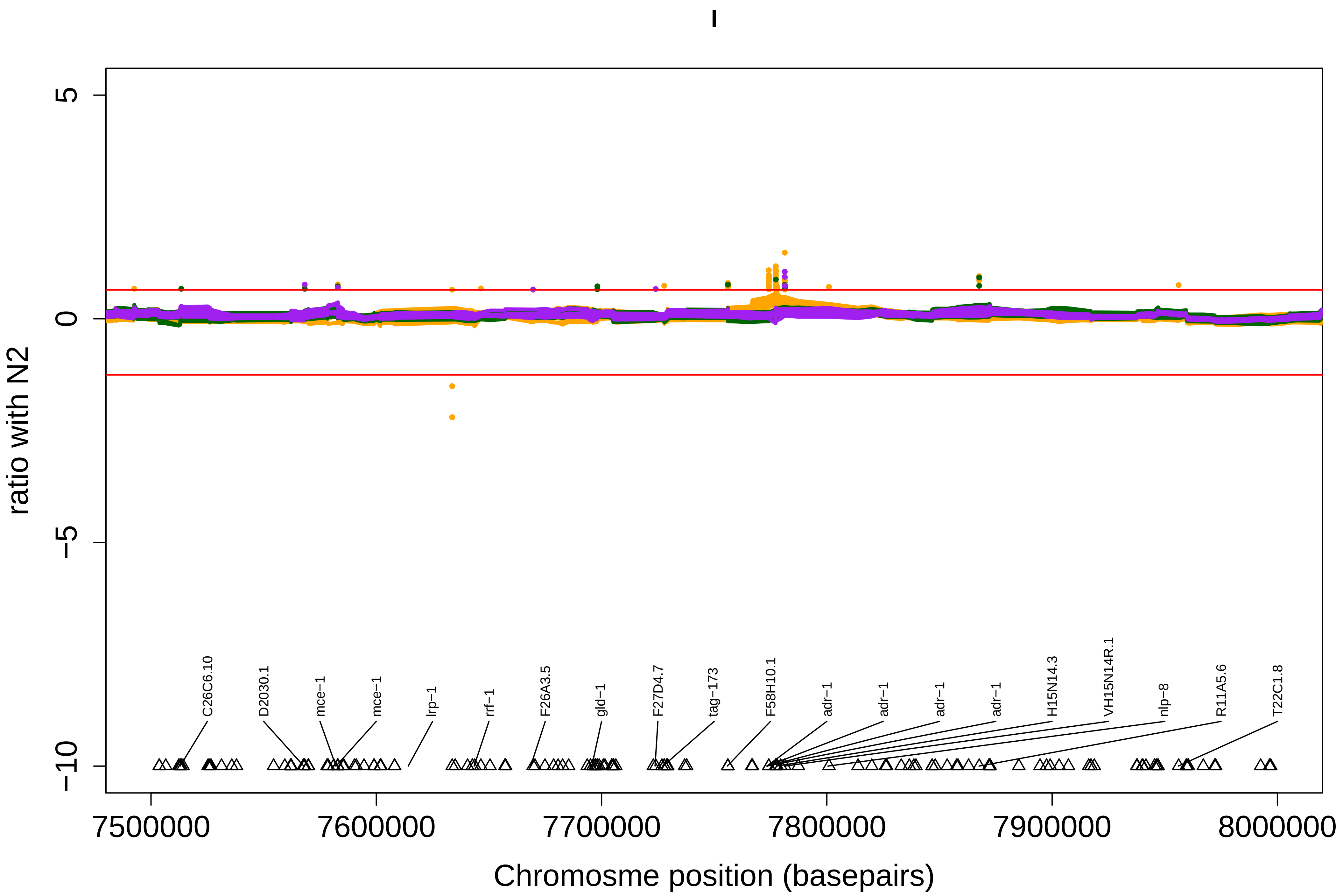
I
ratio with N2
5
0
−5
−10
7500000
7600000
7700000
7800000
7900000
8000000
Chromosme position (basepairs)
C26C6.10
D2030.1
mce−1
mce−1
lrp−1
rrf−1
F26A3.5
gld−1
F27D4.7
tag−173
F58H10.1
adr−1
adr−1
adr−1
adr−1
H15N14.3
VH15N14R.1
nlp−8
R11A5.6
T22C1.8

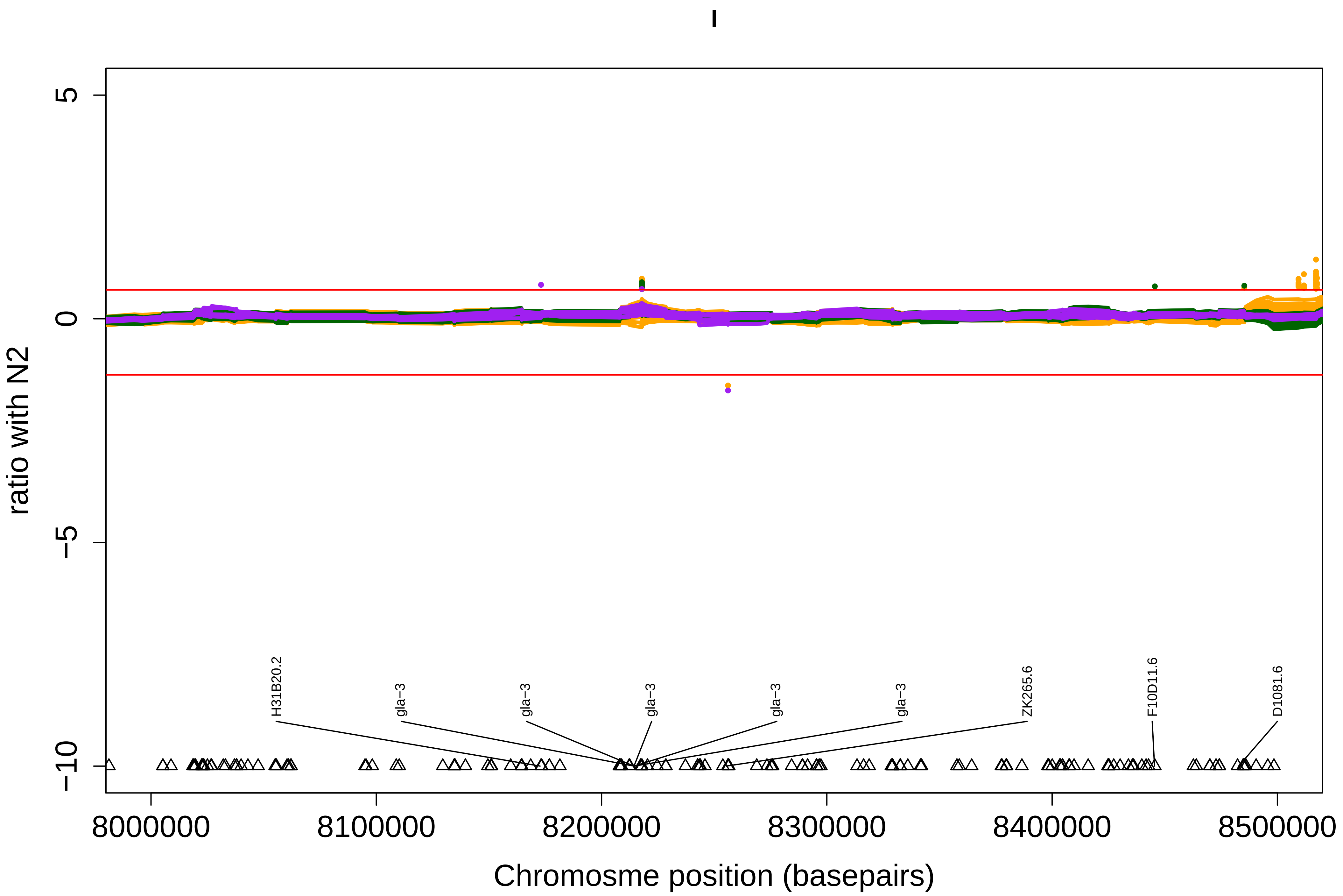
I
ratio with N2
5
0
−5
−10
H31B20.2
gla−3
gla−3
gla−3
gla−3
gla−3
ZK265.6
F10D11.6
D1081.6
8000000
8100000
8200000
8300000
8400000
8500000
Chromosme position (basepairs)

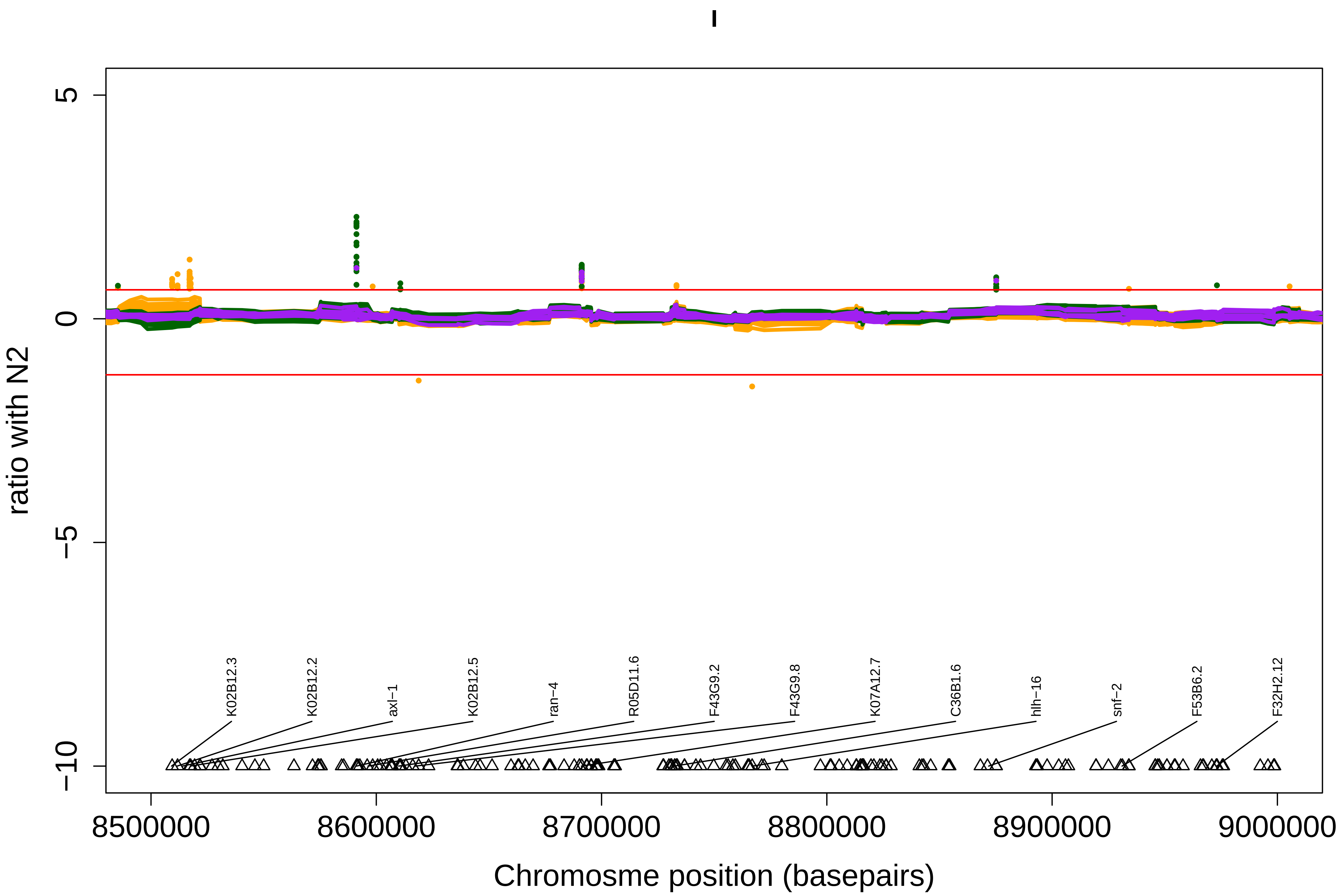

I
ratio with N2
5
0
−5
−10
K02B12.3
K02B12.2
axl−1
K02B12.5
ran−4
R05D11.6
F43G9.2
F43G9.8
K07A12.7
C36B1.6
hlh−16
snf−2
F53B6.2
F32H2.12
8500000
8600000
8700000
8800000
8900000
9000000
Chromosme position (basepairs)

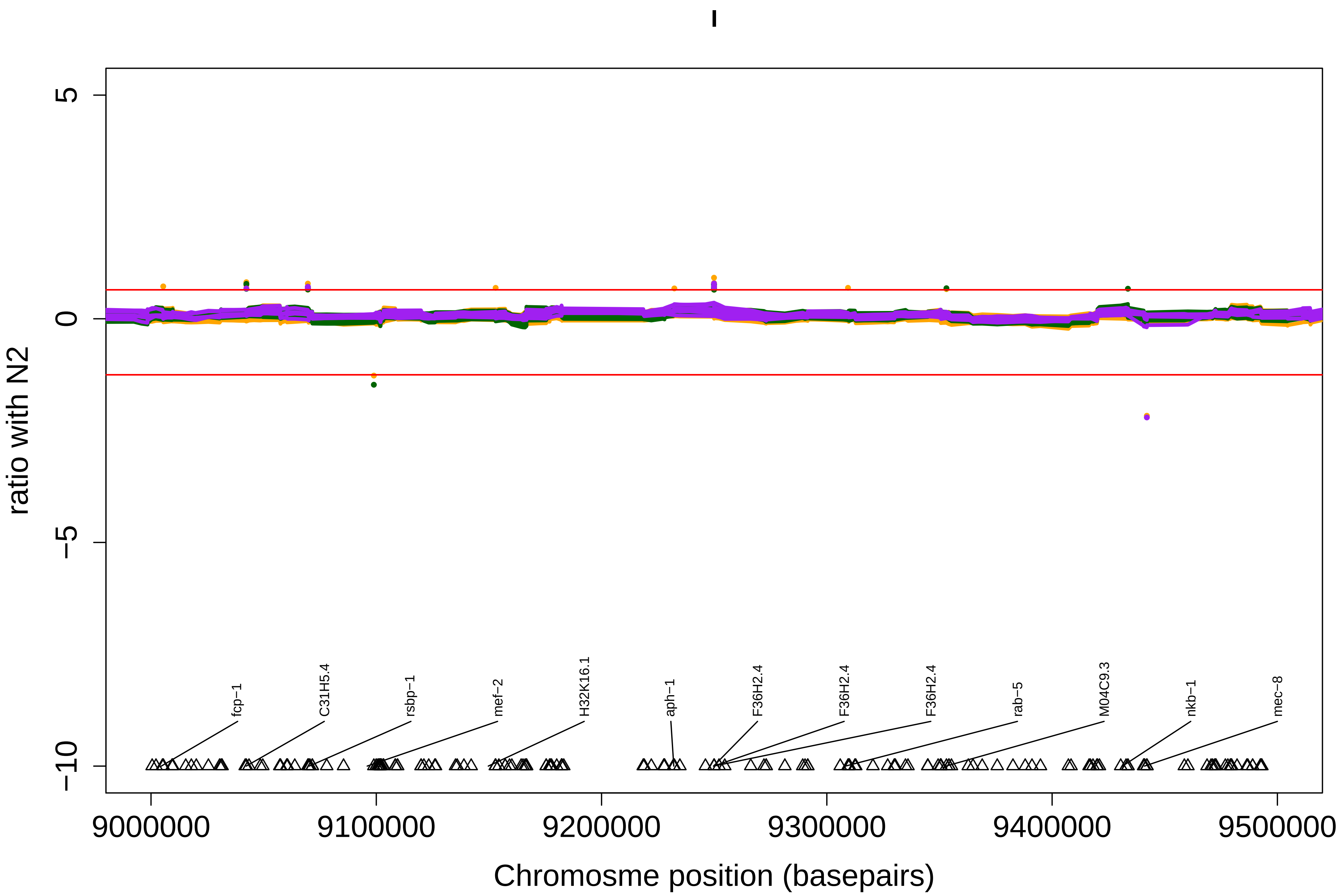

I
ratio with N2
5
0
−5
−10
fcp−1
C31H5.4
rsbp−1
mef−2
H32K16.1
aph−1
F36H2.4
F36H2.4
F36H2.4
rab−5
M04C9.3
nkb−1
mec−8
9000000
9100000
9200000
9300000
9400000
9500000
Chromosme position (basepairs)

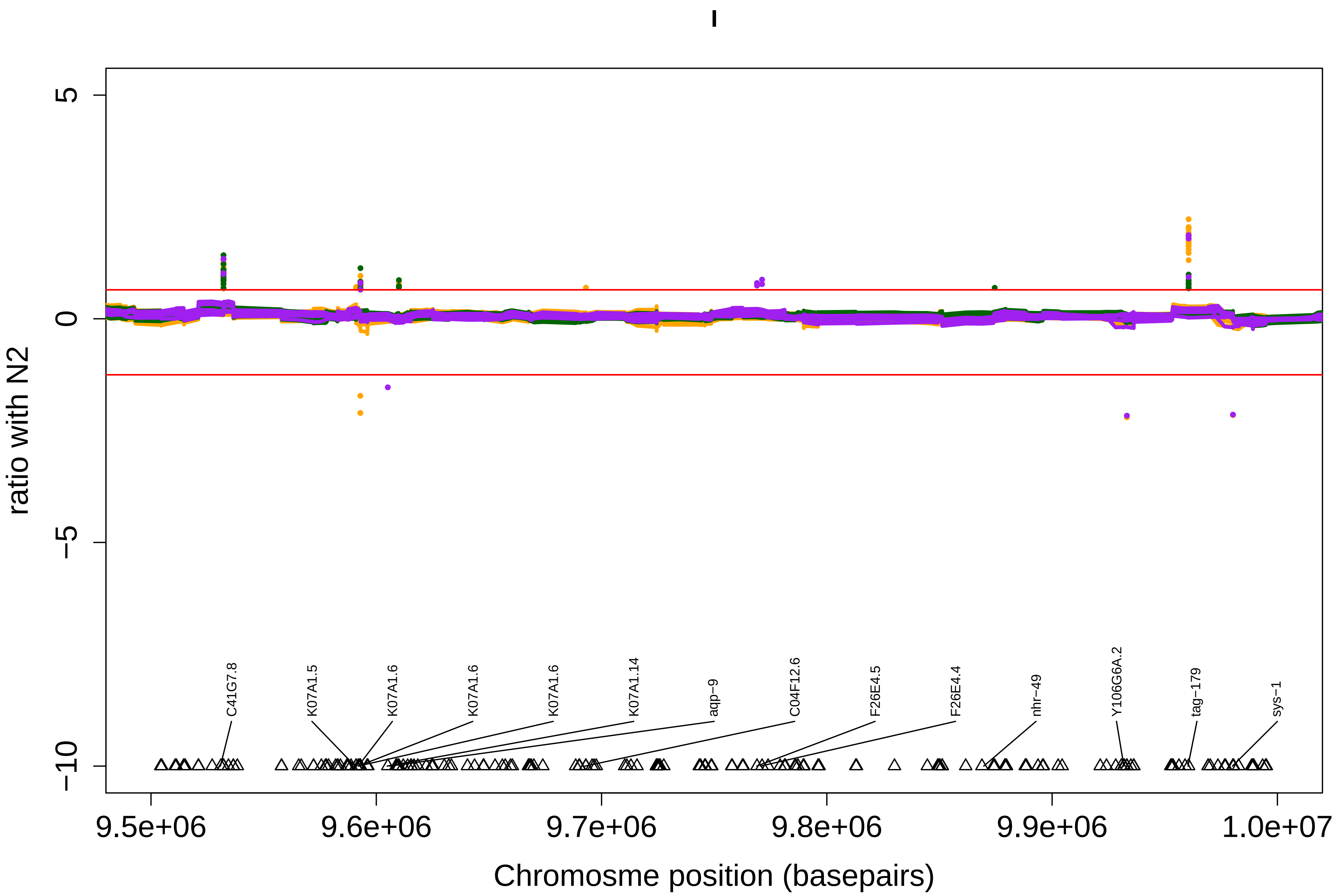
I
ratio with N2
5
0
−5
−10
C41G7.8
K07A1.5
K07A1.6
K07A1.6
K07A1.6
K07A1.14
aqp−9
C04F12.6
F26E4.5
F26E4.4
nhr−49
Y106G6A.2
tag−179
sys−1
9.5e+06
9.6e+06
9.7e+06
9.8e+06
9.9e+06
1.0e+07
Chromosme position (basepairs)

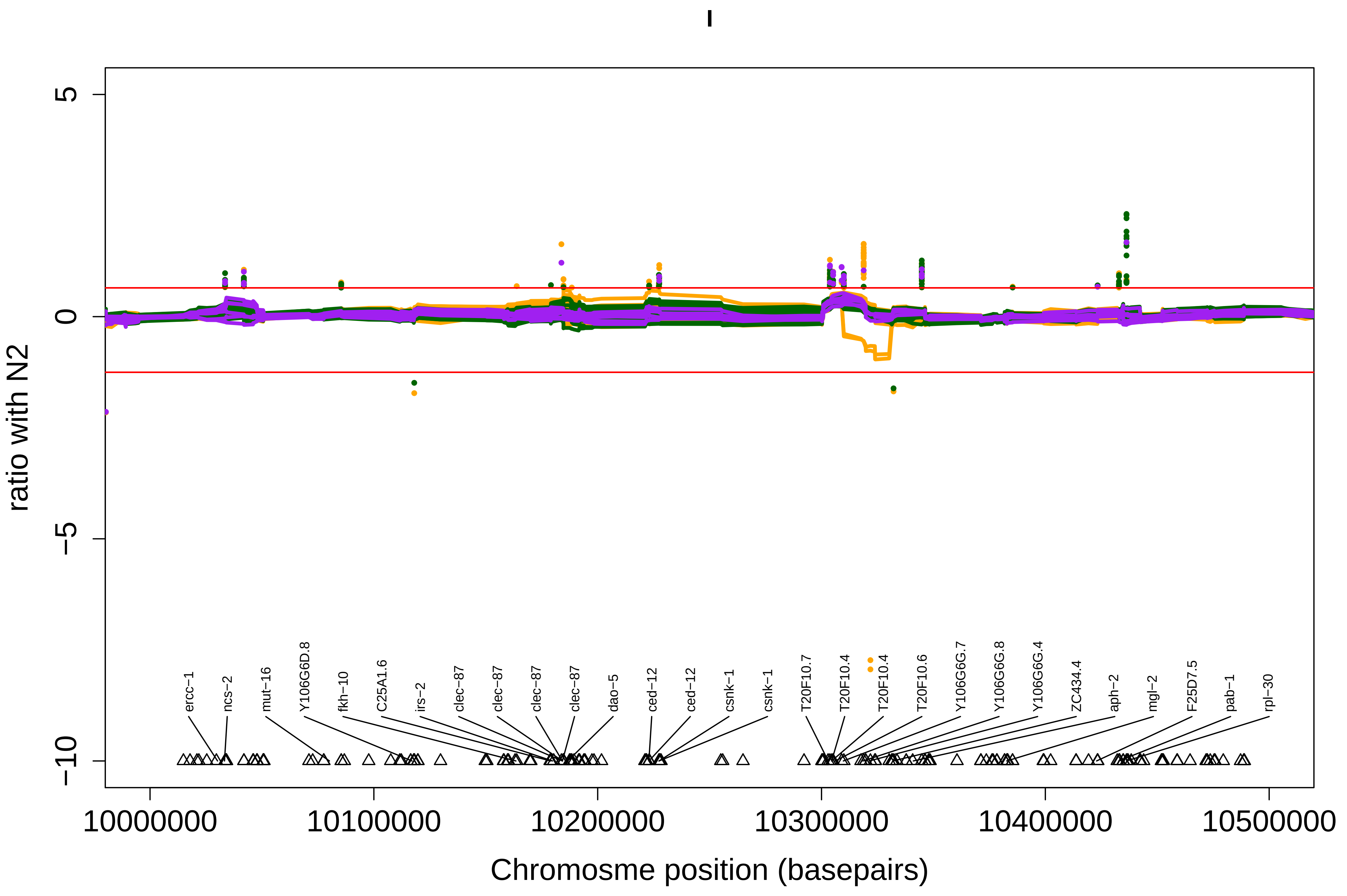

I
ratio with N2
5
0
−5
−10
ercc−1
ncs−2
mut−16
Y106G6D.8
fkh−10
C25A1.6
irs−2
clec−87
clec−87
clec−87
clec−87
dao−5
ced−12
ced−12
csnk−1
csnk−1
T20F10.7
T20F10.4
T20F10.4
T20F10.6
Y106G6G.7
Y106G6G.8
Y106G6G.4
ZC434.4
aph−2
mgl−2
F25D7.5
pab−1
rpl−30
10000000
10100000
10200000
10300000
10400000
10500000
Chromosme position (basepairs)

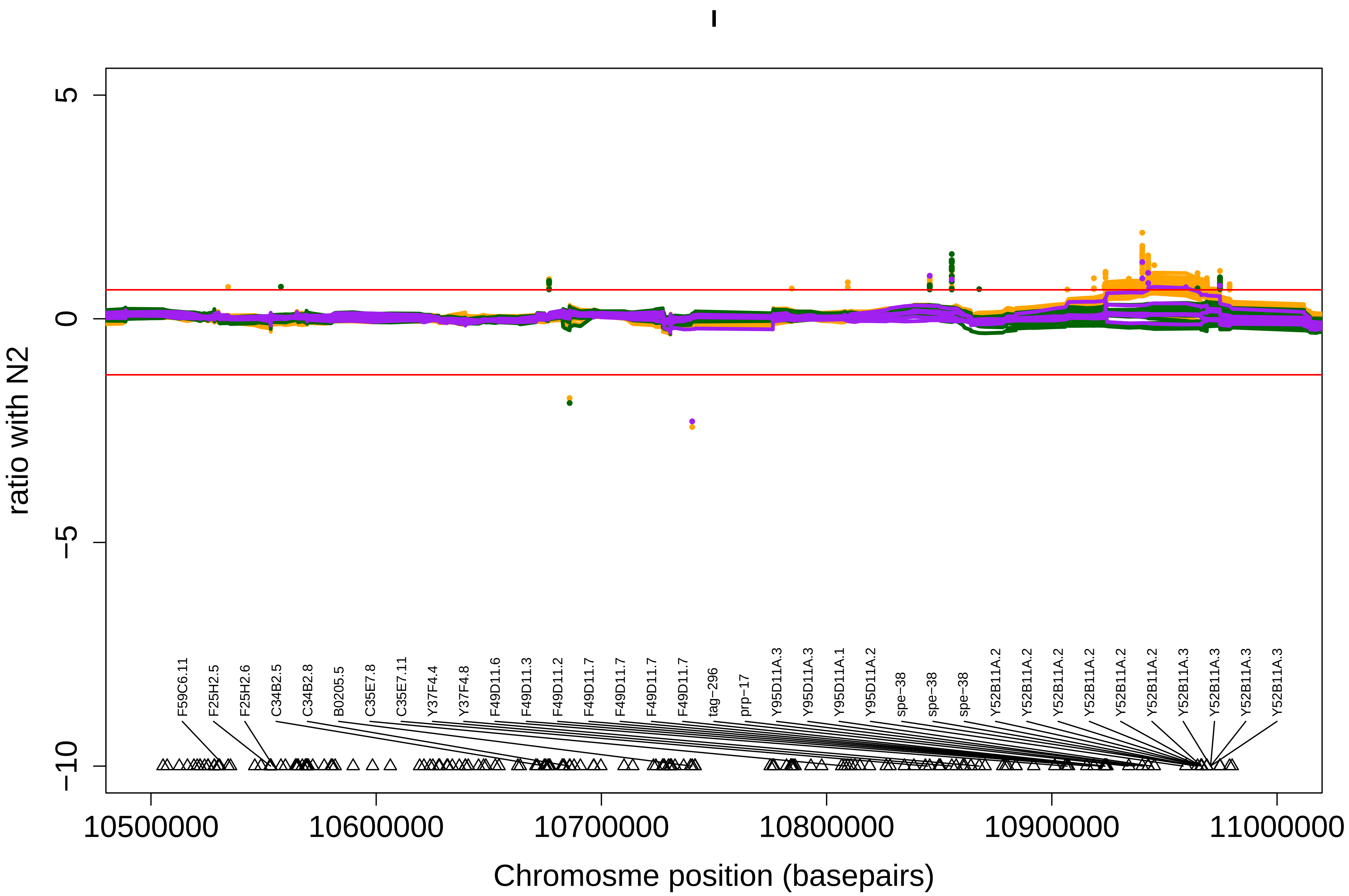
I
ratio with N2
5
0
−5
−10
10500000
10600000
10700000
10800000
10900000
11000000
Chromosme position (basepairs)
F59C6.11
F25H2.5
F25H2.6
C34B2.5
C34B2.8
B0205.5
C35E7.8
C35E7.11
Y37F4.4
Y37F4.8
F49D11.6
F49D11.3
F49D11.2
F49D11.7
F49D11.7
F49D11.7
F49D11.7
tag−296
prp−17
Y95D11A.3
Y95D11A.3
Y95D11A.1
Y95D11A.2
spe−38
spe−38
spe−38
Y52B11A.2
Y52B11A.2
Y52B11A.2
Y52B11A.2
Y52B11A.2
Y52B11A.2
Y52B11A.3
Y52B11A.3
Y52B11A.3
Y52B11A.3

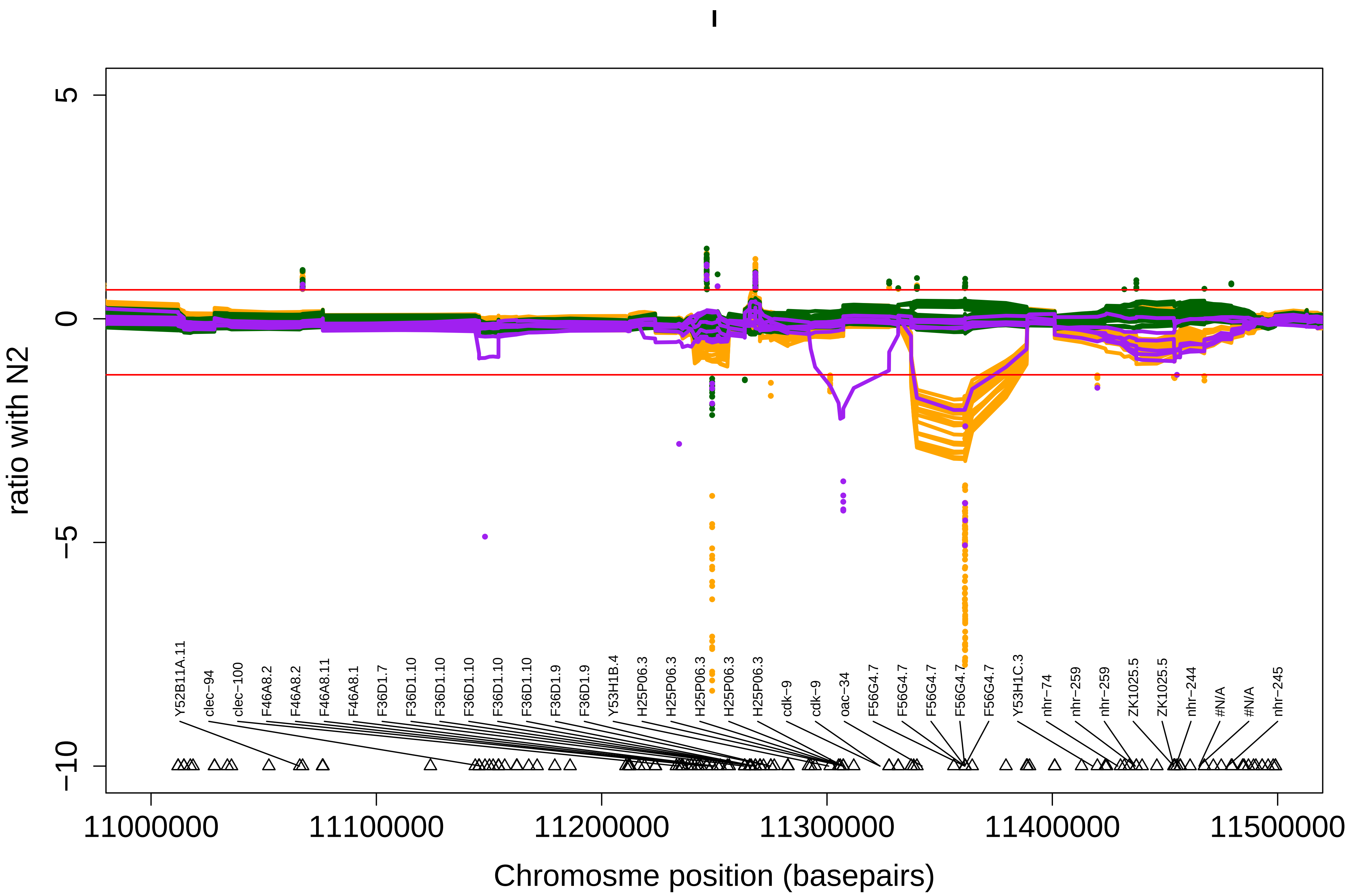

I
ratio with N2
5
0
−5
−10
Y52B11A.11
clec−94
clec−100
F46A8.2
F46A8.2
F46A8.11
F46A8.1
F36D1.7
F36D1.10
F36D1.10
F36D1.10
F36D1.10
F36D1.10
F36D1.9
F36D1.9
Y53H1B.4
H25P06.3
H25P06.3
H25P06.3
H25P06.3
H25P06.3
cdk−9
cdk−9
oac−34
F56G4.7
F56G4.7
F56G4.7
F56G4.7
F56G4.7
Y53H1C.3
nhr−74
nhr−259
nhr−259
ZK1025.5
ZK1025.5
nhr−244
#N/A
#N/A
nhr−245
11000000
11100000
11200000
11300000
11400000
11500000
Chromosme position (basepairs)

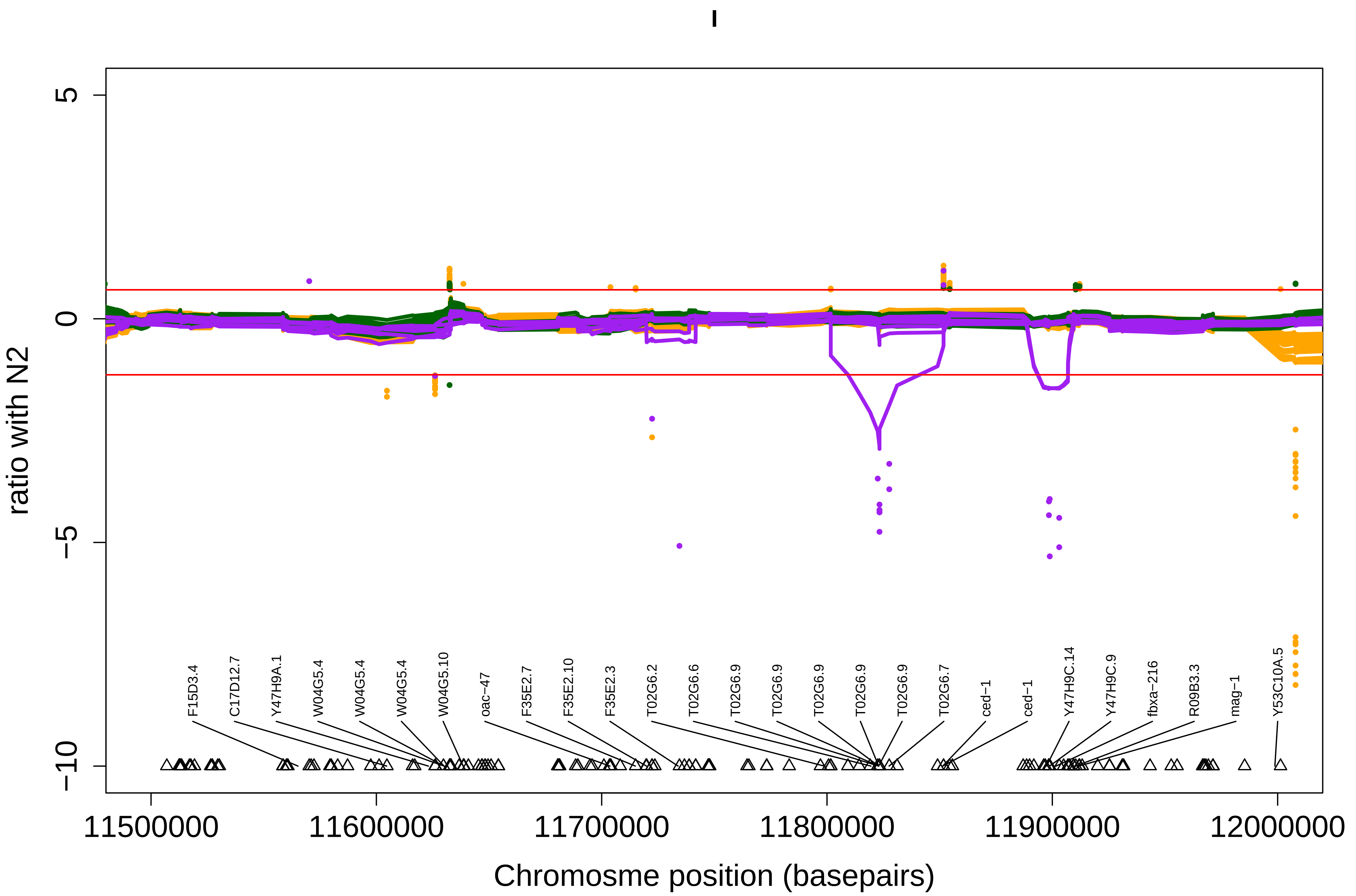
I
ratio with N2
5
0
−5
−10
11500000
11600000
11700000
11800000
11900000
12000000
Chromosme position (basepairs)
F15D3.4
C17D12.7
Y47H9A.1
W04G5.4
W04G5.4
W04G5.4
W04G5.10
oac−47
F35E2.7
F35E2.10
F35E2.3
T02G6.2
T02G6.6
T02G6.9
T02G6.9
T02G6.9
T02G6.9
T02G6.9
T02G6.7
ced−1
ced−1
Y47H9C.14
Y47H9C.9
fbxa−216
R09B3.3
mag−1
Y53C10A.5

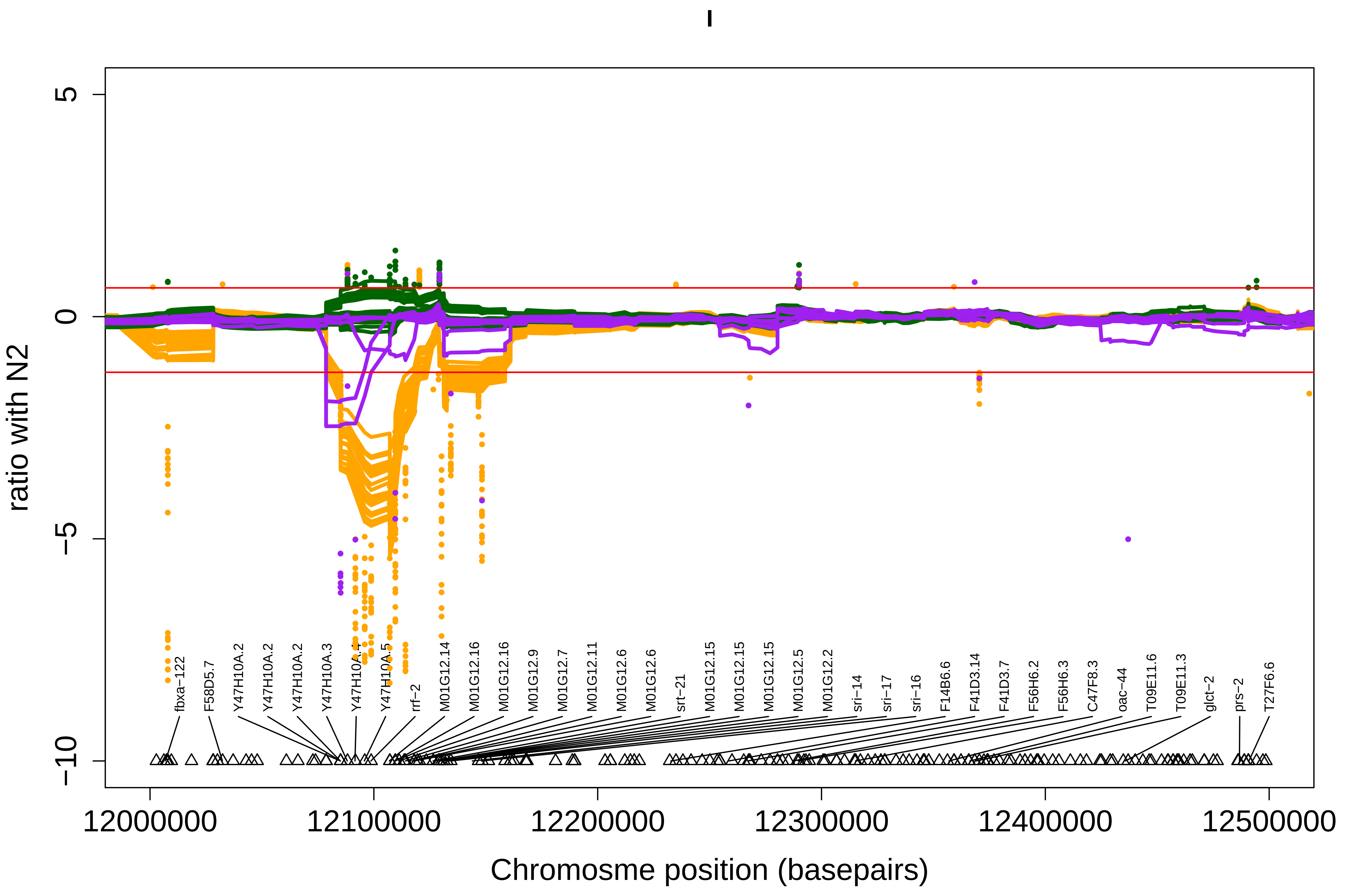

I
ratio with N2
5
0
−5
−10
12000000
12100000
12200000
12300000
12400000
12500000
Chromosme position (basepairs)
fbxa−122
F58D5.7
Y47H10A.2
Y47H10A.2
Y47H10A.2
Y47H10A.3
Y47H10A.5
rrf−2
M01G12.14
M01G12.16
M01G12.16
M01G12.9
M01G12.7
M01G12.11
M01G12.6
M01G12.6
srt−21
M01G12.15
M01G12.15
M01G12.15
M01G12.5
M01G12.2
sri−14
sri−17
sri−16
F14B6.6
F41D3.14
F41D3.7
F56H6.2
F56H6.3
C47F8.3
oac−44
T09E11.6
T09E11.3
glct−2
prs−2
T27F6.6

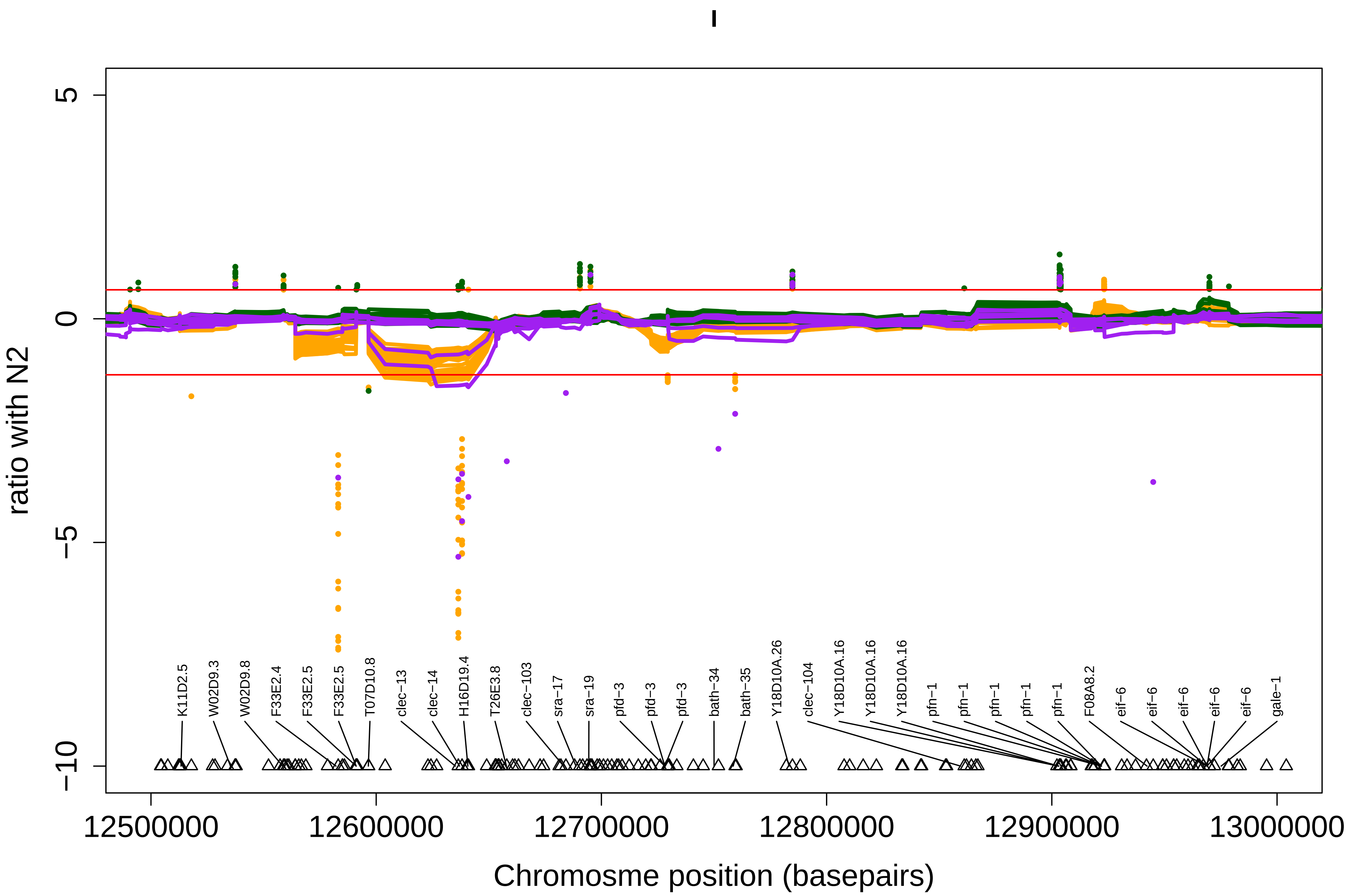
I
ratio with N2
Chromosme position (basepairs)
5
0
−5
−10
12500000
12600000
12700000
12800000
12900000
13000000
K11D2.5
W02D9.3
W02D9.8
F33E2.4
F33E2.5
F33E2.5
T07D10.8
clec−13
clec−14
H16D19.4
T26E3.8
clec−103
sra−17
sra−19
pfd−3
pfd−3
pfd−3
bath−34
bath−35
Y18D10A.26
clec−104
Y18D10A.16
Y18D10A.16
Y18D10A.16
pfn−1
pfn−1
pfn−1
pfn−1
pfn−1
F08A8.2
eif−6
eif−6
eif−6
eif−6
eif−6
gale−1

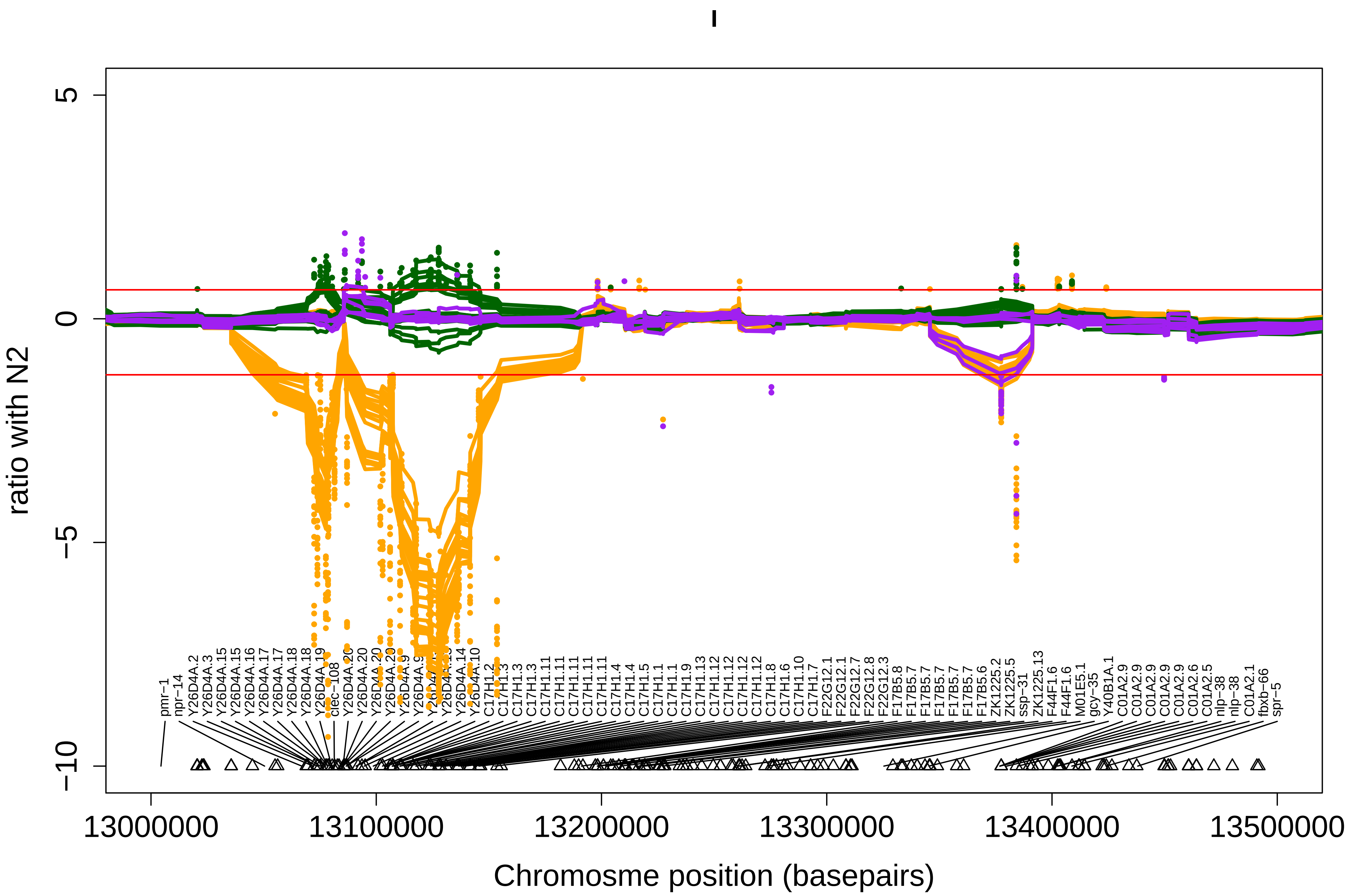
I
ratio with N2
5
0
−5
−10
Chromosme position (basepairs)
13000000
13100000
13200000
13300000
13400000
13500000
pmr−1
npr−14
Y26D4A.2
Y26D4A.3
Y26D4A.15
Y26D4A.15
Y26D4A.16
Y26D4A.17
Y26D4A.17
Y26D4A.18
Y26D4A.18
Y26D4A.19
clec−108
Y26D4A.20
Y26D4A.20
Y26D4A.20
Y26D4A.20
Y26D4A.9
Y26D4A.9
Y26D4A.14
Y26D4A.10
C17H1.2
C17H1.3
C17H1.3
C17H1.3
C17H1.11
C17H1.11
C17H1.11
C17H1.11
C17H1.4
C17H1.4
C17H1.5
C17H1.1
C17H1.1
C17H1.9
C17H1.13
C17H1.12
C17H1.12
C17H1.12
C17H1.12
C17H1.8
C17H1.6
C17H1.10
C17H1.7
F22G12.1
F22G12.1
F22G12.7
F22G12.8
F22G12.3
F17B5.8
F17B5.7
F17B5.7
F17B5.7
F17B5.7
F17B5.6
ZK1225.2
ZK1225.5
ssp−31
ZK1225.13
F44F1.6
F44F1.6
M01E5.1
gcy−35
Y40B1A.1
C01A2.9
C01A2.9
C01A2.9
C01A2.9
C01A2.9
C01A2.6
C01A2.5
nlp−38
nlp−38
C01A2.1
fbxb−66
spr−5

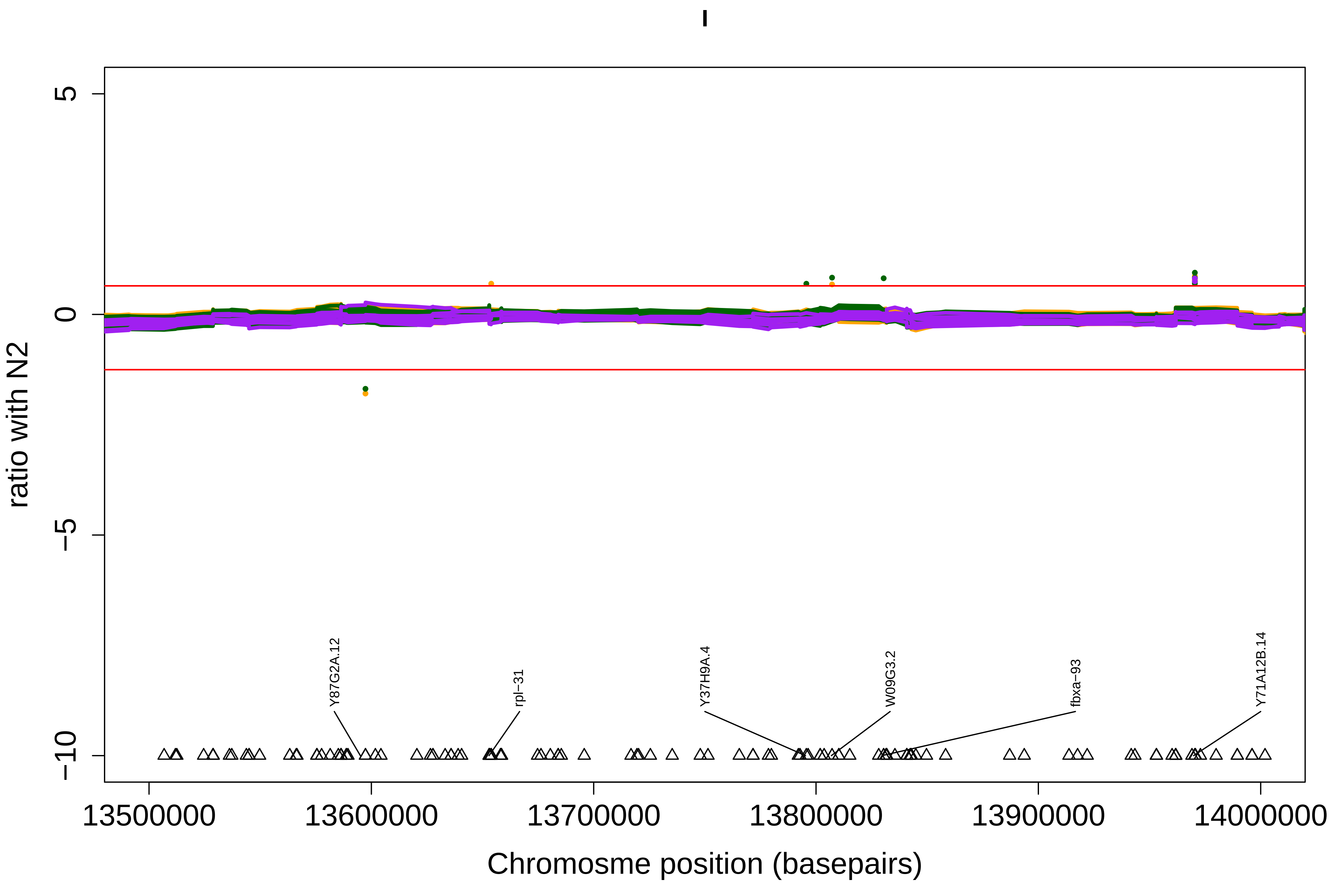
I
ratio with N2
5
0
−5
−10
13500000
13600000
13700000
13800000
13900000
14000000
Chromosme position (basepairs)
Y87G2A.12
rpl−31
Y37H9A.4
W09G3.2
fbxa−93
Y71A12B.14

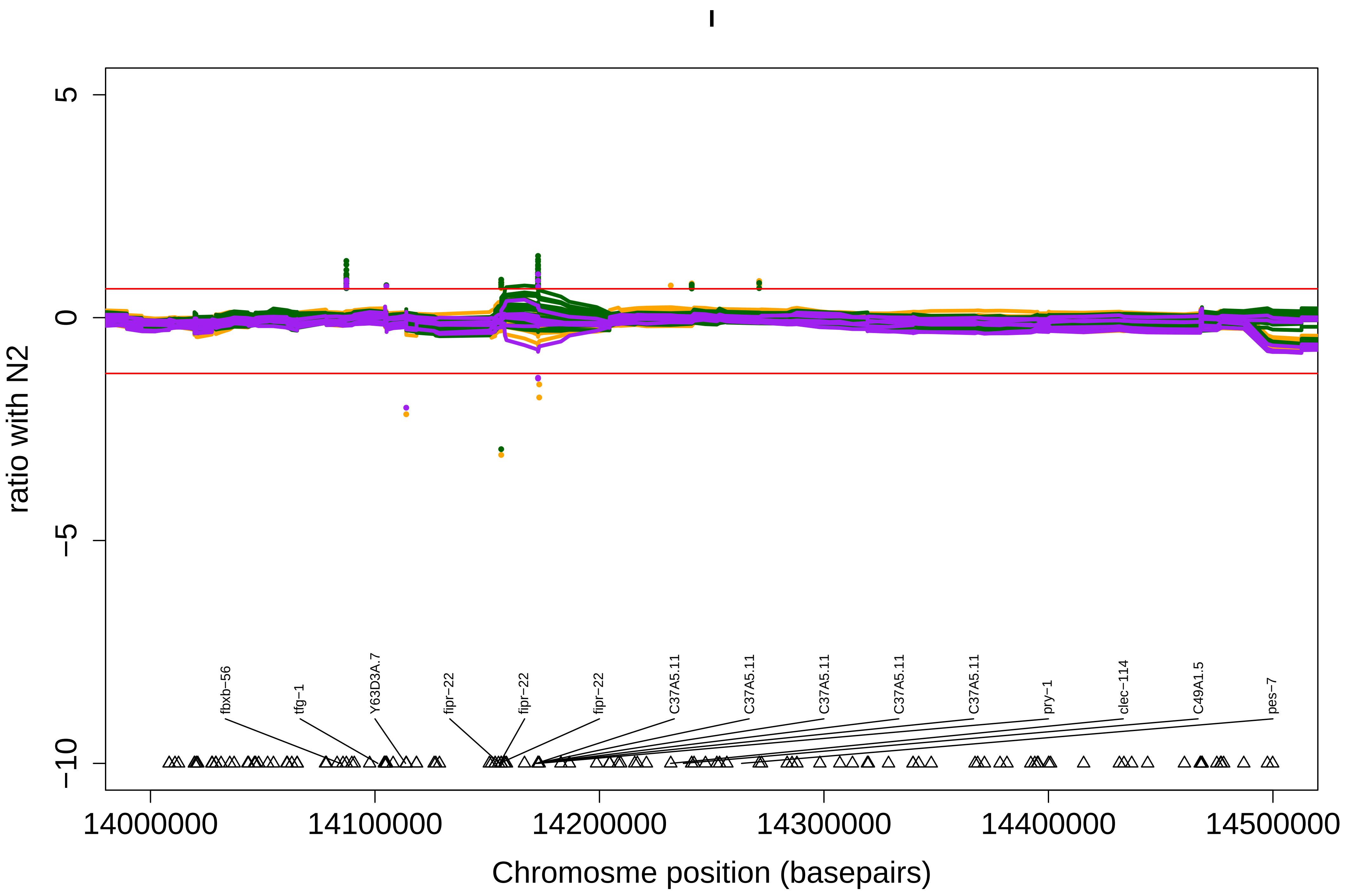
I
ratio with N2
5
0
−5
−10
Chromosme position (basepairs)
14000000
14100000
14200000
14300000
14400000
14500000
fbxb−56
tfg−1
Y63D3A.7
fipr−22
fipr−22
fipr−22
C37A5.11
C37A5.11
C37A5.11
C37A5.11
C37A5.11
pry−1
clec−114
C49A1.5
pes−7

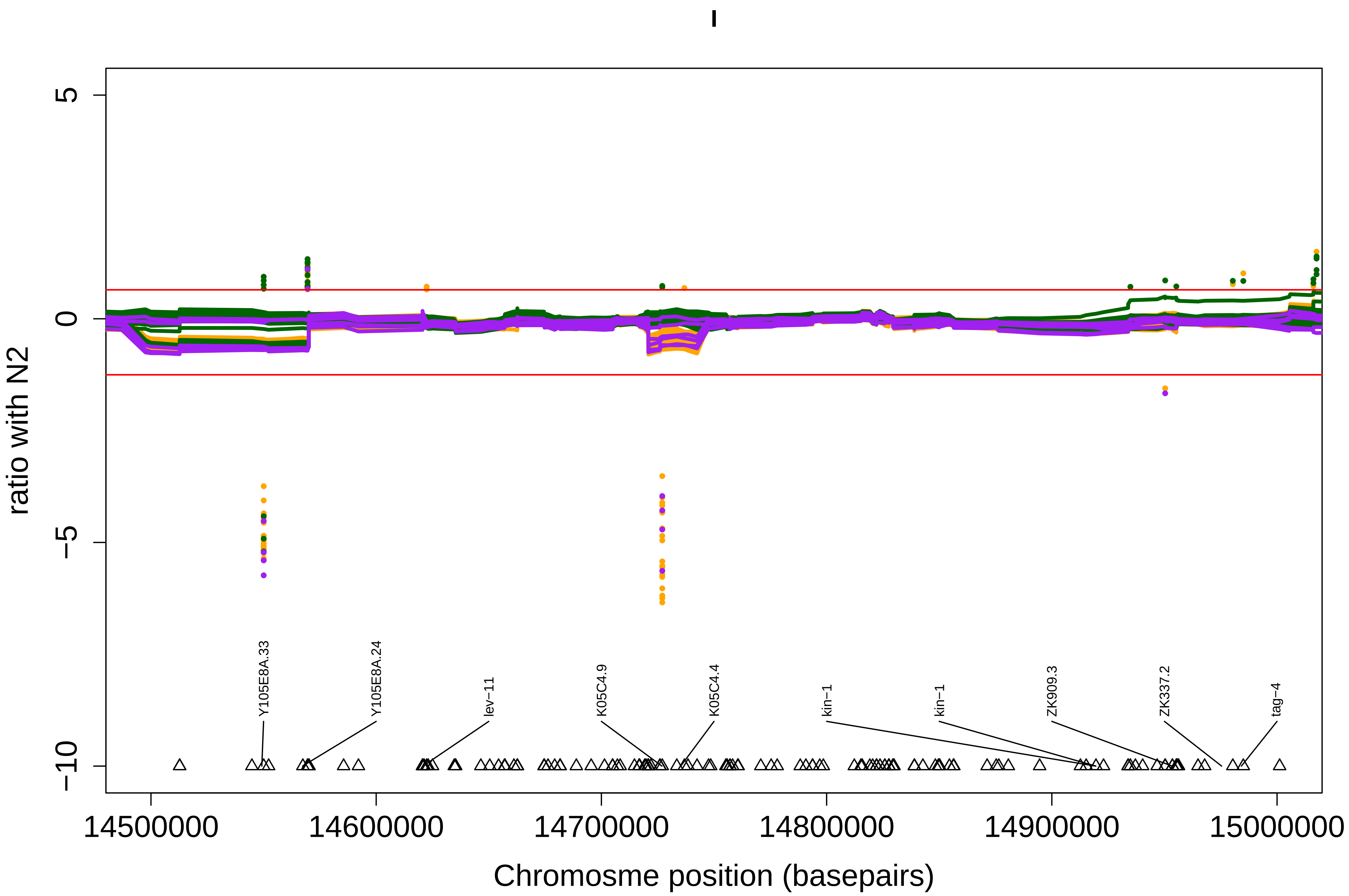
I
ratio with N2
5
0
−5
−10
Y105E8A.33
Y105E8A.24
lev−11
K05C4.9
K05C4.4
kin−1
kin−1
ZK909.3
ZK337.2
tag−4
14500000
14600000
14700000
14800000
14900000
15000000
Chromosme position (basepairs)

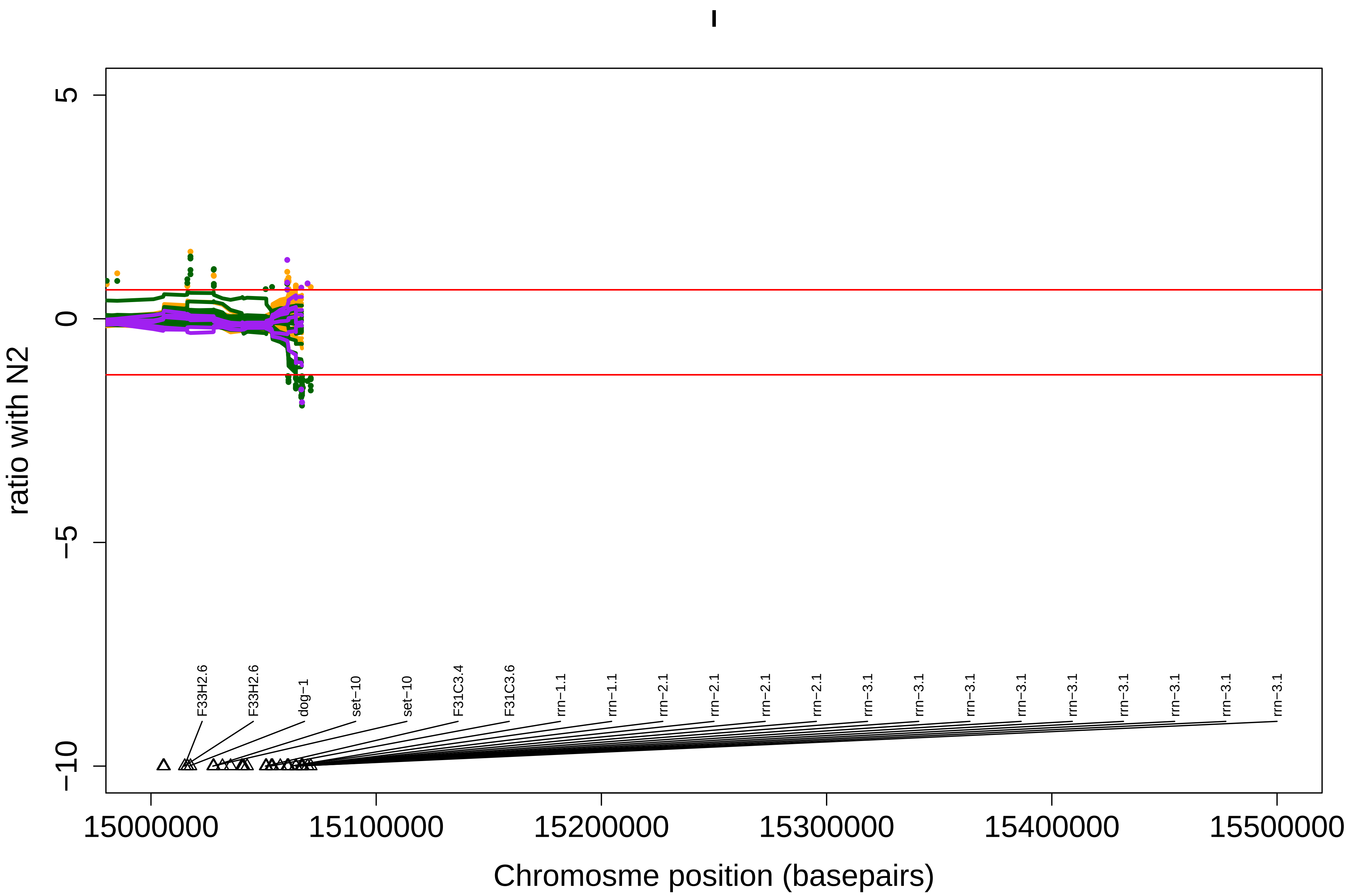
I
ratio with N2
5
0
−5
−10
F33H2.6
F33H2.6
dog−1
set−10
set−10
F31C3.4
F31C3.6
rrn−1.1
rrn−1.1
rrn−2.1
rrn−2.1
rrn−2.1
rrn−2.1
rrn−3.1
rrn−3.1
rrn−3.1
rrn−3.1
rrn−3.1
rrn−3.1
rrn−3.1
rrn−3.1
rrn−3.1
15000000
15100000
15200000
15300000
15400000
15500000
Chromosme position (basepairs)

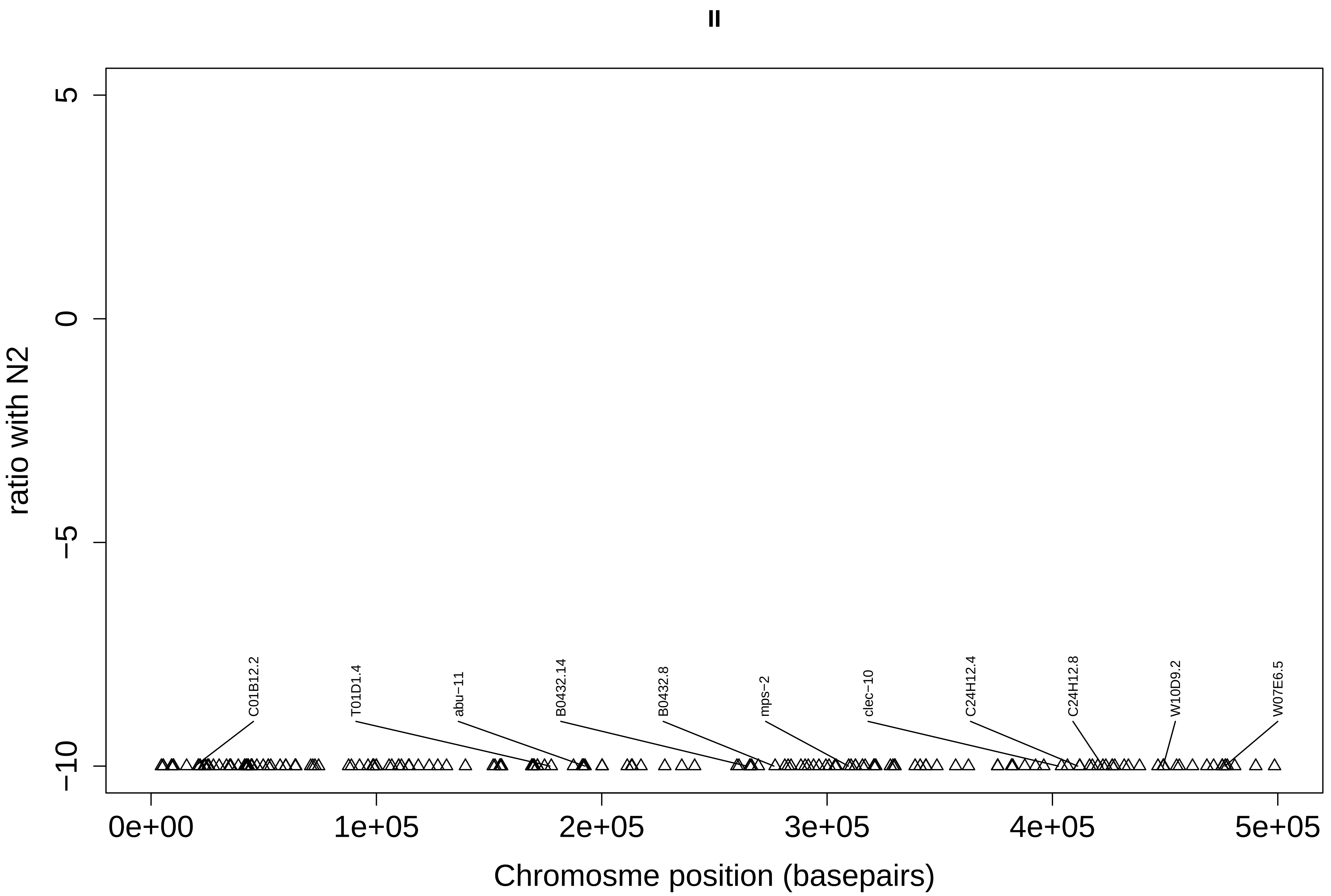

II
5
0
−5
−10
ratio with N2
C01B12.2
T01D1.4
abu−11
B0432.14
B0432.8
mps−2
clec−10
C24H12.4
C24H12.8
W10D9.2
W07E6.5
0e+00
1e+05
2e+05
3e+05
4e+05
5e+05
Chromosme position (basepairs)

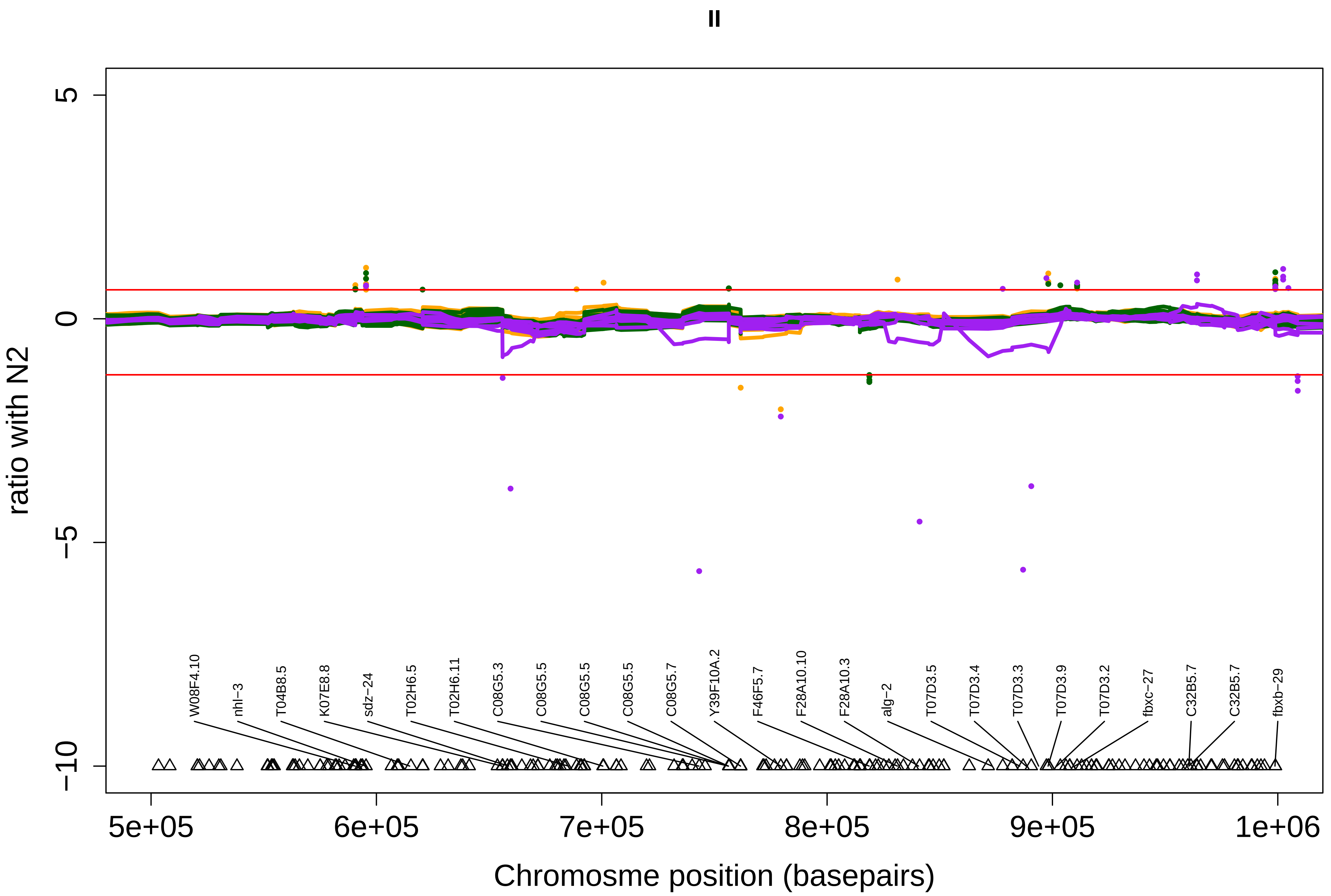

II
ratio with N2
5
0
−5
−10
W08F4.10
nhl−3
T04B8.5
K07E8.8
sdz−24
T02H6.5
T02H6.11
C08G5.3
C08G5.5
C08G5.5
C08G5.5
C08G5.7
Y39F10A.2
F46F5.7
F28A10.10
F28A10.3
alg−2
T07D3.5
T07D3.4
T07D3.3
T07D3.9
T07D3.2
fbxc−27
C32B5.7
C32B5.7
fbxb−29
5e+05
6e+05
7e+05
8e+05
9e+05
1e+06
Chromosme position (basepairs)

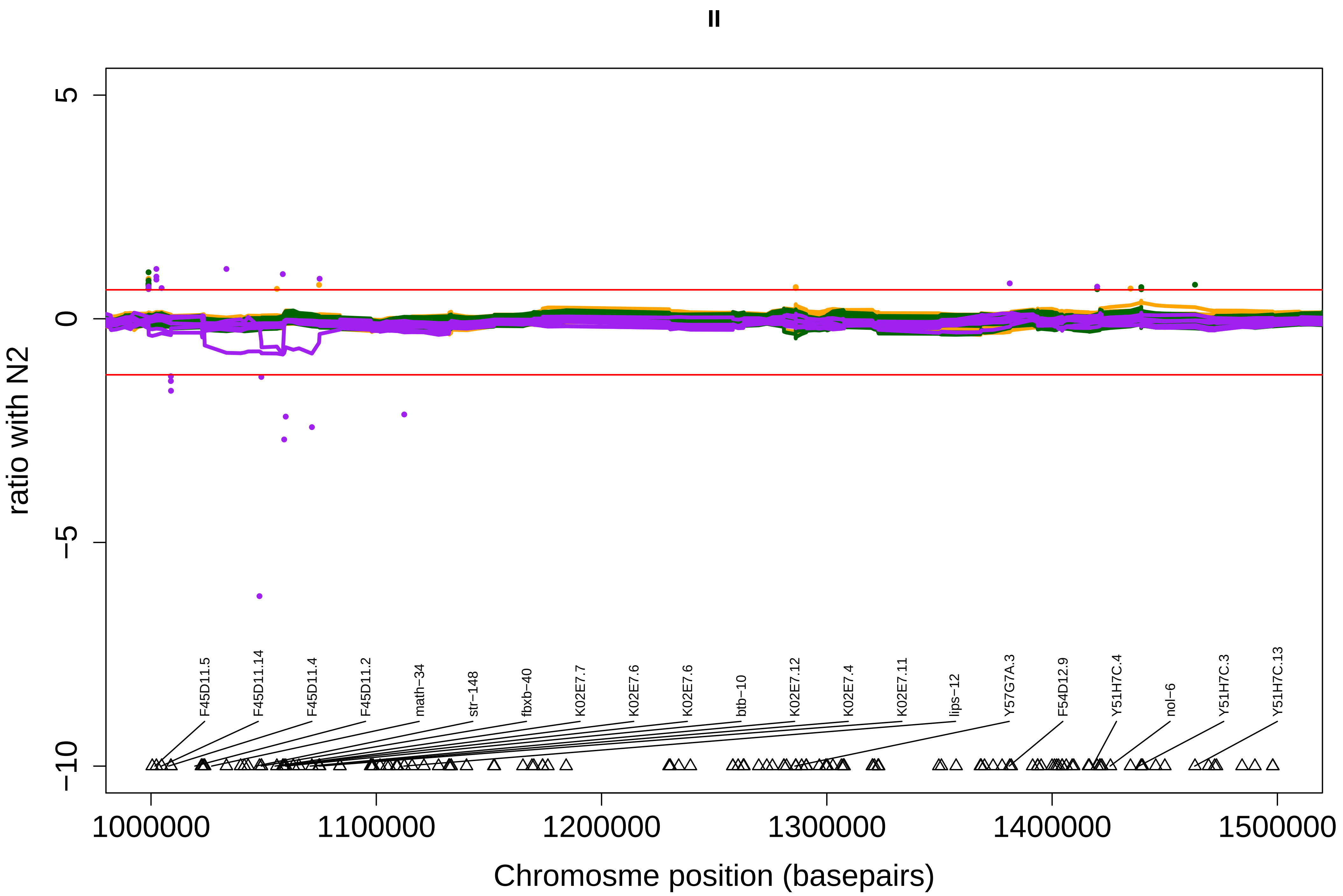

II
ratio with N2
5
0
−5
−10
F45D11.5
F45D11.14
F45D11.4
F45D11.2
math−34
str−148
fbxb−40
K02E7.7
K02E7.6
K02E7.6
btb−10
K02E7.12
K02E7.4
K02E7.11
lips−12
Y57G7A.3
F54D12.9
Y51H7C.4
nol−6
Y51H7C.3
Y51H7C.13
1000000
1100000
1200000
1300000
1400000
1500000
Chromosme position (basepairs)

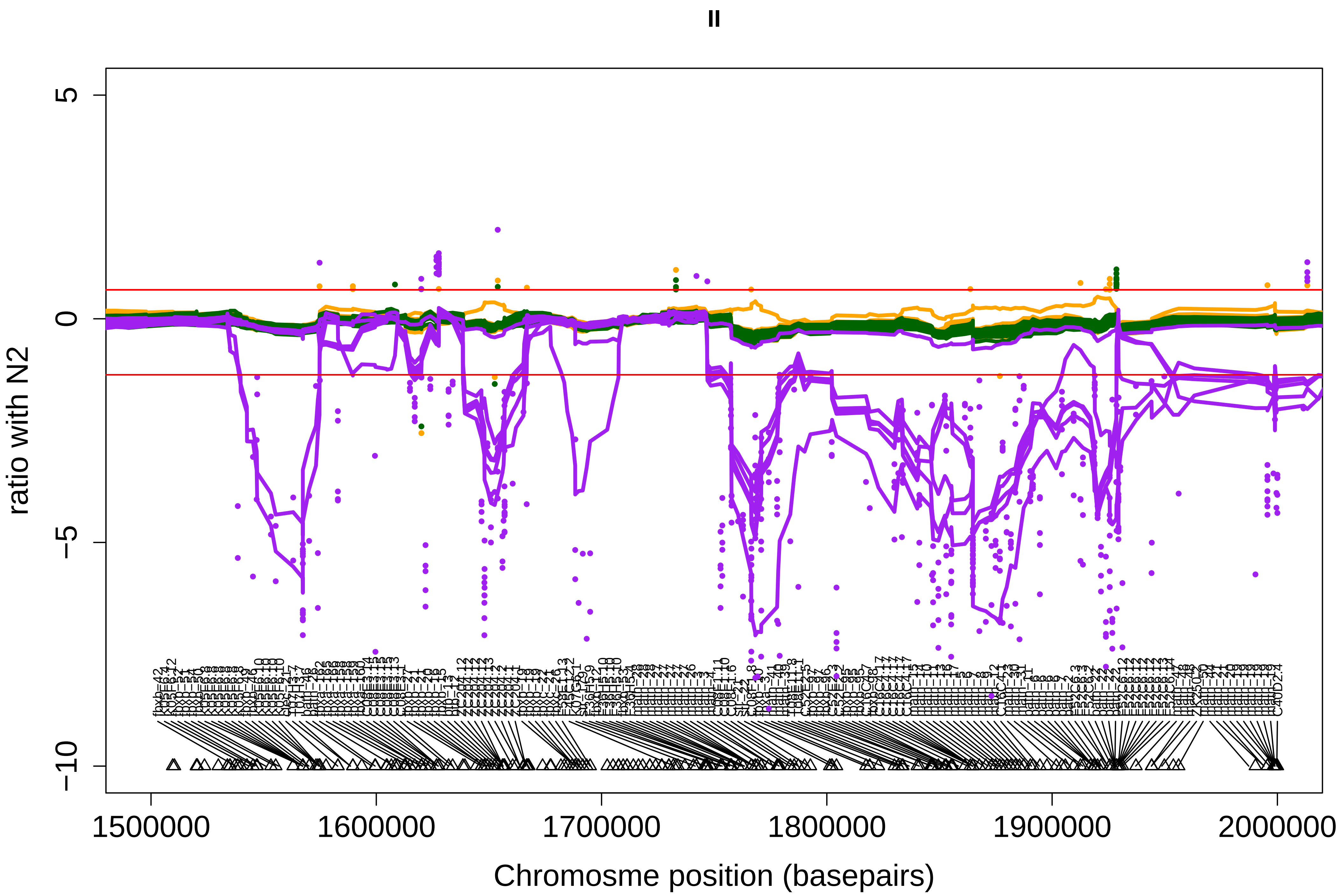
II
ratio with N2
Chromosme position (basepairs)
5
0
−5
−10
1500000
1600000
1700000
1800000
1900000
2000000

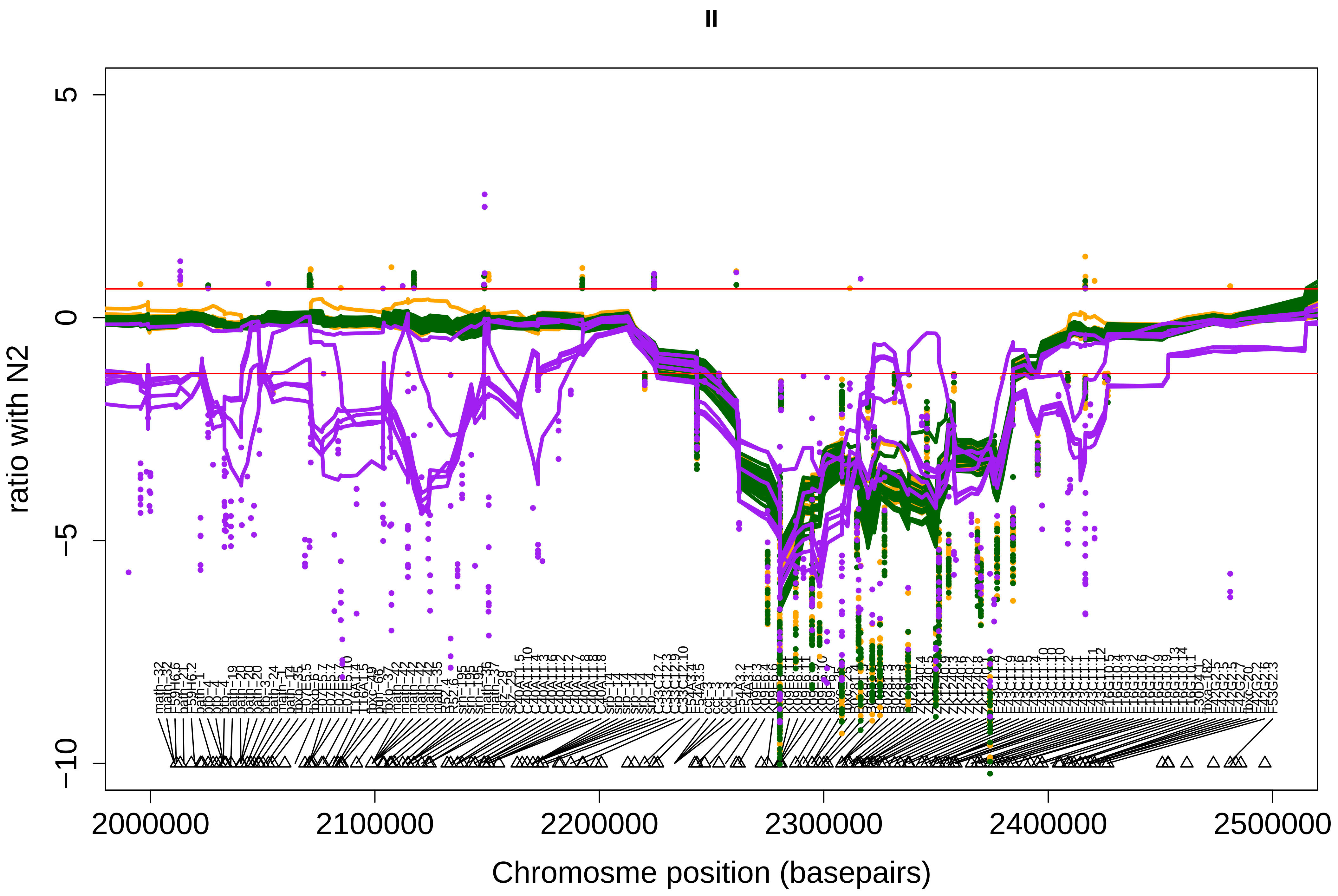
II
ratio with N2
5
0
−5
−10
2000000
2100000
2200000
2300000
2400000
2500000
Chromosme position (basepairs)

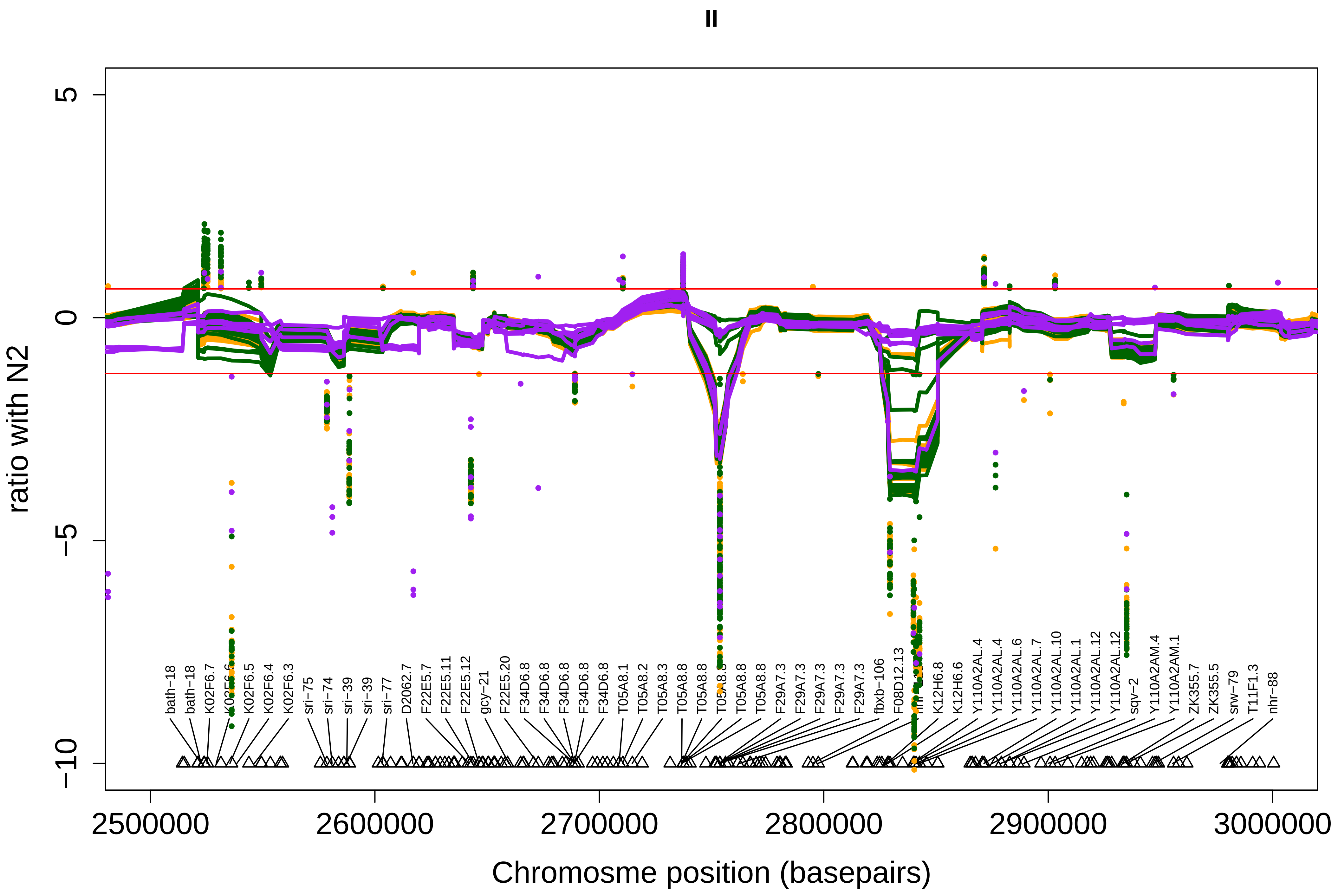
II
ratio with N2
5
0
−5
−10
Chromosme position (basepairs)
2500000
2600000
2700000
2800000
2900000
3000000
bath−18
bath−18
K02F6.7
K02F6.5
K02F6.4
K02F6.3
sri−75
sri−74
sri−39
sri−39
sri−77
D2062.7
F22E5.7
F22E5.11
F22E5.12
gcy−21
F22E5.20
F34D6.8
F34D6.8
F34D6.8
F34D6.8
F34D6.8
T05A8.1
T05A8.2
T05A8.3
T05A8.8
T05A8.8
T05A8.8
T05A8.8
F29A7.3
F29A7.3
F29A7.3
F29A7.3
F29A7.3
fbxb−106
F08D12.13
K12H6.8
K12H6.6
Y110A2AL.4
Y110A2AL.4
Y110A2AL.6
Y110A2AL.7
Y110A2AL.10
Y110A2AL.1
Y110A2AL.12
Y110A2AL.12
sqv−2
Y110A2AM.4
Y110A2AM.1
ZK355.7
ZK355.5
srw−79
T11F1.3
nhr−88

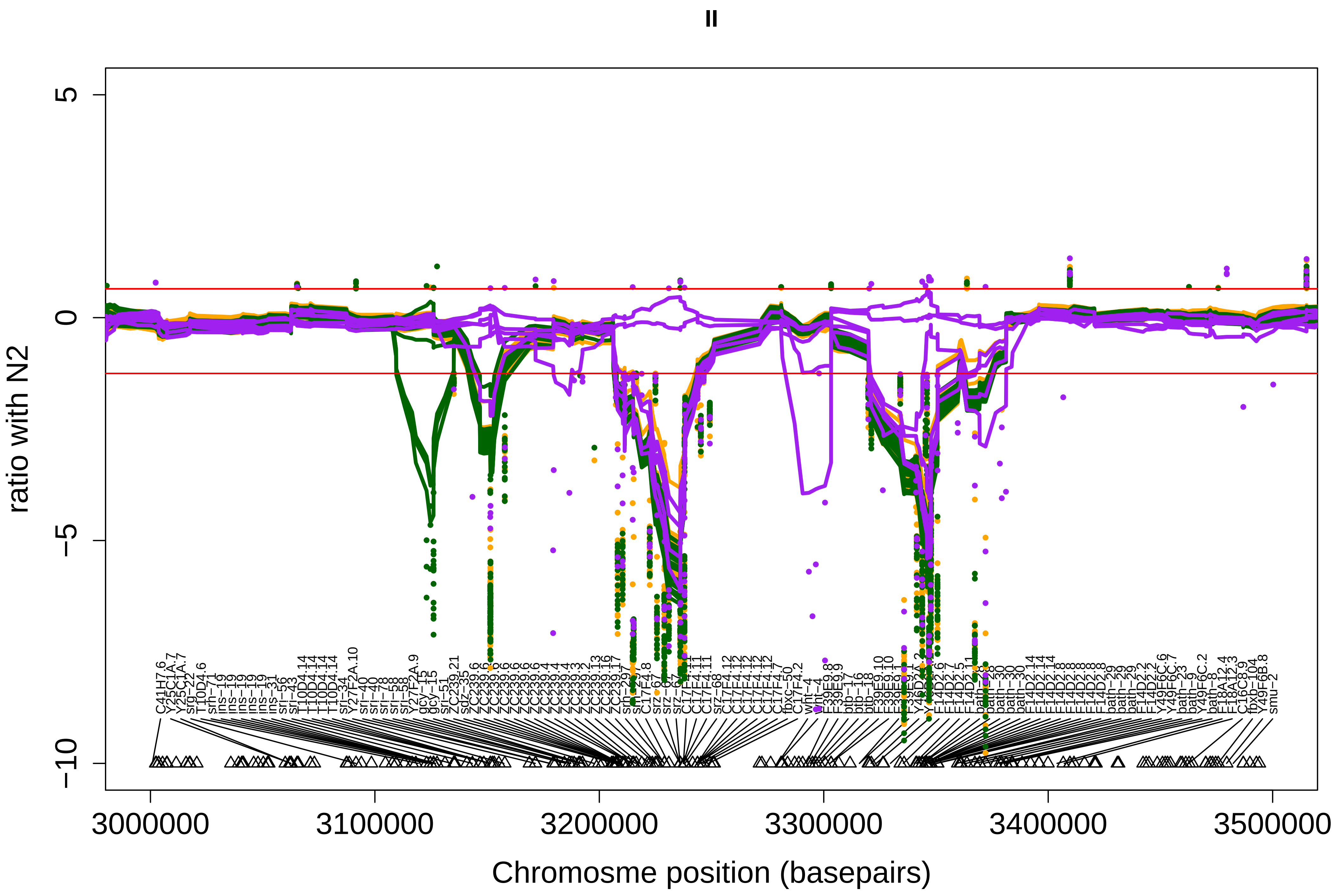
II
ratio with N2
Chromosme position (basepairs)
5
0
−5
−10
3000000
3100000
3200000
3300000
3400000
3500000

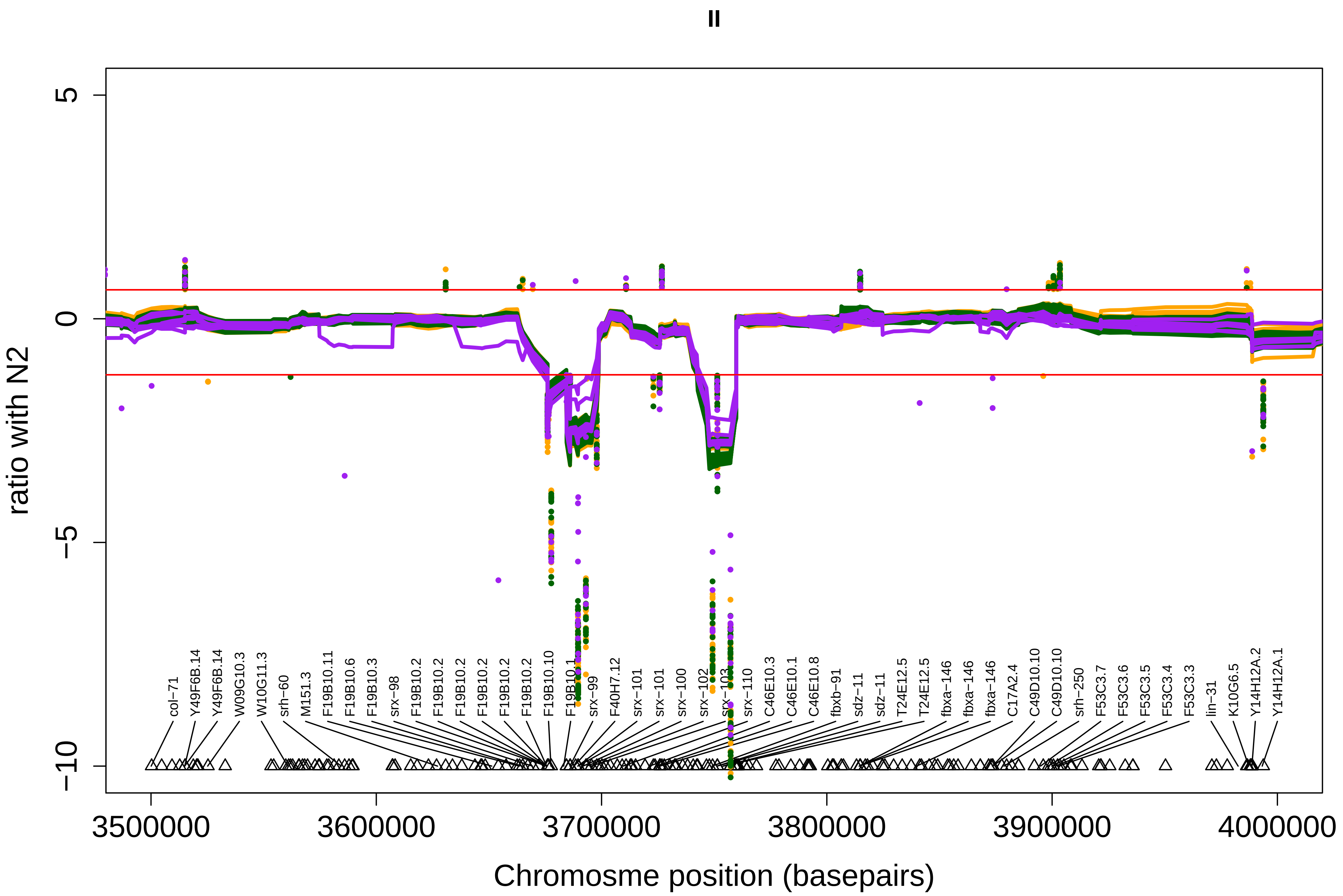
II
ratio with N2
5
0
−5
−10
3500000
3600000
3700000
3800000
3900000
4000000
Chromosme position (basepairs)
col−71
Y49F6B.14
Y49F6B.14
W09G10.3
W10G11.3
srh−60
M151.3
F19B10.11
F19B10.6
F19B10.3
srx−98
F19B10.2
F19B10.2
F19B10.2
F19B10.2
F19B10.2
F19B10.2
F19B10.10
F19B10.1
srx−99
F40H7.12
srx−101
srx−101
srx−100
srx−102
srx−103
srx−110
C46E10.3
C46E10.1
C46E10.8
fbxb−91
sdz−11
sdz−11
T24E12.5
T24E12.5
fbxa−146
fbxa−146
fbxa−146
C17A2.4
C49D10.10
C49D10.10
srh−250
F53C3.7
F53C3.6
F53C3.5
F53C3.4
F53C3.3
lin−31
K10G6.5
Y14H12A.2
Y14H12A.1

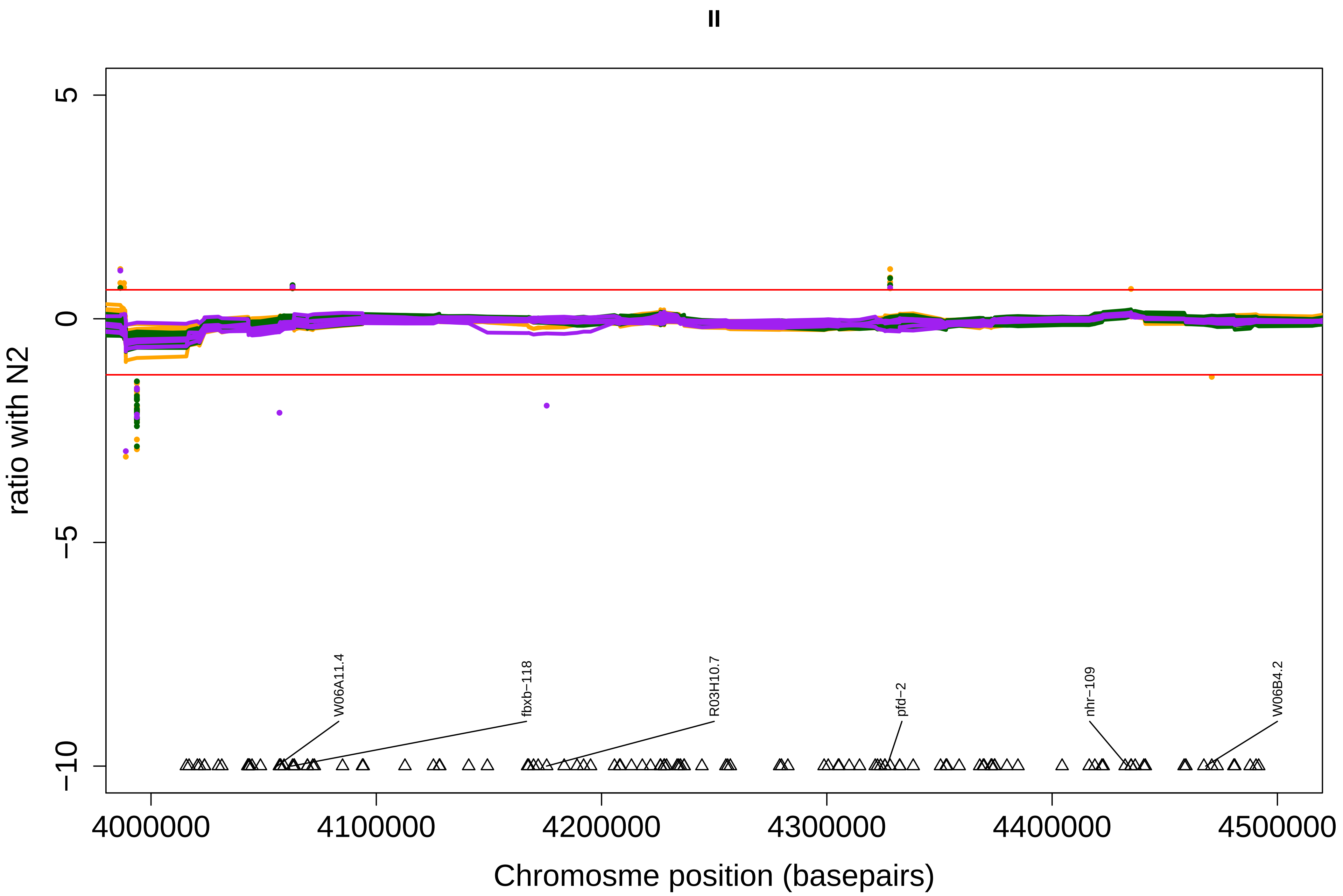

II
ratio with N2
5
0
−5
−10
4000000
4100000
4200000
4300000
4400000
4500000
Chromosme position (basepairs)
W06A11.4
fbxb−118
R03H10.7
pfd−2
nhr−109
W06B4.2

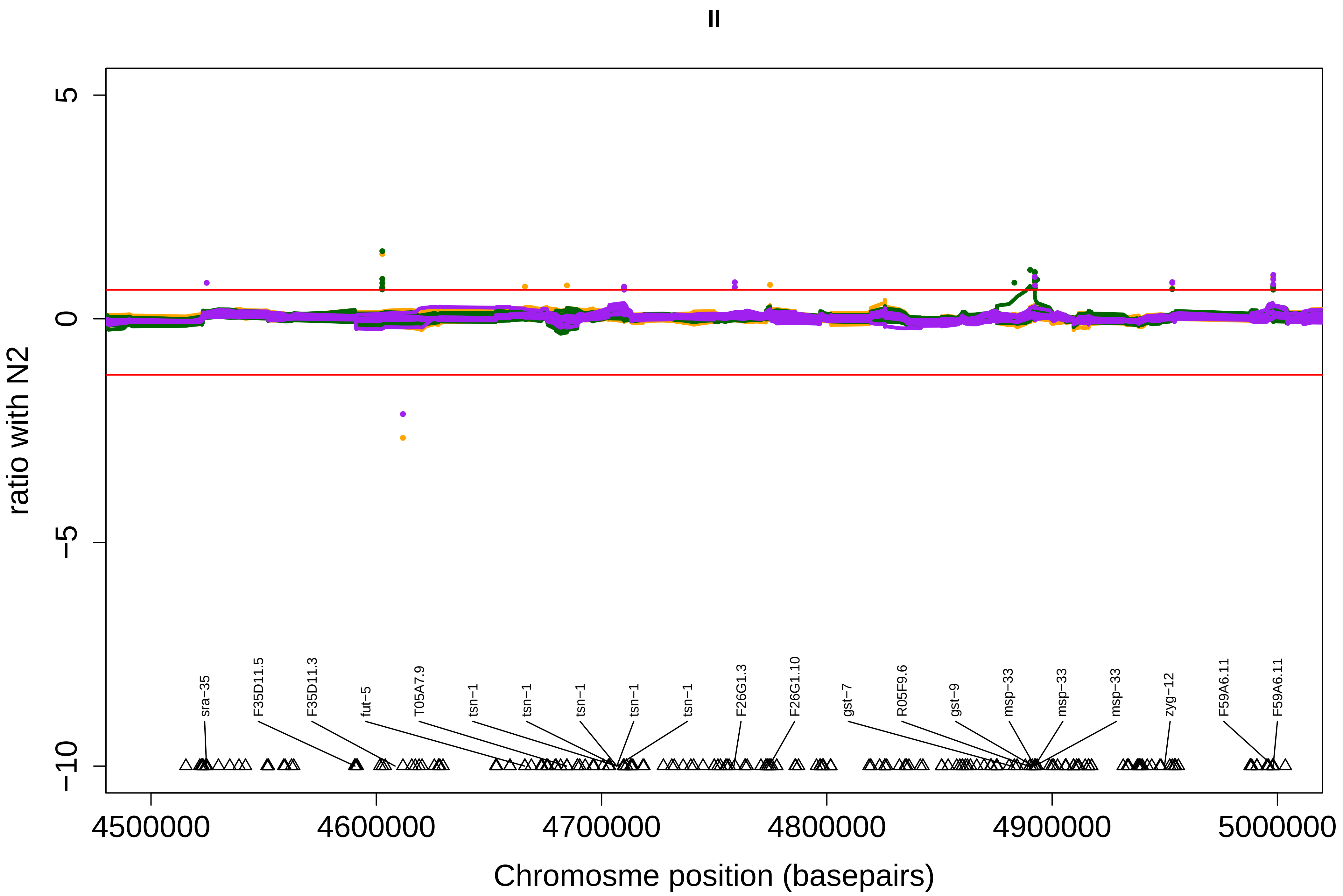

II
5
0
−5
−10
ratio with N2
sra−35
F35D11.5
F35D11.3
fut−5
T05A7.9
tsn−1
tsn−1
tsn−1
tsn−1
tsn−1
F26G1.3
F26G1.10
gst−7
R05F9.6
gst−9
msp−33
msp−33
msp−33
zyg−12
F59A6.11
F59A6.11
4500000
4600000
4700000
4800000
4900000
5000000
Chromosme position (basepairs)

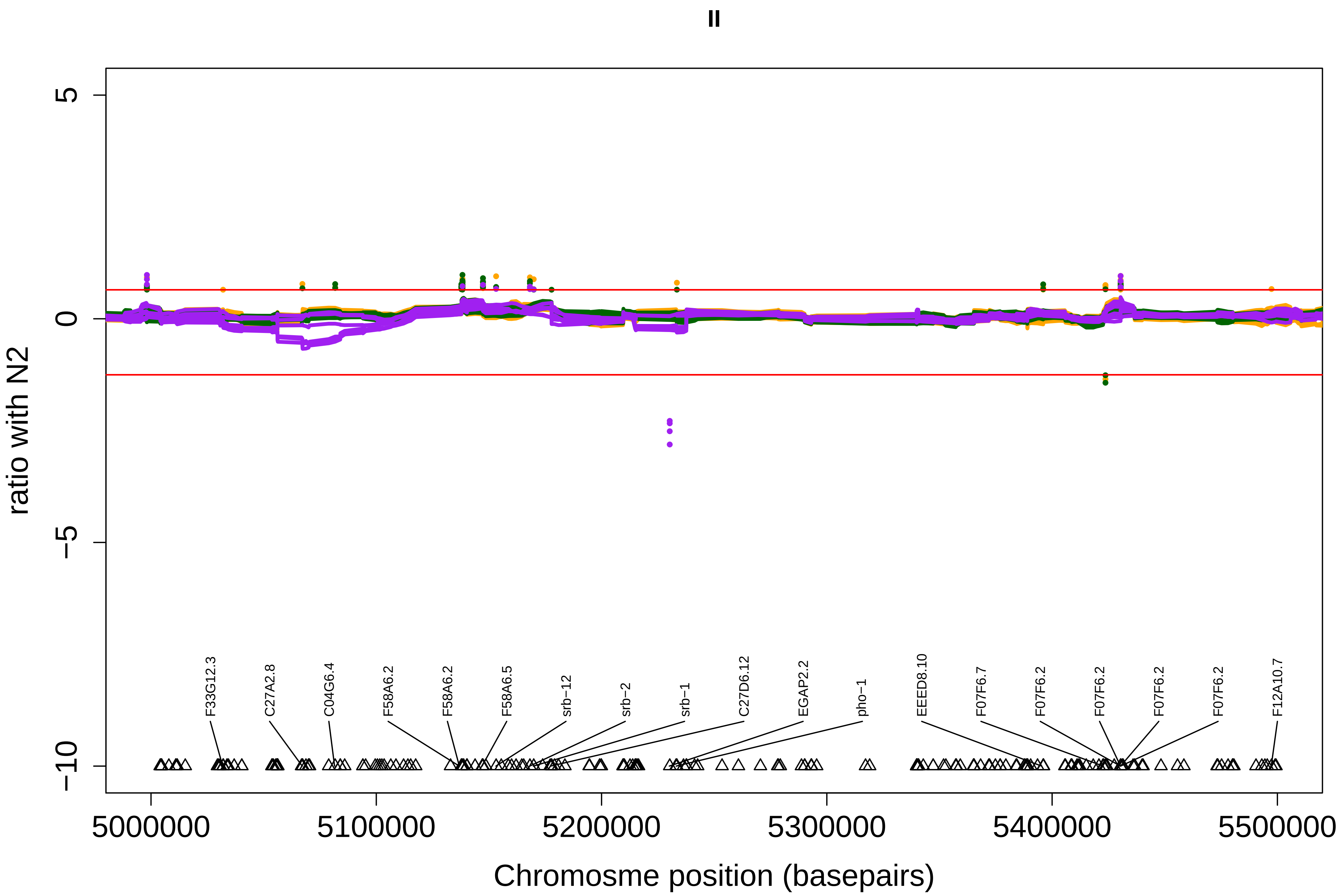
II
ratio with N2
5
0
−5
−10
F33G12.3
C27A2.8
C04G6.4
F58A6.2
F58A6.2
F58A6.5
srb−12
srb−2
srb−1
C27D6.12
EGAP2.2
pho−1
EEED8.10
F07F6.7
F07F6.2
F07F6.2
F07F6.2
F07F6.2
F12A10.7
5000000
5100000
5200000
5300000
5400000
5500000
Chromosme position (basepairs)

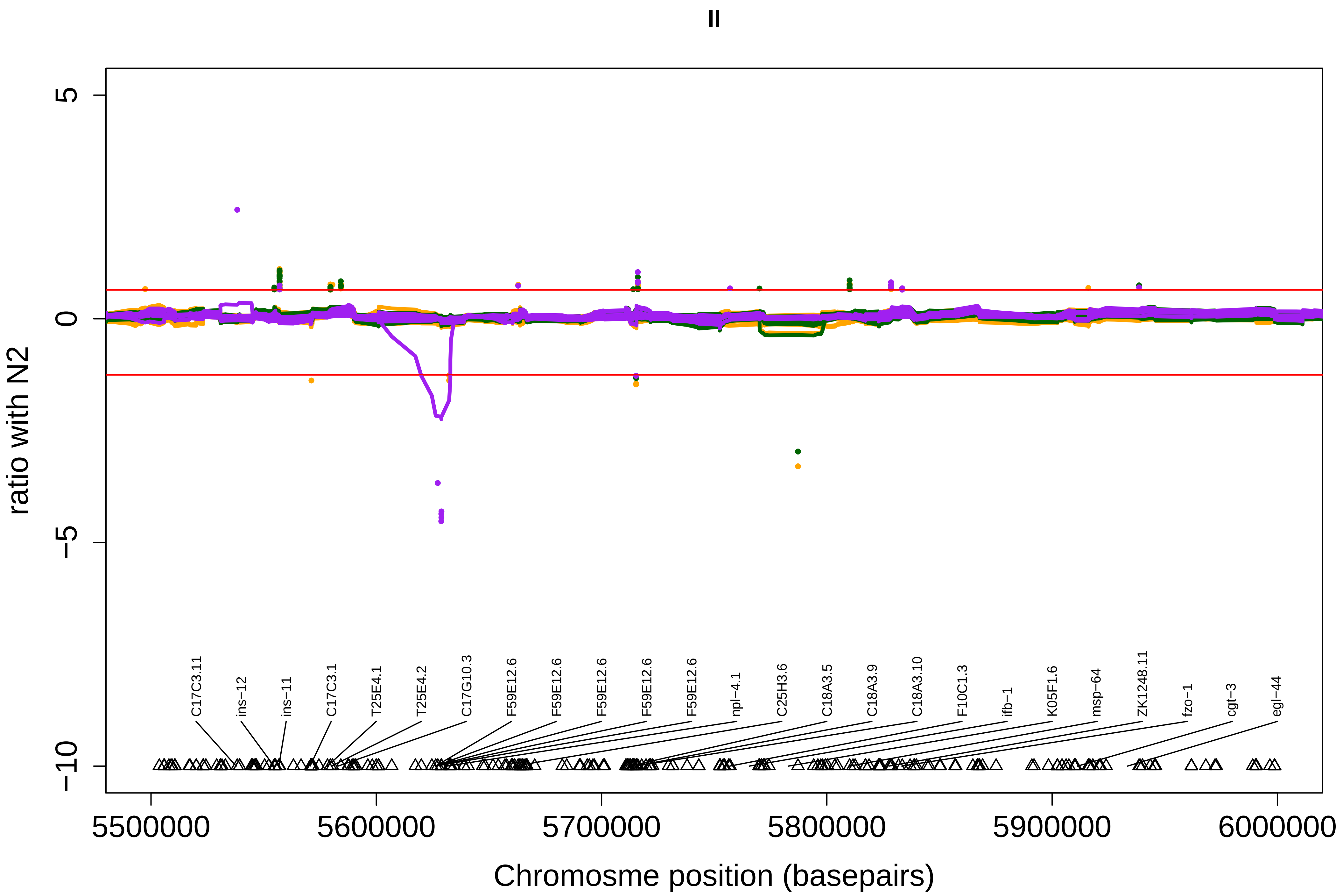
II
ratio with N2
5
0
−5
−10
C17C3.11
ins−12
ins−11
C17C3.1
T25E4.1
T25E4.2
C17G10.3
F59E12.6
F59E12.6
F59E12.6
F59E12.6
F59E12.6
npl−4.1
C25H3.6
C18A3.5
C18A3.9
C18A3.10
F10C1.3
ifb−1
K05F1.6
msp−64
ZK1248.11
fzo−1
cgt−3
egl−44
5500000
5600000
5700000
5800000
5900000
6000000
Chromosme position (basepairs)

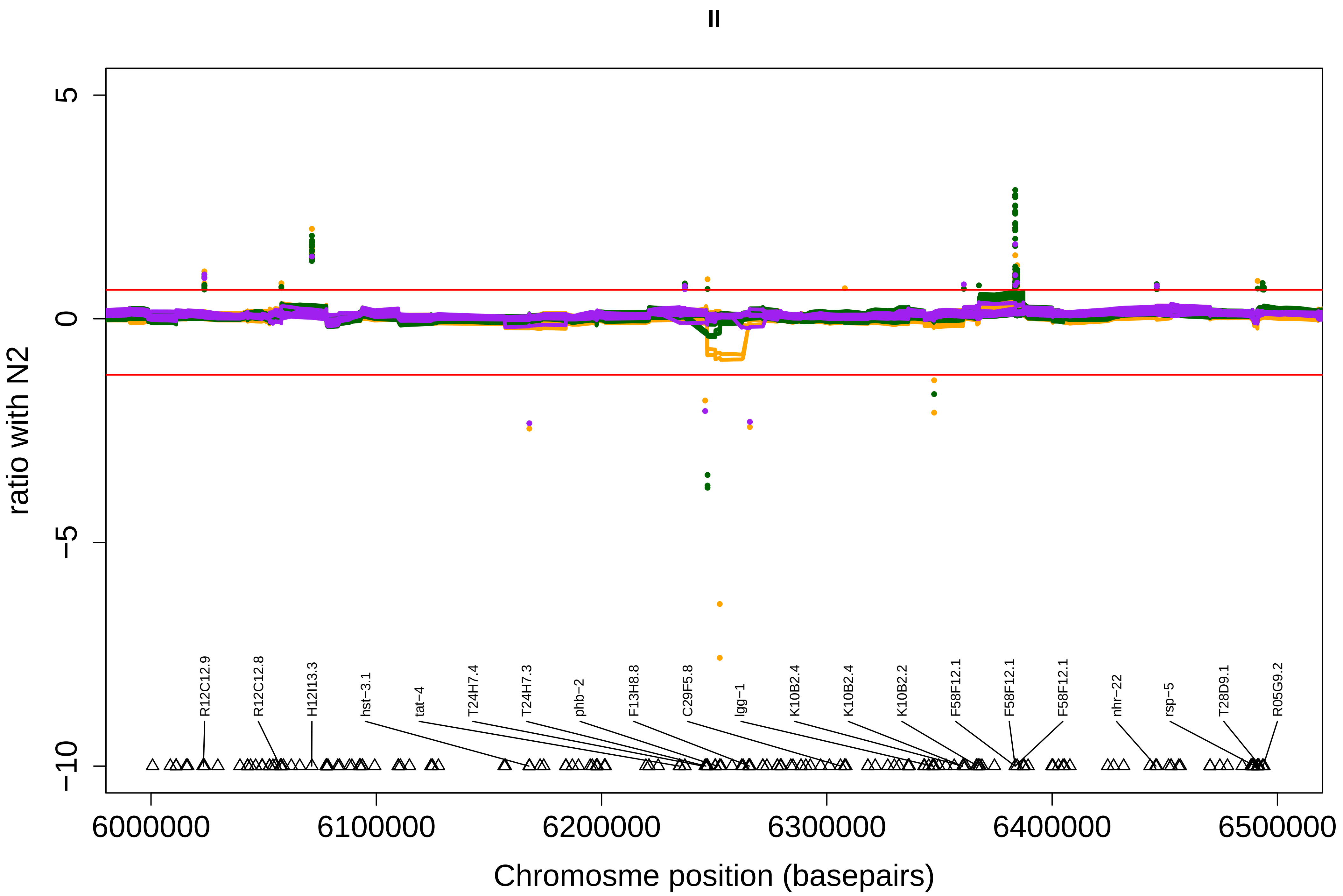

II
ratio with N2
5
0
−5
−10
R12C12.9
R12C12.8
H12I13.3
hst−3.1
tat−4
T24H7.4
T24H7.3
phb−2
F13H8.8
C29F5.8
lgg−1
K10B2.4
K10B2.4
K10B2.2
F58F12.1
F58F12.1
F58F12.1
nhr−22
rsp−5
T28D9.1
R05G9.2
6000000
6100000
6200000
6300000
6400000
6500000
Chromosme position (basepairs)

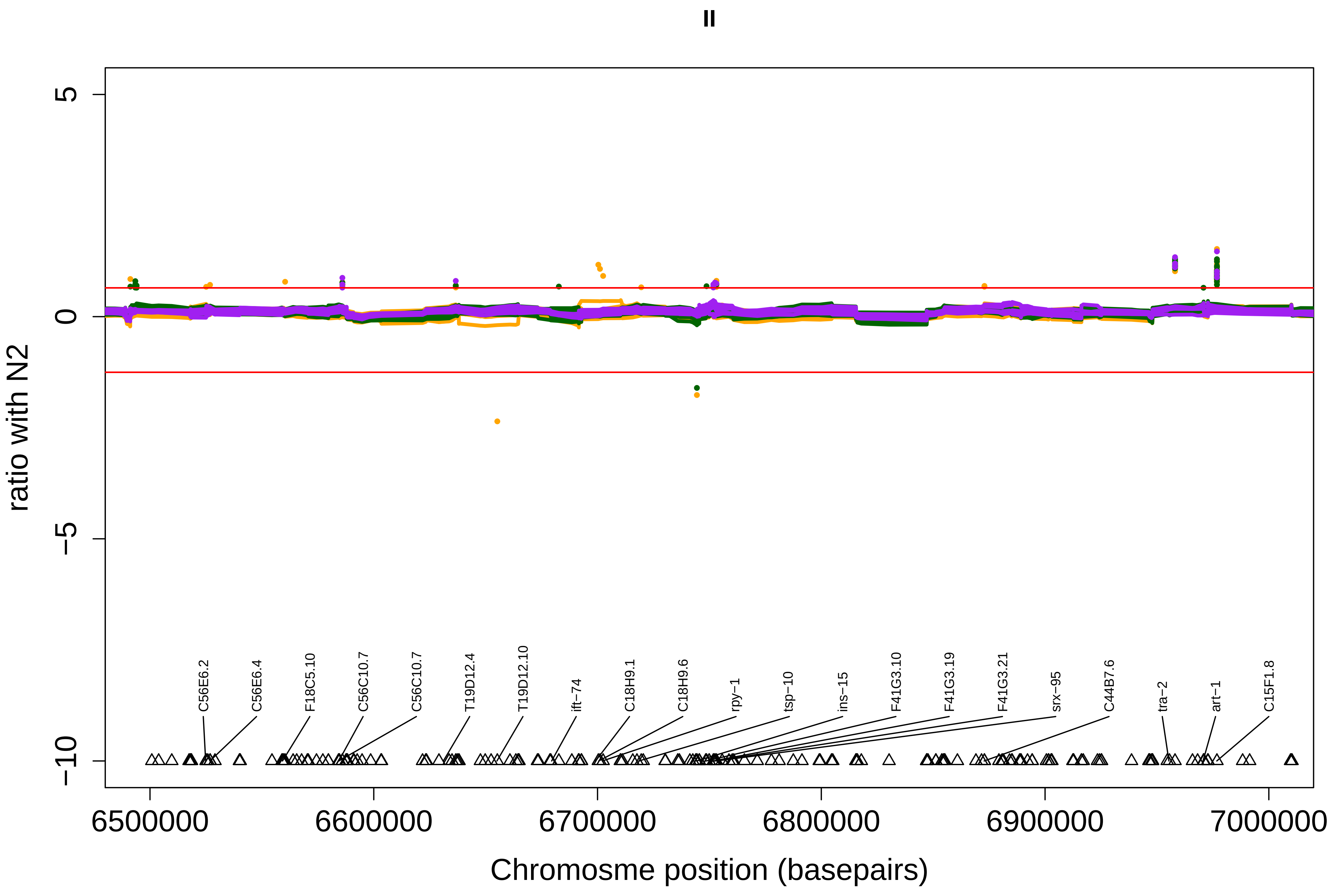

II
ratio with N2
5
0
−5
−10
Chromosme position (basepairs)
6500000
6600000
6700000
6800000
6900000
7000000
C56E6.2
C56E6.4
F18C5.10
C56C10.7
C56C10.7
T19D12.4
T19D12.10
ift−74
C18H9.1
C18H9.6
rpy−1
tsp−10
ins−15
F41G3.10
F41G3.19
F41G3.21
srx−95
C44B7.6
tra−2
art−1
C15F1.8

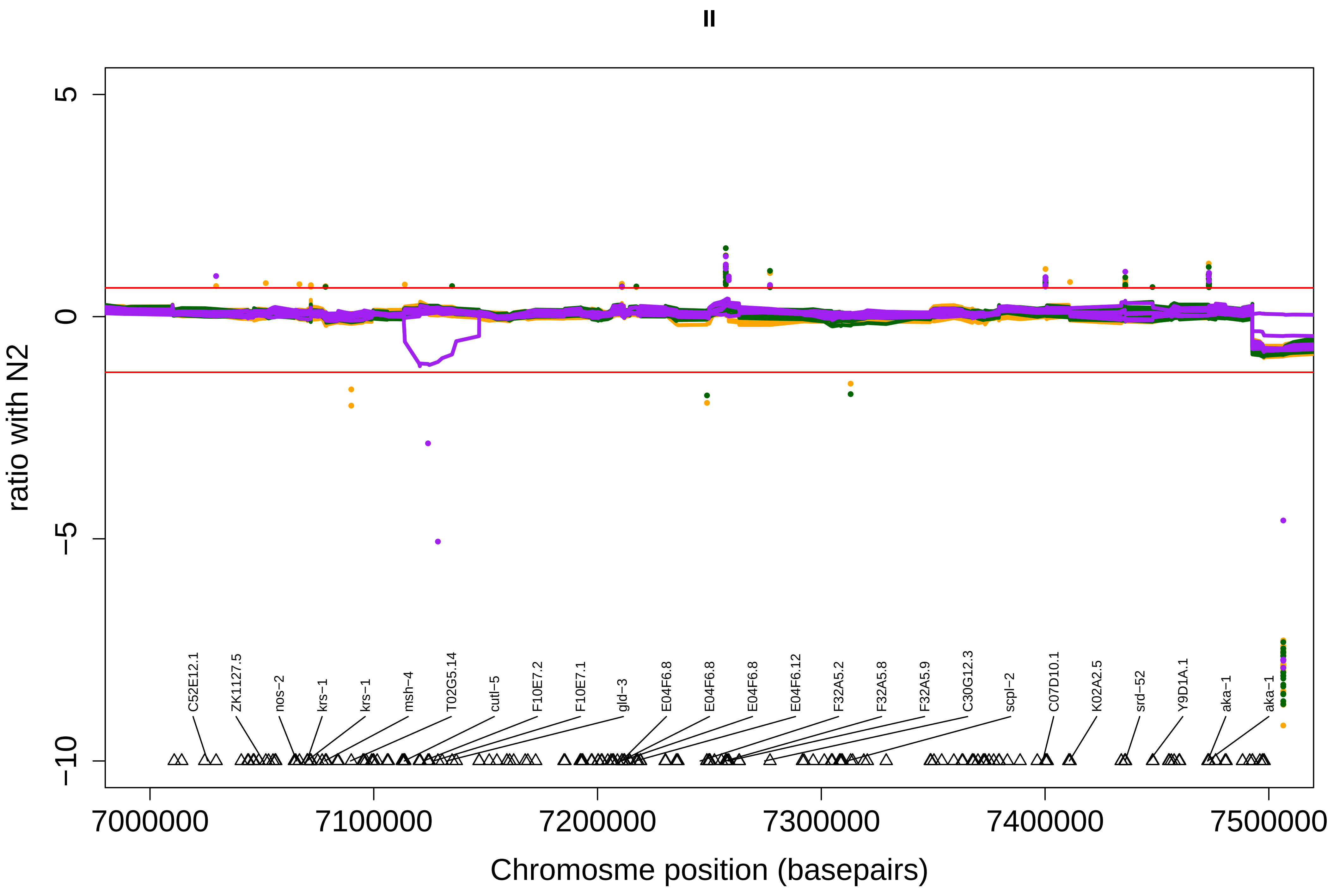
II
ratio with N2
5
0
−5
−10
C52E12.1
ZK1127.5
nos−2
krs−1
krs−1
msh−4
T02G5.14
cutl−5
F10E7.2
F10E7.1
gld−3
E04F6.8
E04F6.8
E04F6.8
E04F6.12
F32A5.2
F32A5.8
F32A5.9
C30G12.3
scpl−2
C07D10.1
K02A2.5
srd−52
Y9D1A.1
aka−1
aka−1
7000000
7100000
7200000
7300000
7400000
7500000
Chromosme position (basepairs)

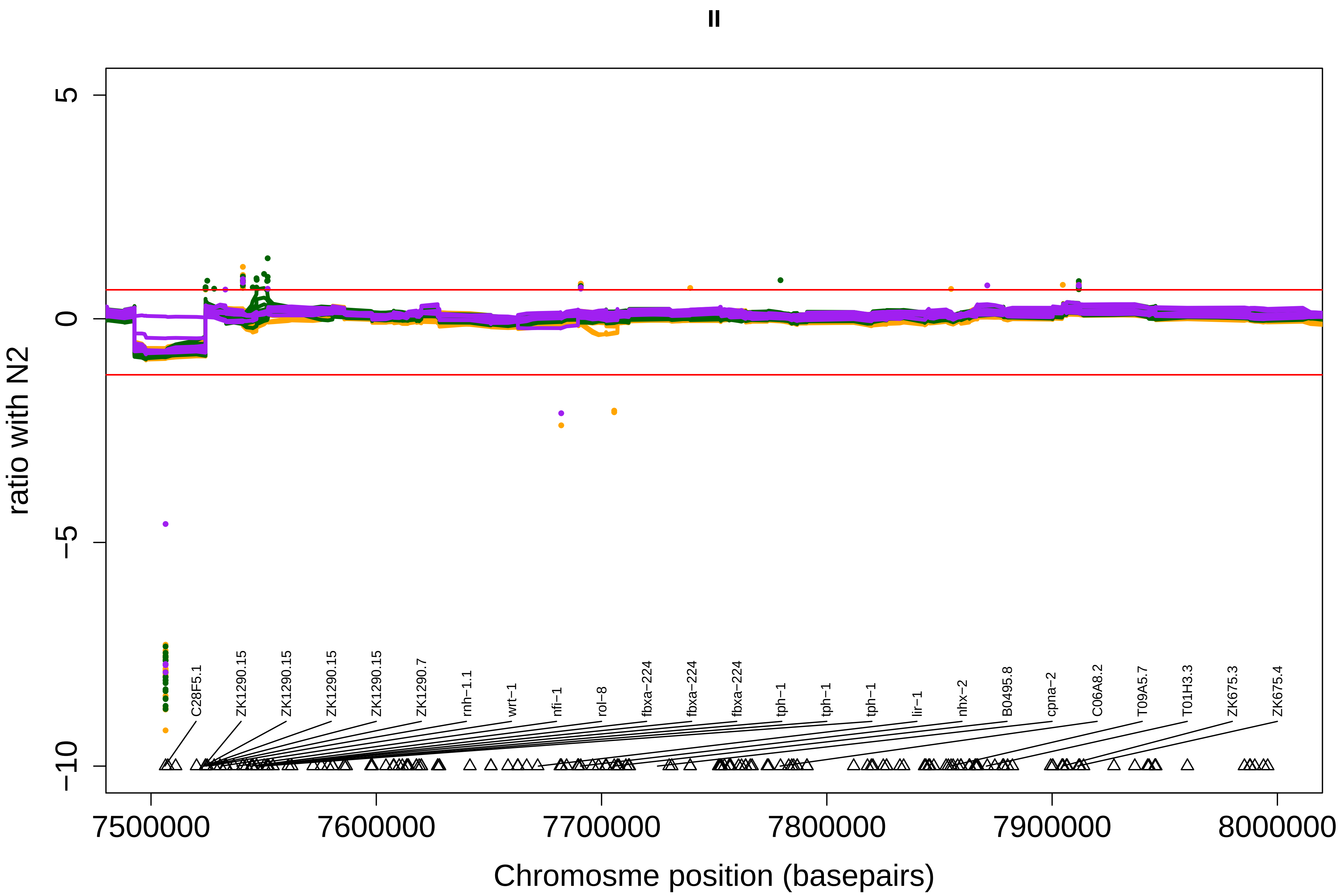
II
ratio with N2
5
0
−5
−10
C28F5.1
ZK1290.15
ZK1290.15
ZK1290.15
ZK1290.15
ZK1290.7
rnh−1.1
wrt−1
nfi−1
rol−8
fbxa−224
fbxa−224
fbxa−224
tph−1
tph−1
tph−1
lir−1
nhx−2
B0495.8
cpna−2
C06A8.2
T09A5.7
T01H3.3
ZK675.3
ZK675.4
7500000
7600000
7700000
7800000
7900000
8000000
Chromosme position (basepairs)

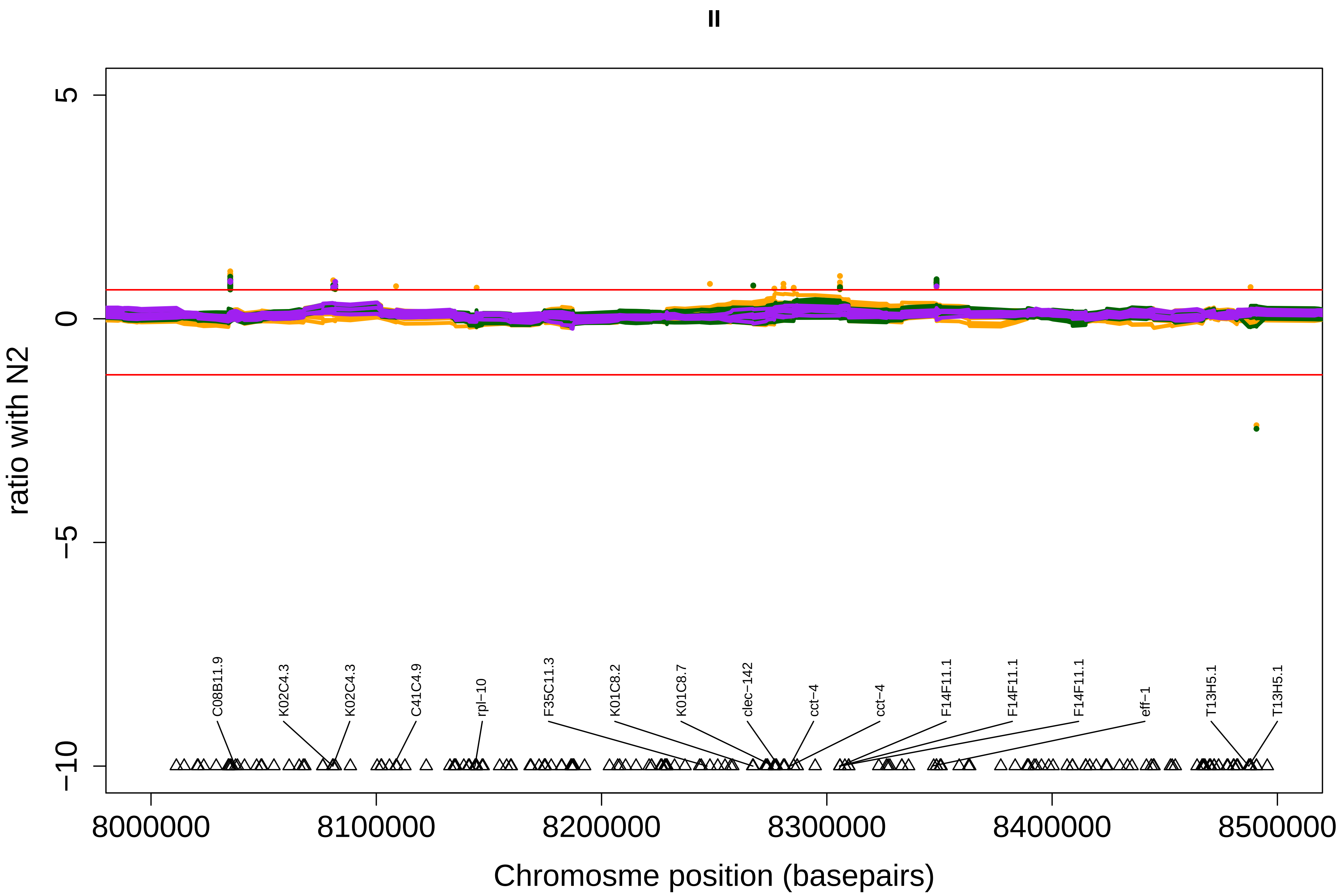

II
5
0
−5
−10
ratio with N2
C08B11.9
K02C4.3
K02C4.3
C41C4.9
rpl−10
F35C11.3
K01C8.2
K01C8.7
clec−142
cct−4
cct−4
F14F11.1
F14F11.1
F14F11.1
eff−1
T13H5.1
T13H5.1
8000000
8100000
8200000
8300000
8400000
8500000
Chromosme position (basepairs)

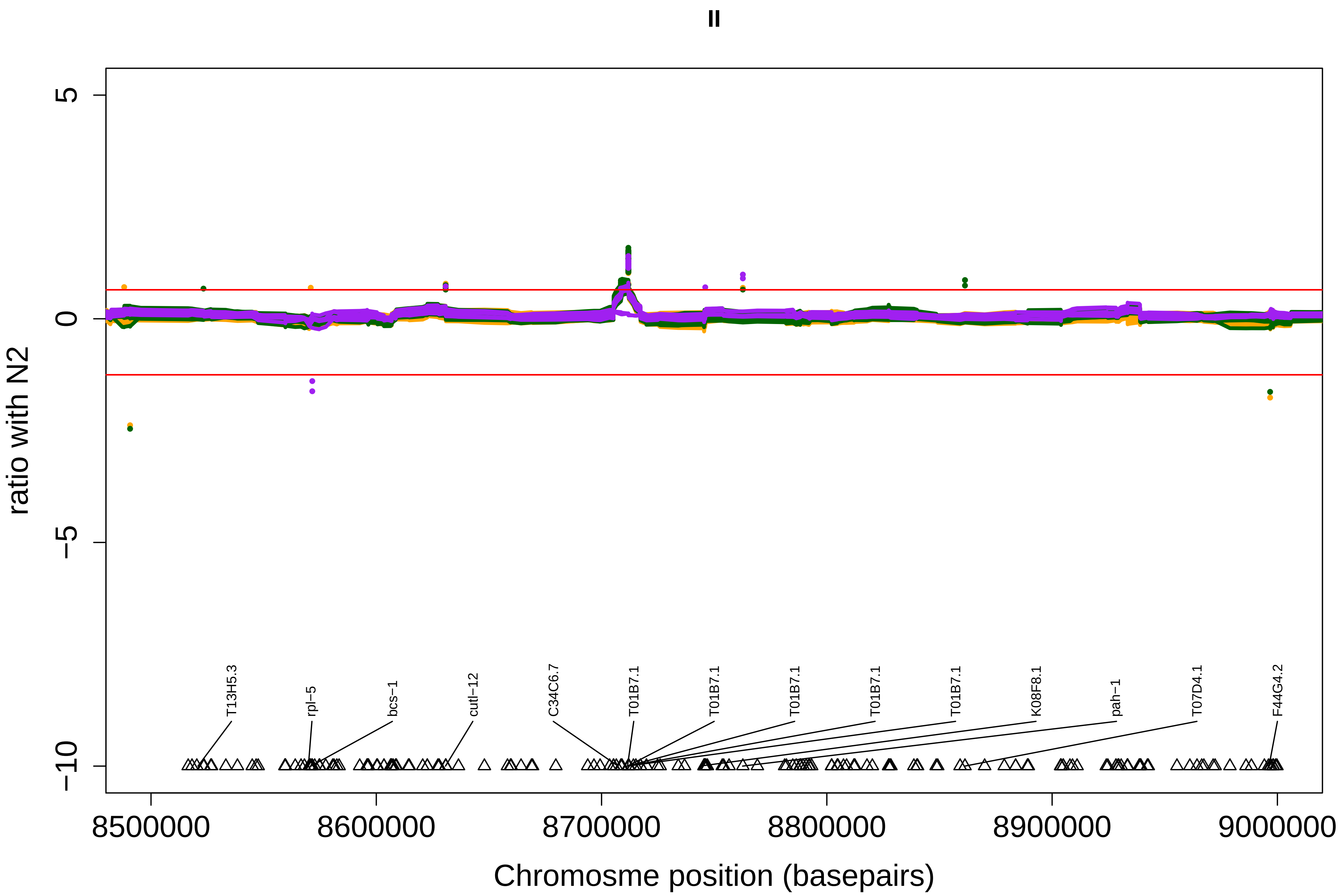

II
ratio with N2
5
0
−5
−10
T13H5.3
rpl−5
bcs−1
cutl−12
C34C6.7
T01B7.1
T01B7.1
T01B7.1
T01B7.1
T01B7.1
K08F8.1
pah−1
T07D4.1
F44G4.2
8500000
8600000
8700000
8800000
8900000
9000000
Chromosme position (basepairs)

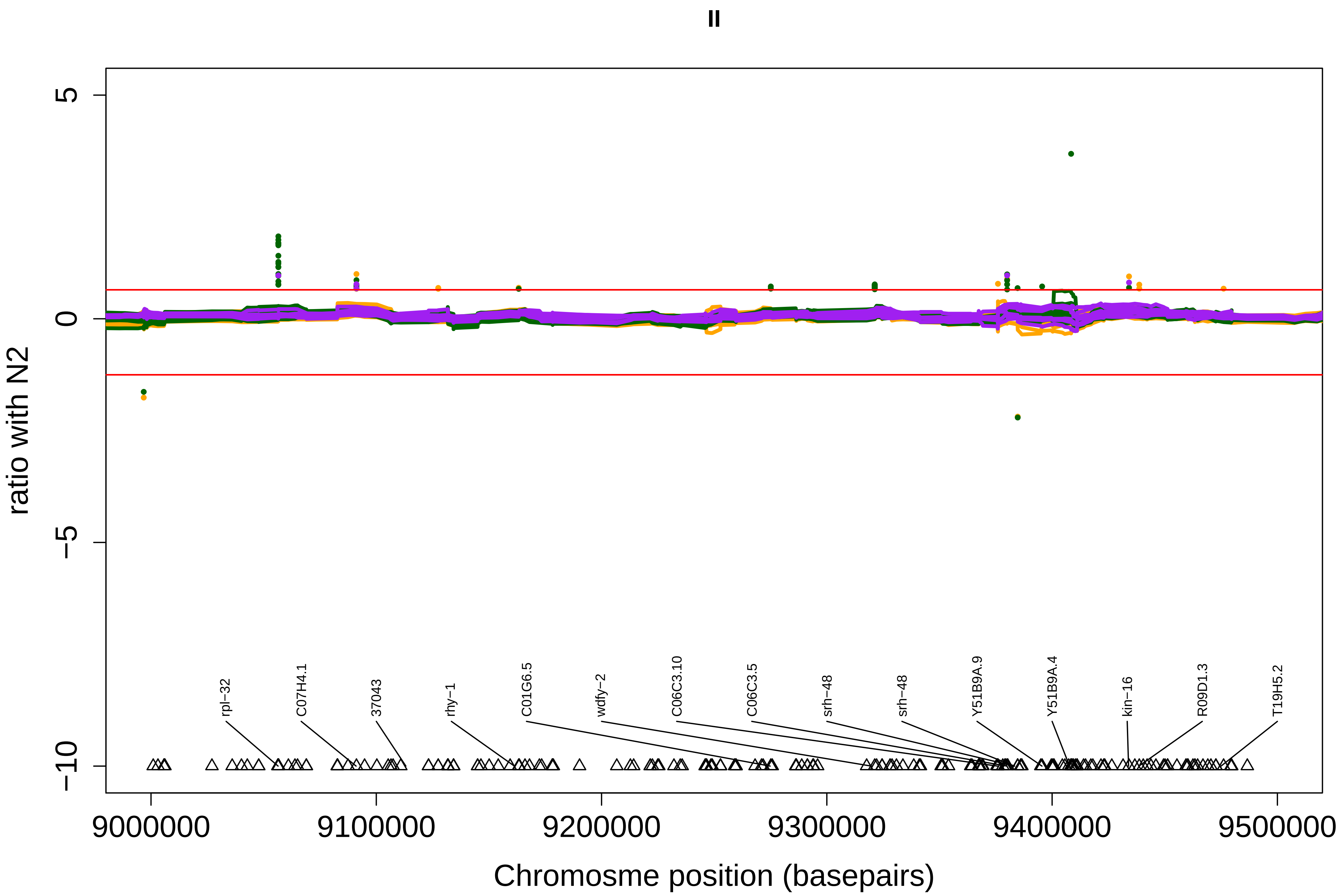
II
ratio with N2
5
0
−5
−10
9000000
9100000
9200000
9300000
9400000
9500000
Chromosme position (basepairs)
rpl−32
C07H4.1
37043
rhy−1
C01G6.5
wdfy−2
C06C3.10
C06C3.5
srh−48
srh−48
Y51B9A.9
Y51B9A.4
kin−16
R09D1.3
T19H5.2

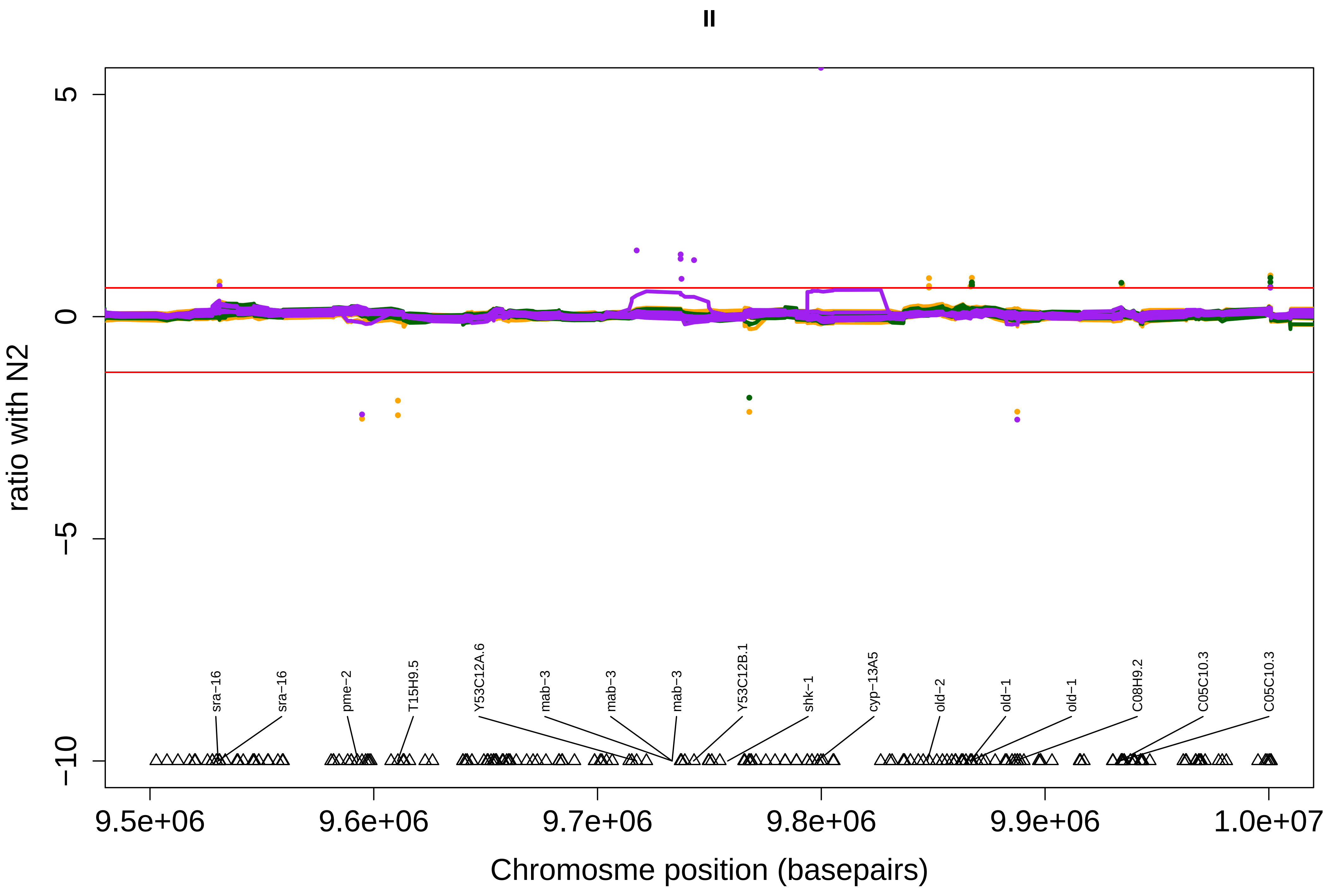

II
ratio with N2
5
0
−5
−10
9.5e+06
9.6e+06
9.7e+06
9.8e+06
9.9e+06
1.0e+07
Chromosme position (basepairs)
sra−16
sra−16
pme−2
T15H9.5
Y53C12A.6
mab−3
mab−3
mab−3
Y53C12B.1
shk−1
cyp−13A5
old−2
old−1
old−1
C08H9.2
C05C10.3
C05C10.3

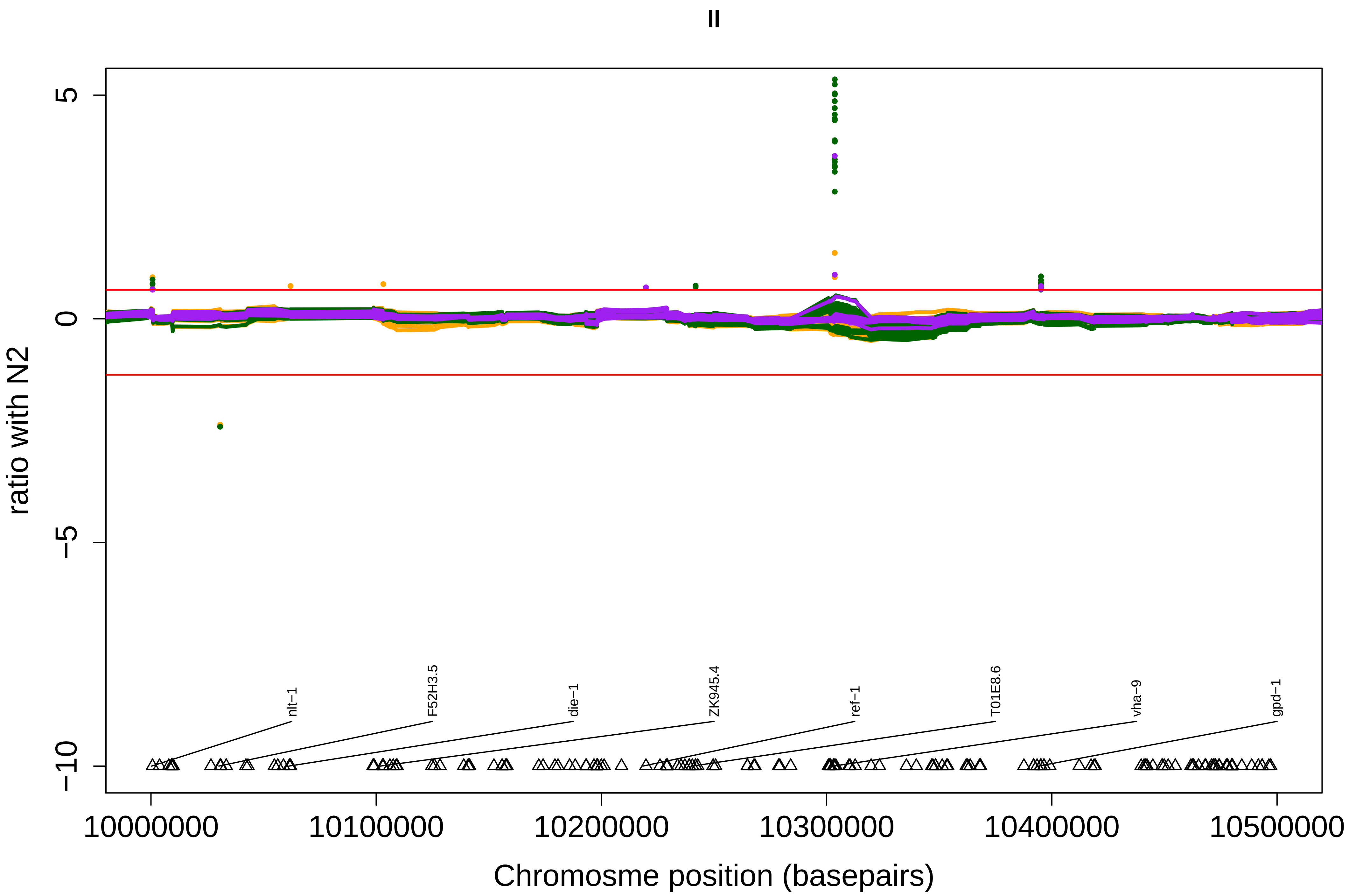

II
ratio with N2
5
0
−5
−10
nlt−1
F52H3.5
die−1
ZK945.4
ref−1
T01E8.6
vha−9
gpd−1
10000000
10100000
10200000
10300000
10400000
10500000
Chromosme position (basepairs)

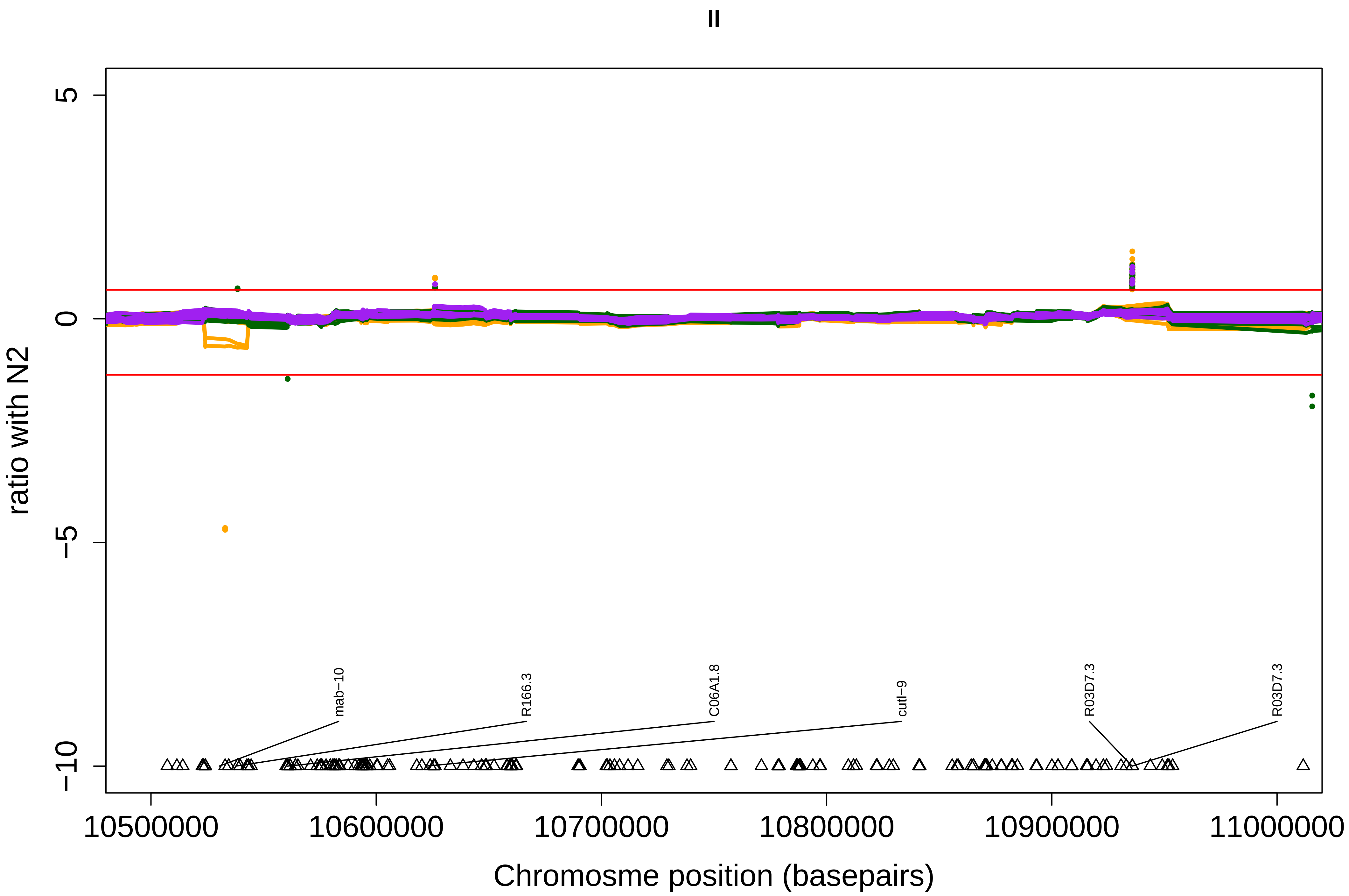
II
ratio with N2
5
0
−5
−10
mab−10
R166.3
C06A1.8
cutl−9
R03D7.3
R03D7.3
10500000
10600000
10700000
10800000
10900000
11000000
Chromosme position (basepairs)

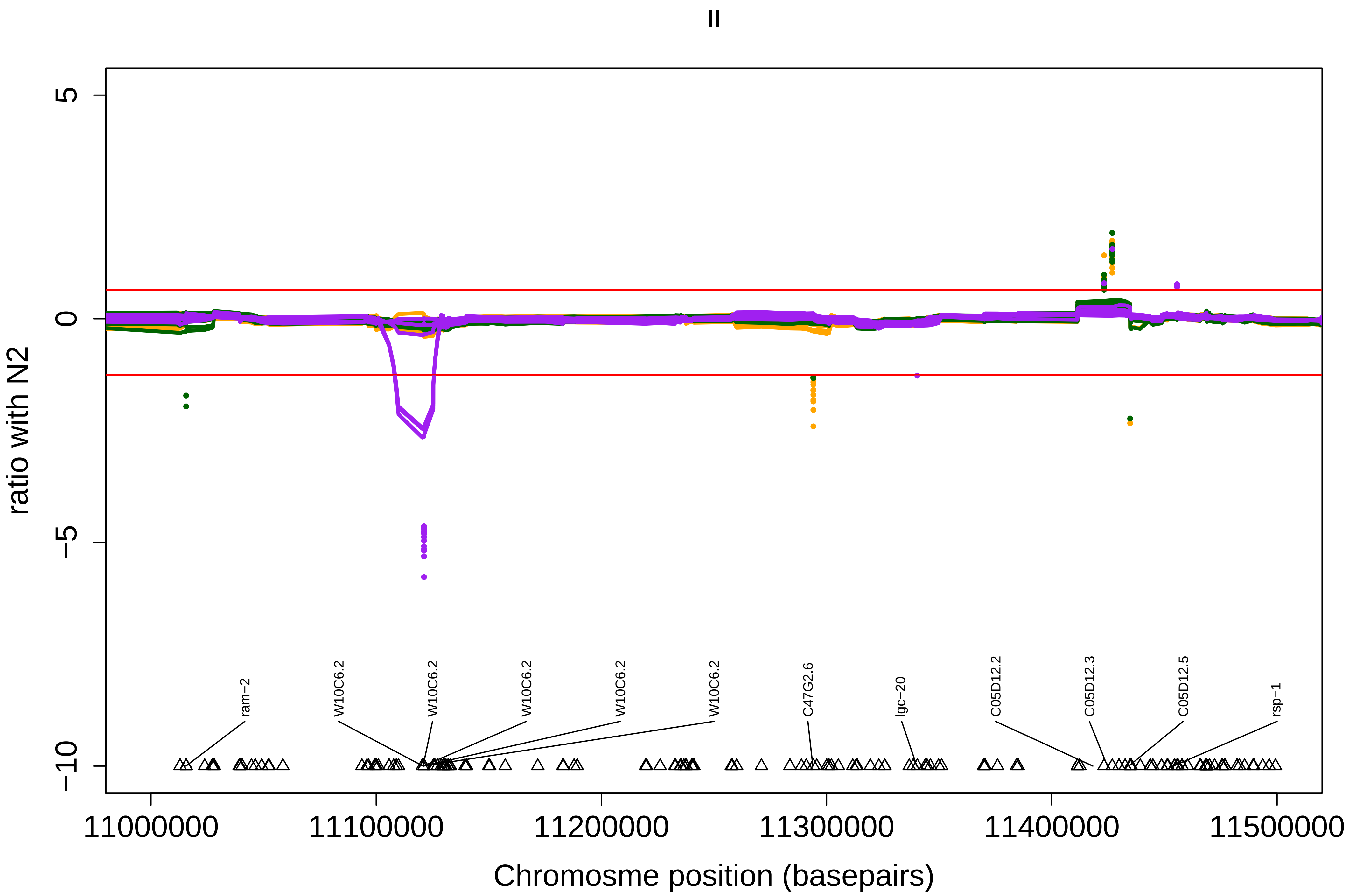

II
ratio with N2
5
0
−5
−10
ram−2
W10C6.2
W10C6.2
W10C6.2
W10C6.2
W10C6.2
C47G2.6
lgc−20
C05D12.2
C05D12.3
C05D12.5
rsp−1
11000000
11100000
11200000
11300000
11400000
11500000
Chromosme position (basepairs)

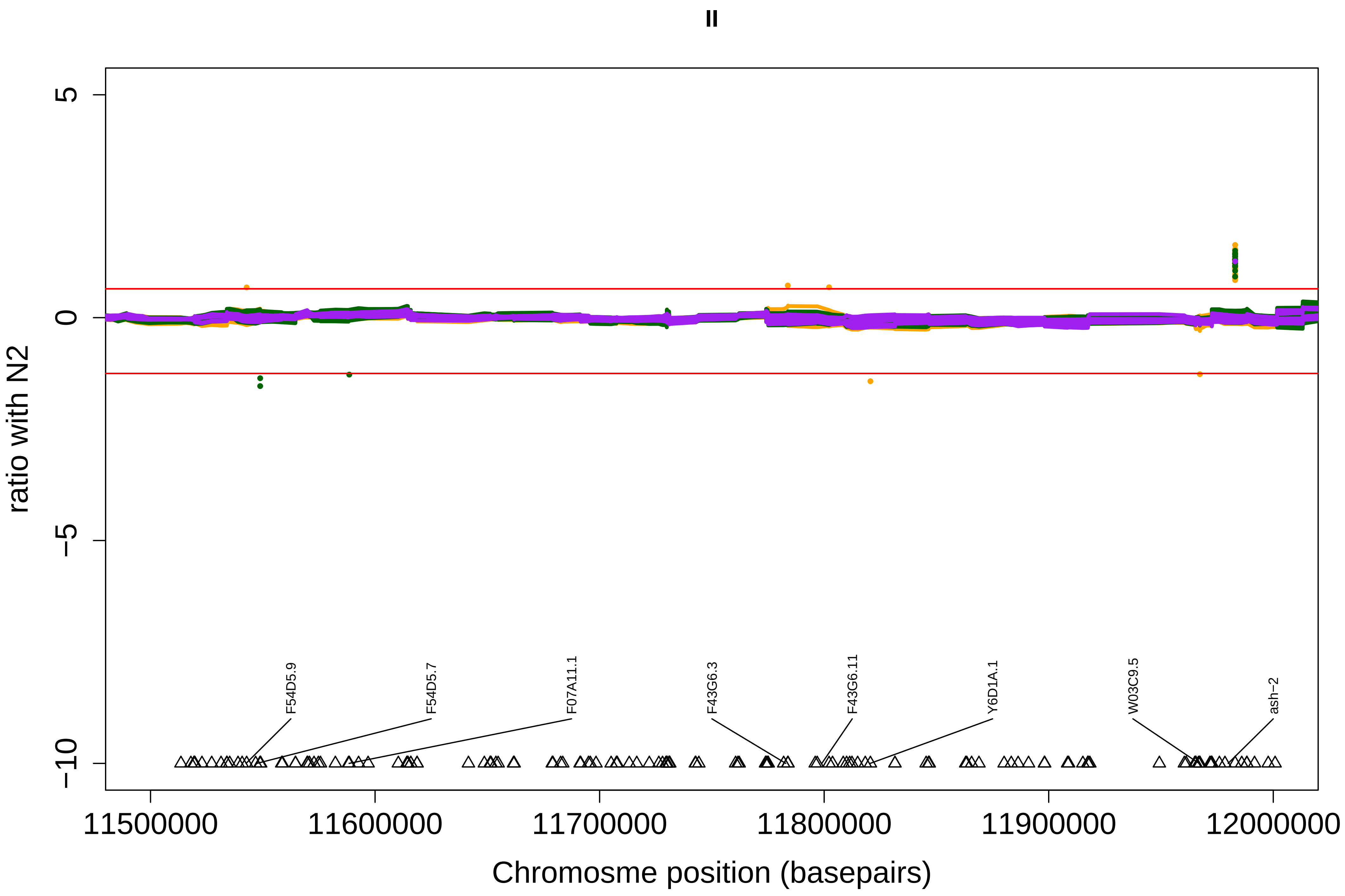
II
ratio with N2
5
0
−5
−10
F54D5.9
F54D5.7
F07A11.1
F43G6.3
F43G6.11
Y6D1A.1
W03C9.5
ash−2
11500000
11600000
11700000
11800000
11900000
12000000
Chromosme position (basepairs)

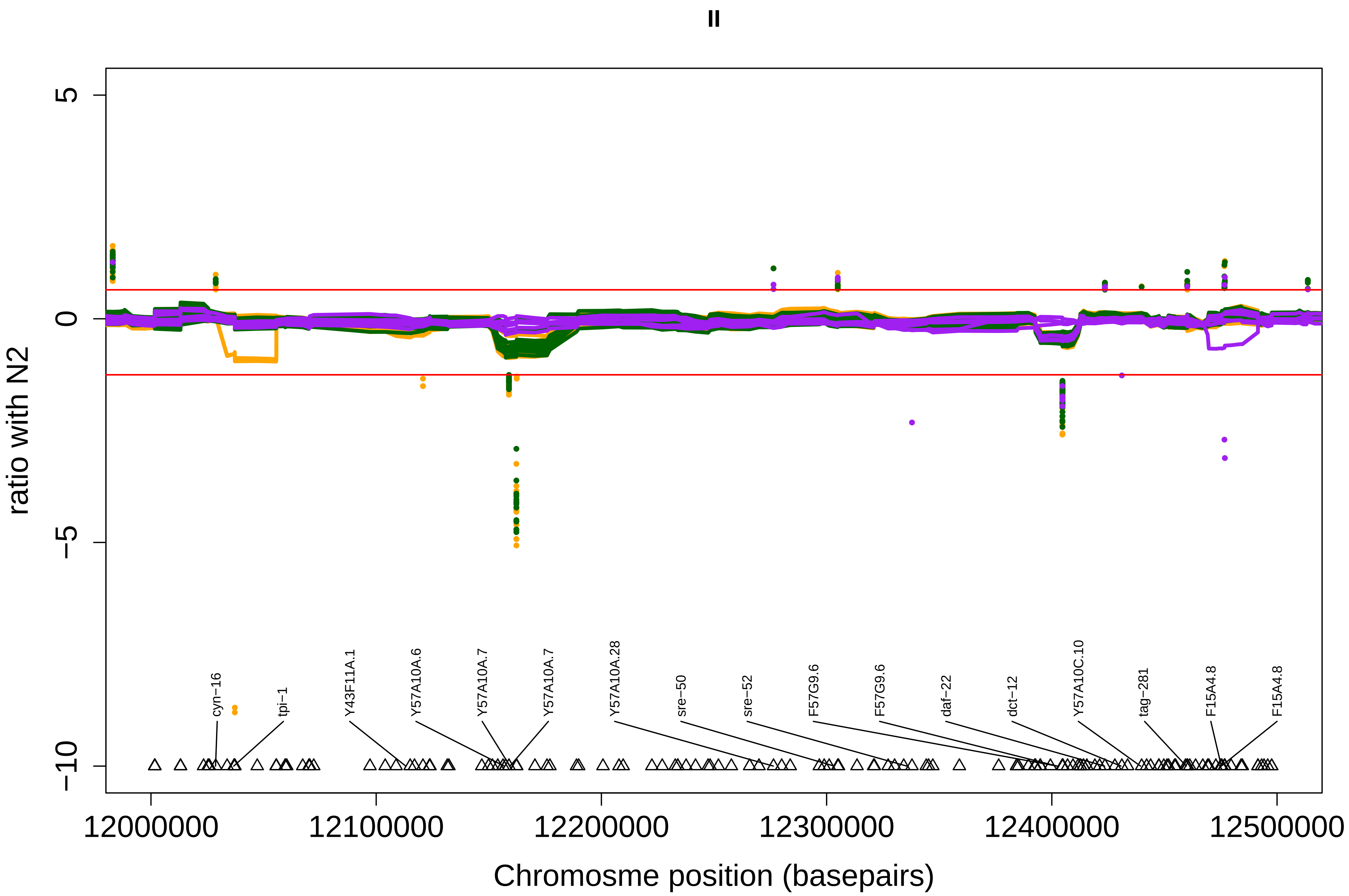
II
ratio with N2
5
0
−5
−10
cyn−16
tpi−1
Y43F11A.1
Y57A10A.6
Y57A10A.7
Y57A10A.7
Y57A10A.28
sre−50
sre−52
F57G9.6
F57G9.6
daf−22
dct−12
Y57A10C.10
tag−281
F15A4.8
F15A4.8
12000000
12100000
12200000
12300000
12400000
12500000
Chromosme position (basepairs)

II

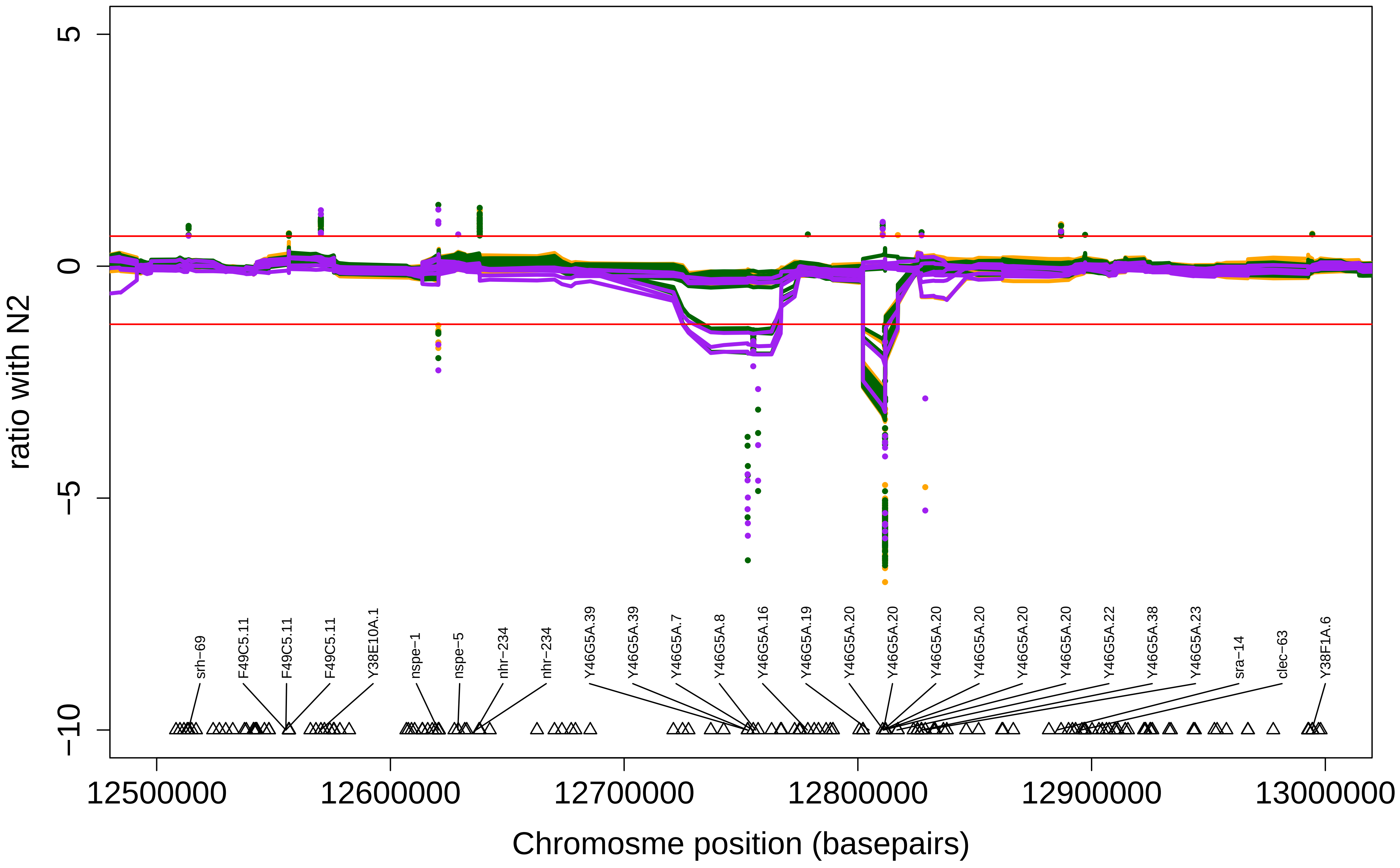
ratio with N2
5
0
−5
−10
srh−69
F49C5.11
F49C5.11
F49C5.11
Y38E10A.1
nspe−1
nspe−5
nhr−234
nhr−234
Y46G5A.39
Y46G5A.39
Y46G5A.7
Y46G5A.8
Y46G5A.16
Y46G5A.19
Y46G5A.20
Y46G5A.20
Y46G5A.20
Y46G5A.20
Y46G5A.20
Y46G5A.20
Y46G5A.22
Y46G5A.38
Y46G5A.23
sra−14
clec−63
Y38F1A.6
12500000
12600000
12700000
12800000
12900000
13000000
Chromosme position (basepairs)

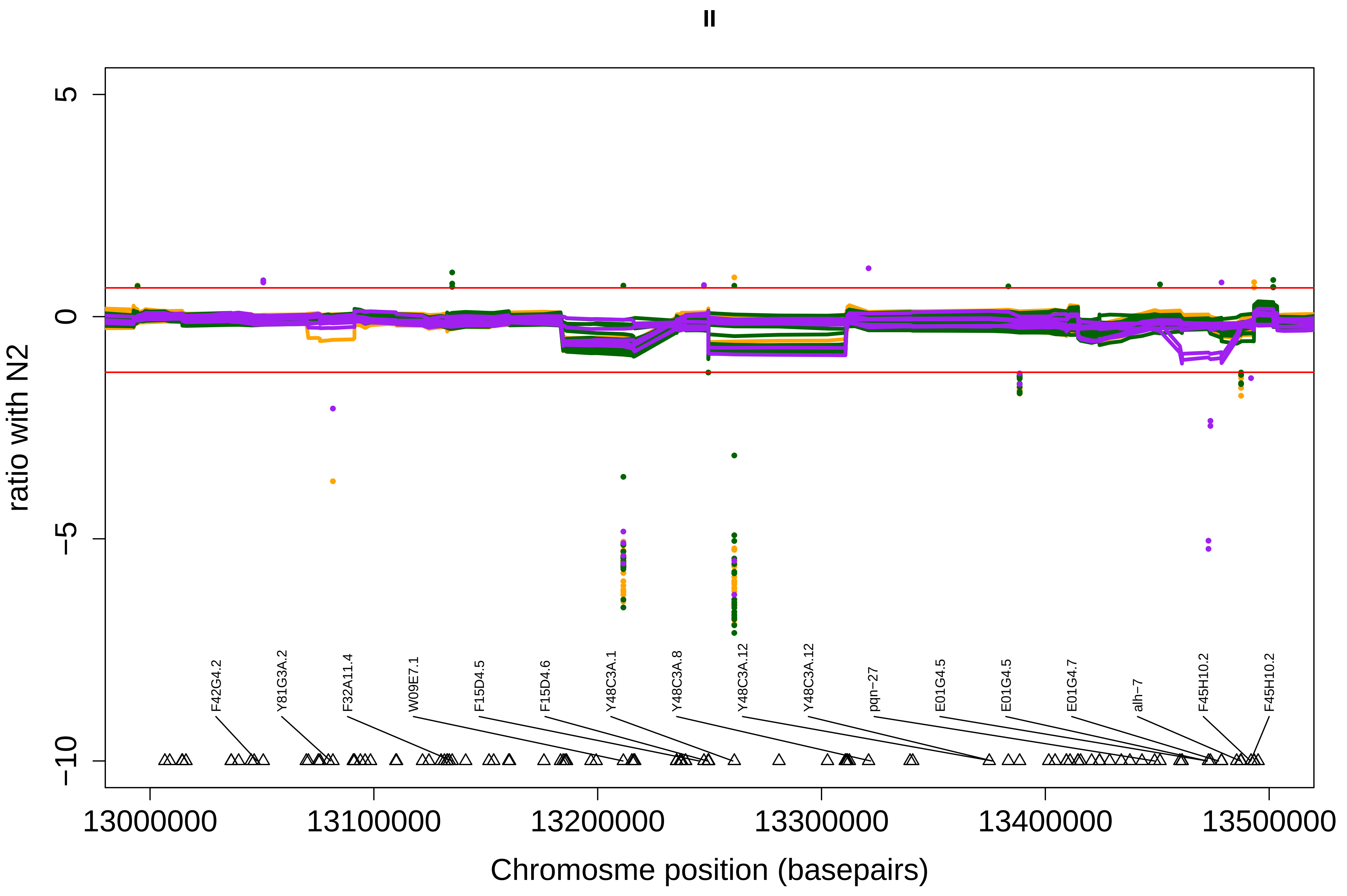

II
ratio with N2
5
0
−5
−10
F42G4.2
Y81G3A.2
F32A11.4
W09E7.1
F15D4.5
F15D4.6
Y48C3A.1
Y48C3A.8
Y48C3A.12
Y48C3A.12
pqn−27
E01G4.5
E01G4.5
E01G4.7
alh−7
F45H10.2
F45H10.2
13000000
13100000
13200000
13300000
13400000
13500000
Chromosme position (basepairs)

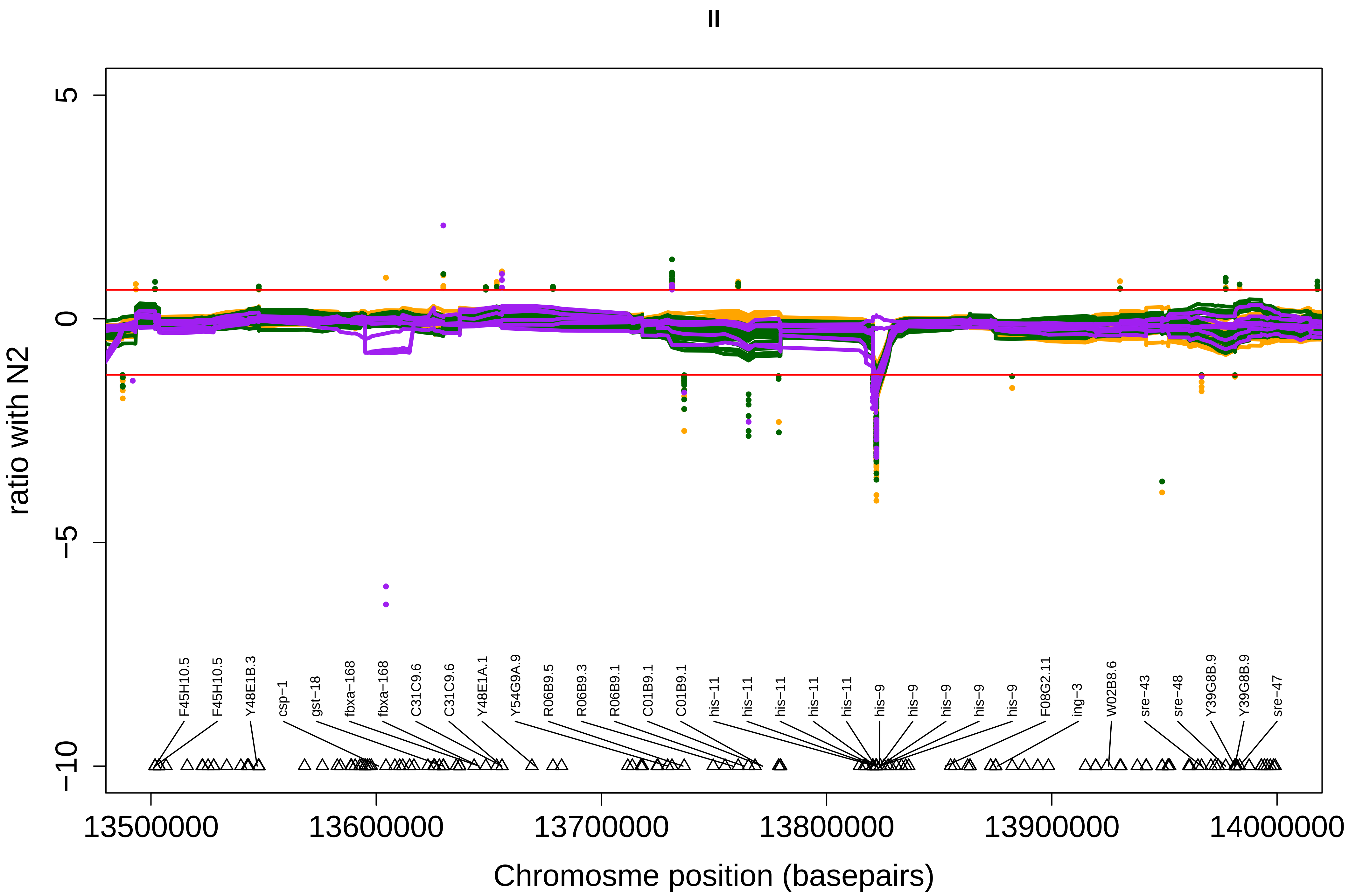
II
ratio with N2
5
0
−5
−10
F45H10.5
F45H10.5
Y48E1B.3
csp−1
gst−18
fbxa−168
fbxa−168
C31C9.6
C31C9.6
Y48E1A.1
Y54G9A.9
R06B9.5
R06B9.3
R06B9.1
C01B9.1
C01B9.1
his−11
his−11
his−11
his−11
his−11
his−9
his−9
his−9
his−9
his−9
F08G2.11
ing−3
W02B8.6
sre−43
sre−48
Y39G8B.9
Y39G8B.9
sre−47
13500000
13600000
13700000
13800000
13900000
14000000
Chromosme position (basepairs)

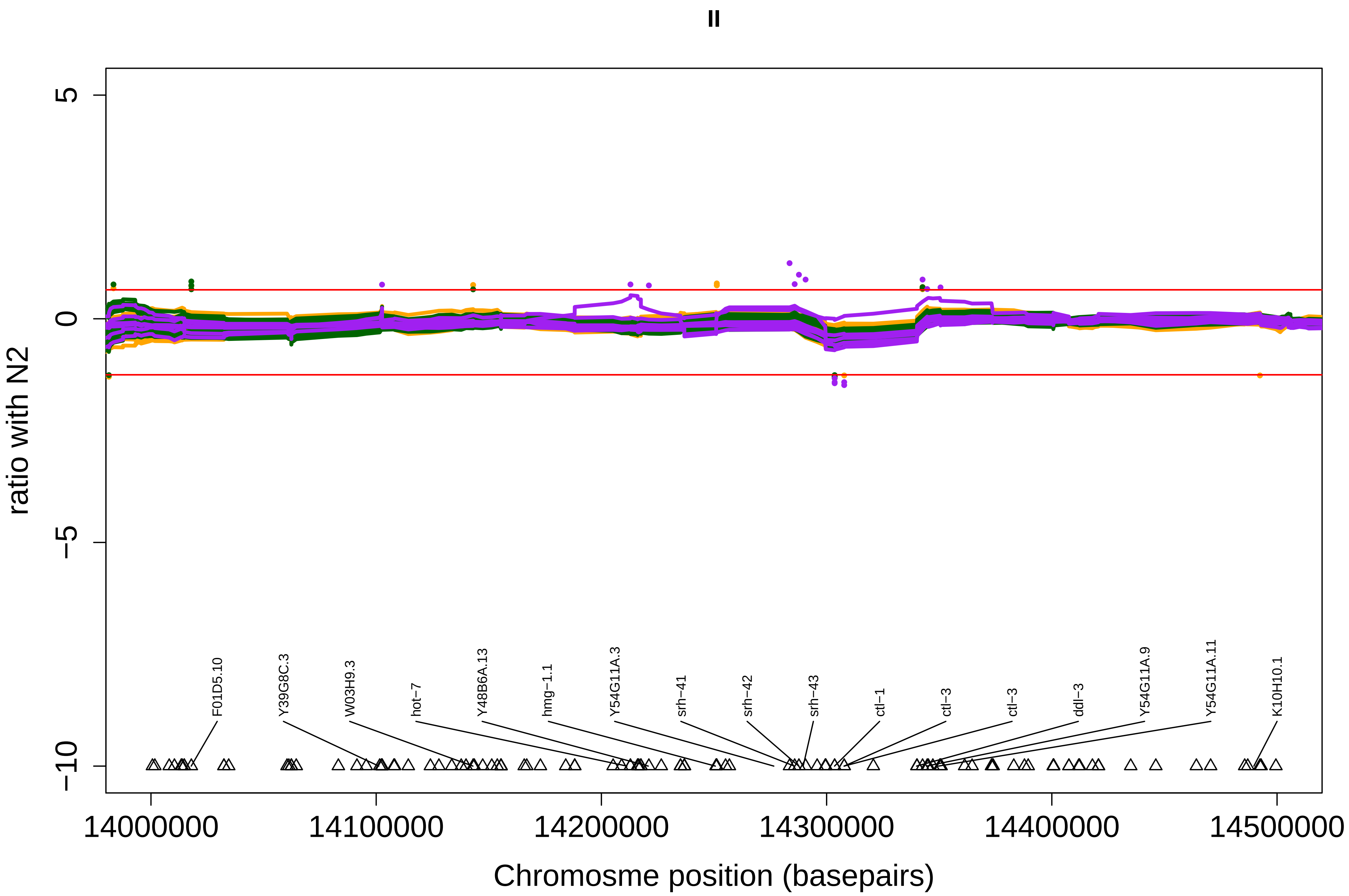

II
ratio with N2
5
0
−5
−10
F01D5.10
Y39G8C.3
W03H9.3
hot−7
Y48B6A.13
hmg−1.1
Y54G11A.3
srh−41
srh−42
srh−43
ctl−1
ctl−3
ctl−3
ddl−3
Y54G11A.9
Y54G11A.11
K10H10.1
14000000
14100000
14200000
14300000
14400000
14500000
Chromosme position (basepairs)

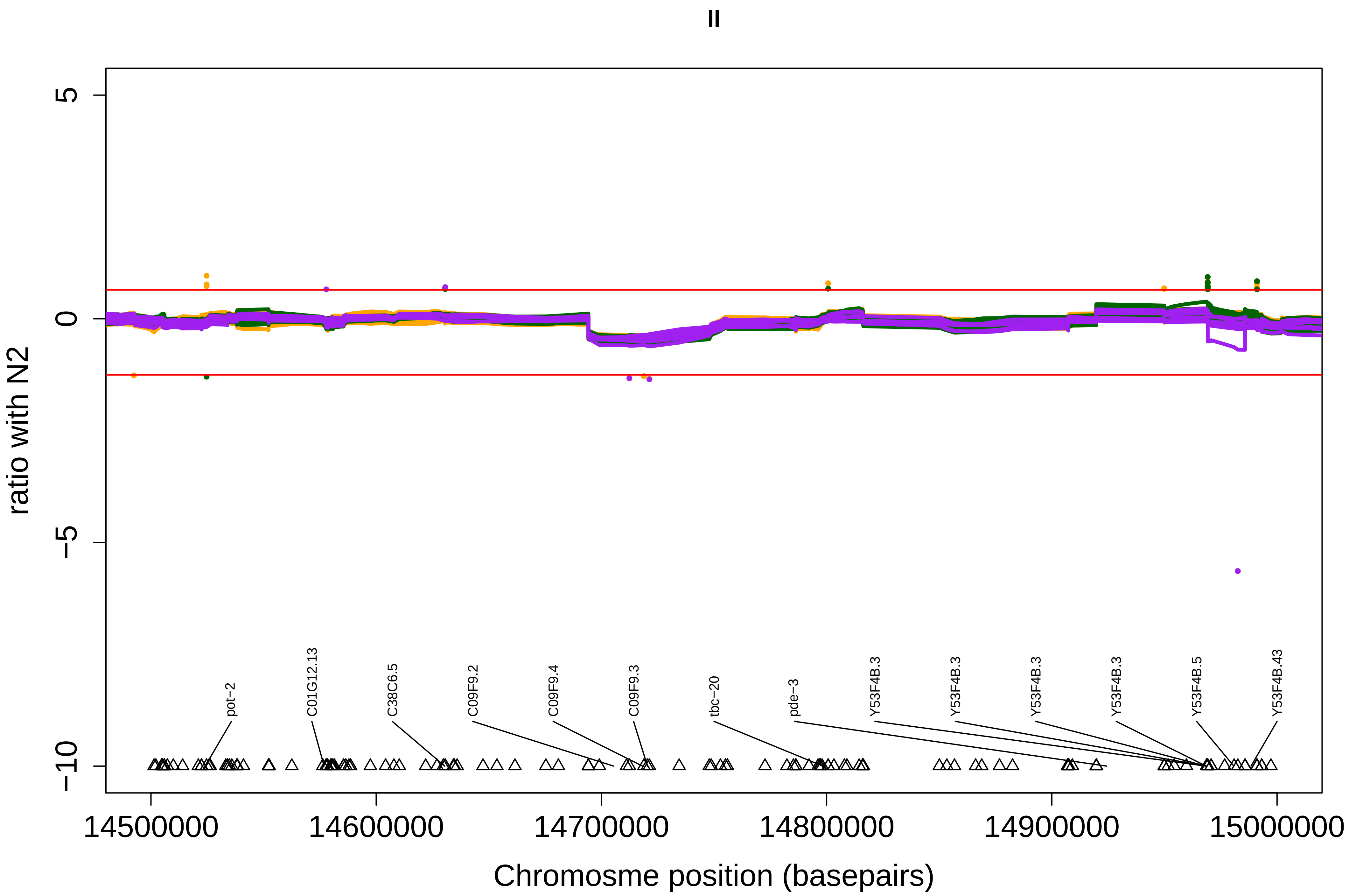

II
ratio with N2
5
0
−5
−10
pot−2
C01G12.13
C38C6.5
C09F9.2
C09F9.4
C09F9.3
tbc−20
pde−3
Y53F4B.3
Y53F4B.3
Y53F4B.3
Y53F4B.3
Y53F4B.5
Y53F4B.43
14500000
14600000
14700000
14800000
14900000
15000000
Chromosme position (basepairs)

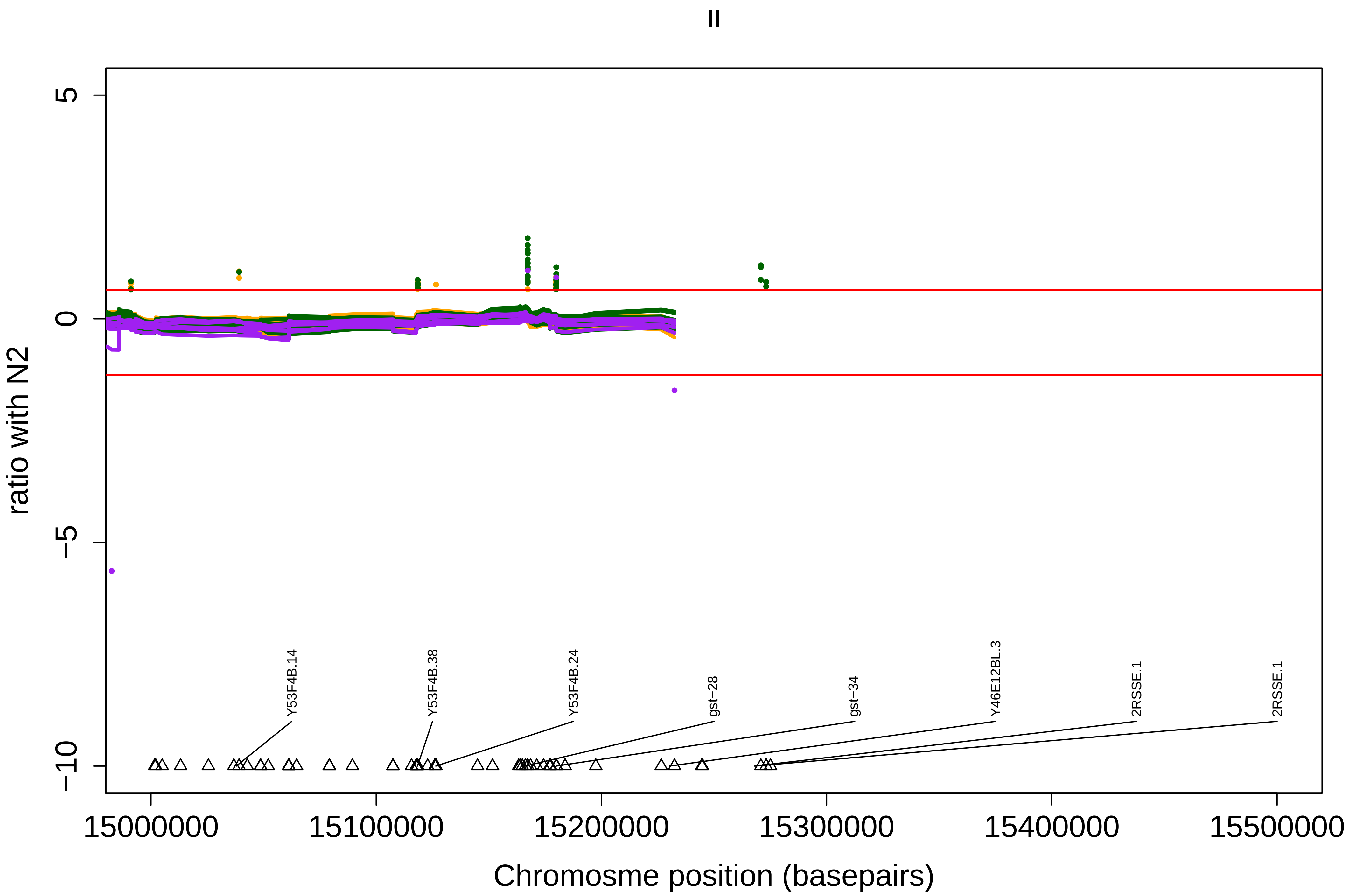
II
ratio with N2
5
0
−5
−10
Y53F4B.14
Y53F4B.38
Y53F4B.24
gst−28
gst−34
Y46E12BL.3
2RSSE.1
2RSSE.1
15000000
15100000
15200000
15300000
15400000
15500000
Chromosme position (basepairs)

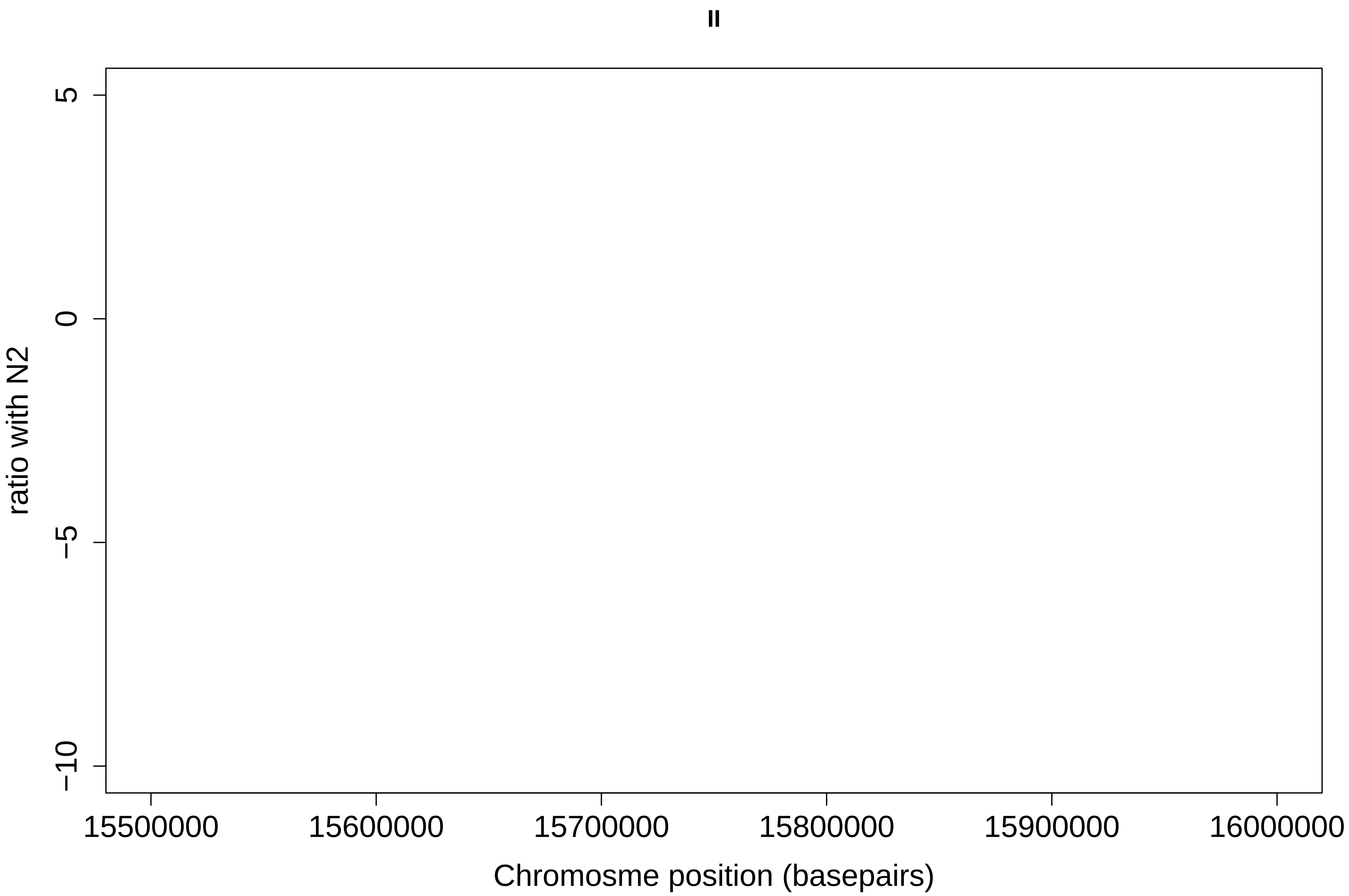
II
ratio with N2
5
0
−5
−10
15500000
15600000
15700000
15800000
15900000
16000000
Chromosme position (basepairs)

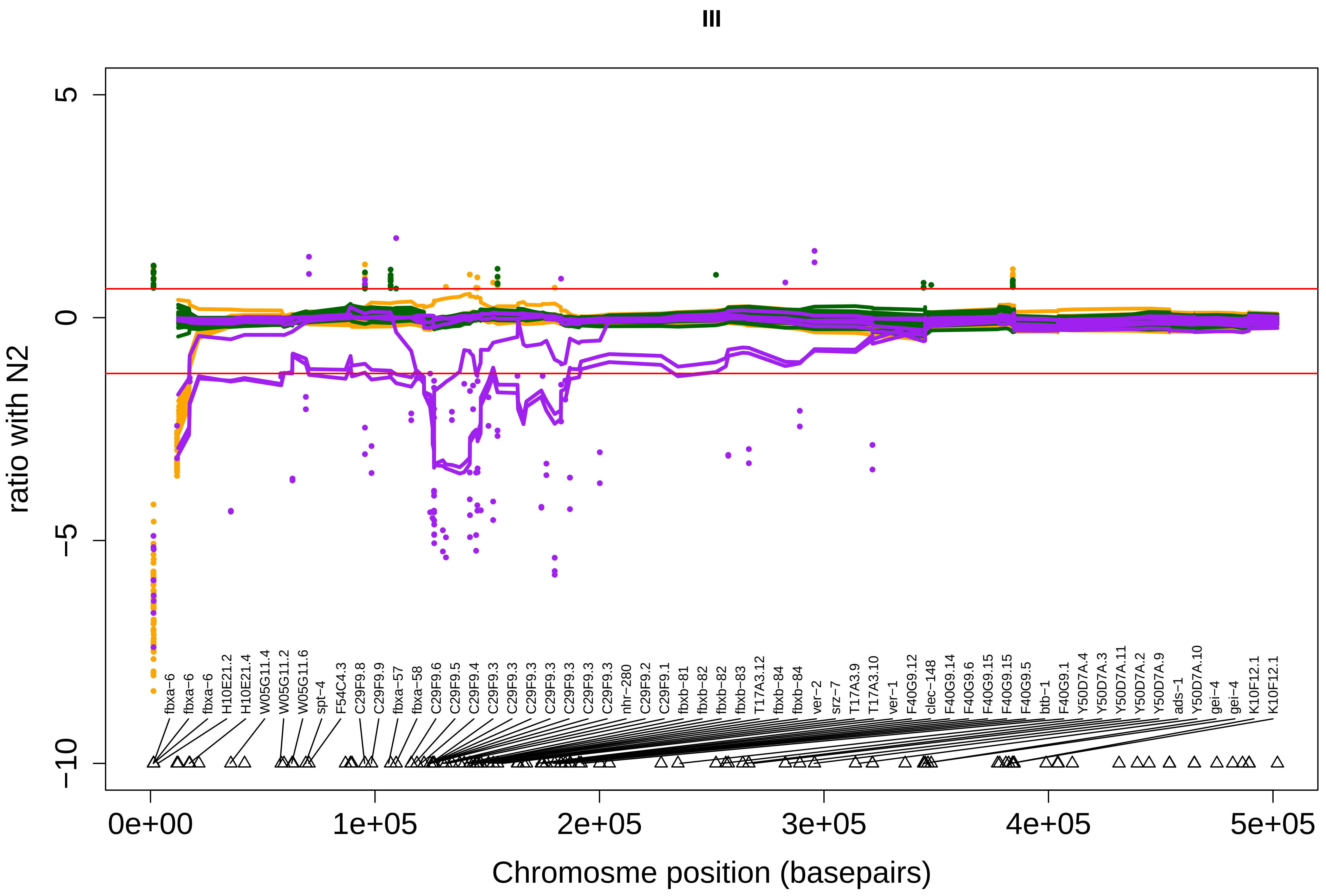

III
ratio with N2
5
0
−5
−10
0e+00
1e+05
2e+05
3e+05
4e+05
5e+05
Chromosme position (basepairs)
fbxa-6
fbxa-6
fbxa-6
H10E21.2
H10E21.4
W05G11.4
W05G11.2
W05G11.6
spt-4
F54C4.3
C29F9.8
C29F9.9
fbxa-57
fbxa-58
C29F9.6
C29F9.5
C29F9.4
C29F9.3
C29F9.3
C29F9.3
C29F9.3
C29F9.3
C29F9.3
C29F9.3
nhr-280
C29F9.2
C29F9.1
fbxb-81
fbxb-82
fbxb-82
fbxb-83
T17A3.12
fbxb-84
fbxb-84
ver-2
srz-7
T17A3.9
T17A3.10
ver-1
F40G9.12
clec-148
F40G9.14
F40G9.6
F40G9.15
F40G9.15
F40G9.5
btb-1
F40G9.1
Y50D7A.4
Y50D7A.3
Y50D7A.11
Y50D7A.2
Y50D7A.9
ads-1
Y50D7A.10
gei-4
gei-4
K10F12.1
K10F12.1

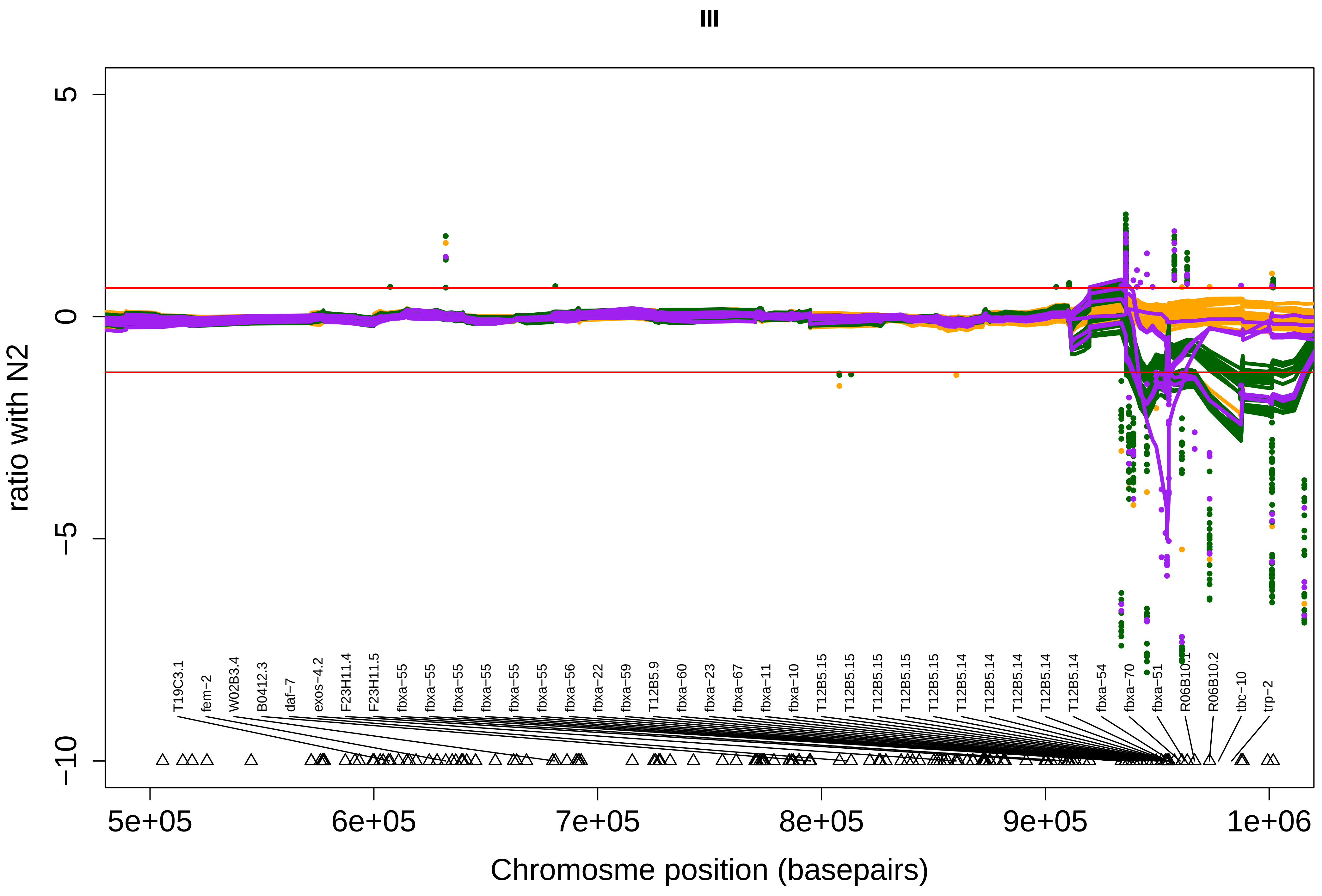
III
ratio with N2
5
0
−5
−10
5e+05
6e+05
7e+05
8e+05
9e+05
1e+06
Chromosme position (basepairs)
T19C3.1
fem−2
W02B3.4
B0412.3
daf−7
exos−4.2
F23H11.4
F23H11.5
fbxa−55
fbxa−55
fbxa−55
fbxa−55
fbxa−55
fbxa−55
fbxa−56
fbxa−22
fbxa−59
T12B5.9
fbxa−60
fbxa−23
fbxa−67
fbxa−11
fbxa−10
T12B5.15
T12B5.15
T12B5.15
T12B5.15
T12B5.15
T12B5.14
T12B5.14
T12B5.14
T12B5.14
T12B5.14
fbxa−54
fbxa−70
fbxa−51
R06B10.1
R06B10.2
tbc−10
trp−2

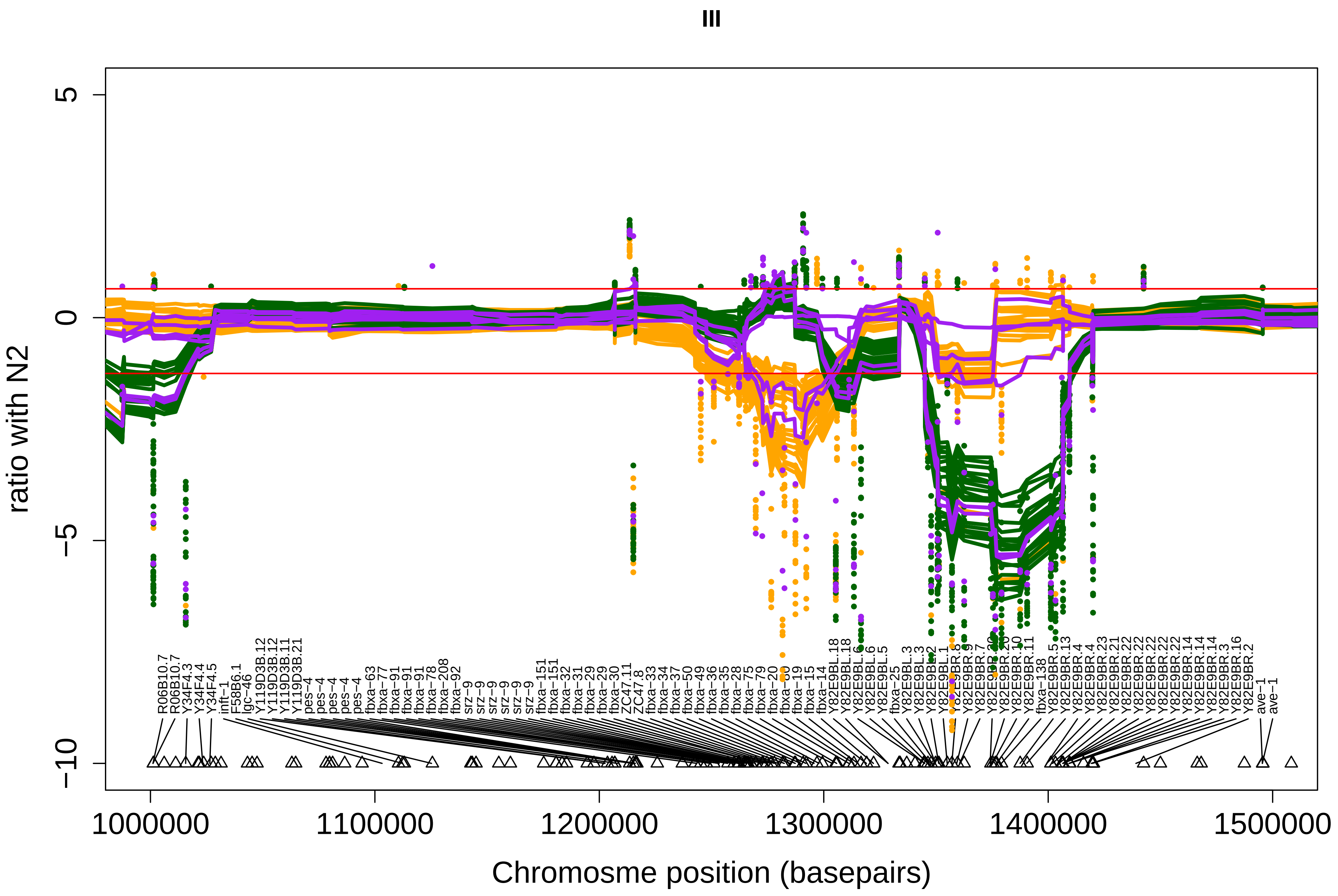
III
ratio with N2
5
0
−5
−10
1000000
1100000
1200000
1300000
1400000
1500000
Chromosme position (basepairs)

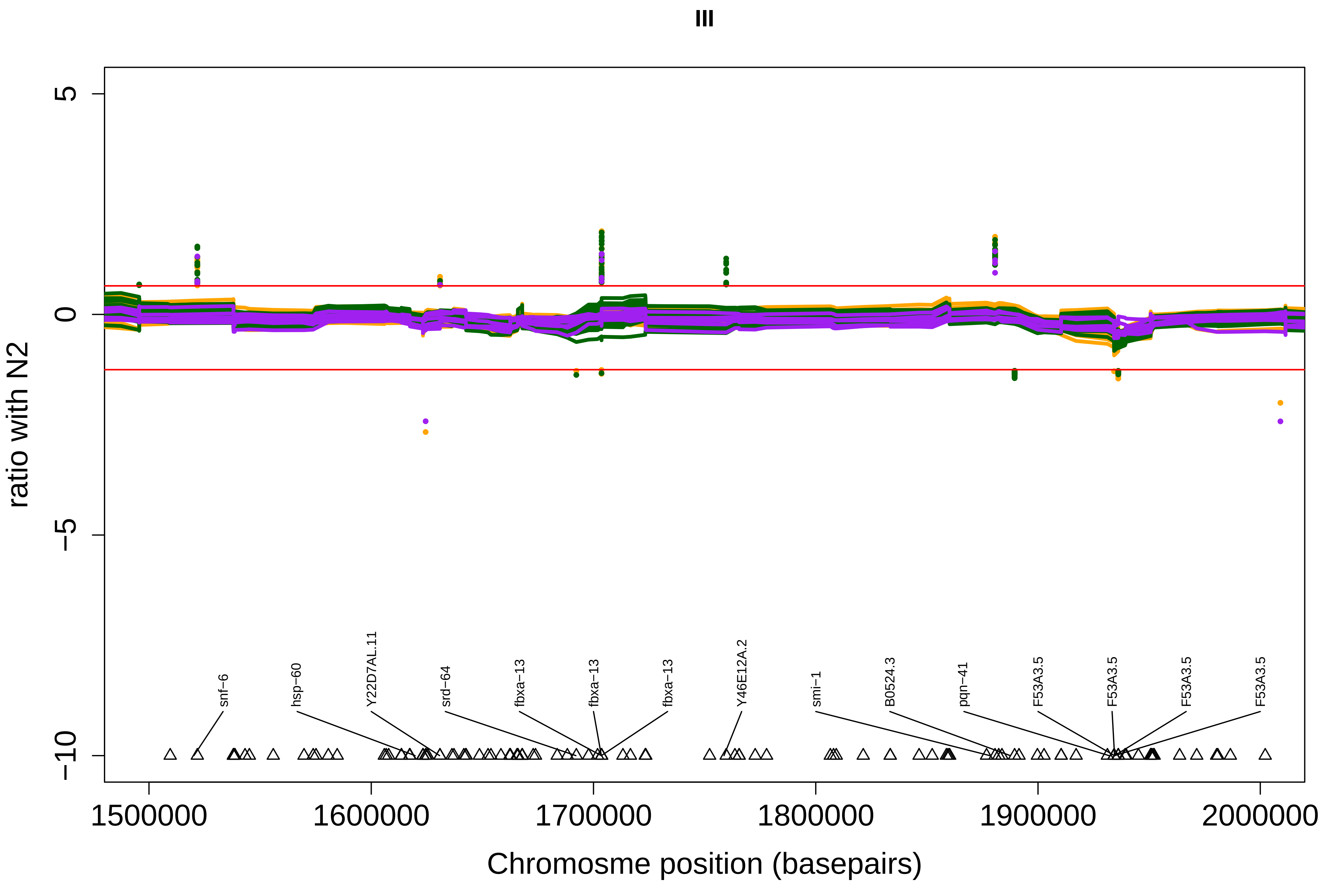
III
ratio with N2
5
0
−5
−10
snf−6
hsp−60
Y22D7AL.11
srd−64
fbxa−13
fbxa−13
fbxa−13
Y46E12A.2
smi−1
B0524.3
pqn−41
F53A3.5
F53A3.5
F53A3.5
F53A3.5
1500000
1600000
1700000
1800000
1900000
2000000
Chromosme position (basepairs)

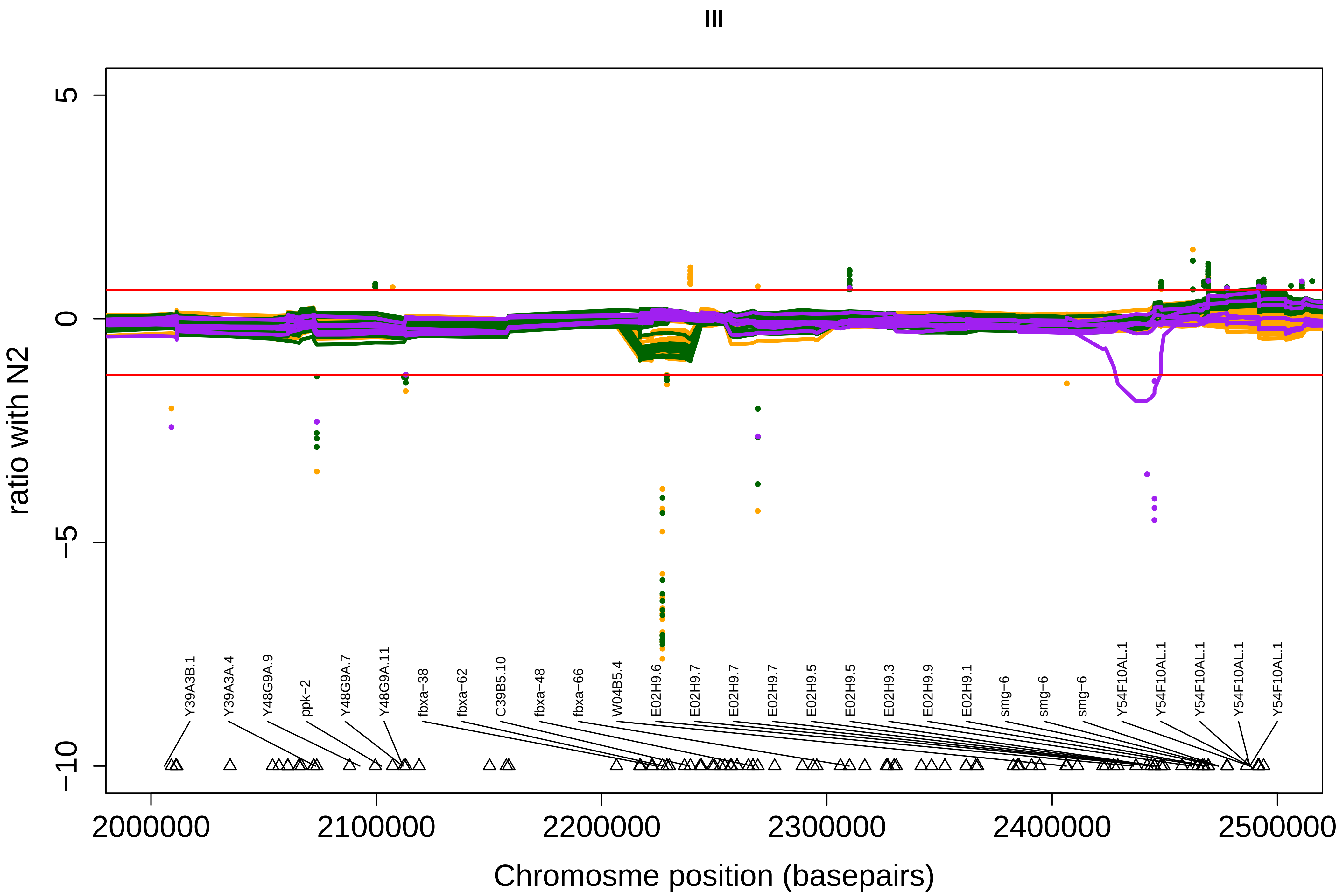
III
ratio with N2
5
0
−5
−10
Y39A3B.1
Y39A3A.4
Y48G9A.9
ppk−2
Y48G9A.7
Y48G9A.11
fbxa−38
fbxa−62
C39B5.10
fbxa−48
fbxa−66
W04B5.4
E02H9.6
E02H9.7
E02H9.7
E02H9.7
E02H9.5
E02H9.5
E02H9.3
E02H9.9
E02H9.1
smg−6
smg−6
smg−6
Y54F10AL.1
Y54F10AL.1
Y54F10AL.1
Y54F10AL.1
Y54F10AL.1
2000000
2100000
2200000
2300000
2400000
2500000
Chromosme position (basepairs)

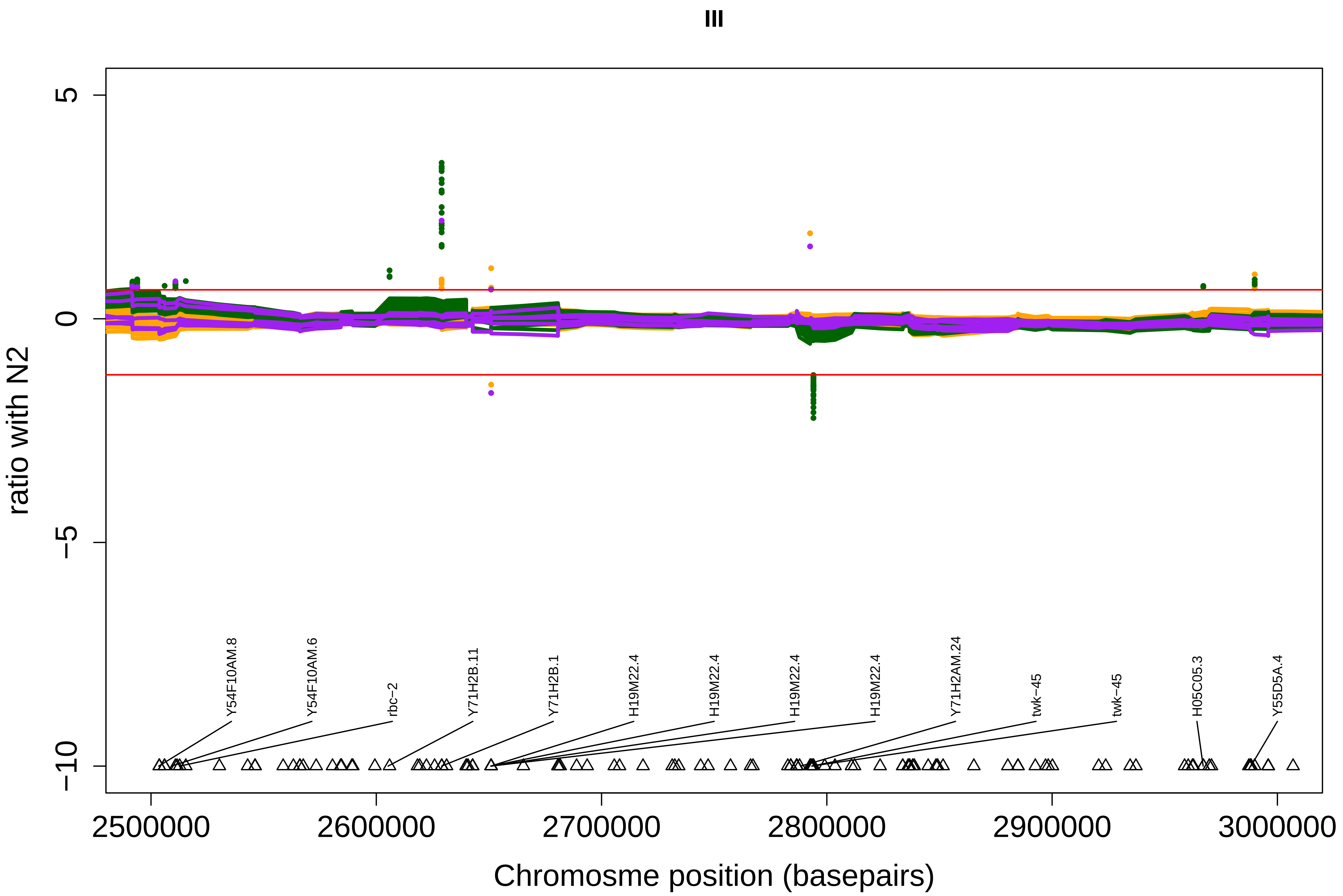
III
ratio with N2
5
0
−5
−10
Chromosme position (basepairs)
2500000
2600000
2700000
2800000
2900000
3000000
Y54F10AM.8
Y54F10AM.6
rbc−2
Y71H2B.11
Y71H2B.1
H19M22.4
H19M22.4
H19M22.4
H19M22.4
Y71H2AM.24
twk−45
twk−45
H05C05.3
Y55D5A.4

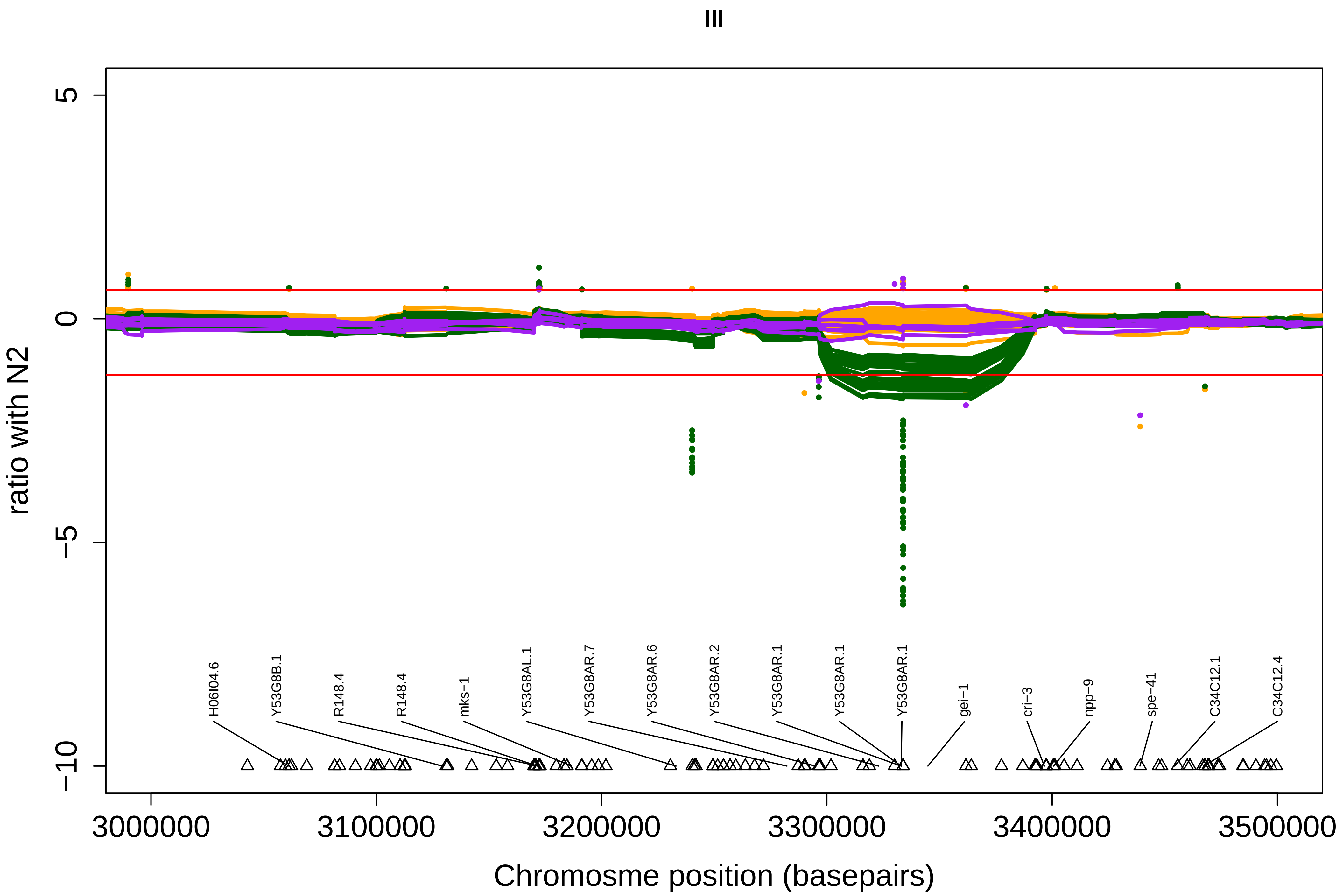

III
ratio with N2
5
0
−5
−10
H06I04.6
Y53G8B.1
R148.4
R148.4
mks−1
Y53G8AL.1
Y53G8AR.7
Y53G8AR.6
Y53G8AR.2
Y53G8AR.1
Y53G8AR.1
Y53G8AR.1
gei−1
cri−3
npp−9
spe−41
C34C12.1
C34C12.4
3000000
3100000
3200000
3300000
3400000
3500000
Chromosme position (basepairs)

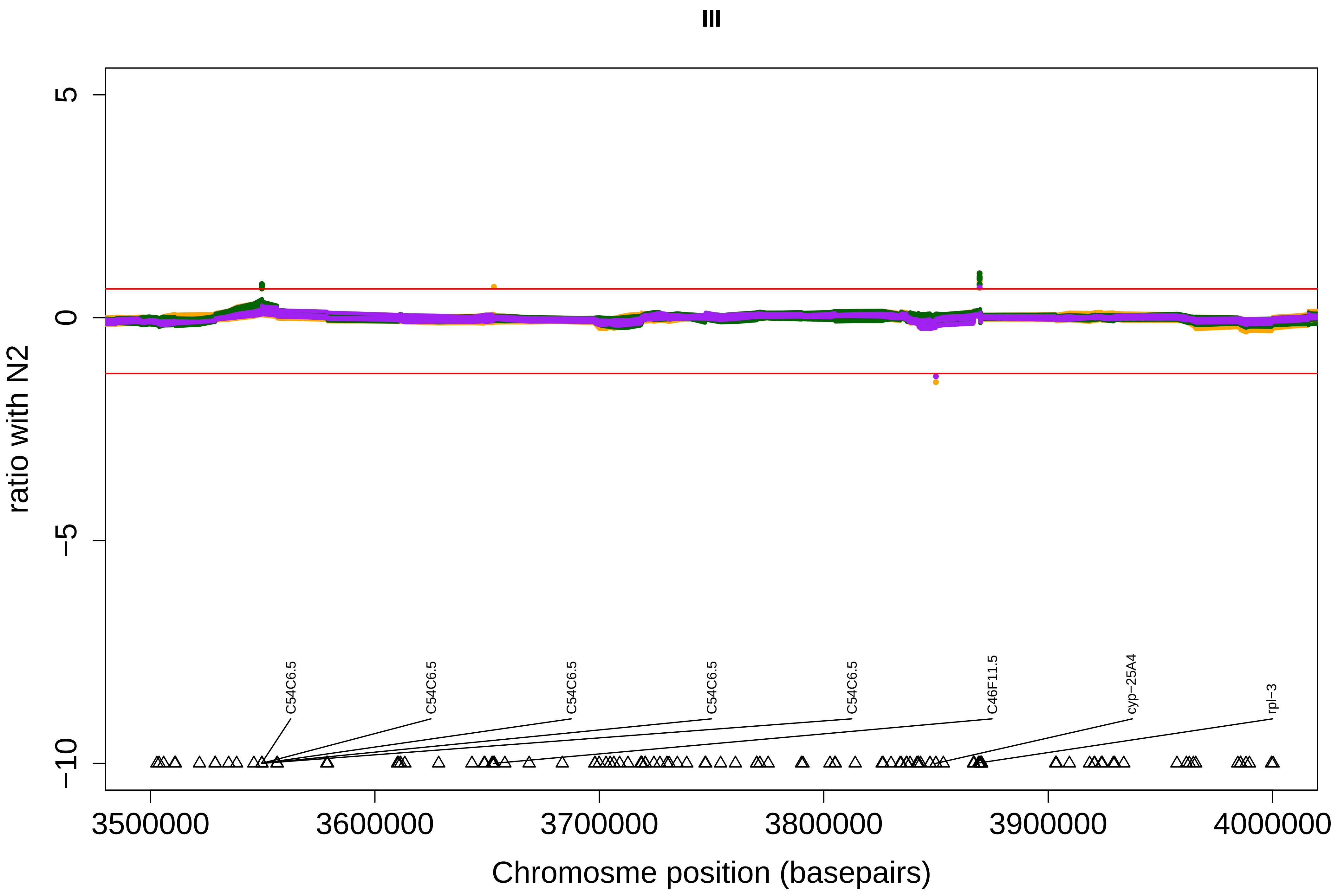

III
ratio with N2
5
0
−5
−10
C54C6.5
C54C6.5
C54C6.5
C54C6.5
C54C6.5
C46F11.5
cyp−25A4
rpl−3
3500000
3600000
3700000
3800000
3900000
4000000
Chromosme position (basepairs)

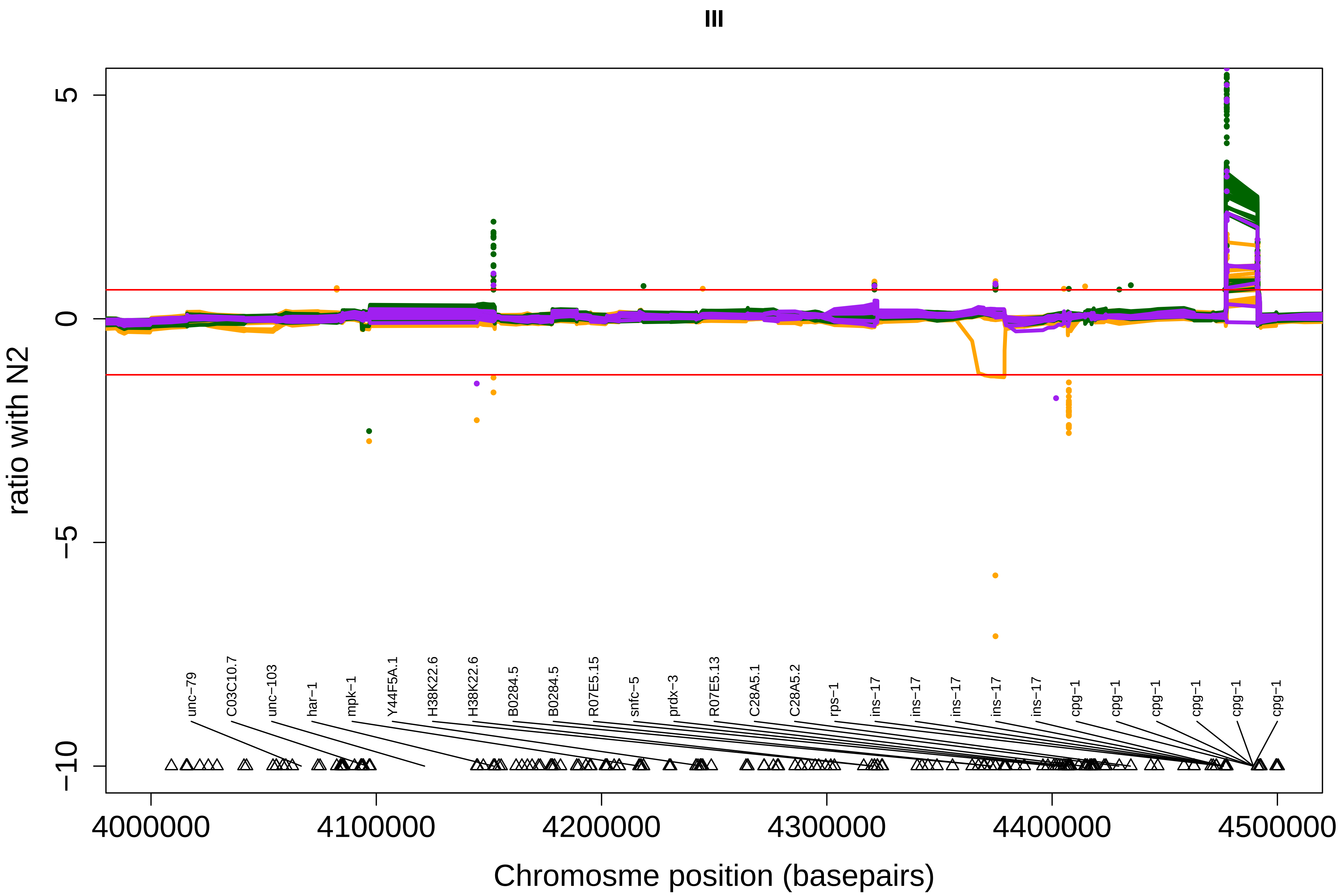
III
ratio with N2
Chromosme position (basepairs)
5
0
−5
−10
4000000
4100000
4200000
4300000
4400000
4500000
unc−79
C03C10.7
unc−103
har−1
mpk−1
Y44F5A.1
H38K22.6
H38K22.6
B0284.5
B0284.5
R07E5.15
snfc−5
prdx−3
R07E5.13
C28A5.1
C28A5.2
rps−1
ins−17
ins−17
ins−17
ins−17
ins−17
cpg−1
cpg−1
cpg−1
cpg−1
cpg−1
cpg−1

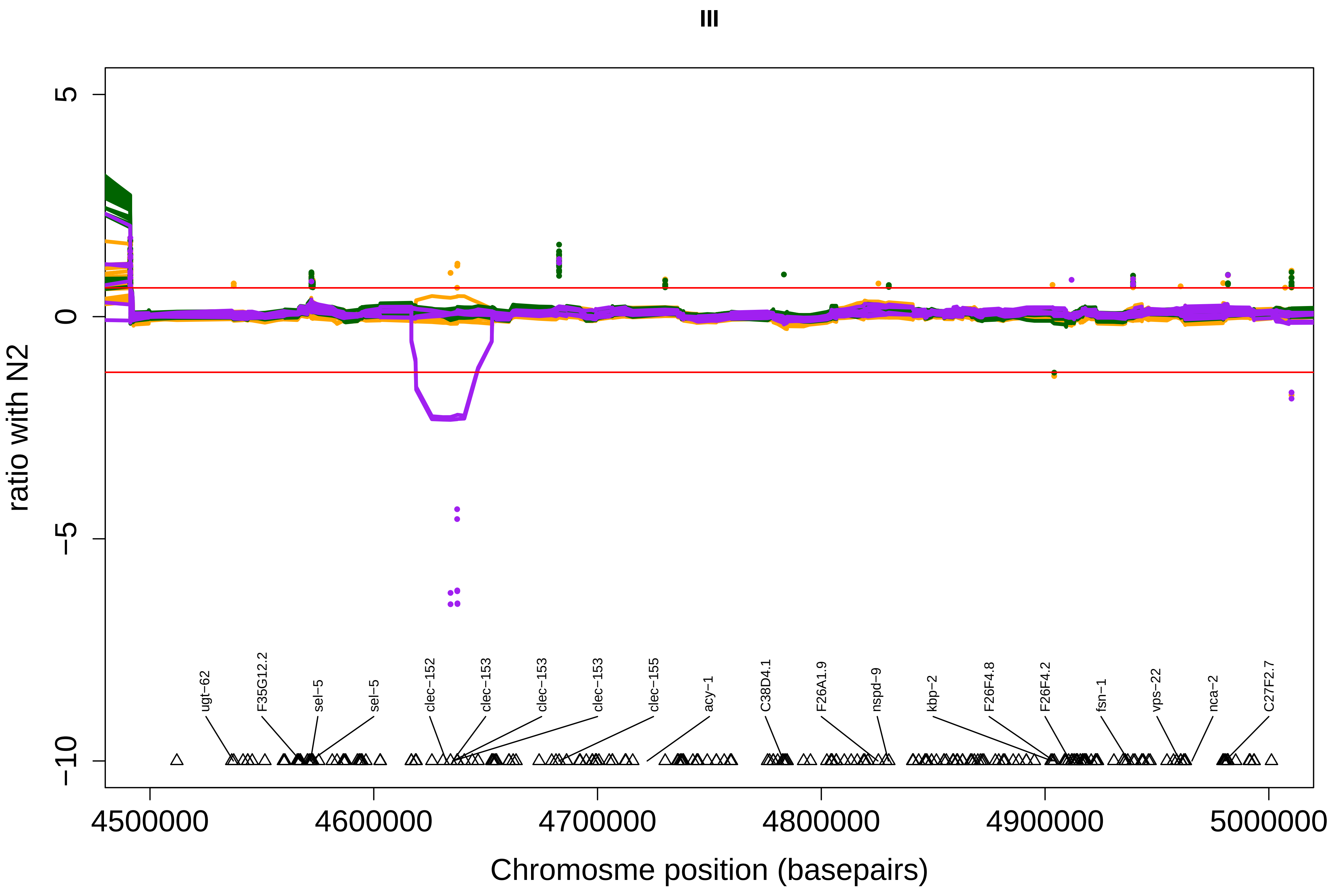

III
ratio with N2
5
0
−5
−10
ugt−62
F35G12.2
sel−5
sel−5
clec−152
clec−153
clec−153
clec−153
clec−155
acy−1
C38D4.1
F26A1.9
nspd−9
kbp−2
F26F4.8
F26F4.2
fsn−1
vps−22
nca−2
C27F2.7
4500000
4600000
4700000
4800000
4900000
5000000
Chromosme position (basepairs)

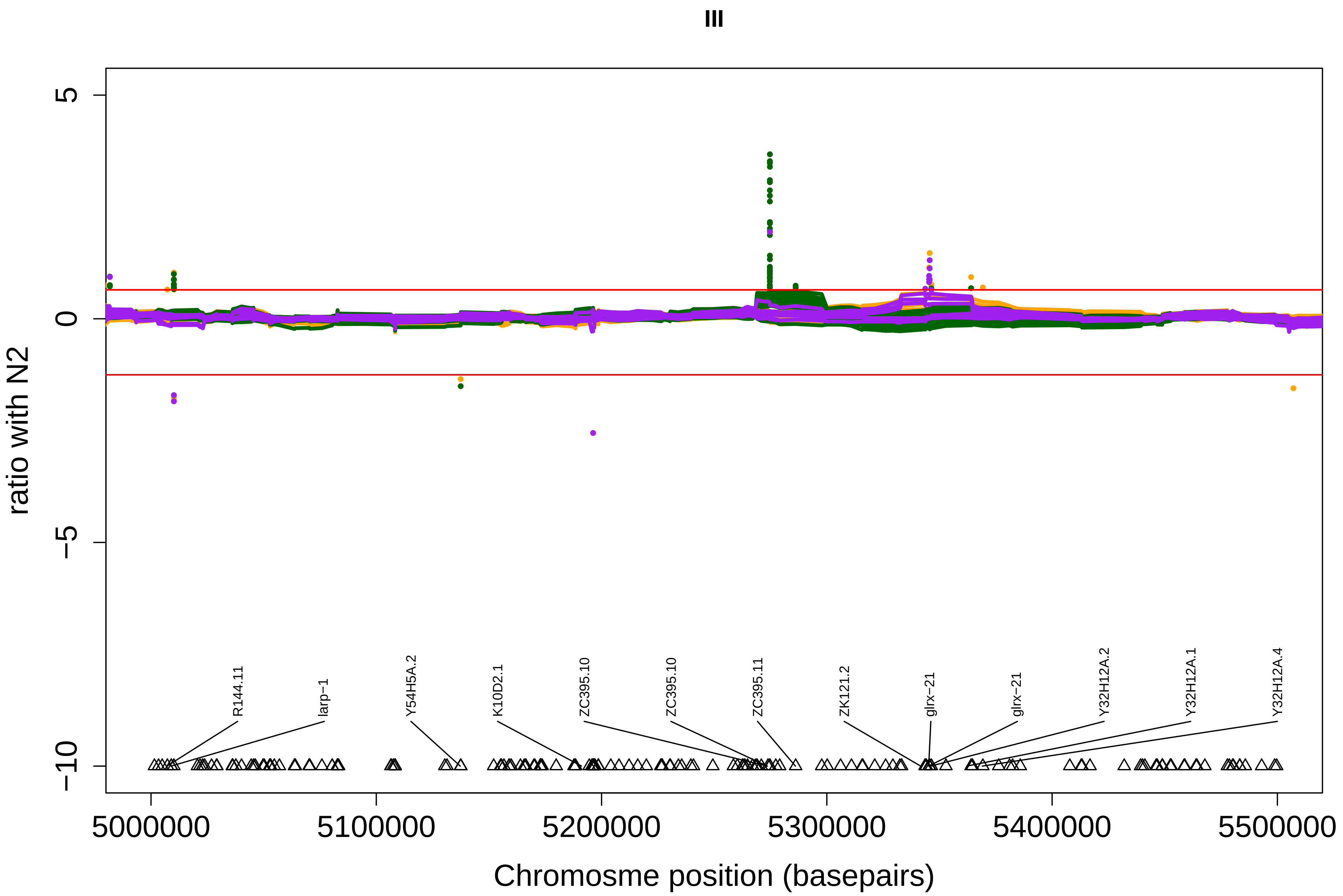

III
ratio with N2
5
0
−5
−10
5000000
5100000
5200000
5300000
5400000
5500000
Chromosme position (basepairs)
R144.11
larp−1
Y54H5A.2
K10D2.1
ZC395.10
ZC395.10
ZC395.11
ZK121.2
glrx−21
glrx−21
Y32H12A.2
Y32H12A.1
Y32H12A.4

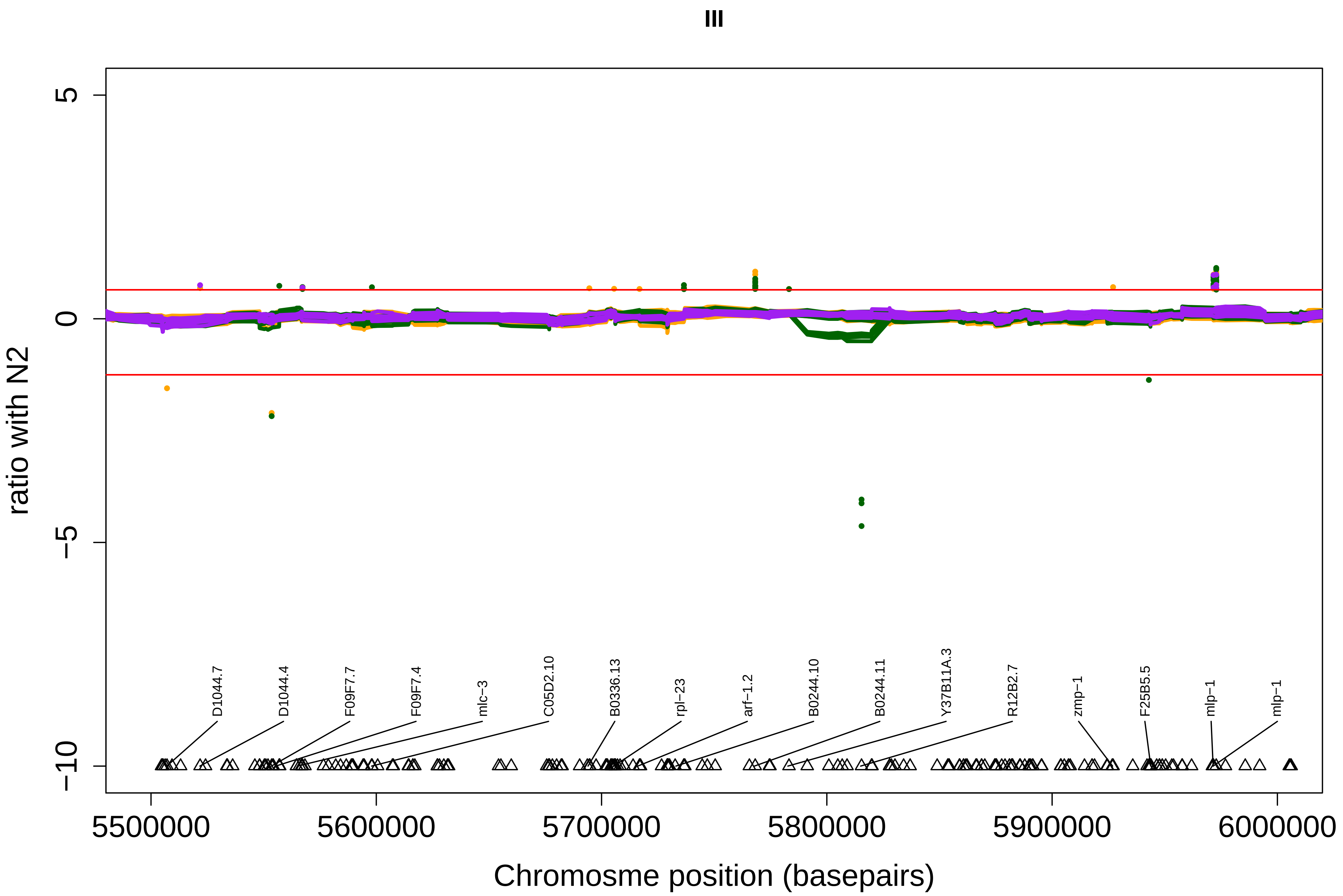
III
ratio with N2
5
0
−5
−10
D1044.7
D1044.4
F09F7.7
F09F7.4
mlc−3
C05D2.10
B0336.13
rpl−23
arf−1.2
B0244.10
B0244.11
Y37B11A.3
R12B2.7
zmp−1
F25B5.5
mlp−1
mlp−1
5500000
5600000
5700000
5800000
5900000
6000000
Chromosme position (basepairs)

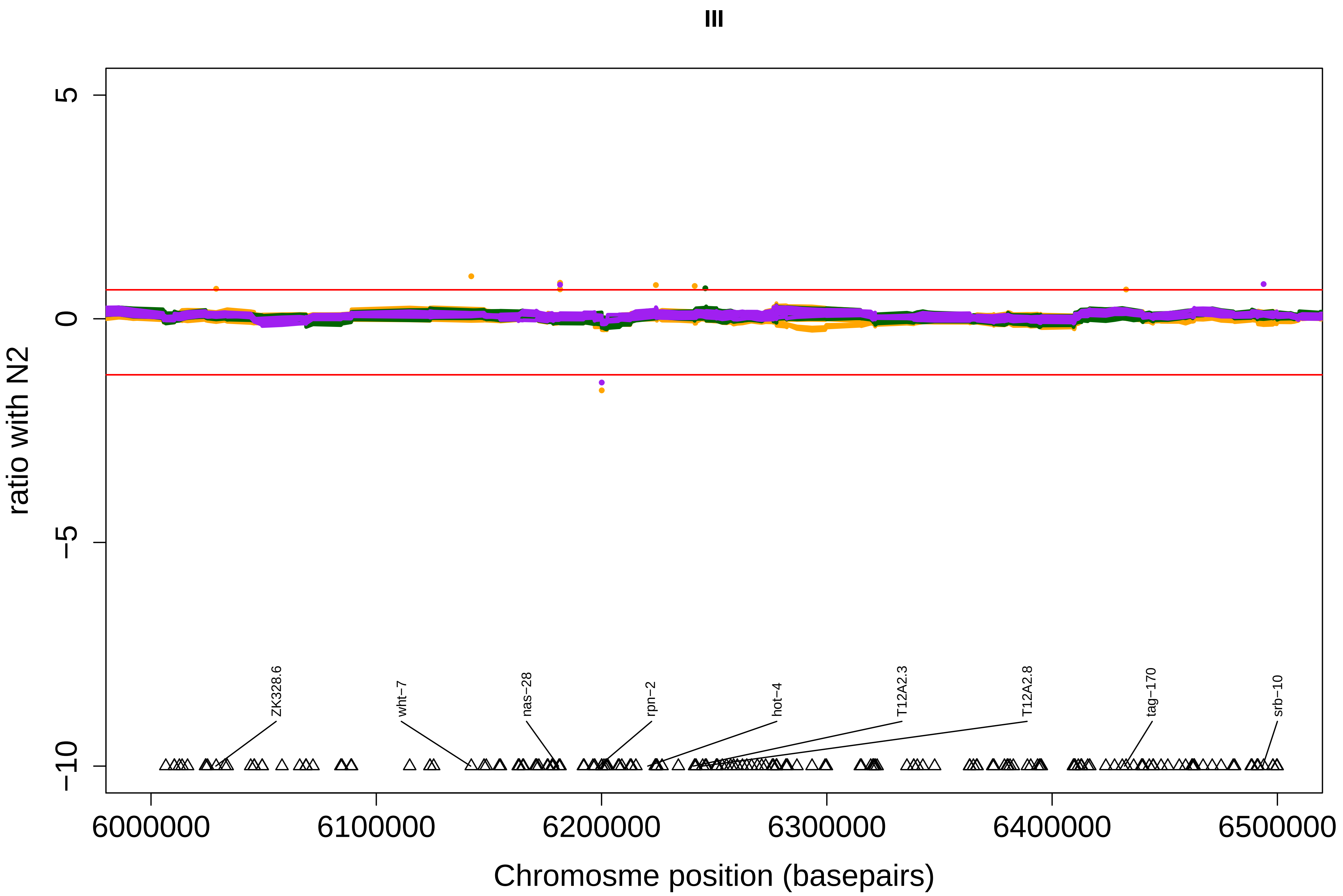
III
ratio with N2
5
0
−5
−10
6000000
6100000
6200000
6300000
6400000
6500000
Chromosme position (basepairs)
ZK328.6
wht−7
nas−28
rpn−2
hot−4
T12A2.3
T12A2.8
tag−170
srb−10

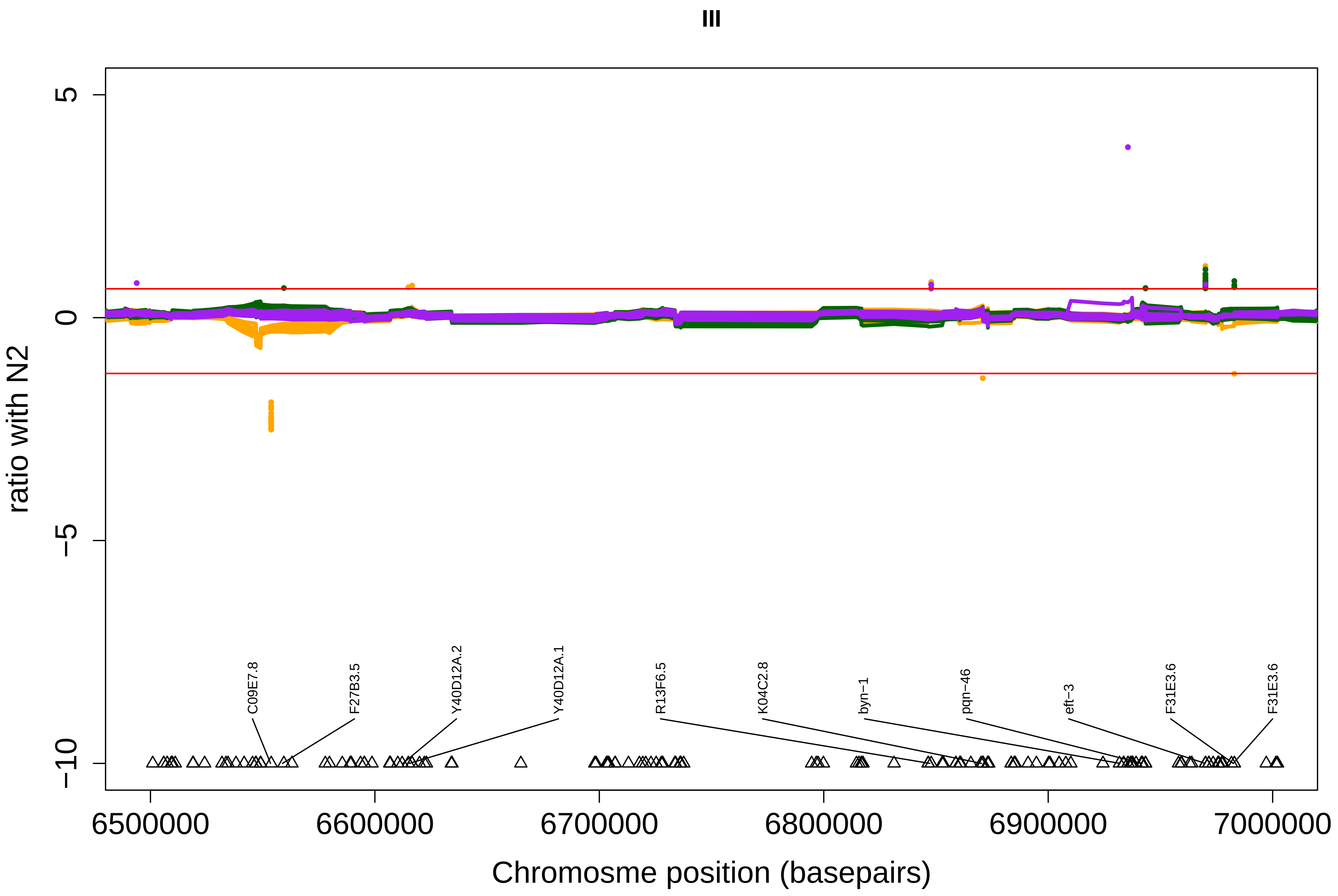
III
ratio with N2
5
0
−5
−10
C09E7.8
F27B3.5
Y40D12A.2
Y40D12A.1
R13F6.5
K04C2.8
byn−1
pqn−46
eft−3
F31E3.6
F31E3.6
6500000
6600000
6700000
6800000
6900000
7000000
Chromosme position (basepairs)

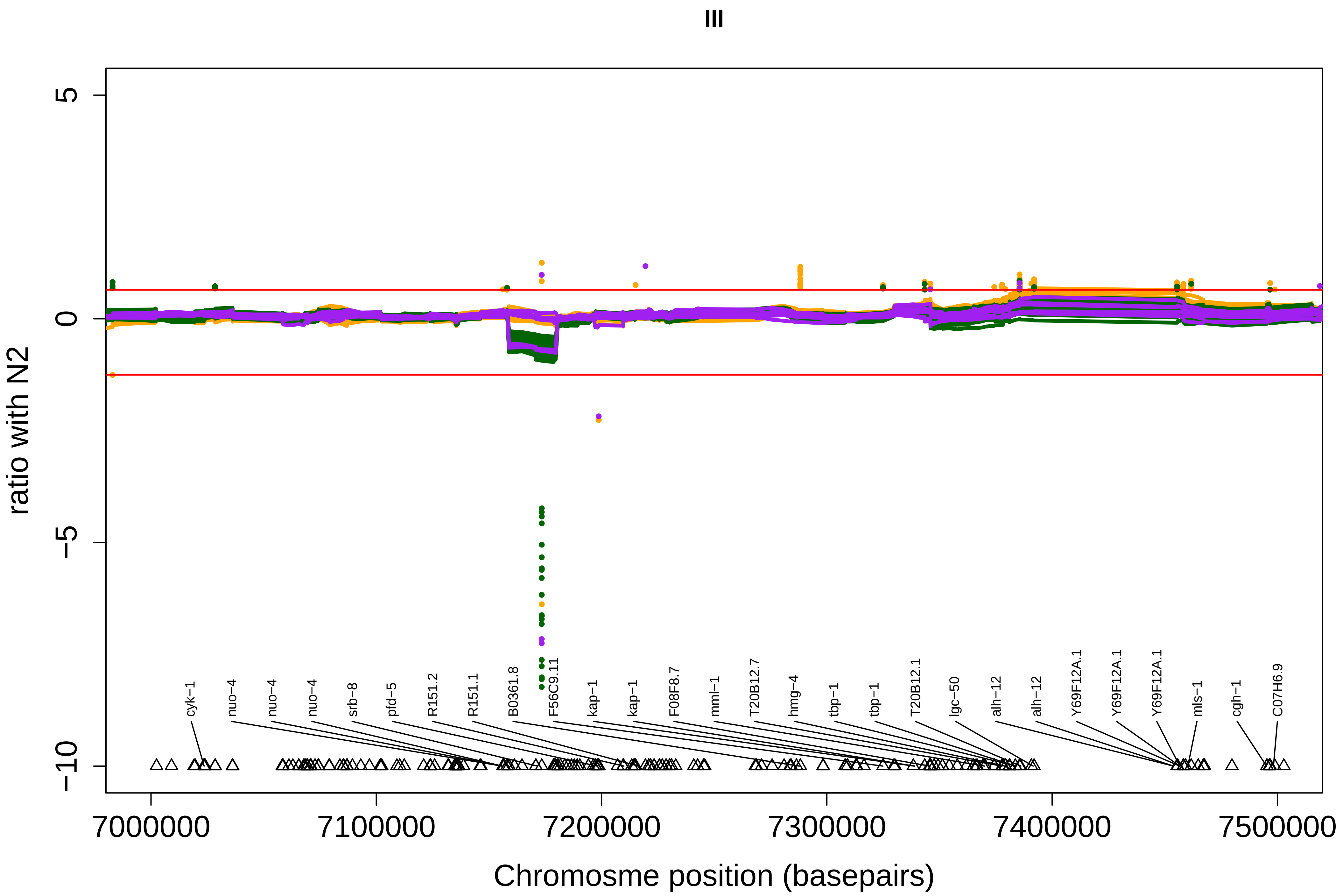
III
ratio with N2
5
0
−5
−10
7000000
7100000
7200000
7300000
7400000
7500000
Chromosme position (basepairs)
cyk−1
nuo−4
nuo−4
nuo−4
srb−8
pfd−5
R151.2
R151.1
B0361.8
F56C9.11
kap−1
kap−1
F08F8.7
mml−1
T20B12.7
hmg−4
tbp−1
tbp−1
T20B12.1
lgc−50
alh−12
alh−12
Y69F12A.1
Y69F12A.1
Y69F12A.1
mls−1
cgh−1
C07H6.9

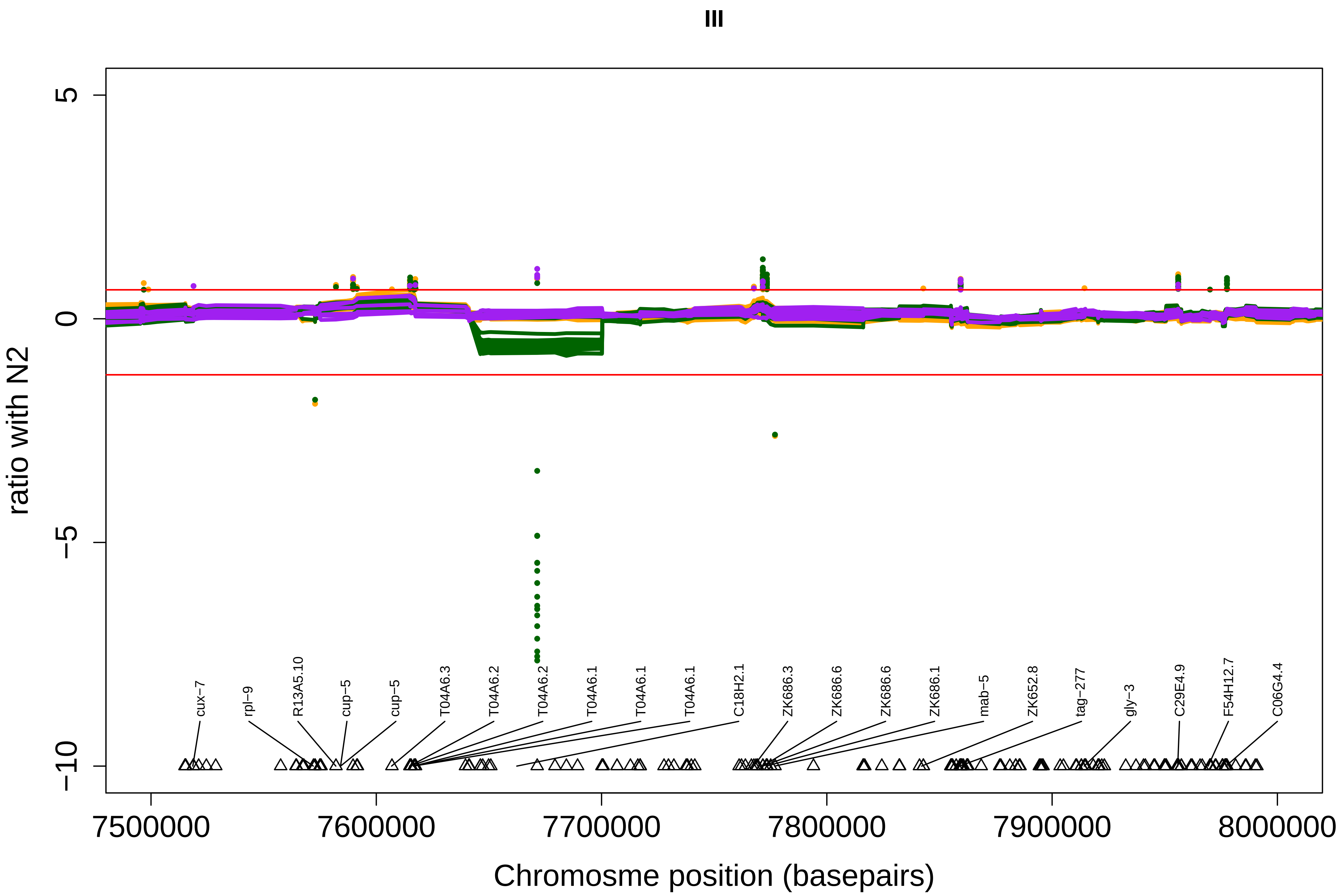

III
ratio with N2
5
0
−5
−10
cux−7
rpl−9
R13A5.10
cup−5
cup−5
T04A6.3
T04A6.2
T04A6.2
T04A6.1
T04A6.1
T04A6.1
C18H2.1
ZK686.3
ZK686.6
ZK686.6
ZK686.1
mab−5
ZK652.8
tag−277
gly−3
C29E4.9
F54H12.7
C06G4.4
7500000
7600000
7700000
7800000
7900000
8000000
Chromosme position (basepairs)

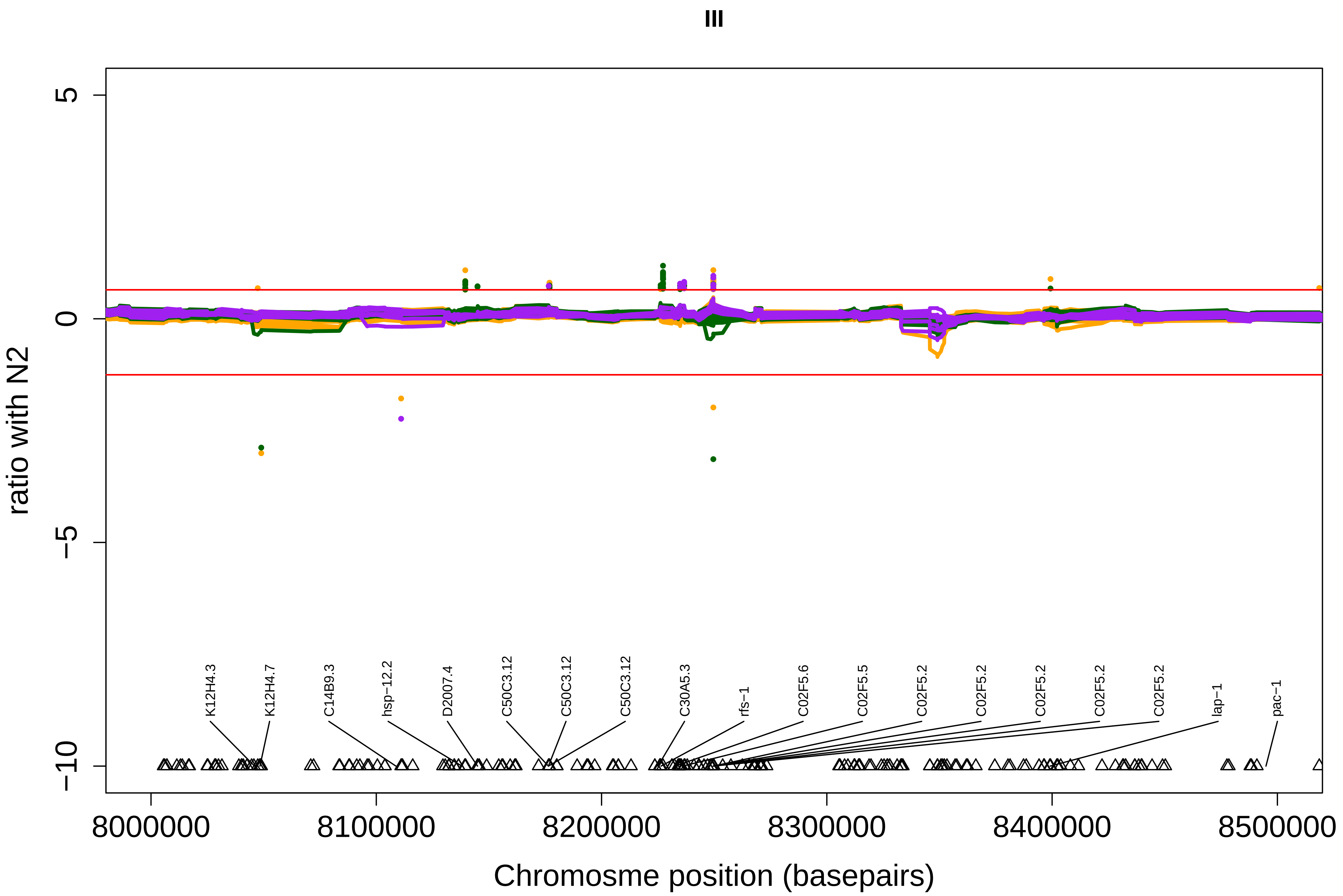

III
ratio with N2
5
0
−5
−10
K12H4.3
K12H4.7
C14B9.3
hsp−12.2
D2007.4
C50C3.12
C50C3.12
C50C3.12
C30A5.3
rfs−1
C02F5.6
C02F5.5
C02F5.2
C02F5.2
C02F5.2
C02F5.2
C02F5.2
lap−1
pac−1
8000000
8100000
8200000
8300000
8400000
8500000
Chromosme position (basepairs)

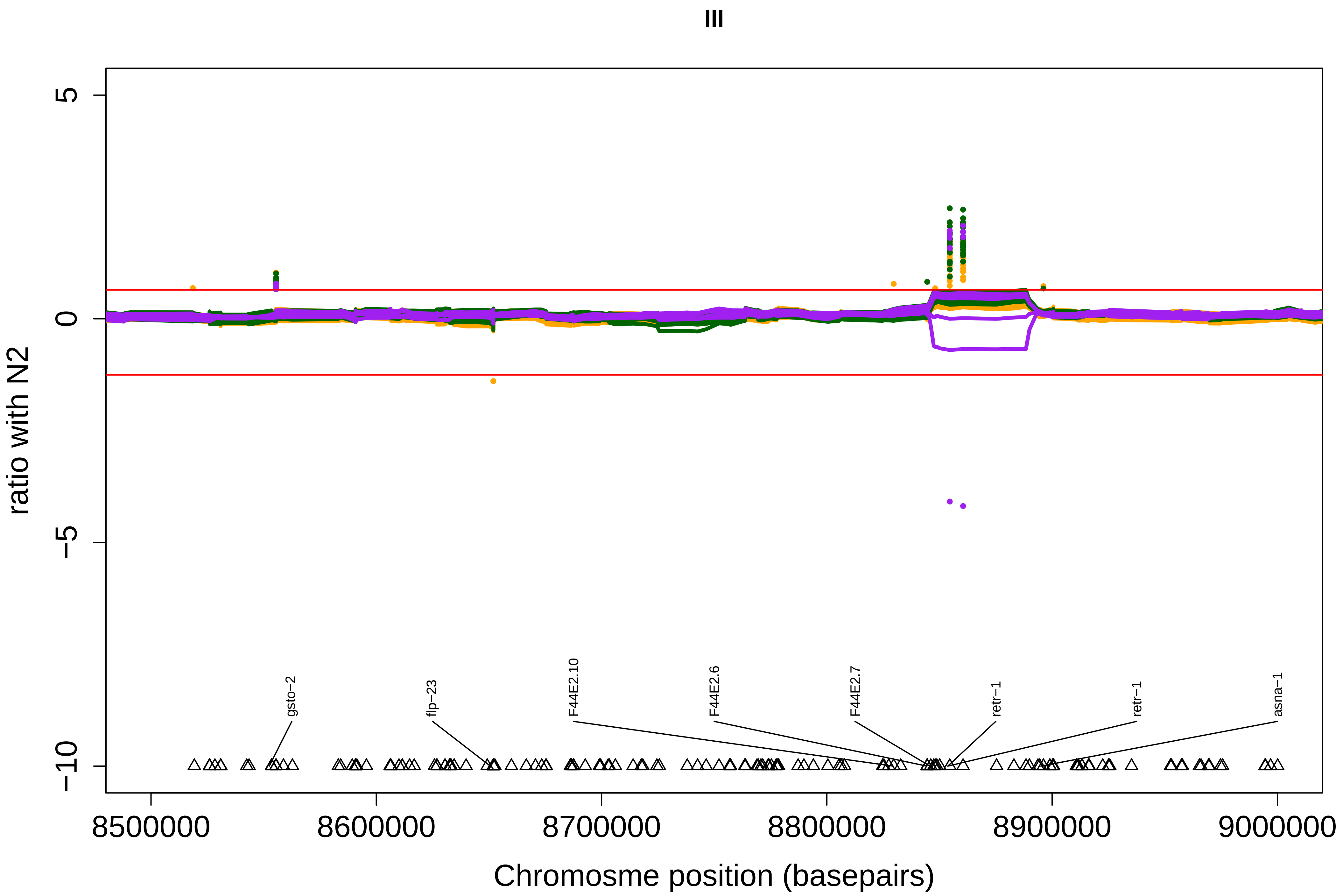
III
ratio with N2
5
0
−5
−10
8500000
8600000
8700000
8800000
8900000
9000000
Chromosme position (basepairs)
gsto−2
flp−23
F44E2.10
F44E2.6
F44E2.7
retr−1
retr−1
asna−1

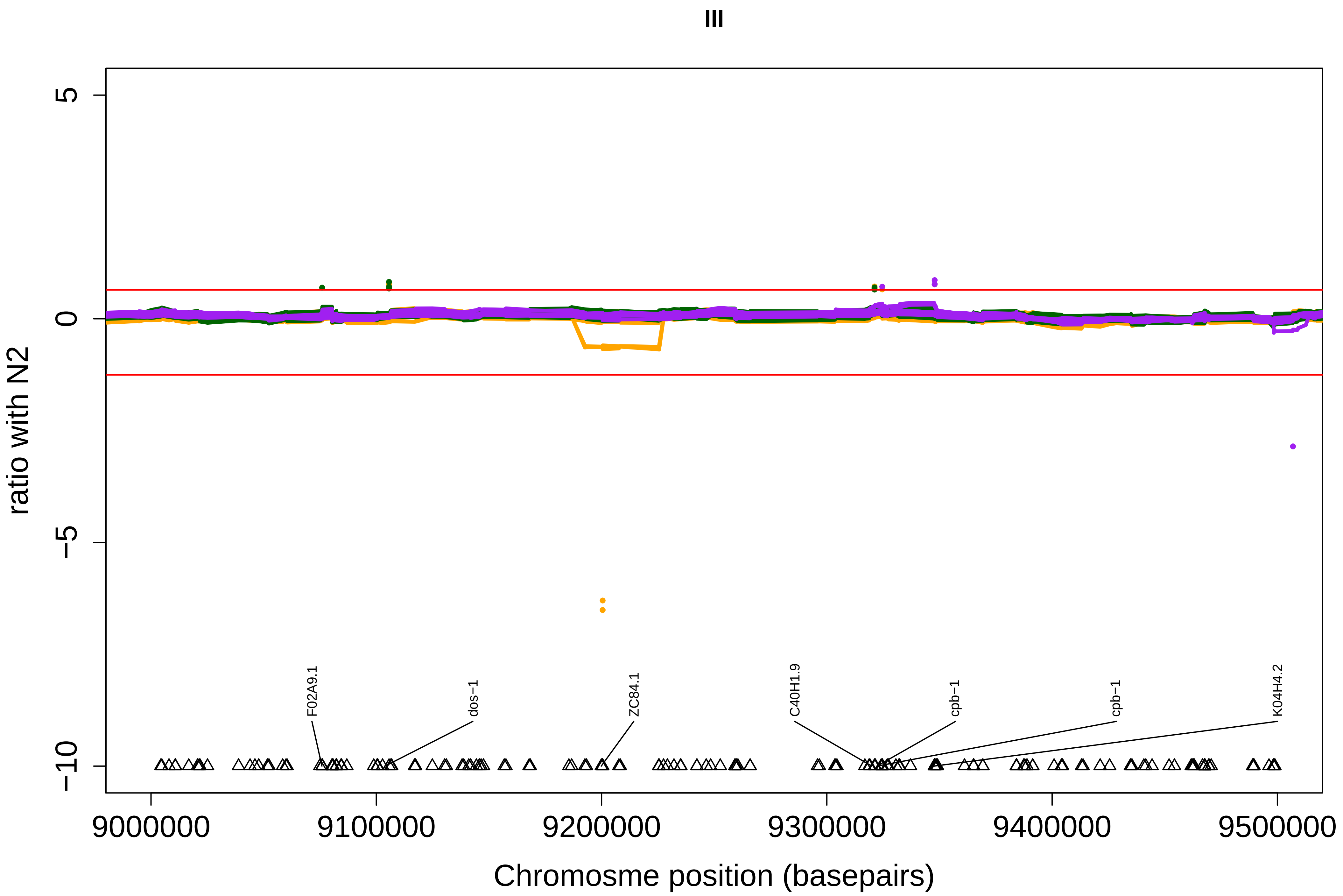
III
ratio with N2
5
0
−5
−10
F02A9.1
dos−1
ZC84.1
C40H1.9
cpb−1
cpb−1
K04H4.2
9000000
9100000
9200000
9300000
9400000
9500000
Chromosme position (basepairs)

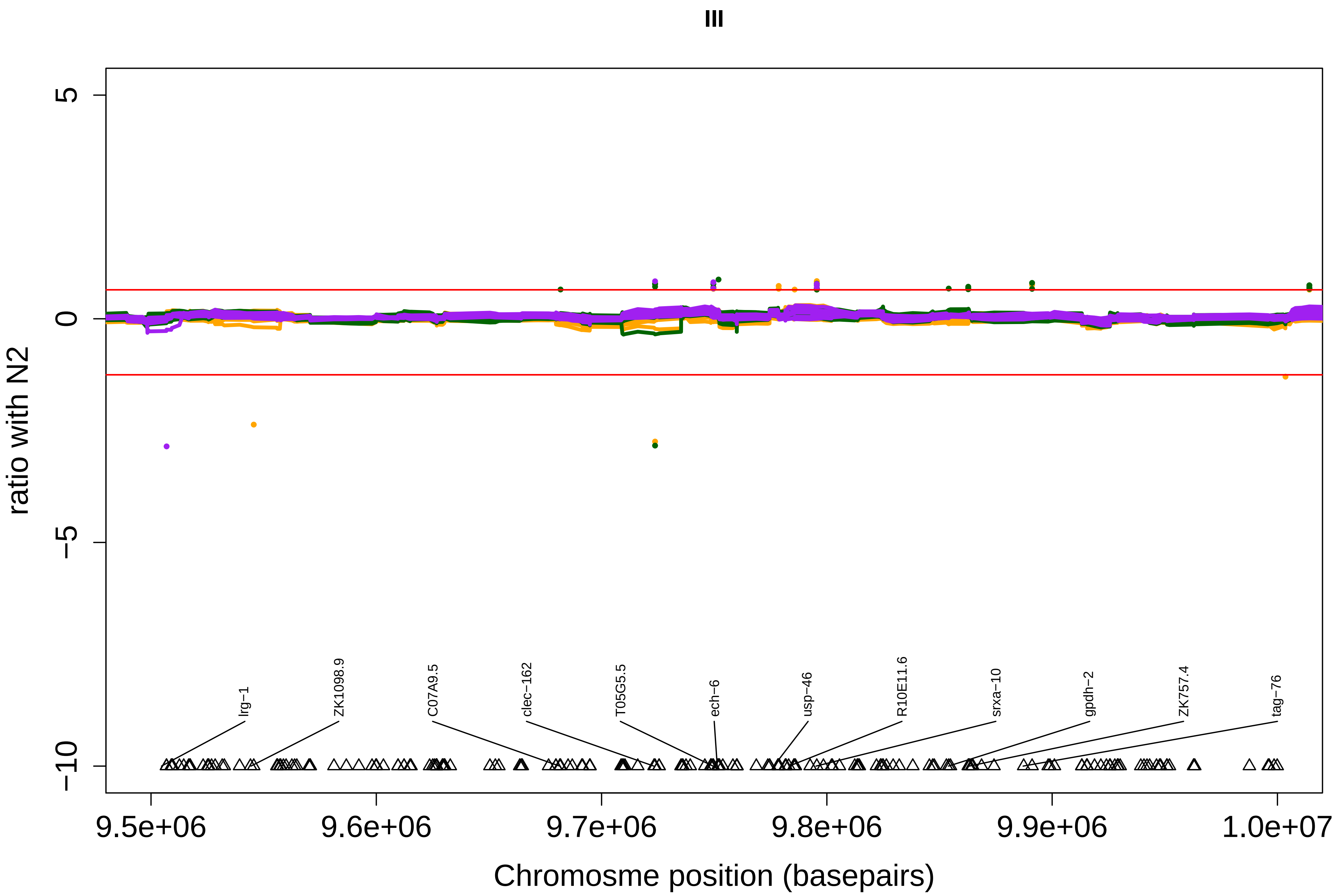
III
ratio with N2
5
0
−5
−10
lrg−1
ZK1098.9
C07A9.5
clec−162
T05G5.5
ech−6
usp−46
R10E11.6
srxa−10
gpdh−2
ZK757.4
tag−76
9.5e+06
9.6e+06
9.7e+06
9.8e+06
9.9e+06
1.0e+07
Chromosme position (basepairs)

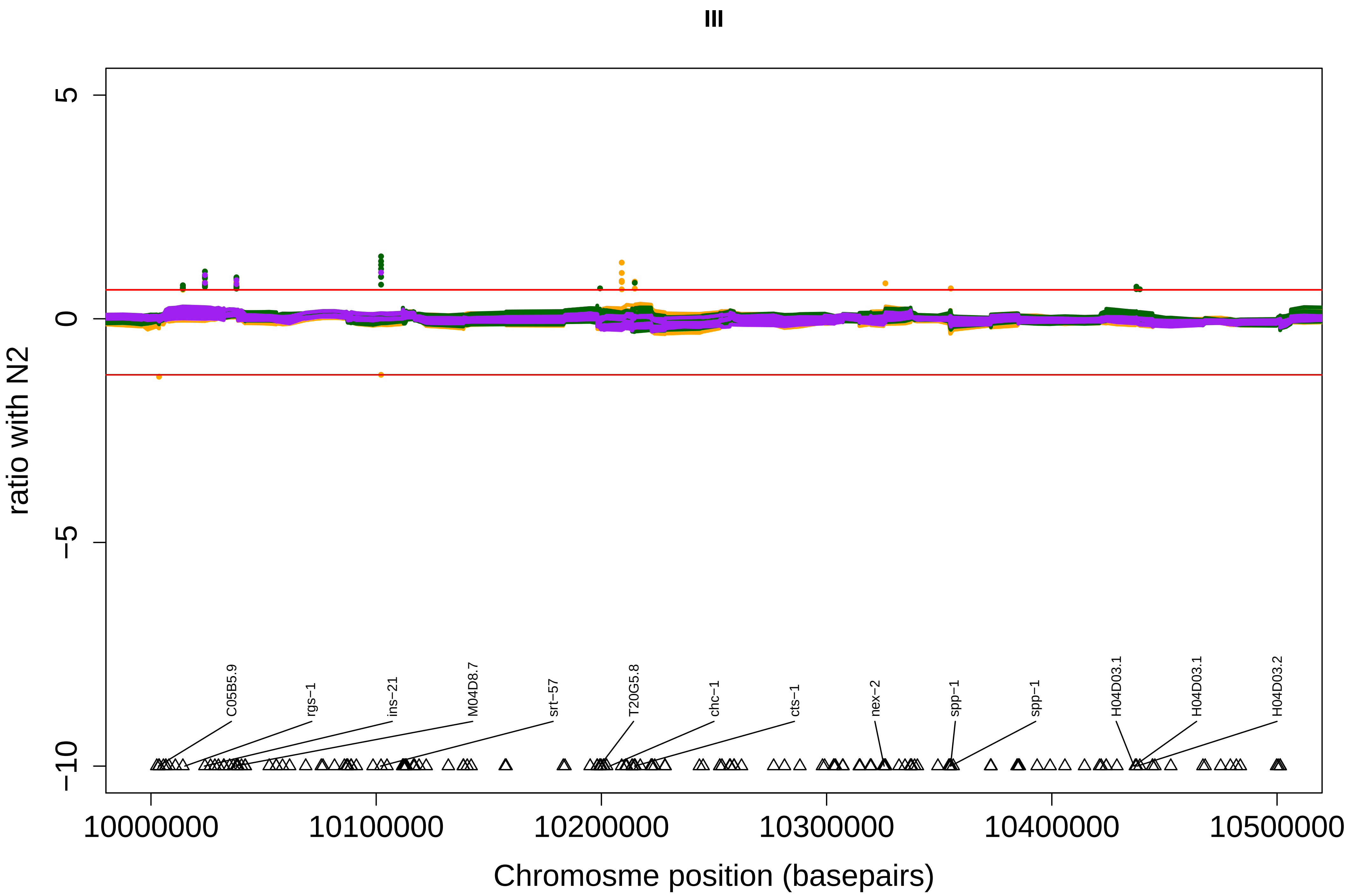

III
5
0
−5
−10
ratio with N2
C05B5.9
rgs−1
ins−21
M04D8.7
srt−57
T20G5.8
chc−1
cts−1
nex−2
spp−1
spp−1
H04D03.1
H04D03.1
H04D03.2
10000000
10100000
10200000
10300000
10400000
10500000
Chromosme position (basepairs)

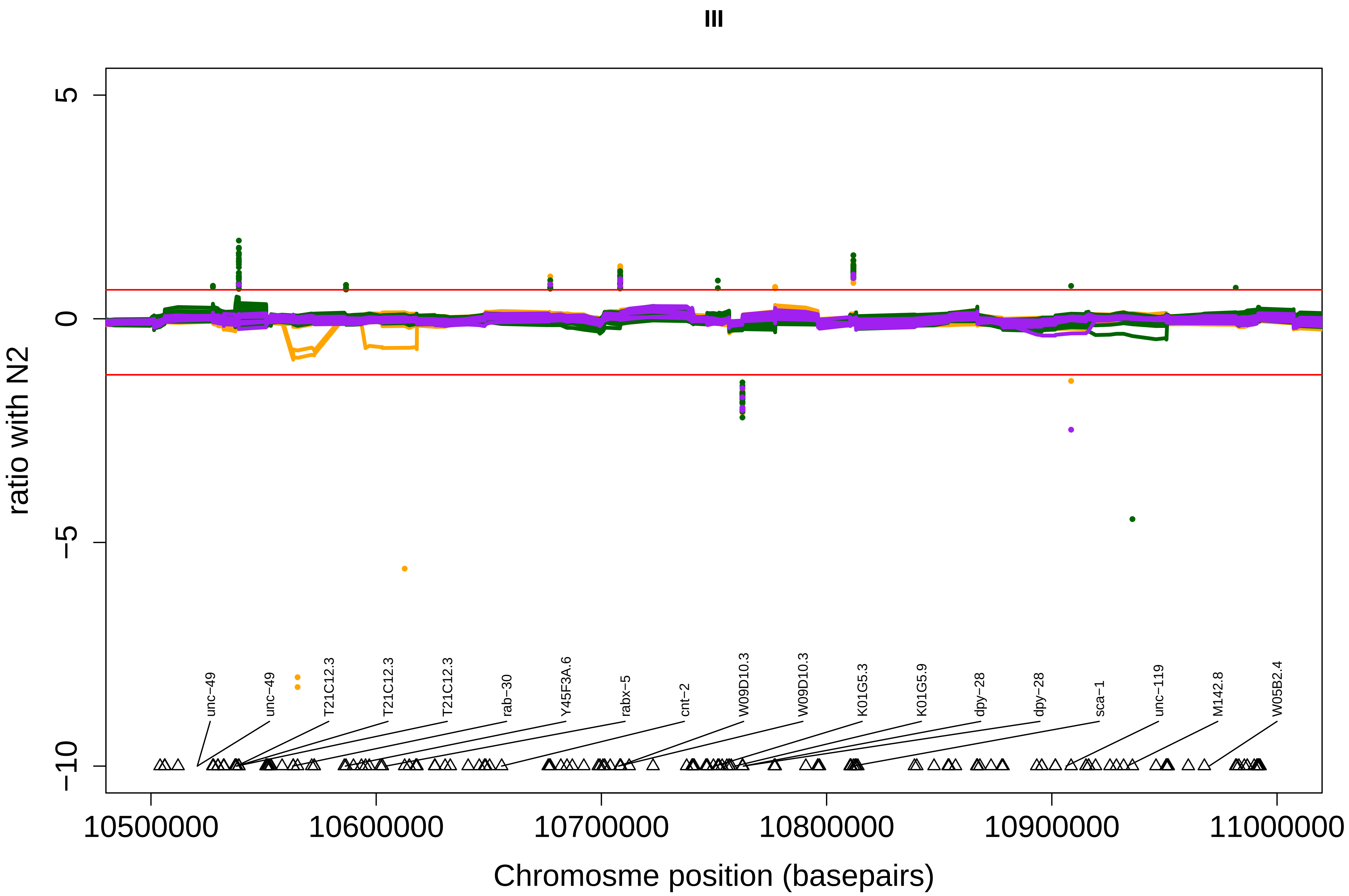

III
ratio with N2
5
0
−5
−10
unc−49
unc−49
T21C12.3
T21C12.3
T21C12.3
rab−30
Y45F3A.6
rabx−5
cnt−2
W09D10.3
W09D10.3
K01G5.3
K01G5.9
dpy−28
dpy−28
sca−1
unc−119
M142.8
W05B2.4
10500000
10600000
10700000
10800000
10900000
11000000
Chromosme position (basepairs)

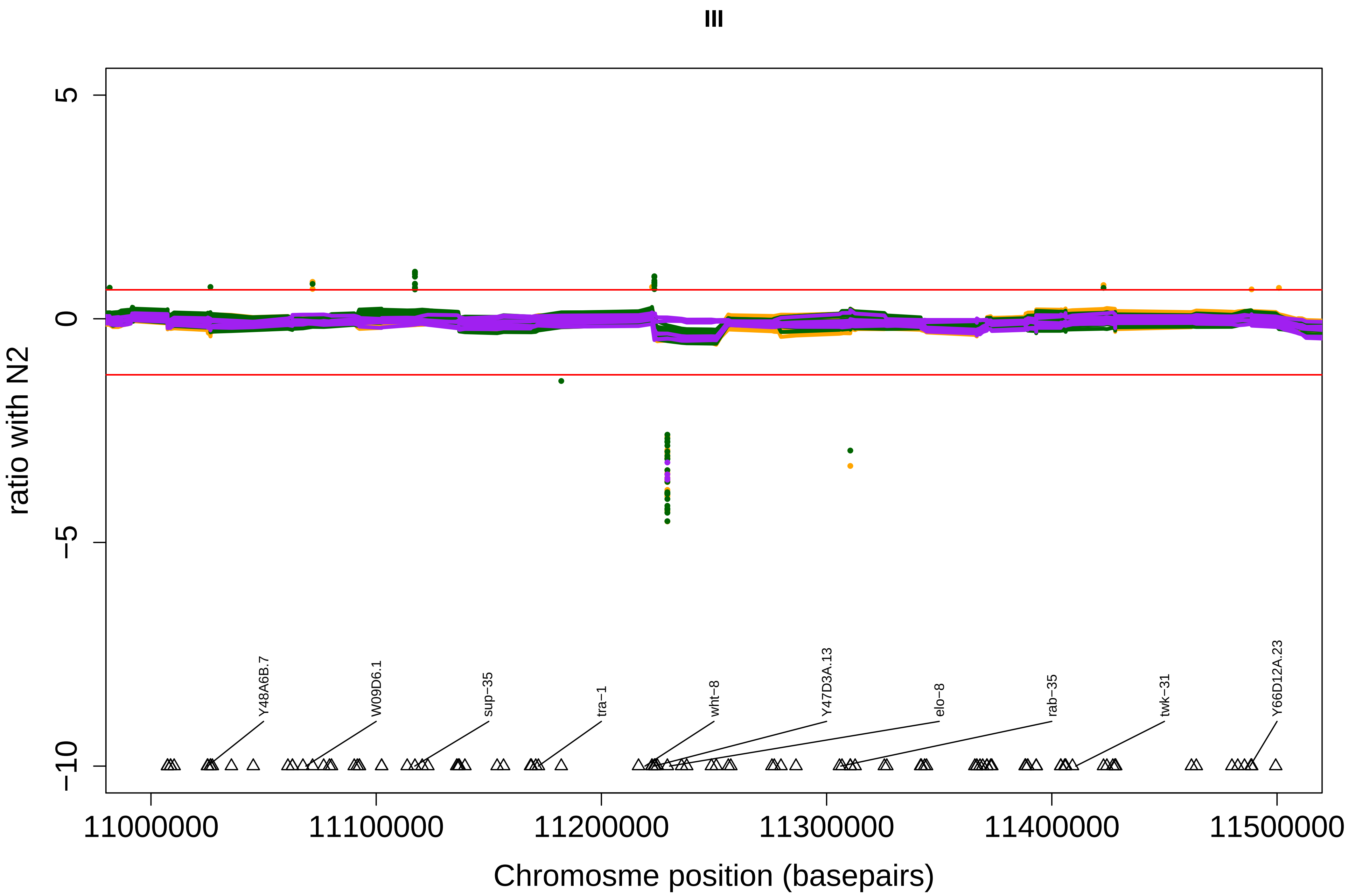
III
ratio with N2
5
0
−5
−10
Y48A6B.7
W09D6.1
sup−35
tra−1
wht−8
Y47D3A.13
elo−8
rab−35
twk−31
Y66D12A.23
11000000
11100000
11200000
11300000
11400000
11500000
Chromosme position (basepairs)

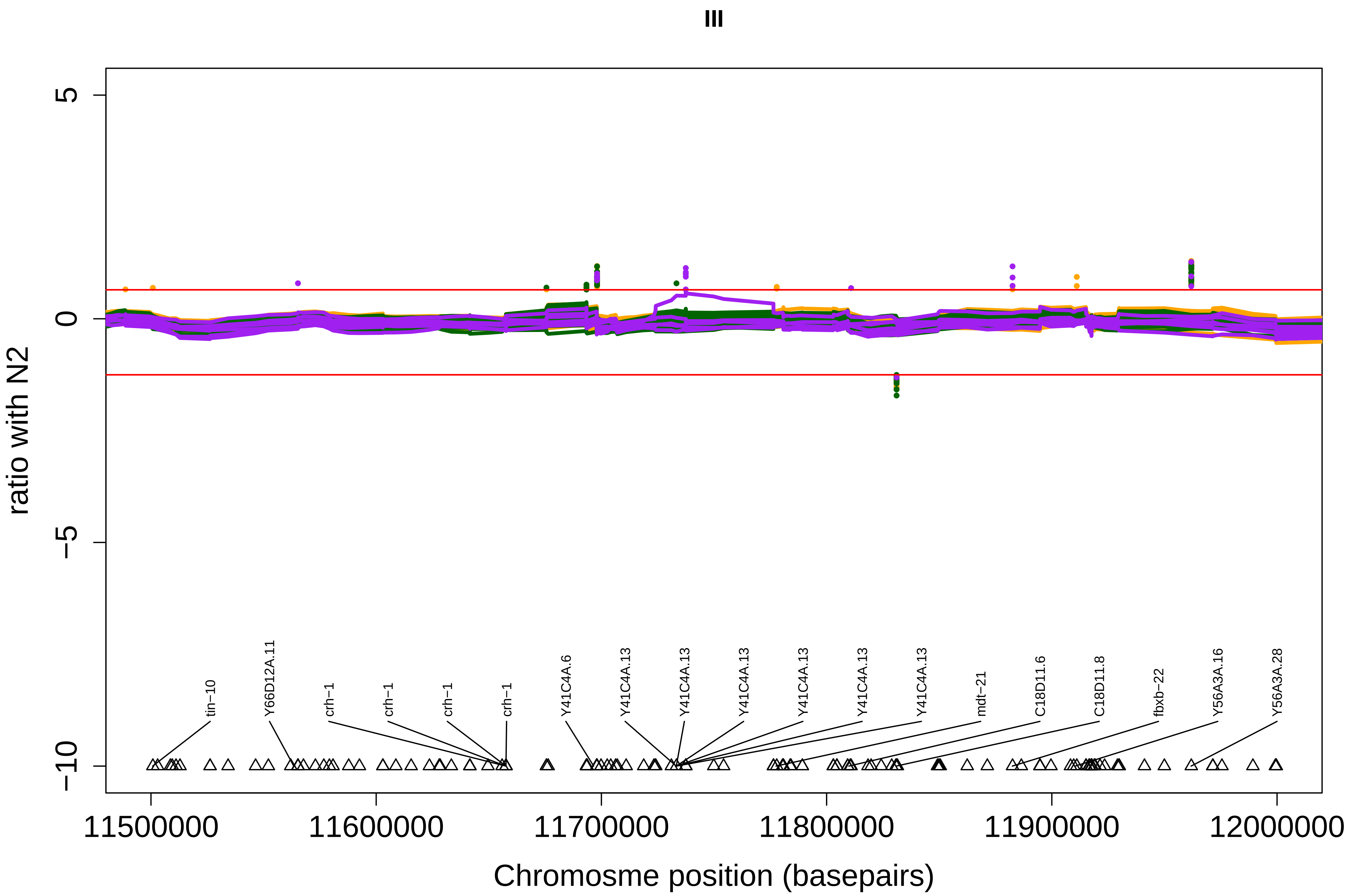

III
ratio with N2
5
0
−5
−10
tin−10
Y66D12A.11
crh−1
crh−1
crh−1
crh−1
Y41C4A.6
Y41C4A.13
Y41C4A.13
Y41C4A.13
Y41C4A.13
Y41C4A.13
Y41C4A.13
mdt−21
C18D11.6
C18D11.8
fbxb−22
Y56A3A.16
Y56A3A.28
11500000
11600000
11700000
11800000
11900000
12000000
Chromosme position (basepairs)

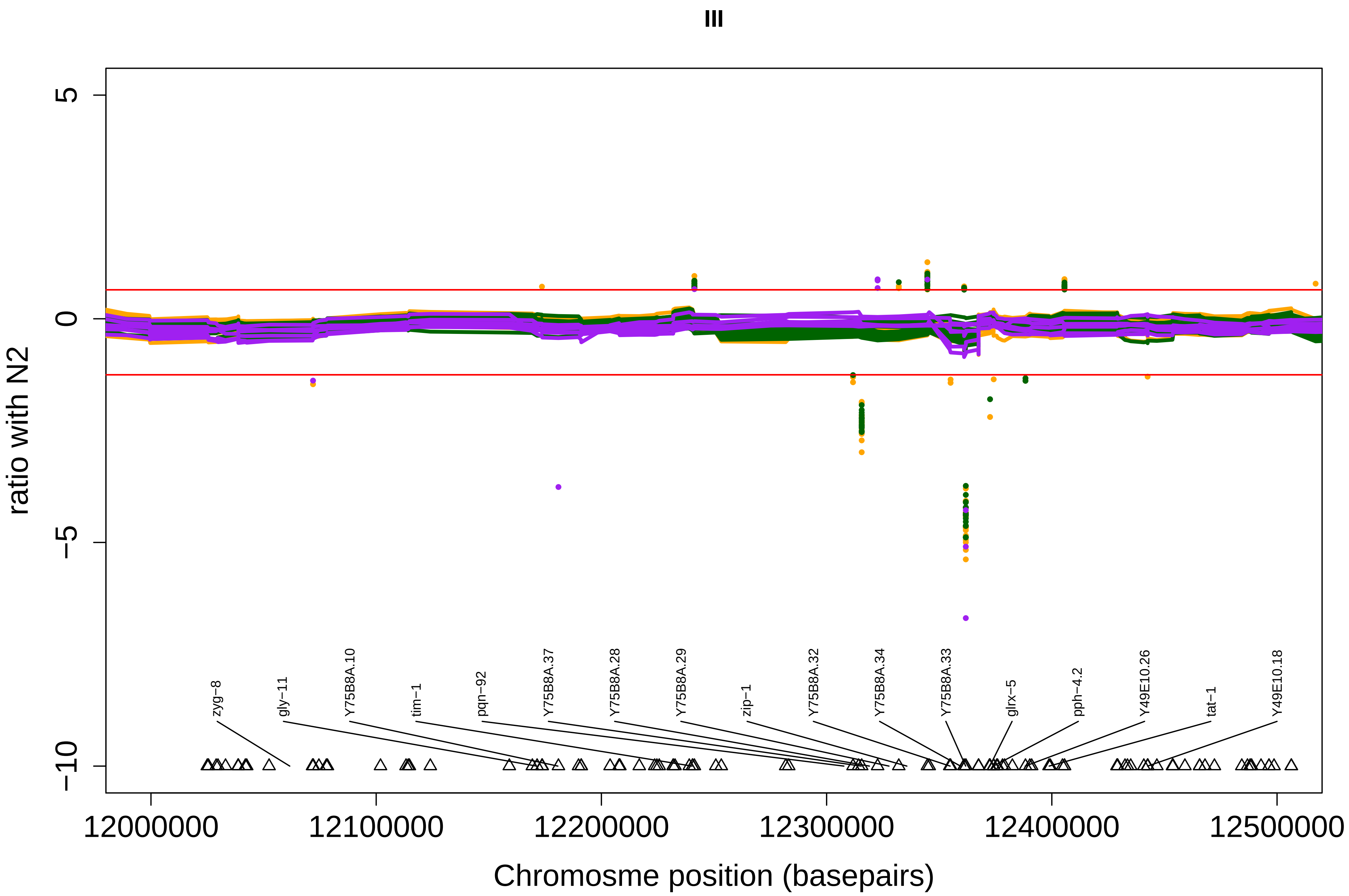

III
ratio with N2
5
0
−5
−10
zyg−8
gly−11
Y75B8A.10
tim−1
pqn−92
Y75B8A.37
Y75B8A.28
Y75B8A.29
zip−1
Y75B8A.32
Y75B8A.34
Y75B8A.33
glrx−5
pph−4.2
Y49E10.26
tat−1
Y49E10.18
12000000
12100000
12200000
12300000
12400000
12500000
Chromosme position (basepairs)

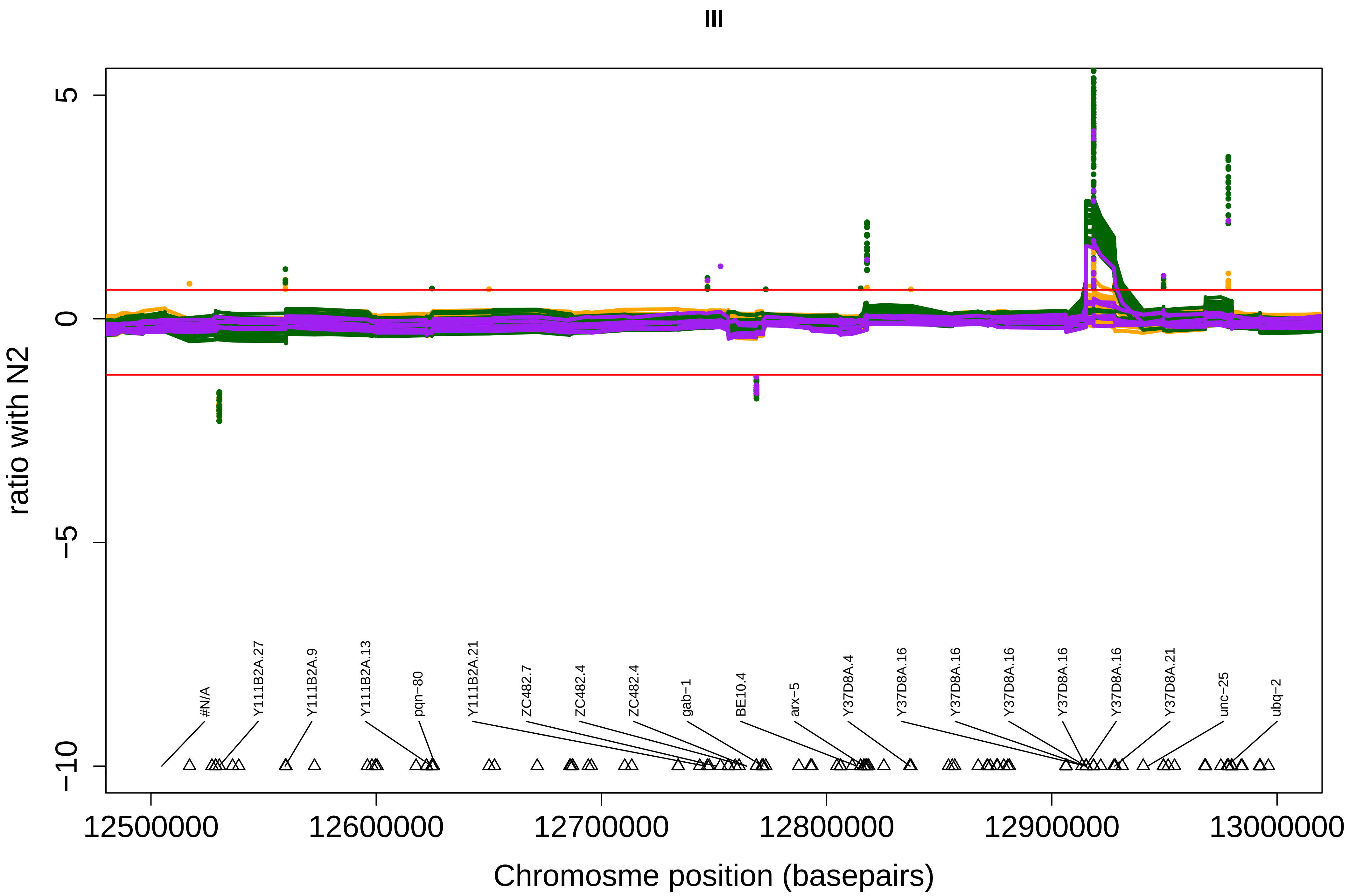
III
ratio with N2
5
0
−5
−10
#N/A
Y111B2A.27
Y111B2A.9
Y111B2A.13
pqn−80
Y111B2A.21
ZC482.7
ZC482.4
ZC482.4
gab−1
BE10.4
arx−5
Y37D8A.4
Y37D8A.16
Y37D8A.16
Y37D8A.16
Y37D8A.16
Y37D8A.16
Y37D8A.21
unc−25
ubq−2
12500000
12600000
12700000
12800000
12900000
13000000
Chromosme position (basepairs)

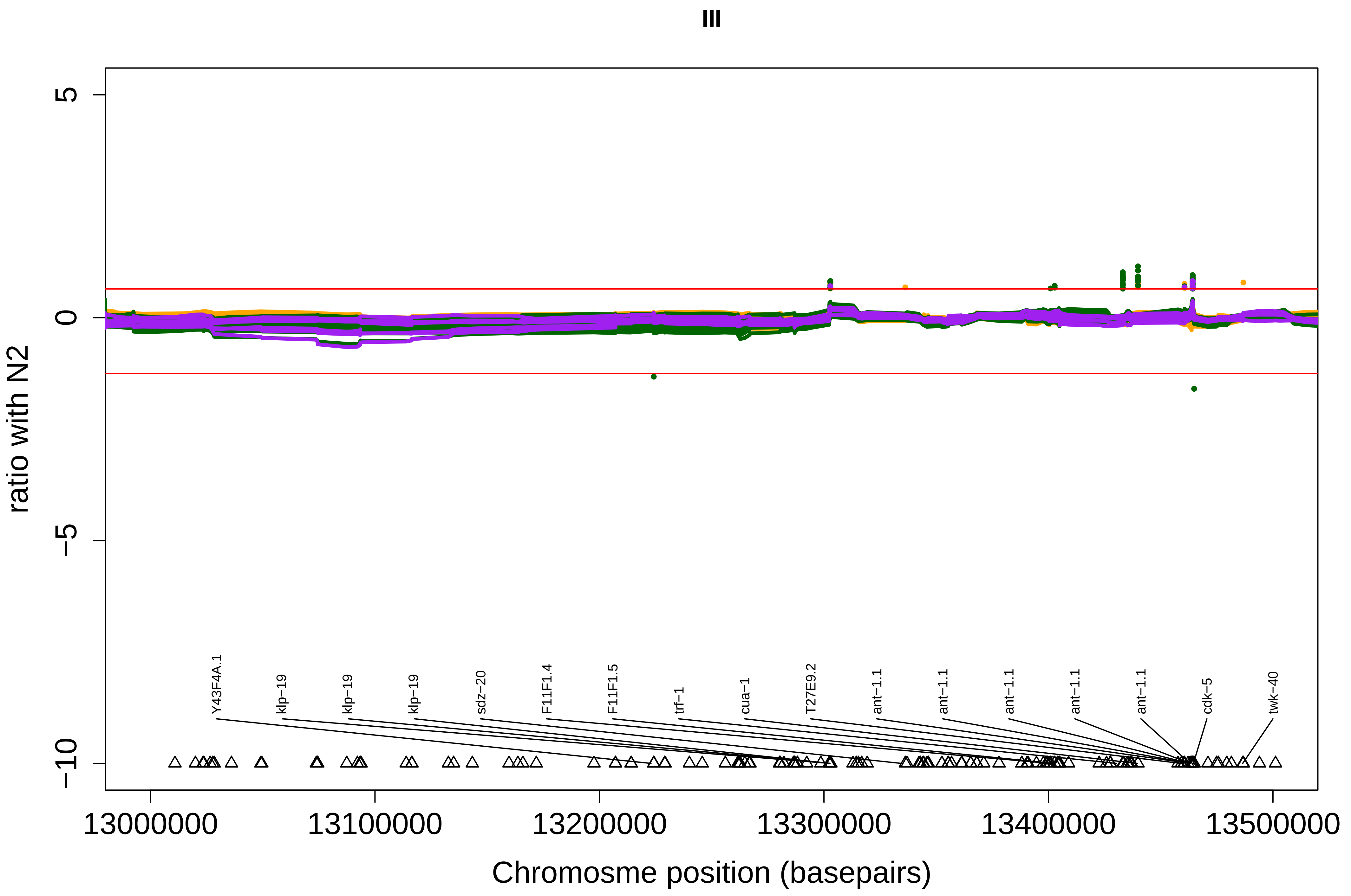

III
ratio with N2
5
0
−5
−10
Y43F4A.1
klp−19
klp−19
klp−19
sdz−20
F11F1.4
F11F1.5
trf−1
cua−1
T27E9.2
ant−1.1
ant−1.1
ant−1.1
ant−1.1
ant−1.1
cdk−5
twk−40
13000000
13100000
13200000
13300000
13400000
13500000
Chromosme position (basepairs)

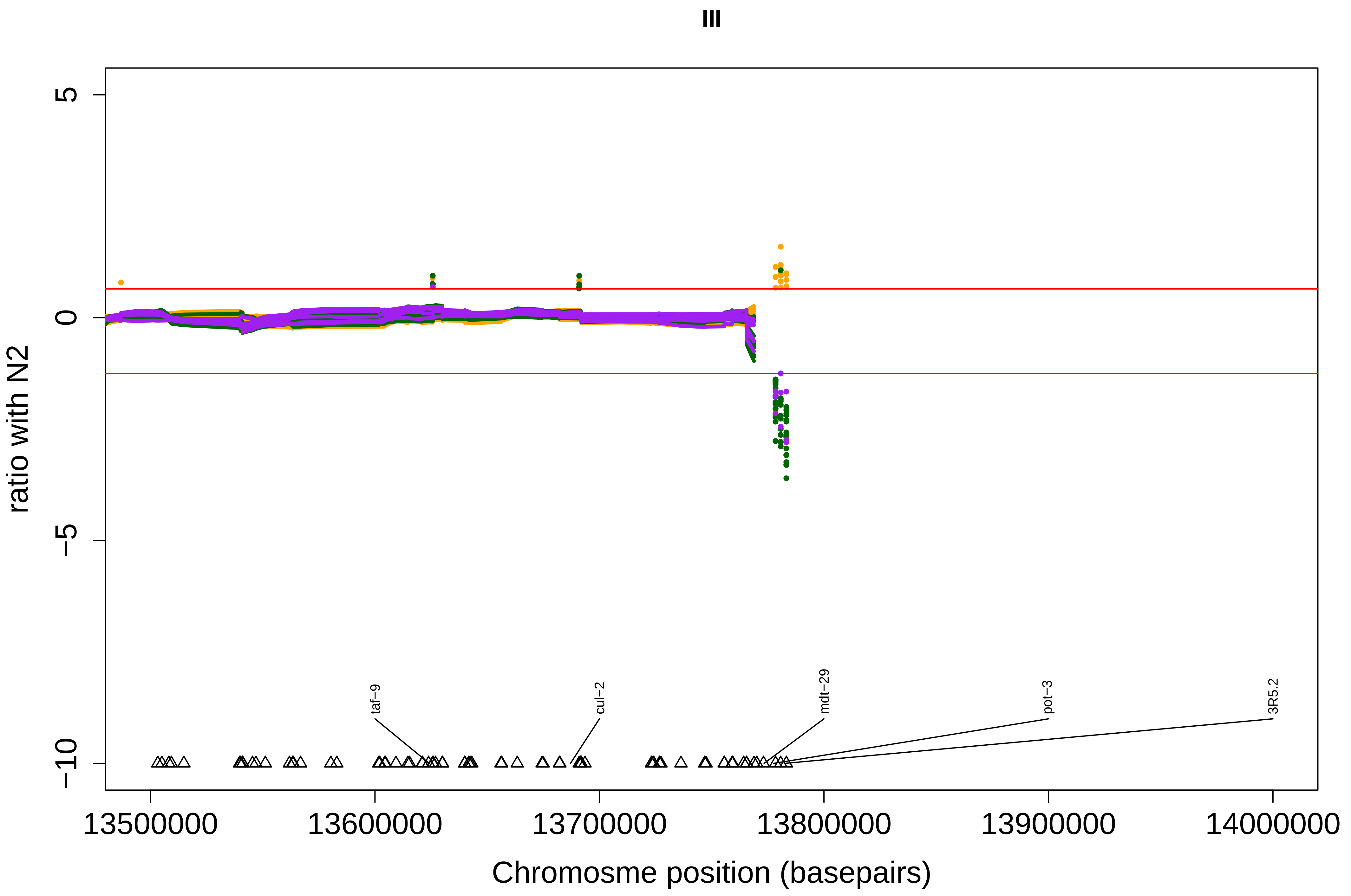

III
ratio with N2
5
0
−5
−10
taf−9
cul−2
mdt−29
pot−3
3R5.2
13500000
13600000
13700000
13800000
13900000
14000000
Chromosme position (basepairs)

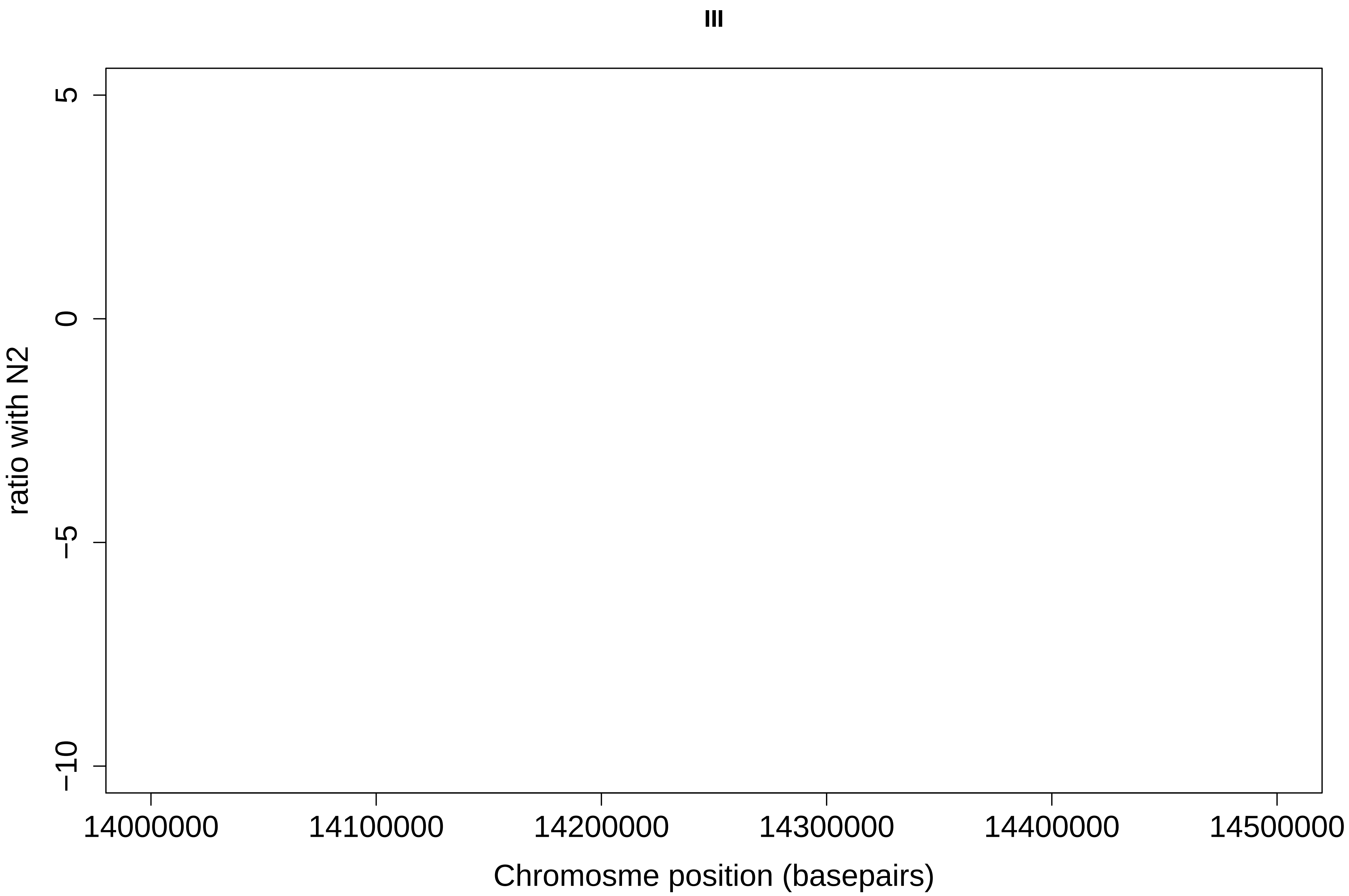
III
5
0
−5
−10
ratio with N2
14000000
14100000
14200000
14300000
14400000
14500000
Chromosme position (basepairs)

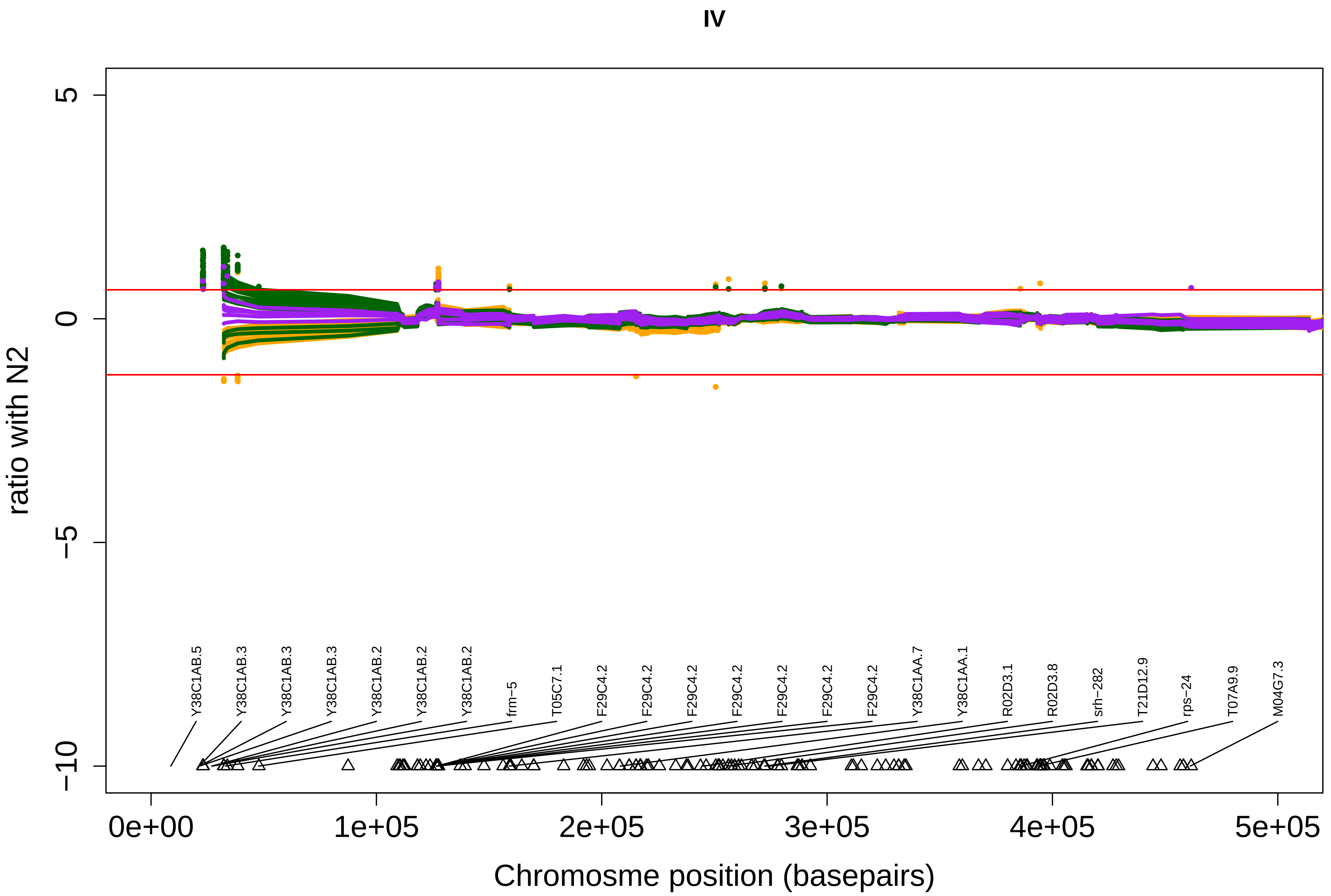
IV
ratio with N2
5
0
−5
−10
0e+00
1e+05
2e+05
3e+05
4e+05
5e+05
Chromosme position (basepairs)
Y38C1AB.5
Y38C1AB.3
Y38C1AB.3
Y38C1AB.3
Y38C1AB.2
Y38C1AB.2
Y38C1AB.2
frm−5
T05C7.1
F29C4.2
F29C4.2
F29C4.2
F29C4.2
F29C4.2
F29C4.2
F29C4.2
Y38C1AA.7
Y38C1AA.1
R02D3.1
R02D3.8
srh−282
T21D12.9
rps−24
T07A9.9
M04G7.3

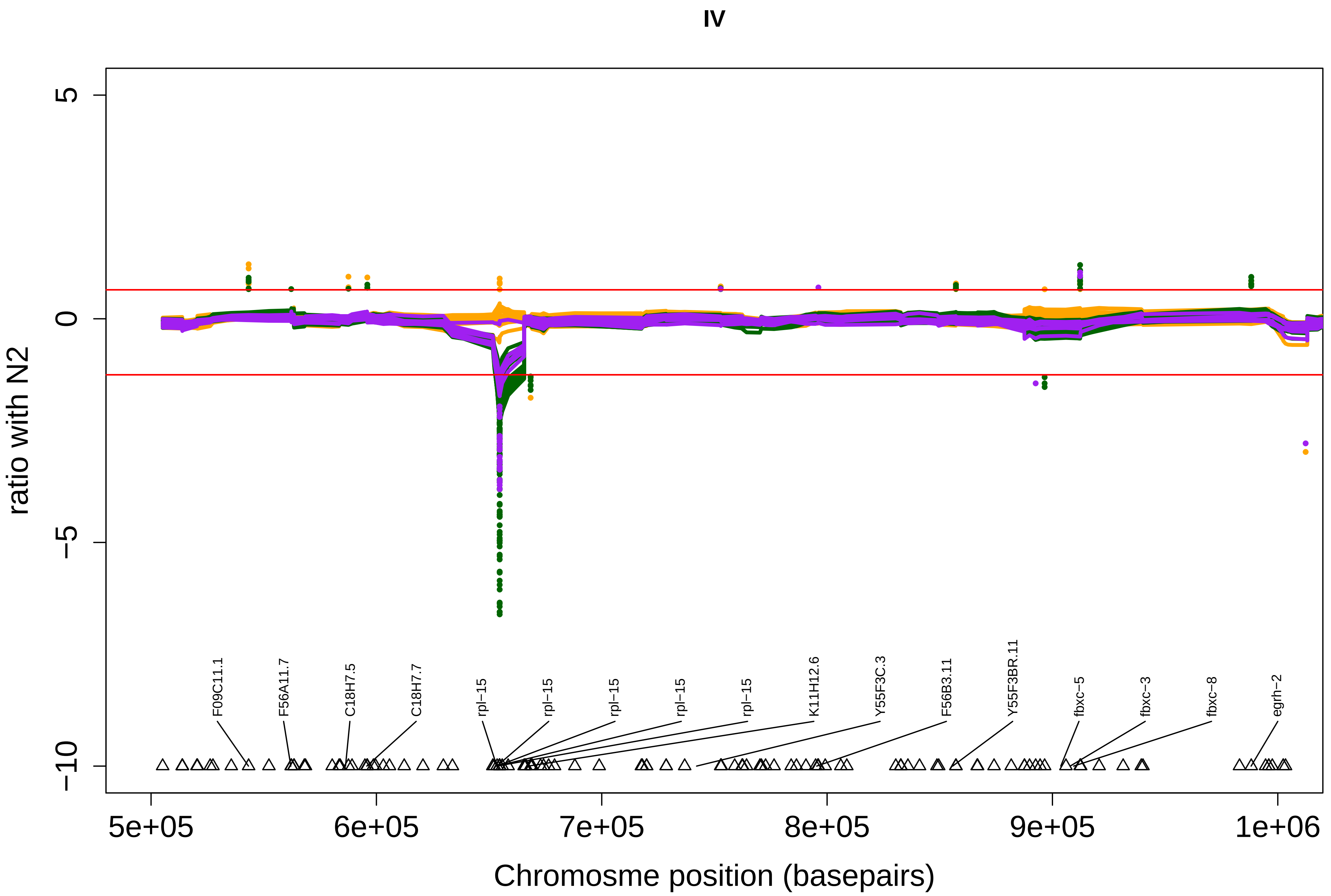
IV
ratio with N2
5
0
−5
−10
5e+05
6e+05
7e+05
8e+05
9e+05
1e+06
Chromosme position (basepairs)
F09C11.1
F56A11.7
C18H7.5
C18H7.7
rpl−15
rpl−15
rpl−15
rpl−15
rpl−15
K11H12.6
Y55F3C.3
F56B3.11
Y55F3BR.11
fbxc−5
fbxc−3
fbxc−8
egrh−2

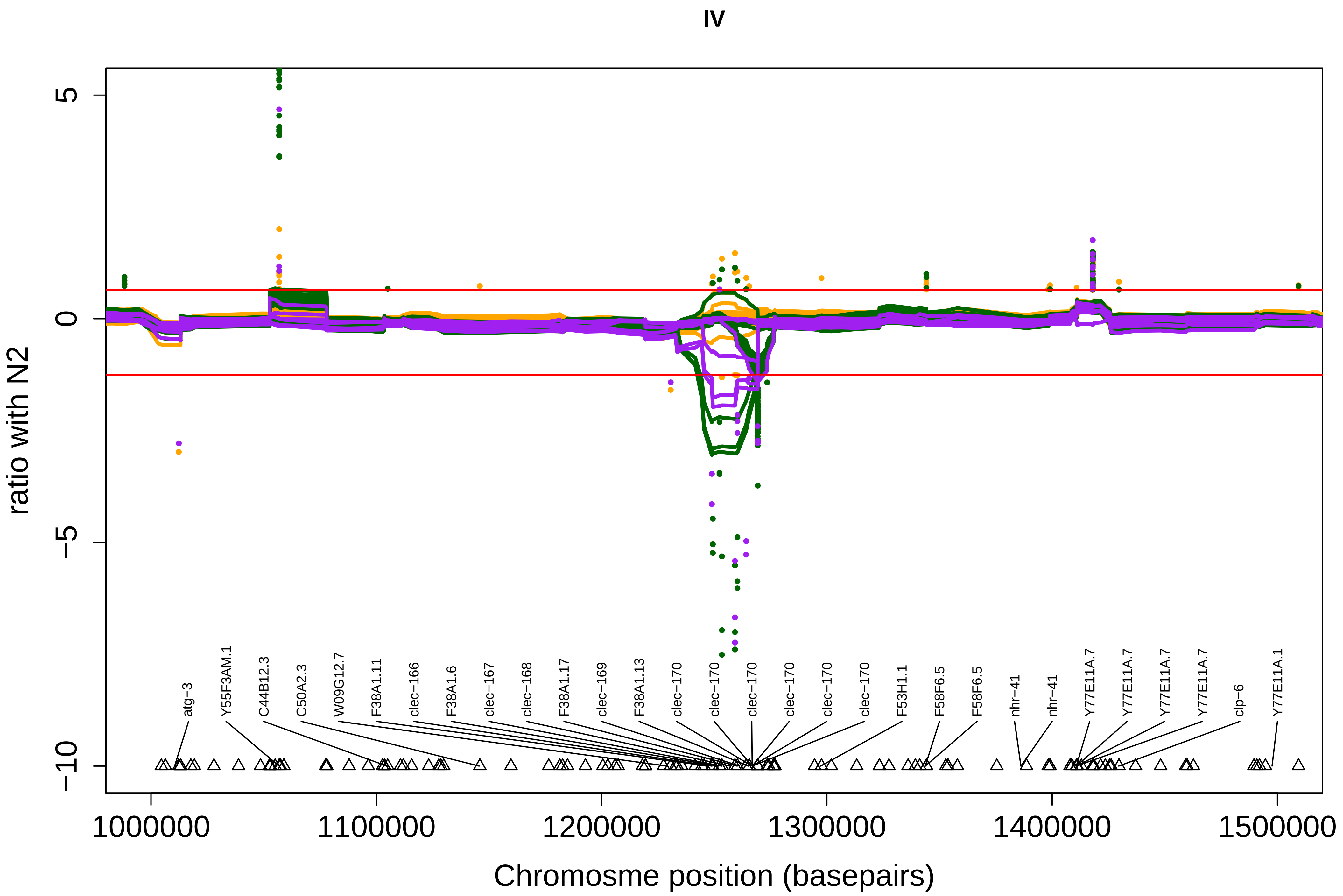

IV
ratio with N2
5
0
−5
−10
atg−3
Y55F3AM.1
C44B12.3
C50A2.3
W09G12.7
F38A1.11
clec−166
F38A1.6
clec−167
clec−168
F38A1.17
clec−169
F38A1.13
clec−170
clec−170
clec−170
clec−170
clec−170
clec−170
F53H1.1
F58F6.5
F58F6.5
nhr−41
nhr−41
Y77E11A.7
Y77E11A.7
Y77E11A.7
Y77E11A.7
clp−6
Y77E11A.1
1000000
1100000
1200000
1300000
1400000
1500000
Chromosme position (basepairs)

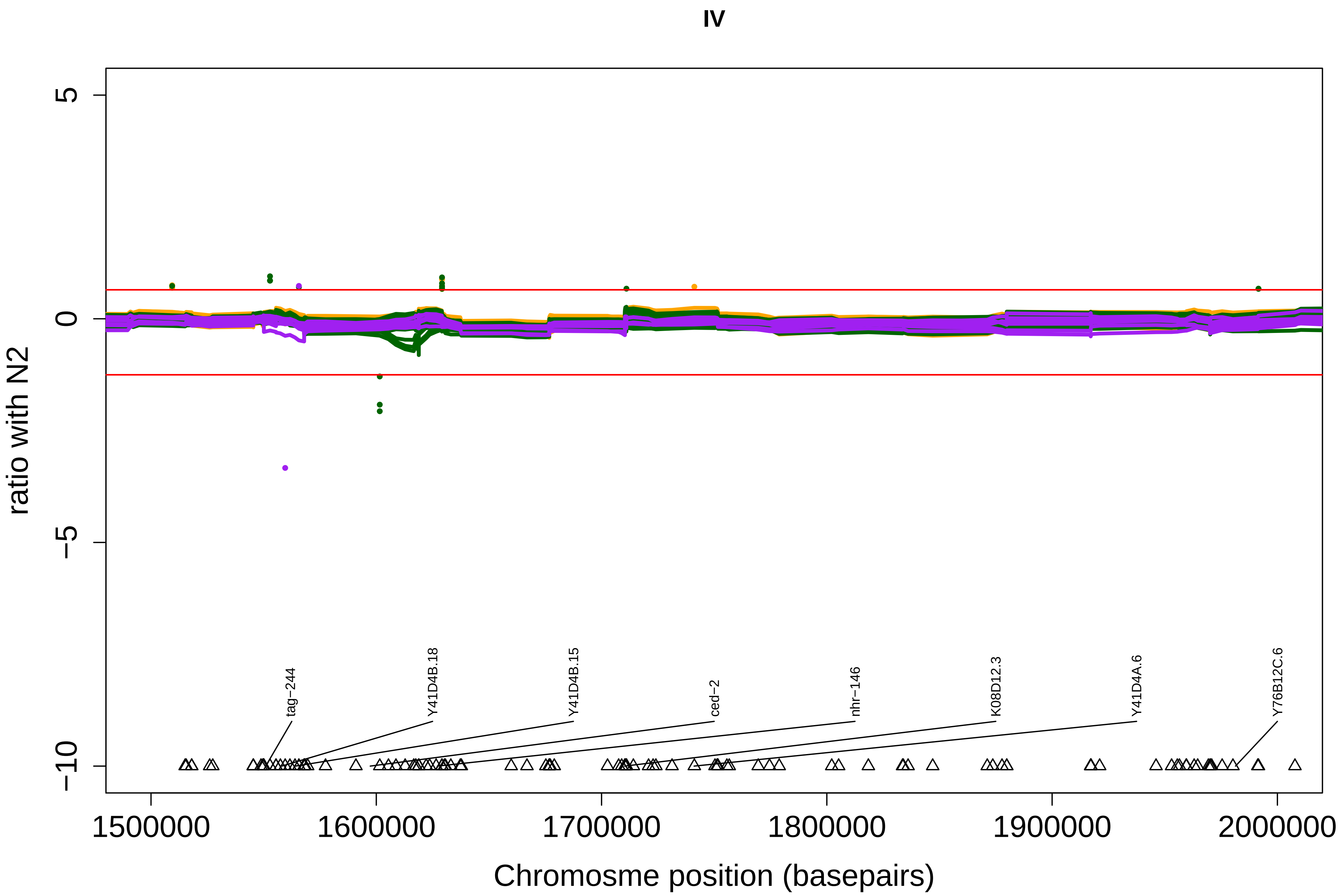
IV
ratio with N2
5
0
−5
−10
tag−244
Y41D4B.18
Y41D4B.15
ced−2
nhr−146
K08D12.3
Y41D4A.6
Y76B12C.6
1500000
1600000
1700000
1800000
1900000
2000000
Chromosme position (basepairs)

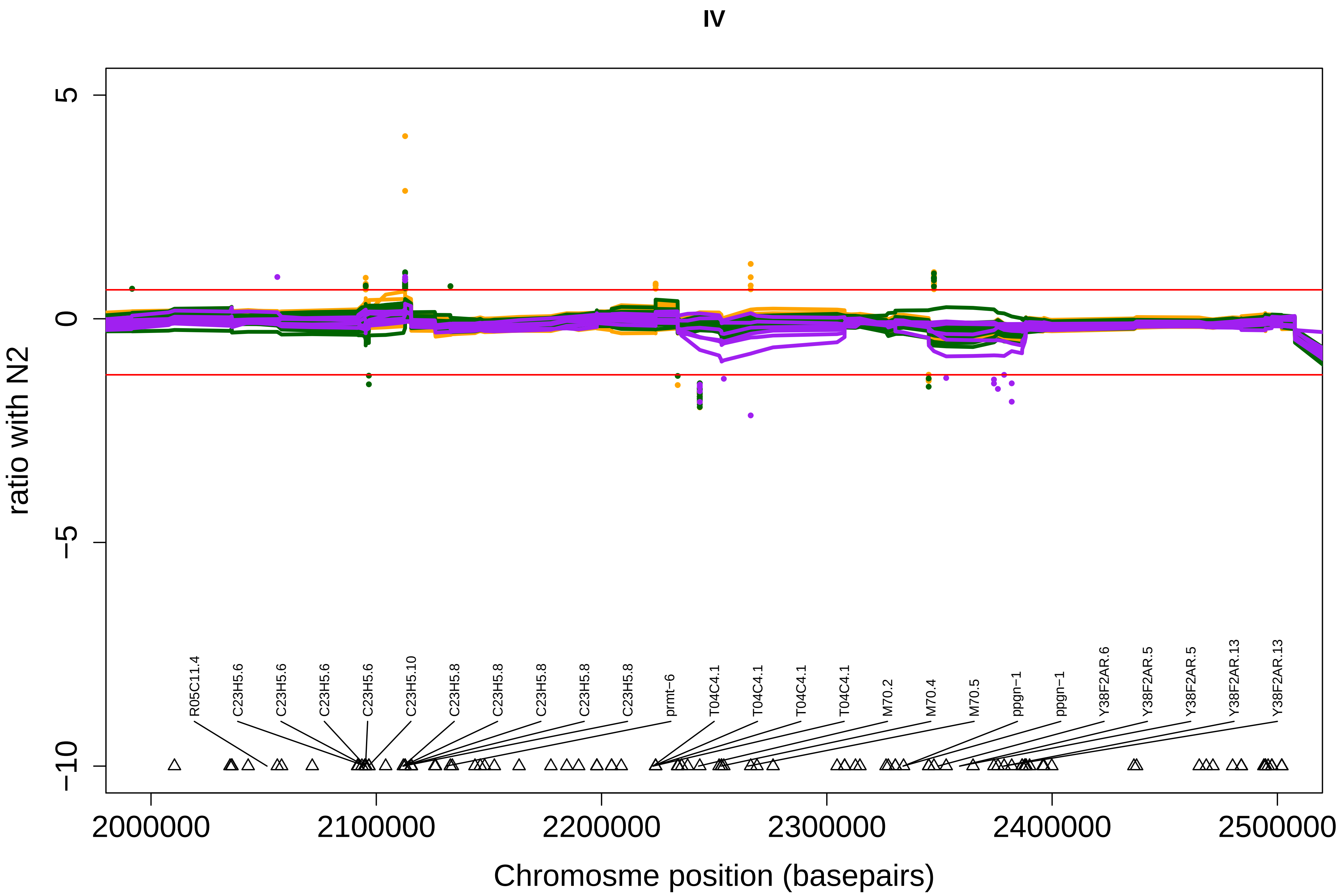
IV
ratio with N2
5
0
−5
−10
R05C11.4
C23H5.6
C23H5.6
C23H5.6
C23H5.6
C23H5.10
C23H5.8
C23H5.8
C23H5.8
C23H5.8
C23H5.8
prmt−6
T04C4.1
T04C4.1
T04C4.1
T04C4.1
M70.2
M70.4
M70.5
ppgn−1
ppgn−1
Y38F2AR.6
Y38F2AR.5
Y38F2AR.5
Y38F2AR.13
Y38F2AR.13
2000000
2100000
2200000
2300000
2400000
2500000
Chromosme position (basepairs)

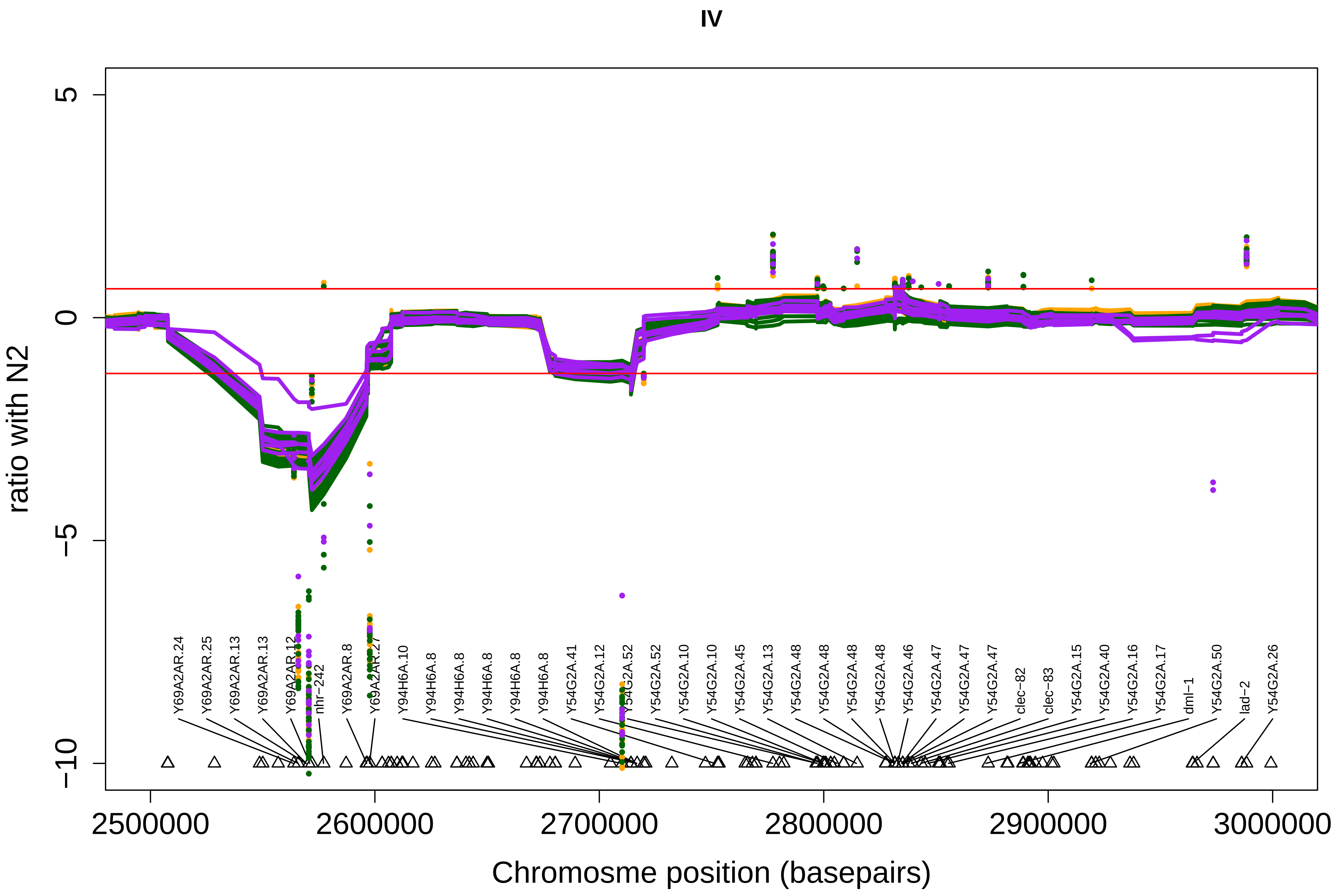
IV
ratio with N2
Chromosme position (basepairs)
5
0
−5
−10
2500000
2600000
2700000
2800000
2900000
3000000
Y69A2AR.24
Y69A2AR.25
Y69A2AR.13
Y69A2AR.13
Y69A2AR.12
nhr−242
Y69A2AR.8
Y69A2AR.27
Y94H6A.10
Y94H6A.8
Y94H6A.8
Y94H6A.8
Y94H6A.8
Y94H6A.8
Y54G2A.41
Y54G2A.12
Y54G2A.52
Y54G2A.52
Y54G2A.10
Y54G2A.10
Y54G2A.45
Y54G2A.13
Y54G2A.48
Y54G2A.48
Y54G2A.48
Y54G2A.48
Y54G2A.46
Y54G2A.47
Y54G2A.47
Y54G2A.47
clec−82
clec−83
Y54G2A.15
Y54G2A.40
Y54G2A.16
Y54G2A.17
dml−1
Y54G2A.50
lad−2
Y54G2A.26

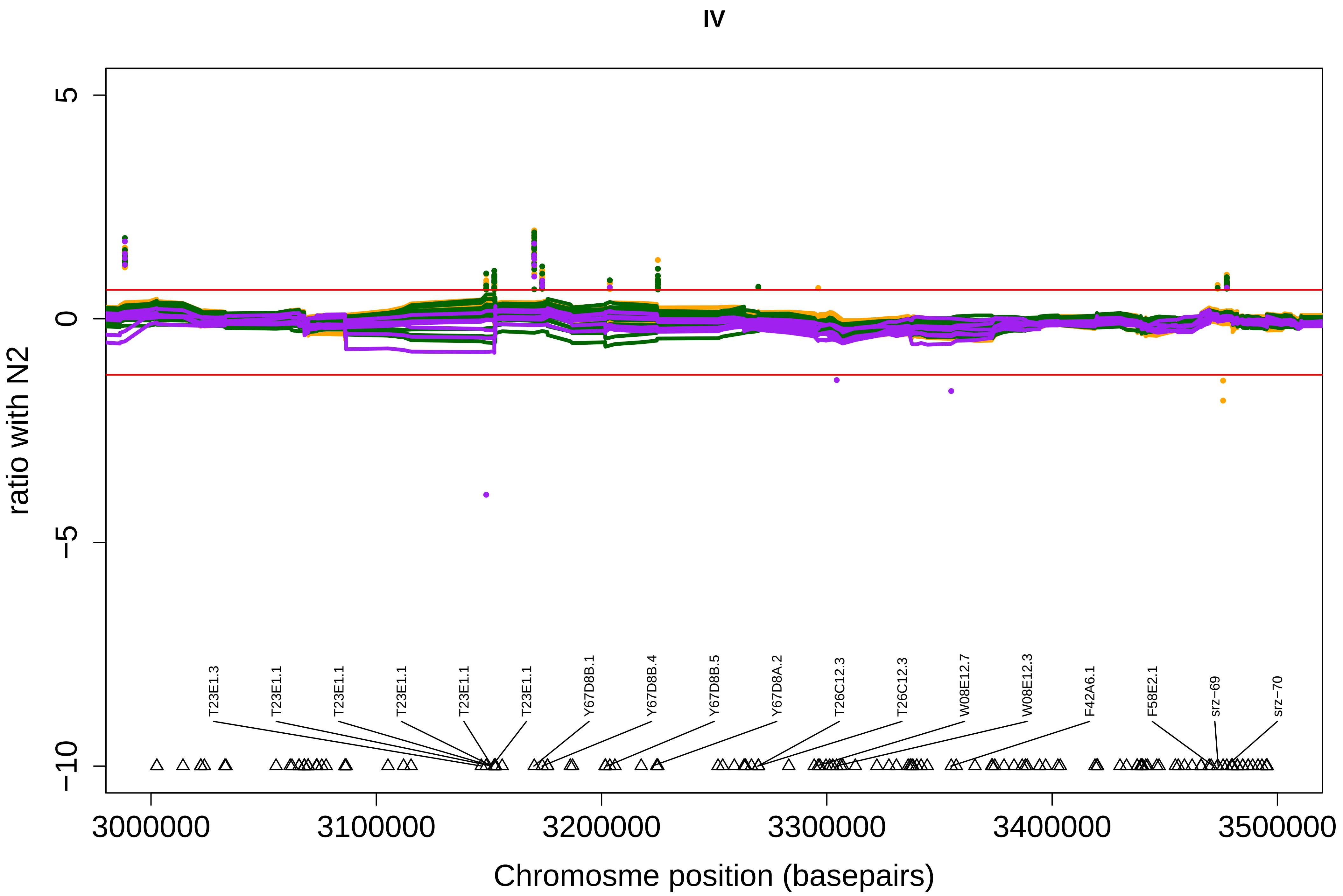
IV
ratio with N2
5
0
−5
−10
3000000
3100000
3200000
3300000
3400000
3500000
Chromosme position (basepairs)
T23E1.3
T23E1.1
T23E1.1
T23E1.1
T23E1.1
T23E1.1
Y67D8B.1
Y67D8B.4
Y67D8B.5
Y67D8A.2
T26C12.3
T26C12.3
W08E12.7
W08E12.3
F42A6.1
F58E2.1
srz−69
srz−70

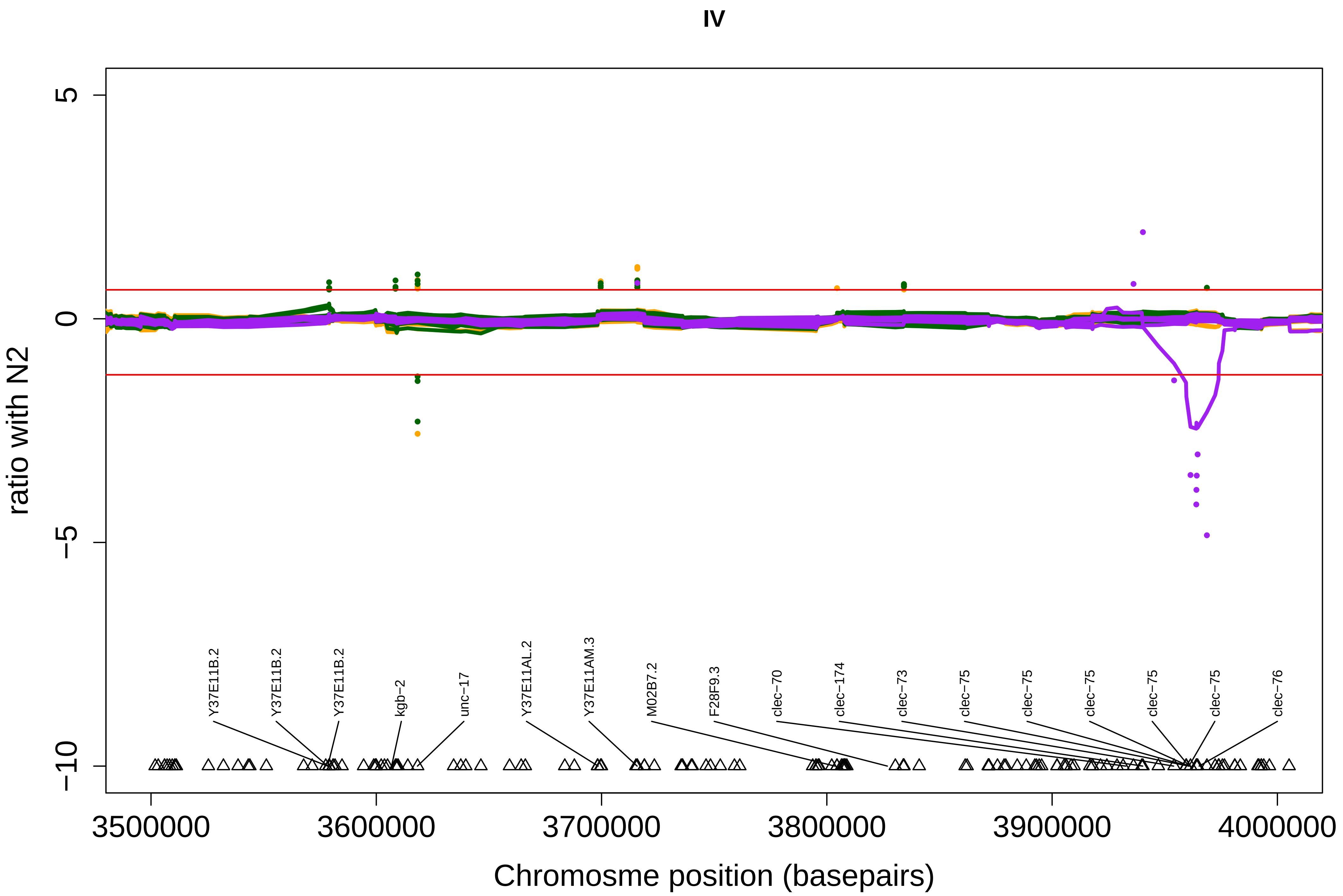
IV
ratio with N2
5
0
−5
−10
3500000
3600000
3700000
3800000
3900000
4000000
Chromosme position (basepairs)
Y37E11B.2
Y37E11B.2
Y37E11B.2
kgb−2
unc−17
Y37E11AL.2
Y37E11AM.3
M02B7.2
F28F9.3
clec−70
clec−174
clec−73
clec−75
clec−75
clec−75
clec−75
clec−75
clec−76

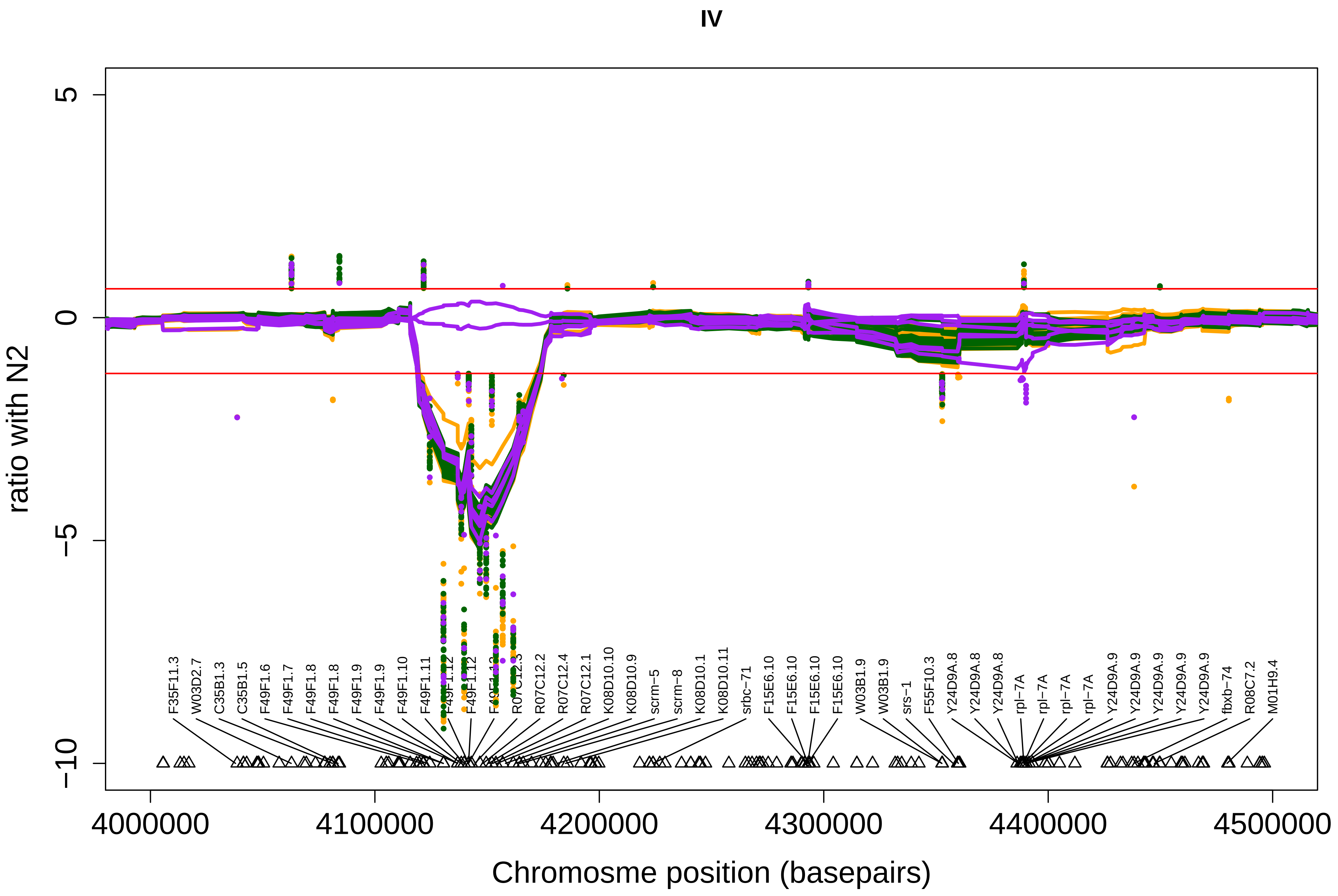
IV
ratio with N2
Chromosme position (basepairs)
5
0
−5
−10
4000000
4100000
4200000
4300000
4400000
4500000
F35F11.3
W03D2.7
C35B1.3
C35B1.5
F49F1.6
F49F1.7
F49F1.8
F49F1.8
F49F1.9
F49F1.9
F49F1.10
F49F1.11
F49F1.12
F49F1.12
R07C12.2
R07C12.4
R07C12.1
K08D10.10
K08D10.9
scrm−5
scrm−8
K08D10.1
K08D10.11
srbc−71
F15E6.10
F15E6.10
F15E6.10
F15E6.10
W03B1.9
W03B1.9
srs−1
F55F10.3
Y24D9A.8
Y24D9A.8
Y24D9A.8
rpl−7A
rpl−7A
rpl−7A
rpl−7A
Y24D9A.9
Y24D9A.9
Y24D9A.9
Y24D9A.9
Y24D9A.9
fbxb−74
R08C7.2
M01H9.4

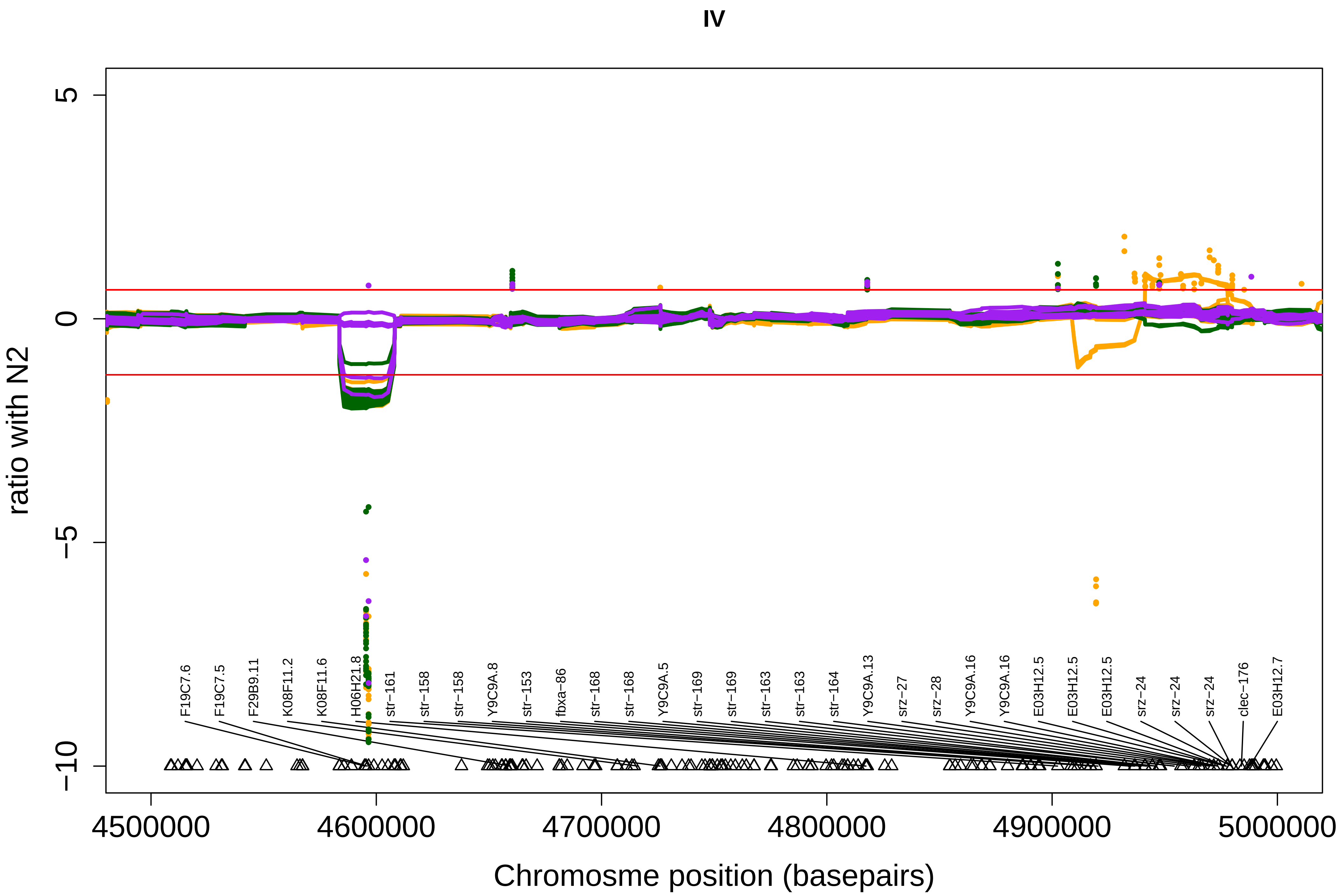
IV
ratio with N2
5
0
−5
−10
4500000
4600000
4700000
4800000
4900000
5000000
Chromosme position (basepairs)
F19C7.6
F19C7.5
F29B9.11
K08F11.2
K08F11.6
H06H21.8
str−161
str−158
str−158
Y9C9A.8
str−153
fbxa−86
str−168
str−168
Y9C9A.5
str−169
str−169
str−163
str−163
str−164
Y9C9A.13
srz−27
srz−28
Y9C9A.16
Y9C9A.16
E03H12.5
E03H12.5
E03H12.5
srz−24
srz−24
srz−24
clec−176
E03H12.7

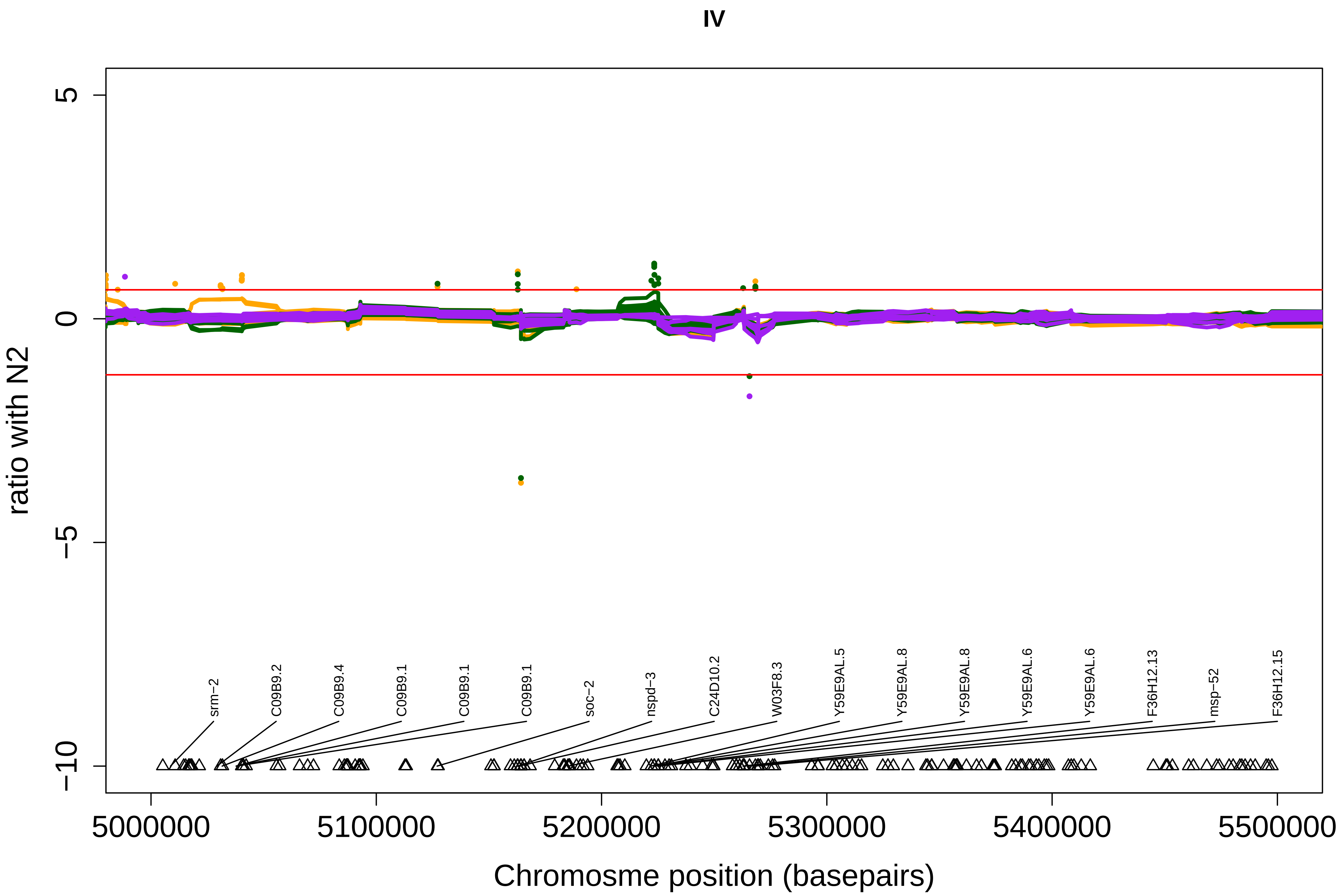
IV
ratio with N2
5
0
−5
−10
srm−2
C09B9.2
C09B9.4
C09B9.1
C09B9.1
C09B9.1
soc−2
nspd−3
C24D10.2
W03F8.3
Y59E9AL.5
Y59E9AL.8
Y59E9AL.8
Y59E9AL.6
Y59E9AL.6
F36H12.13
msp−52
F36H12.15
5000000
5100000
5200000
5300000
5400000
5500000
Chromosme position (basepairs)

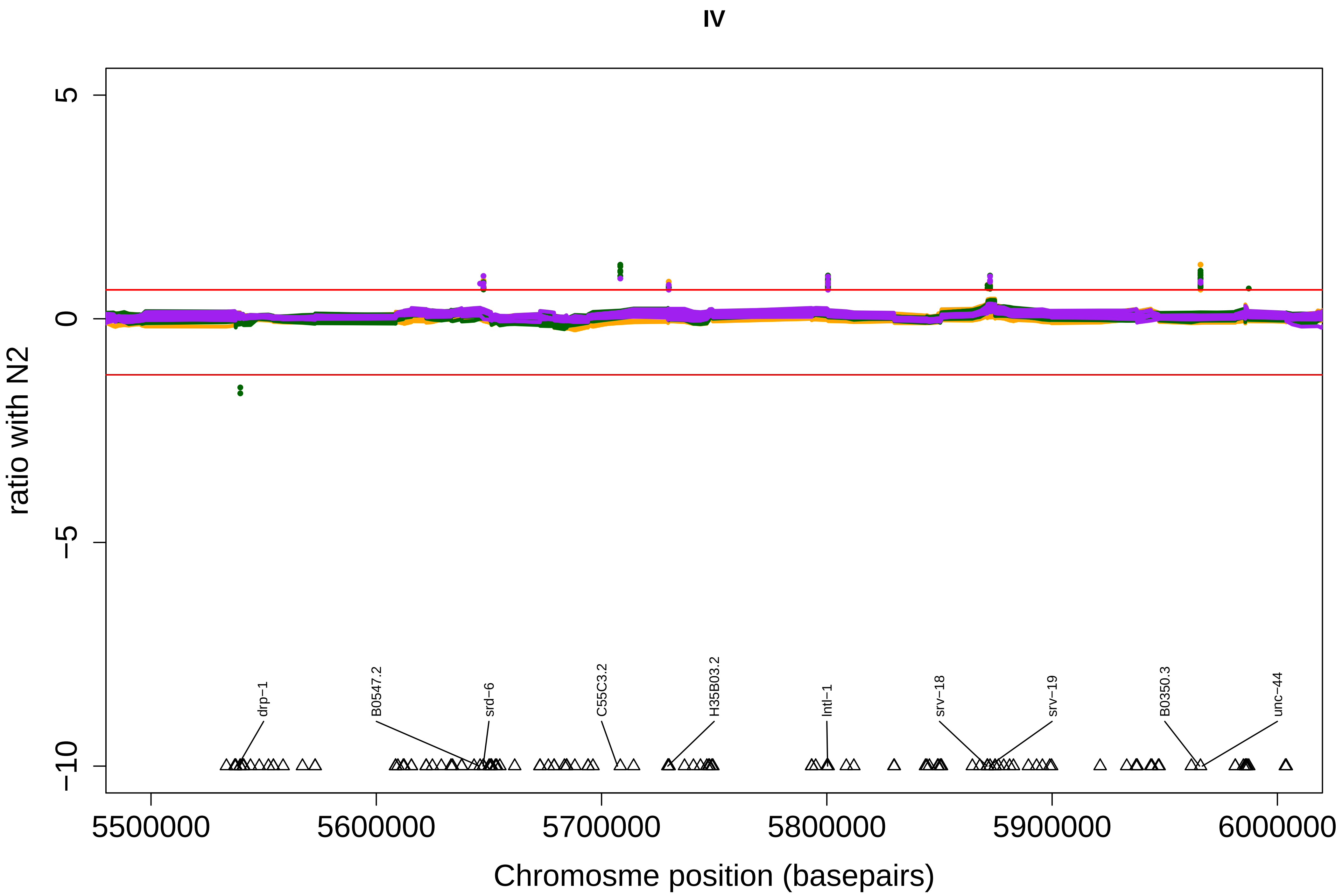

IV
ratio with N2
5
0
−5
−10
5500000
5600000
5700000
5800000
5900000
6000000
Chromosme position (basepairs)
drp−1
B0547.2
srd−6
C55C3.2
H35B03.2
lntl−1
srv−18
srv−19
B0350.3
unc−44

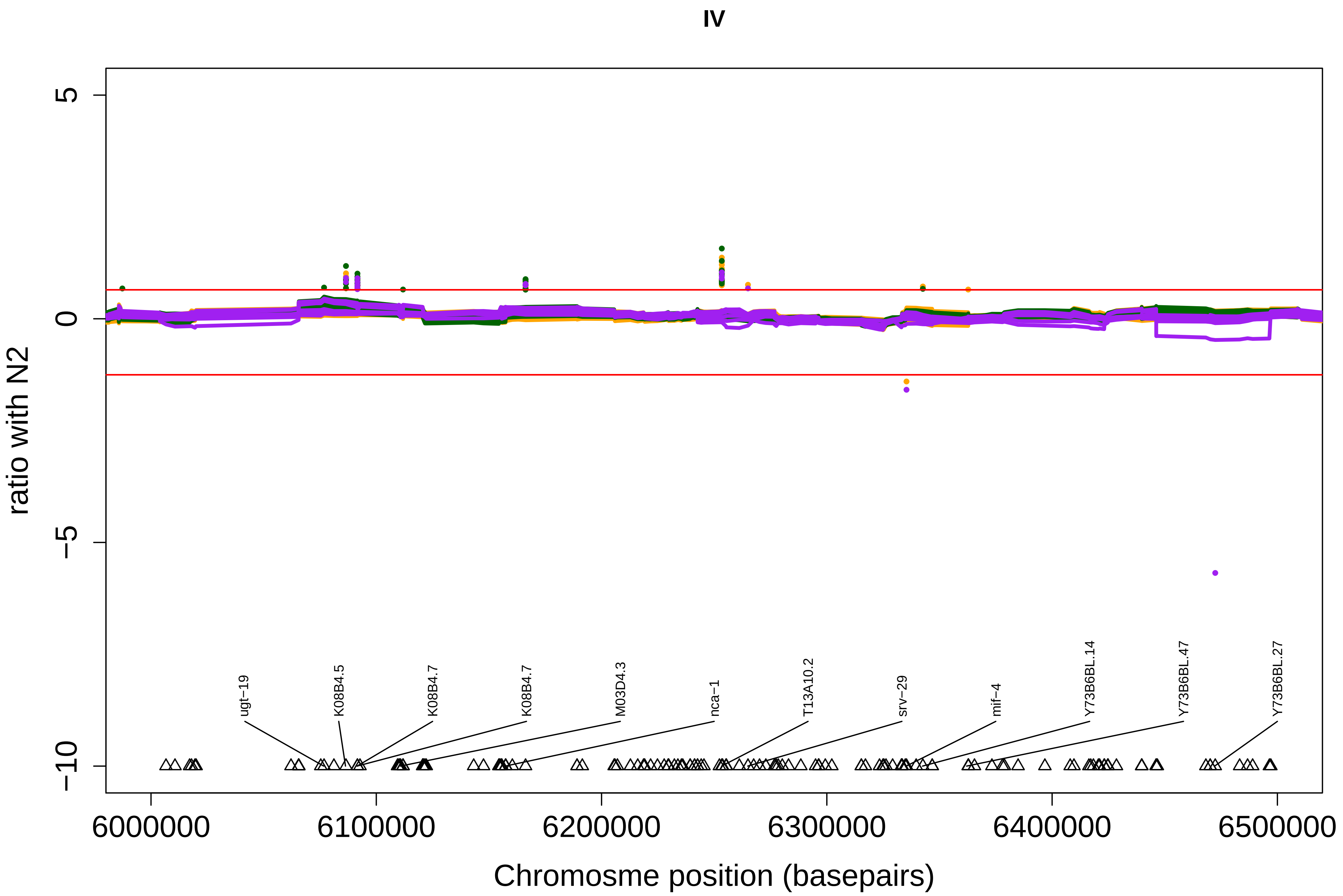
IV
ratio with N2
5
0
−5
−10
ugt−19
K08B4.5
K08B4.7
K08B4.7
M03D4.3
nca−1
T13A10.2
srv−29
mif−4
Y73B6BL.14
Y73B6BL.47
Y73B6BL.27
6000000
6100000
6200000
6300000
6400000
6500000
Chromosme position (basepairs)

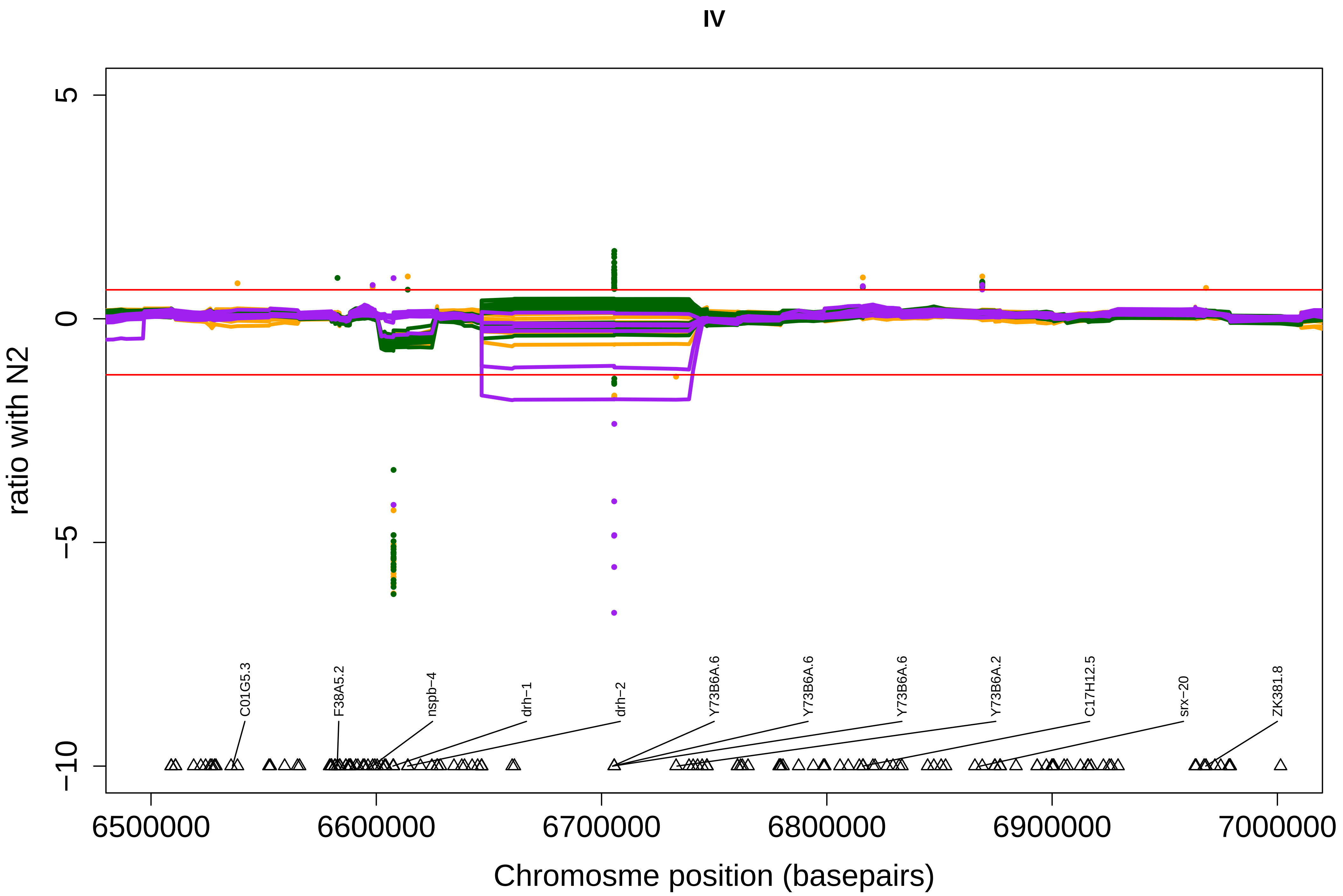
IV
ratio with N2
5
0
−5
−10
C01G5.3
F38A5.2
nspb−4
drh−1
drh−2
Y73B6A.6
Y73B6A.6
Y73B6A.6
Y73B6A.2
C17H12.5
srx−20
ZK381.8
6500000
6600000
6700000
6800000
6900000
7000000
Chromosme position (basepairs)

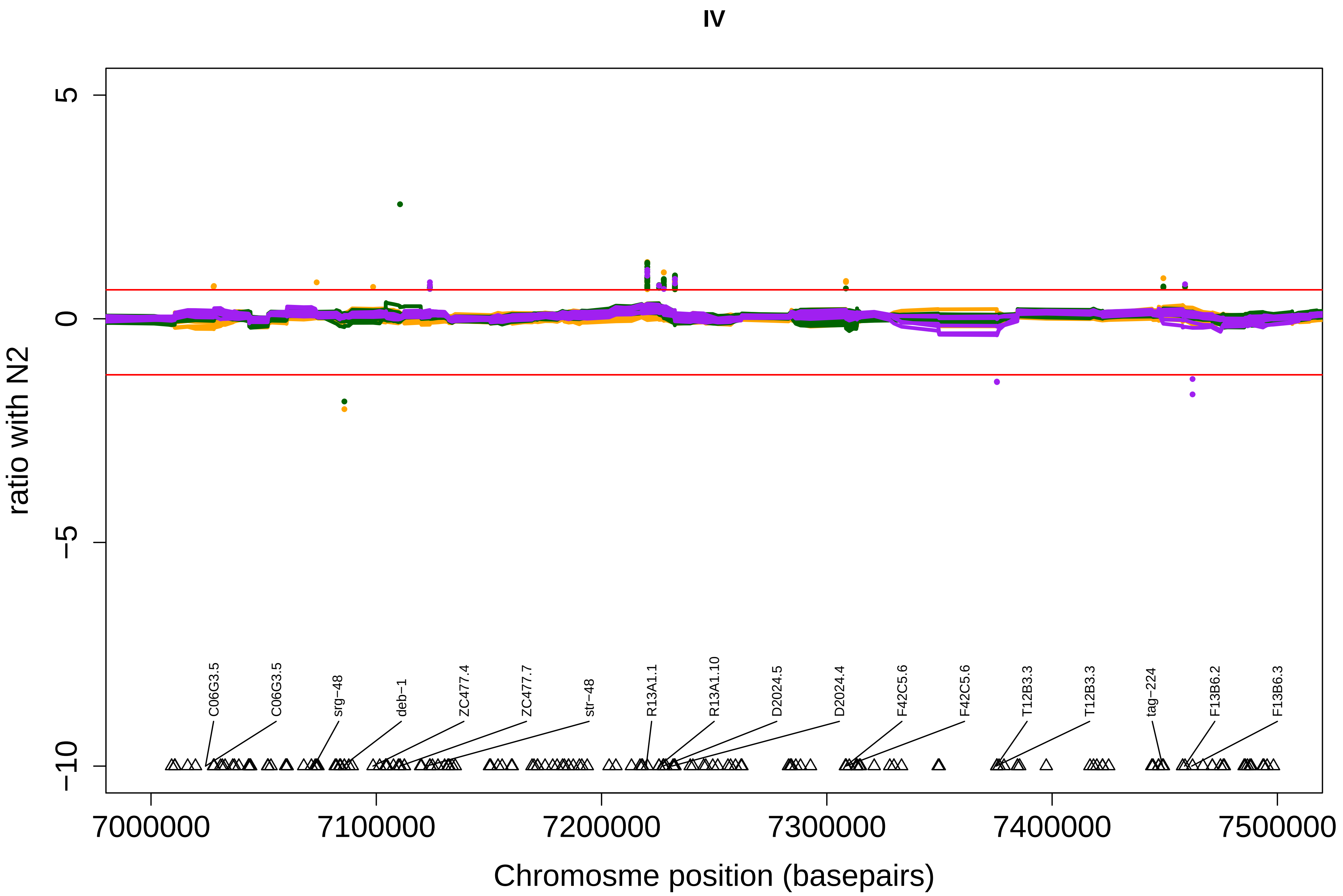
IV
ratio with N2
5
0
−5
−10
C06G3.5
C06G3.5
srg−48
deb−1
ZC477.4
ZC477.7
str−48
R13A1.1
R13A1.10
D2024.5
D2024.4
F42C5.6
F42C5.6
T12B3.3
T12B3.3
tag−224
F13B6.2
F13B6.3
7000000
7100000
7200000
7300000
7400000
7500000
Chromosme position (basepairs)

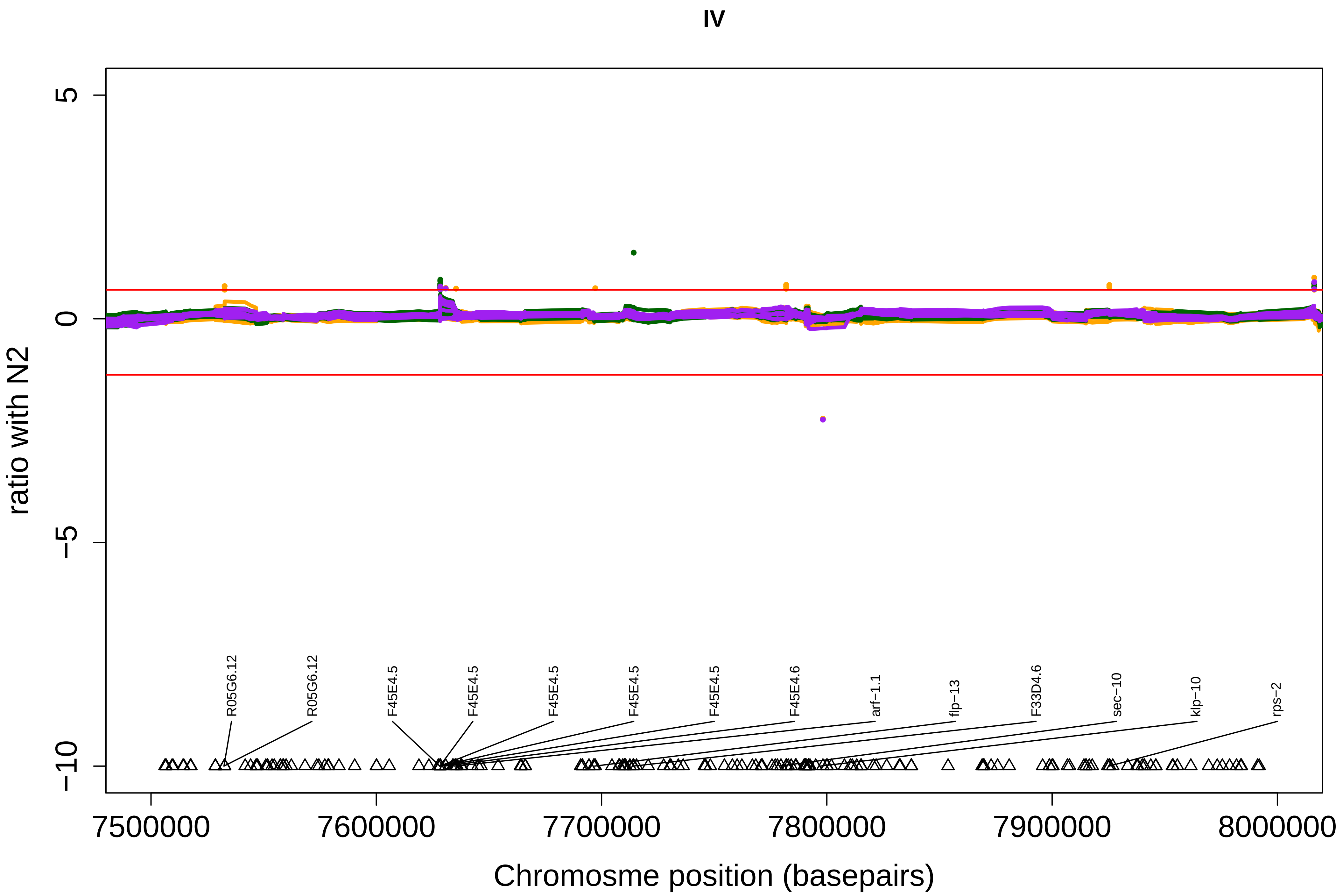
IV
ratio with N2
5
0
−5
−10
R05G6.12
R05G6.12
F45E4.5
F45E4.5
F45E4.5
F45E4.5
F45E4.5
F45E4.6
arf−1.1
flp−13
F33D4.6
sec−10
klp−10
rps−2
7500000
7600000
7700000
7800000
7900000
8000000
Chromosme position (basepairs)

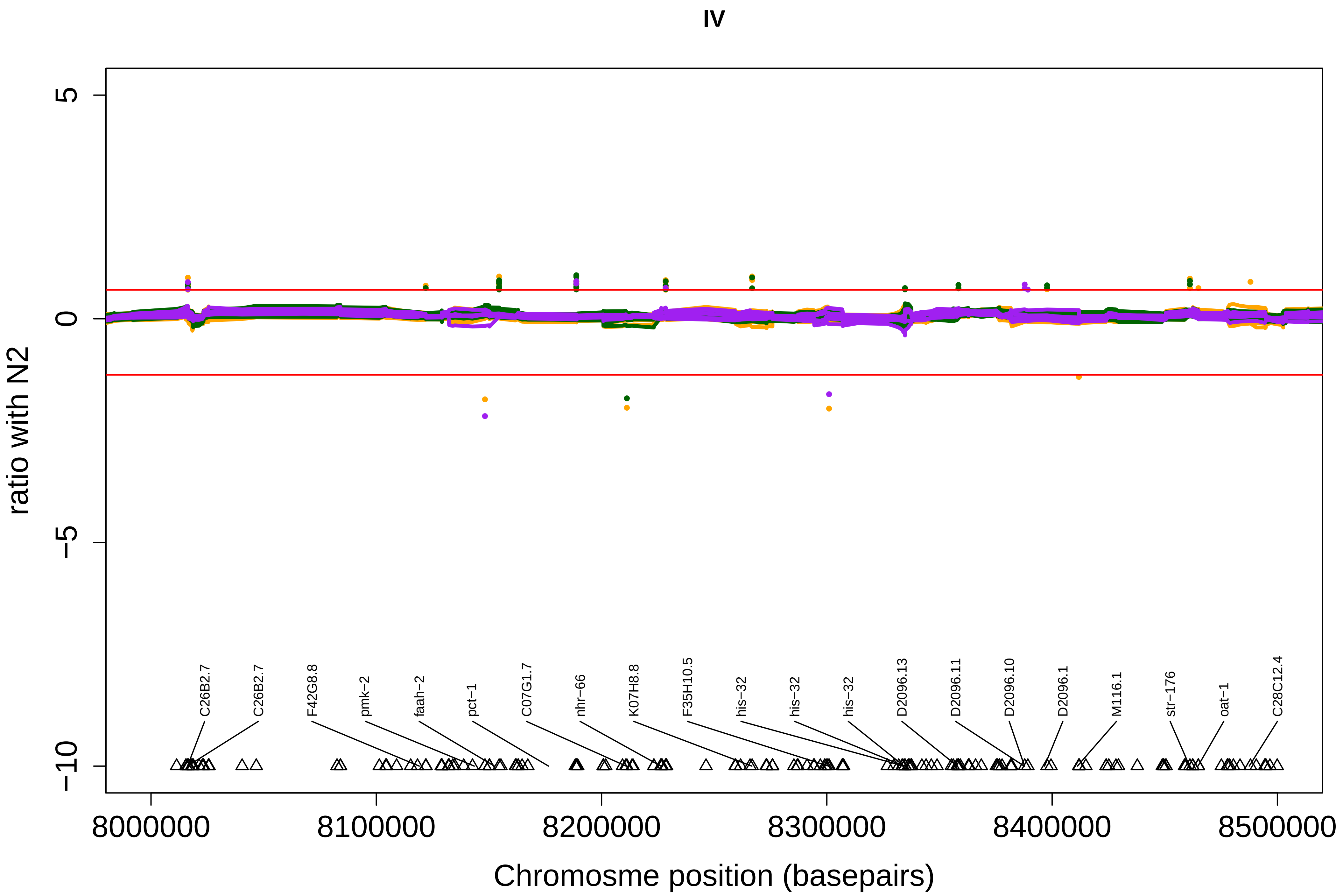

IV
ratio with N2
5
0
−5
−10
C26B2.7
C26B2.7
F42G8.8
pmk−2
faah−2
pct−1
C07G1.7
nhr−66
K07H8.8
F35H10.5
his−32
his−32
his−32
D2096.13
D2096.11
D2096.10
D2096.1
M116.1
str−176
oat−1
C28C12.4
8000000
8100000
8200000
8300000
8400000
8500000
Chromosme position (basepairs)

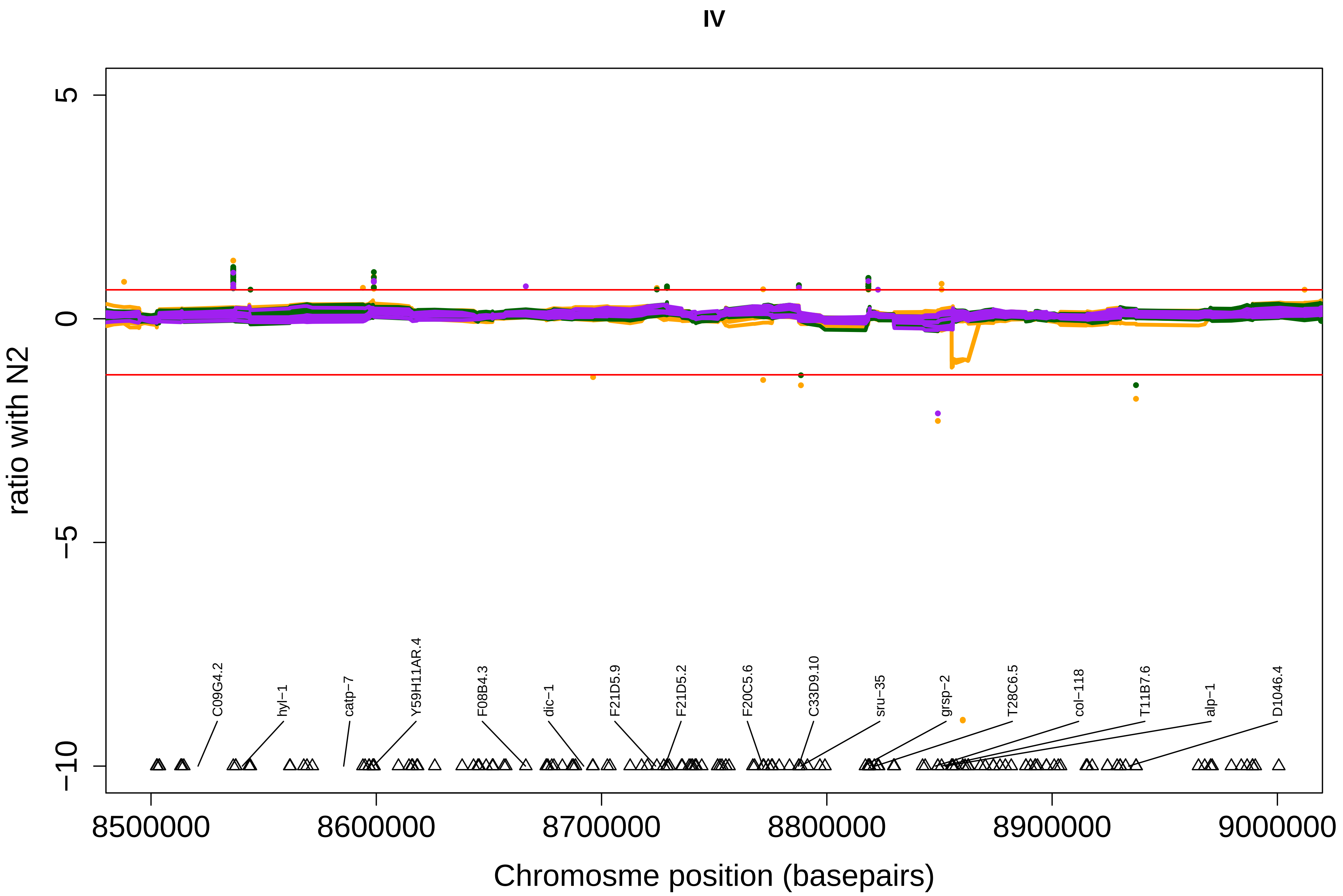
IV
ratio with N2
5
0
−5
−10
C09G4.2
hyl−1
catp−7
Y59H11AR.4
F08B4.3
dic−1
F21D5.9
F21D5.2
F20C5.6
C33D9.10
sru−35
grsp−2
T28C6.5
col−118
T11B7.6
alp−1
D1046.4
8500000
8600000
8700000
8800000
8900000
9000000
Chromosme position (basepairs)

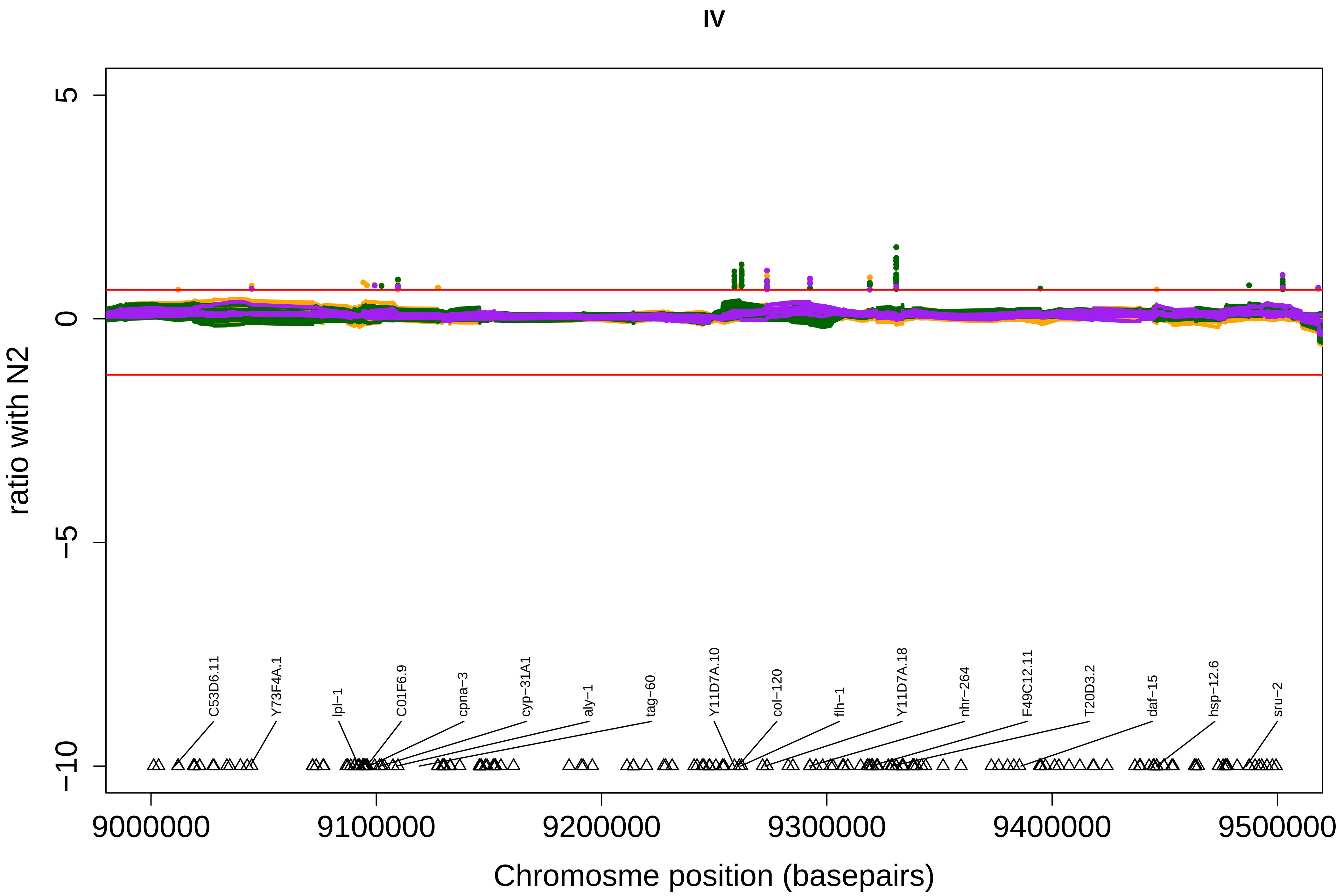

IV
ratio with N2
5
0
−5
−10
C53D6.11
Y73F4A.1
lpl−1
C01F6.9
cpna−3
cyp−31A1
aly−1
tag−60
Y11D7A.10
col−120
flh−1
Y11D7A.18
nhr−264
F49C12.11
T20D3.2
daf−15
hsp−12.6
sru−2
9000000
9100000
9200000
9300000
9400000
9500000
Chromosme position (basepairs)

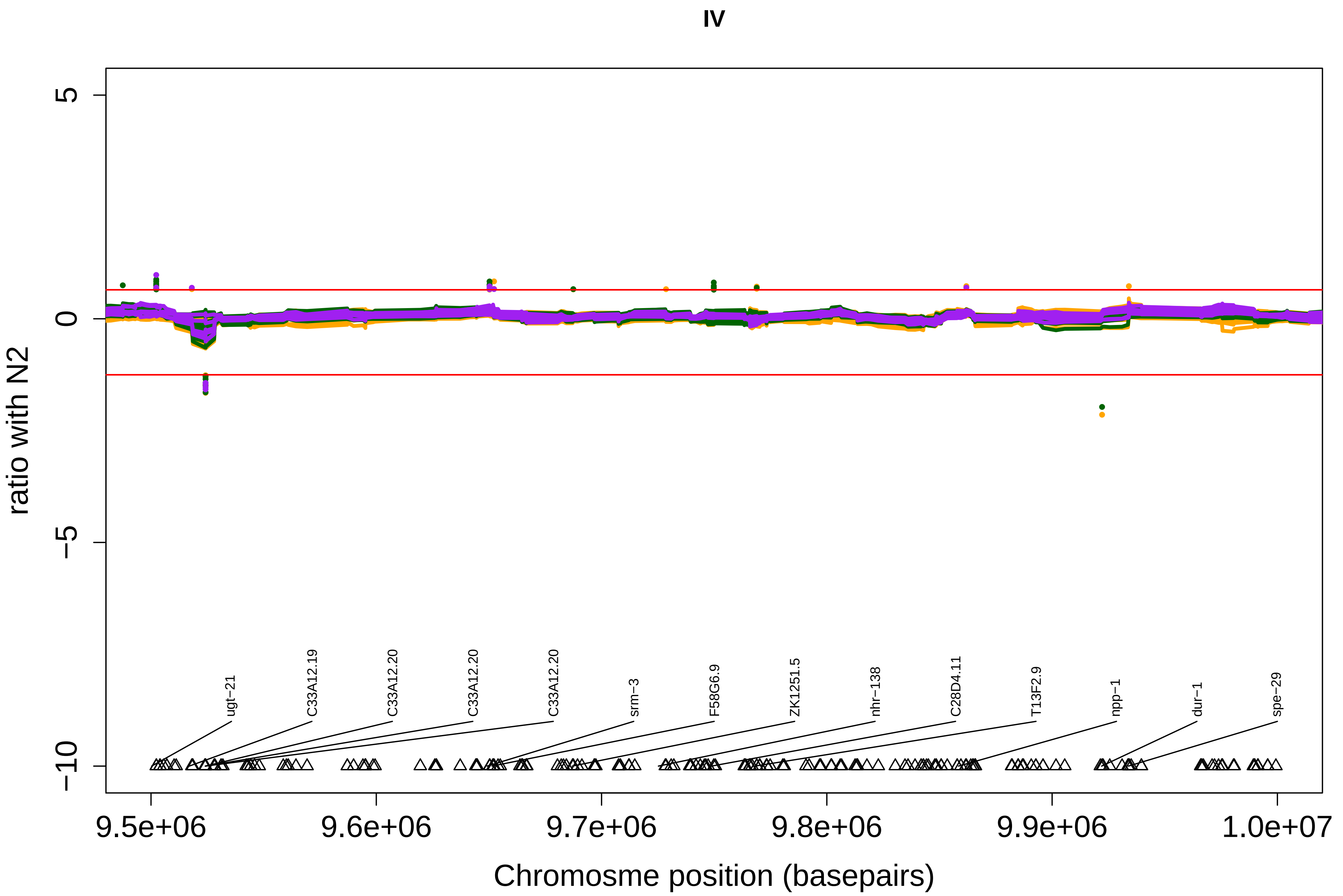

IV
ratio with N2
5
0
−5
−10
9.5e+06
9.6e+06
9.7e+06
9.8e+06
9.9e+06
1.0e+07
Chromosme position (basepairs)
ugt−21
C33A12.19
C33A12.20
C33A12.20
C33A12.20
srm−3
F58G6.9
ZK1251.5
nhr−138
C28D4.11
T13F2.9
npp−1
dur−1
spe−29

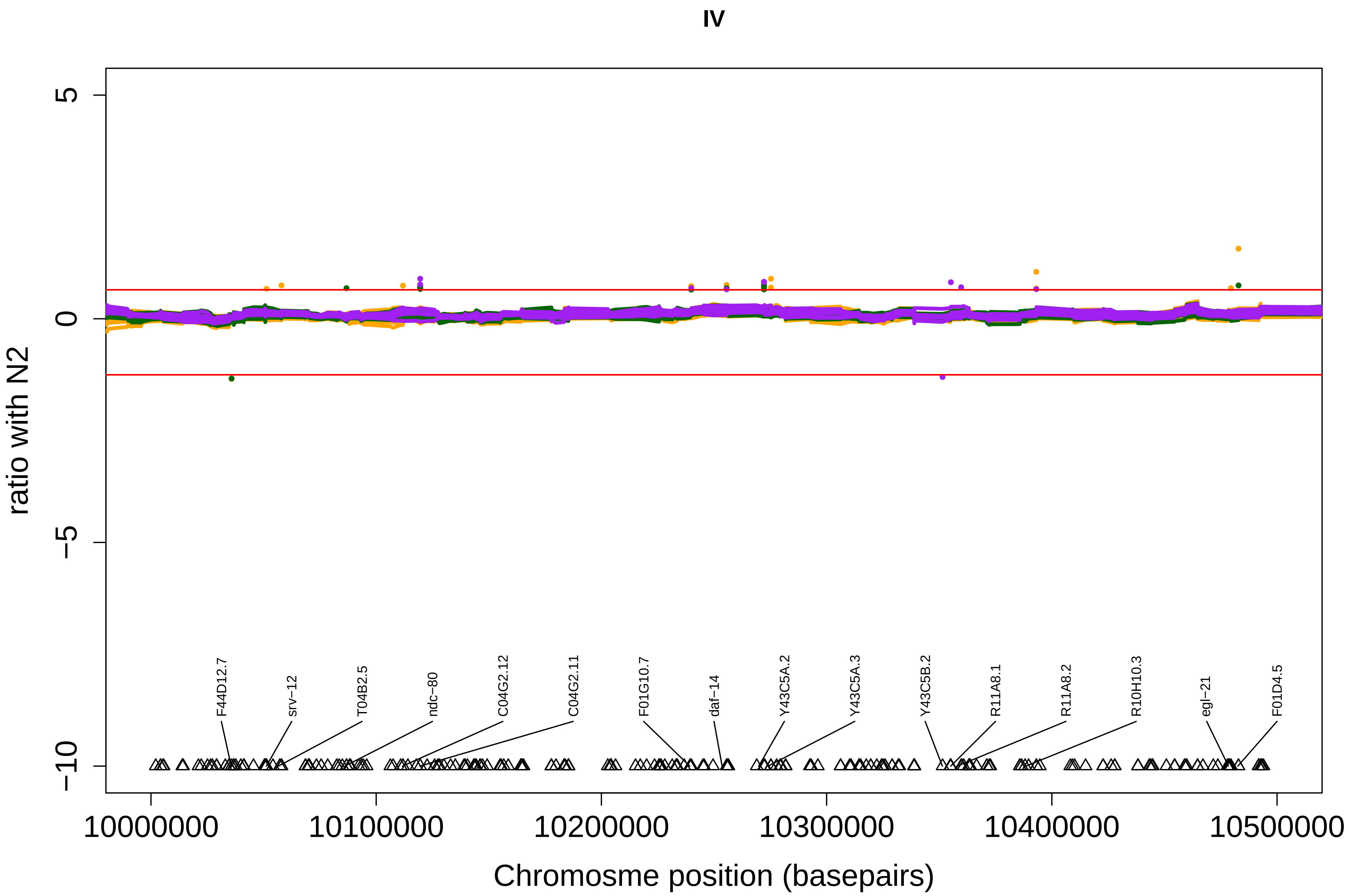
IV
ratio with N2
5
0
−5
−10
F44D12.7
srv−12
T04B2.5
ndc−80
C04G2.12
C04G2.11
F01G10.7
daf−14
Y43C5A.2
Y43C5A.3
Y43C5B.2
R11A8.1
R11A8.2
R10H10.3
egl−21
F01D4.5
10000000
10100000
10200000
10300000
10400000
10500000
Chromosme position (basepairs)

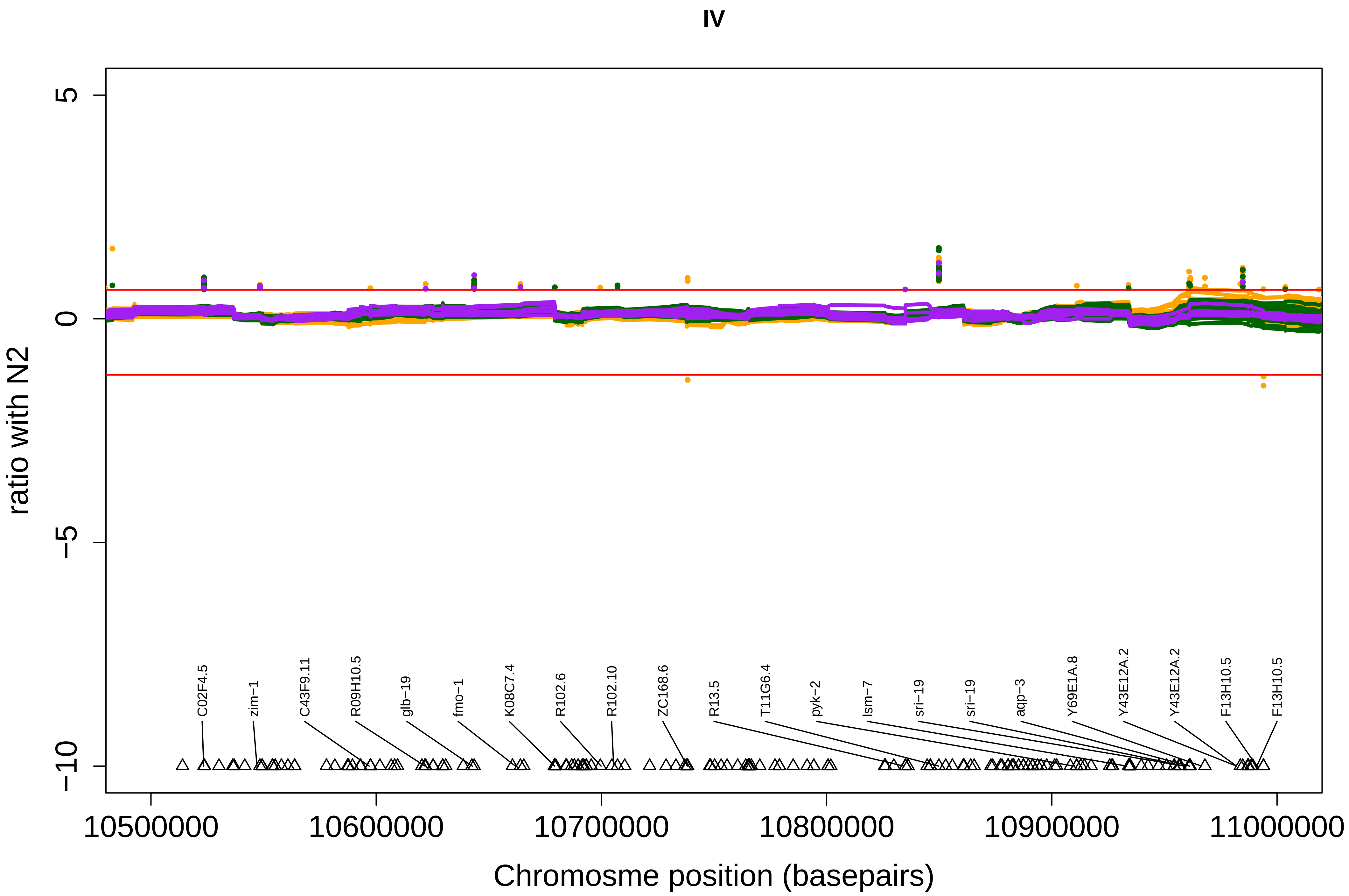
IV
ratio with N2
5
0
−5
−10
10500000
10600000
10700000
10800000
10900000
11000000
Chromosme position (basepairs)
C02F4.5
zim−1
C43F9.11
R09H10.5
glb−19
fmo−1
K08C7.4
R102.6
R102.10
ZC168.6
R13.5
T11G6.4
pyk−2
lsm−7
sri−19
sri−19
aqp−3
Y69E1A.8
Y43E12A.2
Y43E12A.2
F13H10.5
F13H10.5

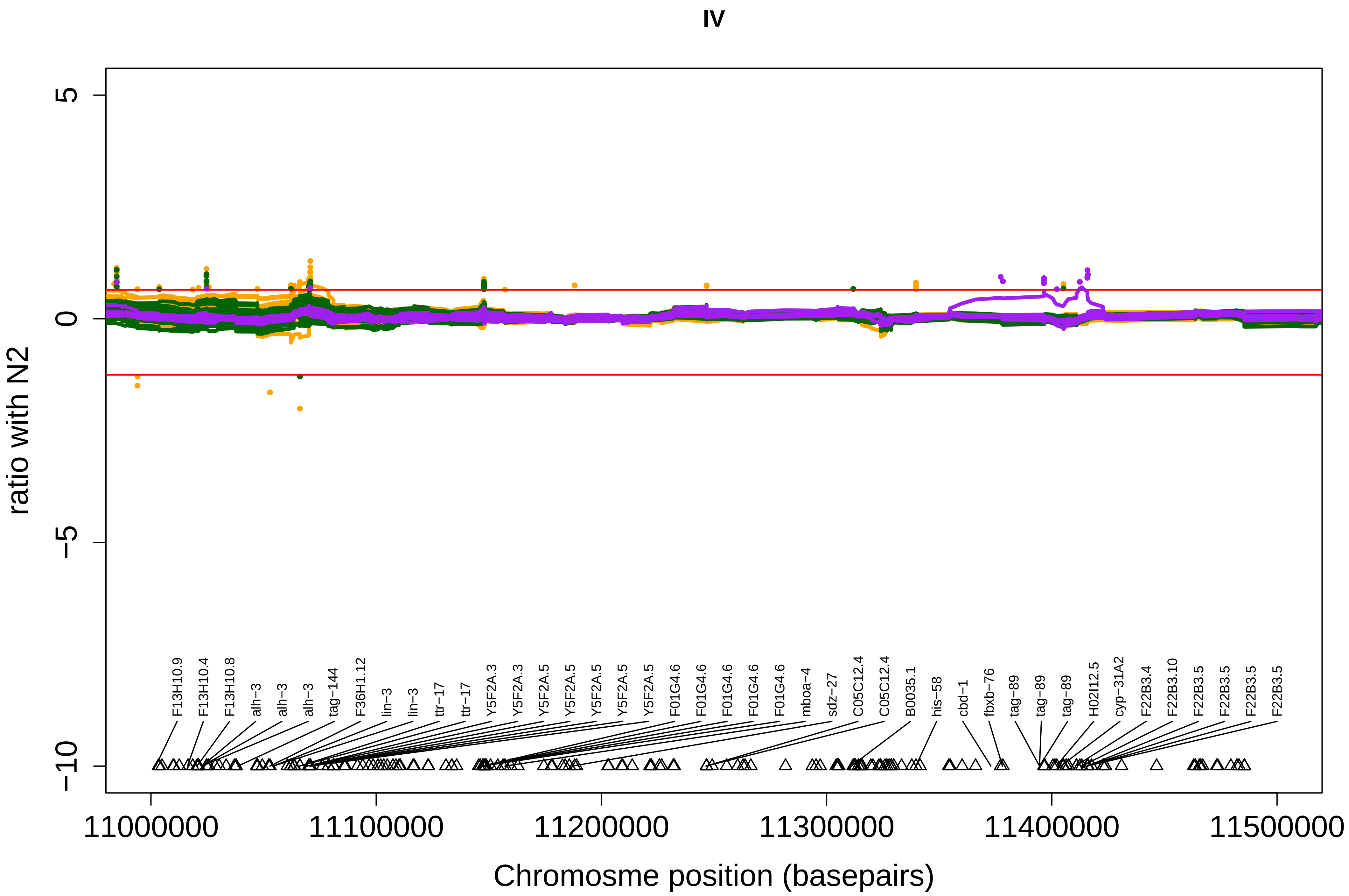
IV
ratio with N2
5
0
−5
−10
11000000
11100000
11200000
11300000
11400000
11500000
Chromosme position (basepairs)
F13H10.9
F13H10.4
F13H10.8
alh−3
alh−3
alh−3
tag−144
F36H1.12
lin−3
lin−3
ttr−17
ttr−17
Y5F2A.3
Y5F2A.3
Y5F2A.5
Y5F2A.5
Y5F2A.5
Y5F2A.5
Y5F2A.5
F01G4.6
F01G4.6
F01G4.6
F01G4.6
F01G4.6
mboa−4
sdz−27
C05C12.4
C05C12.4
B0035.1
his−58
cbd−1
fbxb−76
tag−89
tag−89
tag−89
H02I12.5
cyp−31A2
F22B3.4
F22B3.10
F22B3.5
F22B3.5
F22B3.5
F22B3.5

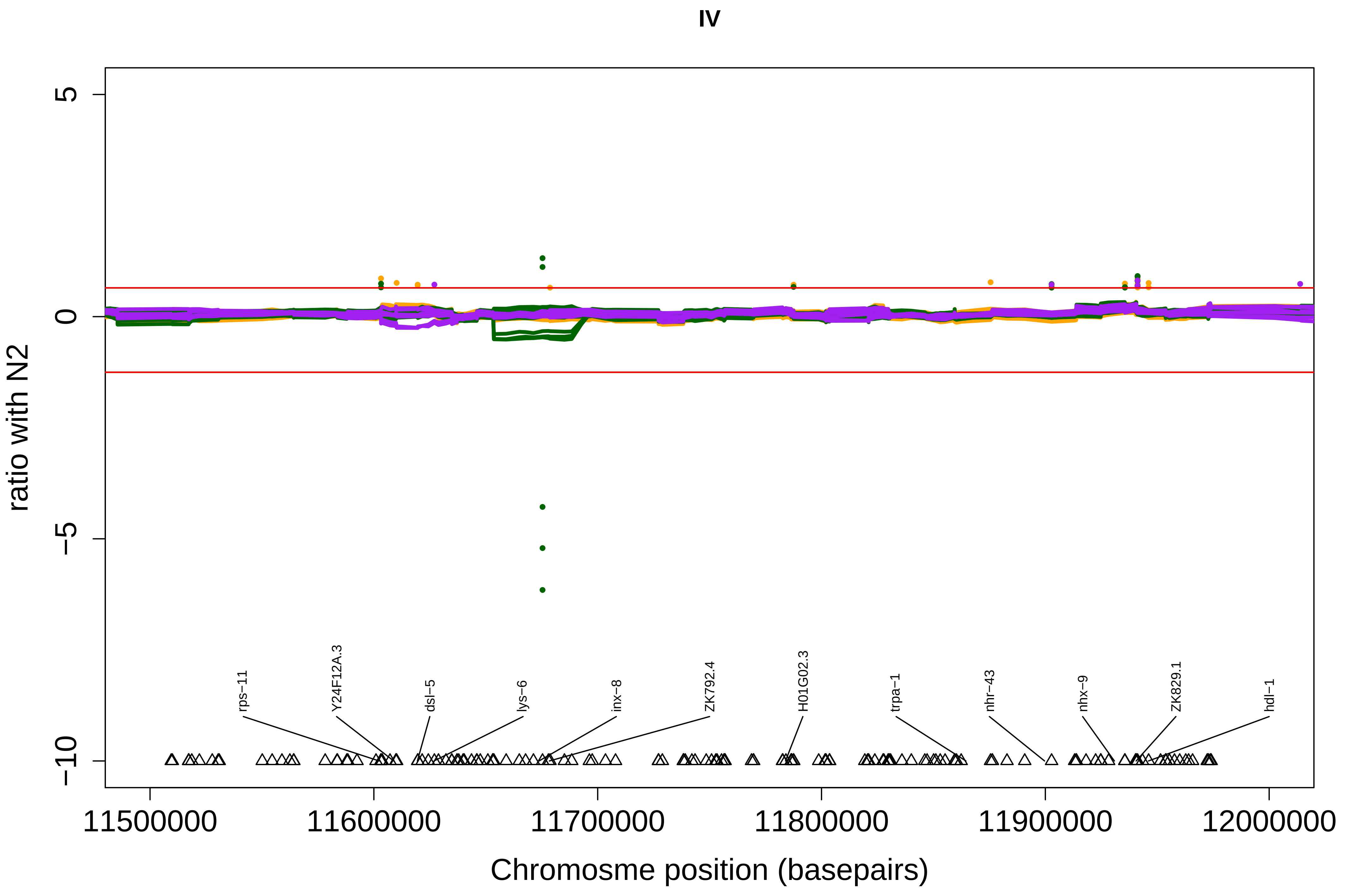

IV
ratio with N2
5
0
−5
−10
rps−11
Y24F12A.3
dsl−5
lys−6
inx−8
ZK792.4
H01G02.3
trpa−1
nhr−43
nhx−9
ZK829.1
hdl−1
11500000
11600000
11700000
11800000
11900000
12000000
Chromosme position (basepairs)

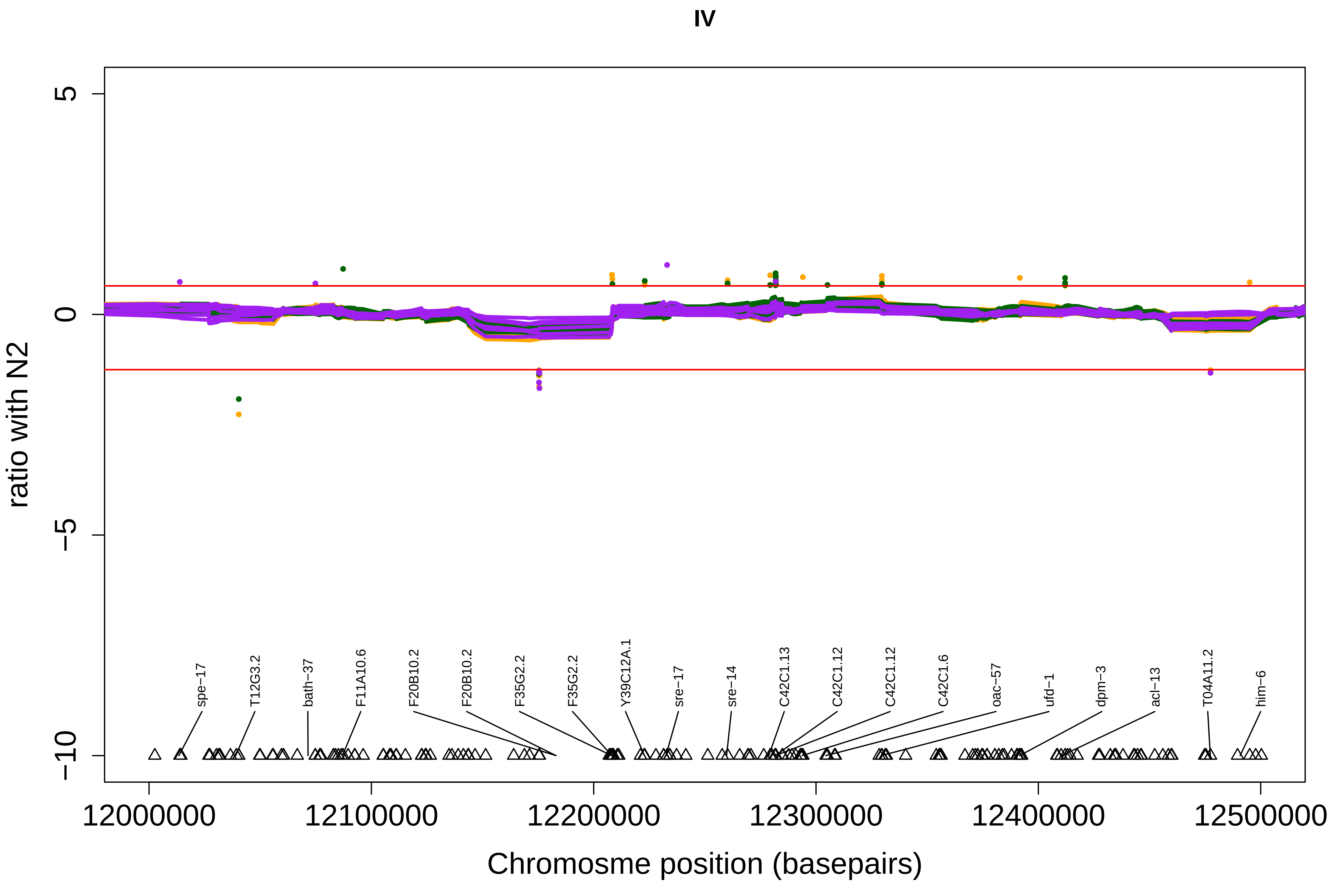
IV
ratio with N2
5
0
−5
−10
spe−17
T12G3.2
bath−37
F11A10.6
F20B10.2
F20B10.2
F35G2.2
F35G2.2
Y39C12A.1
sre−17
sre−14
C42C1.13
C42C1.12
C42C1.12
C42C1.6
oac−57
ufd−1
dpm−3
acl−13
T04A11.2
him−6
12000000
12100000
12200000
12300000
12400000
12500000
Chromosme position (basepairs)

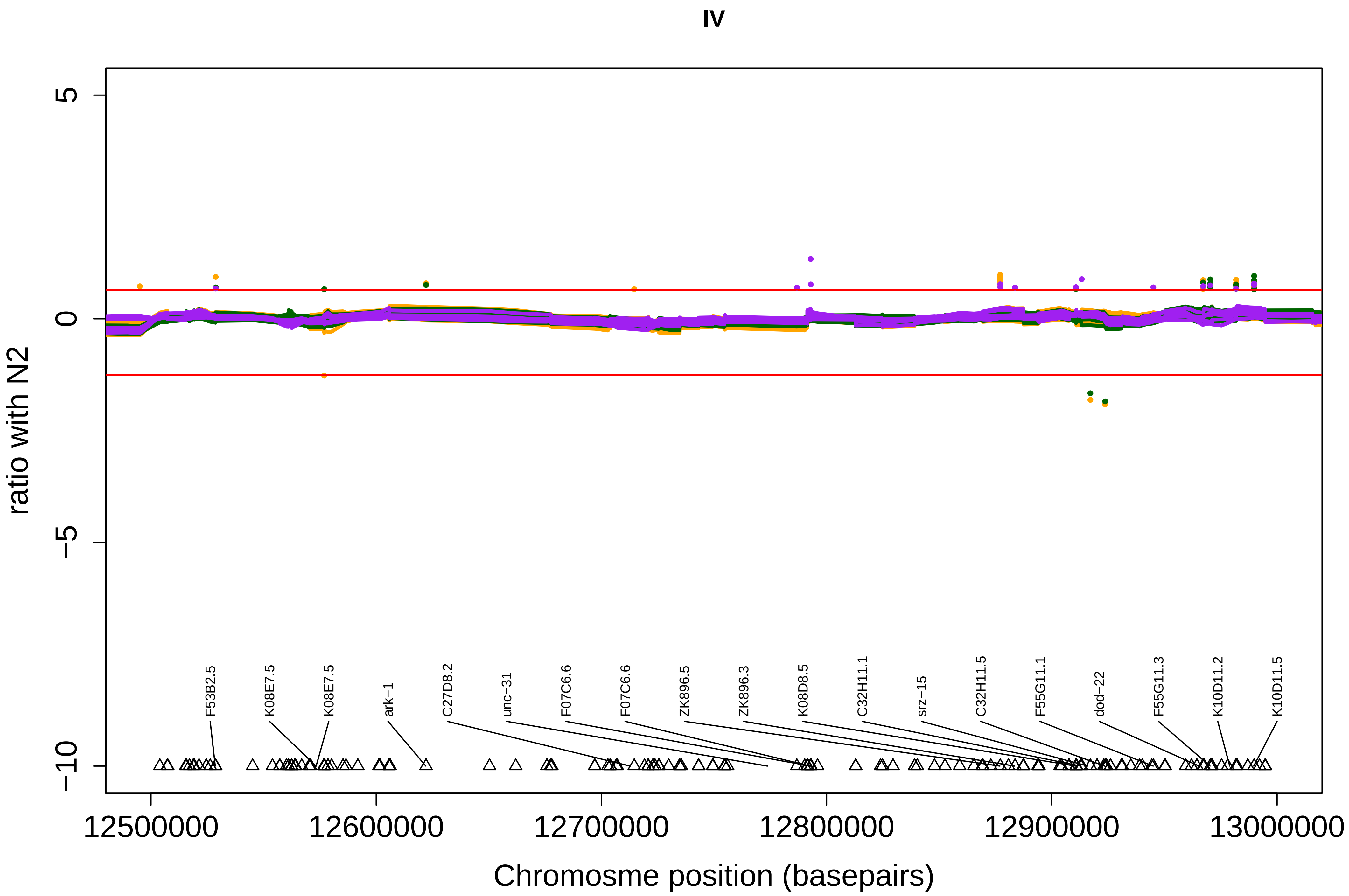
IV
ratio with N2
5
0
−5
−10
12500000
12600000
12700000
12800000
12900000
13000000
Chromosme position (basepairs)
F53B2.5
K08E7.5
K08E7.5
ark−1
C27D8.2
unc−31
F07C6.6
F07C6.6
ZK896.5
ZK896.3
K08D8.5
C32H11.1
srz−15
C32H11.5
F55G11.1
dod−22
F55G11.3
K10D11.2
K10D11.5

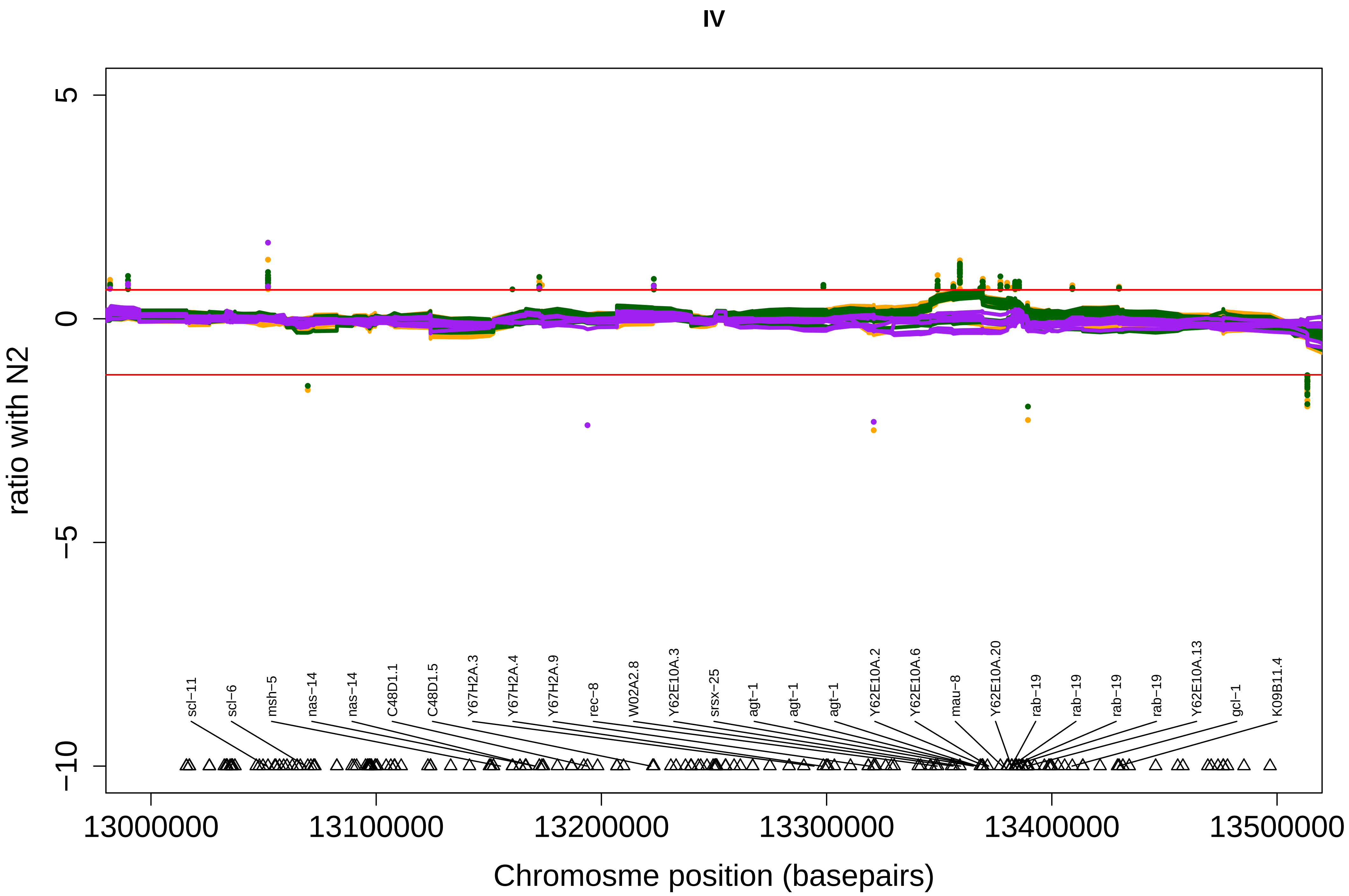

IV
ratio with N2
5
0
−5
−10
Chromosme position (basepairs)
13000000
13100000
13200000
13300000
13400000
13500000
scl−11
scl−6
msh−5
nas−14
nas−14
C48D1.1
C48D1.5
Y67H2A.3
Y67H2A.4
Y67H2A.9
rec−8
W02A2.8
Y62E10A.3
srsx−25
agt−1
agt−1
agt−1
Y62E10A.2
Y62E10A.6
mau−8
Y62E10A.20
rab−19
rab−19
rab−19
rab−19
Y62E10A.13
gcl−1
K09B11.4

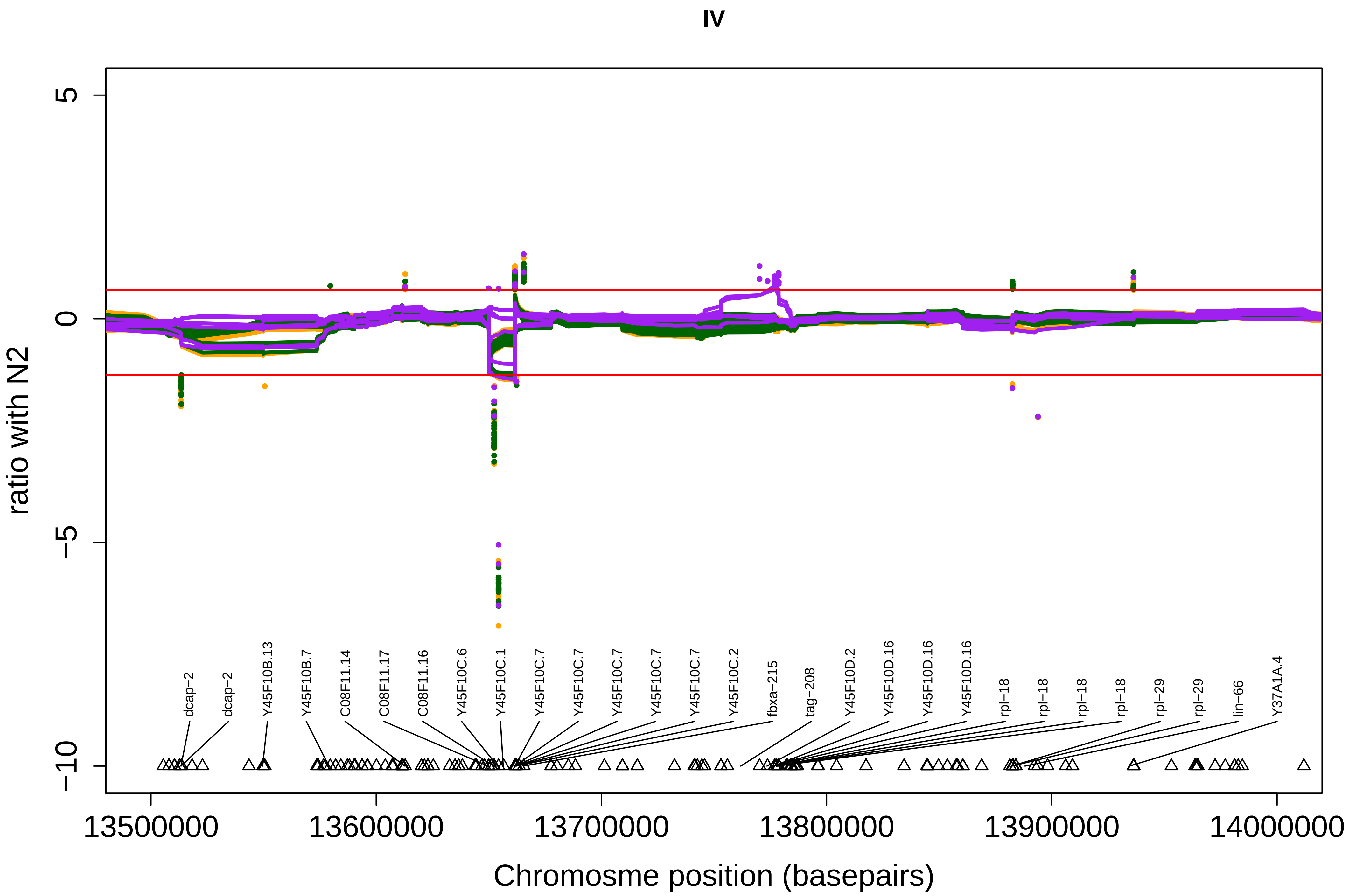

IV
ratio with N2
5
0
−5
−10
13500000
13600000
13700000
13800000
13900000
14000000
Chromosme position (basepairs)
dcap−2
dcap−2
Y45F10B.13
Y45F10B.7
C08F11.14
C08F11.17
C08F11.16
Y45F10C.6
Y45F10C.1
Y45F10C.7
Y45F10C.7
Y45F10C.7
Y45F10C.7
Y45F10C.7
Y45F10C.2
fbxa−215
tag−208
Y45F10D.2
Y45F10D.16
Y45F10D.16
Y45F10D.16
rpl−18
rpl−18
rpl−18
rpl−18
rpl−29
rpl−29
lin−66
Y37A1A.4

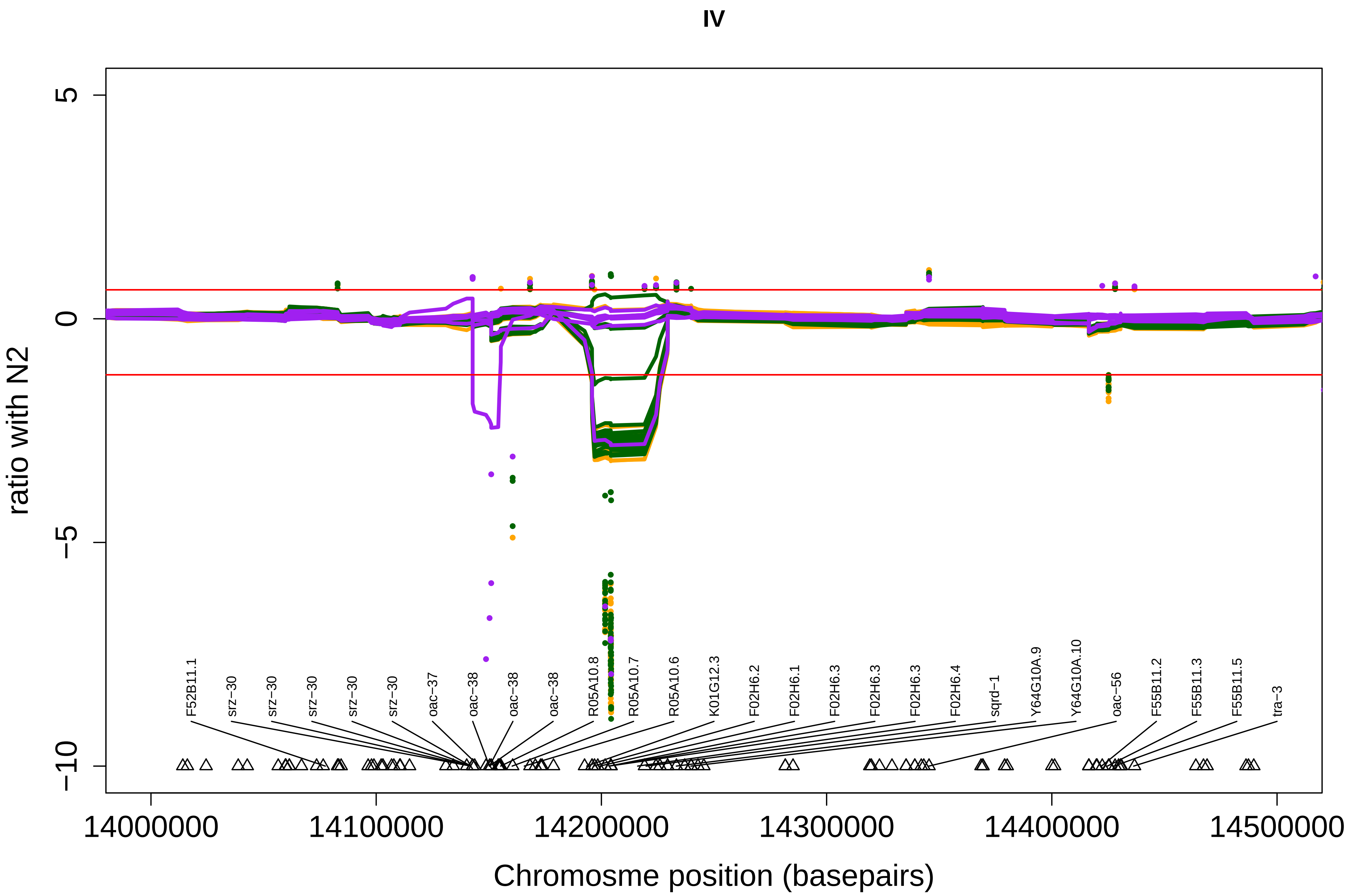
IV
ratio with N2
5
0
−5
−10
F52B11.1
srz−30
srz−30
srz−30
srz−30
srz−30
oac−37
oac−38
oac−38
oac−38
R05A10.8
R05A10.7
R05A10.6
K01G12.3
F02H6.2
F02H6.1
F02H6.3
F02H6.3
F02H6.3
F02H6.4
sqrd−1
Y64G10A.9
Y64G10A.10
oac−56
F55B11.2
F55B11.3
F55B11.5
tra−3
14000000
14100000
14200000
14300000
14400000
14500000
Chromosme position (basepairs)

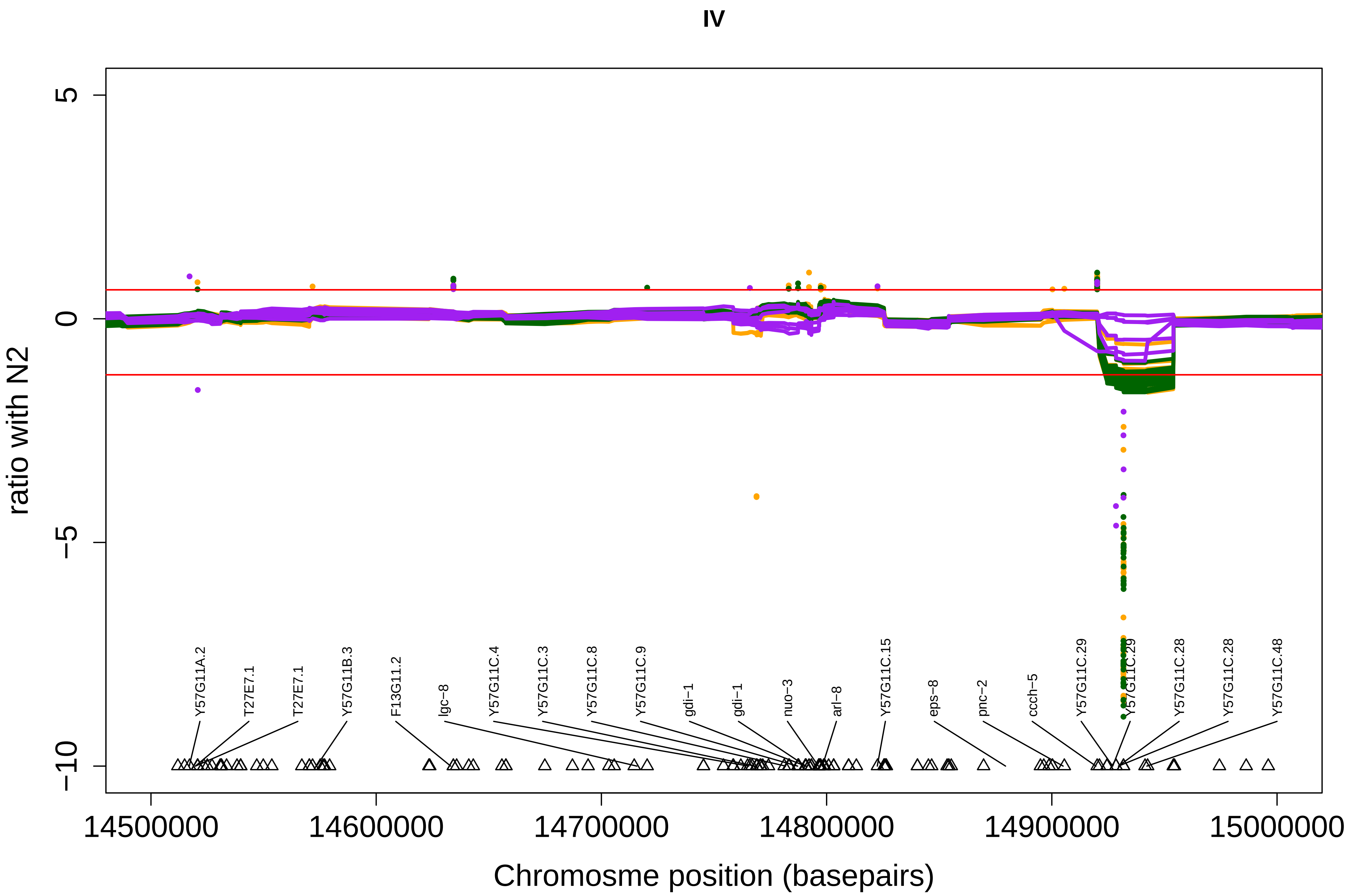
IV
ratio with N2
5
0
−5
−10
Y57G11A.2
T27E7.1
T27E7.1
Y57G11B.3
F13G11.2
lgc−8
Y57G11C.4
Y57G11C.3
Y57G11C.8
Y57G11C.9
gdi−1
gdi−1
nuo−3
arl−8
Y57G11C.15
eps−8
pnc−2
ccch−5
Y57G11C.29
Y57G11C.29
Y57G11C.28
Y57G11C.28
Y57G11C.48
14500000
14600000
14700000
14800000
14900000
15000000
Chromosme position (basepairs)

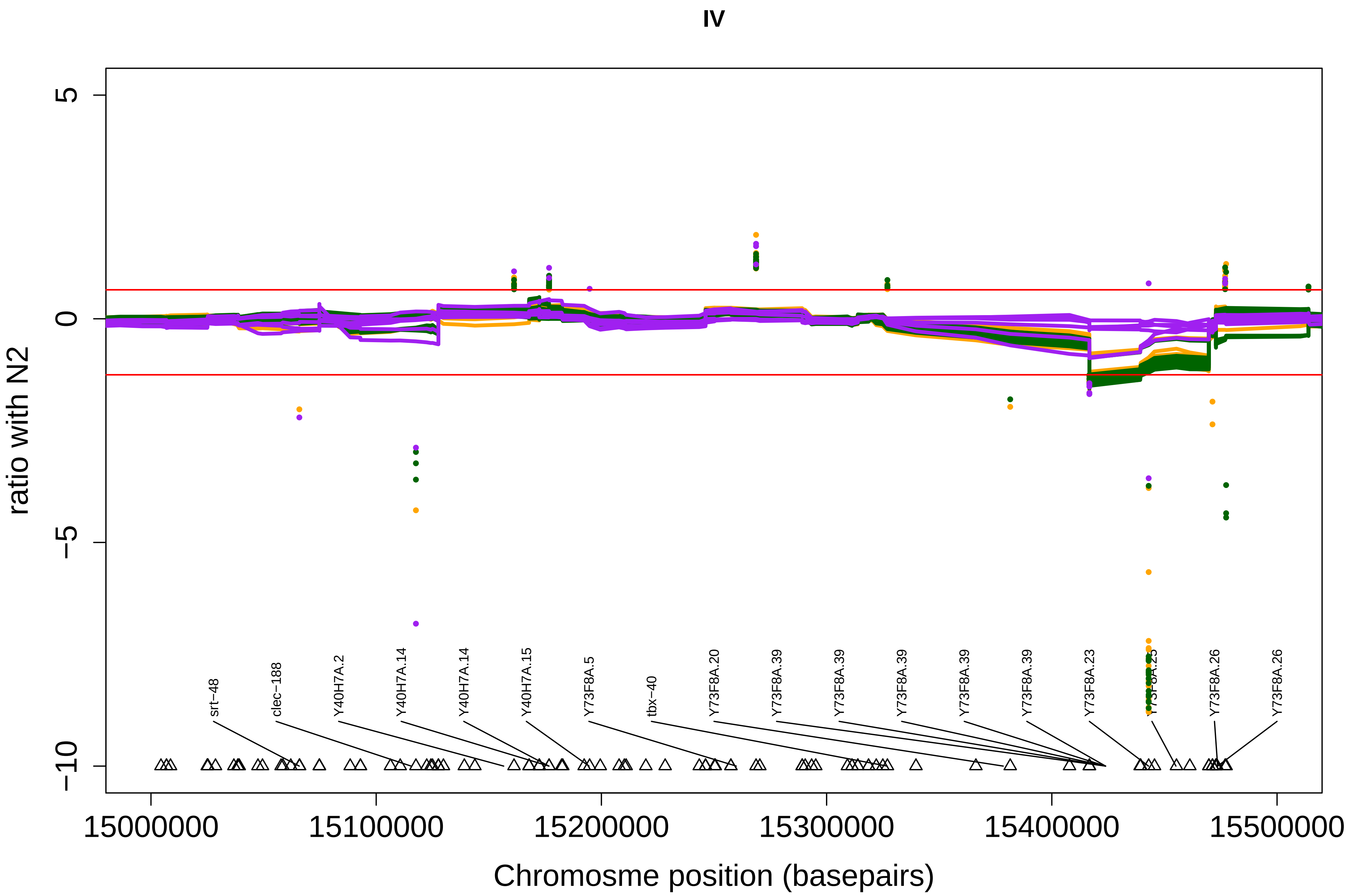
IV
5
0
−5
−10
ratio with N2
15000000
15100000
15200000
15300000
15400000
15500000
Chromosme position (basepairs)
srt−48
clec−188
Y40H7A.2
Y40H7A.14
Y40H7A.14
Y40H7A.15
Y73F8A.5
tbx−40
Y73F8A.20
Y73F8A.39
Y73F8A.39
Y73F8A.39
Y73F8A.39
Y73F8A.39
Y73F8A.23
Y73F8A.26
Y73F8A.26

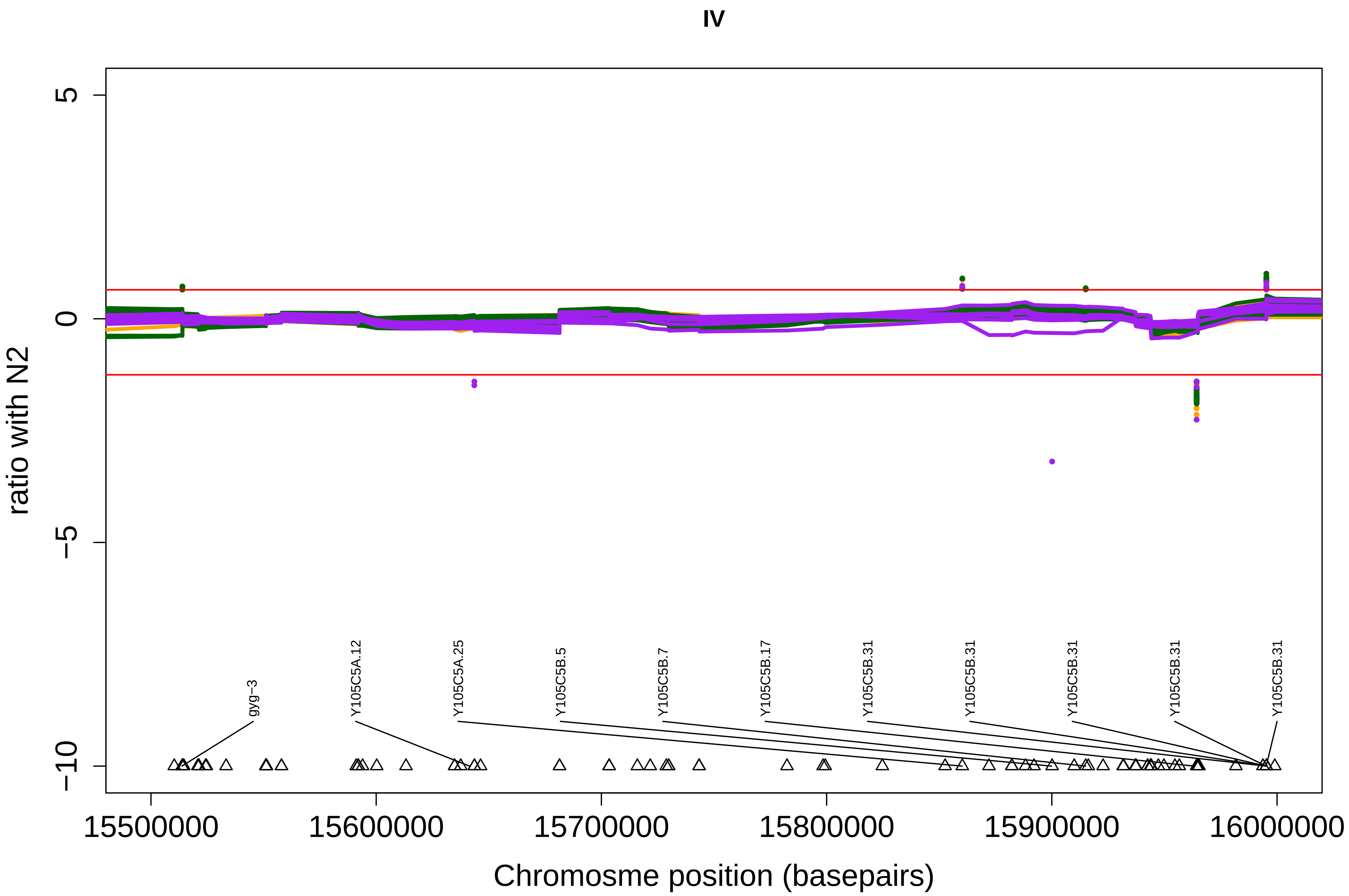
IV
ratio with N2
5
0
−5
−10
gyg−3
Y105C5A.12
Y105C5A.25
Y105C5B.5
Y105C5B.7
Y105C5B.17
Y105C5B.31
Y105C5B.31
Y105C5B.31
Y105C5B.31
Y105C5B.31
15500000
15600000
15700000
15800000
15900000
16000000
Chromosme position (basepairs)

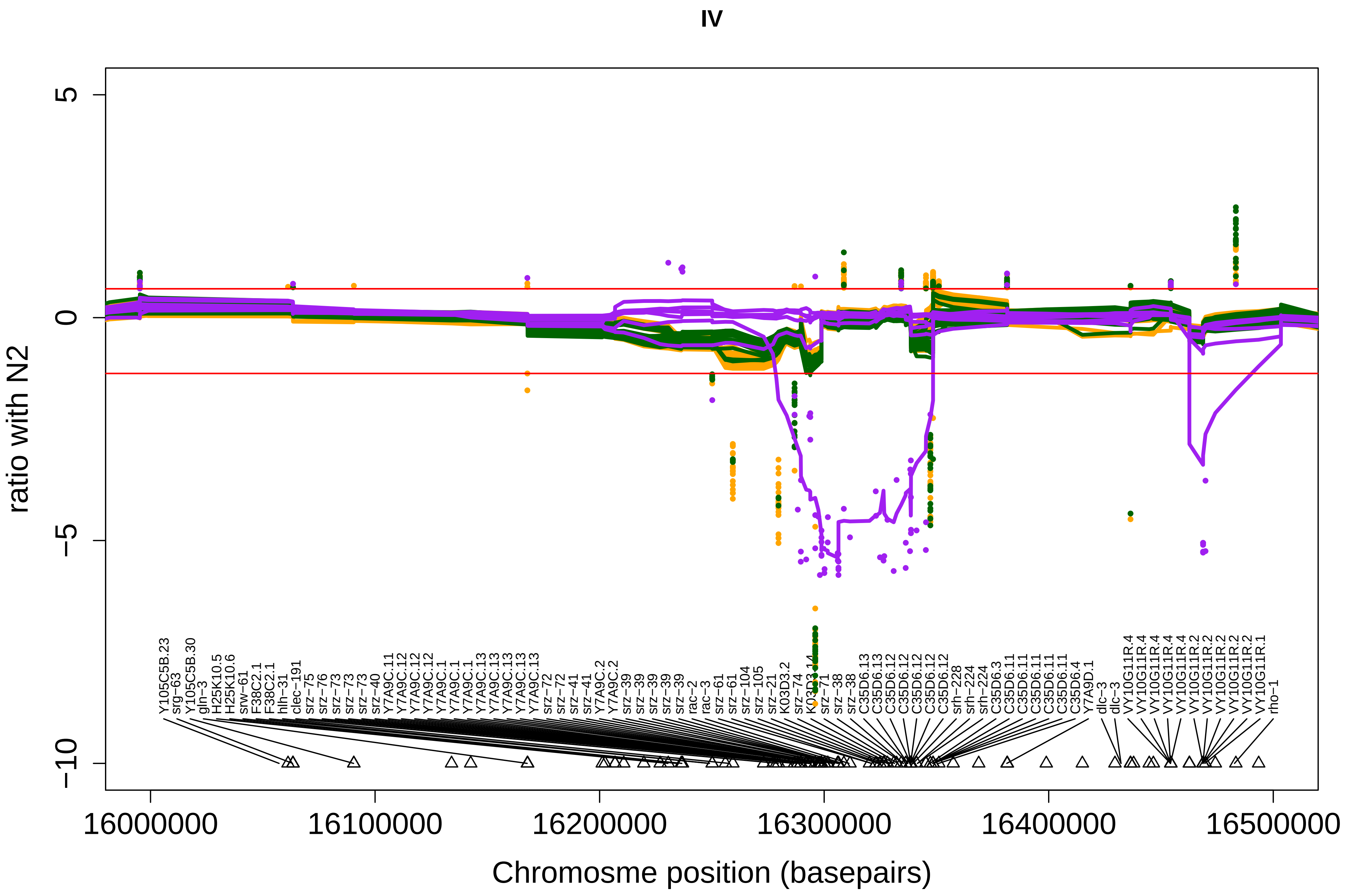
IV
ratio with N2
5
0
−5
−10
16000000
16100000
16200000
16300000
16400000
16500000
Chromosme position (basepairs)
Y105C5B.23
srg−63
Y105C5B.30
gln−3
H25K10.5
H25K10.6
srw−61
F38C2.1
F38C2.1
hlh−31
clec−191
srz−75
srz−76
srz−73
srz−73
srz−40
Y7A9C.11
Y7A9C.12
Y7A9C.12
Y7A9C.1
Y7A9C.1
Y7A9C.1
Y7A9C.13
Y7A9C.13
Y7A9C.13
Y7A9C.13
srz−72
srz−72
srz−41
srz−41
Y7A9C.2
Y7A9C.2
srz−39
srz−39
srz−39
srz−39
srz−39
rac−2
rac−3
srz−61
srz−61
srz−104
srz−105
srz−21
K03D3.2
srz−74
srz−71
srz−38
srz−38
C35D6.13
C35D6.13
C35D6.12
C35D6.12
C35D6.12
C35D6.12
srh−228
srh−224
srh−224
C35D6.3
C35D6.11
C35D6.11
C35D6.11
C35D6.11
C35D6.4
Y7A9D.1
dlc−3
dlc−3
VY10G11R.4
VY10G11R.4
VY10G11R.4
VY10G11R.4
VY10G11R.4
VY10G11R.2
VY10G11R.2
VY10G11R.2
VY10G11R.2
VY10G11R.2
VY10G11R.1
rho−1

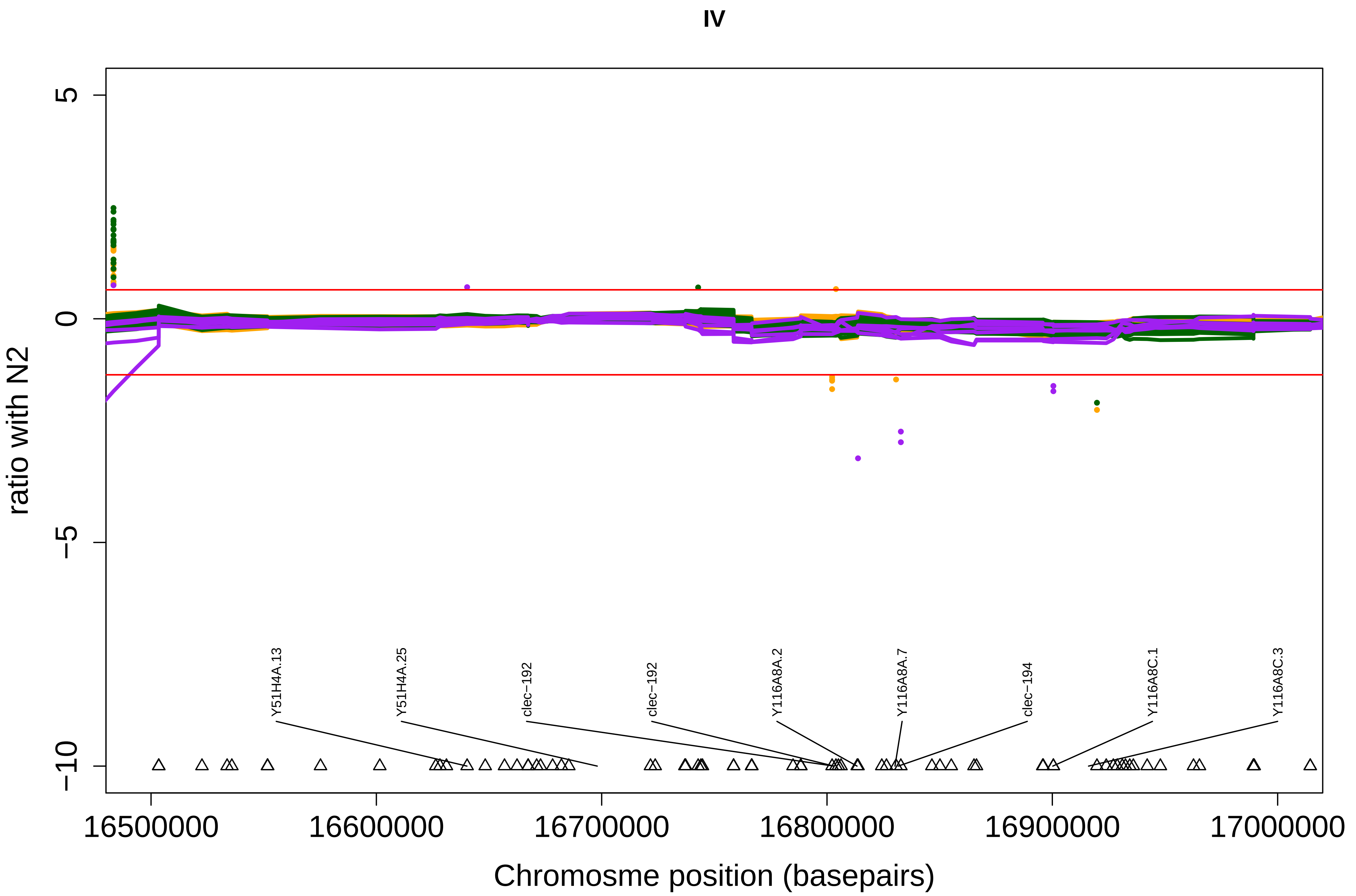

IV
ratio with N2
5
0
−5
−10
Y51H4A.13
Y51H4A.25
clec−192
clec−192
Y116A8A.2
Y116A8A.7
clec−194
Y116A8C.1
Y116A8C.3
16500000
16600000
16700000
16800000
16900000
17000000
Chromosme position (basepairs)

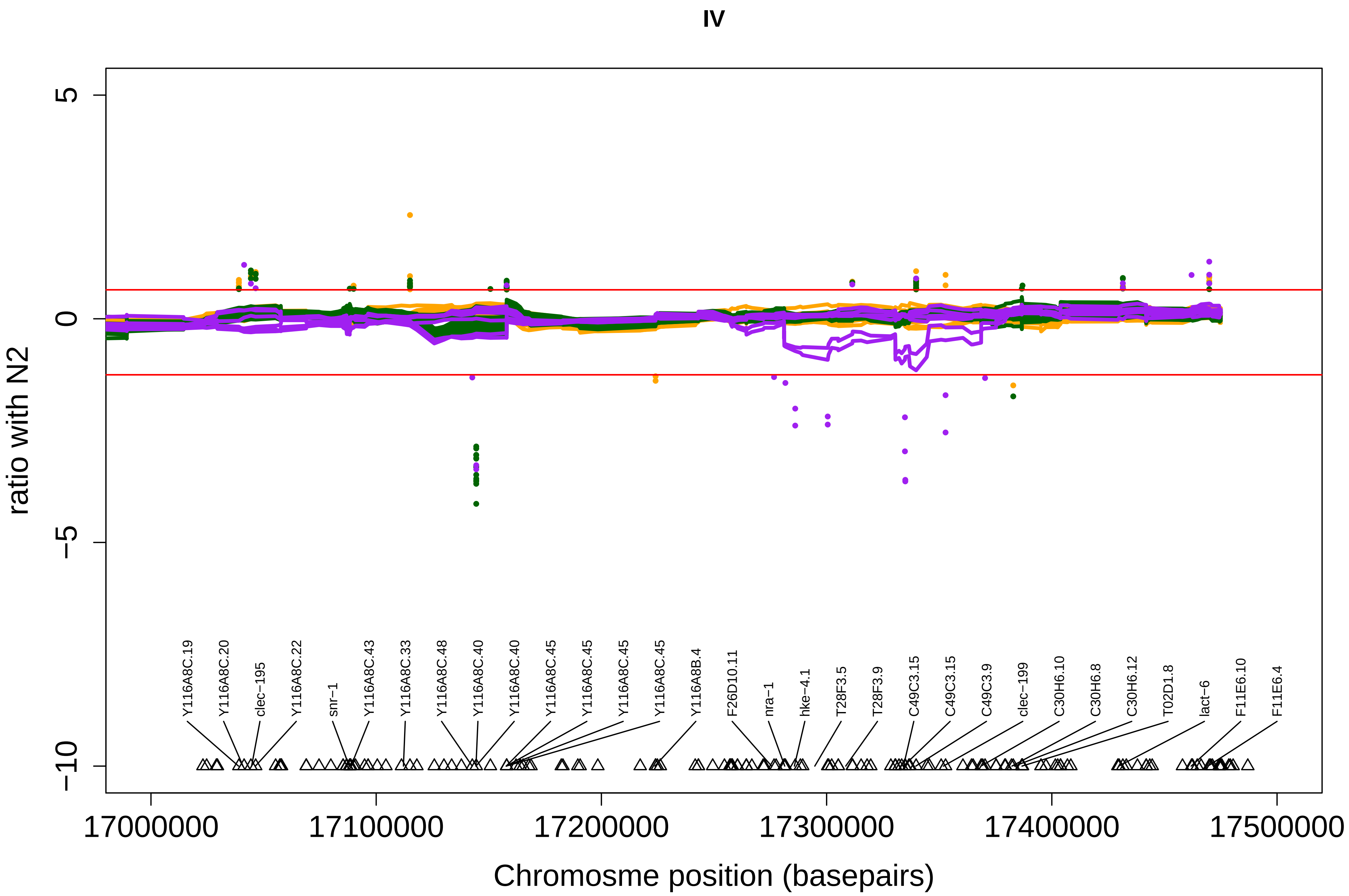
IV
ratio with N2
5
0
−5
−10
Chromosme position (basepairs)
17000000
17100000
17200000
17300000
17400000
17500000
Y116A8C.19
Y116A8C.20
clec−195
Y116A8C.22
snr−1
Y116A8C.43
Y116A8C.33
Y116A8C.48
Y116A8C.40
Y116A8C.40
Y116A8C.45
Y116A8C.45
Y116A8C.45
Y116A8C.45
Y116A8B.4
F26D10.11
nra−1
hke−4.1
T28F3.5
T28F3.9
C49C3.15
C49C3.15
C49C3.9
clec−199
C30H6.10
C30H6.8
C30H6.12
T02D1.8
lact−6
F11E6.10
F11E6.4

IV

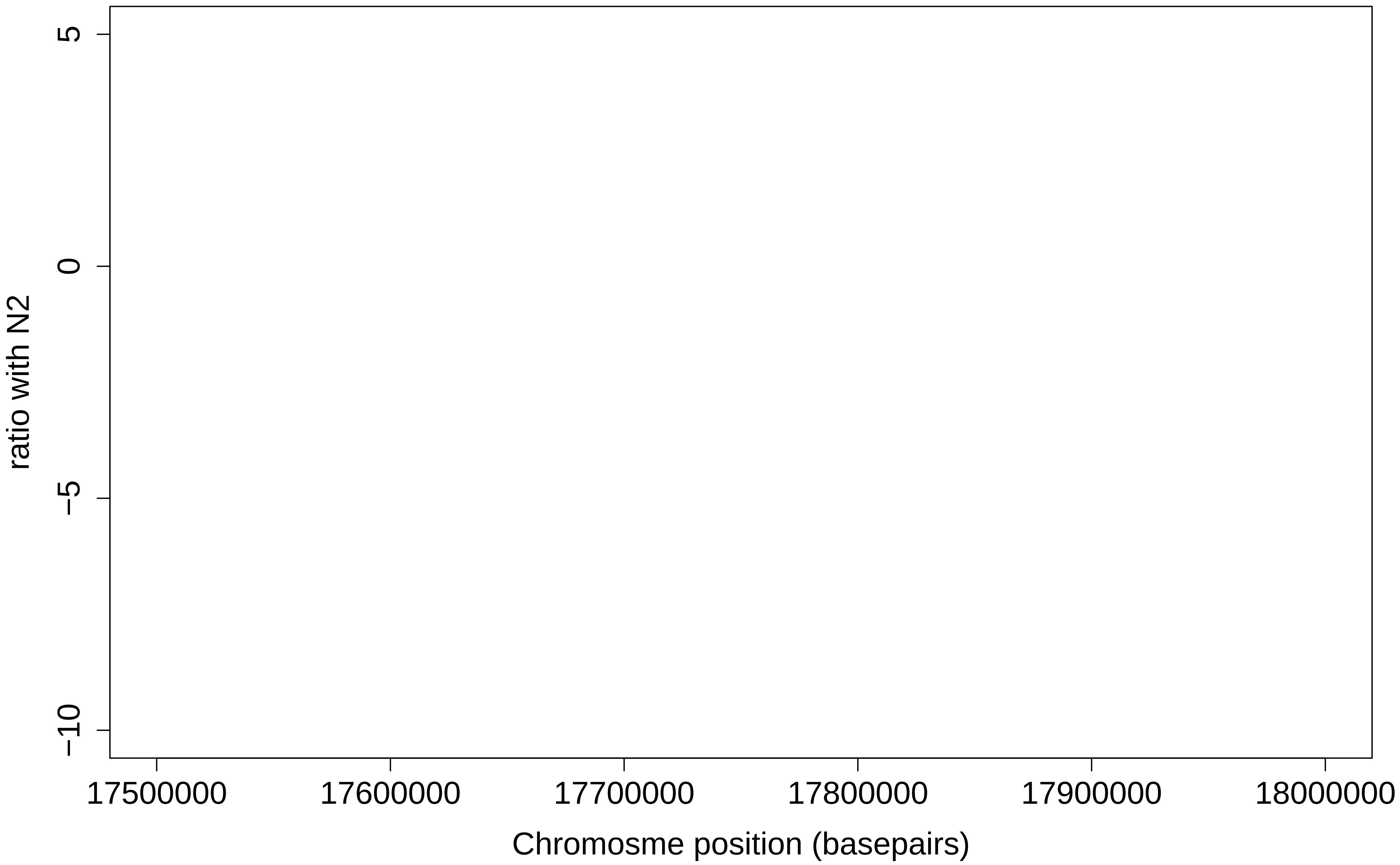
5
0
−5
−10
ratio with N2
17500000
17600000
17700000
17800000
17900000
18000000
Chromosme position (basepairs)

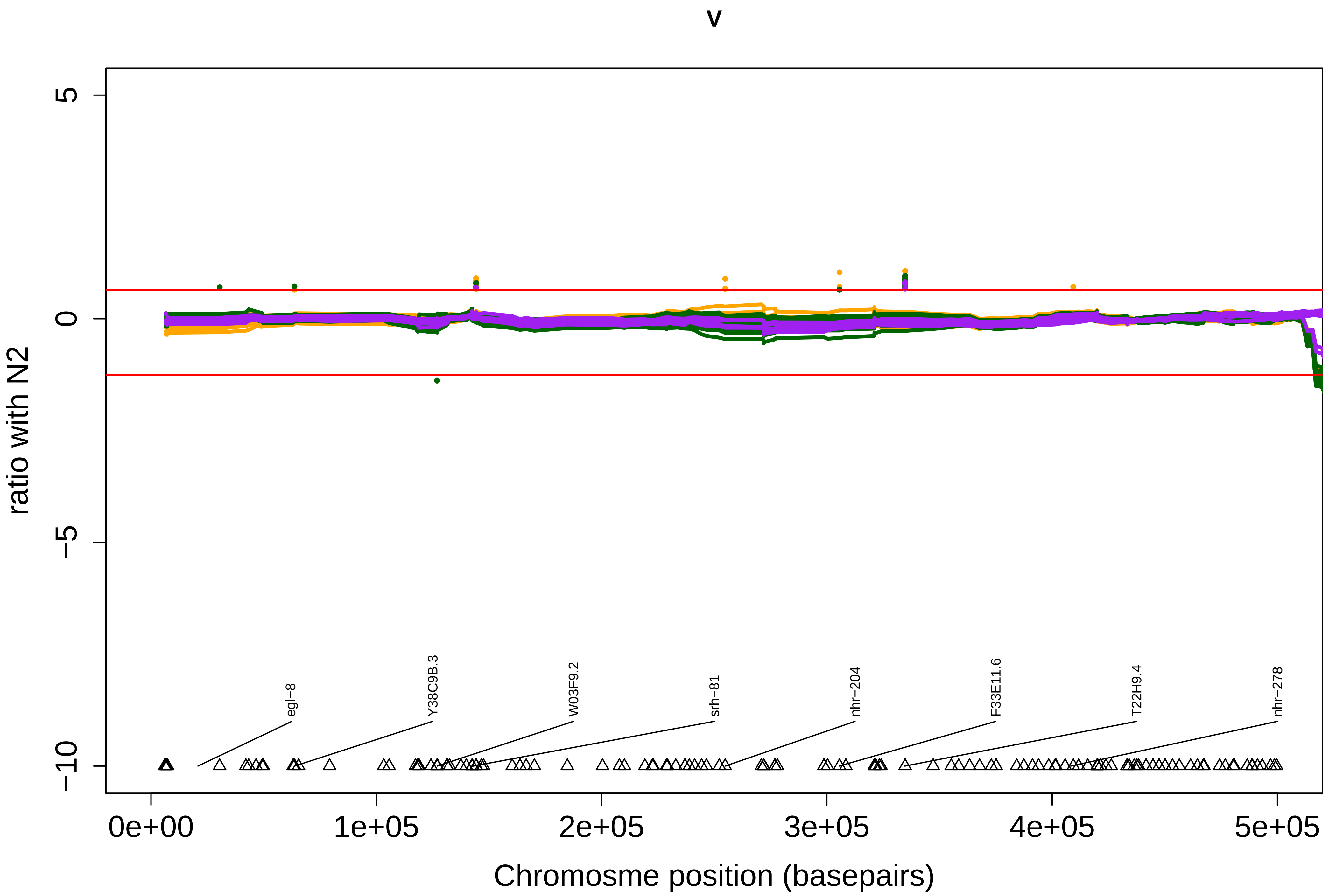
V
ratio with N2
5
0
−5
−10
egl−8
Y38C9B.3
W03F9.2
srh−81
nhr−204
F33E11.6
T22H9.4
nhr−278
0e+00
1e+05
2e+05
3e+05
4e+05
5e+05
Chromosme position (basepairs)

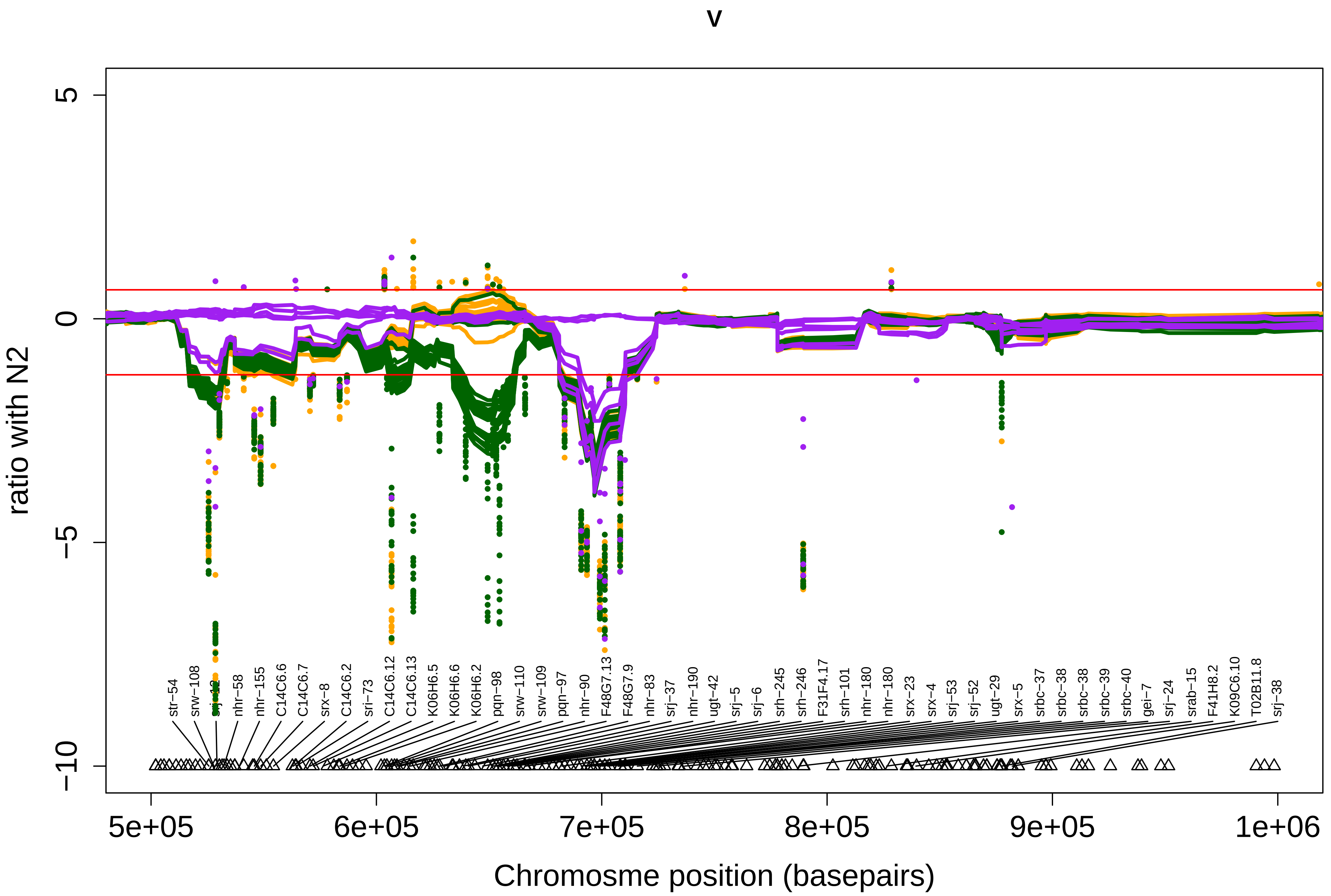
V
ratio with N2
Chromosme position (basepairs)
5
0
−5
−10
5e+05
6e+05
7e+05
8e+05
9e+05
1e+06
str−54
srw−108
srj−12
nhr−58
nhr−155
C14C6.6
C14C6.7
srx−8
C14C6.2
sri−73
C14C6.12
C14C6.13
K06H6.5
K06H6.6
K06H6.2
pqn−98
srw−110
srw−109
pqn−97
nhr−90
F48G7.13
F48G7.9
nhr−83
srj−37
nhr−190
ugt−42
srj−5
srj−6
srh−245
srh−246
F31F4.17
srh−101
nhr−180
nhr−180
srx−23
srx−4
srj−53
srj−52
ugt−29
srx−5
srbc−37
srbc−38
srbc−38
srbc−39
srbc−40
gei−7
srj−24
srab−15
F41H8.2
K09C6.10
T02B11.8
srj−38

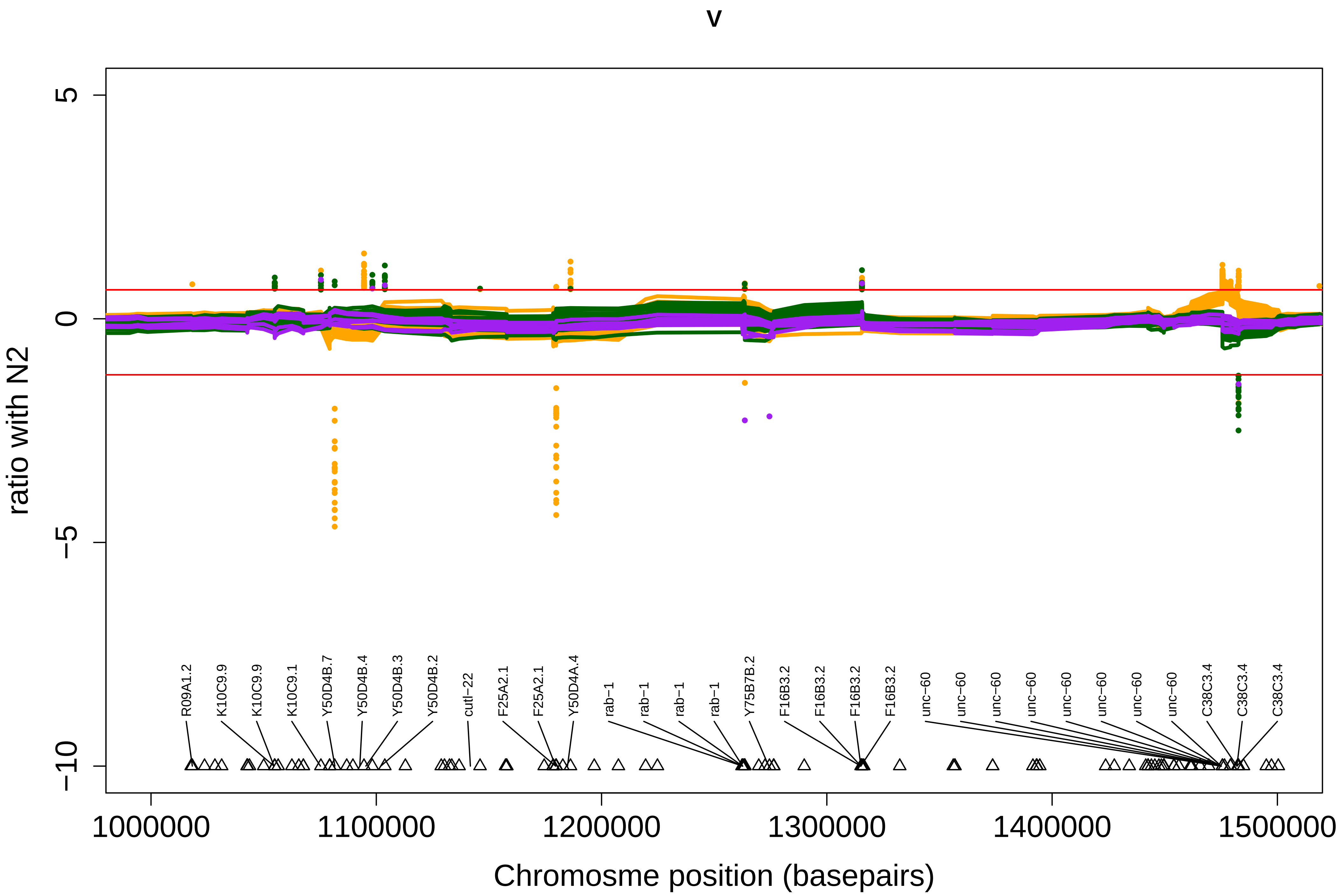

V
ratio with N2
5
0
−5
−10
R09A1.2
K10C9.9
K10C9.9
K10C9.1
Y50D4B.7
Y50D4B.4
Y50D4B.3
Y50D4B.2
cutl−22
F25A2.1
F25A2.1
Y50D4A.4
rab−1
rab−1
rab−1
rab−1
Y75B7B.2
F16B3.2
F16B3.2
F16B3.2
F16B3.2
unc−60
unc−60
unc−60
unc−60
unc−60
unc−60
unc−60
unc−60
C38C3.4
C38C3.4
C38C3.4
1000000
1100000
1200000
1300000
1400000
1500000
Chromosme position (basepairs)

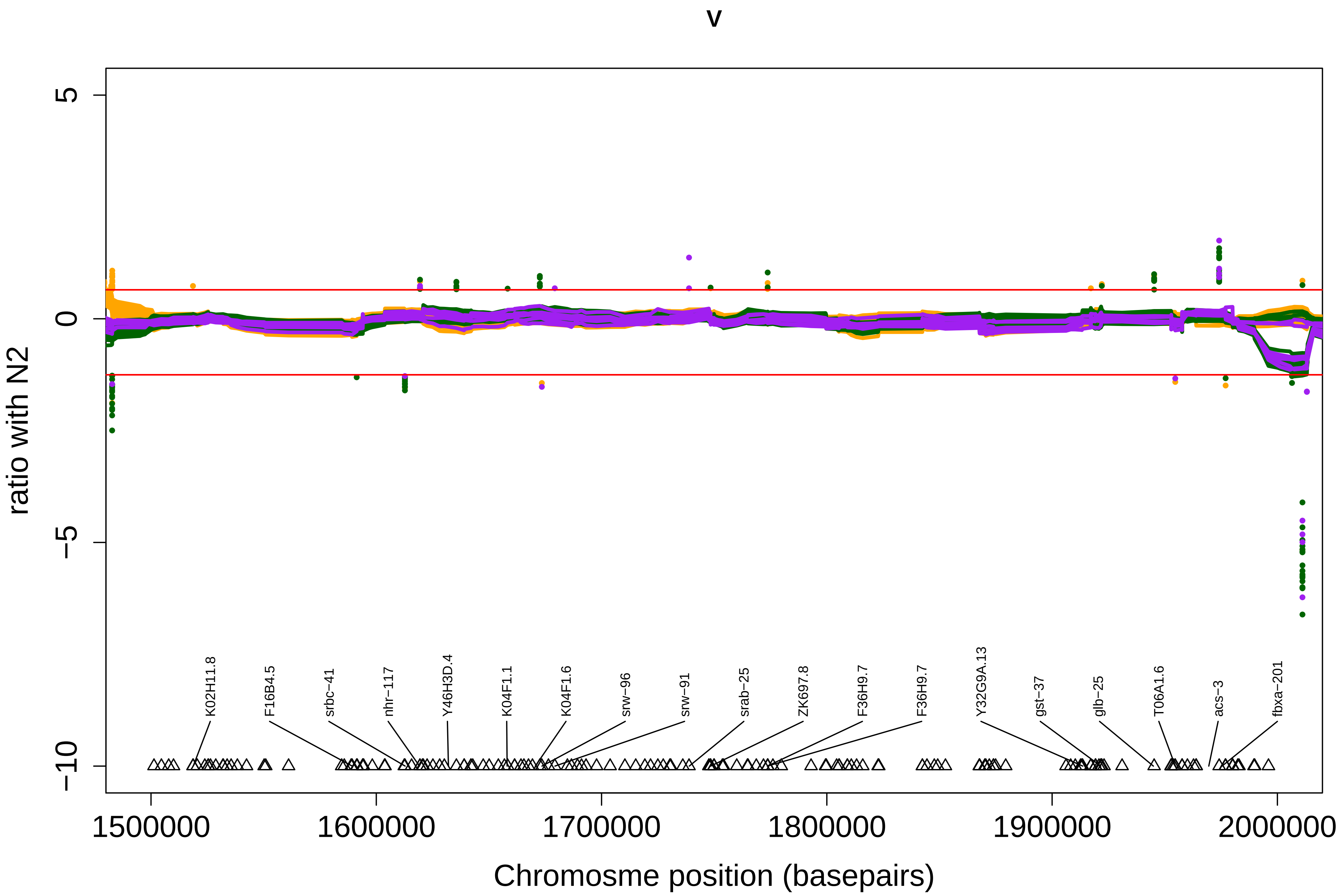

V
ratio with N2
5
0
−5
−10
K02H11.8
F16B4.5
srbc−41
nhr−117
Y46H3D.4
K04F1.1
K04F1.6
srw−96
srw−91
srab−25
ZK697.8
F36H9.7
F36H9.7
Y32G9A.13
gst−37
glb−25
T06A1.6
acs−3
fbxa−201
1500000
1600000
1700000
1800000
1900000
2000000
Chromosme position (basepairs)

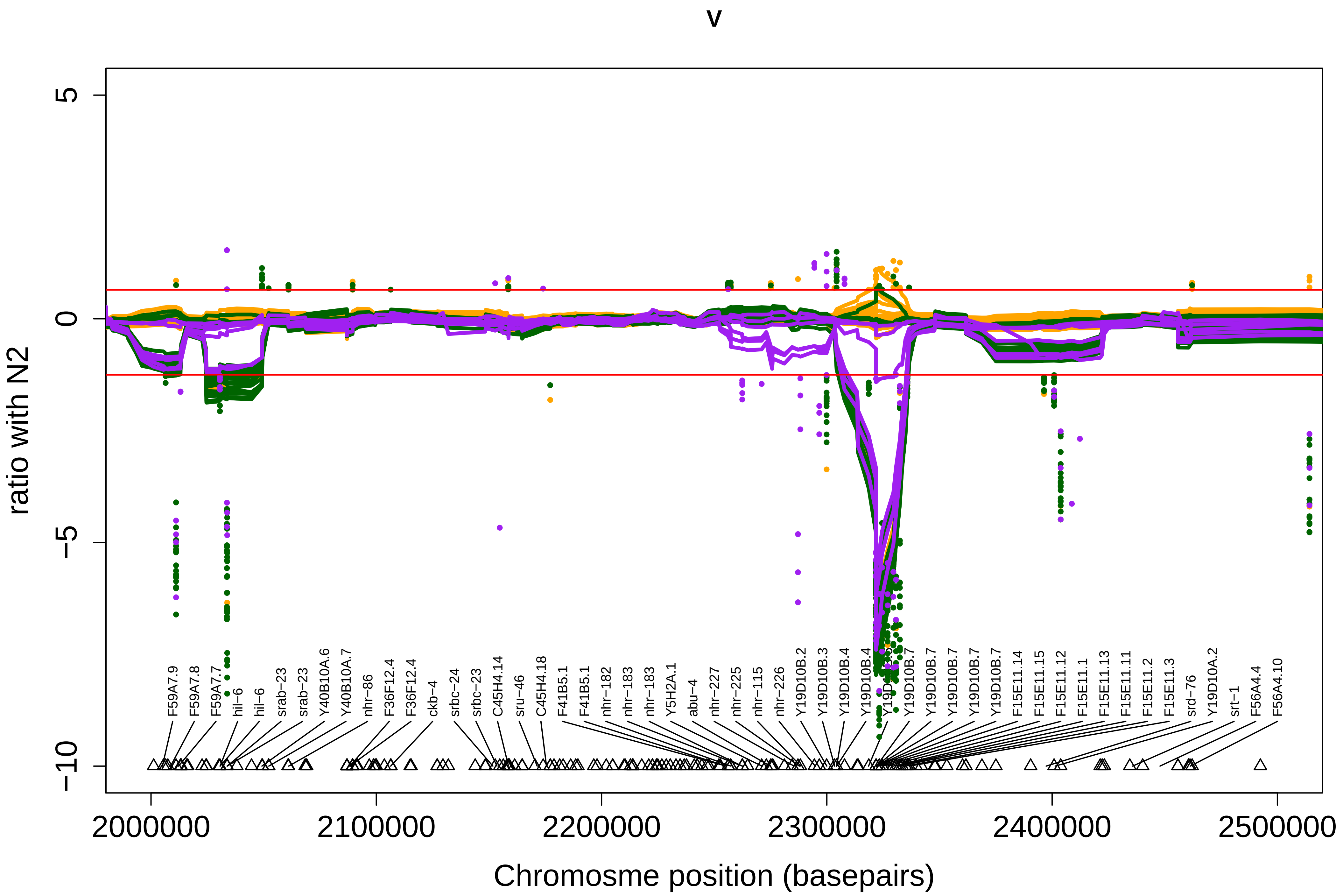

V
ratio with N2
5
0
−5
−10
Chromosme position (basepairs)
2000000
2100000
2200000
2300000
2400000
2500000
F59A7.9
F59A7.8
F59A7.7
hil−6
hil−6
srab−23
srab−23
Y40B10A.6
Y40B10A.7
nhr−86
F36F12.4
F36F12.4
ckb−4
srbc−24
srbc−23
C45H4.14
sru−46
C45H4.18
F41B5.1
F41B5.1
nhr−182
nhr−183
nhr−183
Y5H2A.1
abu−4
nhr−227
nhr−225
nhr−115
nhr−226
Y19D10B.2
Y19D10B.3
Y19D10B.4
Y19D10B.4
Y19D10B.7
Y19D10B.7
Y19D10B.7
Y19D10B.7
Y19D10B.7
F15E11.14
F15E11.15
F15E11.12
F15E11.1
F15E11.13
F15E11.11
F15E11.2
F15E11.3
srd−76
Y19D10A.2
srt−1
F56A4.4
F56A4.10

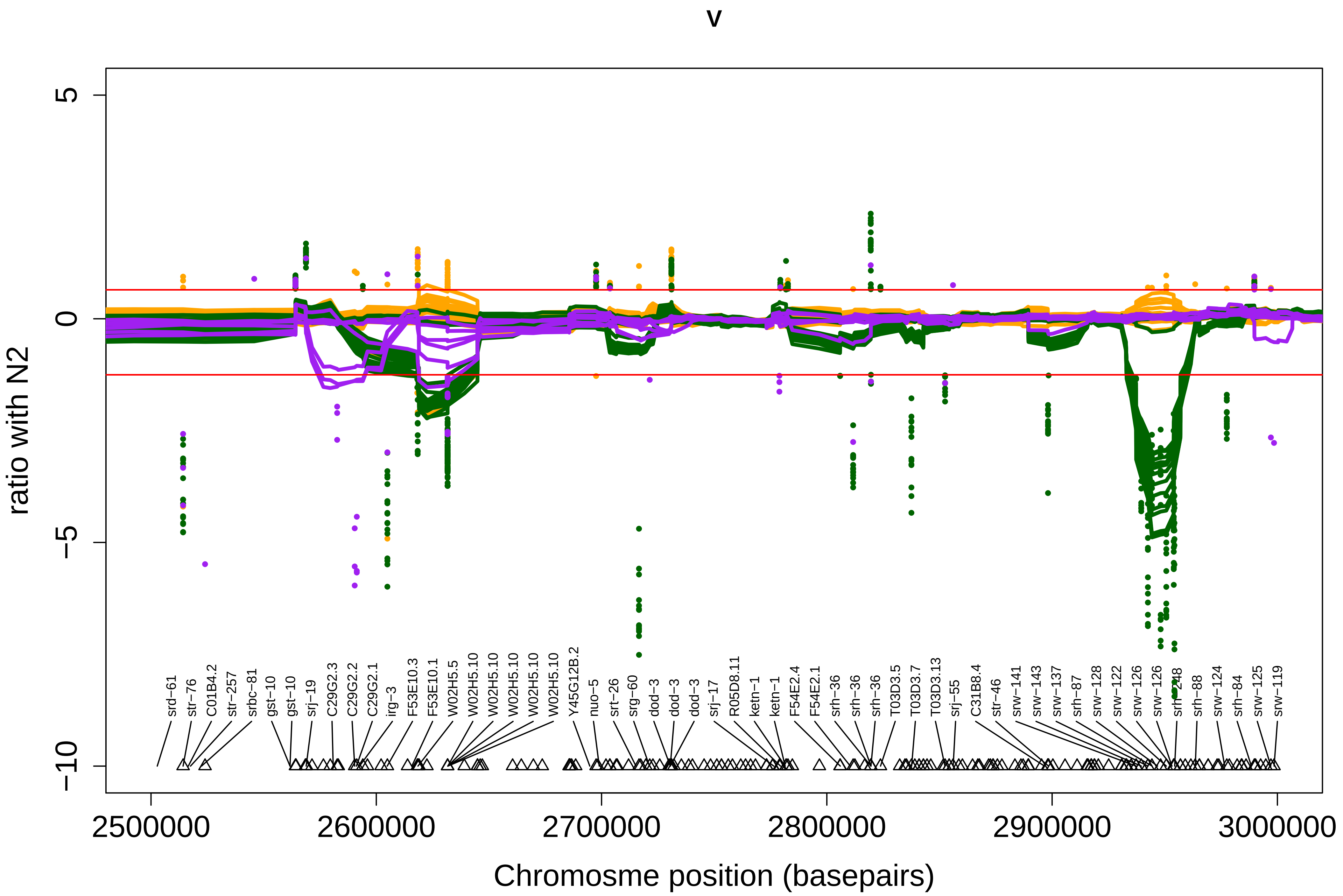
V
ratio with N2
Chromosme position (basepairs)
5
0
−5
−10
2500000
2600000
2700000
2800000
2900000
3000000
srd−61
str−76
C01B4.2
str−257
srbc−81
gst−10
gst−10
srj−19
C29G2.3
C29G2.2
C29G2.1
irg−3
F53E10.3
F53E10.1
W02H5.5
W02H5.10
W02H5.10
W02H5.10
W02H5.10
W02H5.10
Y45G12B.2
nuo−5
srt−26
srg−60
dod−3
dod−3
dod−3
srj−17
R05D8.11
ketn−1
ketn−1
F54E2.4
F54E2.1
srh−36
srh−36
srh−36
T03D3.5
T03D3.7
T03D3.13
srj−55
C31B8.4
str−46
srw−141
srw−143
srw−137
srh−87
srw−128
srw−122
srw−126
srw−126
srh−248
srh−88
srw−124
srh−84
srw−125
srw−119

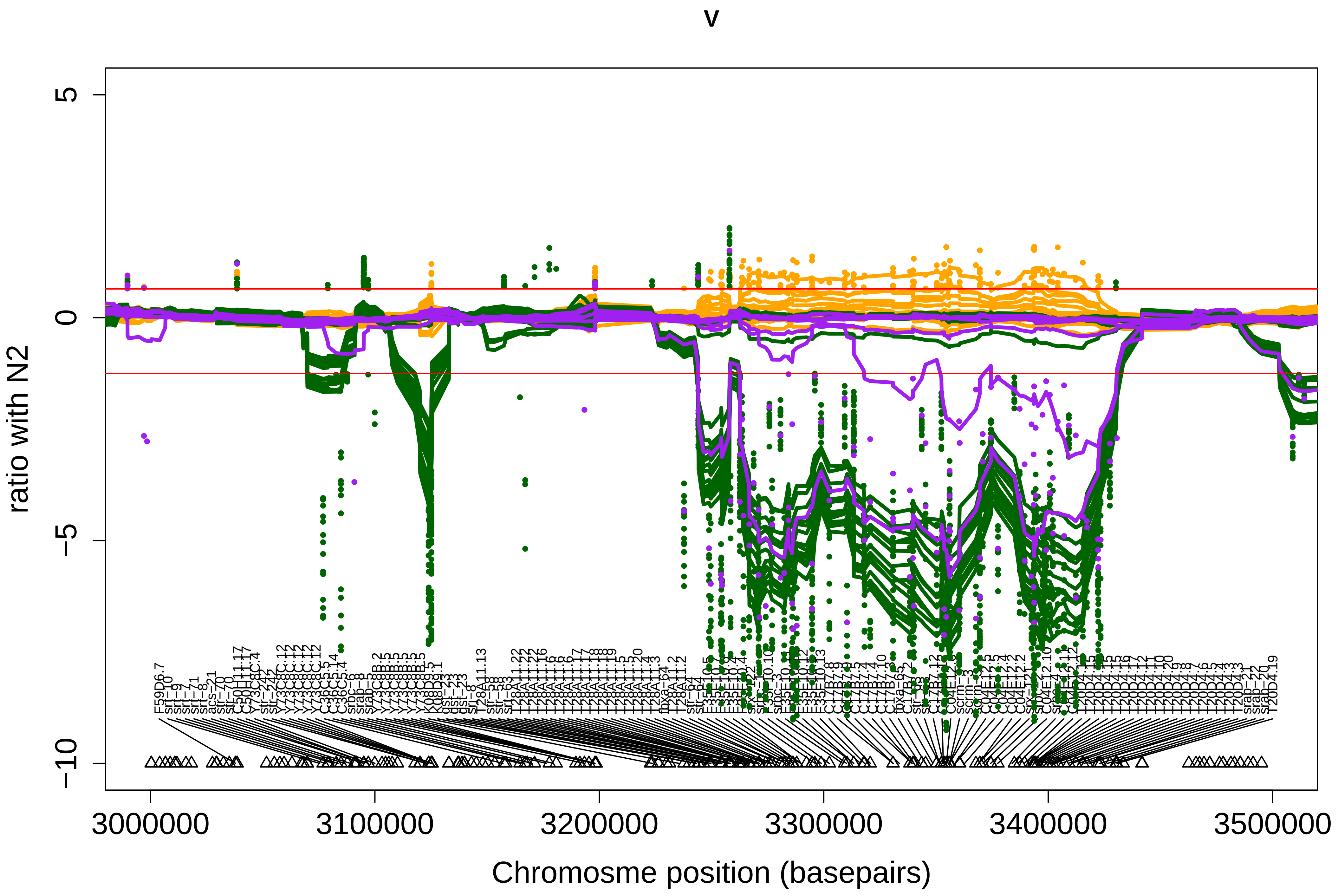
V
ratio with N2
5
0
−5
−10
3000000
3100000
3200000
3300000
3400000
3500000
Chromosme position (basepairs)

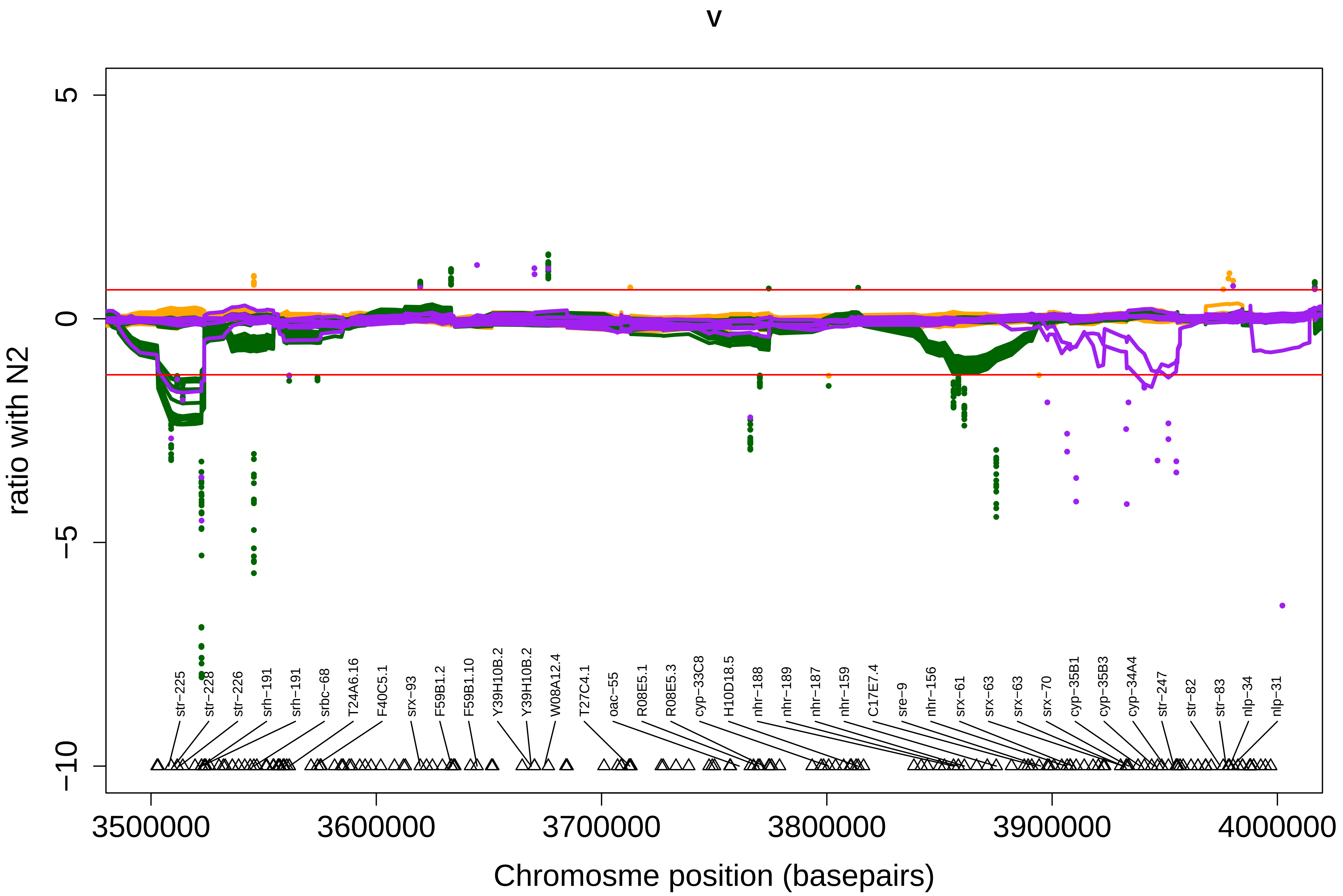

V
ratio with N2
5
0
−5
−10
str−225
str−228
str−226
srh−191
srh−191
srbc−68
T24A6.16
F40C5.1
srx−93
F59B1.2
F59B1.10
Y39H10B.2
Y39H10B.2
W08A12.4
T27C4.1
oac−55
R08E5.1
R08E5.3
cyp−33C8
H10D18.5
nhr−188
nhr−189
nhr−187
nhr−159
C17E7.4
sre−9
nhr−156
srx−61
srx−63
srx−63
srx−70
cyp−35B1
cyp−35B3
cyp−34A4
str−247
str−82
str−83
nlp−34
nlp−31
3500000
3600000
3700000
3800000
3900000
4000000
Chromosme position (basepairs)

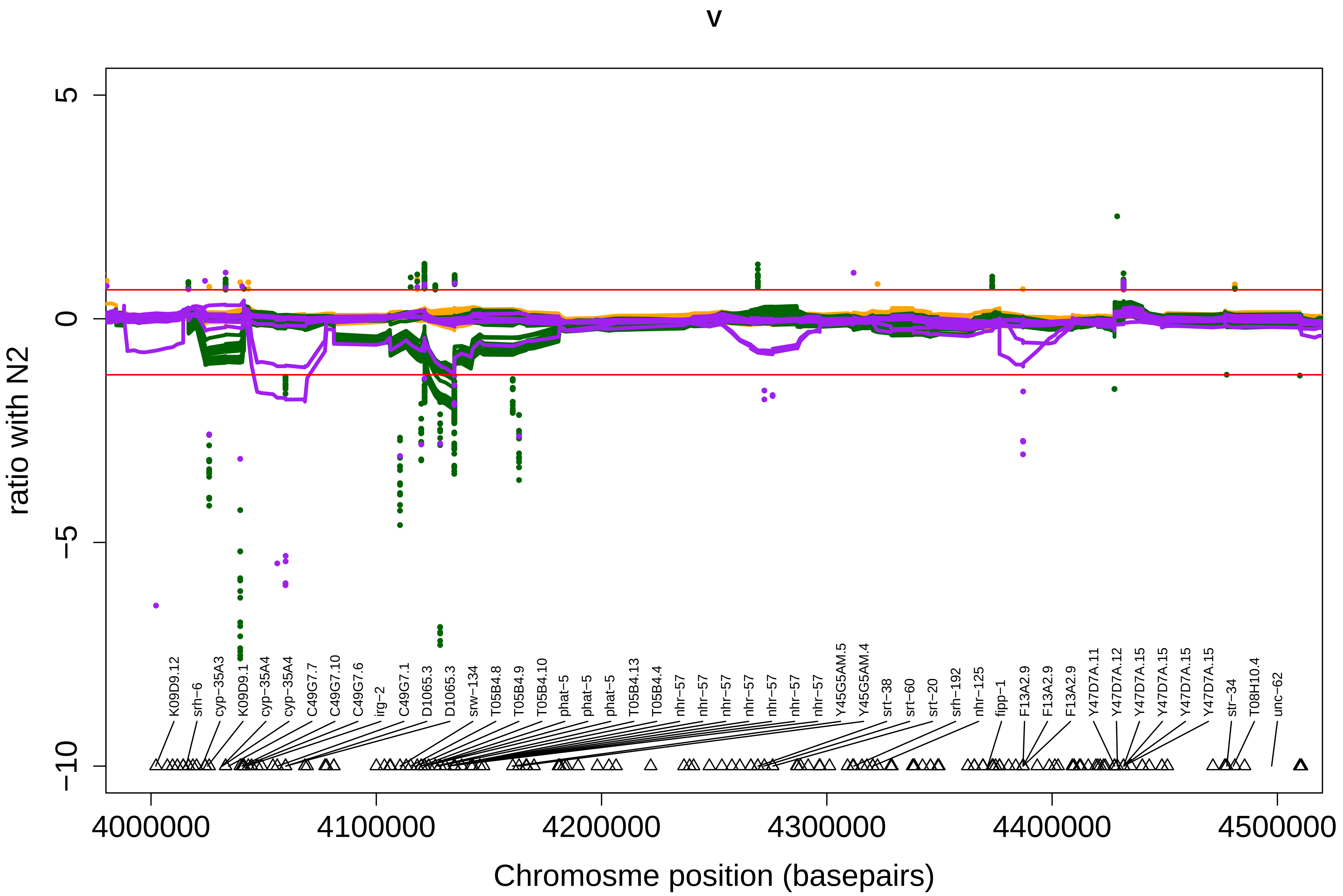
V
ratio with N2
5
0
−5
−10
Chromosme position (basepairs)
4000000
4100000
4200000
4300000
4400000
4500000
K09D9.12
srh−6
cyp−35A3
K09D9.1
cyp−35A4
cyp−35A4
C49G7.7
C49G7.10
C49G7.6
irg−2
C49G7.1
D1065.3
D1065.3
srw−134
T05B4.8
T05B4.9
T05B4.10
phat−5
phat−5
phat−5
T05B4.13
T05B4.4
nhr−57
nhr−57
nhr−57
nhr−57
nhr−57
nhr−57
nhr−57
Y45G5AM.5
Y45G5AM.4
srt−38
srt−60
srt−20
srh−192
nhr−125
fipp−1
F13A2.9
F13A2.9
F13A2.9
Y47D7A.11
Y47D7A.12
Y47D7A.15
Y47D7A.15
Y47D7A.15
Y47D7A.15
str−34
T08H10.4
unc−62

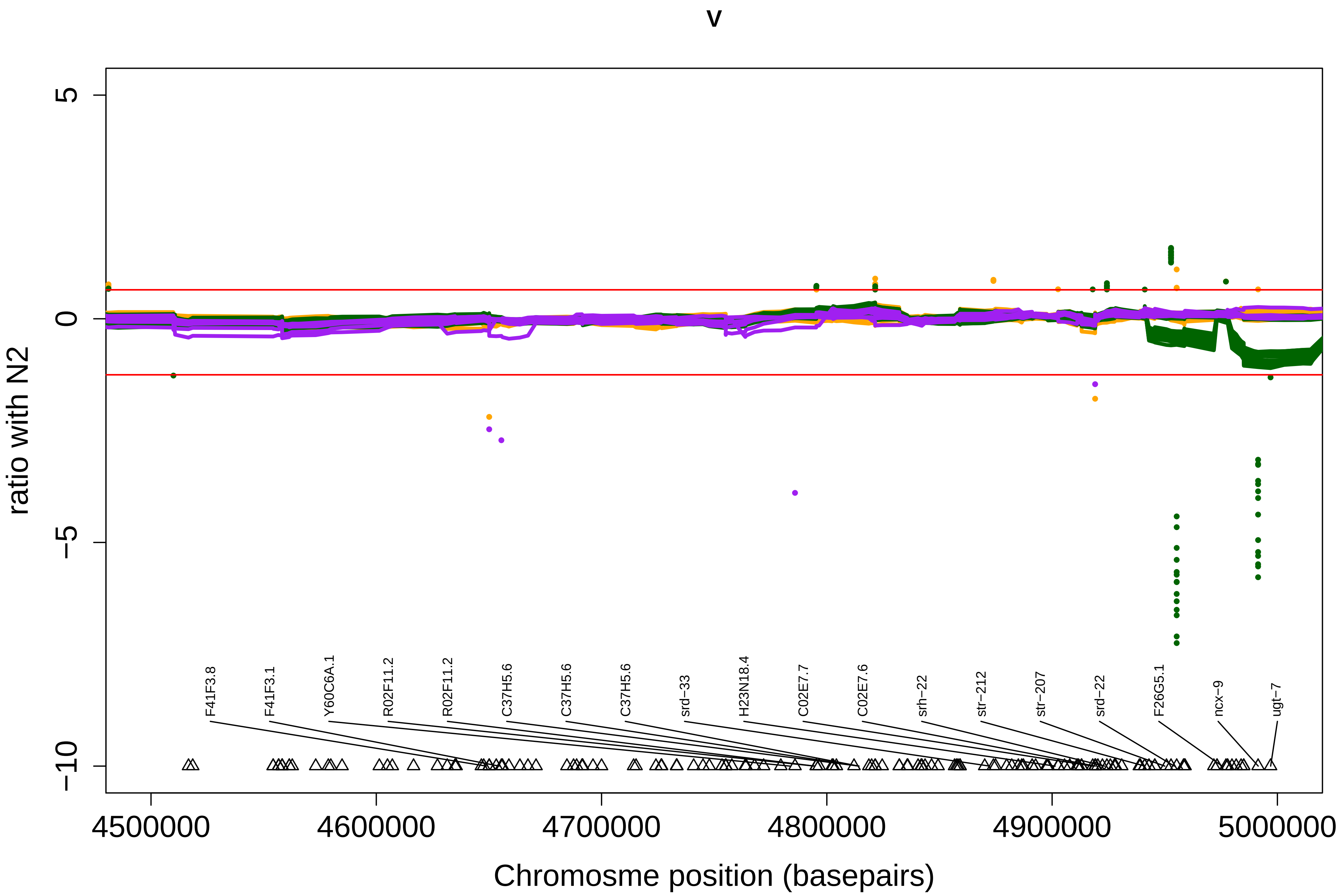
V
ratio with N2
Chromosme position (basepairs)
5
0
−5
−10
4500000
4600000
4700000
4800000
4900000
5000000
F41F3.8
F41F3.1
Y60C6A.1
R02F11.2
R02F11.2
C37H5.6
C37H5.6
C37H5.6
srd−33
H23N18.4
C02E7.7
C02E7.6
srh−22
str−212
str−207
srd−22
F26G5.1
ncx−9
ugt−7

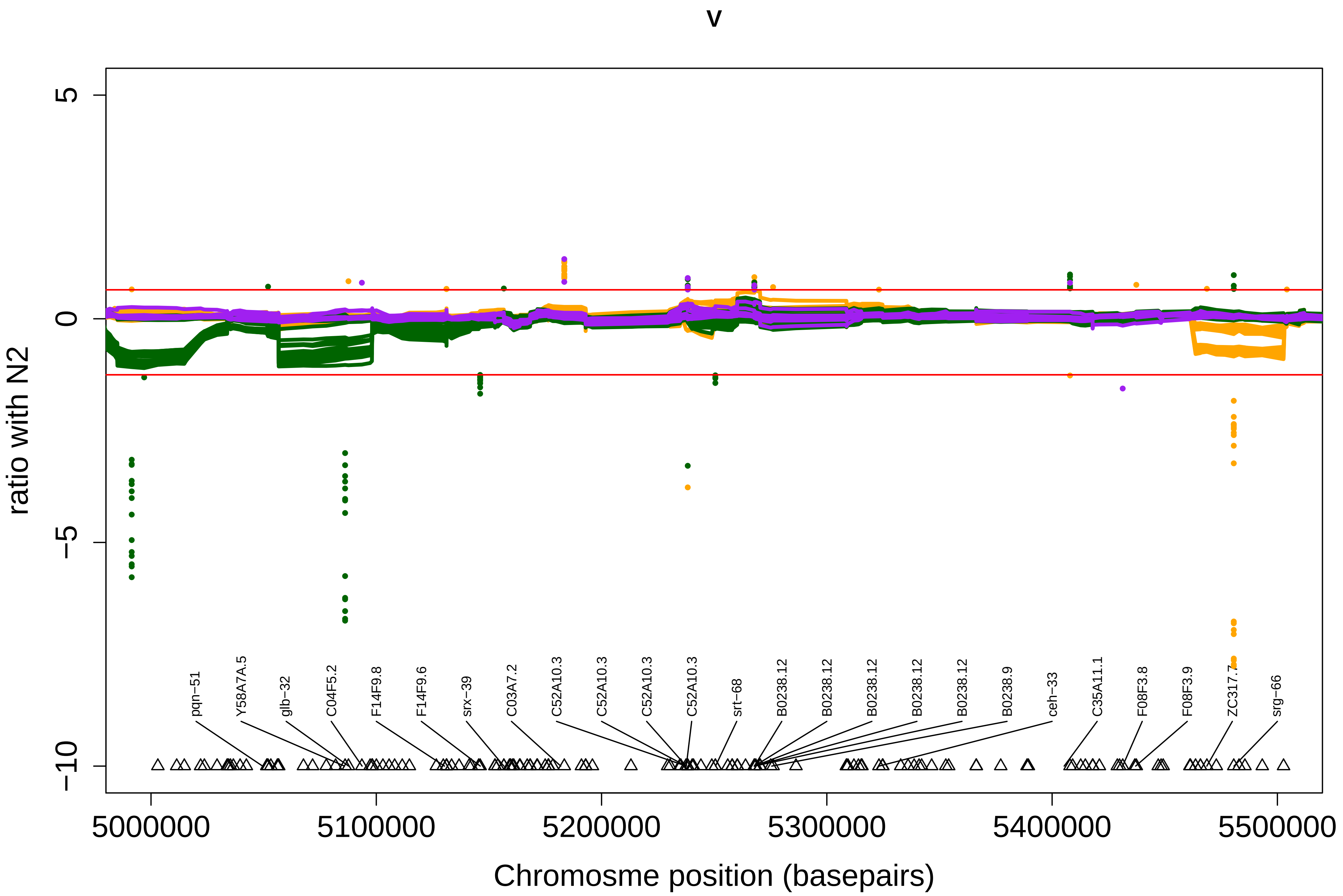
V
ratio with N2
5
0
−5
−10
pqn−51
Y58A7A.5
glb−32
C04F5.2
F14F9.8
F14F9.6
srx−39
C03A7.2
C52A10.3
C52A10.3
C52A10.3
C52A10.3
srt−68
B0238.12
B0238.12
B0238.12
B0238.12
B0238.12
B0238.9
ceh−33
C35A11.1
F08F3.8
F08F3.9
ZC317.7
srg−66
5000000
5100000
5200000
5300000
5400000
5500000
Chromosme position (basepairs)

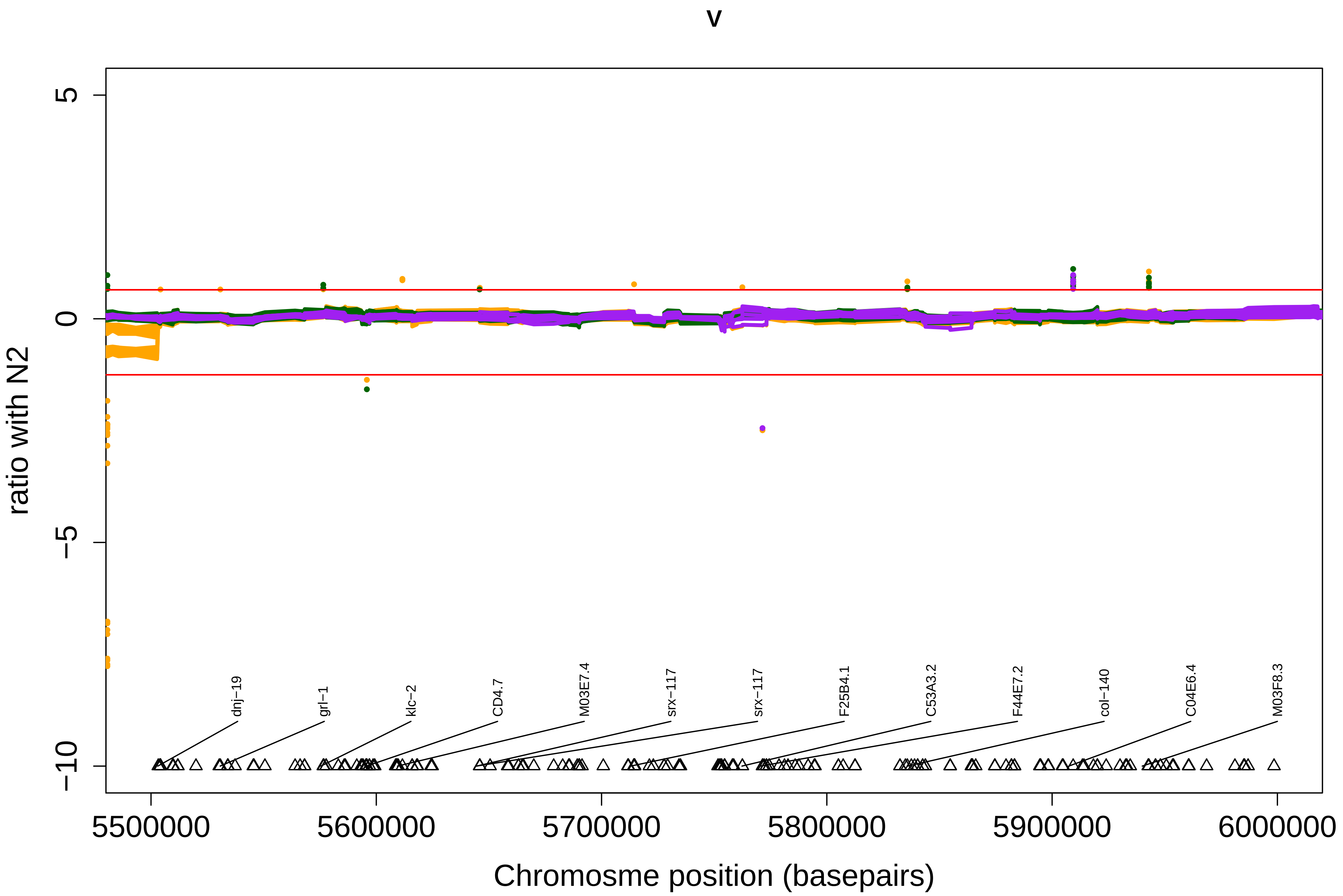

V
ratio with N2
5
0
−5
−10
dnj−19
grl−1
klc−2
CD4.7
M03E7.4
srx−117
srx−117
F25B4.1
C53A3.2
F44E7.2
col−140
C04E6.4
M03F8.3
5500000
5600000
5700000
5800000
5900000
6000000
Chromosme position (basepairs)

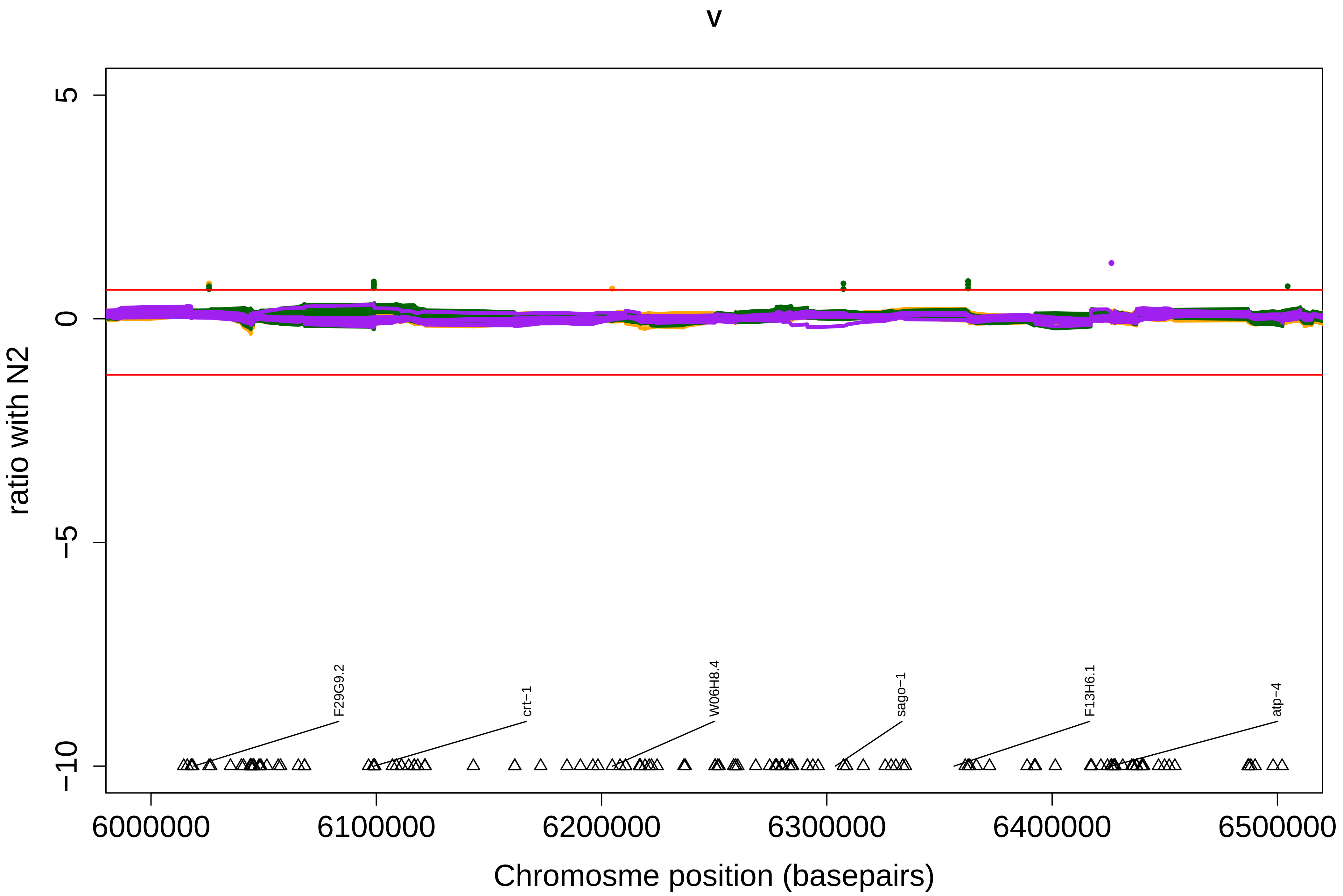
V
ratio with N2
5
0
−5
−10
F29G9.2
crt−1
W06H8.4
sago−1
F13H6.1
atp−4
6000000
6100000
6200000
6300000
6400000
6500000
Chromosme position (basepairs)

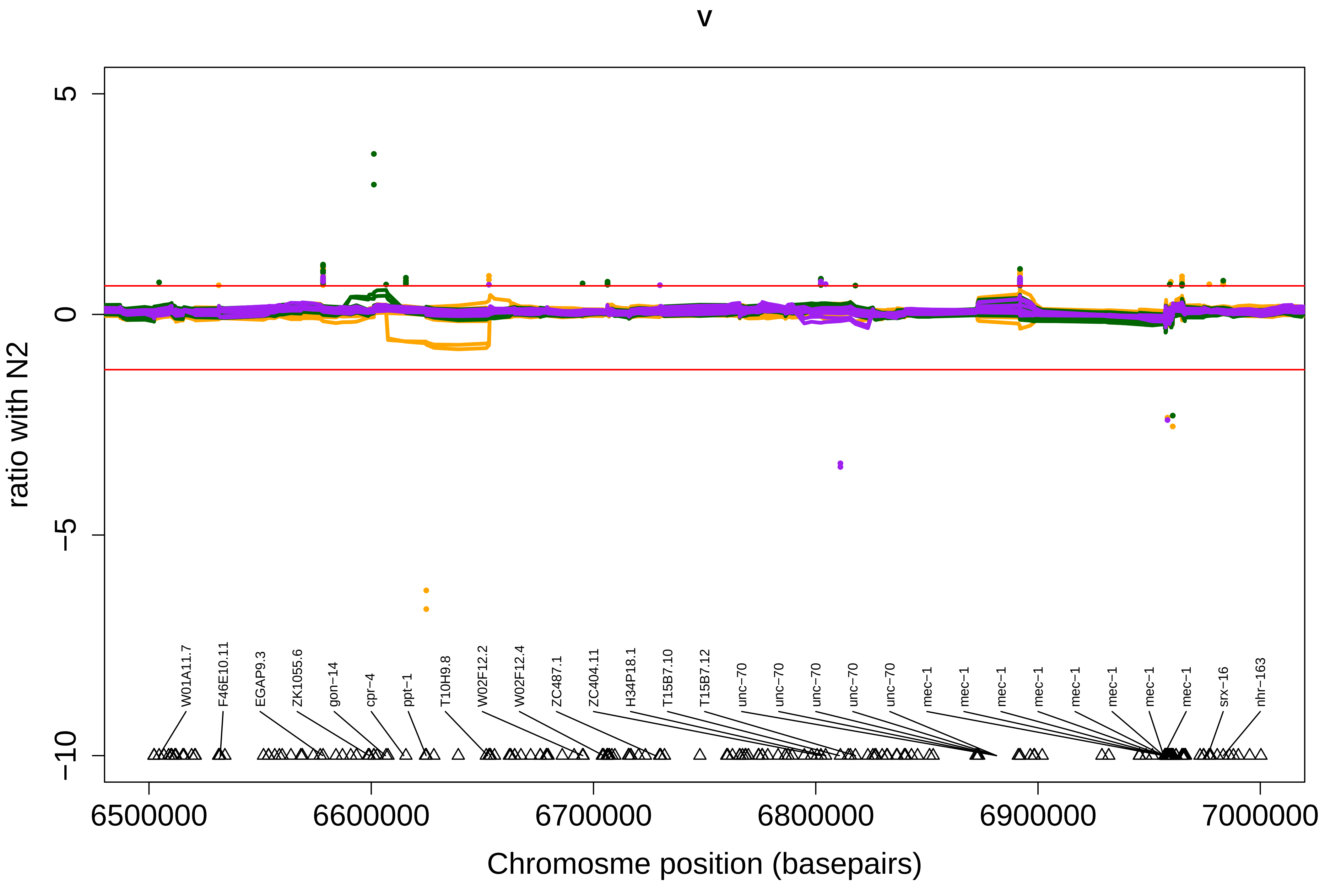

V
ratio with N2
5
0
−5
−10
Chromosme position (basepairs)
6500000
6600000
6700000
6800000
6900000
7000000
W01A11.7
F46E10.11
EGAP9.3
ZK1055.6
gon−14
cpr−4
ppt−1
T10H9.8
W02F12.2
W02F12.4
ZC487.1
ZC404.11
H34P18.1
T15B7.10
T15B7.12
unc−70
unc−70
unc−70
unc−70
unc−70
mec−1
mec−1
mec−1
mec−1
mec−1
mec−1
mec−1
mec−1
srx−16
nhr−163

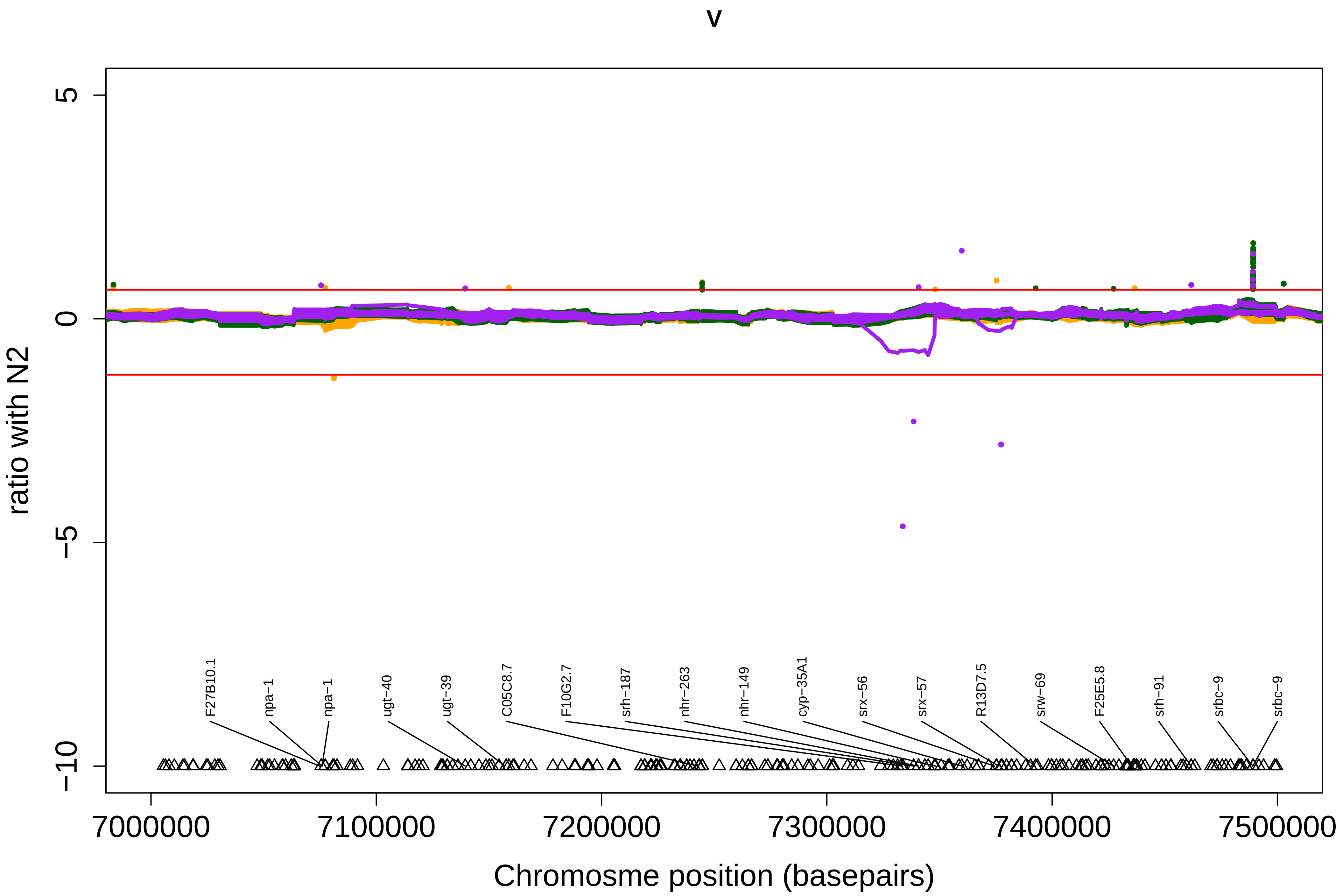

V
ratio with N2
5
0
−5
−10
7000000
7100000
7200000
7300000
7400000
7500000
Chromosme position (basepairs)
F27B10.1
npa−1
npa−1
ugt−40
ugt−39
C05C8.7
F10G2.7
srh−187
nhr−263
nhr−149
cyp−35A1
srx−56
srx−57
R13D7.5
srw−69
F25E5.8
srh−91
srbc−9
srbc−9

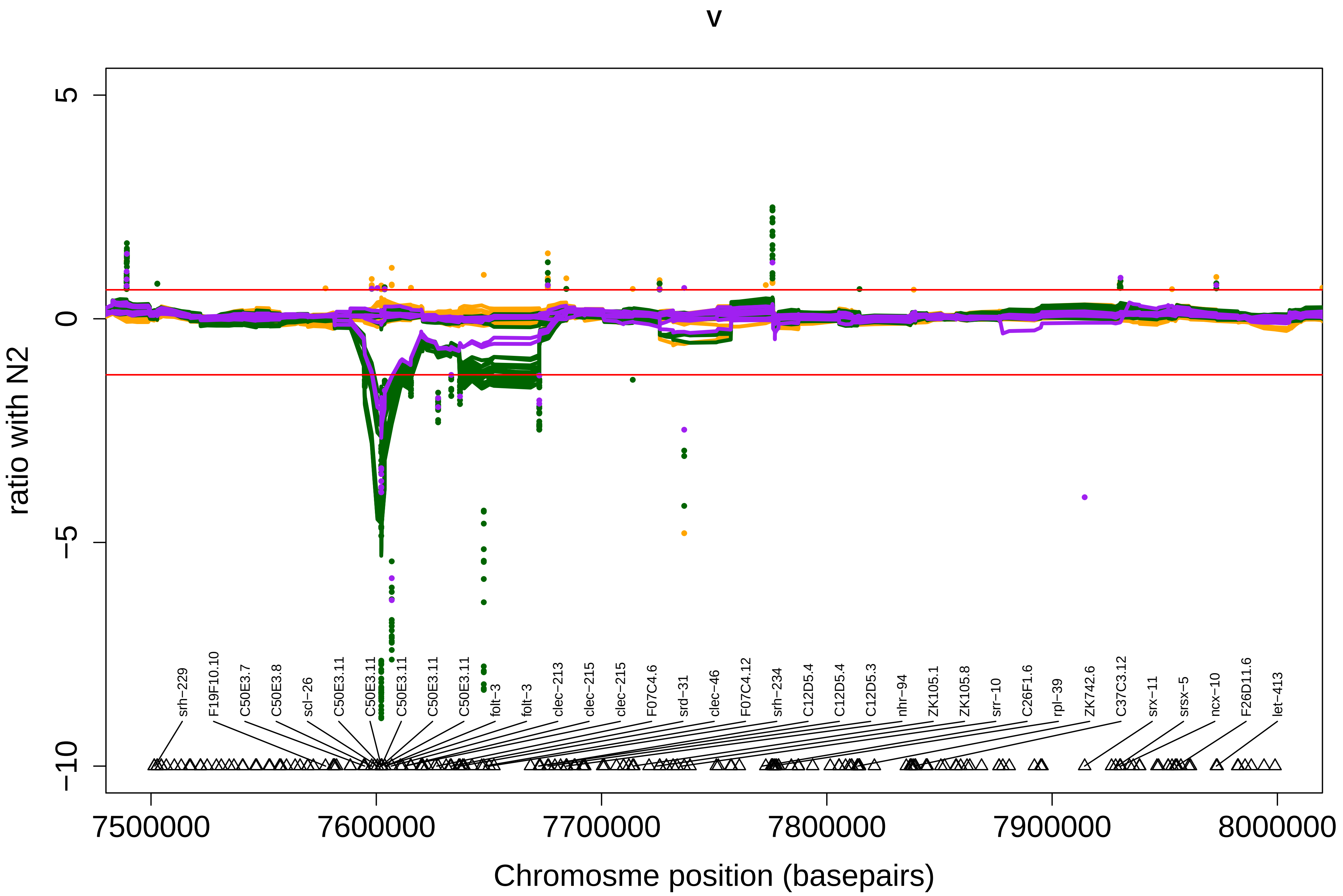
V
ratio with N2
Chromosme position (basepairs)
5
0
−5
−10
7500000
7600000
7700000
7800000
7900000
8000000
srh−229
F19F10.10
C50E3.7
C50E3.8
scl−26
C50E3.11
C50E3.11
C50E3.11
C50E3.11
C50E3.11
folt−3
folt−3
clec−213
clec−215
clec−215
F07C4.6
srd−31
clec−46
F07C4.12
srh−234
C12D5.4
C12D5.4
C12D5.3
nhr−94
ZK105.1
ZK105.8
srr−10
C26F1.6
rpl−39
ZK742.6
C37C3.12
srx−11
srsx−5
ncx−10
F26D11.6
let−413

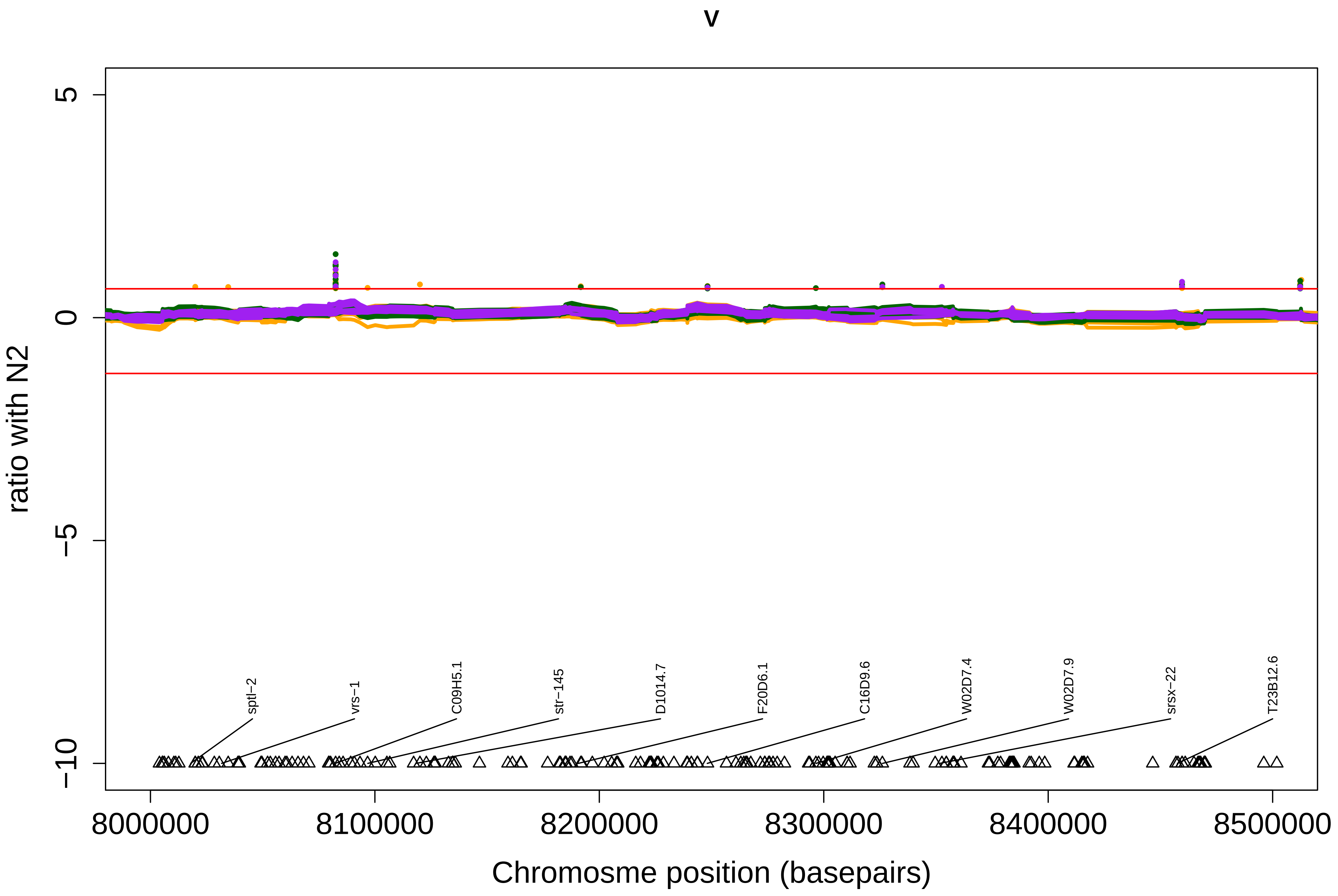

V
ratio with N2
5
0
−5
−10
8000000
8100000
8200000
8300000
8400000
8500000
Chromosme position (basepairs)
sptl−2
vrs−1
C09H5.1
str−145
D1014.7
F20D6.1
C16D9.6
W02D7.4
W02D7.9
srsx−22
T23B12.6

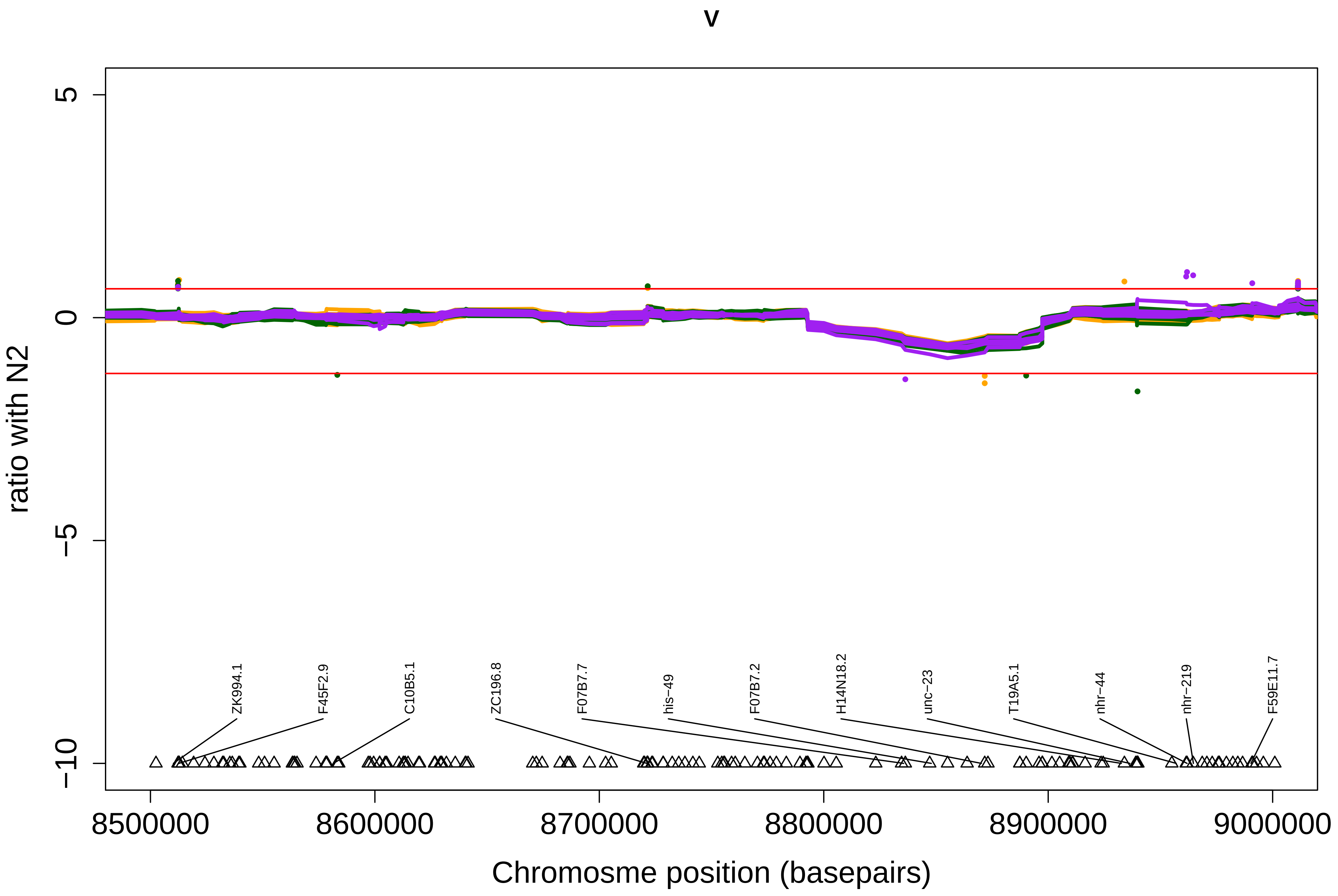
V
ratio with N2
5
0
−5
−10
8500000
8600000
8700000
8800000
8900000
9000000
Chromosme position (basepairs)
ZK994.1
F45F2.9
C10B5.1
ZC196.8
F07B7.7
his−49
F07B7.2
H14N18.2
unc−23
T19A5.1
nhr−44
nhr−219
F59E11.7

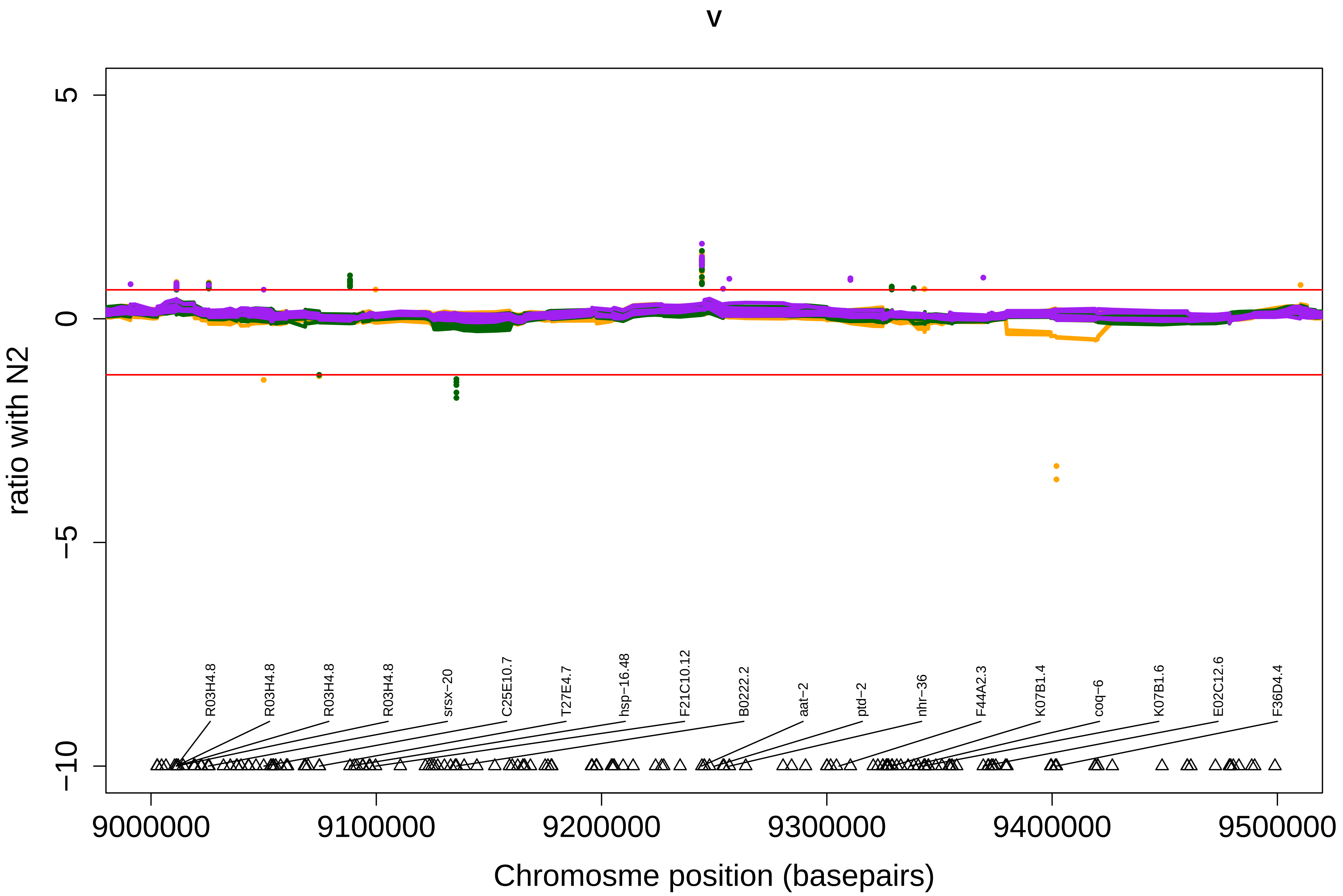

V
ratio with N2
5
0
−5
−10
R03H4.8
R03H4.8
R03H4.8
R03H4.8
srsx−20
C25E10.7
T27E4.7
hsp−16.48
F21C10.12
B0222.2
aat−2
ptd−2
nhr−36
F44A2.3
K07B1.4
coq−6
K07B1.6
E02C12.6
F36D4.4
9000000
9100000
9200000
9300000
9400000
9500000
Chromosme position (basepairs)

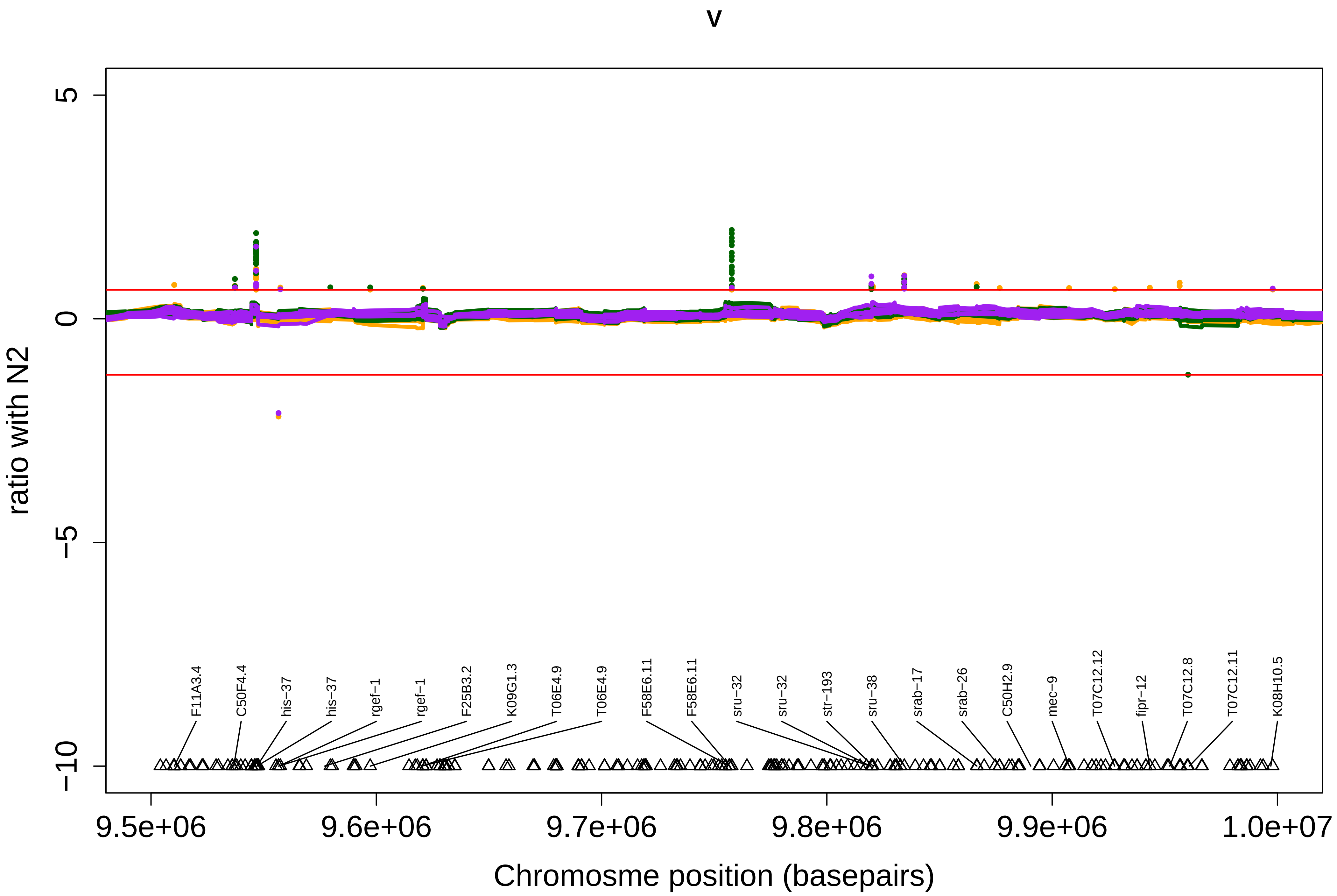

V
ratio with N2
5
0
−5
−10
F11A3.4
C50F4.4
his−37
his−37
rgef−1
rgef−1
F25B3.2
K09G1.3
T06E4.9
T06E4.9
F58E6.11
F58E6.11
sru−32
sru−32
str−193
sru−38
srab−17
srab−26
C50H2.9
mec−9
T07C12.12
fipr−12
T07C12.8
T07C12.11
K08H10.5
9.5e+06
9.6e+06
9.7e+06
9.8e+06
9.9e+06
1.0e+07
Chromosme position (basepairs)

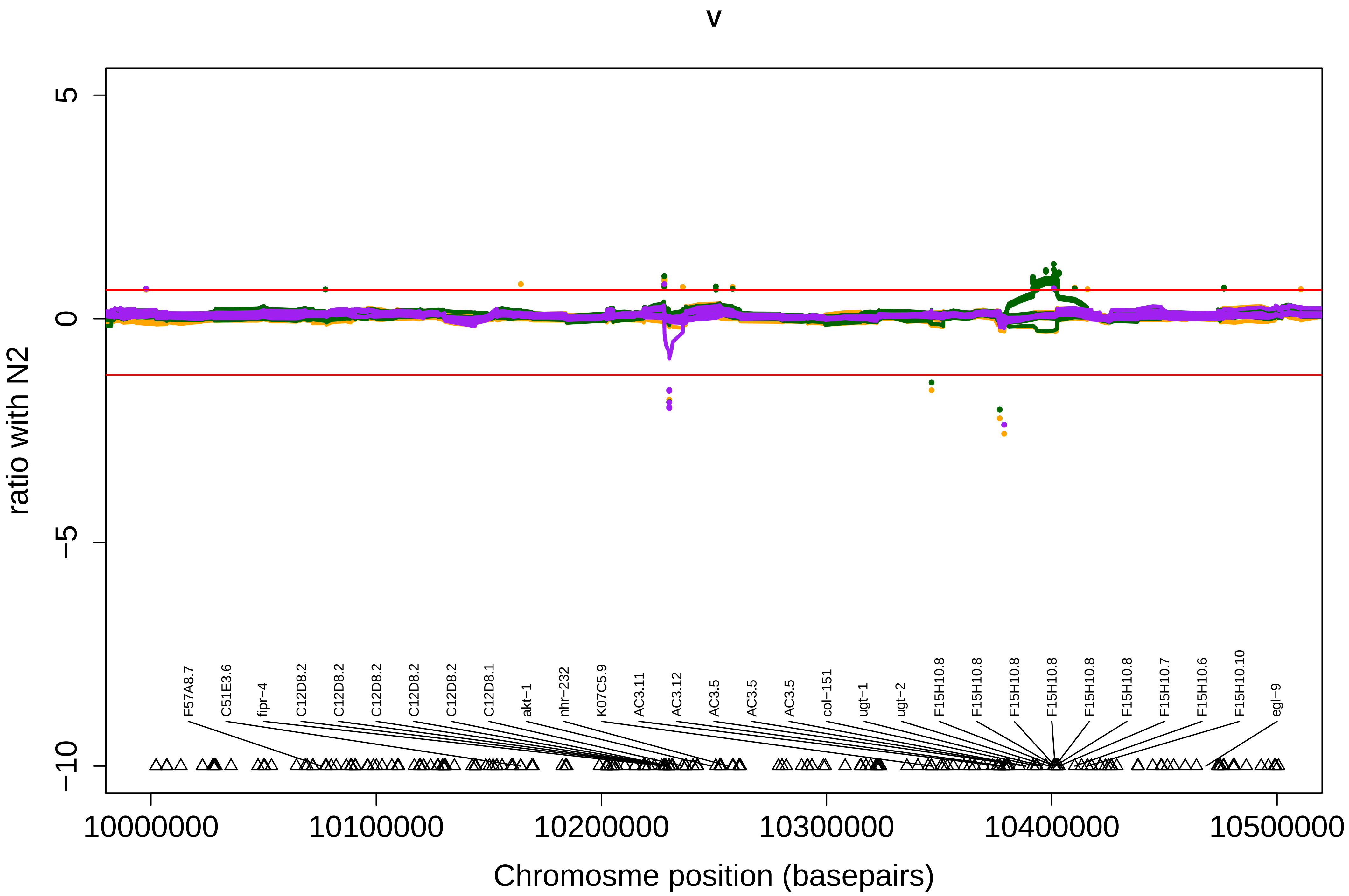

V
ratio with N2
5
0
−5
−10
10000000
10100000
10200000
10300000
10400000
10500000
Chromosme position (basepairs)
F57A8.7
C51E3.6
fipr−4
C12D8.2
C12D8.2
C12D8.2
C12D8.2
C12D8.2
C12D8.1
akt−1
nhr−232
K07C5.9
AC3.11
AC3.12
AC3.5
AC3.5
AC3.5
col−151
ugt−1
ugt−2
F15H10.8
F15H10.8
F15H10.8
F15H10.8
F15H10.8
F15H10.8
F15H10.7
F15H10.6
F15H10.10
egl−9

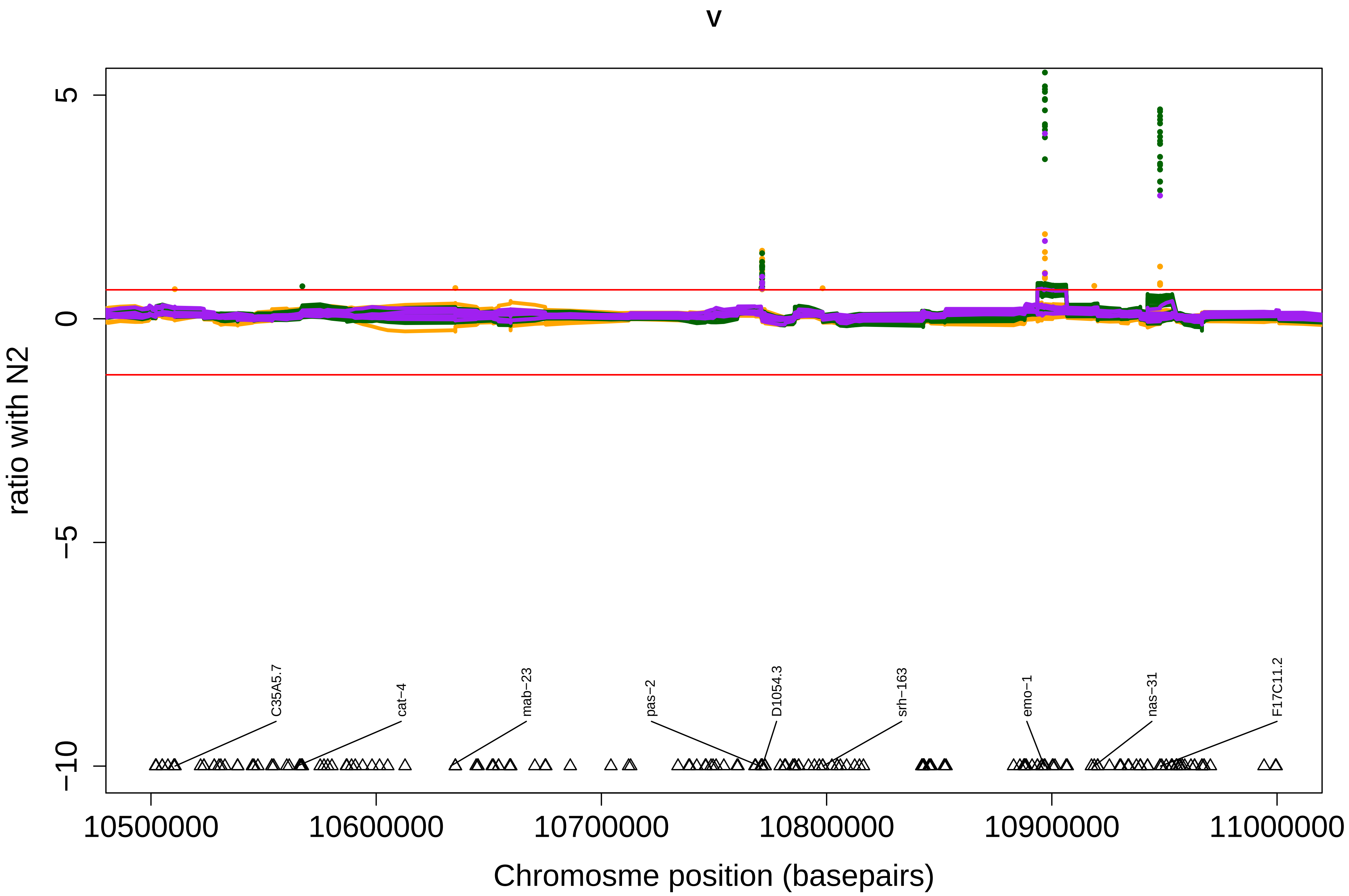

V
ratio with N2
5
0
−5
−10
C35A5.7
cat−4
mab−23
pas−2
D1054.3
srh−163
emo−1
nas−31
F17C11.2
10500000
10600000
10700000
10800000
10900000
11000000
Chromosme position (basepairs)

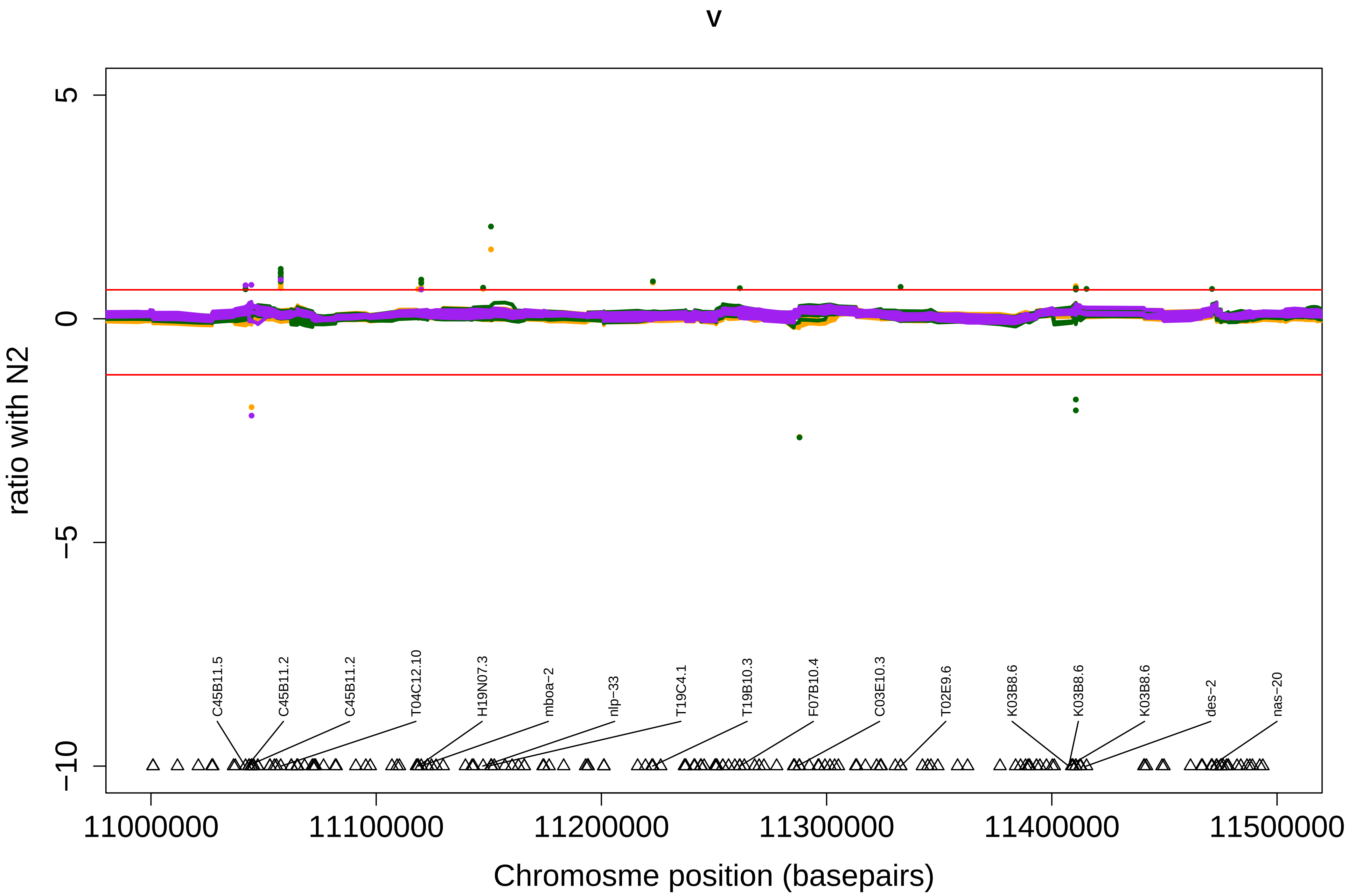

V
ratio with N2
5
0
−5
−10
C45B11.5
C45B11.2
C45B11.2
T04C12.10
H19N07.3
mboa−2
nlp−33
T19C4.1
T19B10.3
F07B10.4
C03E10.3
T02E9.6
K03B8.6
K03B8.6
K03B8.6
des−2
nas−20
11000000
11100000
11200000
11300000
11400000
11500000
Chromosme position (basepairs)

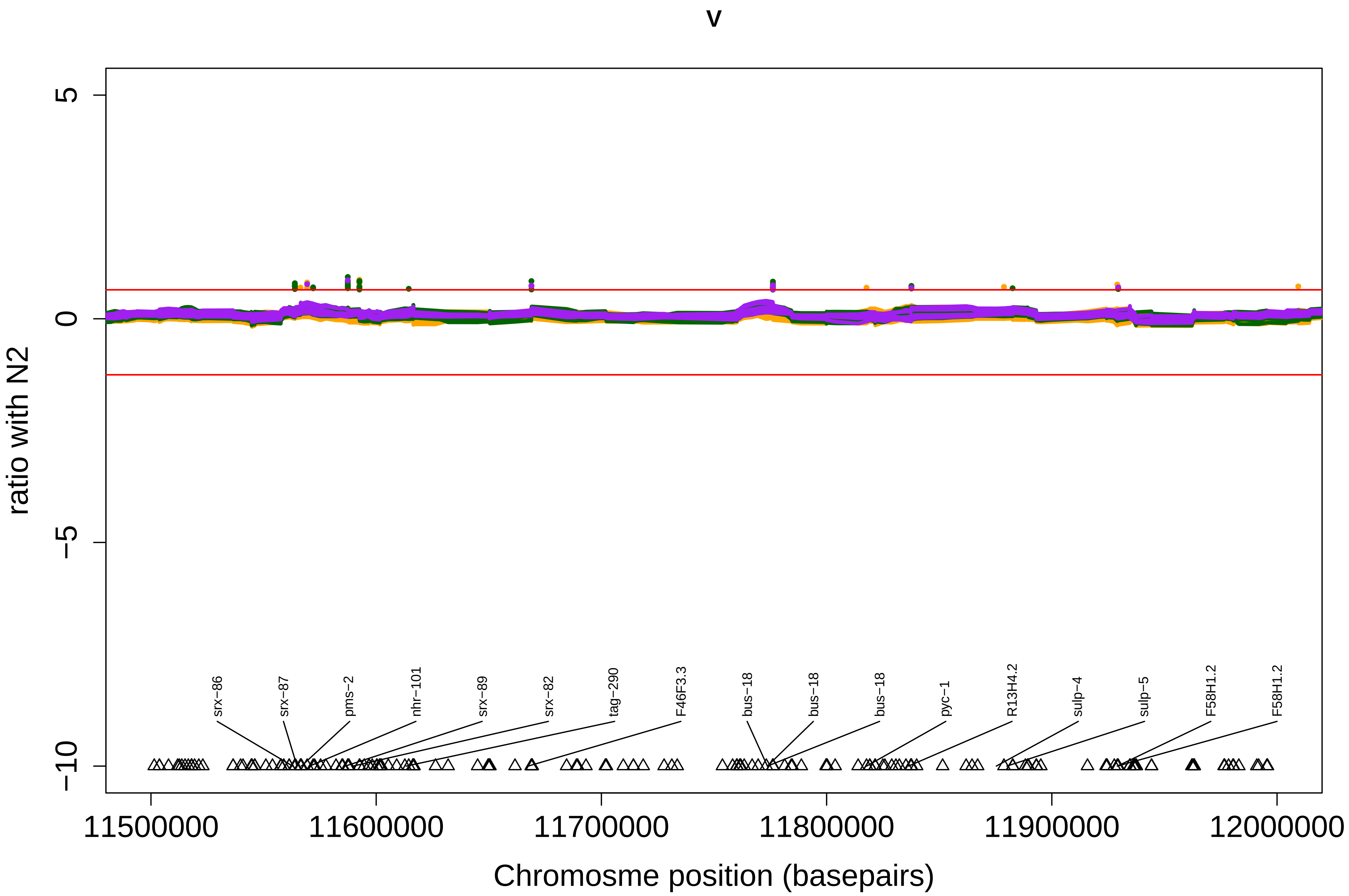

V
ratio with N2
5
0
−5
−10
srx−86
srx−87
pms−2
nhr−101
srx−89
srx−82
tag−290
F46F3.3
bus−18
bus−18
bus−18
pyc−1
R13H4.2
sulp−4
sulp−5
F58H1.2
F58H1.2
11500000
11600000
11700000
11800000
11900000
12000000
Chromosme position (basepairs)

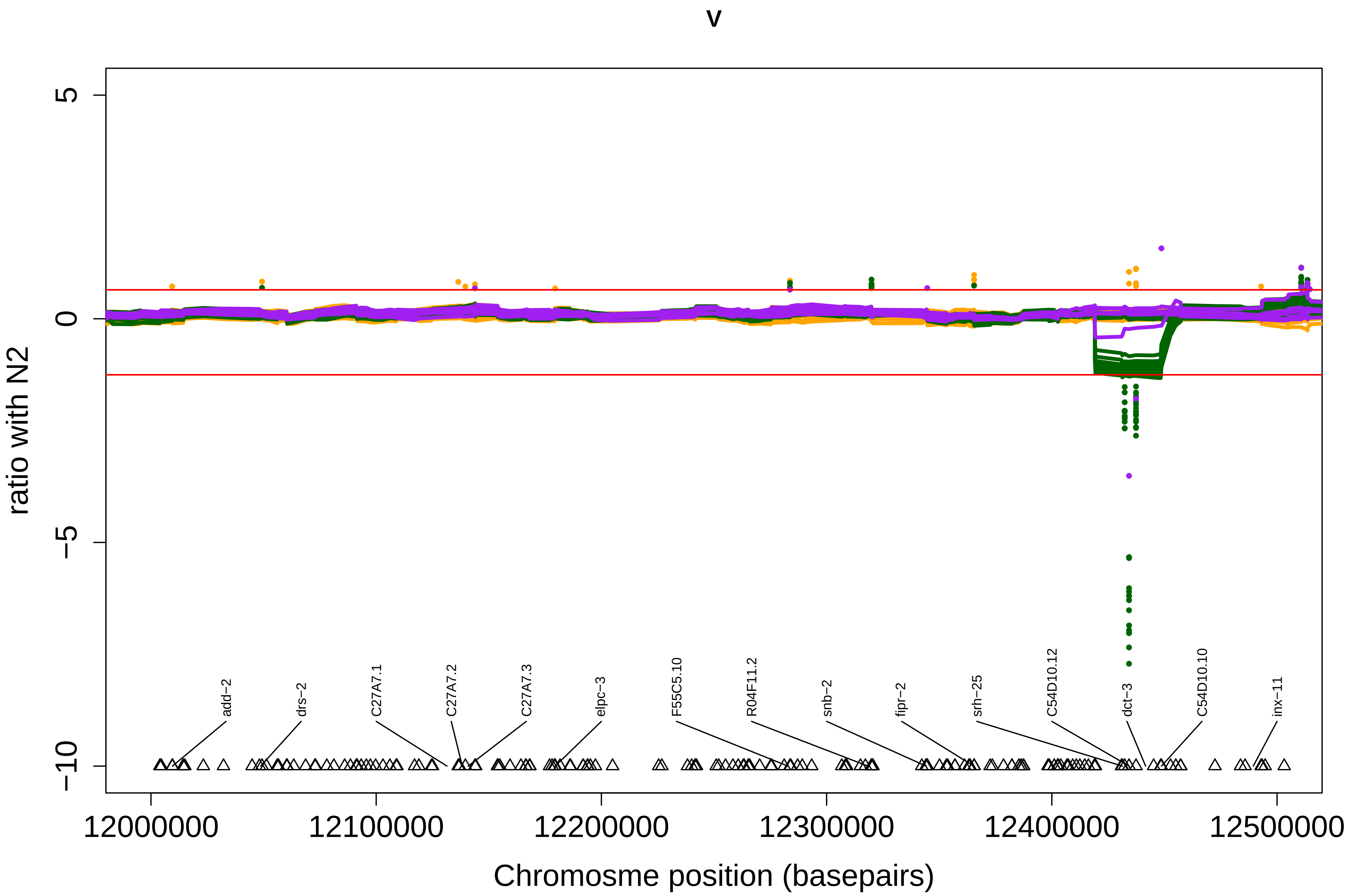
V
ratio with N2
5
0
−5
−10
add−2
drs−2
C27A7.1
C27A7.2
C27A7.3
elpc−3
F55C5.10
R04F11.2
snb−2
flpr−2
srh−25
C54D10.12
dct−3
C54D10.10
inx−11
12000000
12100000
12200000
12300000
12400000
12500000
Chromosme position (basepairs)

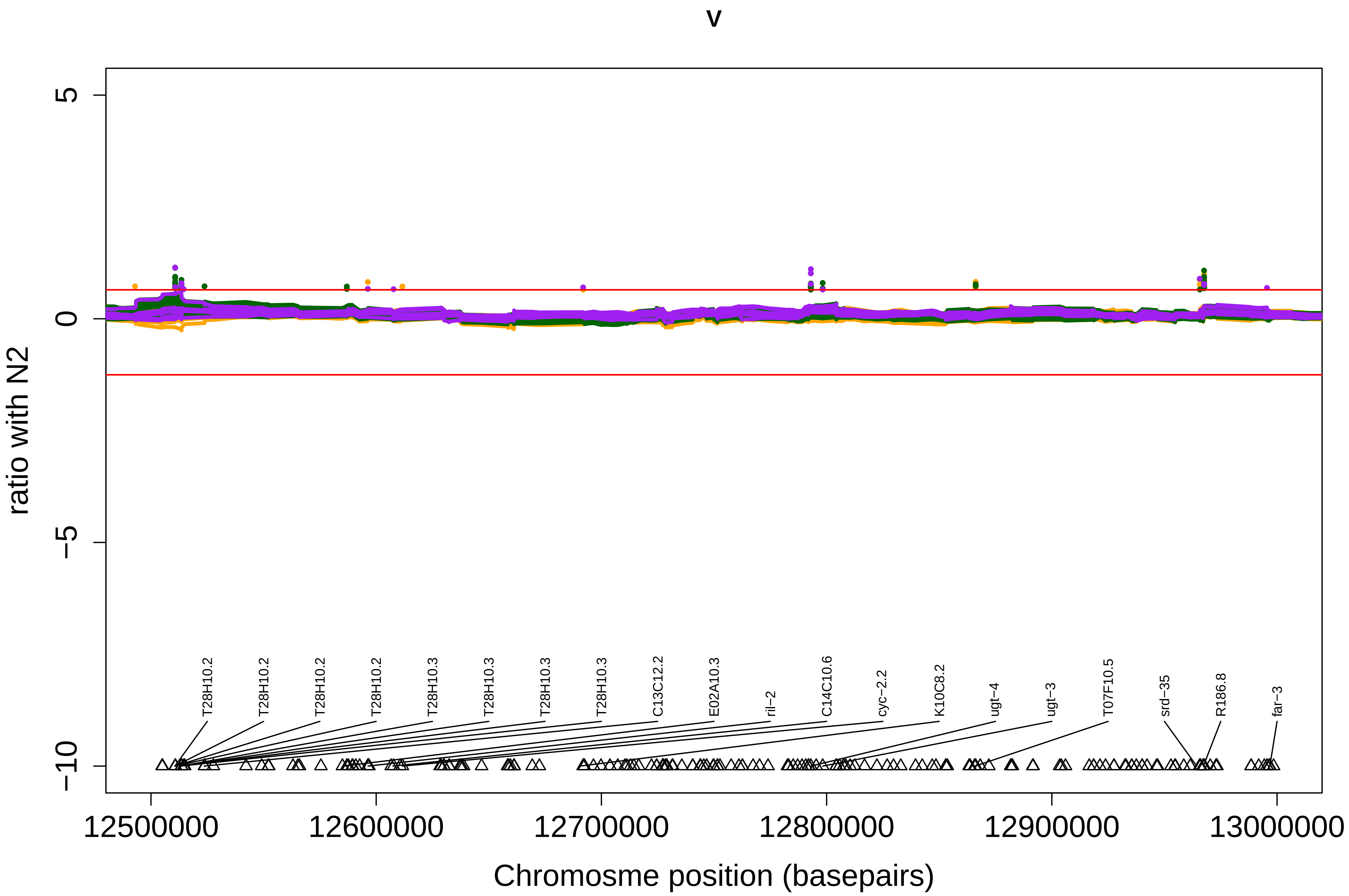
V
ratio with N2
Chromosme position (basepairs)
5
0
−5
−10
12500000
12600000
12700000
12800000
12900000
13000000
T28H10.2
T28H10.2
T28H10.2
T28H10.2
T28H10.3
T28H10.3
T28H10.3
T28H10.3
C13C12.2
E02A10.3
ril−2
C14C10.6
cyc−2.2
K10C8.2
ugt−4
ugt−3
T07F10.5
srd−35
R186.8
far−3

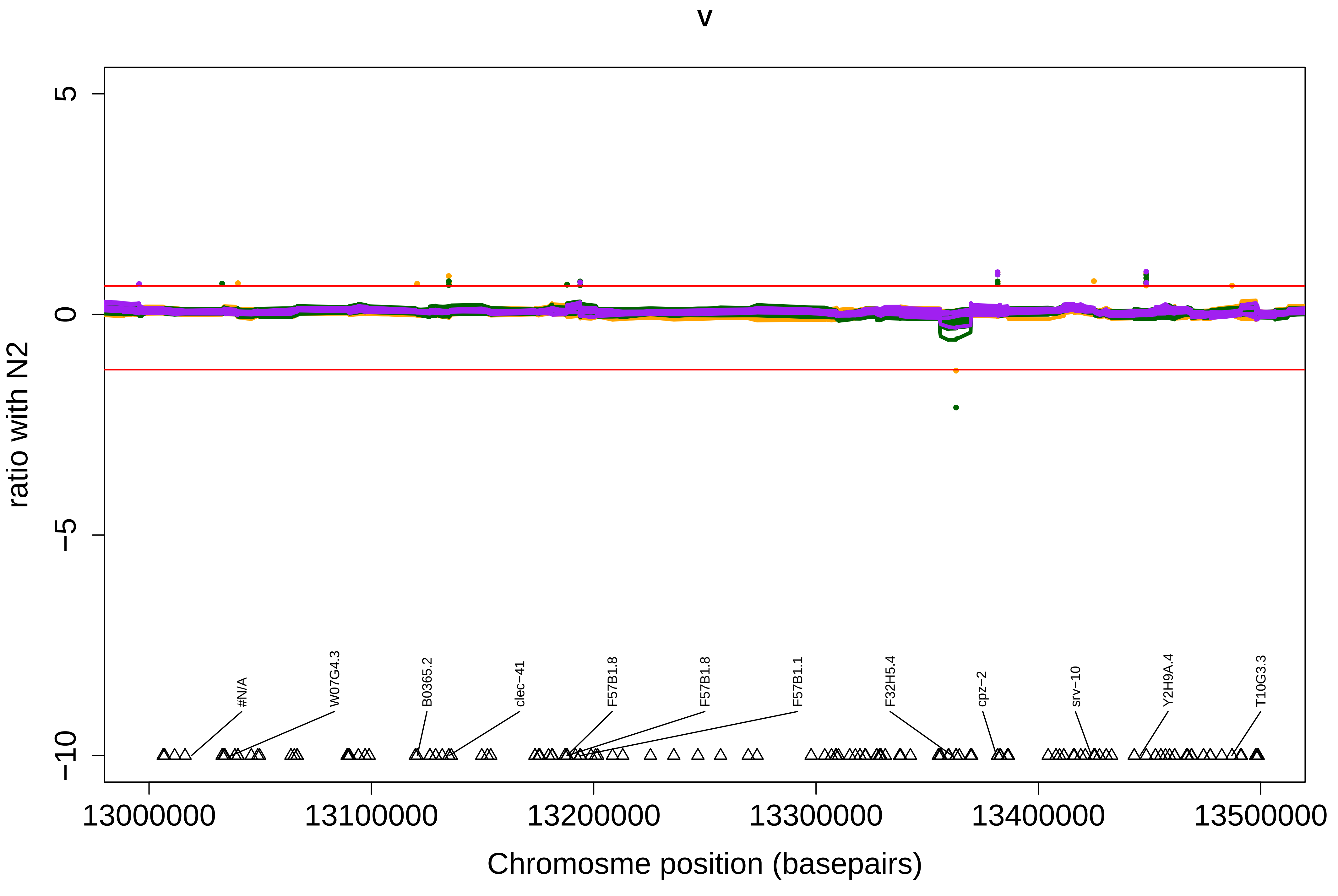
V
ratio with N2
5
0
−5
−10
13000000
13100000
13200000
13300000
13400000
13500000
Chromosme position (basepairs)
#N/A
W07G4.3
B0365.2
clec−41
F57B1.8
F57B1.8
F57B1.1
F32H5.4
cpz−2
srv−10
Y2H9A.4
T10G3.3

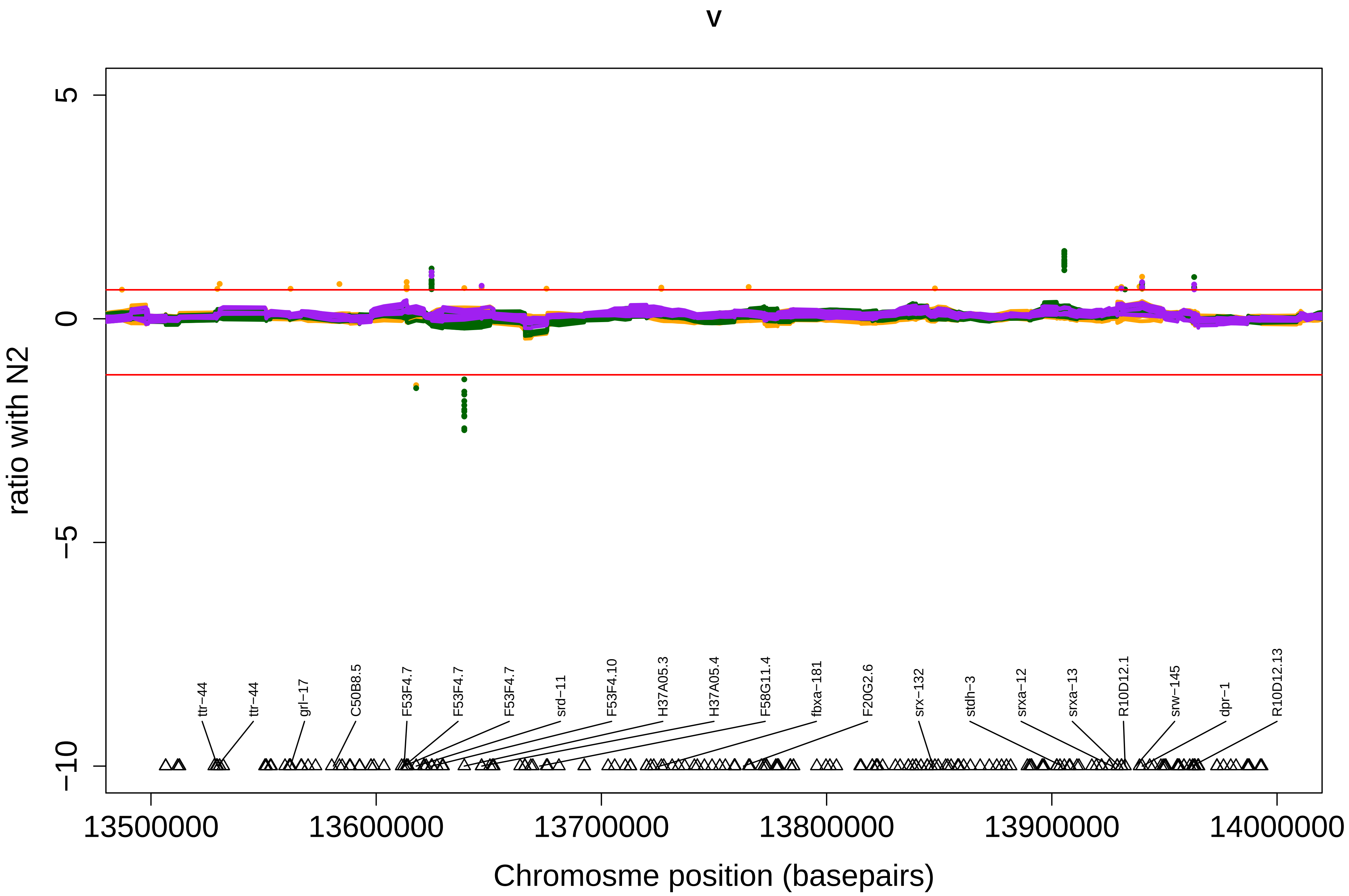
V
ratio with N2
5
0
−5
−10
ttr−44
ttr−44
grl−17
C50B8.5
F53F4.7
F53F4.7
F53F4.7
srd−11
F53F4.10
H37A05.3
H37A05.4
F58G11.4
fbxa−181
F20G2.6
srx−132
stdh−3
srxa−12
srxa−13
R10D12.1
srw−145
dpr−1
R10D12.13
13500000
13600000
13700000
13800000
13900000
14000000
Chromosme position (basepairs)

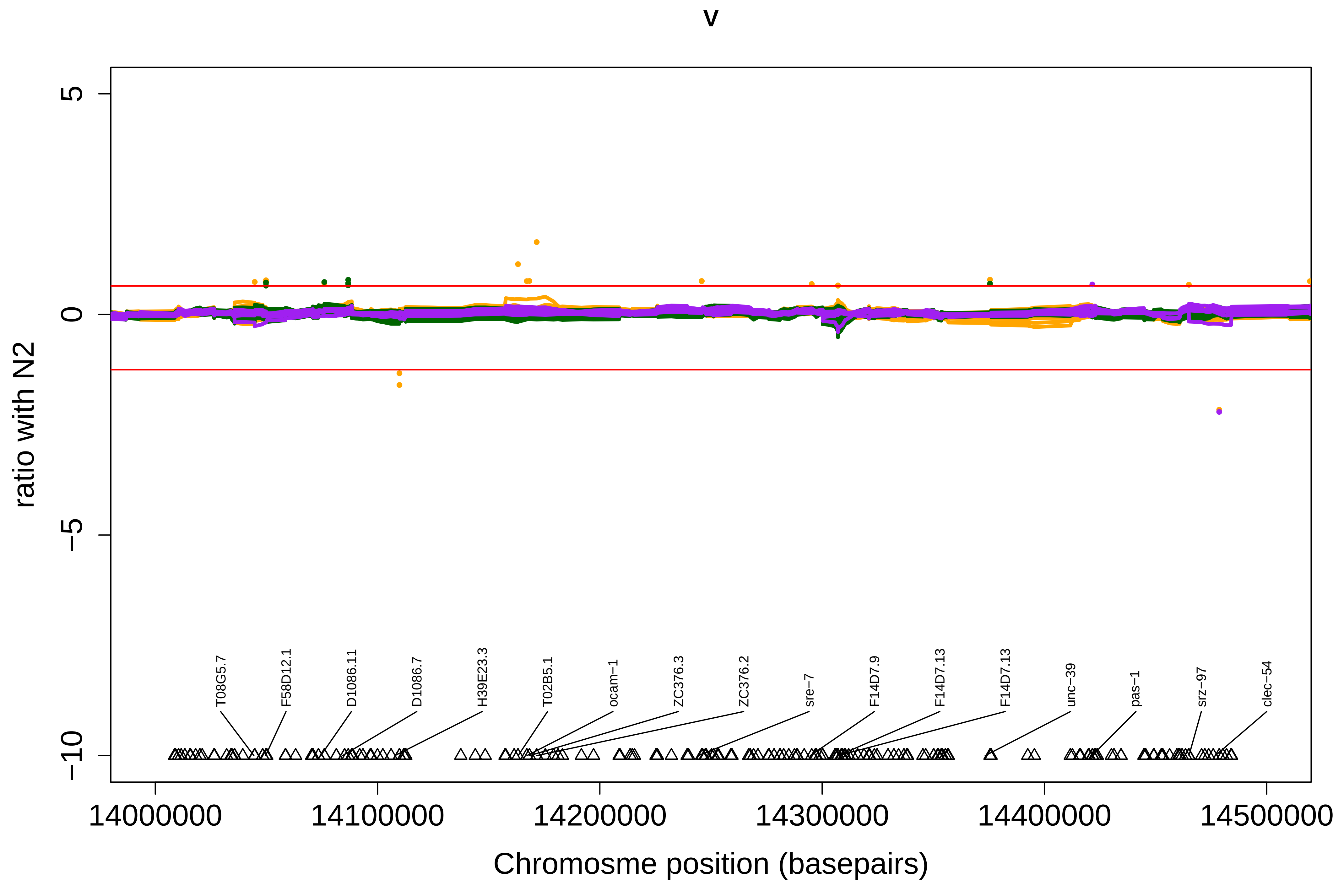

V
ratio with N2
5
0
−5
−10
T08G5.7
F58D12.1
D1086.11
D1086.7
H39E23.3
T02B5.1
ocam−1
ZC376.3
ZC376.2
sre−7
F14D7.9
F14D7.13
F14D7.13
unc−39
pas−1
srz−97
clec−54
14000000
14100000
14200000
14300000
14400000
14500000
Chromosme position (basepairs)

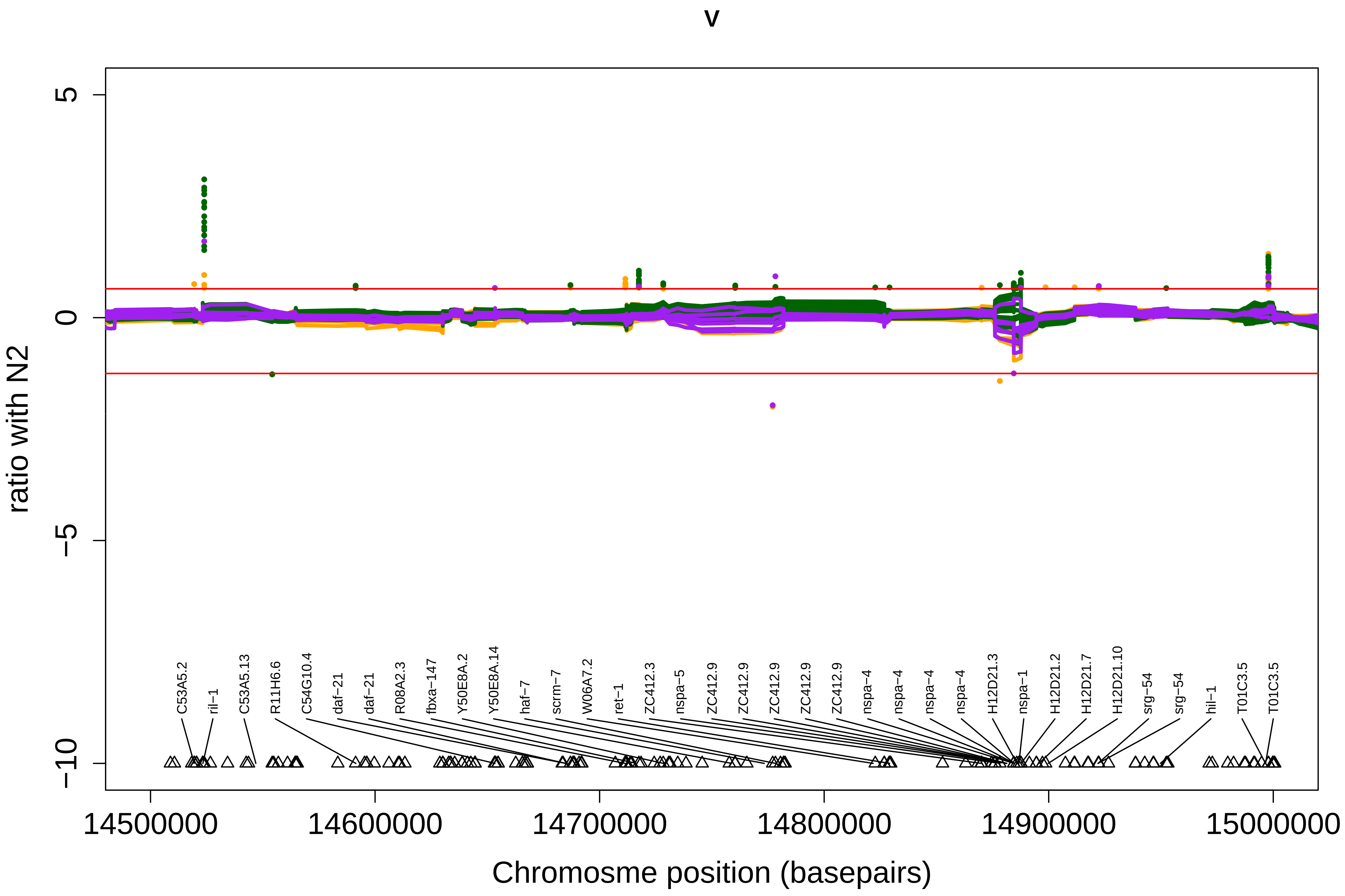

V
ratio with N2
5
0
−5
−10
Chromosme position (basepairs)
14500000
14600000
14700000
14800000
14900000
15000000
C53A5.2
ril−1
C53A5.13
R11H6.6
C54G10.4
daf−21
daf−21
R08A2.3
fbxa−147
Y50E8A.2
Y50E8A.14
haf−7
scrm−7
W06A7.2
ret−1
ZC412.3
nspa−5
ZC412.9
ZC412.9
ZC412.9
ZC412.9
ZC412.9
nspa−4
nspa−4
nspa−4
nspa−4
H12D21.3
nspa−1
H12D21.2
H12D21.7
H12D21.10
srg−54
srg−54
hil−1
T01C3.5
T01C3.5

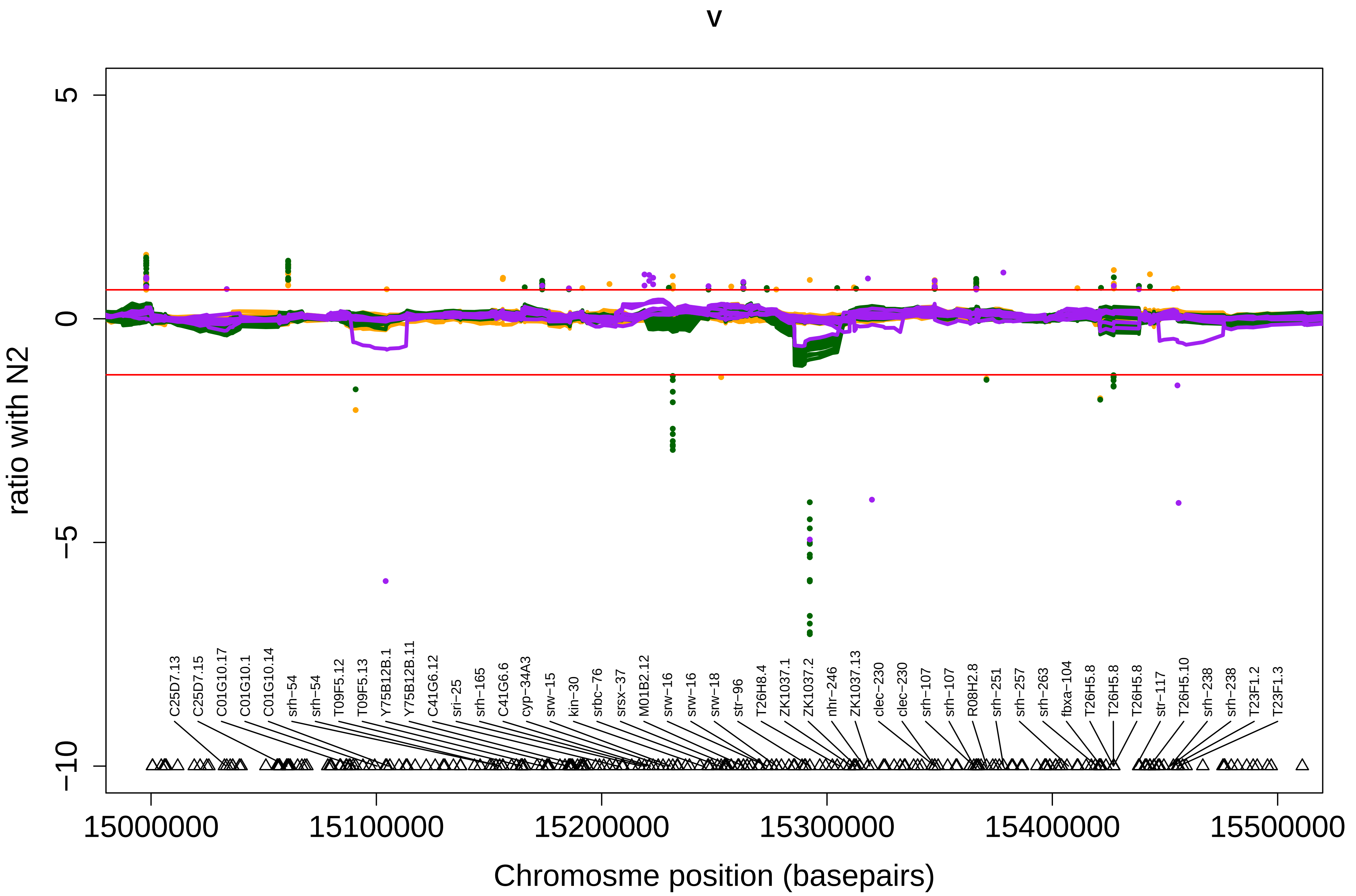
V
ratio with N2
Chromosme position (basepairs)
5
0
−5
−10
15000000
15100000
15200000
15300000
15400000
15500000
C25D7.13
C25D7.15
C01G10.17
C01G10.1
C01G10.14
srh−54
srh−54
T09F5.12
T09F5.13
Y75B12B.1
Y75B12B.11
C41G6.12
sri−25
srh−165
C41G6.6
cyp−34A3
srw−15
kin−30
srbc−76
srsx−37
M01B2.12
srw−16
srw−16
srw−18
str−96
T26H8.4
ZK1037.1
ZK1037.2
nhr−246
ZK1037.13
clec−230
clec−230
srh−107
srh−107
R08H2.8
srh−251
srh−257
srh−263
fbxa−104
T26H5.8
T26H5.8
T26H5.8
str−117
T26H5.10
srh−238
srh−238
T23F1.2
T23F1.3

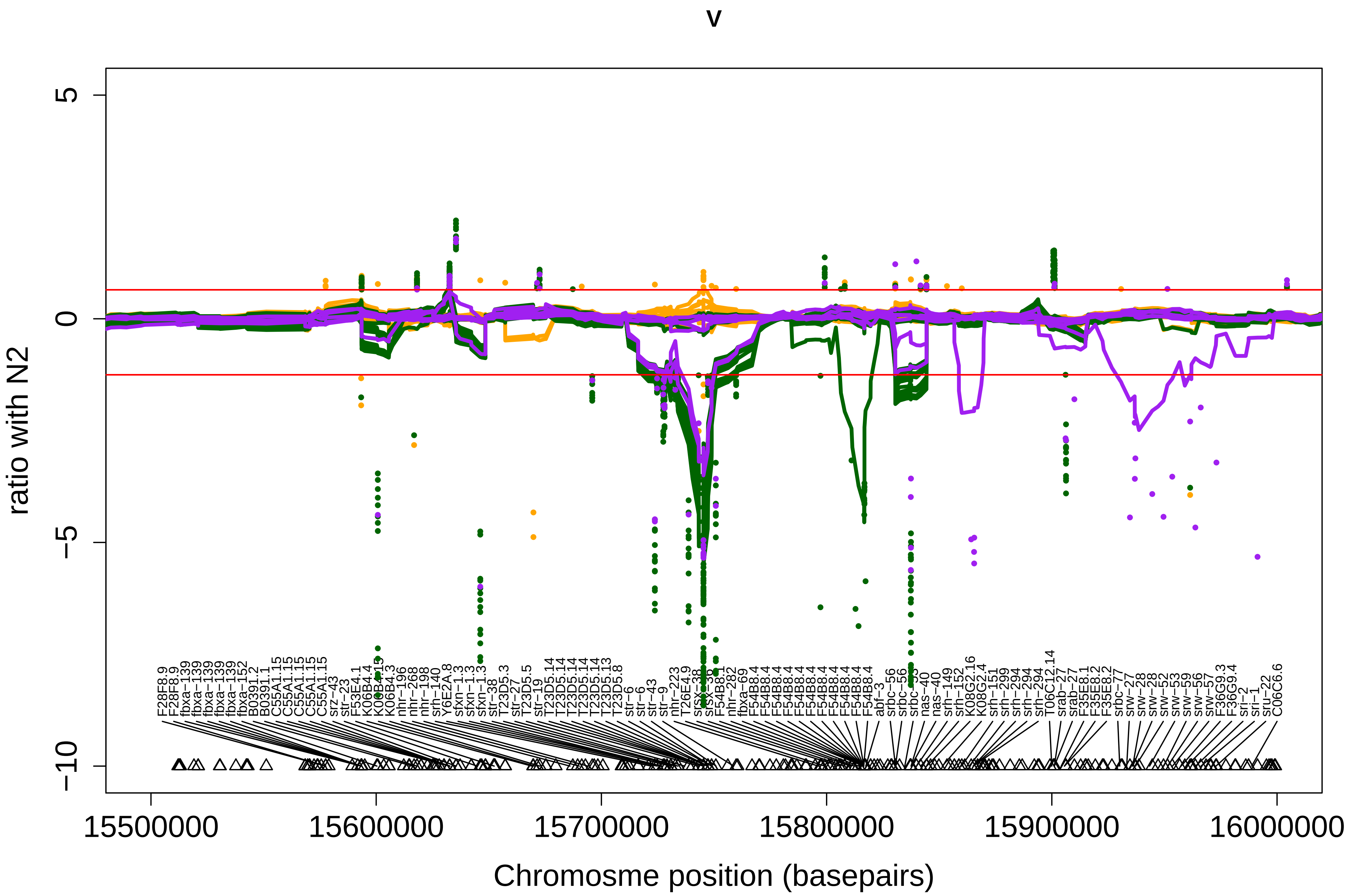
V
ratio with N2
5
0
−5
−10
15500000
15600000
15700000
15800000
15900000
16000000
Chromosme position (basepairs)

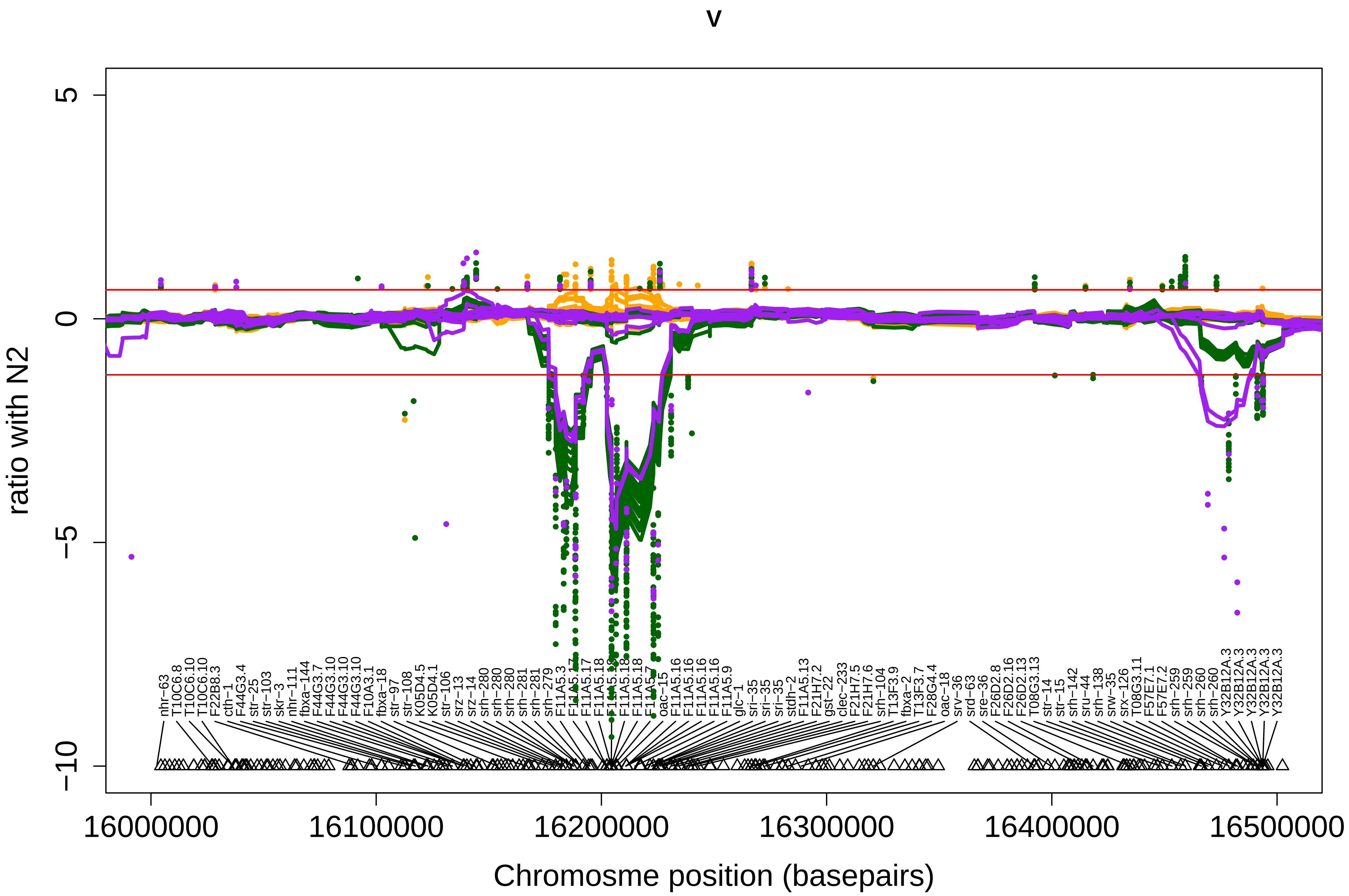

V
ratio with N2
Chromosme position (basepairs)
5
0
−5
−10
16000000
16100000
16200000
16300000
16400000
16500000

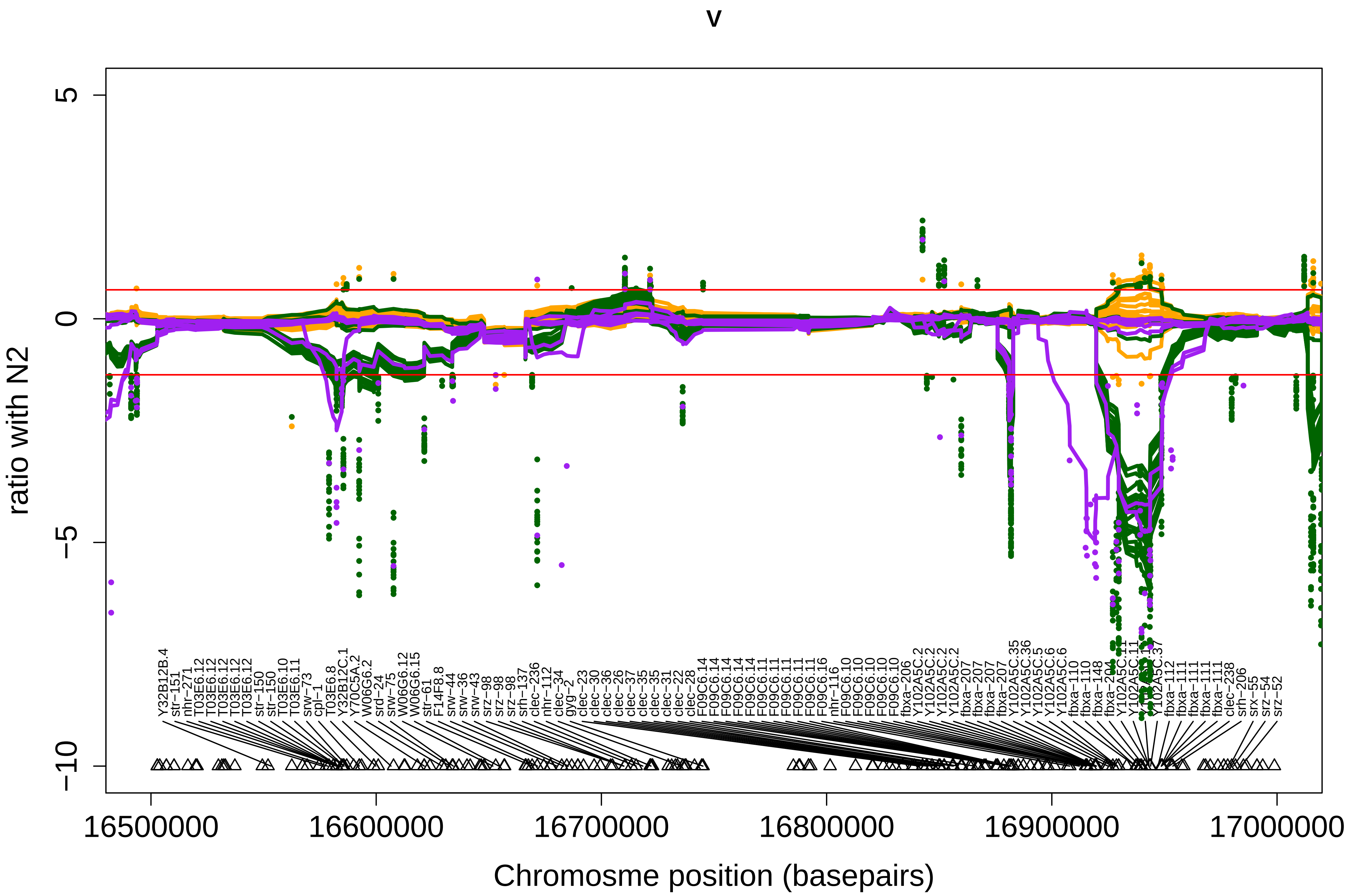
V
ratio with N2
5
0
−5
−10
16500000
16600000
16700000
16800000
16900000
17000000
Chromosme position (basepairs)

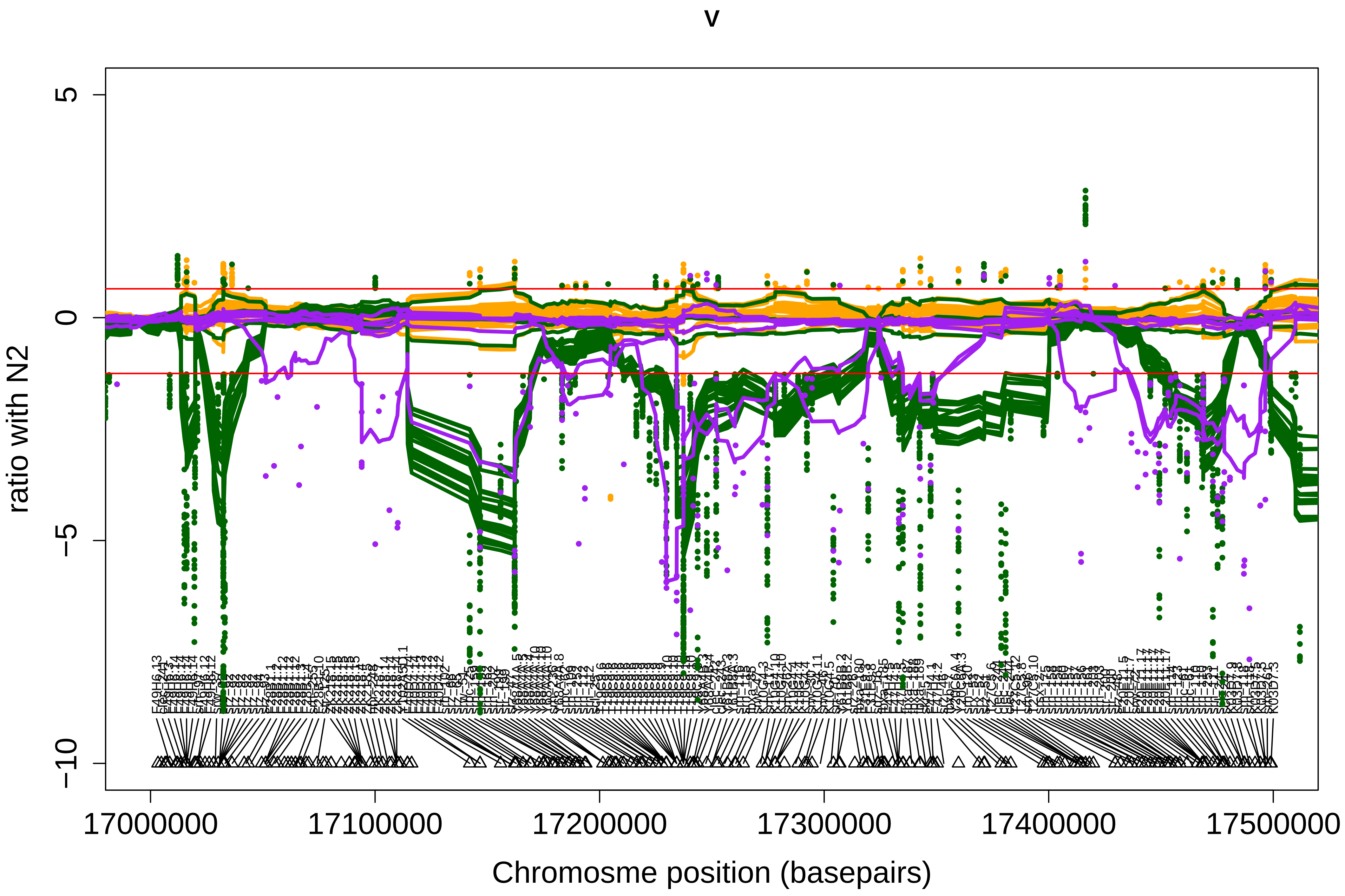
V
ratio with N2
5
0
−5
−10
17000000
17100000
17200000
17300000
17400000
17500000
Chromosme position (basepairs)

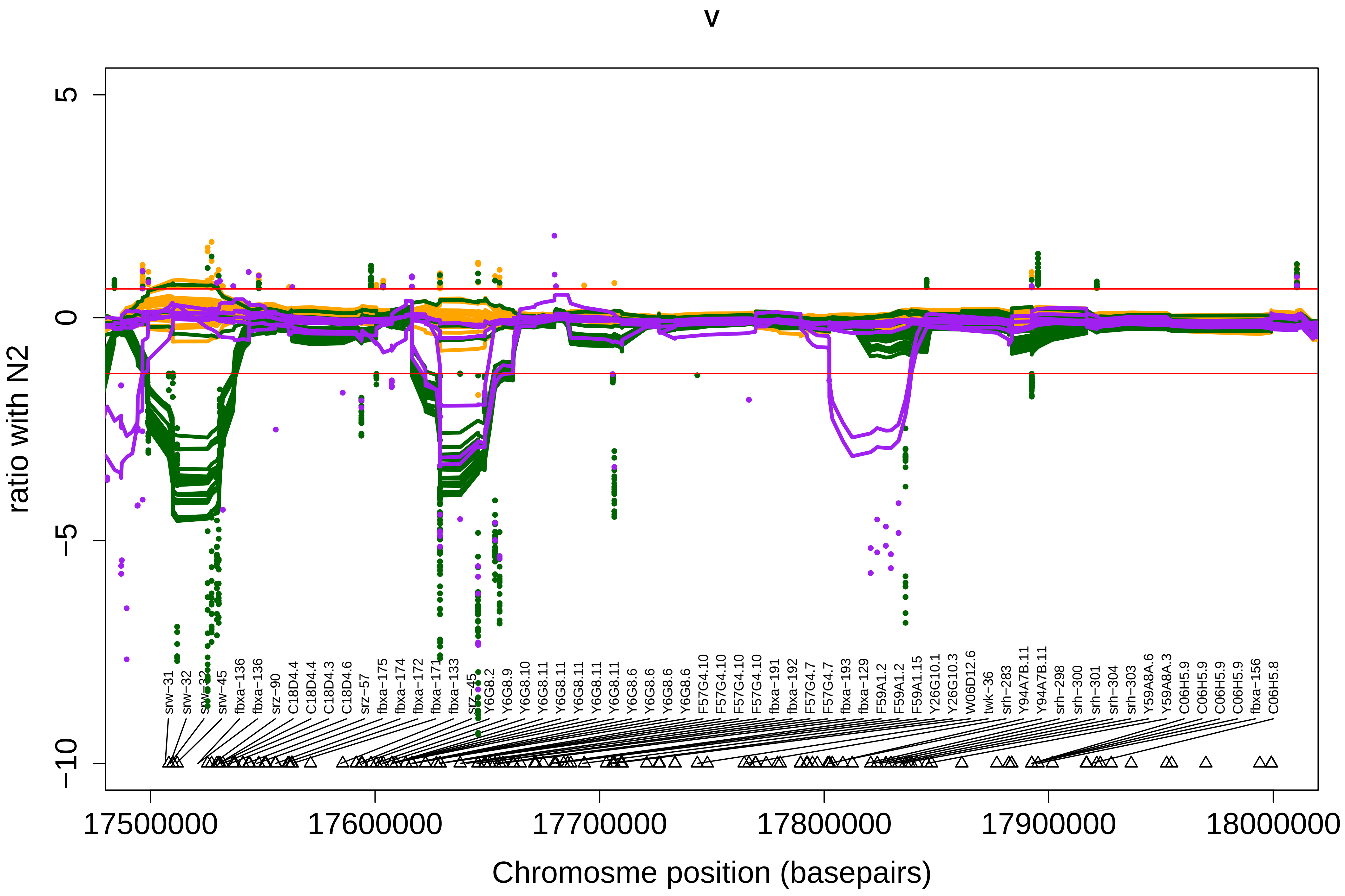

V
ratio with N2
5
0
−5
−10
17500000
17600000
17700000
17800000
17900000
18000000
Chromosme position (basepairs)
srw−31
srw−32
srw−45
fbxa−136
fbxa−136
srz−90
C18D4.4
C18D4.4
C18D4.3
C18D4.6
srz−57
fbxa−175
fbxa−174
fbxa−172
fbxa−171
fbxa−133
srz−45
Y6G8.2
Y6G8.9
Y6G8.10
Y6G8.11
Y6G8.11
Y6G8.11
Y6G8.11
Y6G8.11
Y6G8.6
Y6G8.6
Y6G8.6
Y6G8.6
F57G4.10
F57G4.10
F57G4.10
F57G4.10
fbxa−191
fbxa−192
F57G4.7
F57G4.7
fbxa−193
fbxa−129
F59A1.2
F59A1.2
F59A1.15
Y26G10.1
Y26G10.3
W06D12.6
twk−36
srh−283
Y94A7B.11
Y94A7B.11
srh−298
srh−300
srh−301
srh−304
srh−303
Y59A8A.6
Y59A8A.3
C06H5.9
C06H5.9
C06H5.9
C06H5.9
fbxa−156
C06H5.8

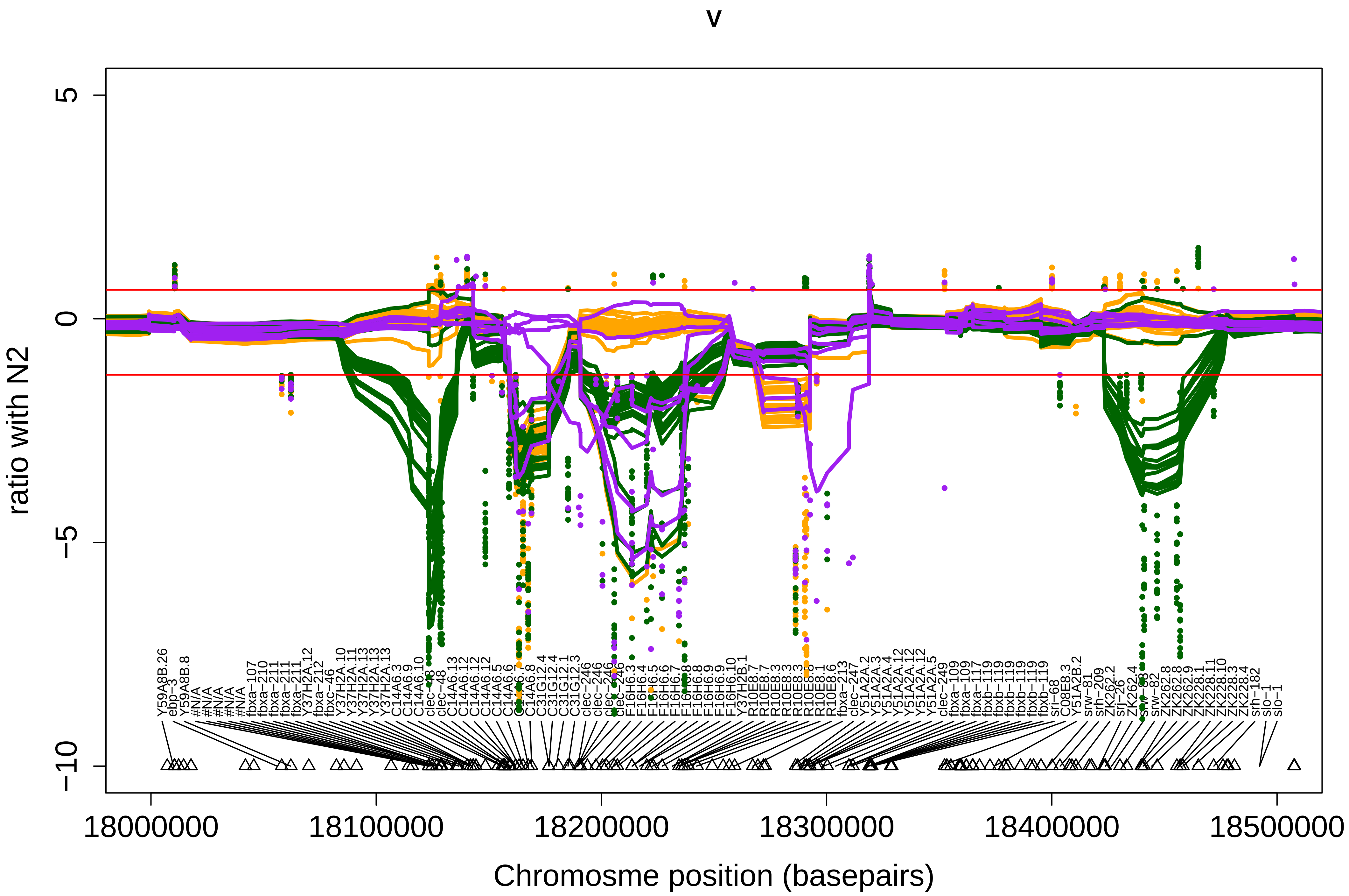

V
ratio with N2
Chromosme position (basepairs)
5
0
−5
−10
18000000
18100000
18200000
18300000
18400000
18500000

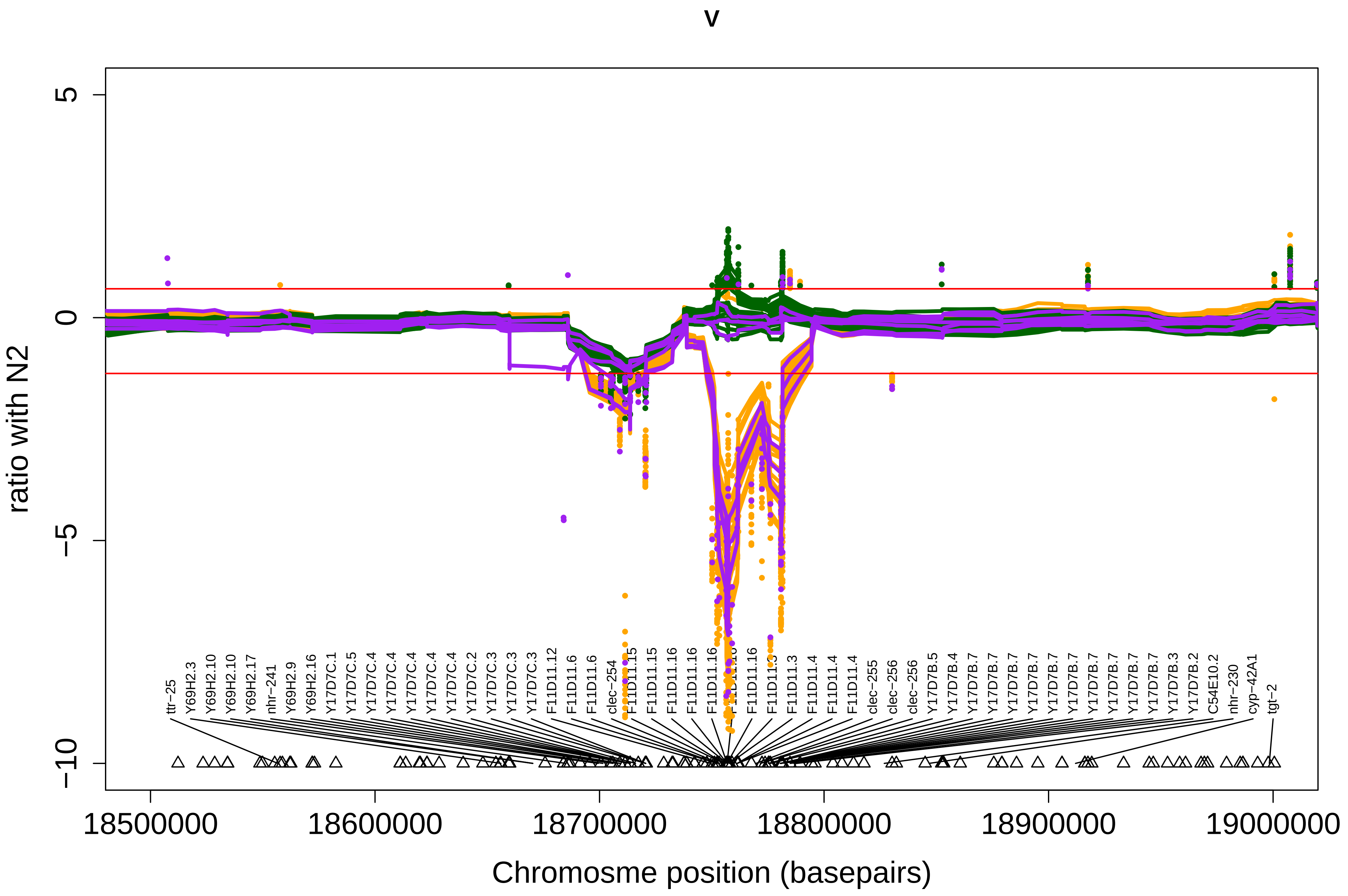
V
ratio with N2
Chromosme position (basepairs)
5
0
−5
−10
18500000
18600000
18700000
18800000
18900000
19000000
ttr−25
Y69H2.3
Y69H2.10
Y69H2.10
Y69H2.17
nhr−241
Y69H2.9
Y69H2.16
Y17D7C.1
Y17D7C.5
Y17D7C.4
Y17D7C.4
Y17D7C.4
Y17D7C.4
Y17D7C.4
Y17D7C.2
Y17D7C.3
Y17D7C.3
Y17D7C.3
F11D11.12
F11D11.6
F11D11.6
clec−254
F11D11.15
F11D11.16
F11D11.16
F11D11.16
F11D11.16
F11D11.3
F11D11.4
F11D11.4
F11D11.4
clec−255
clec−256
clec−256
Y17D7B.5
Y17D7B.4
Y17D7B.7
Y17D7B.7
Y17D7B.7
Y17D7B.7
Y17D7B.7
Y17D7B.7
Y17D7B.7
Y17D7B.7
Y17D7B.7
Y17D7B.7
Y17D7B.3
Y17D7B.2
C54E10.2
nhr−230
cyp−42A1
tgt−2

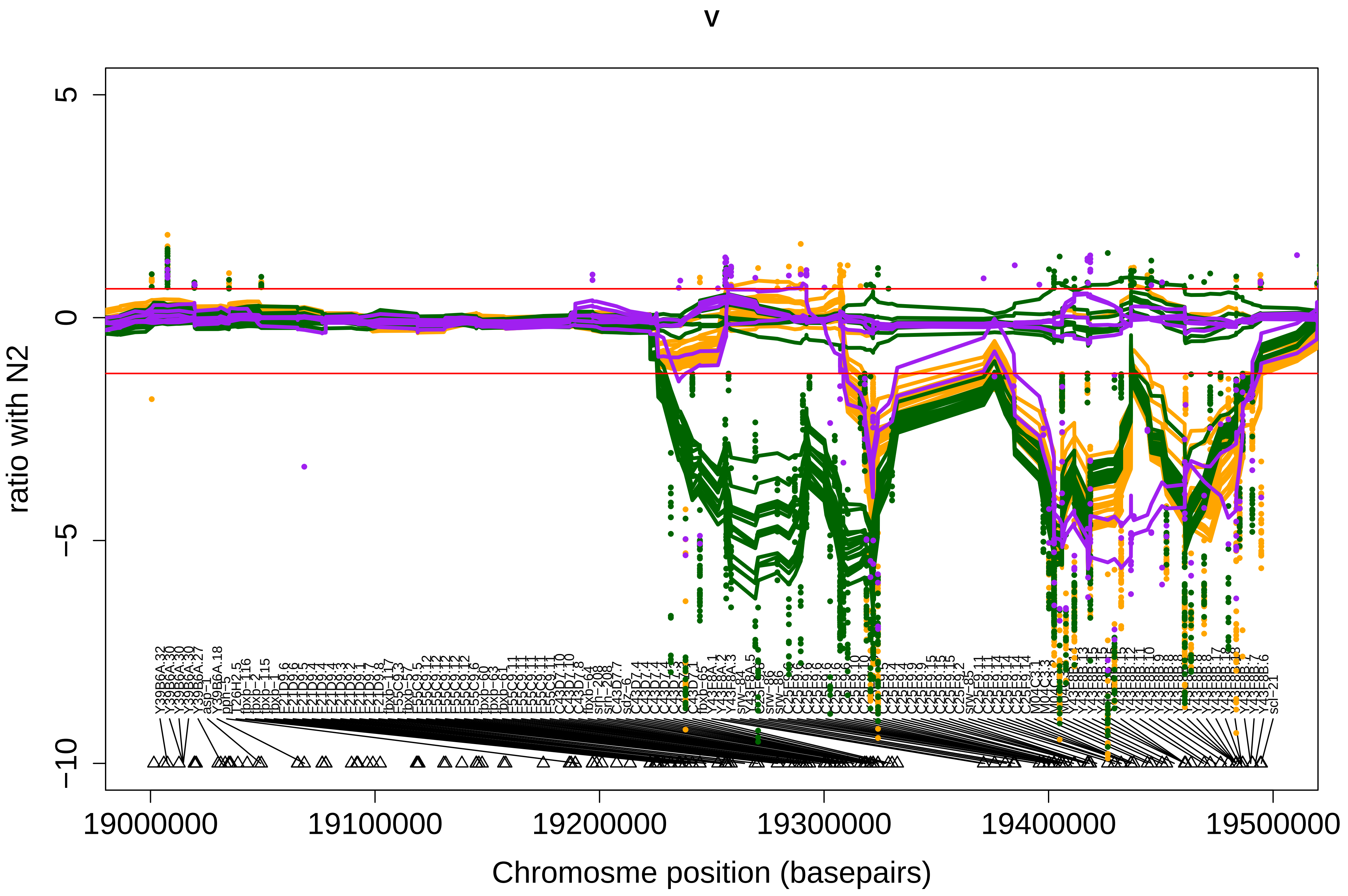
V
ratio with N2
5
0
−5
−10
19000000
19100000
19200000
19300000
19400000
19500000
Chromosme position (basepairs)

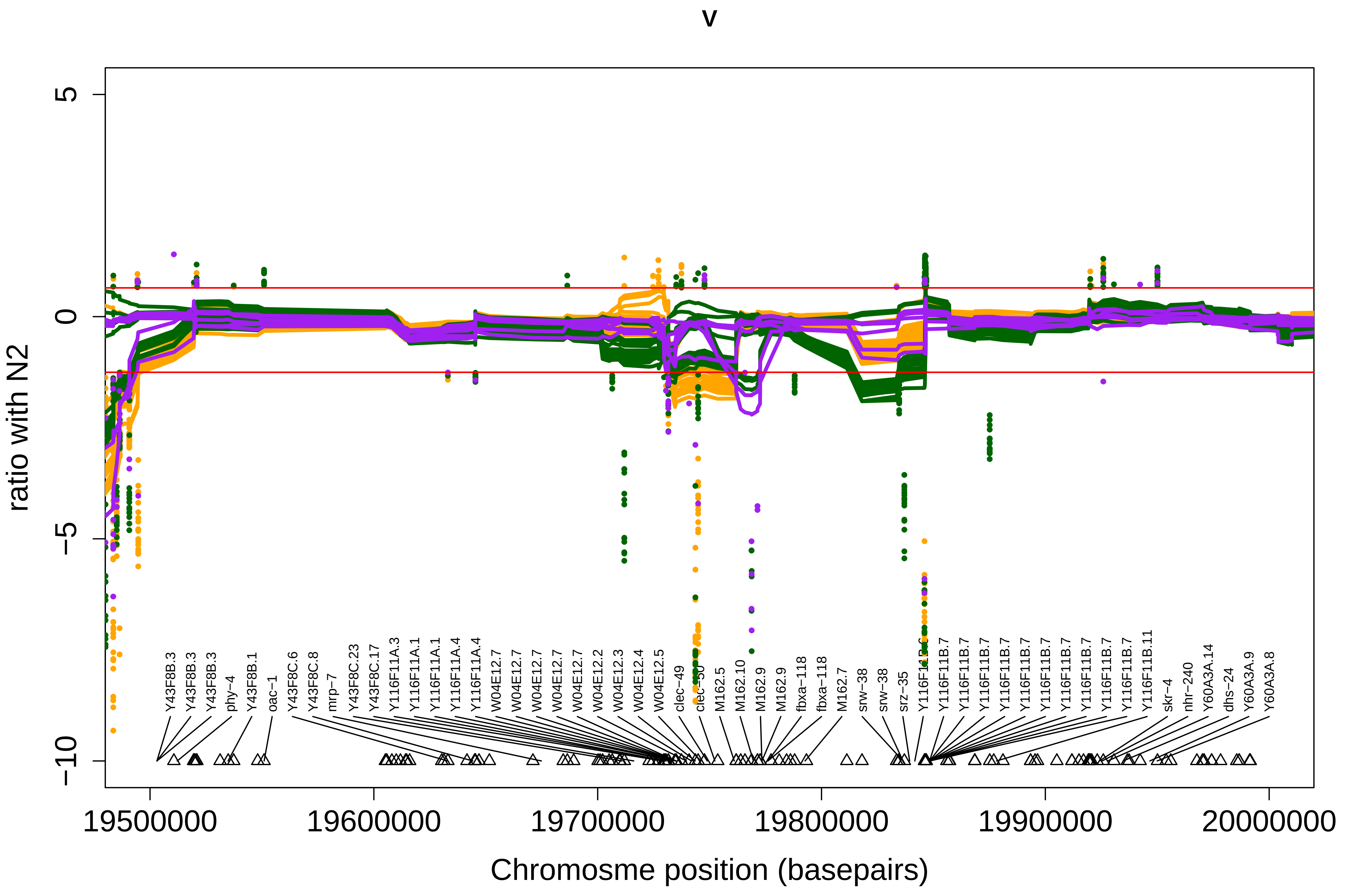
V
ratio with N2
Chromosme position (basepairs)
5
0
−5
−10
19500000
19600000
19700000
19800000
19900000
20000000
Y43F8B.3
Y43F8B.3
Y43F8B.3
phy−4
Y43F8B.1
oac−1
Y43F8C.6
Y43F8C.8
mrp−7
Y43F8C.23
Y43F8C.17
Y116F11A.3
Y116F11A.1
Y116F11A.1
Y116F11A.4
Y116F11A.4
W04E12.7
W04E12.7
W04E12.7
W04E12.7
W04E12.7
W04E12.2
W04E12.3
W04E12.4
W04E12.5
clec−49
clec−50
M162.5
M162.10
M162.9
M162.9
fbxa−118
fbxa−118
M162.7
srw−38
srw−38
srz−35
Y116F11B.7
Y116F11B.7
Y116F11B.7
Y116F11B.7
Y116F11B.7
Y116F11B.7
Y116F11B.7
Y116F11B.7
Y116F11B.7
Y116F11B.11
skr−4
nhr−240
Y60A3A.14
dhs−24
Y60A3A.9
Y60A3A.8

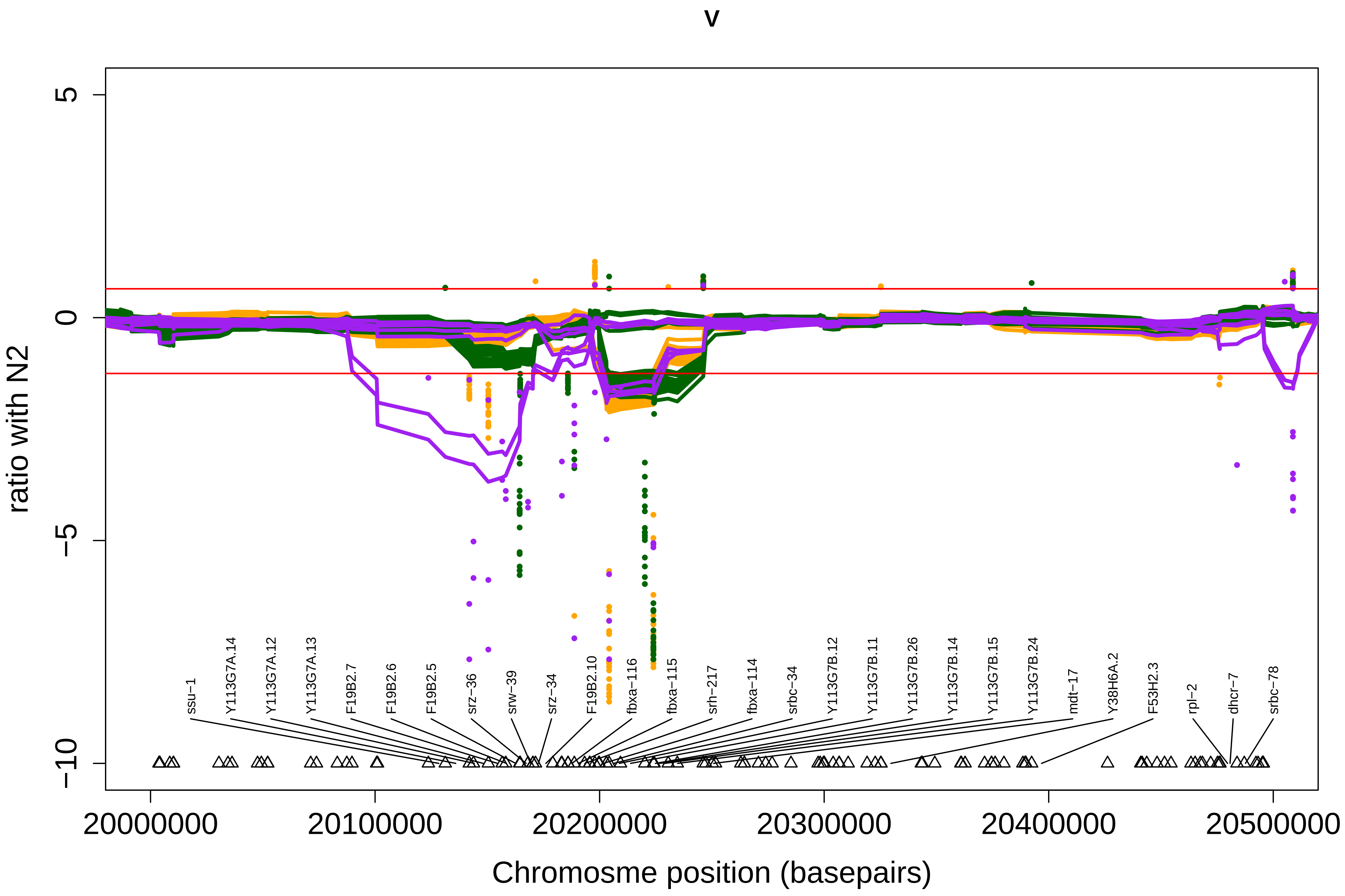
V
ratio with N2
5
0
−5
−10
ssu−1
Y113G7A.14
Y113G7A.12
Y113G7A.13
F19B2.7
F19B2.6
F19B2.5
srz−36
srw−39
srz−34
F19B2.10
fbxa−116
fbxa−115
srh−217
fbxa−114
srbc−34
Y113G7B.12
Y113G7B.11
Y113G7B.26
Y113G7B.14
Y113G7B.15
Y113G7B.24
mdt−17
Y38H6A.2
F53H2.3
rpl−2
dhcr−7
srbc−78
20000000
20100000
20200000
20300000
20400000
20500000
Chromosme position (basepairs)

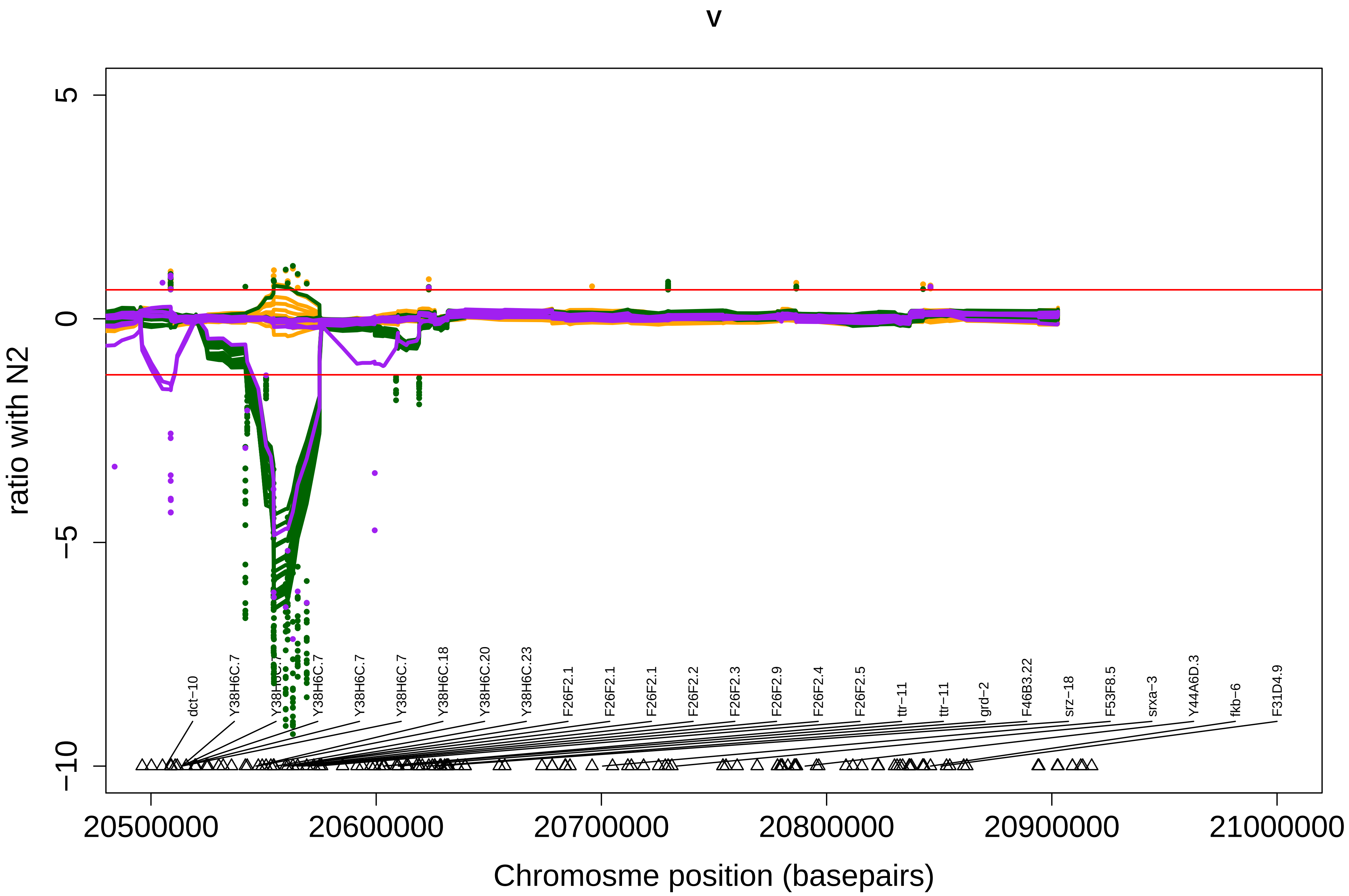
V
ratio with N2
Chromosme position (basepairs)
5
0
−5
−10
20500000
20600000
20700000
20800000
20900000
21000000
dct−10
Y38H6C.7
Y38H6C.7
Y38H6C.7
Y38H6C.7
Y38H6C.7
Y38H6C.18
Y38H6C.20
Y38H6C.23
F26F2.1
F26F2.1
F26F2.1
F26F2.2
F26F2.3
F26F2.9
F26F2.4
F26F2.5
ttr−11
ttr−11
grd−2
F46B3.22
srz−18
F53F8.5
srxa−3
Y44A6D.3
fkb−6
F31D4.9

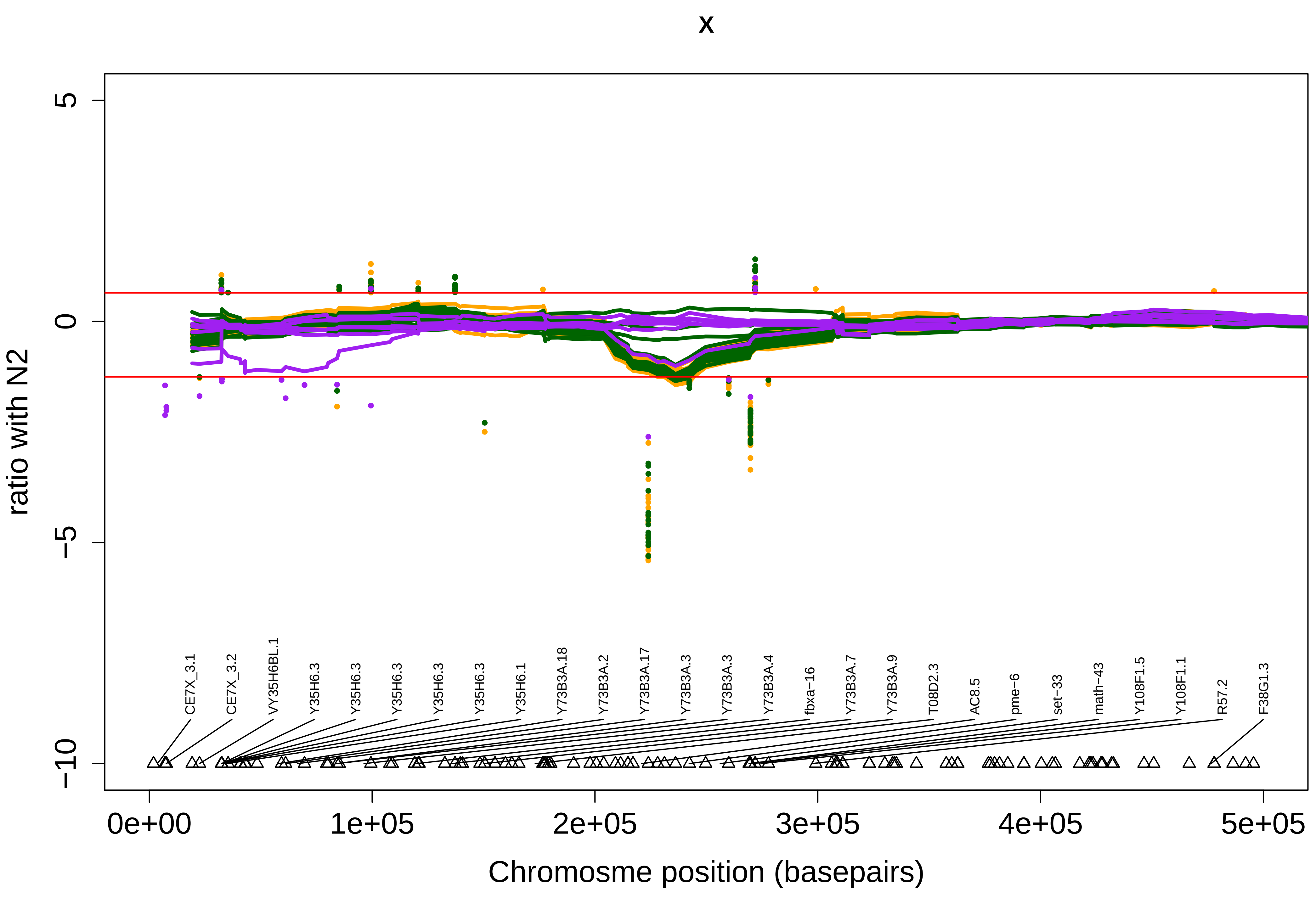
X
ratio with N2
Chromosme position (basepairs)
5
0
−5
−10
0e+00
1e+05
2e+05
3e+05
4e+05
5e+05
CE7X_3.1
CE7X_3.2
VY35H6BL.1
Y35H6.3
Y35H6.3
Y35H6.3
Y35H6.3
Y35H6.3
Y35H6.1
Y73B3A.18
Y73B3A.2
Y73B3A.17
Y73B3A.3
Y73B3A.3
Y73B3A.4
fbxa−16
Y73B3A.7
Y73B3A.9
T08D2.3
AC8.5
pme−6
set−33
math−43
Y108F1.5
Y108F1.1
R57.2
F38G1.3

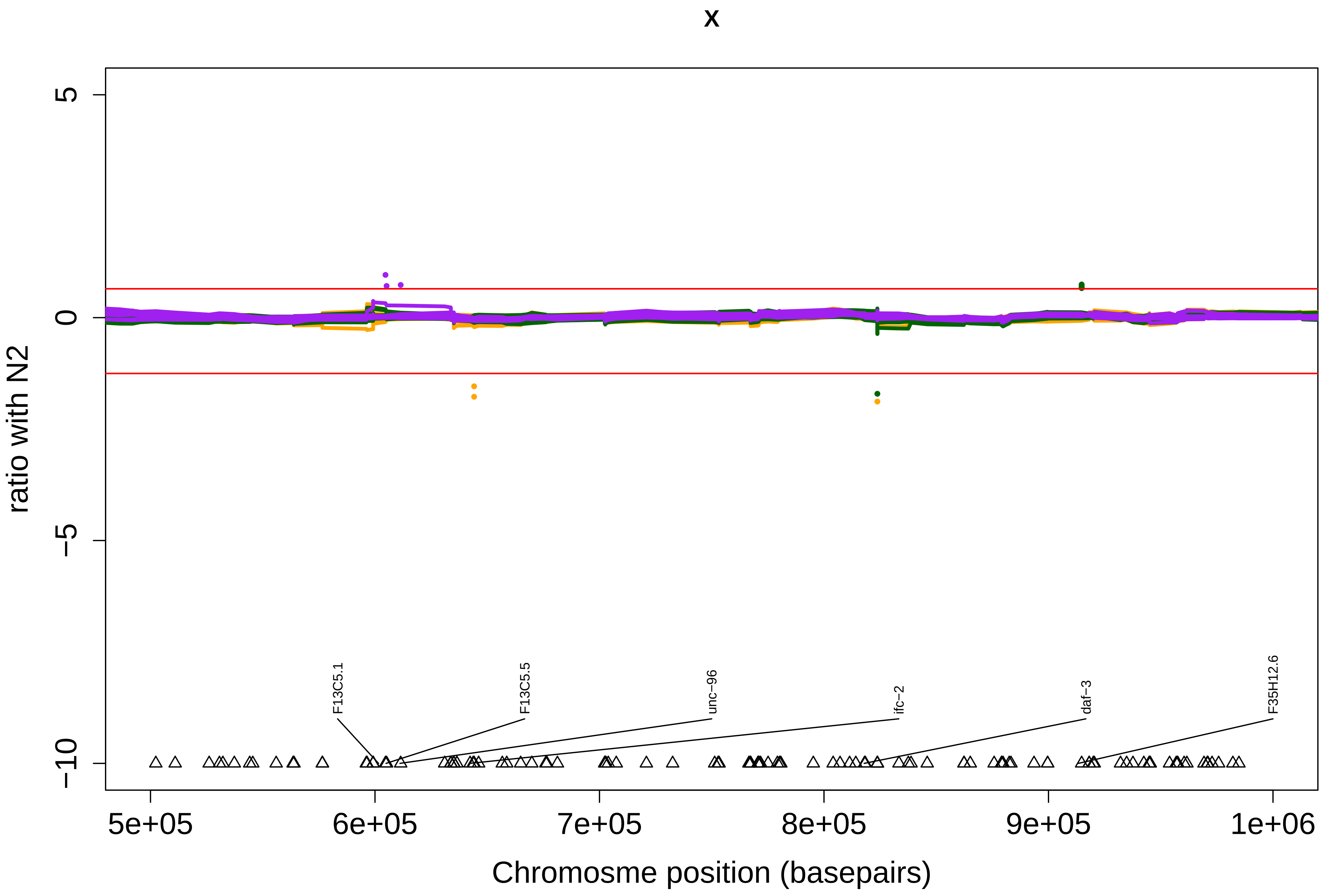
X
ratio with N2
5
0
−5
−10
5e+05
6e+05
7e+05
8e+05
9e+05
1e+06
Chromosme position (basepairs)
F13C5.1
F13C5.5
unc−96
ifc−2
daf−3
F35H12.6

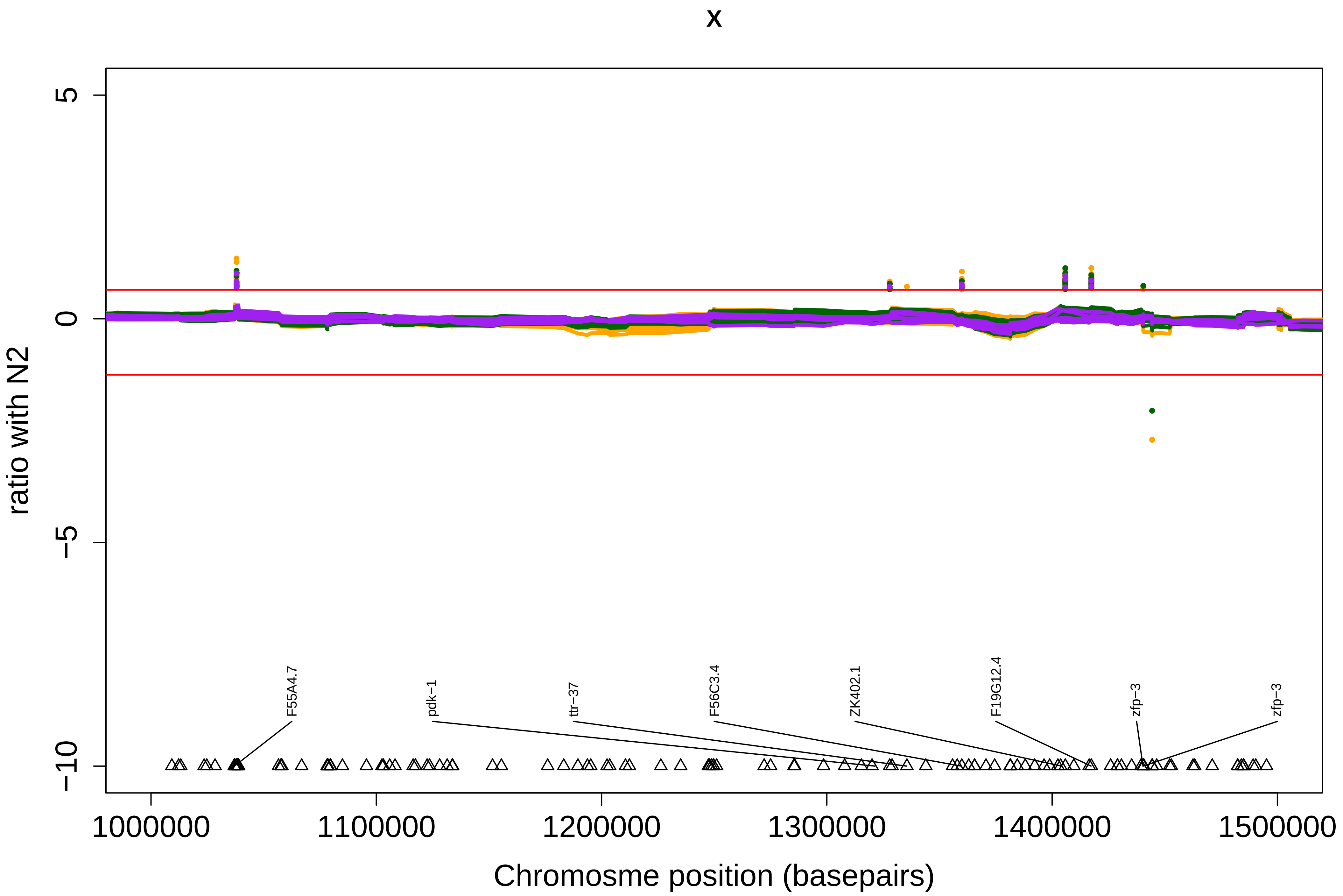
X
ratio with N2
5
0
−5
−10
F55A4.7
pdk−1
ttr−37
F56C3.4
ZK402.1
F19G12.4
zfp−3
zfp−3
1000000
1100000
1200000
1300000
1400000
1500000
Chromosme position (basepairs)

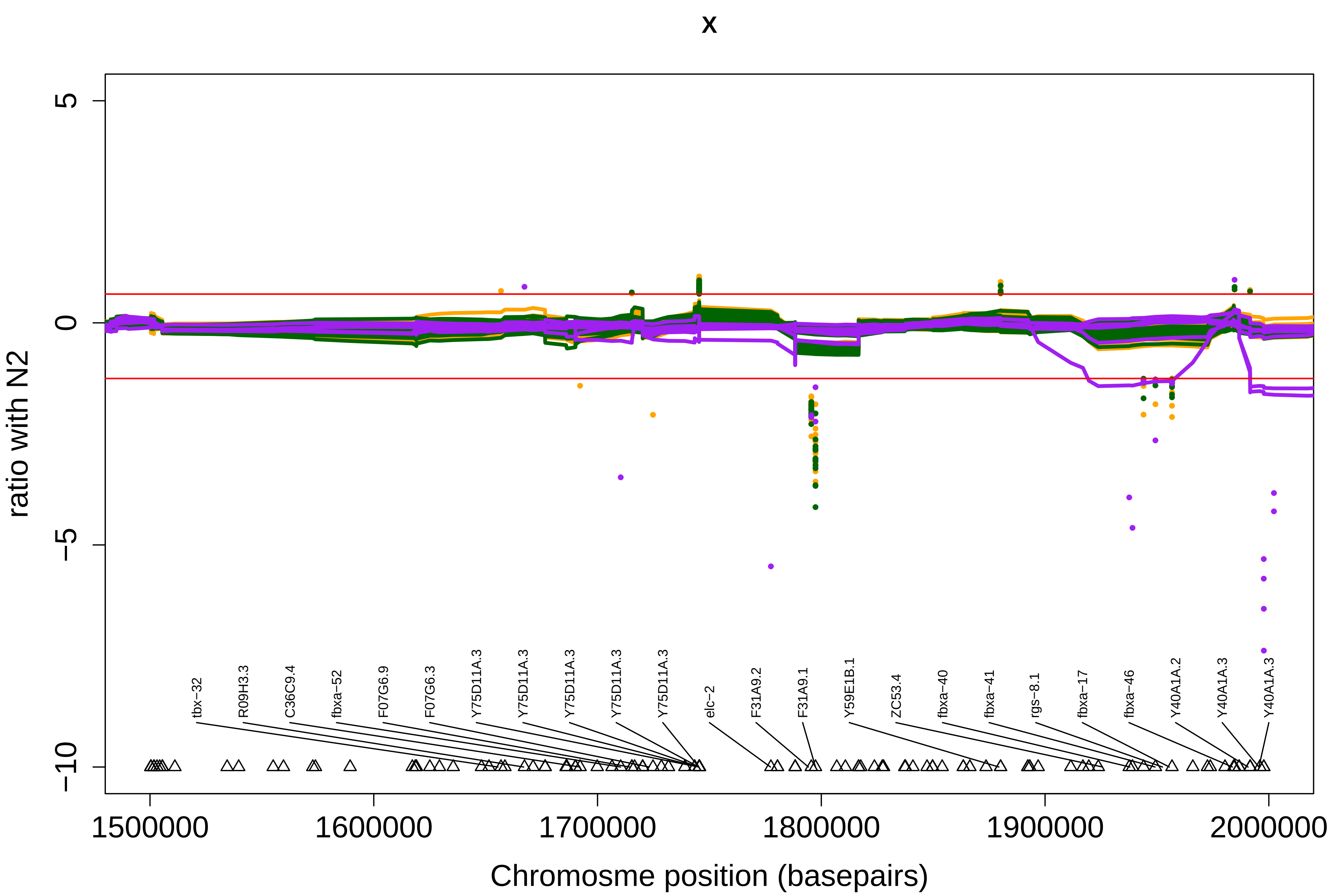

X
ratio with N2
5
0
−5
−10
1500000
1600000
1700000
1800000
1900000
2000000
Chromosme position (basepairs)
tbx−32
R09H3.3
C36C9.4
fbxa−52
F07G6.9
F07G6.3
Y75D11A.3
Y75D11A.3
Y75D11A.3
Y75D11A.3
Y75D11A.3
elc−2
F31A9.2
F31A9.1
Y59E1B.1
ZC53.4
fbxa−40
fbxa−41
rgs−8.1
fbxa−17
fbxa−46
Y40A1A.2
Y40A1A.3
Y40A1A.3

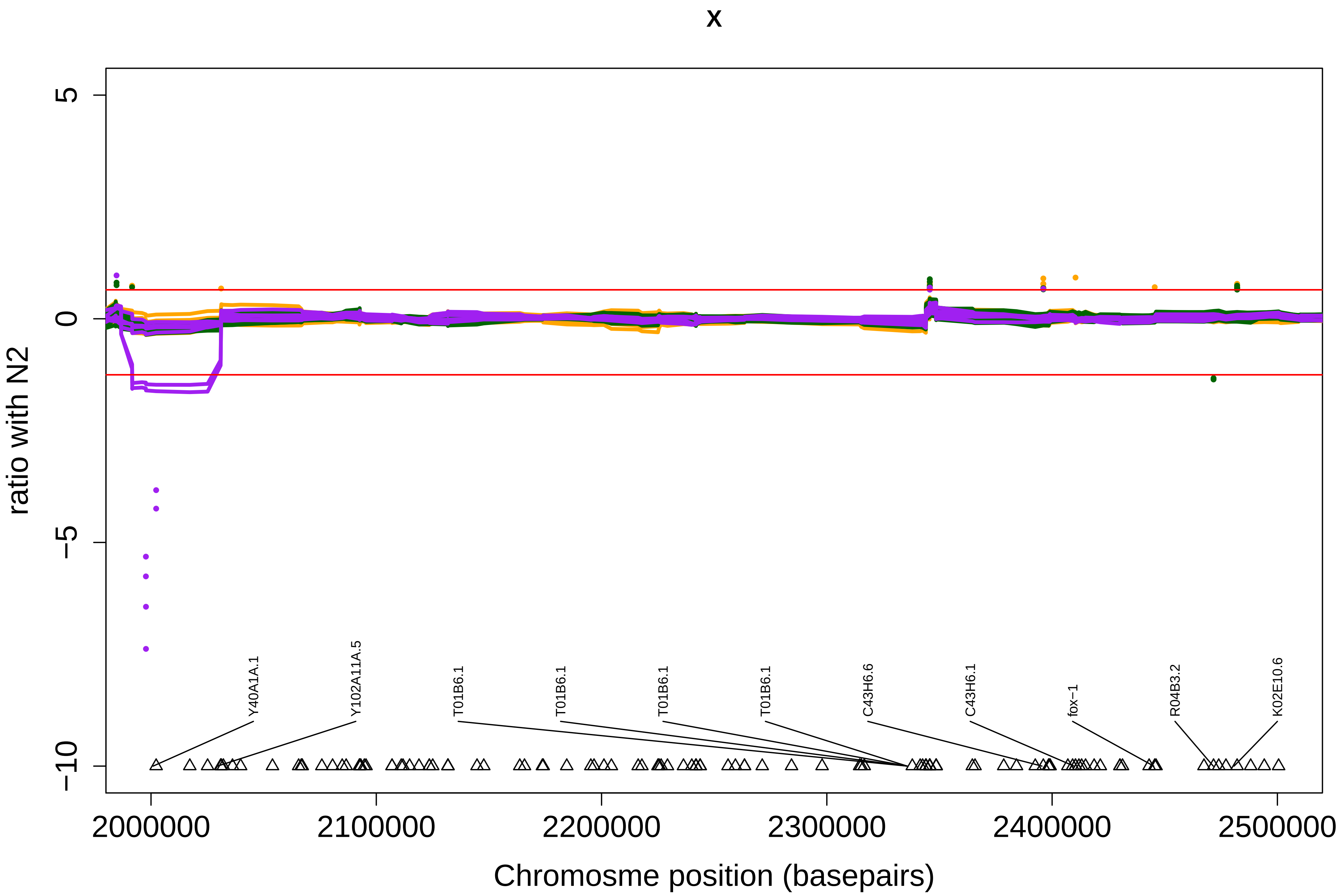
X
ratio with N2
5
0
−5
−10
Chromosme position (basepairs)
2000000
2100000
2200000
2300000
2400000
2500000
Y40A1A.1
Y102A11A.5
T01B6.1
T01B6.1
T01B6.1
T01B6.1
C43H6.6
C43H6.1
fox−1
R04B3.2
K02E10.6

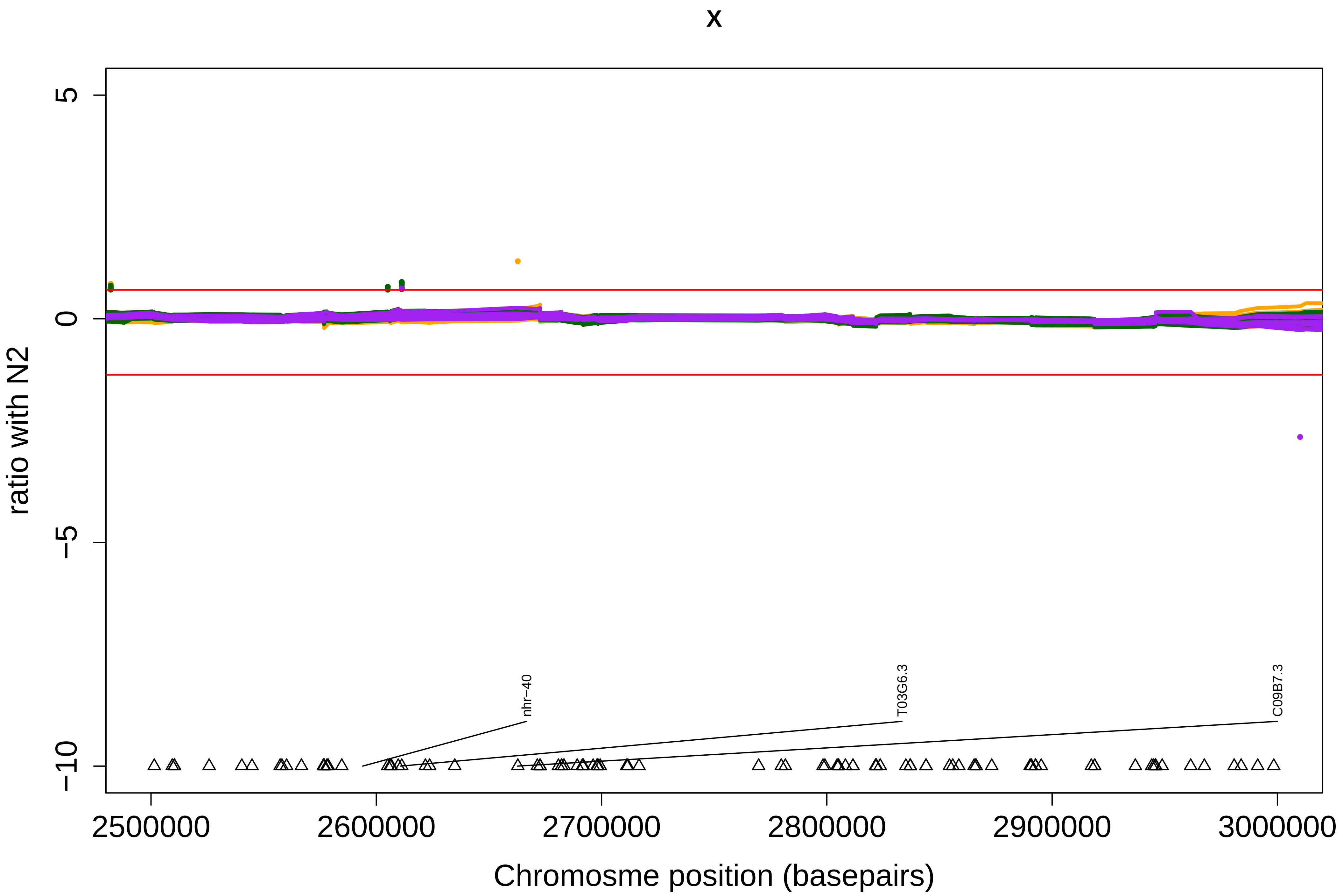
X
ratio with N2
5
0
−5
−10
nhr−40
T03G6.3
C09B7.3
2500000
2600000
2700000
2800000
2900000
3000000
Chromosme position (basepairs)

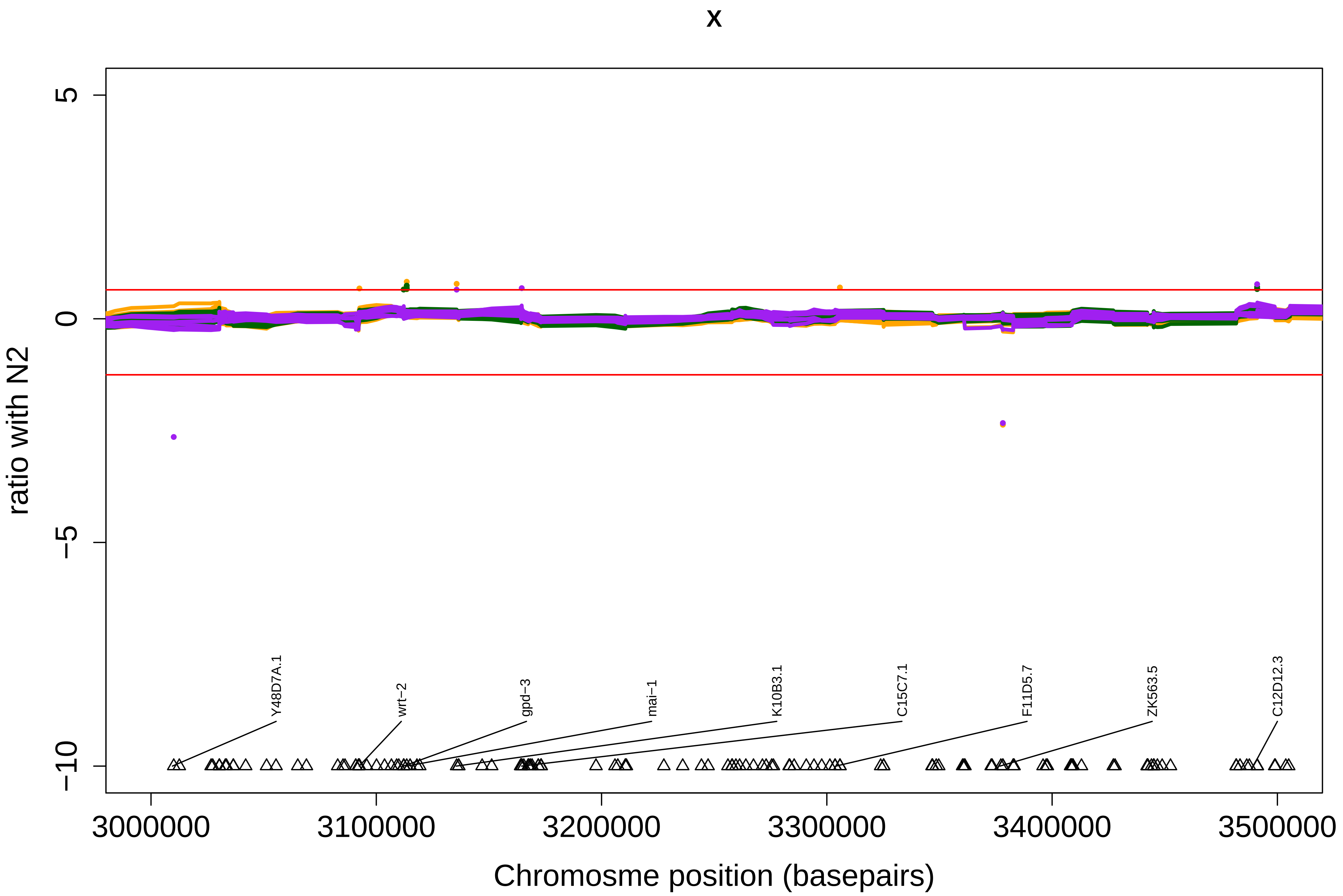

X
ratio with N2
5
0
−5
−10
Y48D7A.1
wrt−2
gpd−3
mai−1
K10B3.1
C15C7.1
F11D5.7
ZK563.5
C12D12.3
3000000
3100000
3200000
3300000
3400000
3500000
Chromosme position (basepairs)

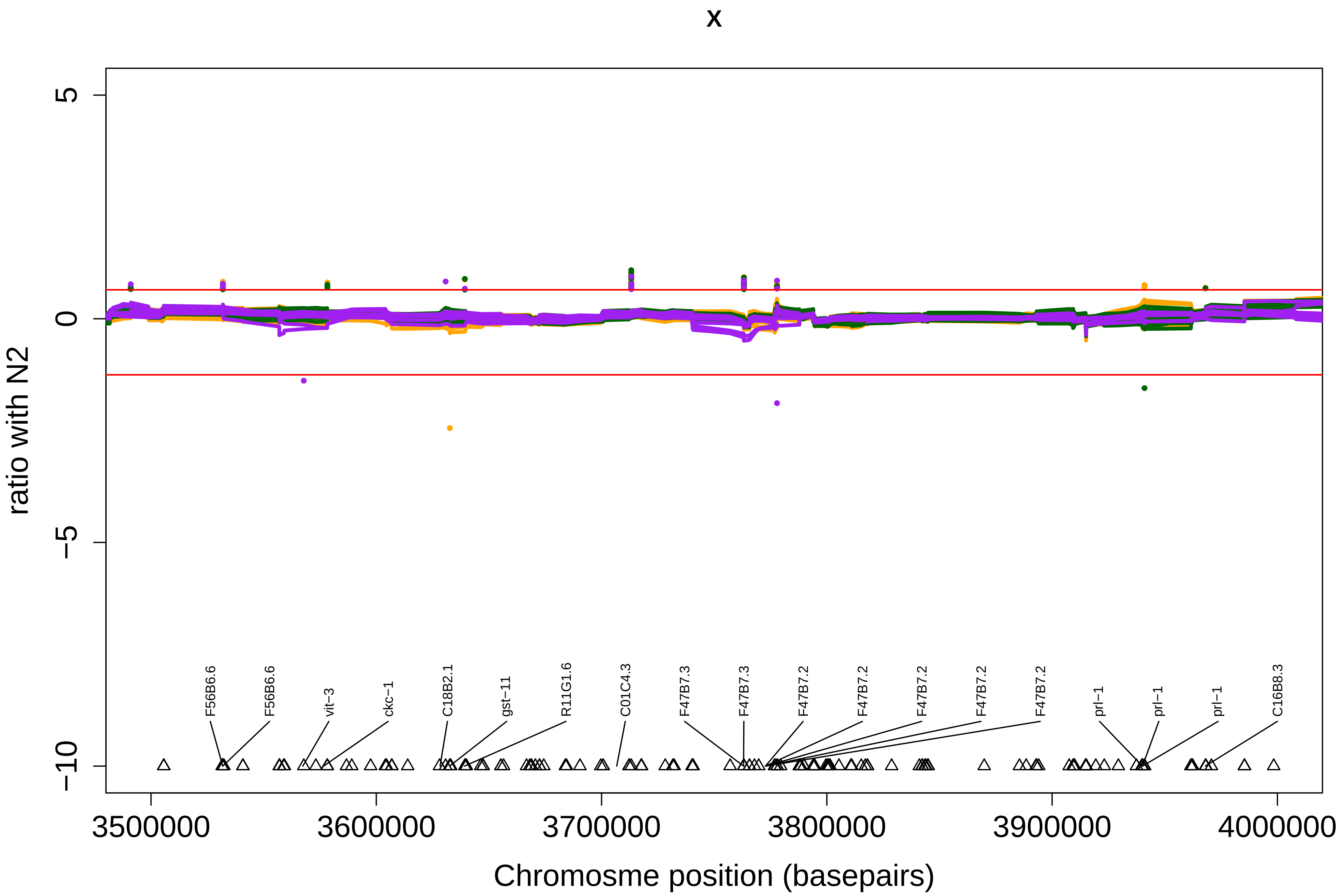

X
ratio with N2
5
0
−5
−10
F56B6.6
F56B6.6
vit−3
ckc−1
C18B2.1
gst−11
R11G1.6
C01C4.3
F47B7.3
F47B7.3
F47B7.2
F47B7.2
F47B7.2
F47B7.2
F47B7.2
prl−1
prl−1
prl−1
C16B8.3
3500000
3600000
3700000
3800000
3900000
4000000
Chromosme position (basepairs)

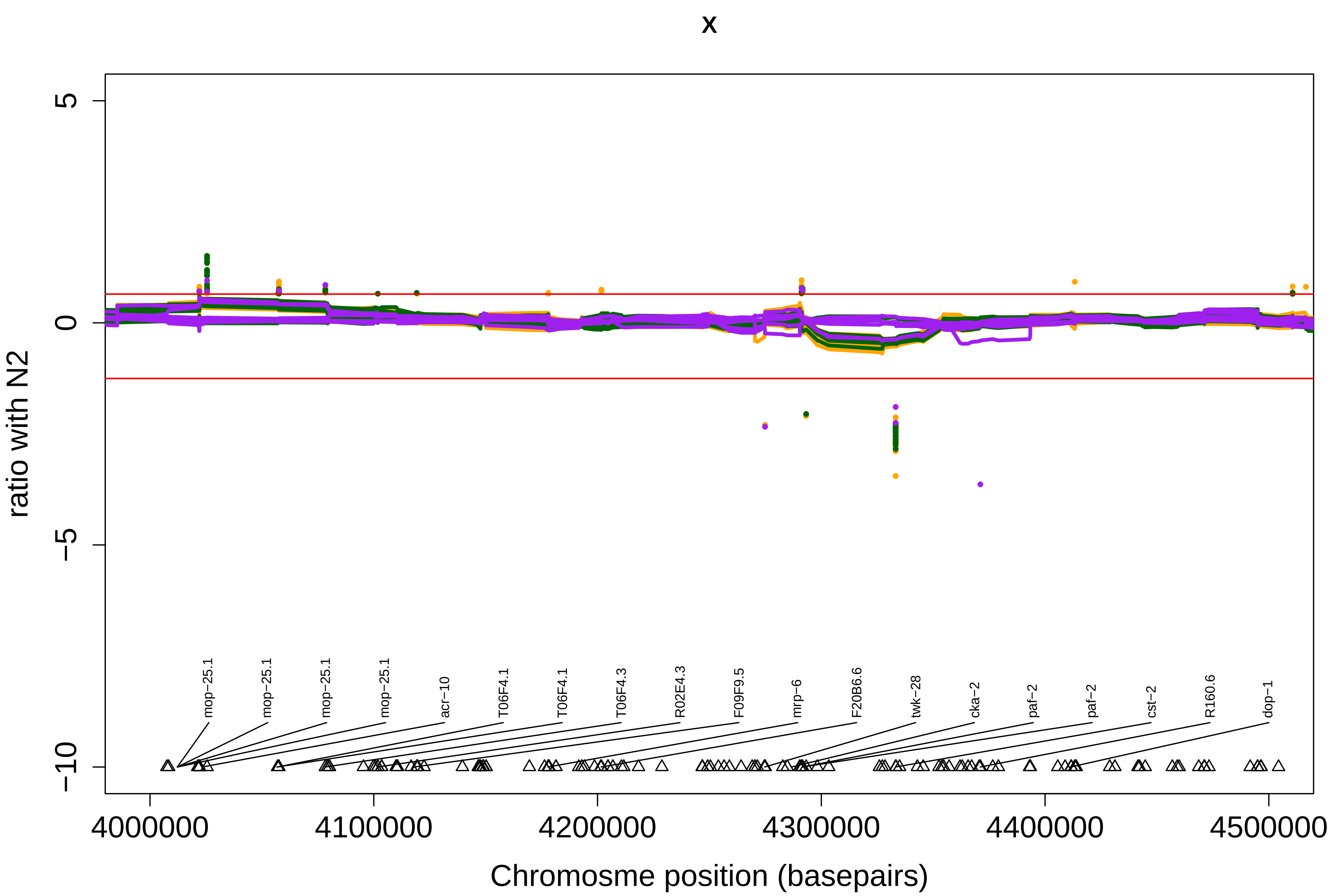
X
ratio with N2
5
0
−5
−10
mop−25.1
mop−25.1
mop−25.1
mop−25.1
acr−10
T06F4.1
T06F4.1
T06F4.3
R02E4.3
F09F9.5
mrp−6
F20B6.6
twk−28
cka−2
paf−2
paf−2
cst−2
R160.6
dop−1
4000000
4100000
4200000
4300000
4400000
4500000
Chromosme position (basepairs)

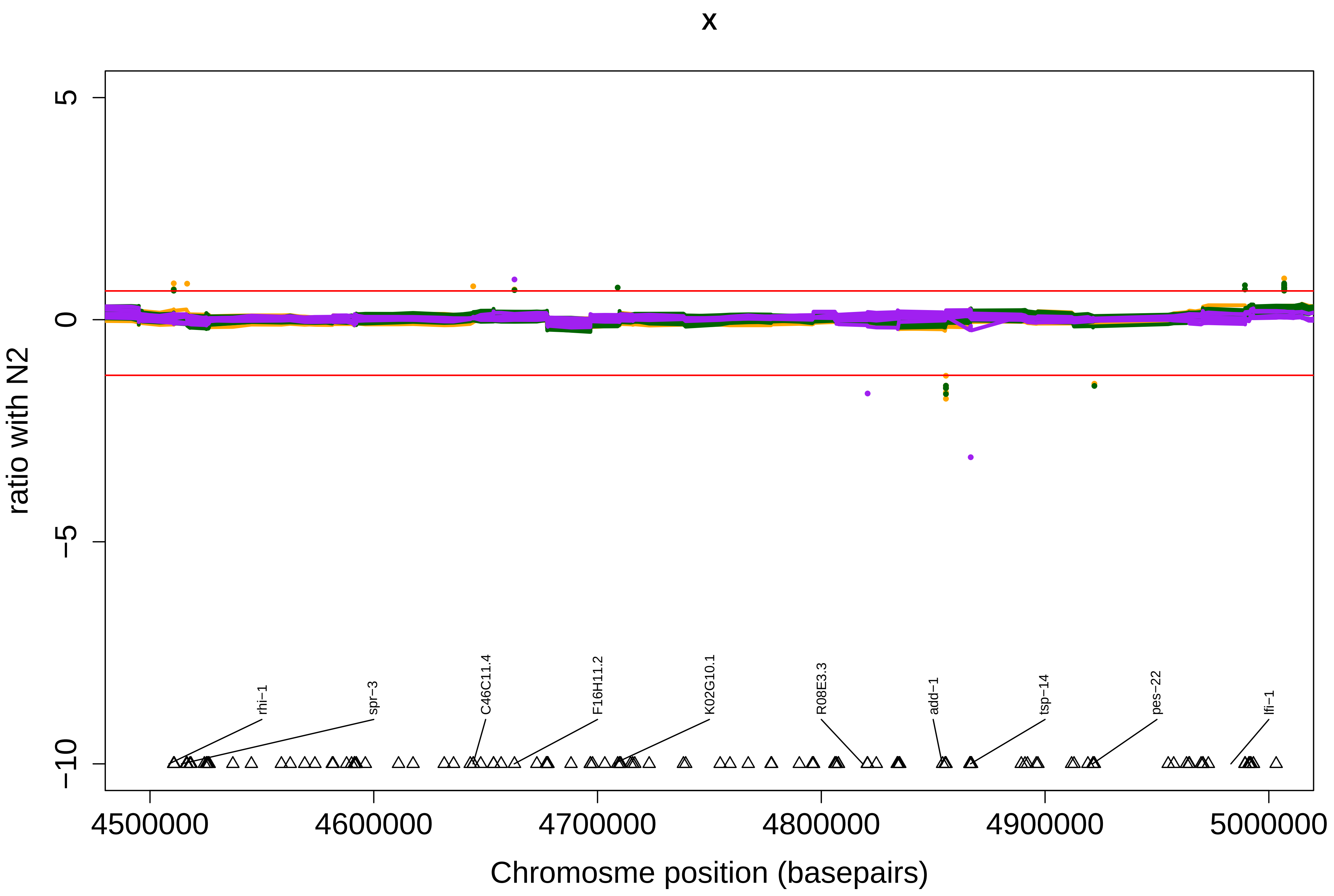

X
ratio with N2
5
0
−5
−10
rhi−1
spr−3
C46C11.4
F16H11.2
K02G10.1
R08E3.3
add−1
tsp−14
pes−22
lfi−1
4500000
4600000
4700000
4800000
4900000
5000000
Chromosme position (basepairs)

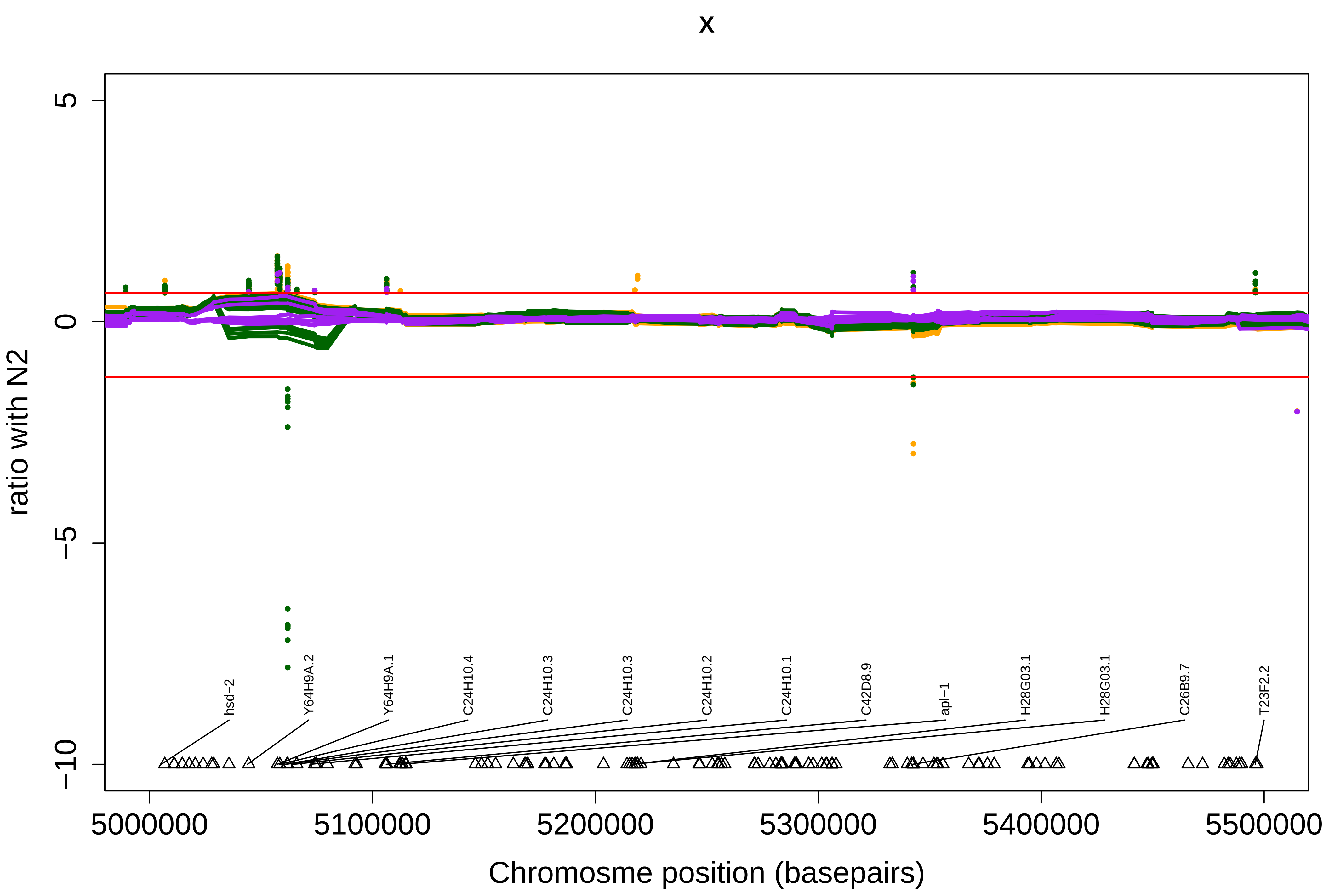

X
ratio with N2
Chromosme position (basepairs)
5
0
−5
−10
5000000
5100000
5200000
5300000
5400000
5500000
hsd−2
Y64H9A.2
Y64H9A.1
C24H10.4
C24H10.3
C24H10.3
C24H10.2
C24H10.1
C42D8.9
apl−1
H28G03.1
H28G03.1
C26B9.7
T23F2.2

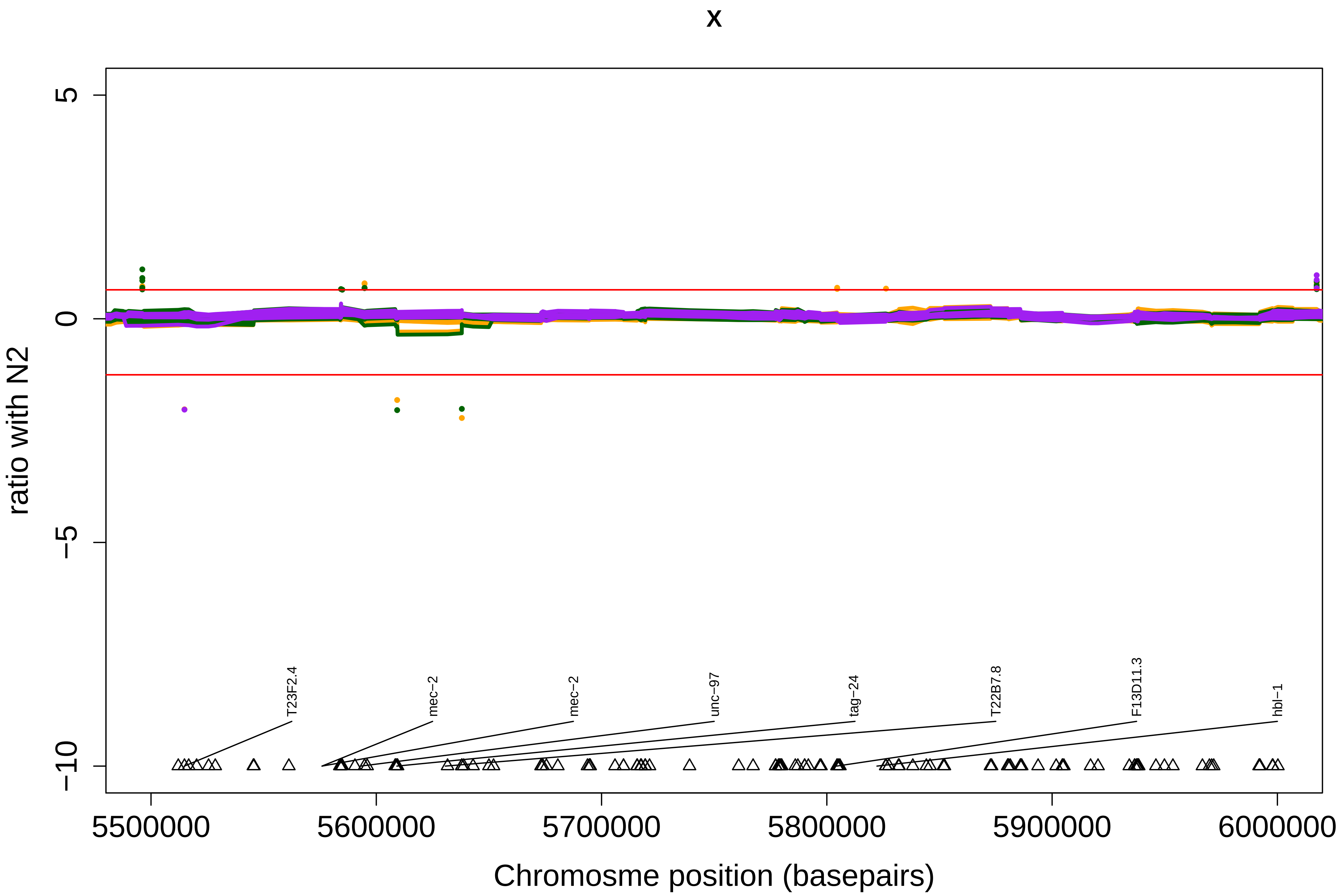

X
ratio with N2
5
0
−5
−10
T23F2.4
mec−2
mec−2
unc−97
tag−24
T22B7.8
F13D11.3
hbl−1
5500000
5600000
5700000
5800000
5900000
6000000
Chromosme position (basepairs)

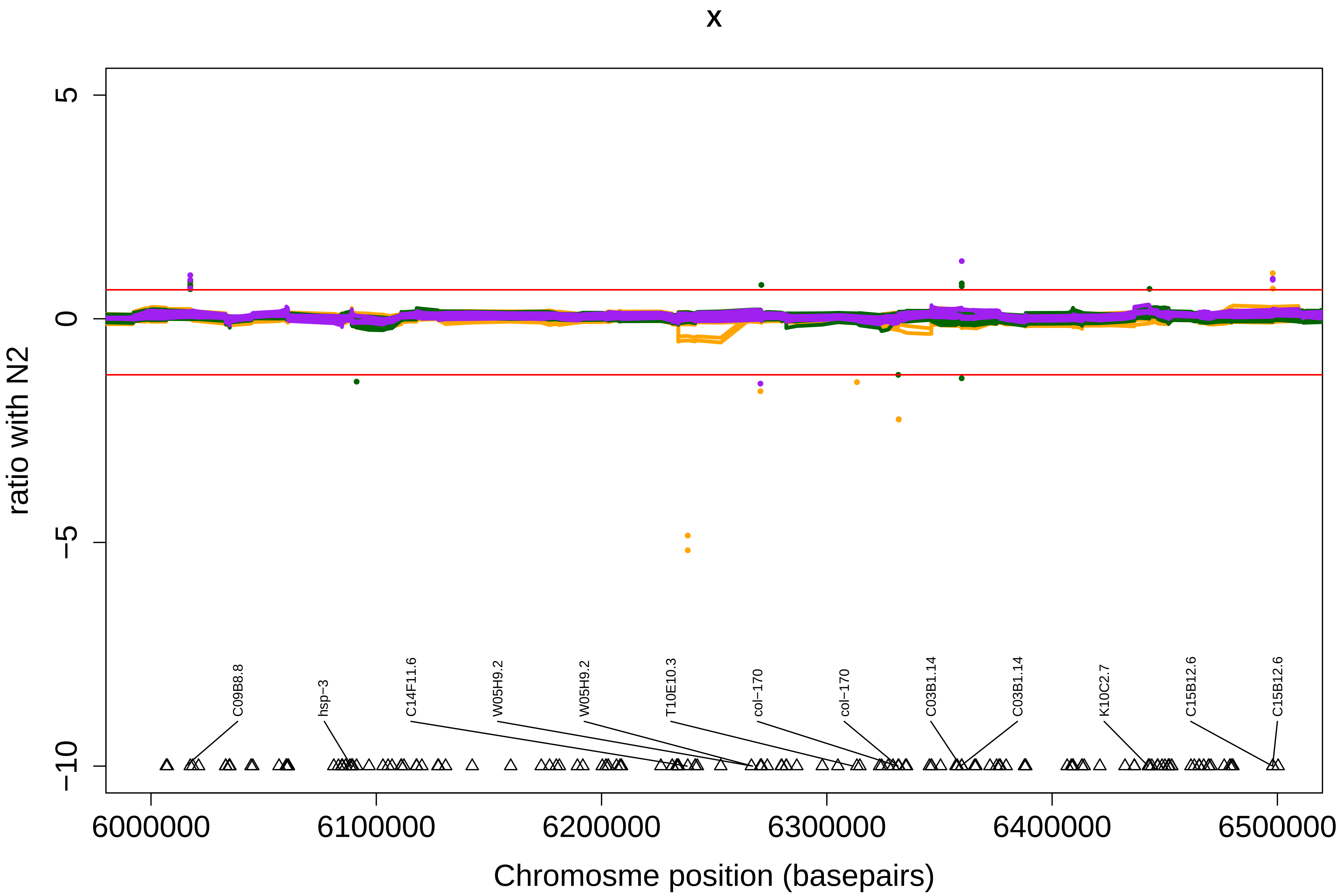

X
ratio with N2
5
0
−5
−10
C09B8.8
hsp−3
C14F11.6
W05H9.2
W05H9.2
T10E10.3
col−170
col−170
C03B1.14
C03B1.14
K10C2.7
C15B12.6
C15B12.6
6000000
6100000
6200000
6300000
6400000
6500000
Chromosme position (basepairs)

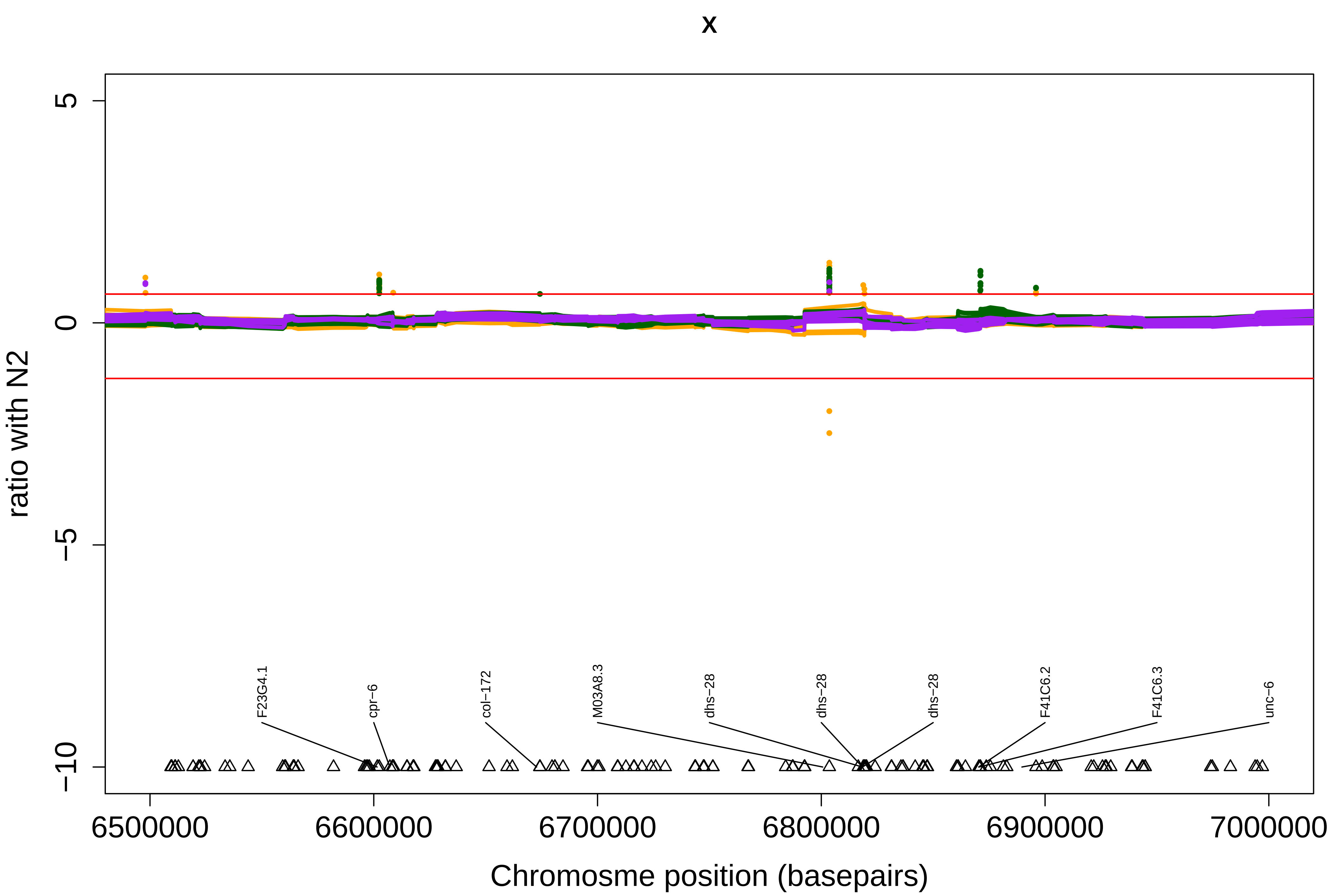
X
ratio with N2
5
0
−5
−10
F23G4.1
cpr−6
col−172
M03A8.3
dhs−28
dhs−28
dhs−28
F41C6.2
F41C6.3
unc−6
6500000
6600000
6700000
6800000
6900000
7000000
Chromosme position (basepairs)

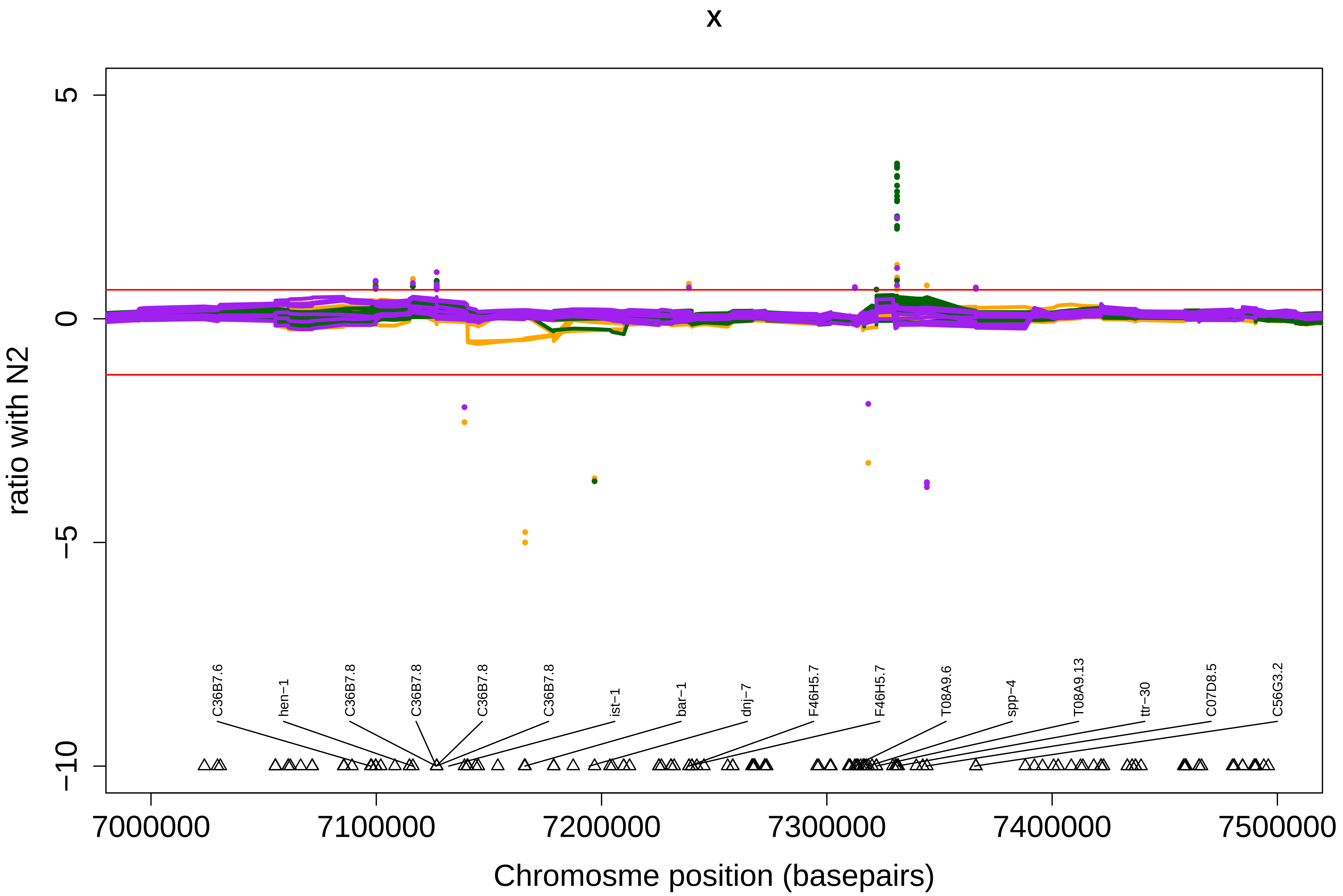
X
ratio with N2
5
0
−5
−10
C36B7.6
hen−1
C36B7.8
C36B7.8
C36B7.8
C36B7.8
ist−1
bar−1
dnj−7
F46H5.7
F46H5.7
T08A9.6
spp−4
T08A9.13
ttr−30
C07D8.5
C56G3.2
7000000
7100000
7200000
7300000
7400000
7500000
Chromosme position (basepairs)

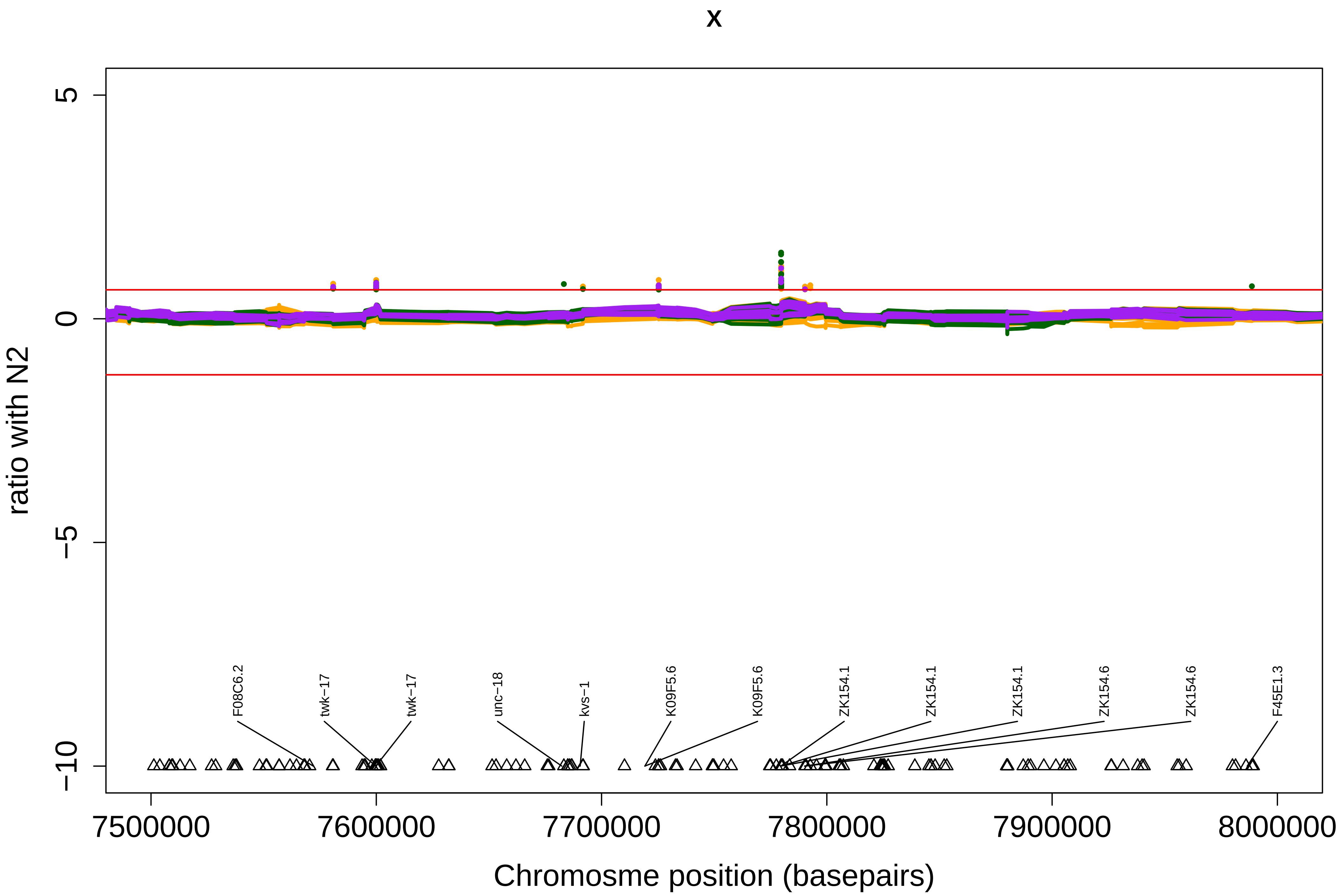

X
ratio with N2
5
0
−5
−10
F08C6.2
twk−17
twk−17
unc−18
kvs−1
K09F5.6
K09F5.6
ZK154.1
ZK154.1
ZK154.1
ZK154.6
ZK154.6
F45E1.3
7500000
7600000
7700000
7800000
7900000
8000000
Chromosme position (basepairs)

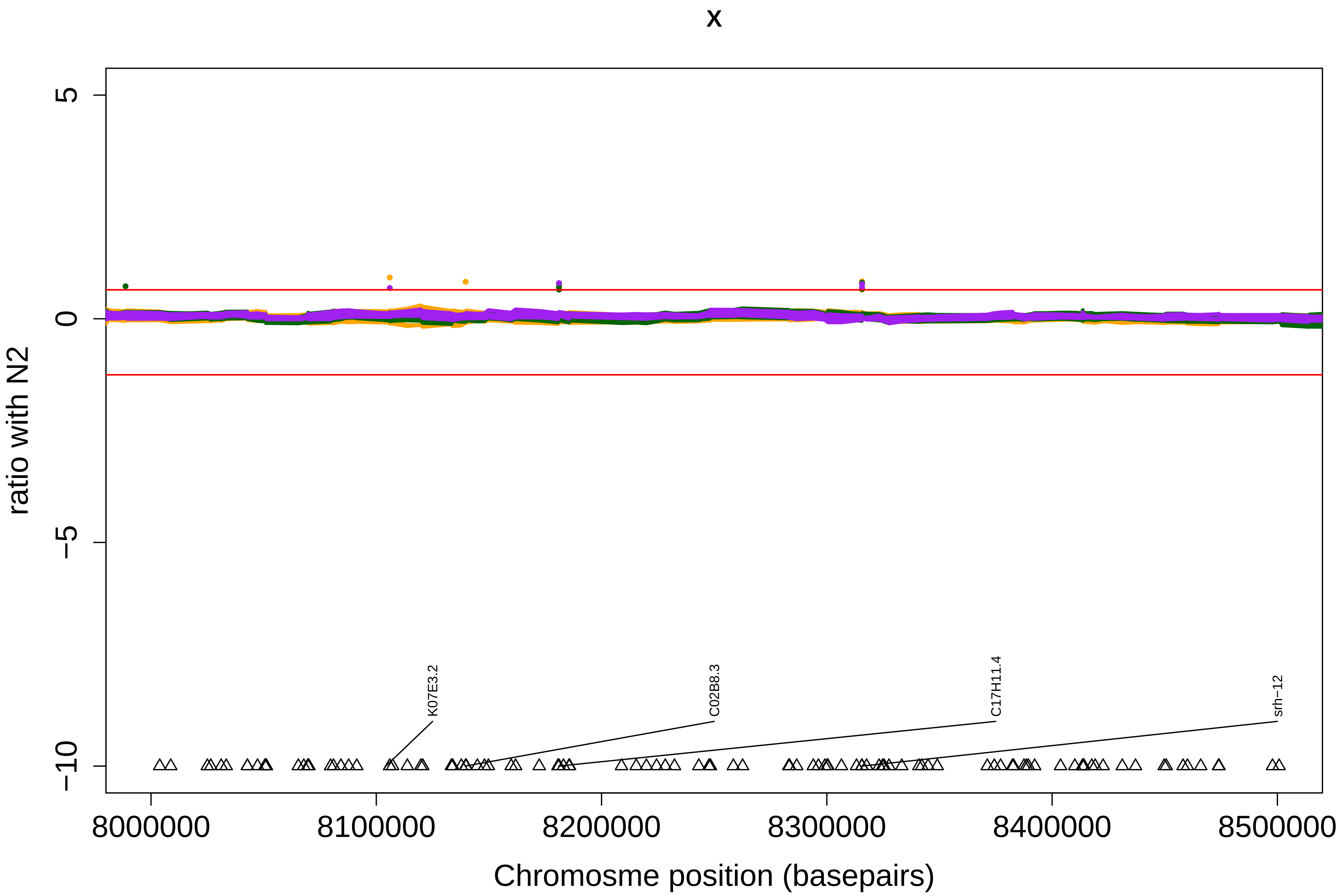
X
5
0
−5
−10
ratio with N2
K07E3.2
C02B8.3
C17H11.4
srh−12
8000000
8100000
8200000
8300000
8400000
8500000
Chromosme position (basepairs)

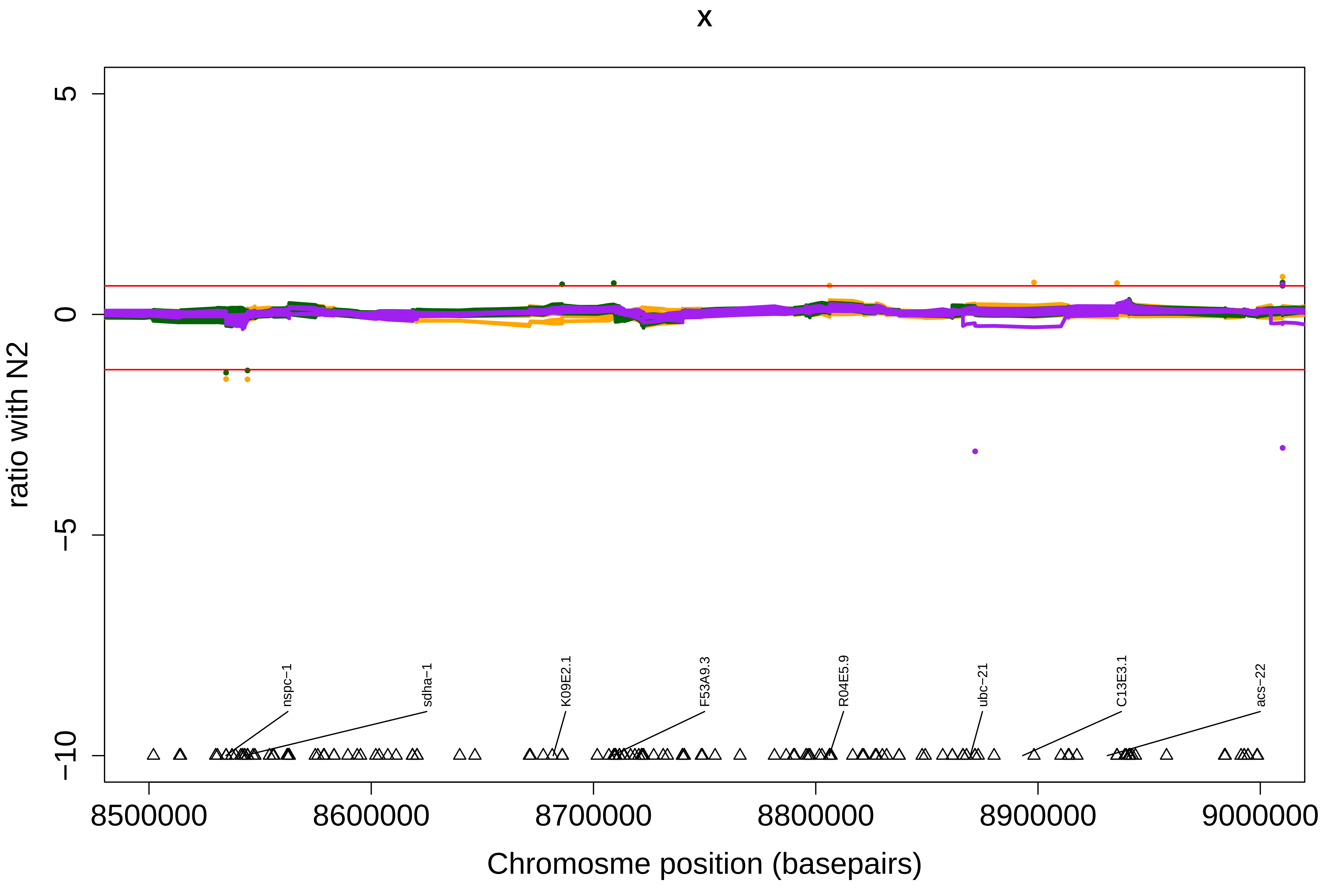

X
ratio with N2
5
0
−5
−10
nspc−1
sdha−1
K09E2.1
F53A9.3
R04E5.9
ubc−21
C13E3.1
acs−22
8500000
8600000
8700000
8800000
8900000
9000000
Chromosme position (basepairs)

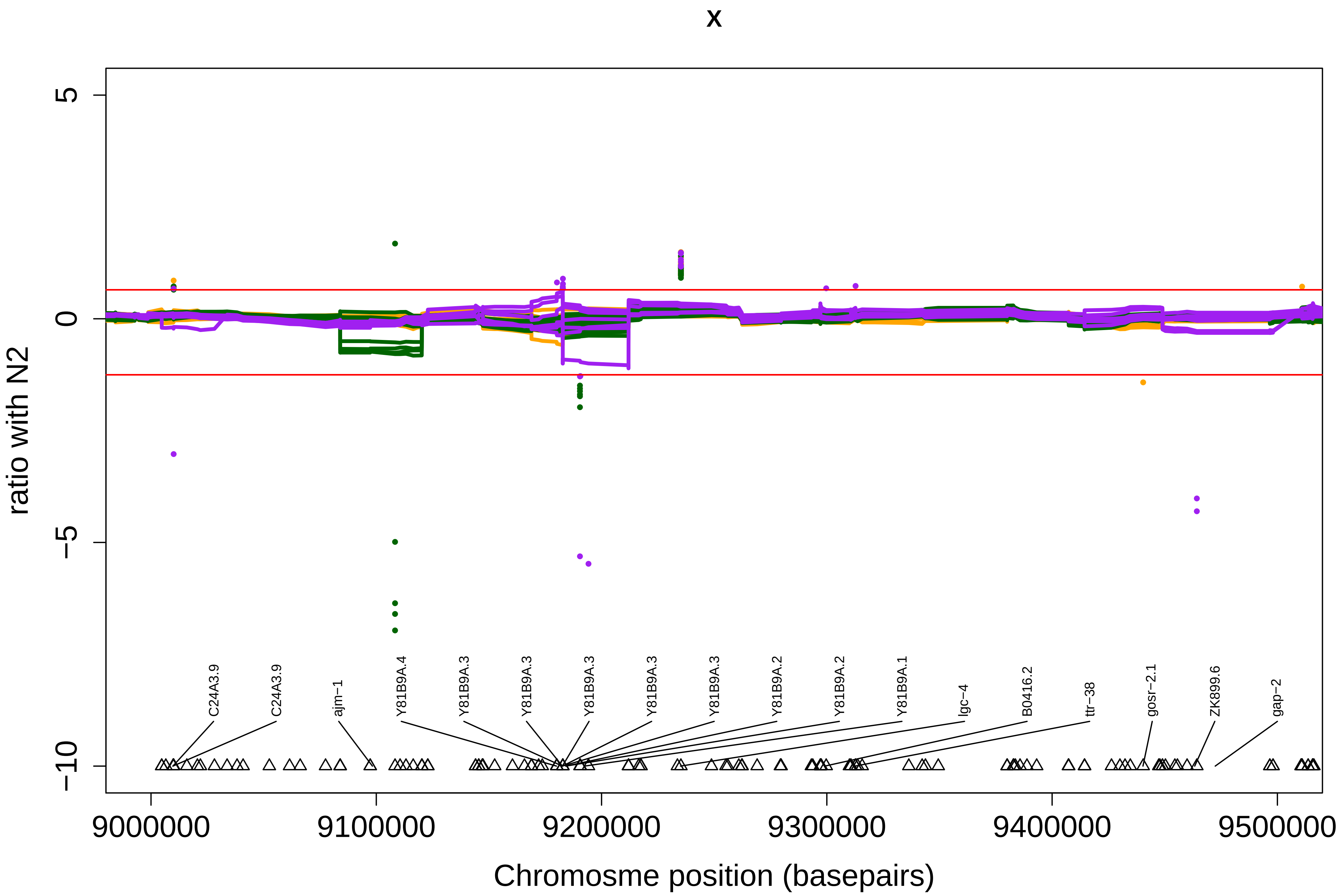

X
ratio with N2
5
0
−5
−10
C24A3.9
C24A3.9
ajm−1
Y81B9A.4
Y81B9A.3
Y81B9A.3
Y81B9A.3
Y81B9A.3
Y81B9A.3
Y81B9A.2
Y81B9A.2
Y81B9A.1
lgc−4
B0416.2
ttr−38
gosr−2.1
ZK899.6
gap−2
9000000
9100000
9200000
9300000
9400000
9500000
Chromosme position (basepairs)

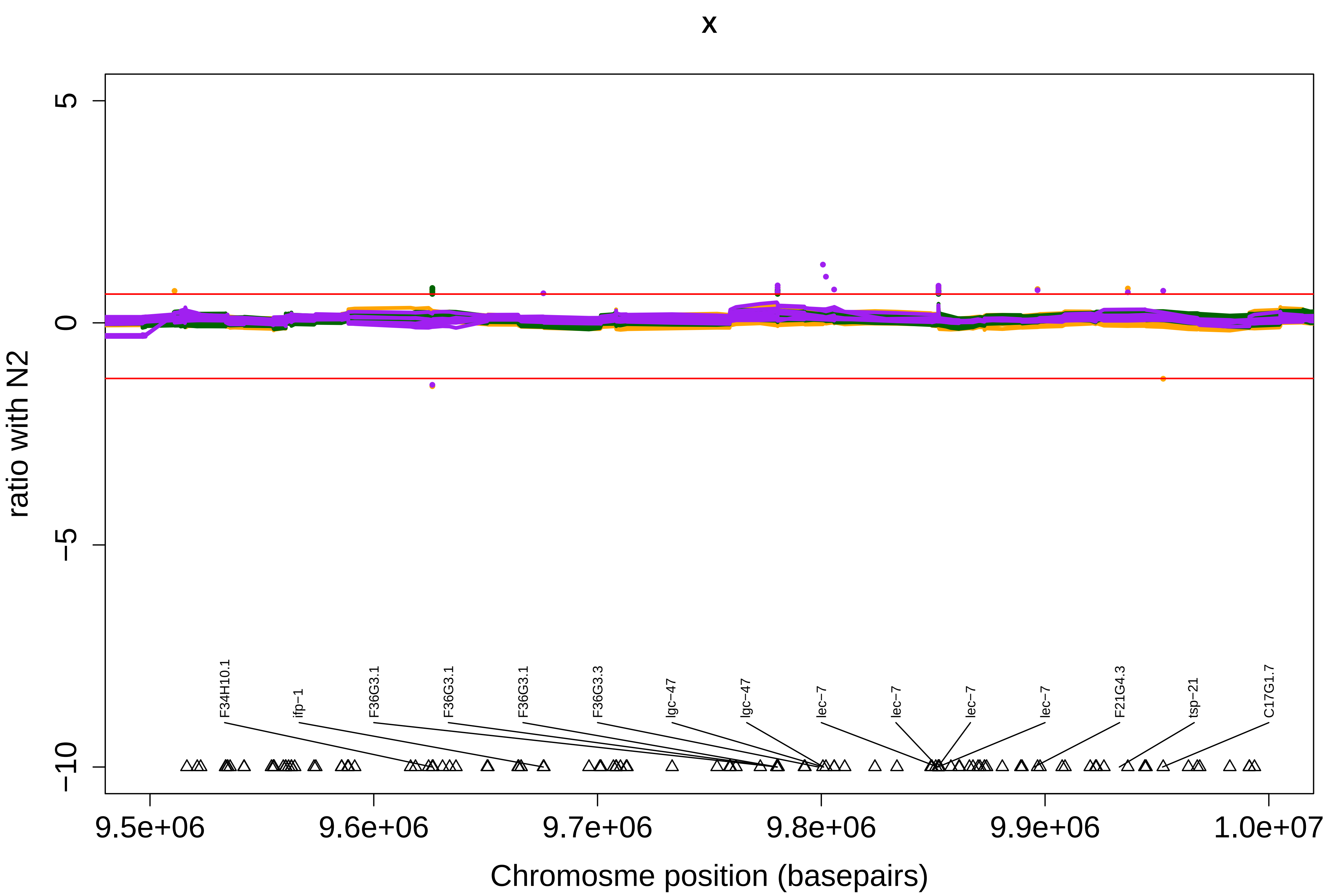

X
ratio with N2
5
0
−5
−10
F34H10.1
ifp−1
F36G3.1
F36G3.1
F36G3.1
F36G3.3
lgc−47
lgc−47
lec−7
lec−7
lec−7
lec−7
F21G4.3
tsp−21
C17G1.7
9.5e+06
9.6e+06
9.7e+06
9.8e+06
9.9e+06
1.0e+07
Chromosme position (basepairs)

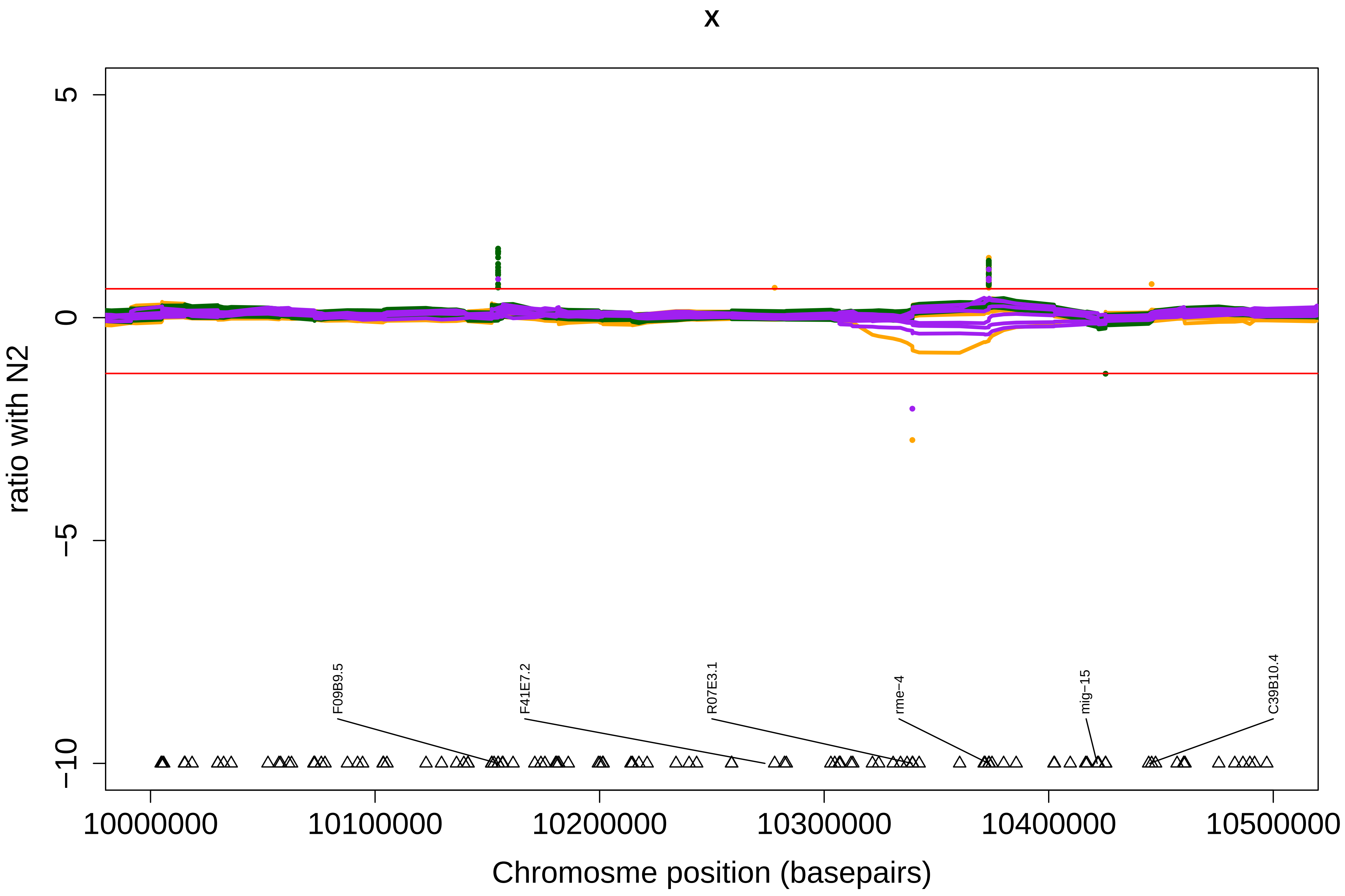
X
ratio with N2
5
0
−5
−10
F09B9.5
F41E7.2
R07E3.1
rme−4
mig−15
C39B10.4
10000000
10100000
10200000
10300000
10400000
10500000
Chromosme position (basepairs)

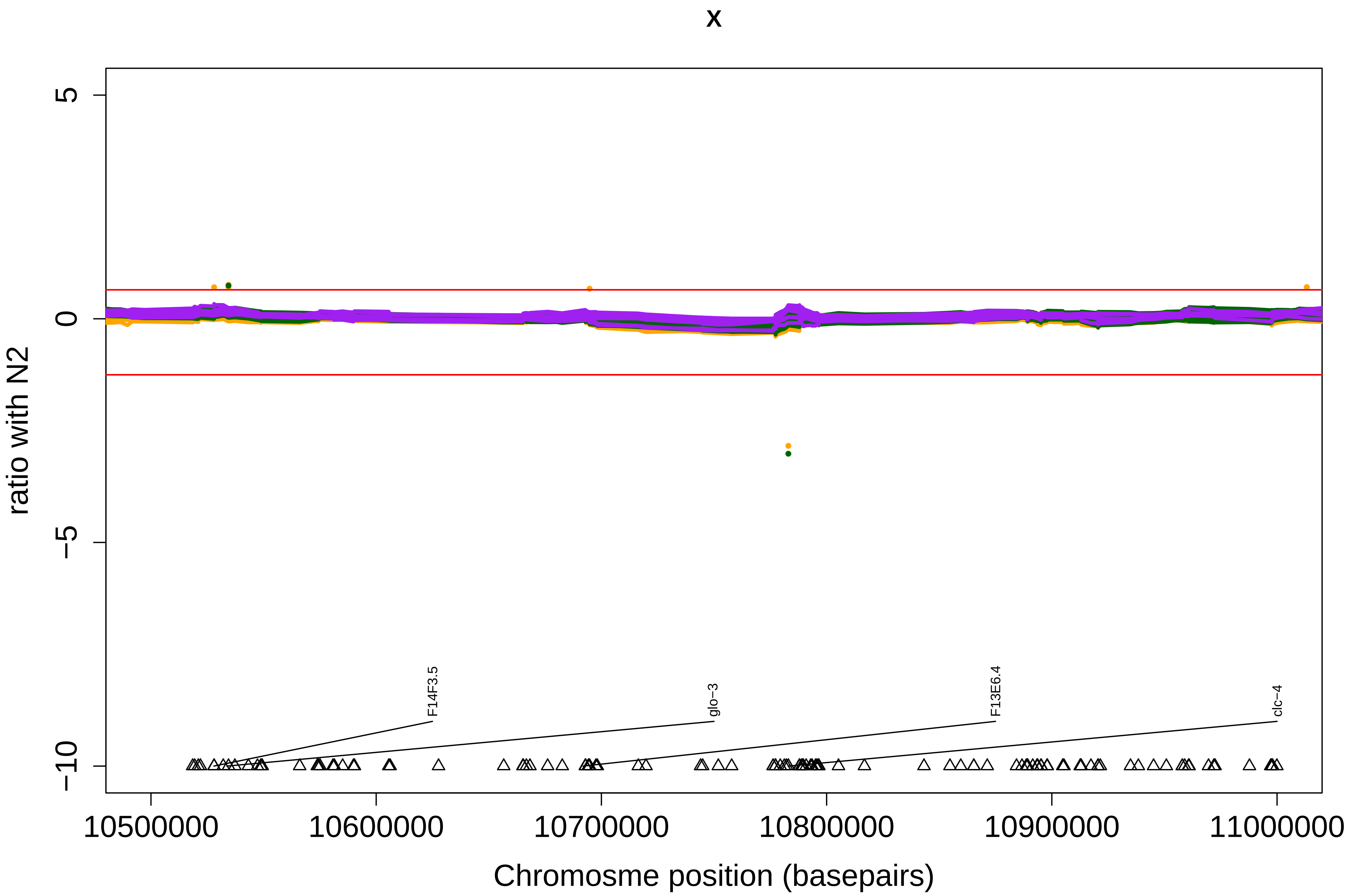

X
ratio with N2
5
0
−5
−10
F14F3.5
glo−3
F13E6.4
clc−4
10500000
10600000
10700000
10800000
10900000
11000000
Chromosme position (basepairs)

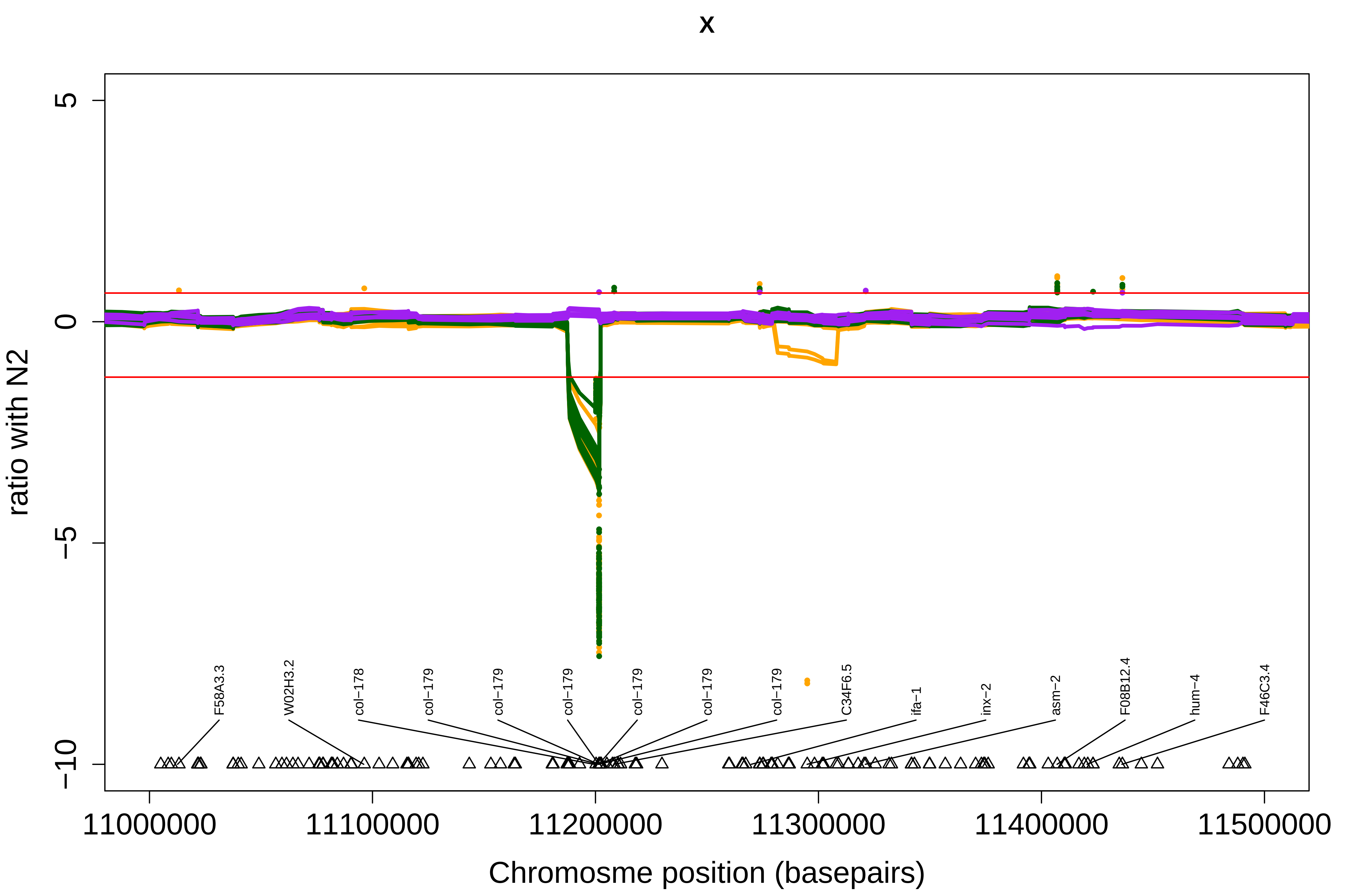
X
ratio with N2
5
0
−5
−10
F58A3.3
W02H3.2
col−178
col−179
col−179
col−179
col−179
col−179
col−179
C34F6.5
ifa−1
inx−2
asm−2
F08B12.4
hum−4
F46C3.4
11000000
11100000
11200000
11300000
11400000
11500000
Chromosme position (basepairs)

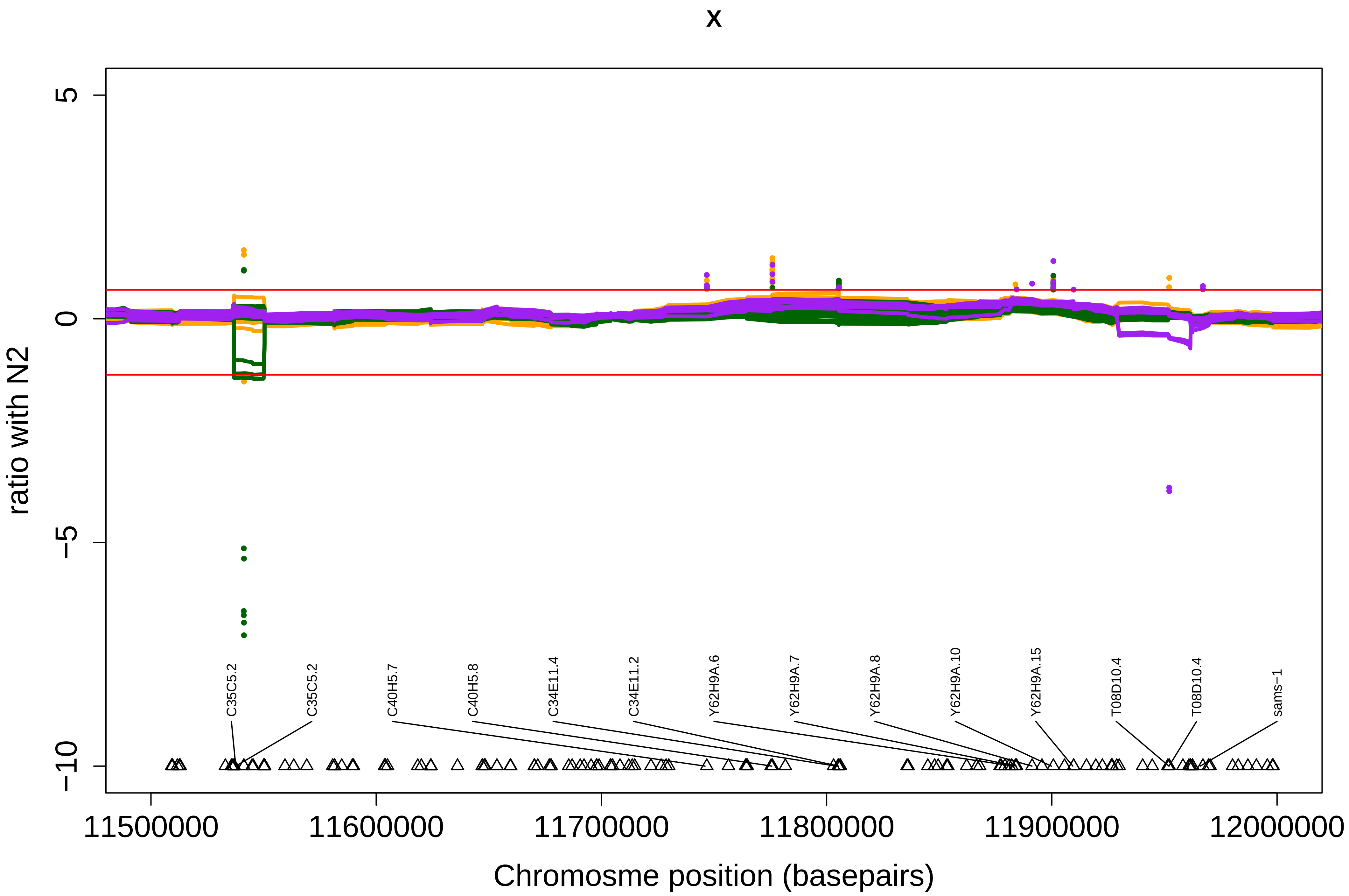

X
ratio with N2
Chromosme position (basepairs)
5
0
−5
−10
11500000
11600000
11700000
11800000
11900000
12000000
C35C5.2
C35C5.2
C40H5.7
C40H5.8
C34E11.4
C34E11.2
Y62H9A.6
Y62H9A.7
Y62H9A.8
Y62H9A.10
Y62H9A.15
T08D10.4
T08D10.4
sams−1

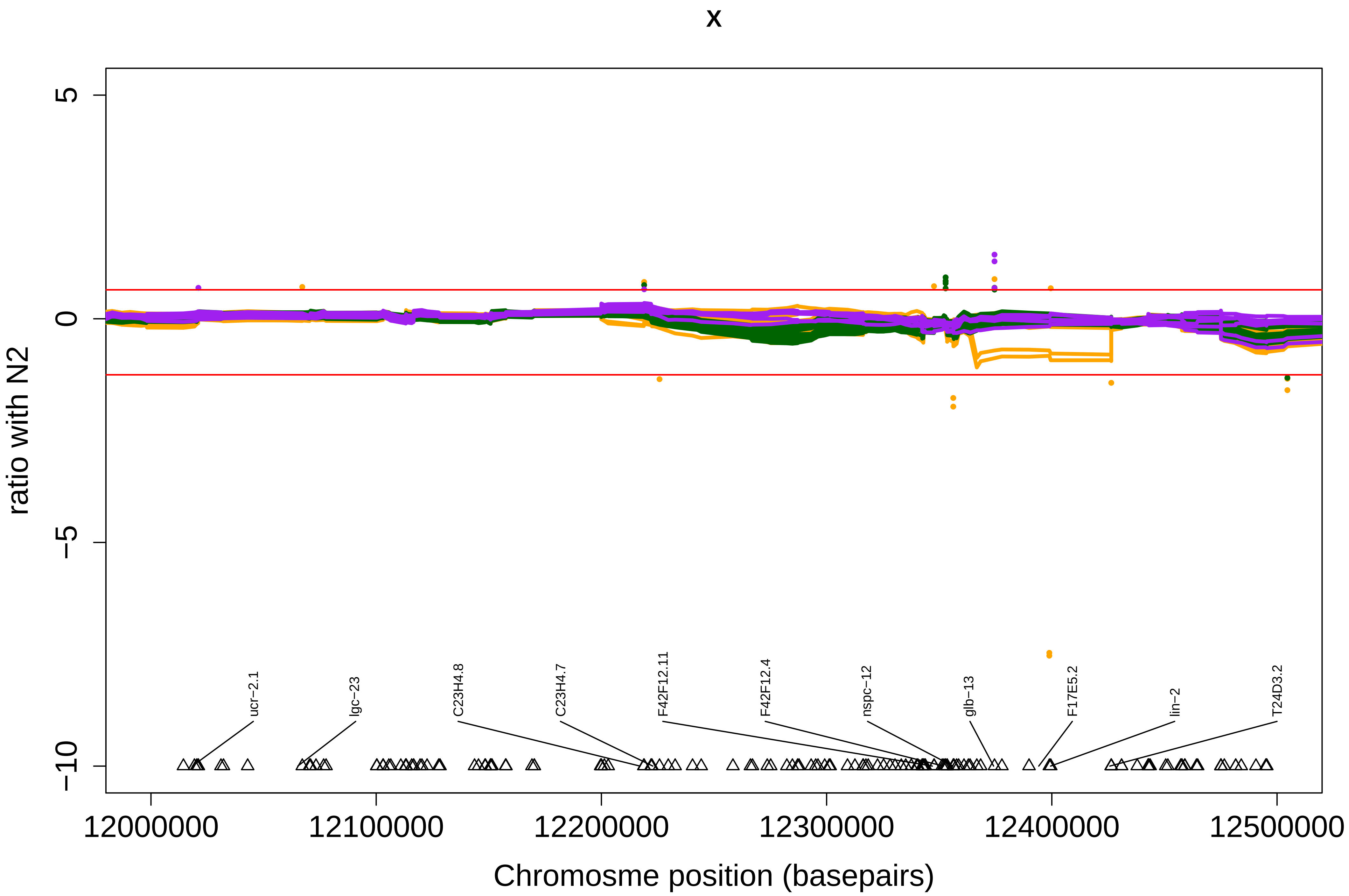

X
ratio with N2
5
0
−5
−10
ucr−2.1
lgc−23
C23H4.8
C23H4.7
F42F12.11
F42F12.4
nspc−12
glb−13
F17E5.2
lin−2
T24D3.2
12000000
12100000
12200000
12300000
12400000
12500000
Chromosme position (basepairs)

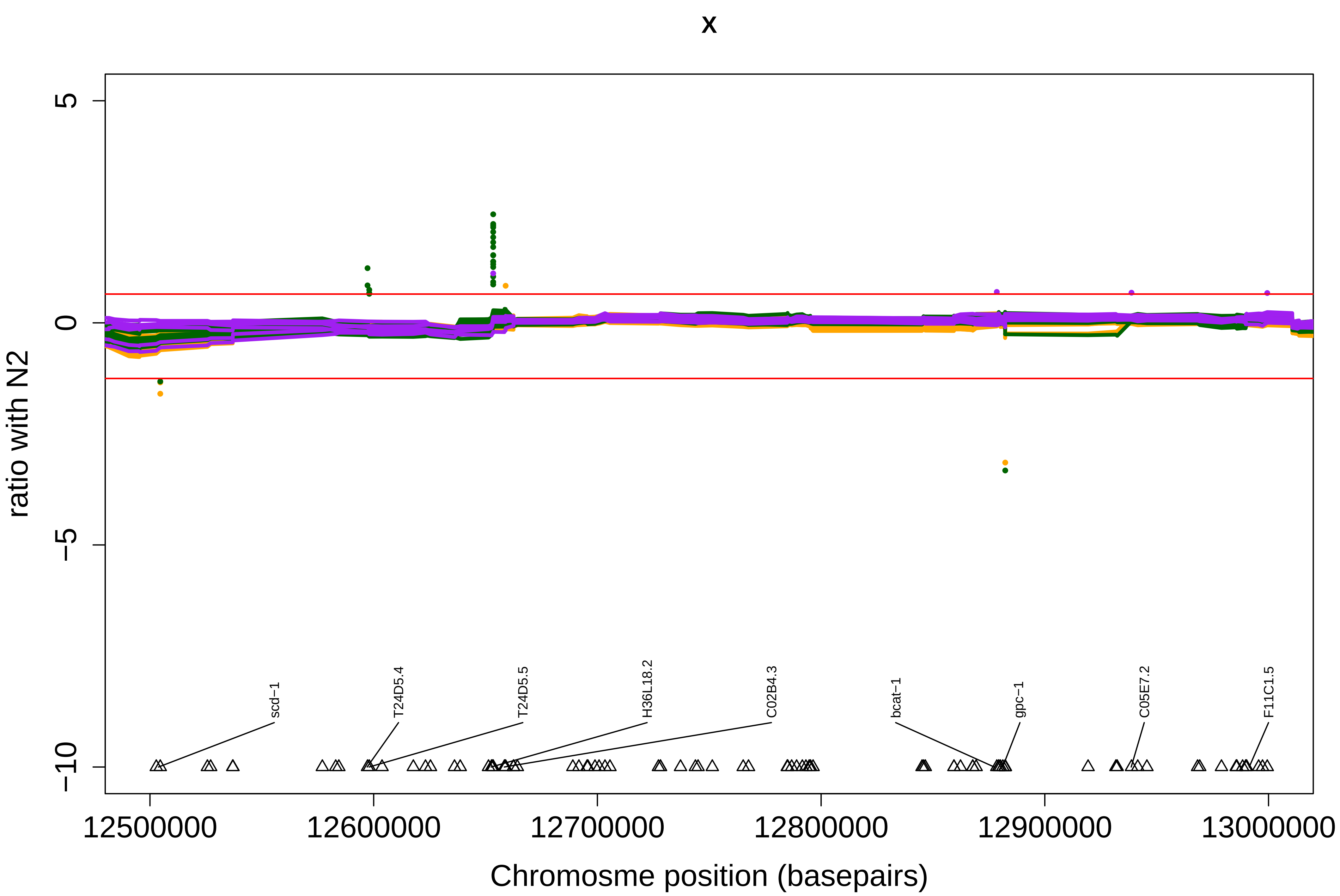
X
ratio with N2
5
0
−5
−10
scd−1
T24D5.4
T24D5.5
H36L18.2
C02B4.3
bcat−1
gpc−1
C05E7.2
F11C1.5
12500000
12600000
12700000
12800000
12900000
13000000
Chromosme position (basepairs)

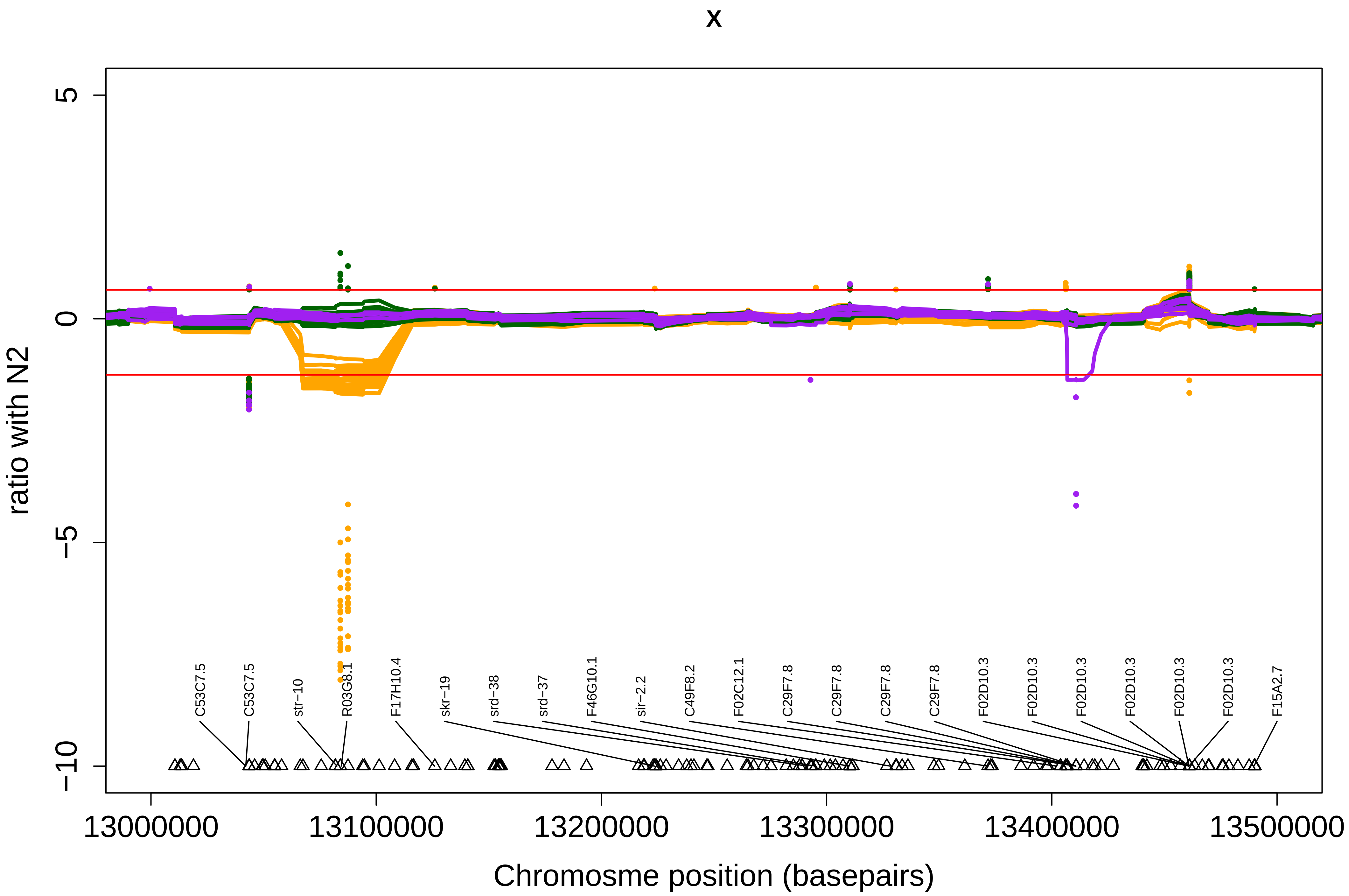

X
ratio with N2
Chromosme position (basepairs)
5
0
−5
−10
13000000
13100000
13200000
13300000
13400000
13500000
C53C7.5
C53C7.5
str−10
R03G8.1
F17H10.4
skr−19
srd−38
srd−37
F46G10.1
sir−2.2
C49F8.2
F02C12.1
C29F7.8
C29F7.8
C29F7.8
C29F7.8
F02D10.3
F02D10.3
F02D10.3
F02D10.3
F02D10.3
F02D10.3
F15A2.7

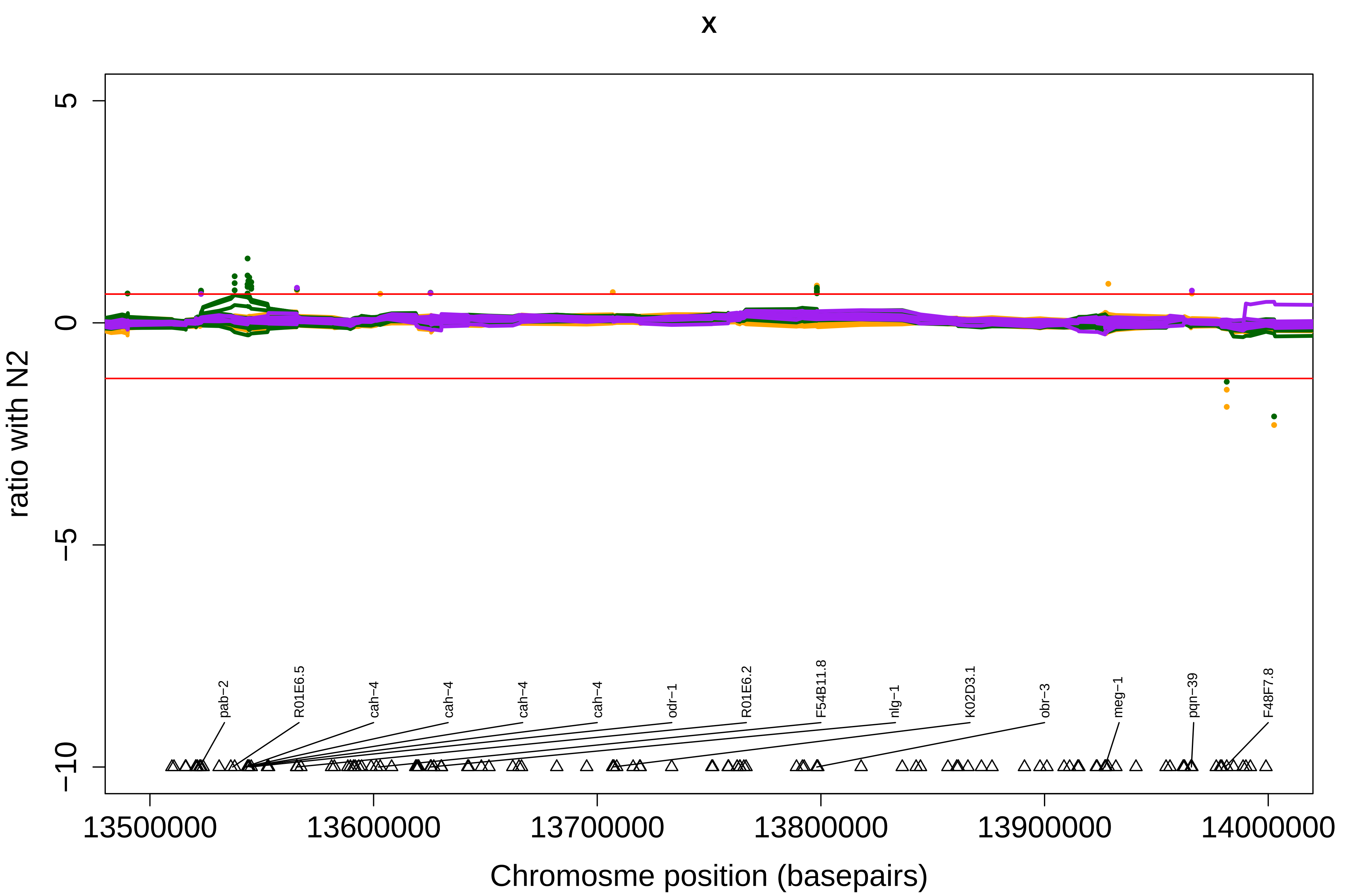

X
ratio with N2
5
0
−5
−10
pab−2
R01E6.5
cah−4
cah−4
cah−4
cah−4
odr−1
R01E6.2
F54B11.8
nlg−1
K02D3.1
obr−3
meg−1
pqn−39
F48F7.8
13500000
13600000
13700000
13800000
13900000
14000000
Chromosme position (basepairs)

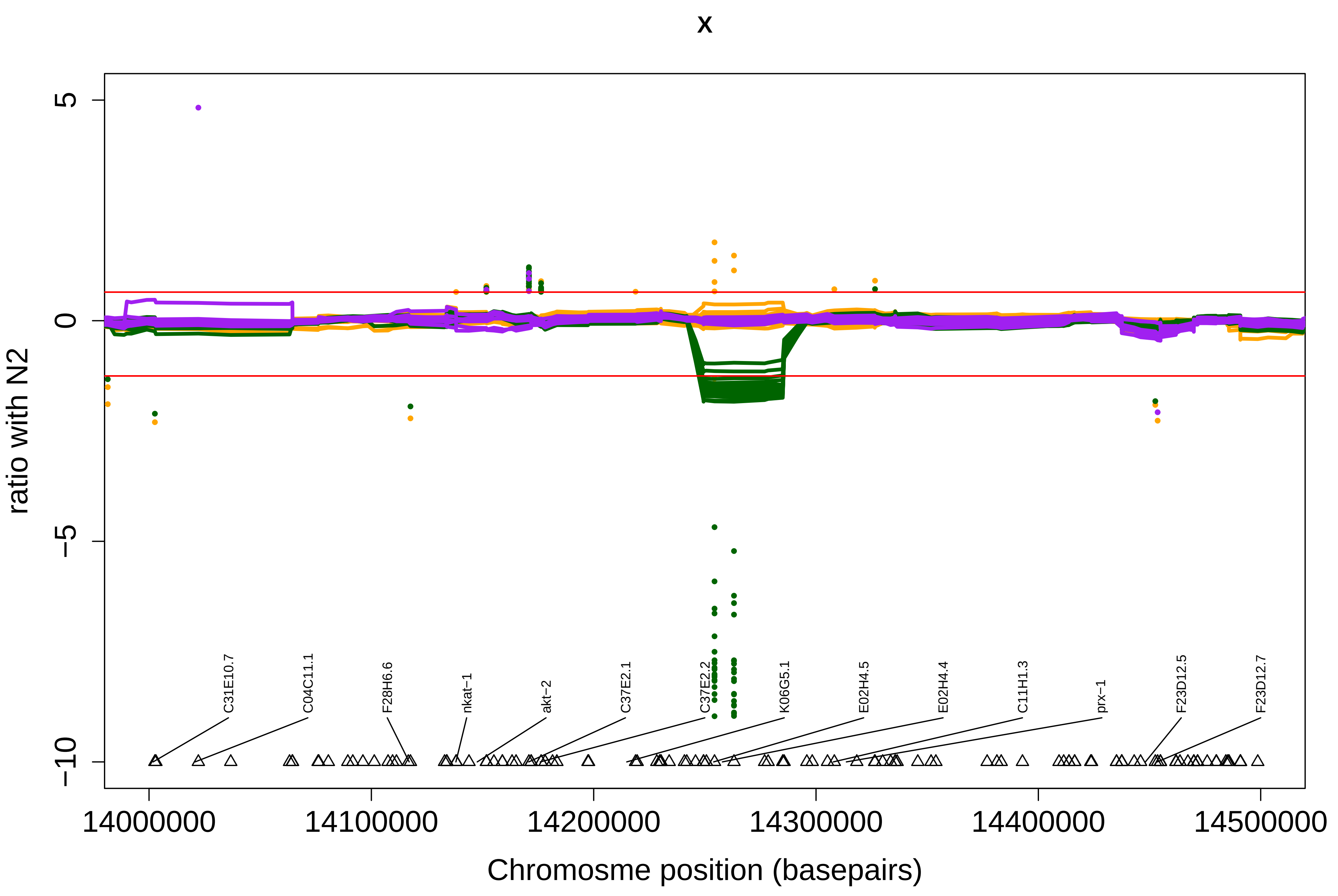

X
ratio with N2
Chromosme position (basepairs)
5
0
−5
−10
14000000
14100000
14200000
14300000
14400000
14500000
C31E10.7
C04C11.1
F28H6.6
nkat−1
akt−2
C37E2.1
C37E2.2
K06G5.1
E02H4.5
E02H4.4
C11H1.3
prx−1
F23D12.5
F23D12.7

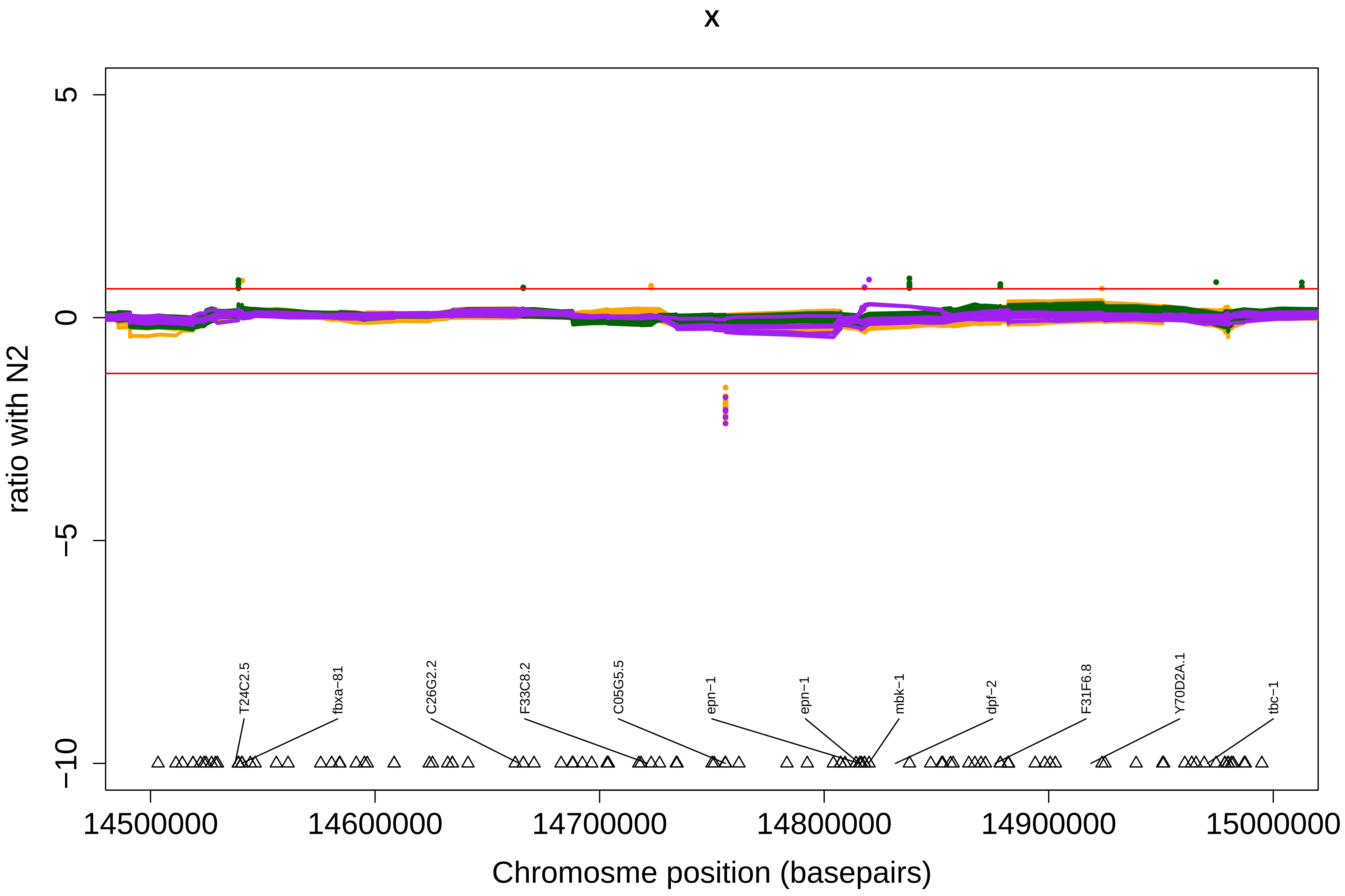

X
ratio with N2
5
0
−5
−10
T24C2.5
fbxa−81
C26G2.2
F33C8.2
C05G5.5
epn−1
epn−1
mbk−1
dpf−2
F31F6.8
Y70D2A.1
tbc−1
14500000
14600000
14700000
14800000
14900000
15000000
Chromosme position (basepairs)

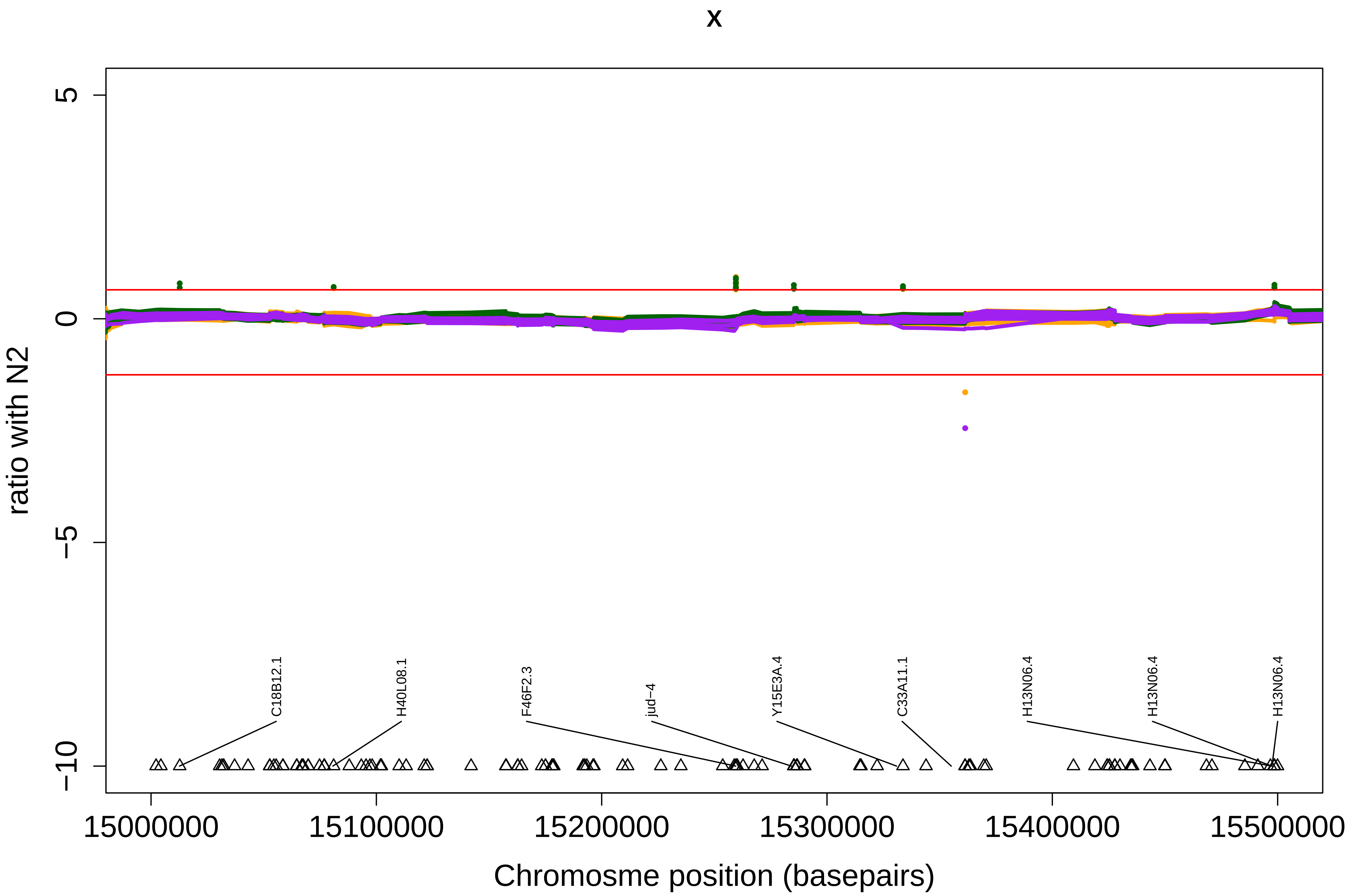
X
ratio with N2
5
0
−5
−10
C18B12.1
H40L08.1
F46F2.3
jud−4
Y15E3A.4
C33A11.1
H13N06.4
H13N06.4
H13N06.4
15000000
15100000
15200000
15300000
15400000
15500000
Chromosme position (basepairs)

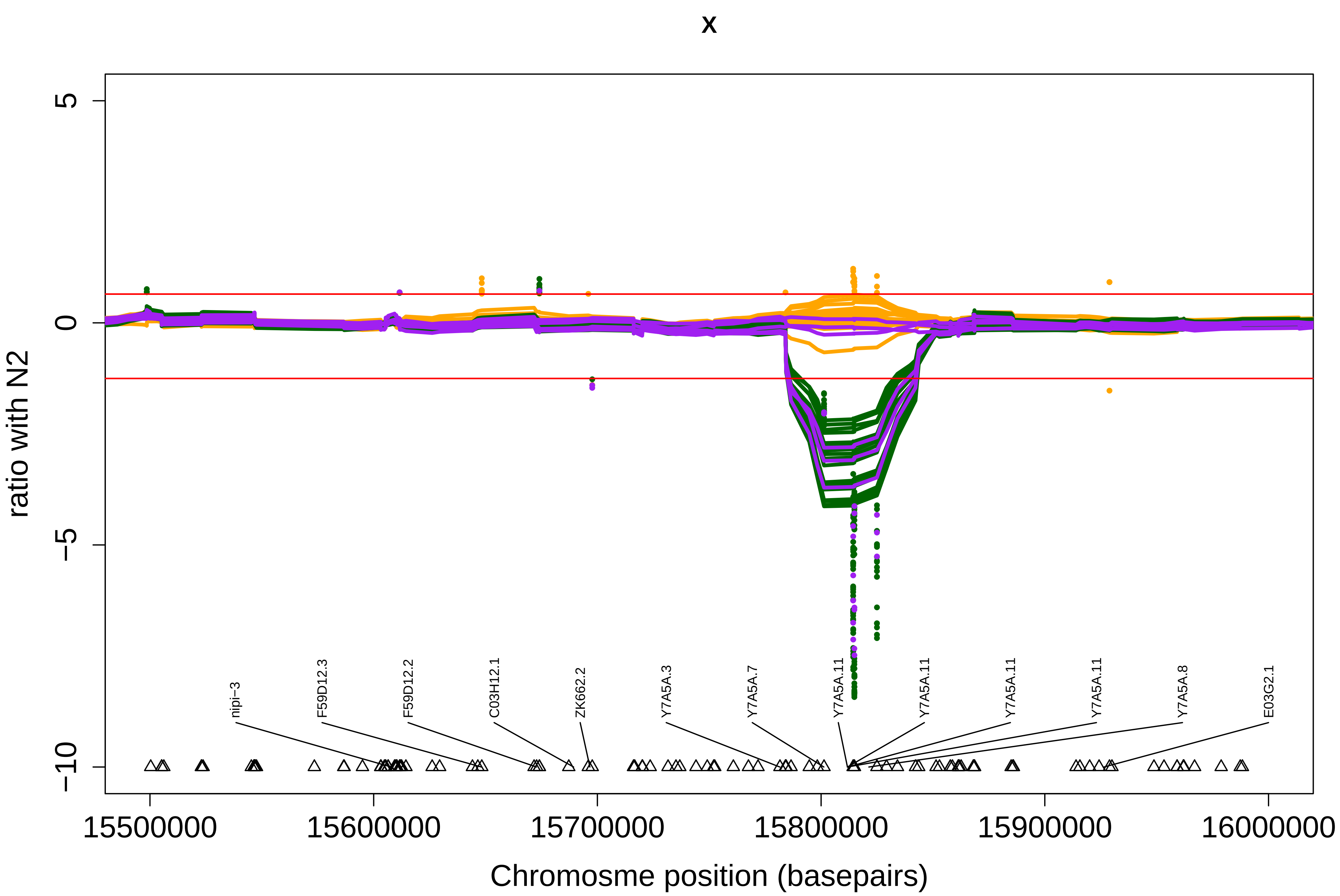

X
ratio with N2
5
0
−5
−10
nipi−3
F59D12.3
F59D12.2
C03H12.1
ZK662.2
Y7A5A.3
Y7A5A.7
Y7A5A.11
Y7A5A.11
Y7A5A.11
Y7A5A.11
Y7A5A.8
E03G2.1
15500000
15600000
15700000
15800000
15900000
16000000
Chromosme position (basepairs)

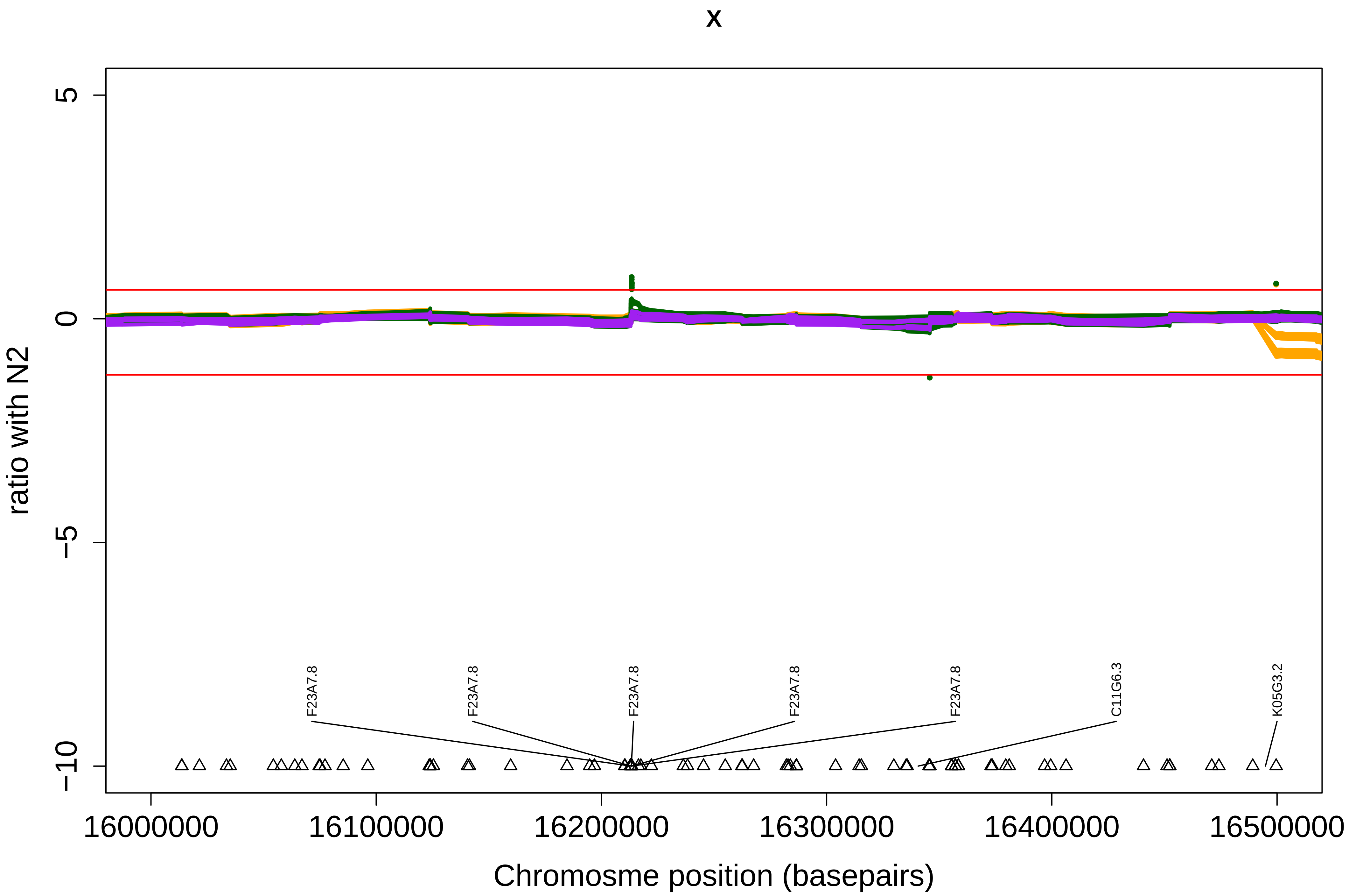
X
ratio with N2
5
0
−5
−10
F23A7.8
F23A7.8
F23A7.8
F23A7.8
F23A7.8
C11G6.3
K05G3.2
16000000
16100000
16200000
16300000
16400000
16500000
Chromosme position (basepairs)

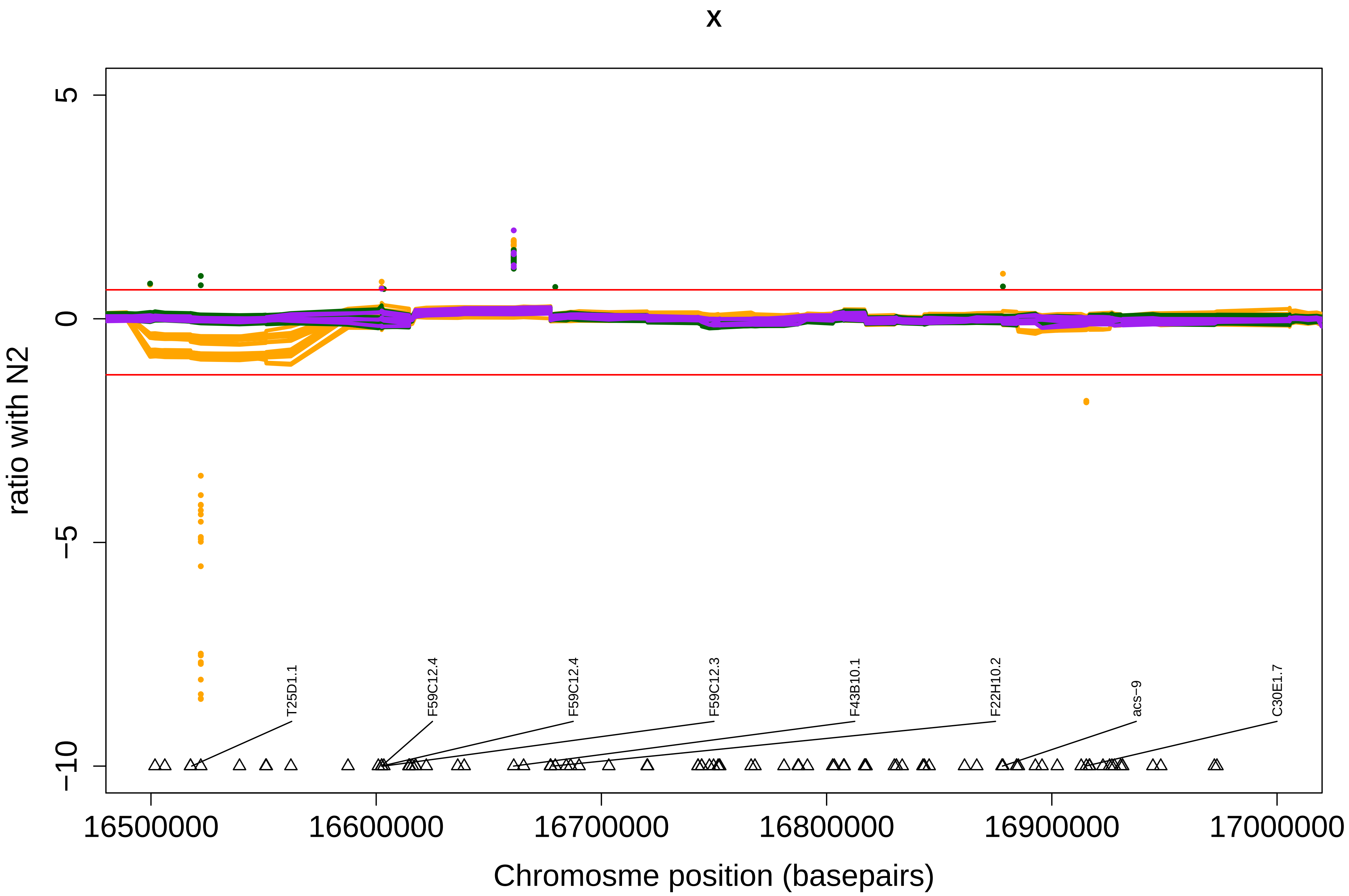
X
ratio with N2
5
0
−5
−10
T25D1.1
F59C12.4
F59C12.4
F59C12.3
F43B10.1
F22H10.2
acs−9
C30E1.7
16500000
16600000
16700000
16800000
16900000
17000000
Chromosme position (basepairs)

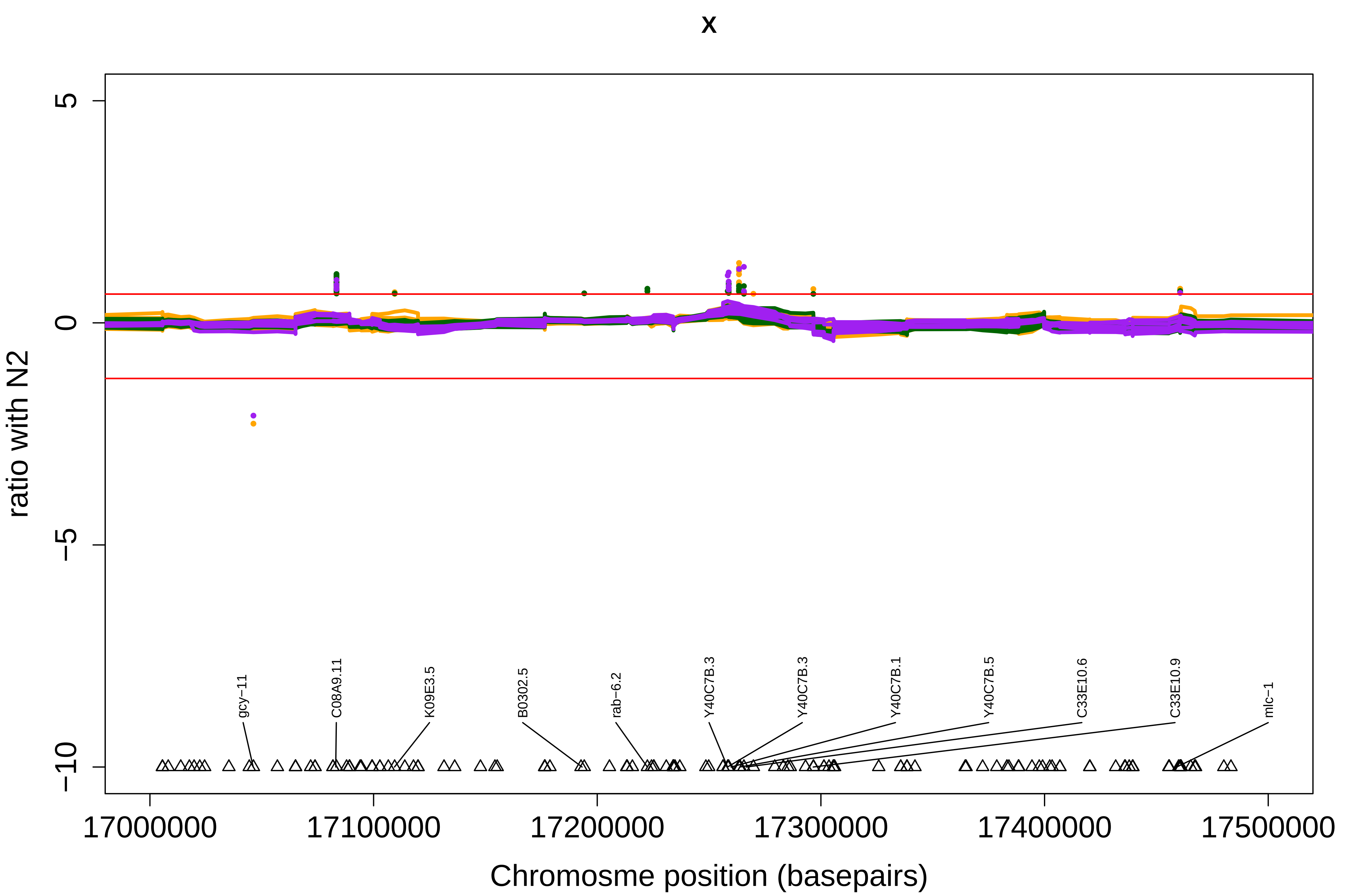
X
ratio with N2
5
0
−5
−10
gcy−11
C08A9.11
K09E3.5
B0302.5
rab−6.2
Y40C7B.3
Y40C7B.3
Y40C7B.1
Y40C7B.5
C33E10.6
C33E10.9
mlc−1
17000000
17100000
17200000
17300000
17400000
17500000
Chromosme position (basepairs)

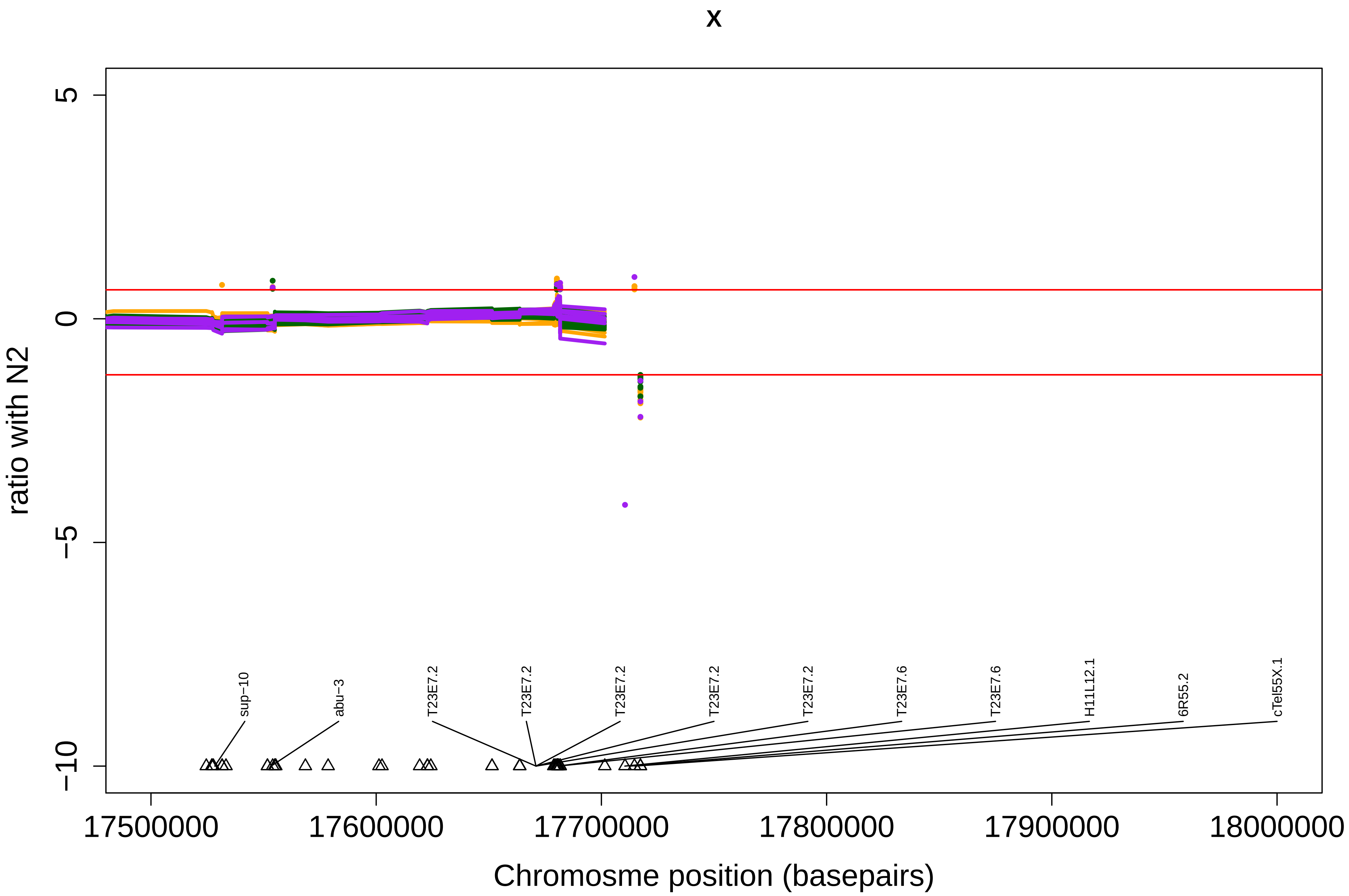

X
ratio with N2
5
0
−5
−10
sup−10
abu−3
T23E7.2
T23E7.2
T23E7.2
T23E7.2
T23E7.2
T23E7.6
T23E7.6
H11L12.1
6R55.2
cTel55X.1
17500000
17600000
17700000
17800000
17900000
18000000
Chromosme position (basepairs)

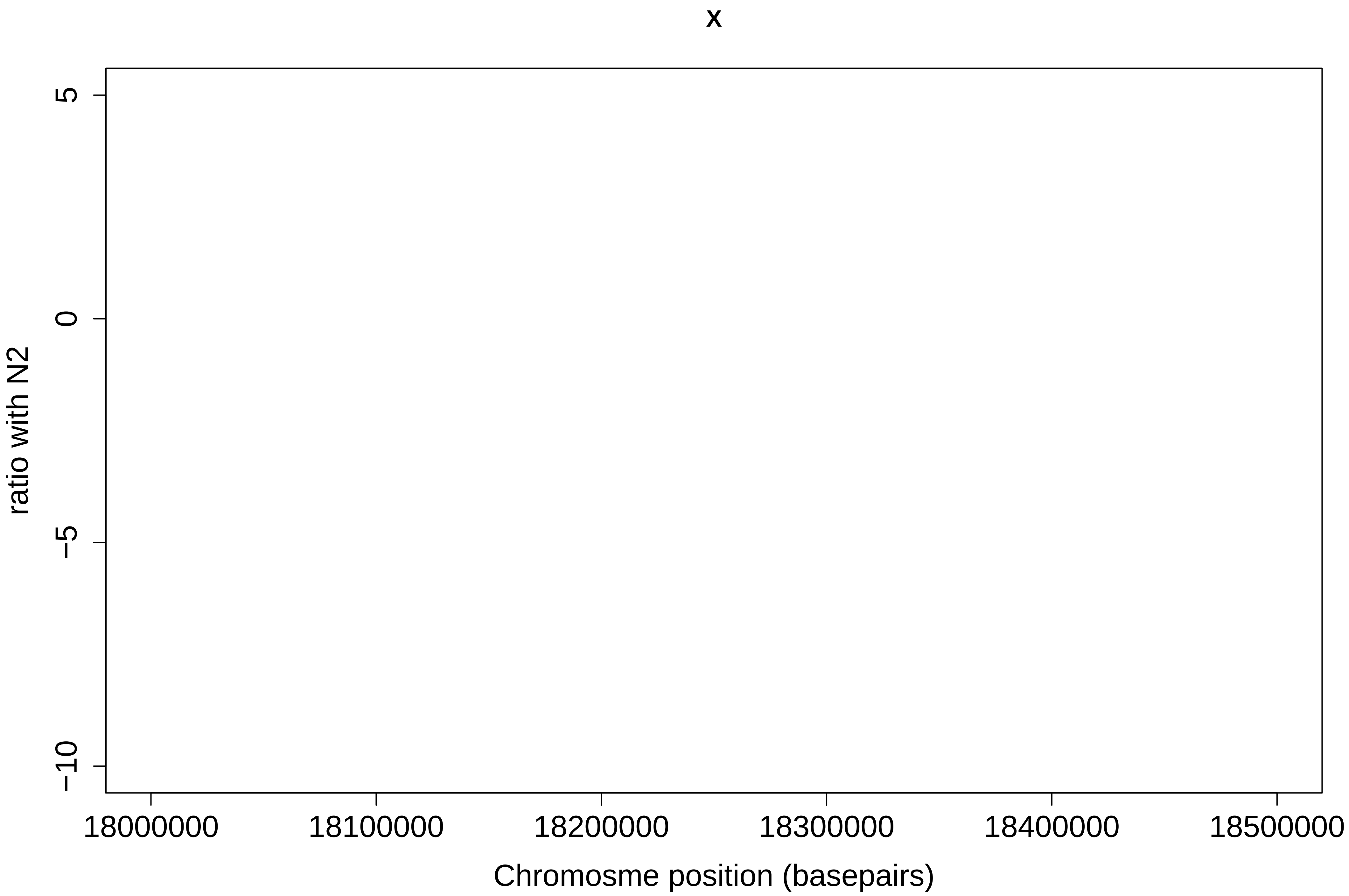
X
ratio with N2
5
0
−5
−10
18000000
18100000
18200000
18300000
18400000
18500000
Chromosme position (basepairs)
