# Supplementary figures and images for: Gene-environment and protein-degradation signatures characterize genomic and phenotypic diversity in wild Caenorhabditis elegans populations
Source: BMC Biol. 2013 Aug 19;11:93. doi: 10.1186/1741-7007-11-93 (PMC3846632; doi:10.1186/1741-7007-11-93)

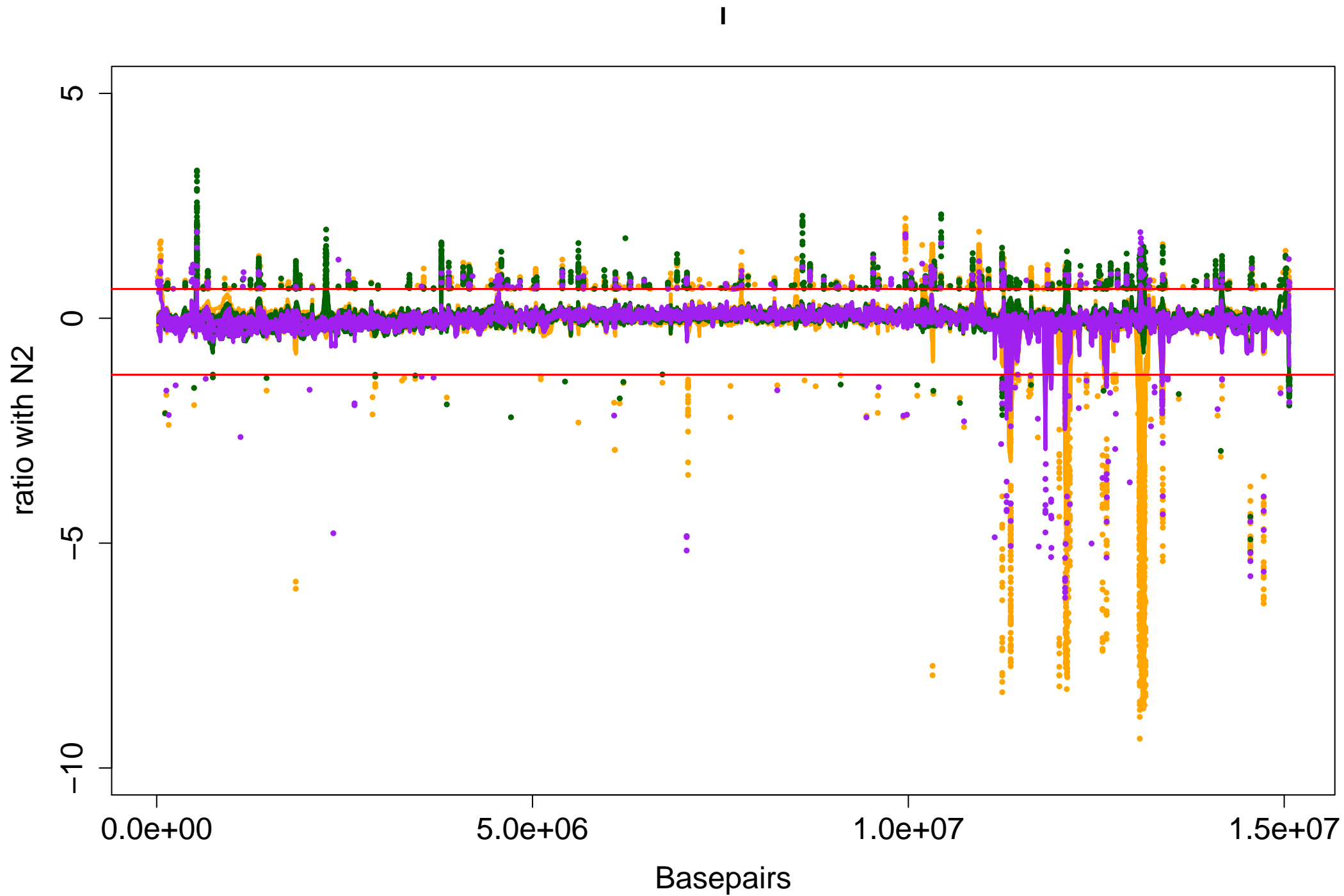

II

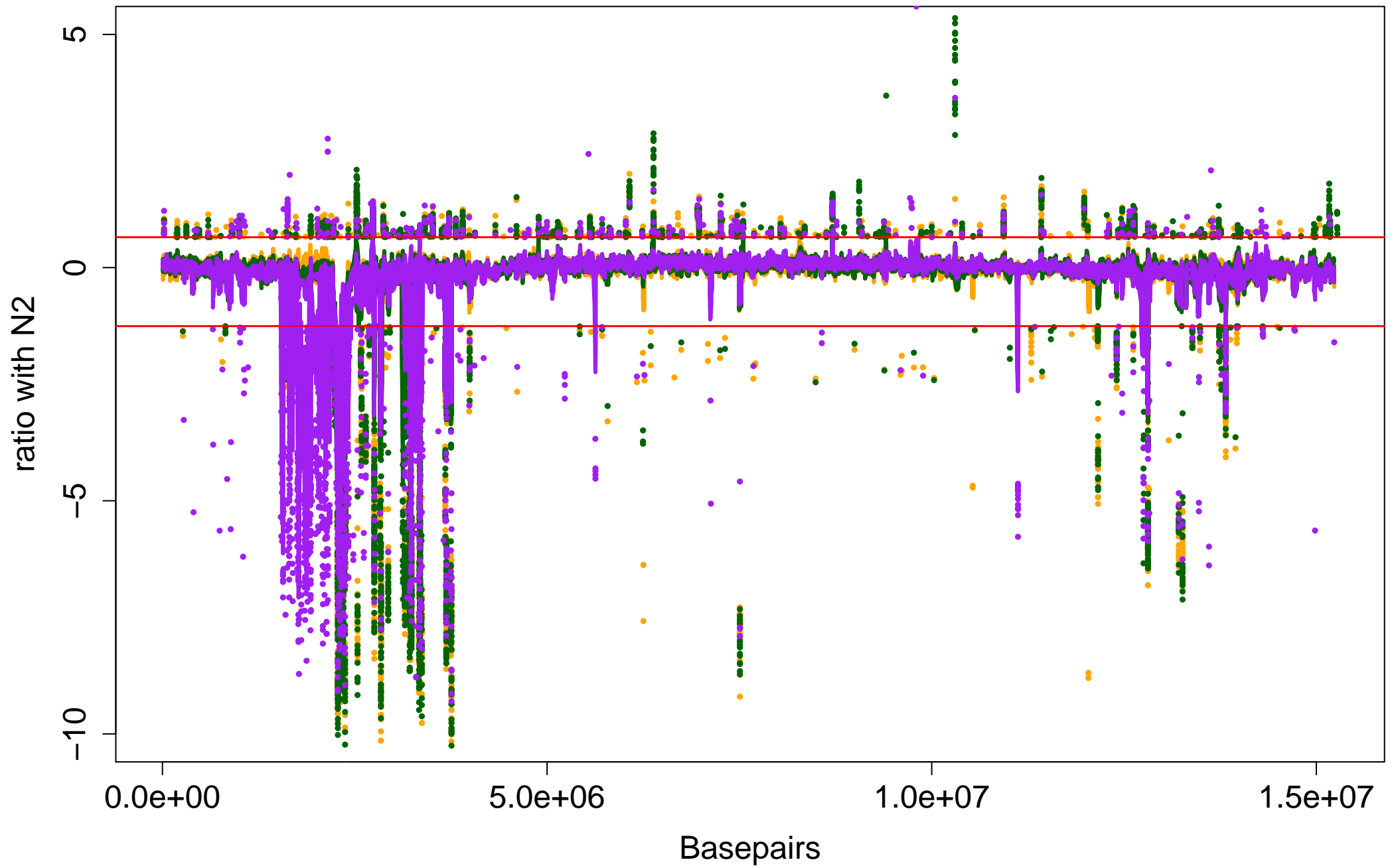

III

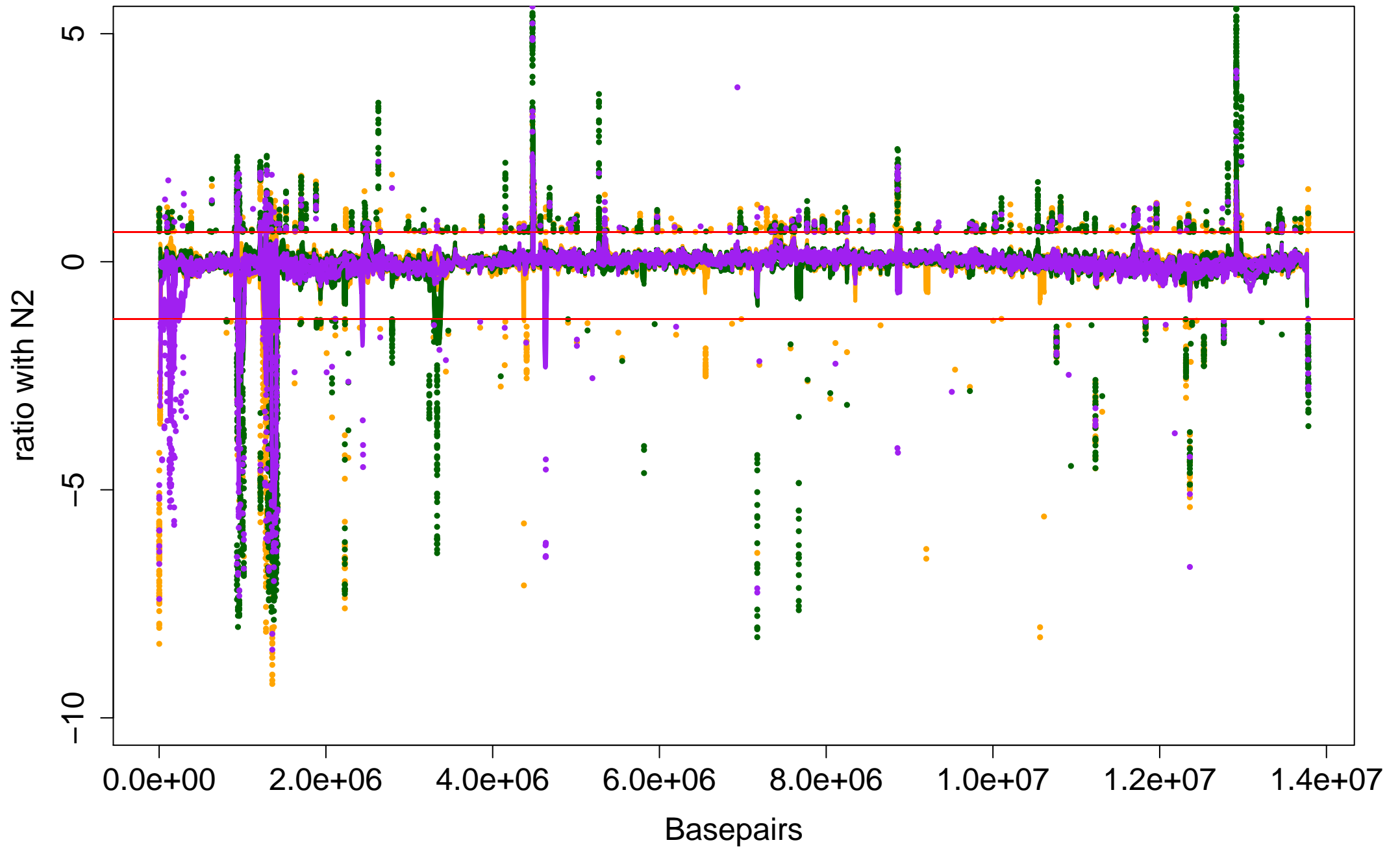

IV

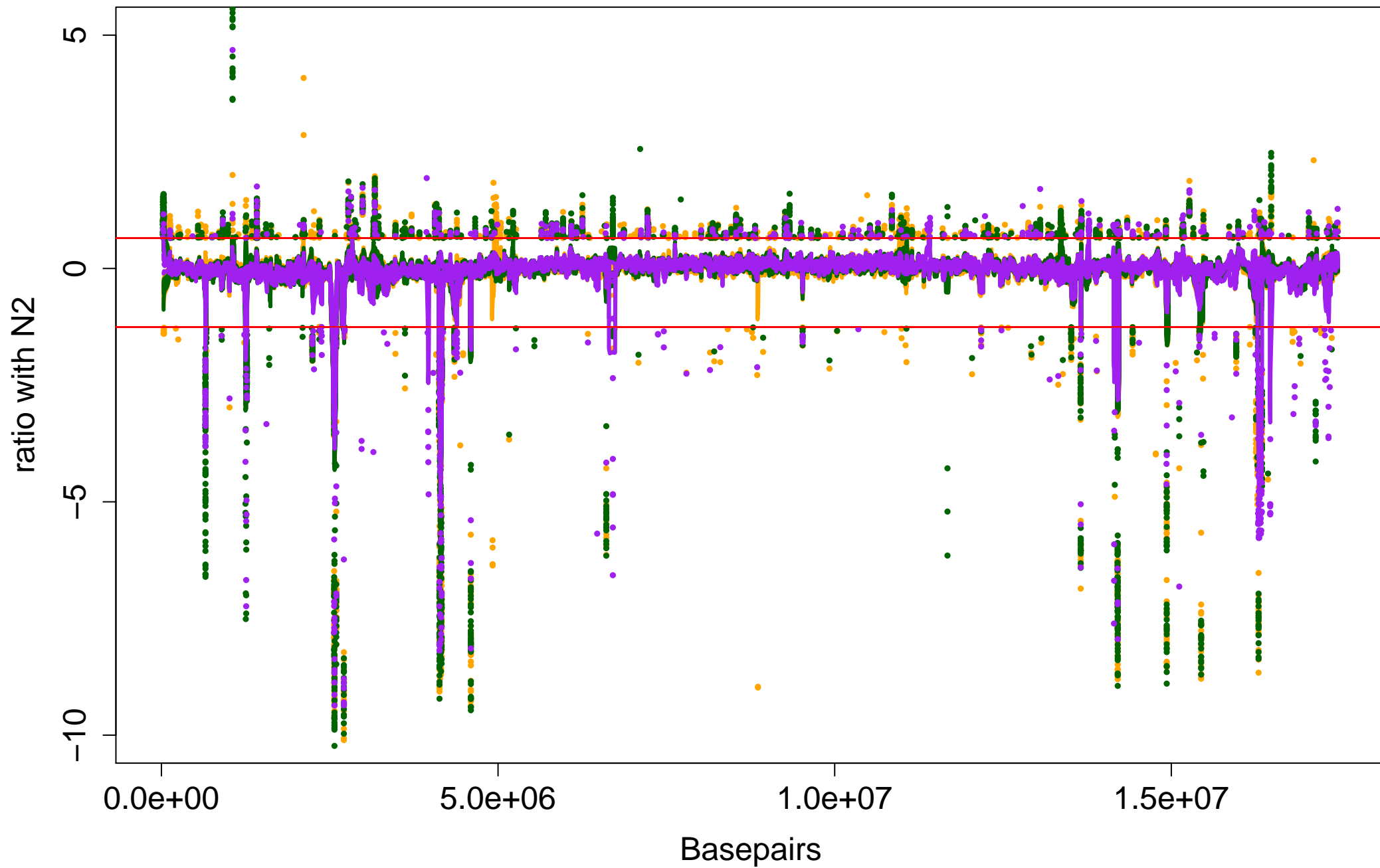

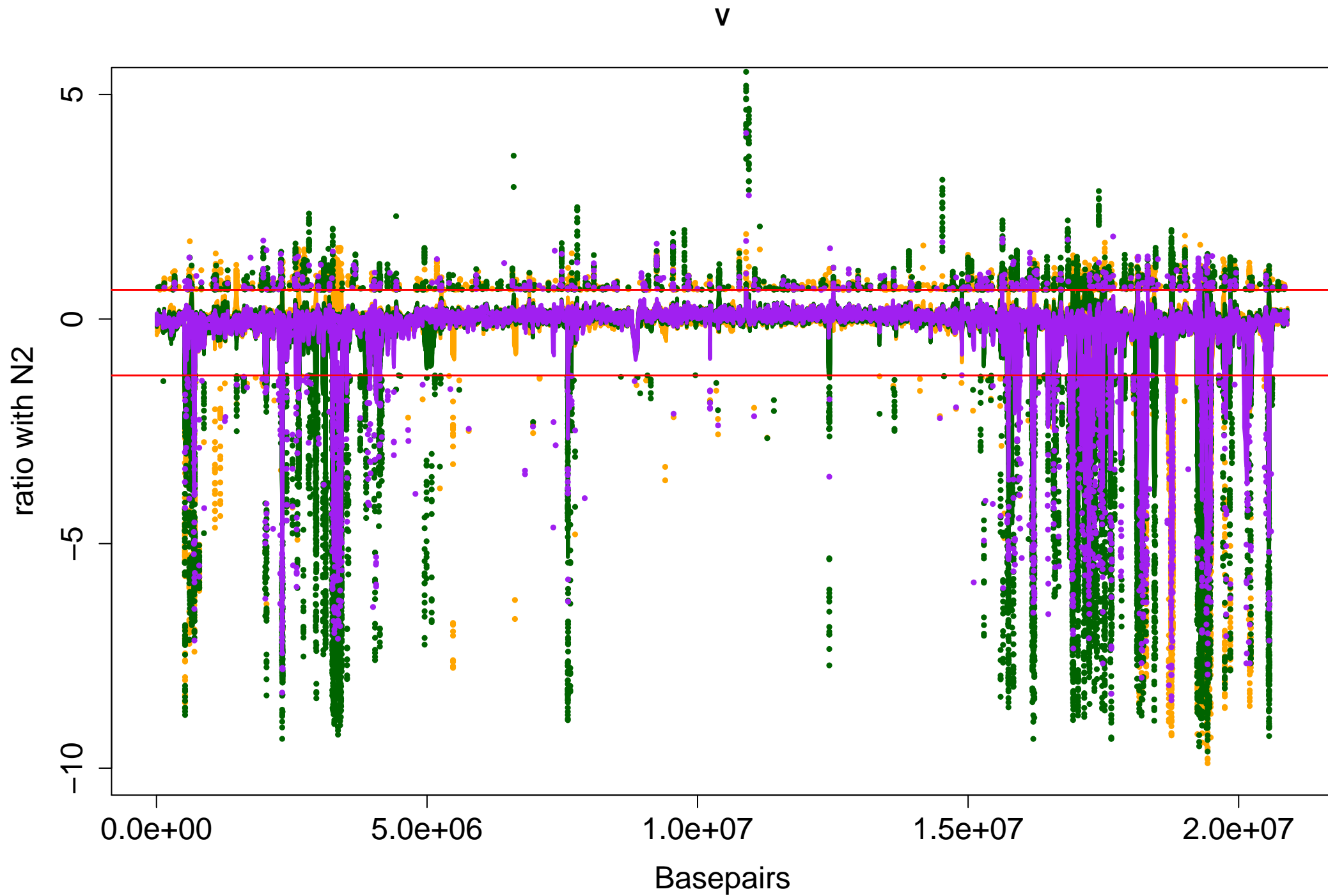

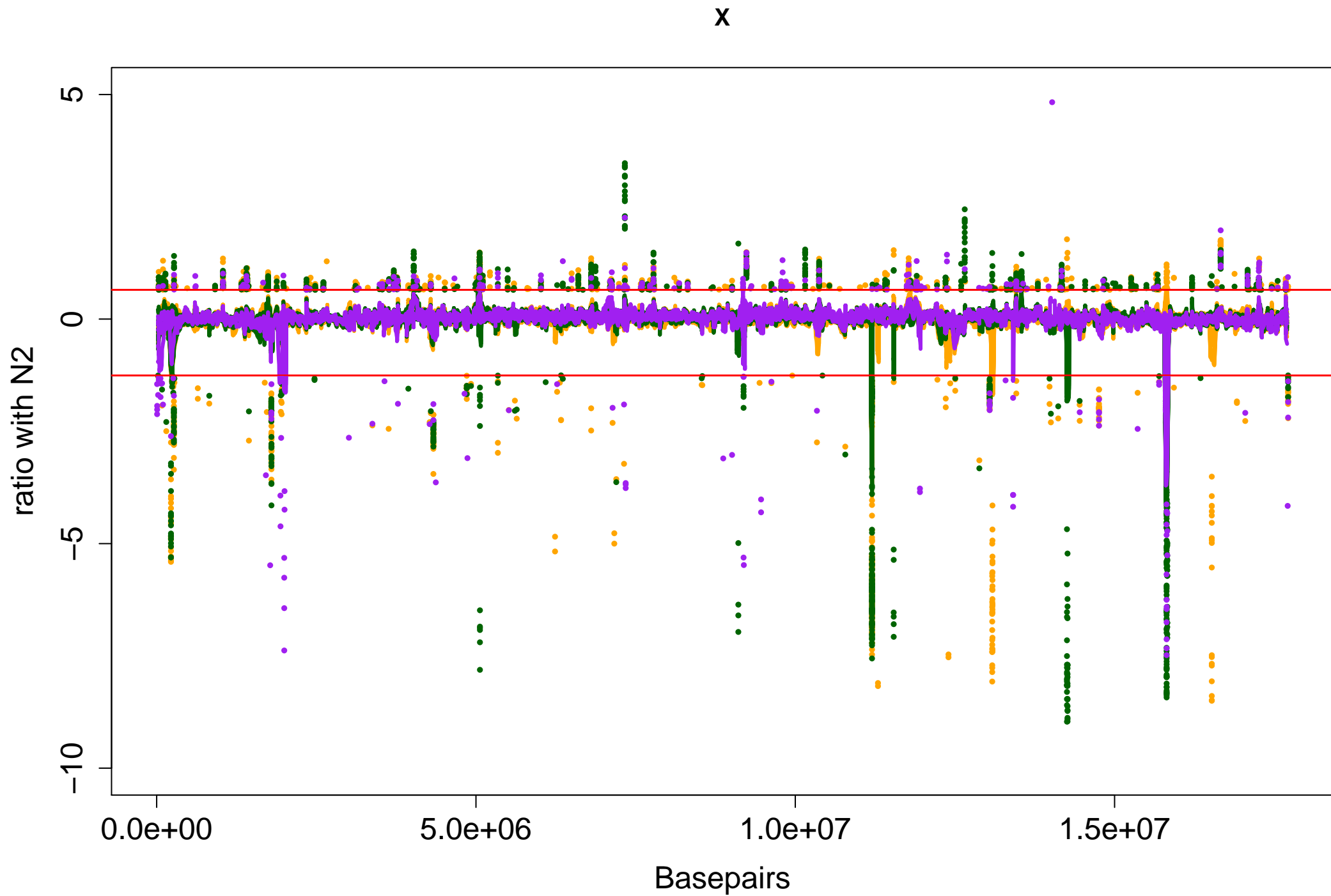

Supplement: Additional file 3 — Genome-wide overview of DNA hybridization differences per chromosome. The chromosome number is stated at the top of each page. Wild isolates from Orsay are shown in orange, wild isolates from Santeuil in green, and the out-group strains in purple. On the y-axis, the log2 ratio of the individual lines with N2 is shown as dots, and the thresholds for the moving averages are shown as horizontal red lines. [file 1741-7007-11-93-S3.pdf]

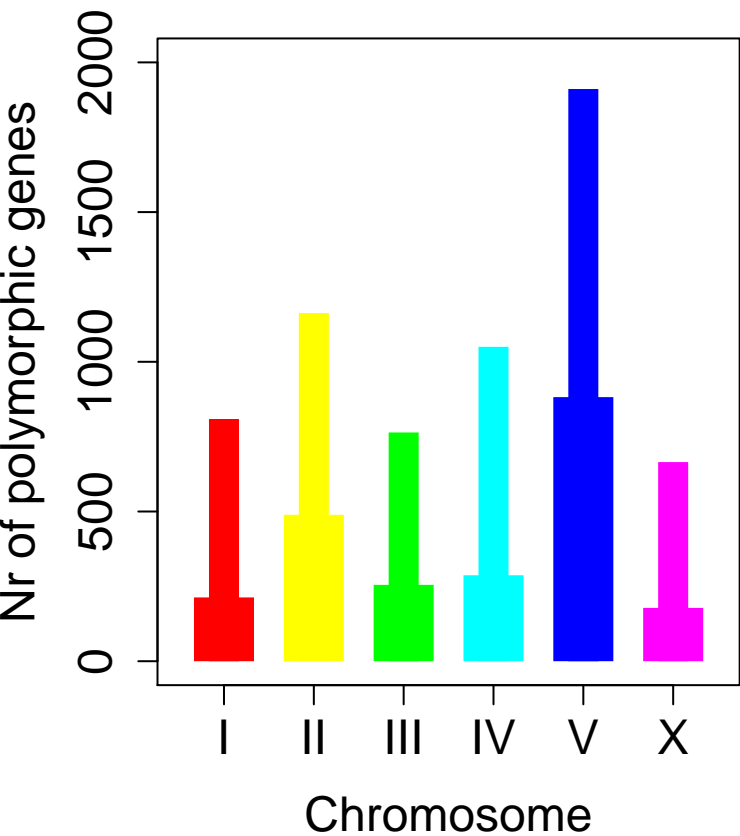

Supplement: Additional file 4 — Number of polymorphic genes per chromosome. The wide part of the bars shows the number of genes with an absolute ratio greater than 1, while the narrow part shows the number of genes with an absolute ratio greater than 0.5. Total number of genes per chromosome (with percentage of polymorphic genes (ratio >0.5) in parentheses): chromosome I, 2,969 genes (28%); II: 3,588 (32%); III: 2,680 (28%); IV: 3,435 (30%); V: 5,400 (35%); X: 2,809 (24%). [file 1741-7007-11-93-S4.pdf]

**A**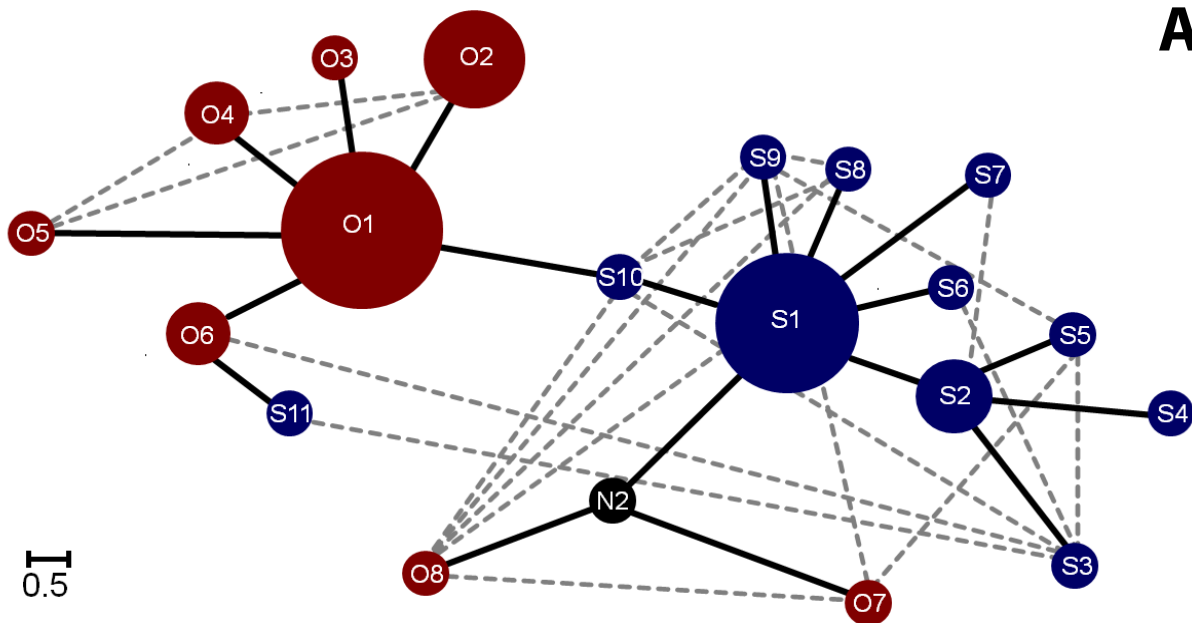**B**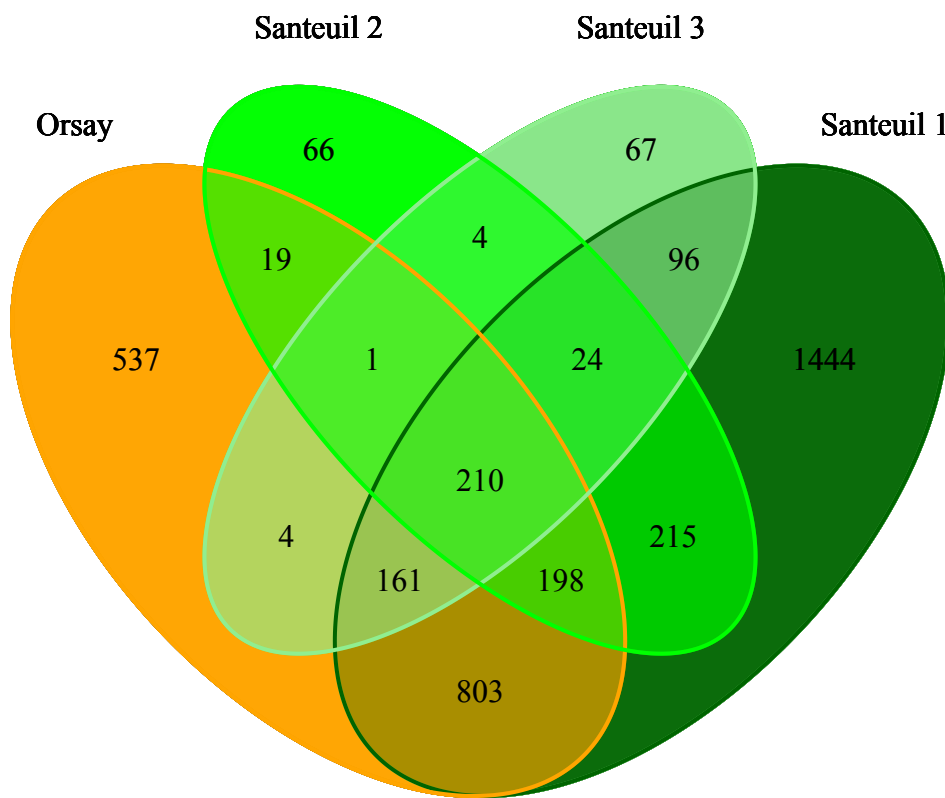

Supplement: Additional file 5 — Minimum spanning network and Venn diagram. (A) Minimum spanning network constructed using microsatellite data. The letter ‘O’ and red color refers to Orsay; S and blue to Santeuil. Circle size is proportional to the number of strains with a particular genotype. The solid lines show the main relationships among genotypes, while the dotted lines show alternative connections. Line length correlates with the inferred number of evolutionary differences. The minimum spanning network was reconstructed with the software program Arlequin. (B) Venn diagram of genes for which DNA hybridization intensity per genetic group was significantly different from that of the the out-group. Total number of genes for each strain: Orsay: 1,933; Santeuil 1: 3,181; Santeuil 2: 737; Santeuil 3: 567. Group S1 appeared to be the most divergent from the out-group, with 3,181 genes that differed significantly. A large part of these genes (803) was also shared with the Orsay group. The number of significantly different genes that were the same for S3 and S2 or O (4 in both cases) and for S2 and O (1 gene) was remarkably low. S2 and O also shared a small number (n =19) of the same significantly different genes. [file 1741-7007-11-93-S5.pdf]

# A

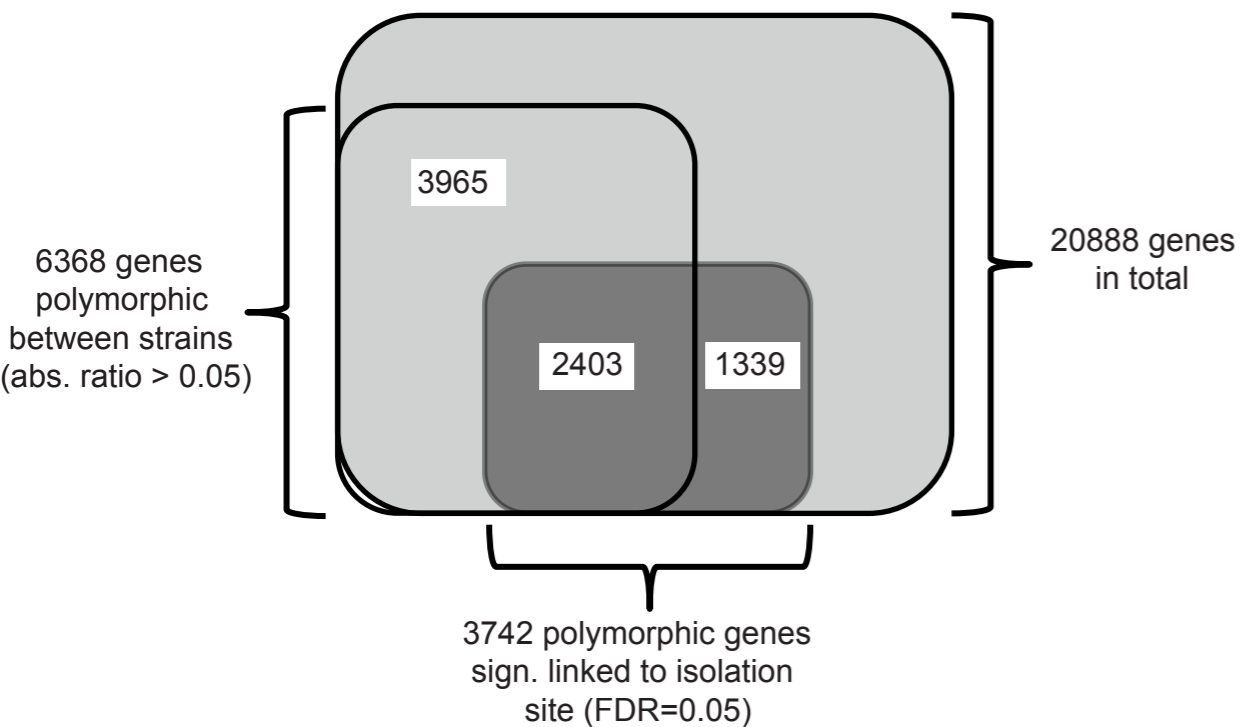

Percentage of all genes in group

# B

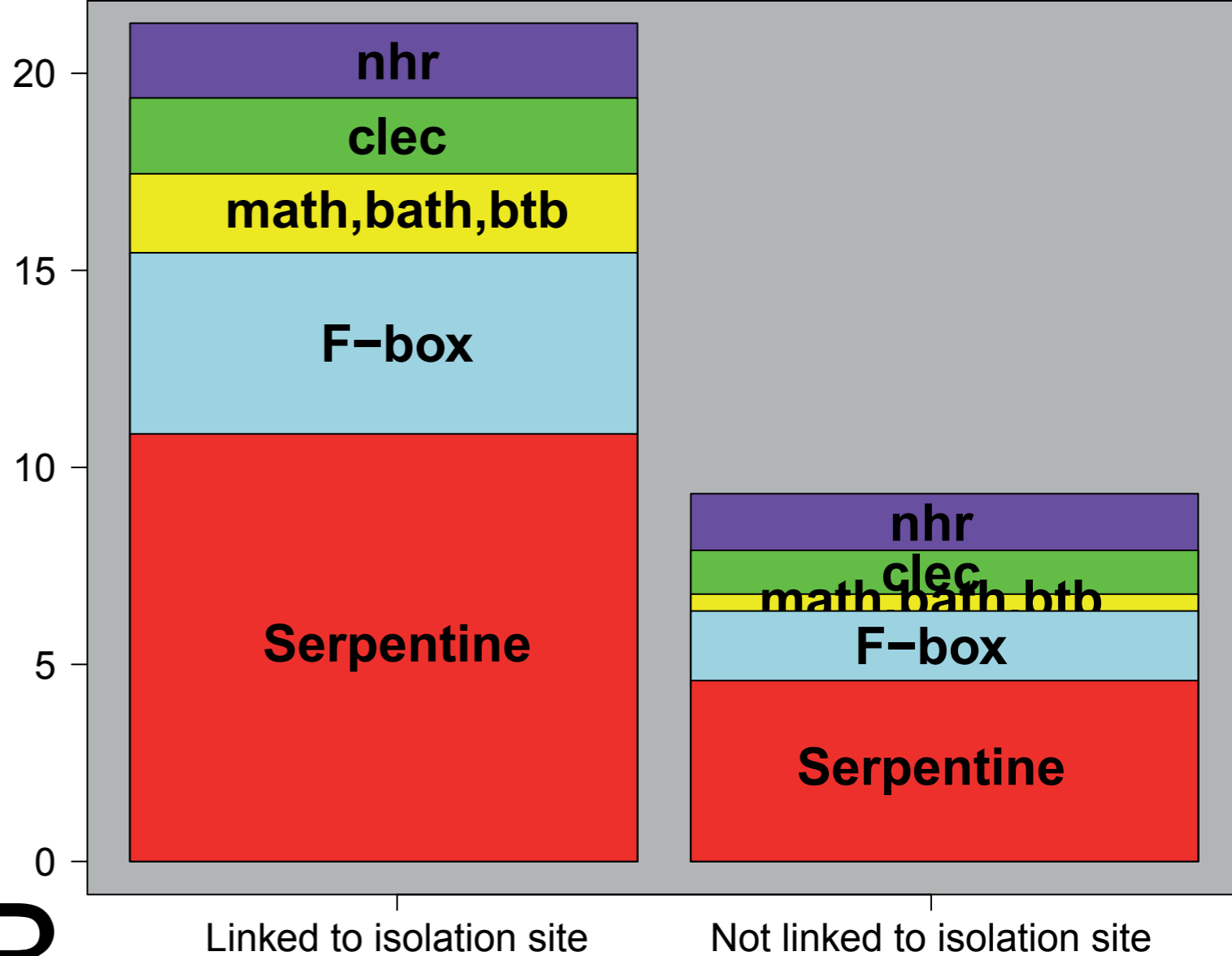

Supplement: Additional file 6 — Polymorphic genes and genes linked or not linked to isolation sites. (A) Schematic overview of the groups of polymorphic genes based on DNA hybridization data. (B) Percentage of all genes detected using hybridization of genomic C. elegans DNA on microarrays that were linked or not linked to the isolation sites. Together, the gene classes serpentine receptors, F-box, math, bath, btb, clec, and nhr composed almost 25% of the polymorphic genes significantly linked to isolation site. These same gene classes made up less than 10% of the genes that could not be linked to isolation site. [file 1741-7007-11-93-S6.pdf]

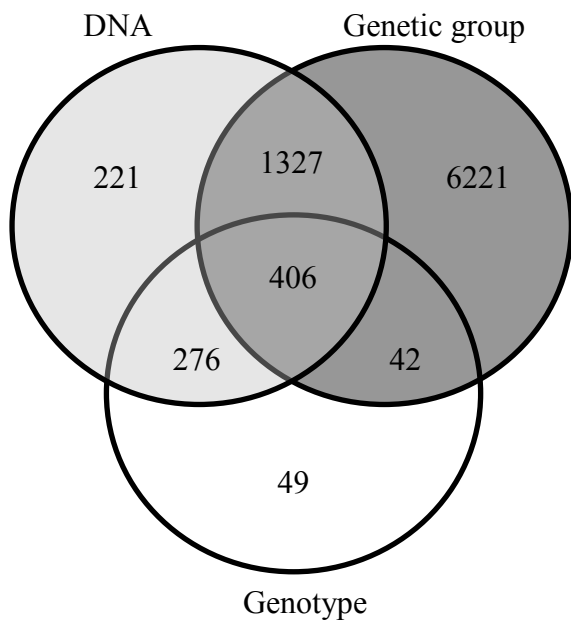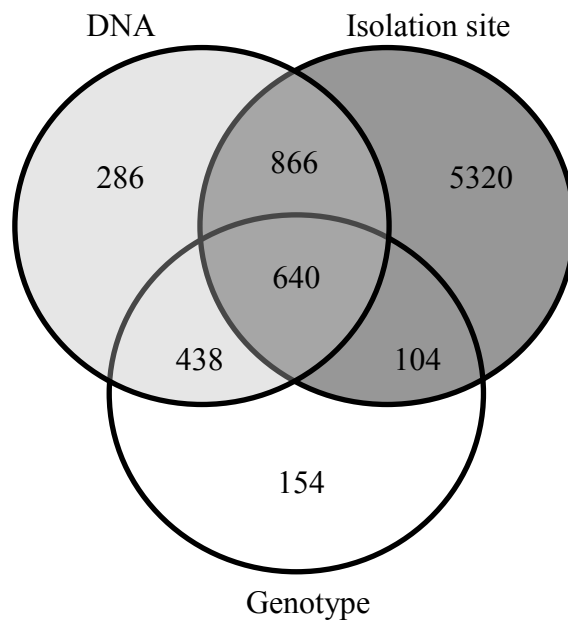

Supplement: Additional file 7 — Venn diagram of the genes that showed expression differences due to DNA polymorphisms and genotype in combination with either genetic group (O, S1, S2, and S3) or isolation site (Santeuil and Orsay). Total number of genes: genotype (left diagram): 773; genotype (right diagram): 1,336; DNA: 2,230; genetic group: 7,996; isolation site: 6,930. [file 1741-7007-11-93-S7.pdf]

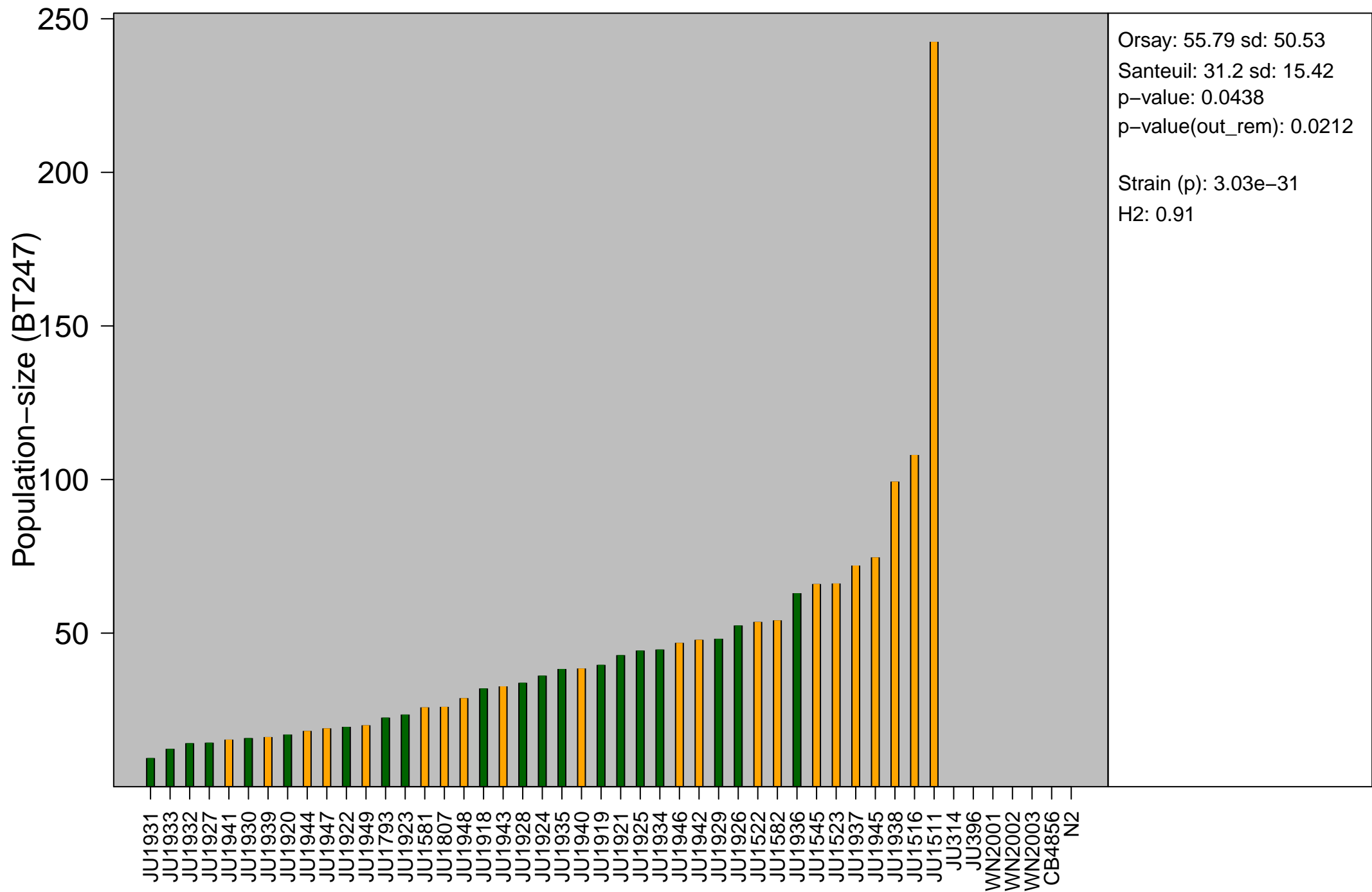

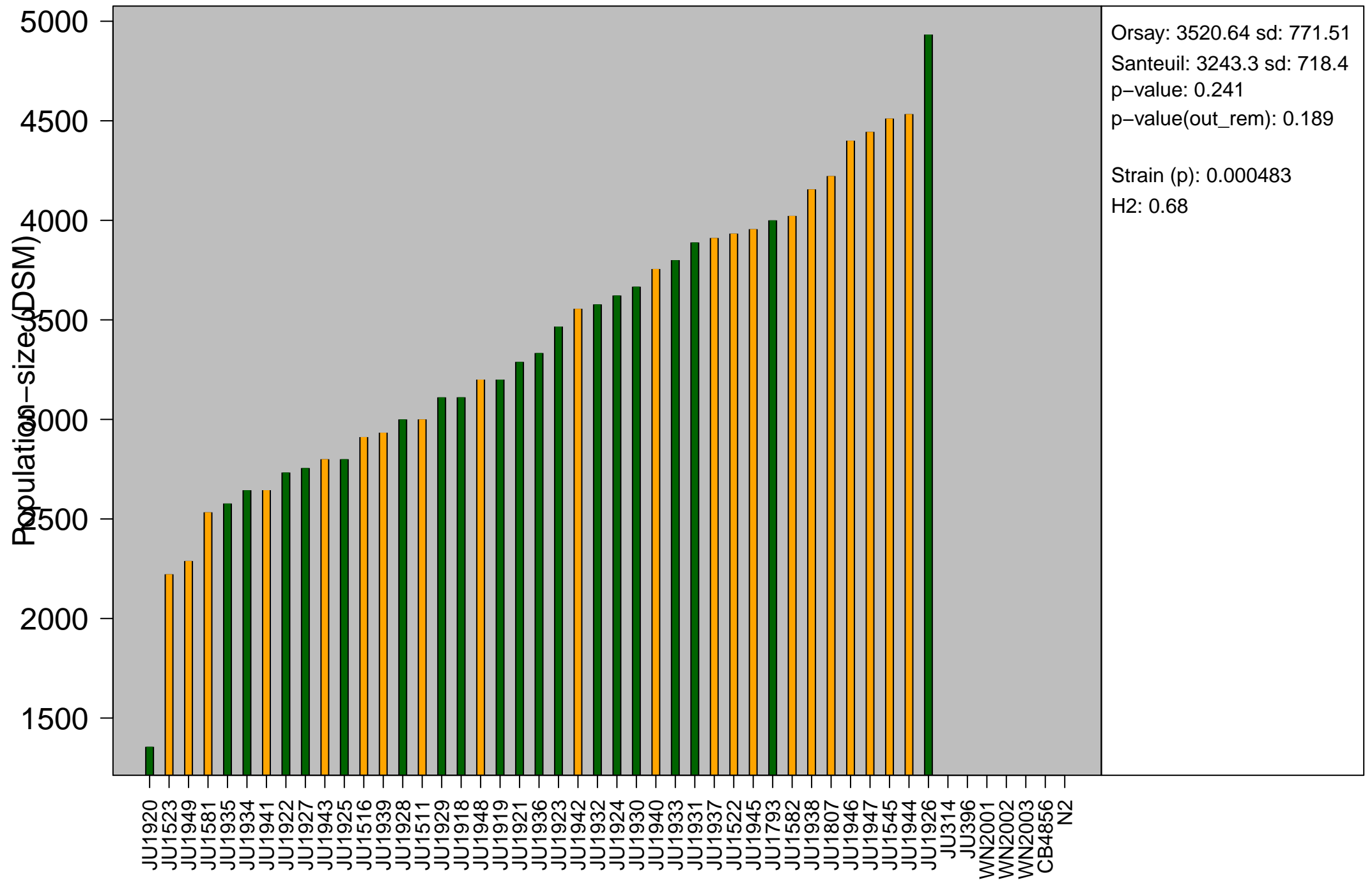

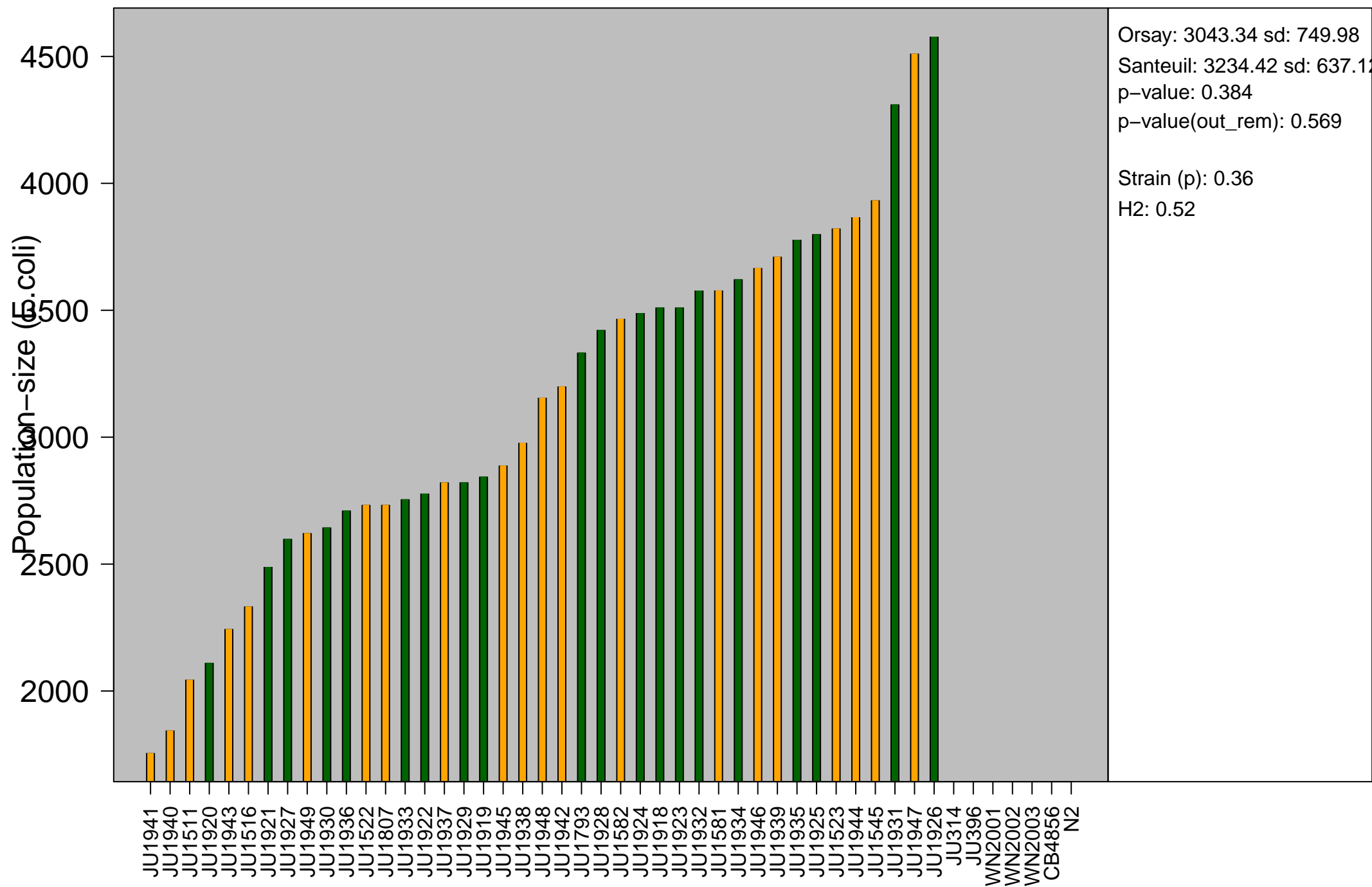

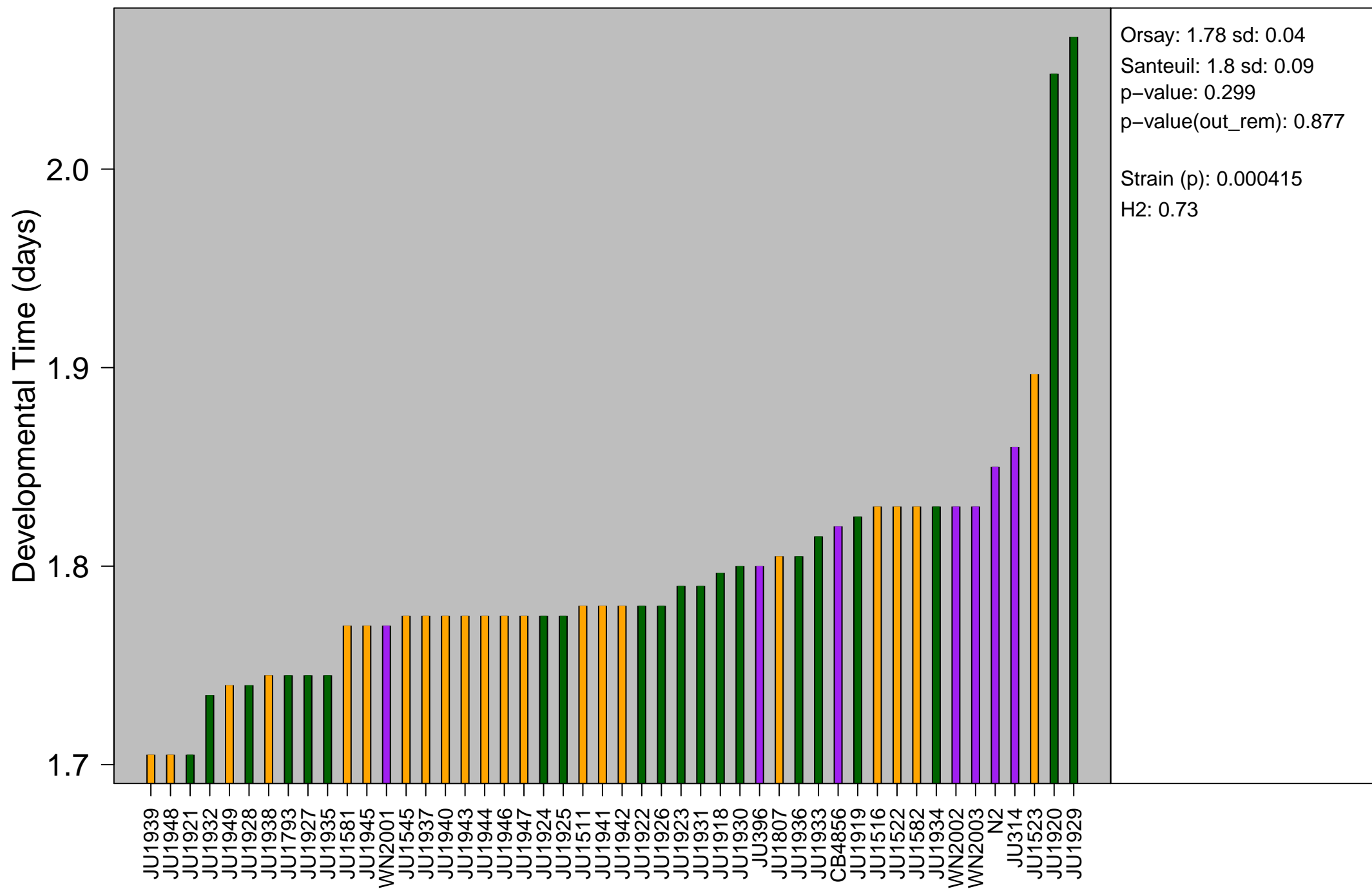

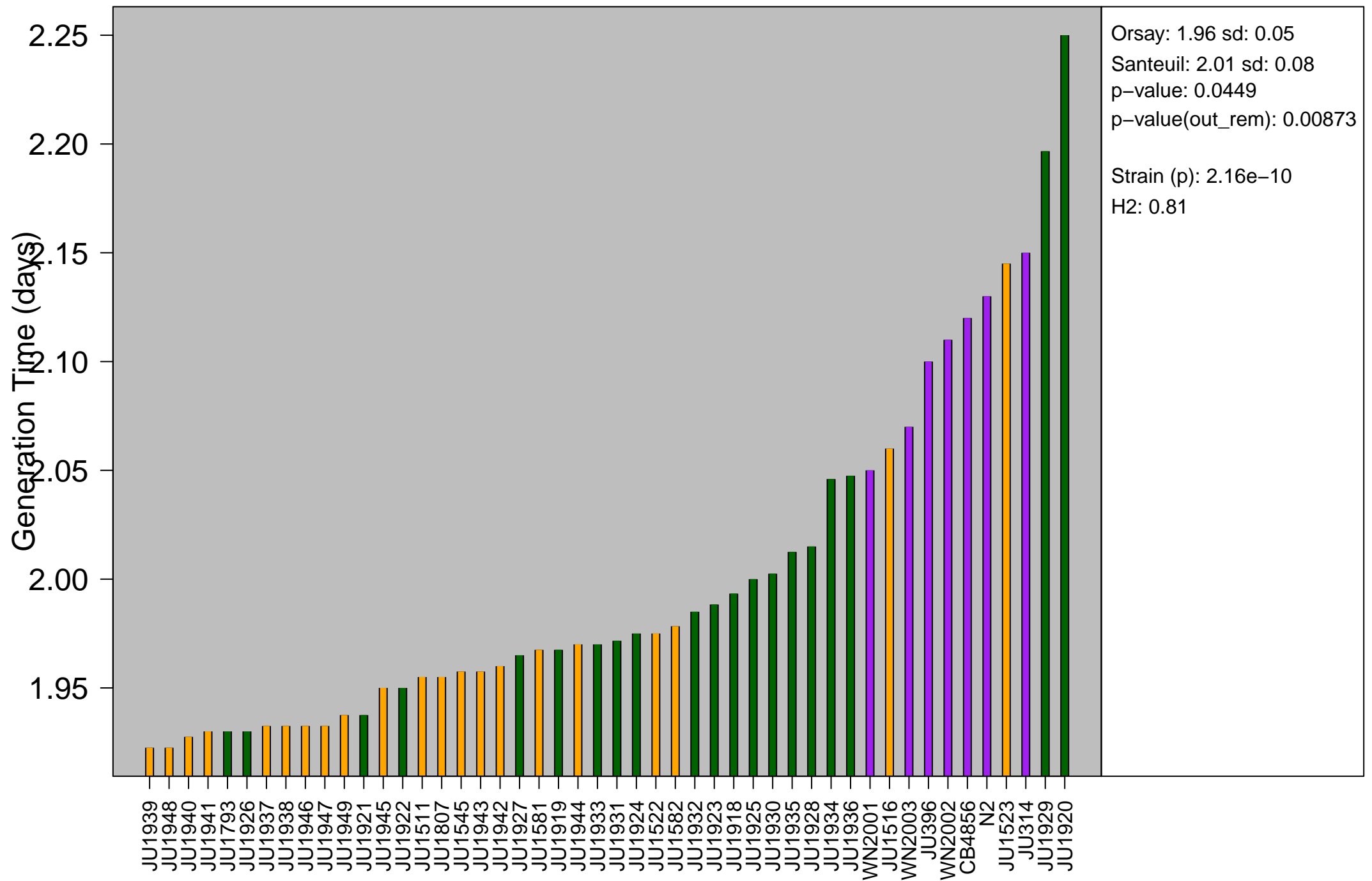

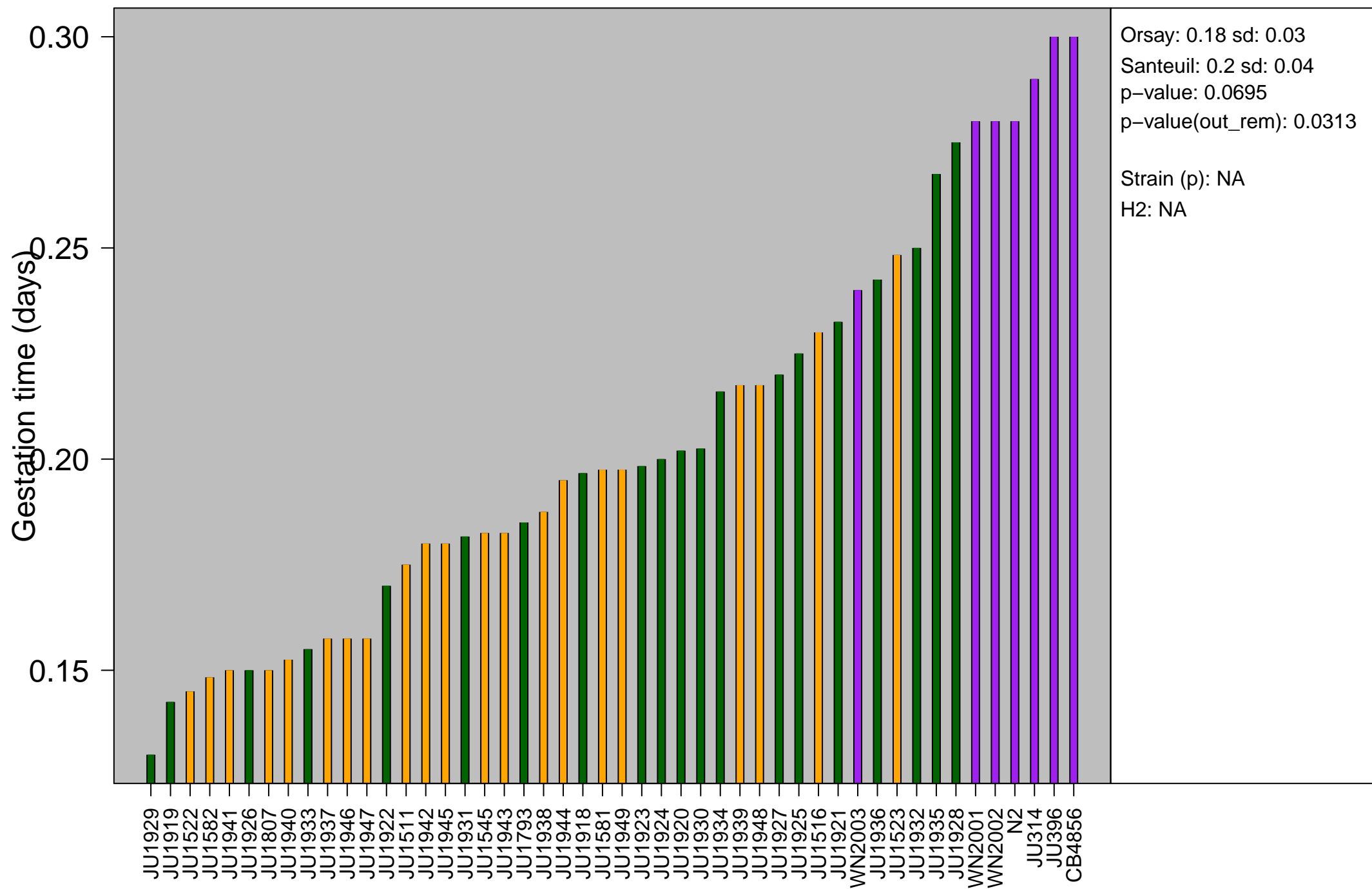

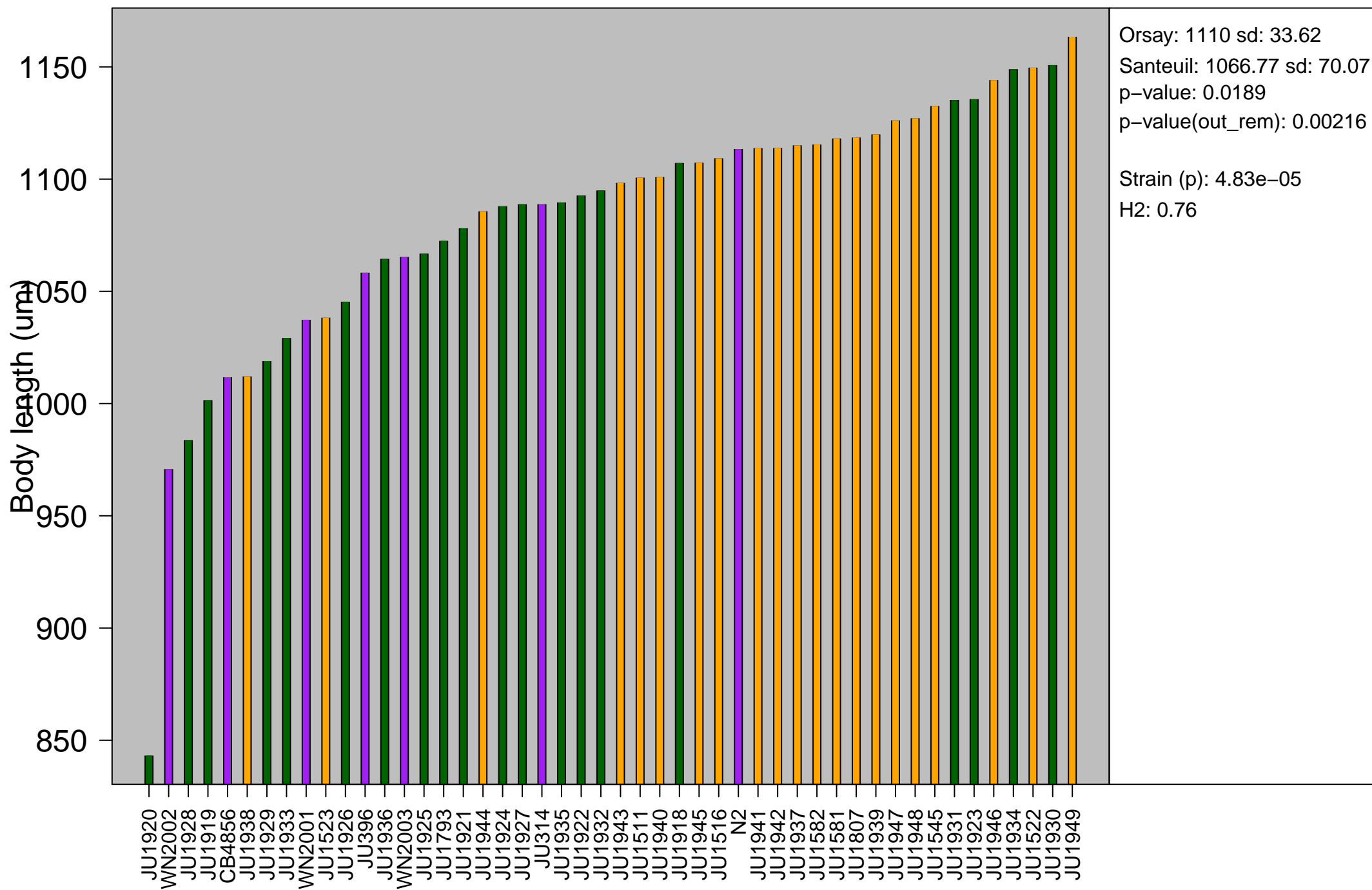

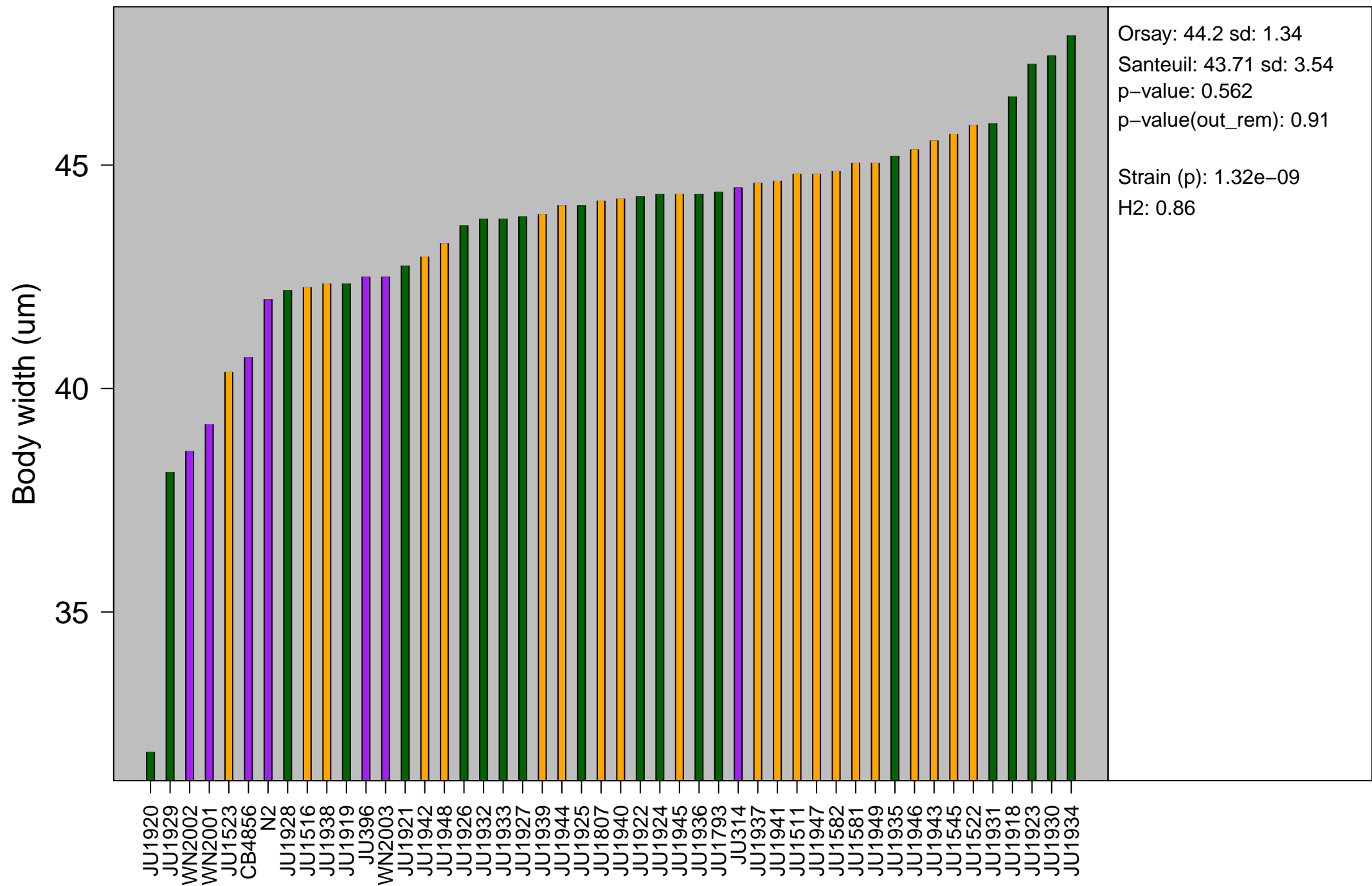

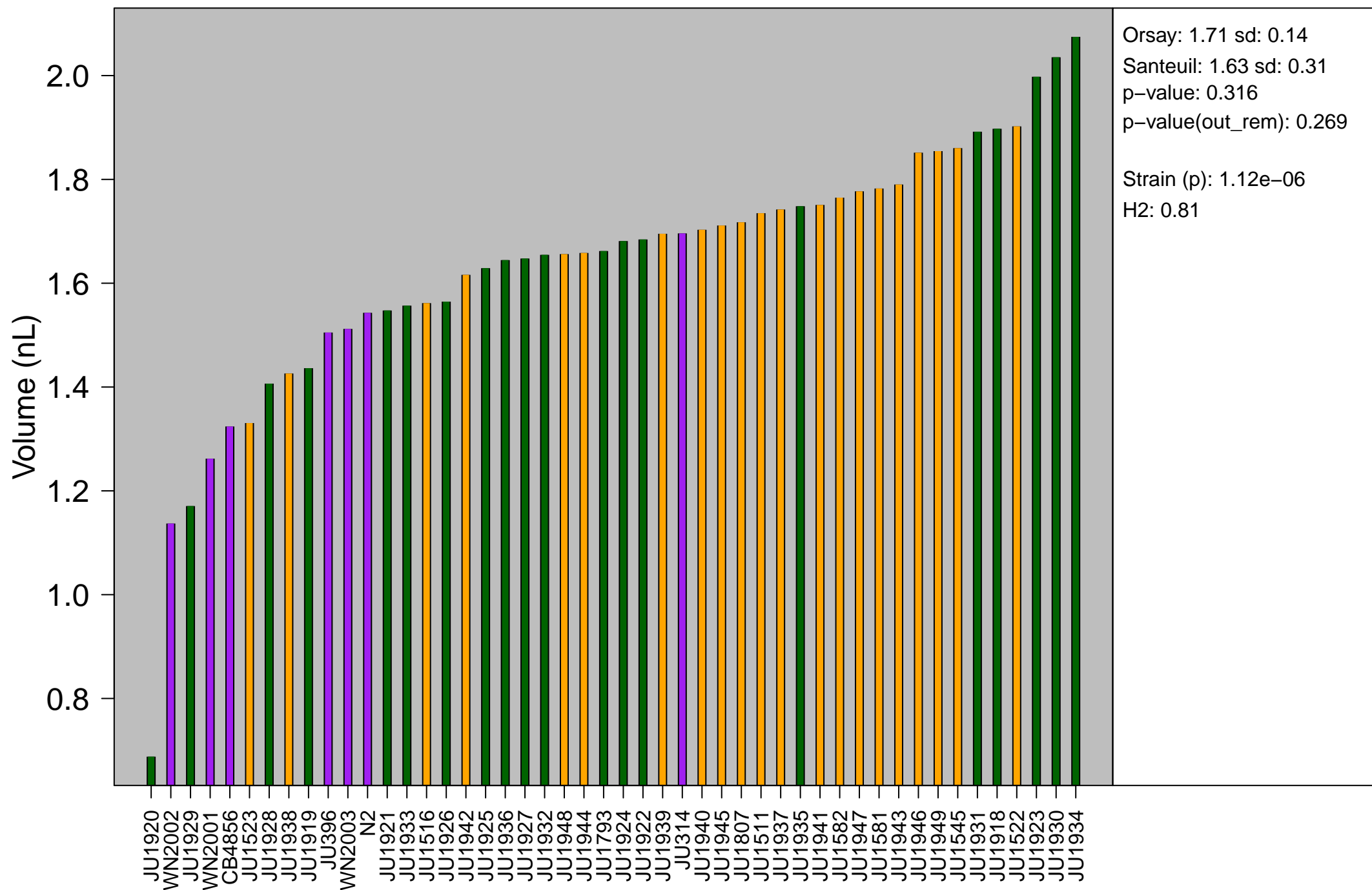

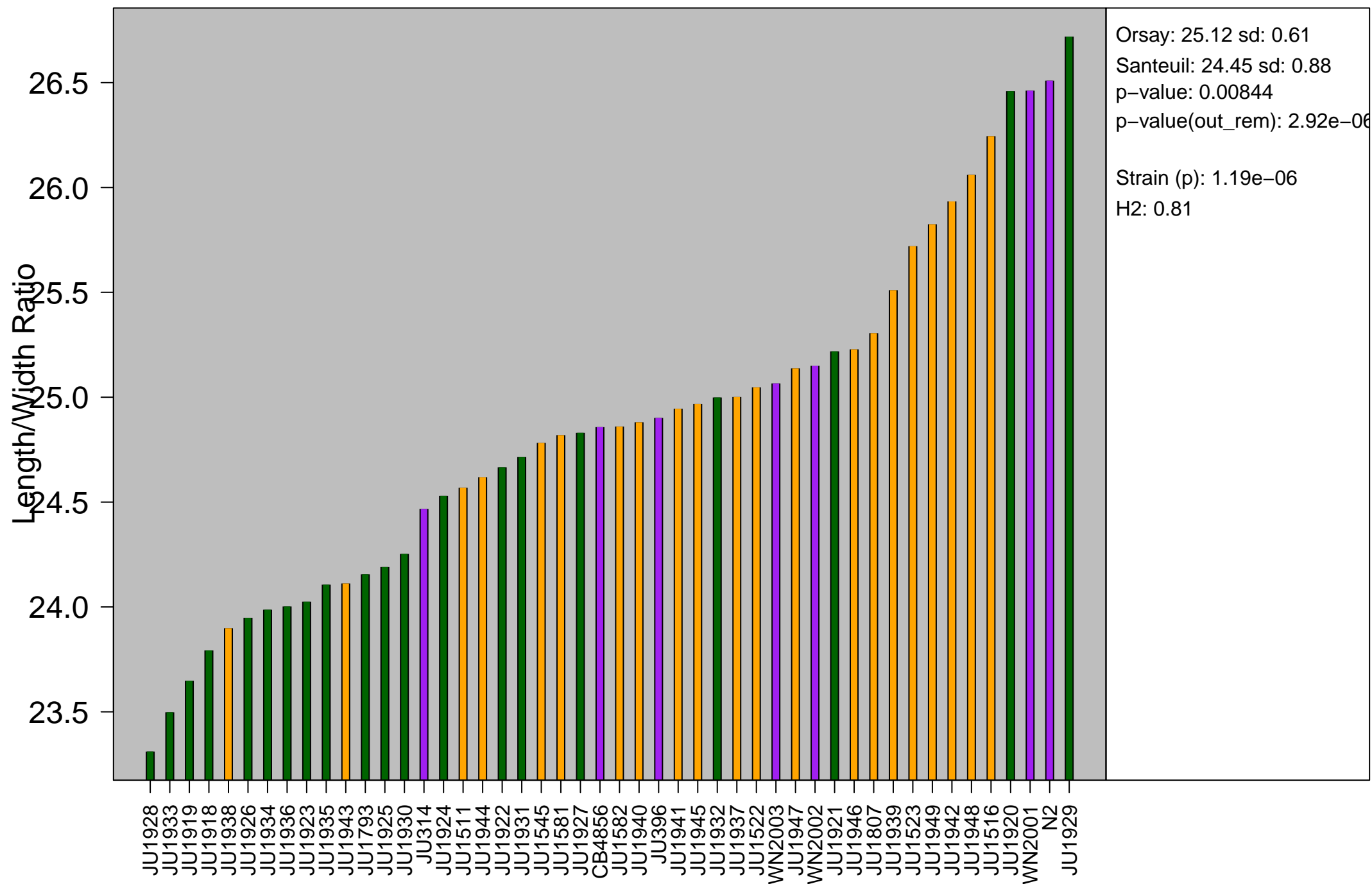

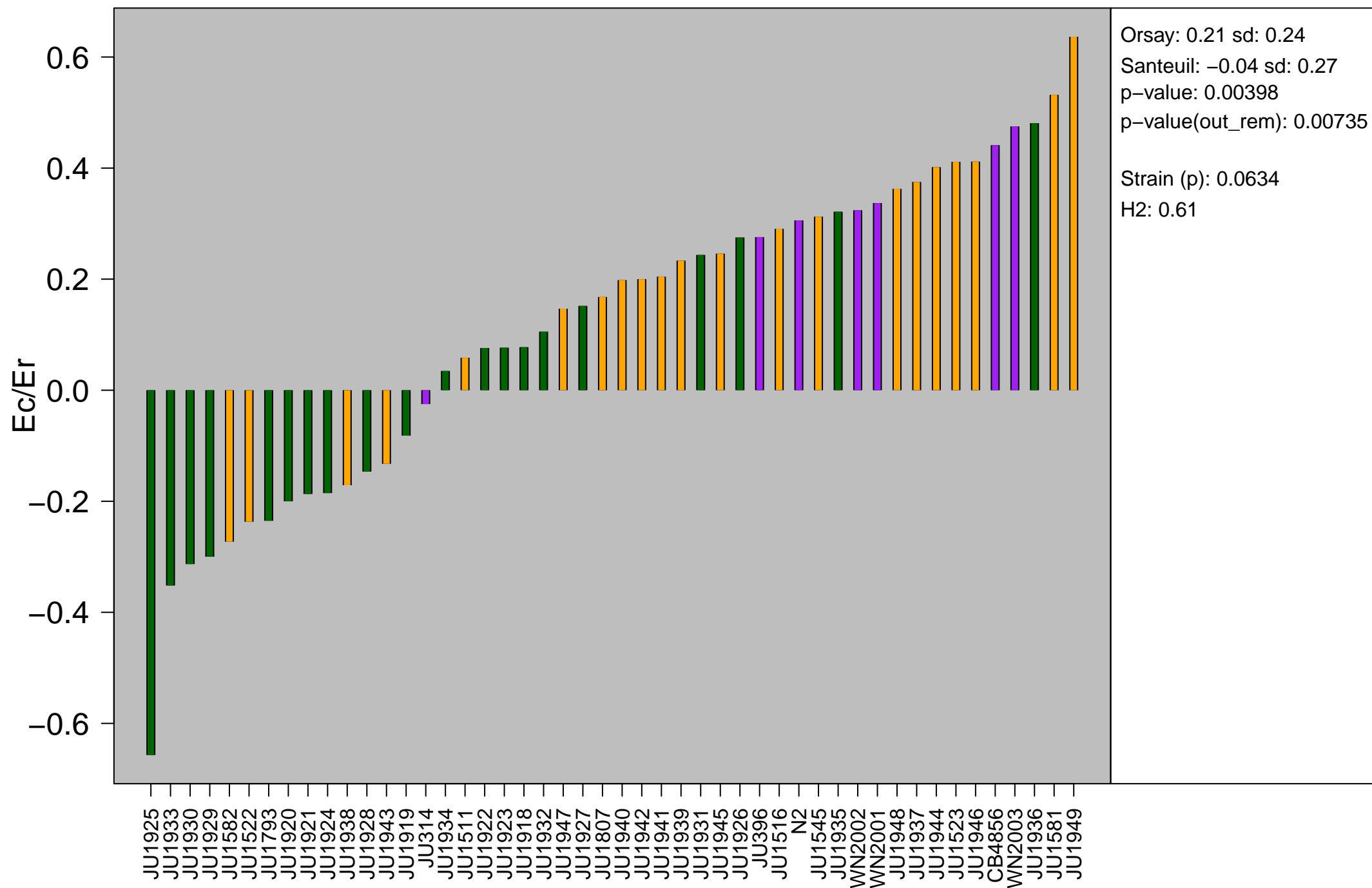

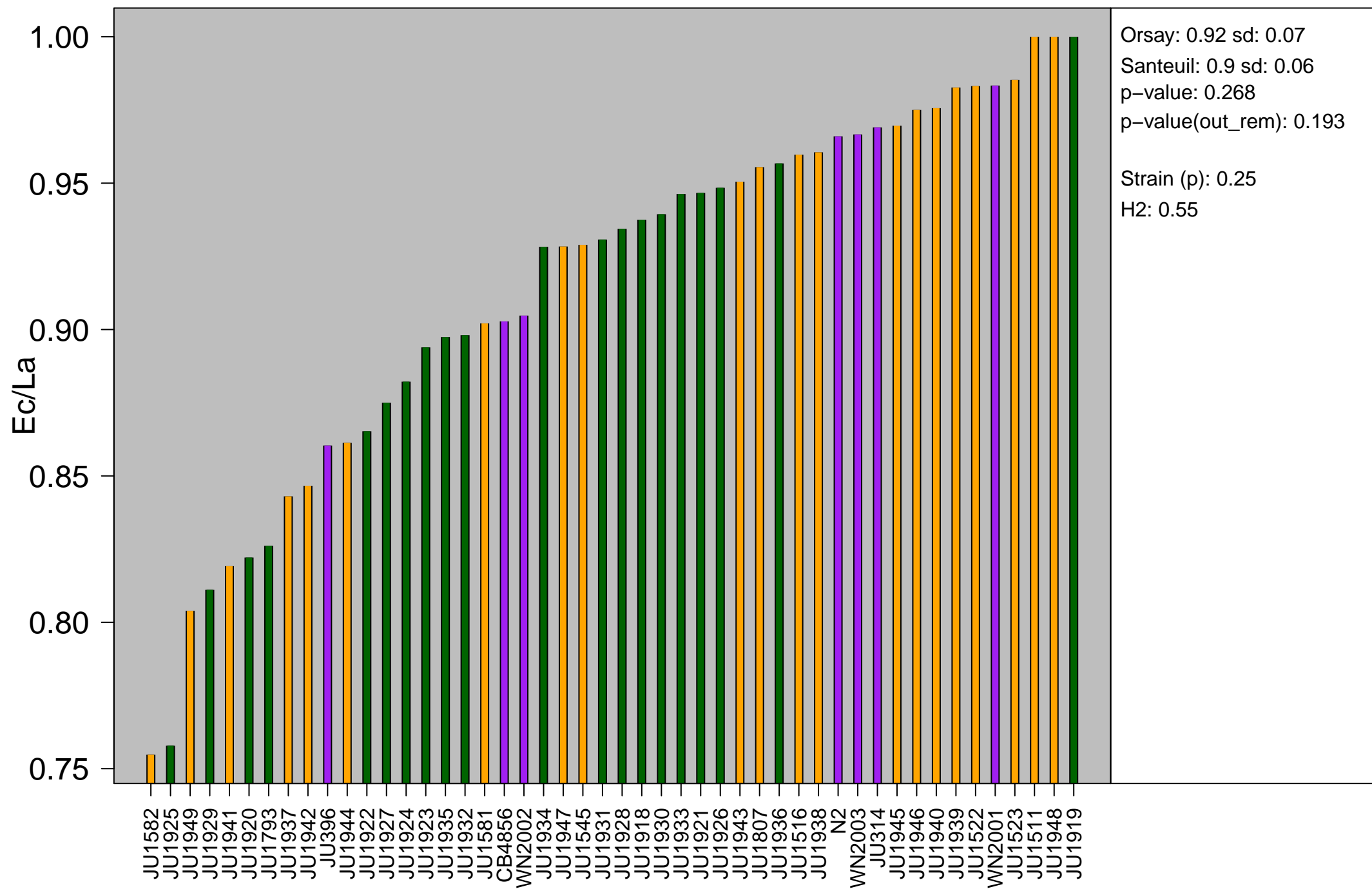

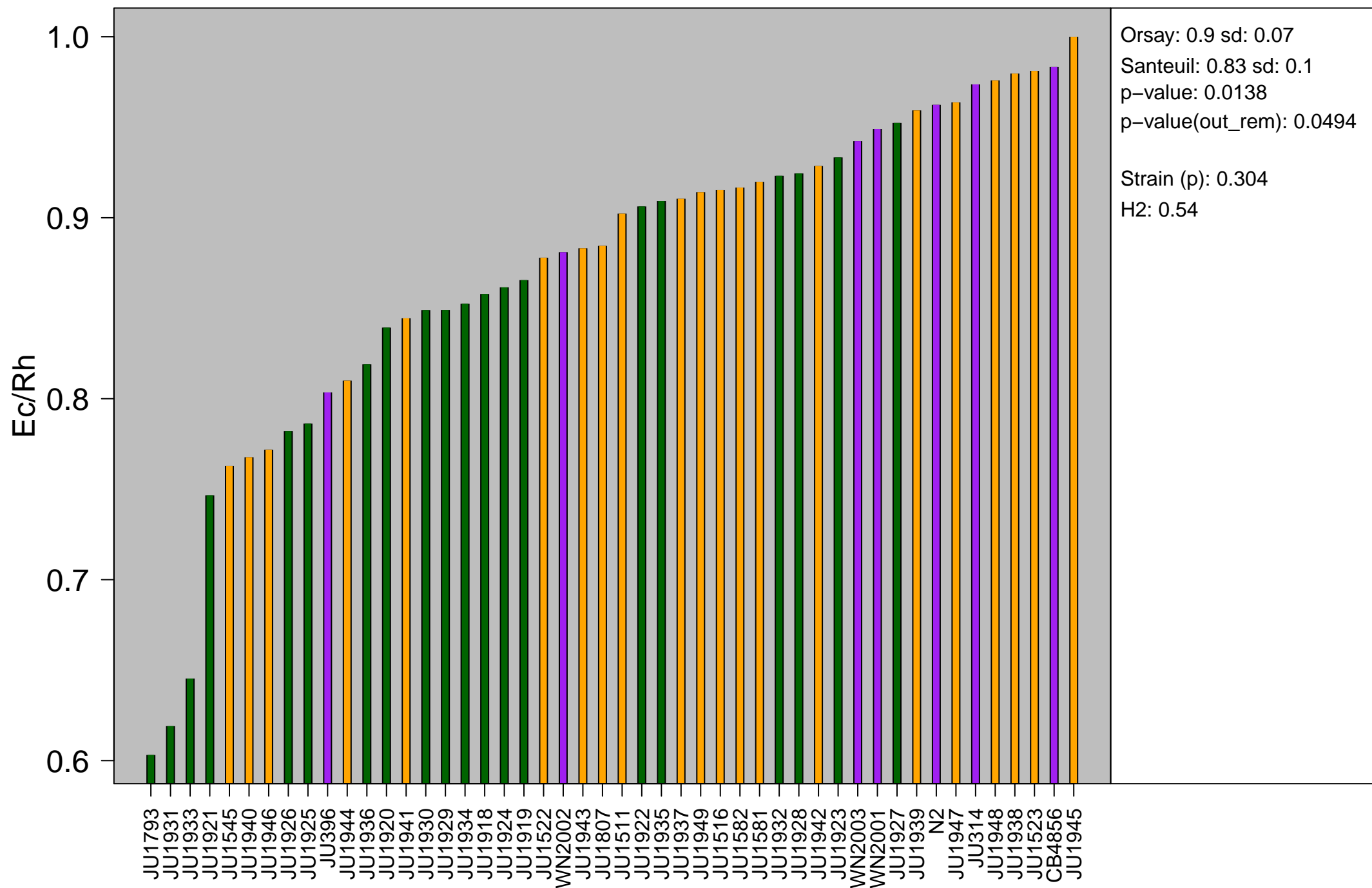

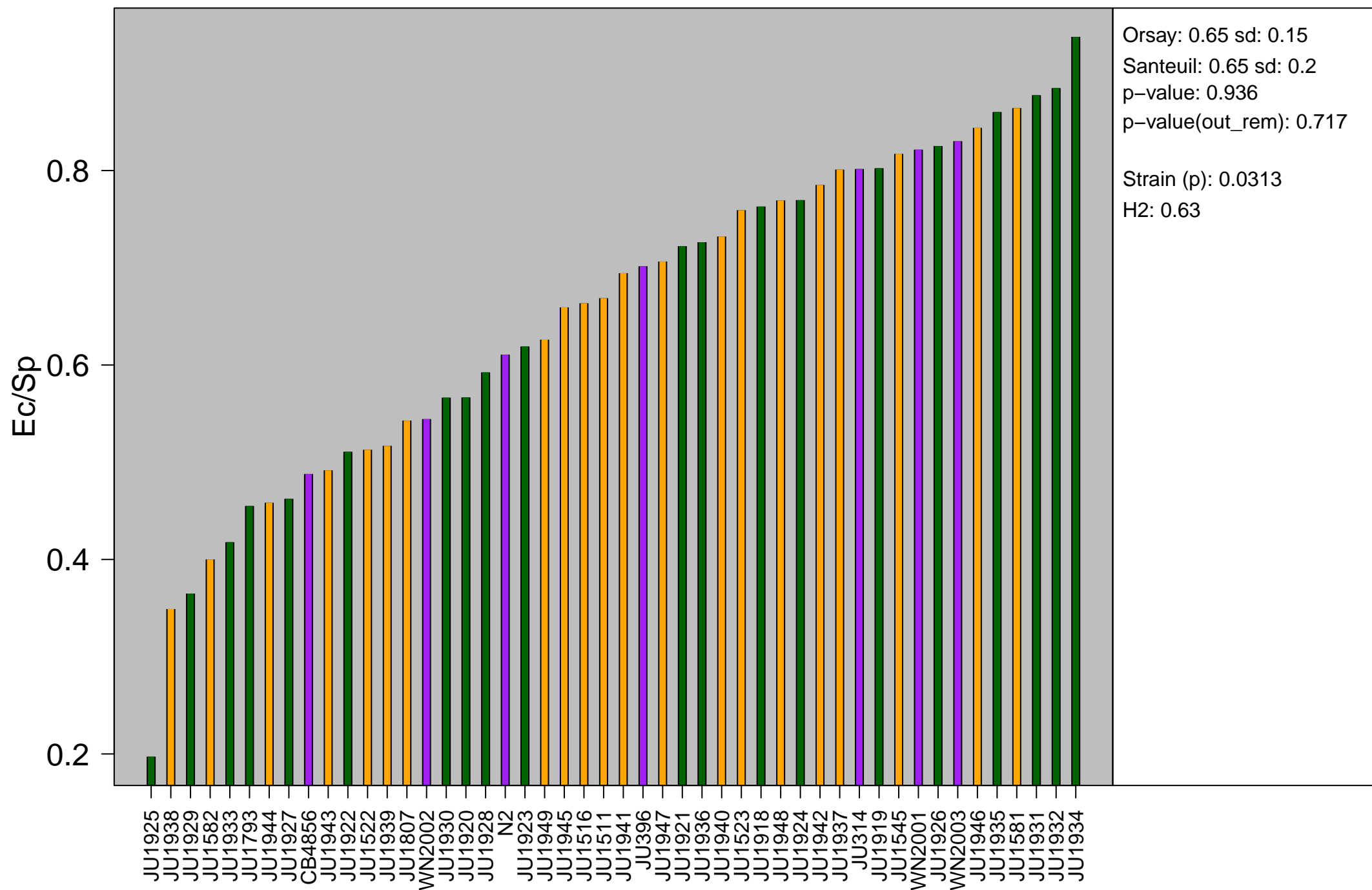

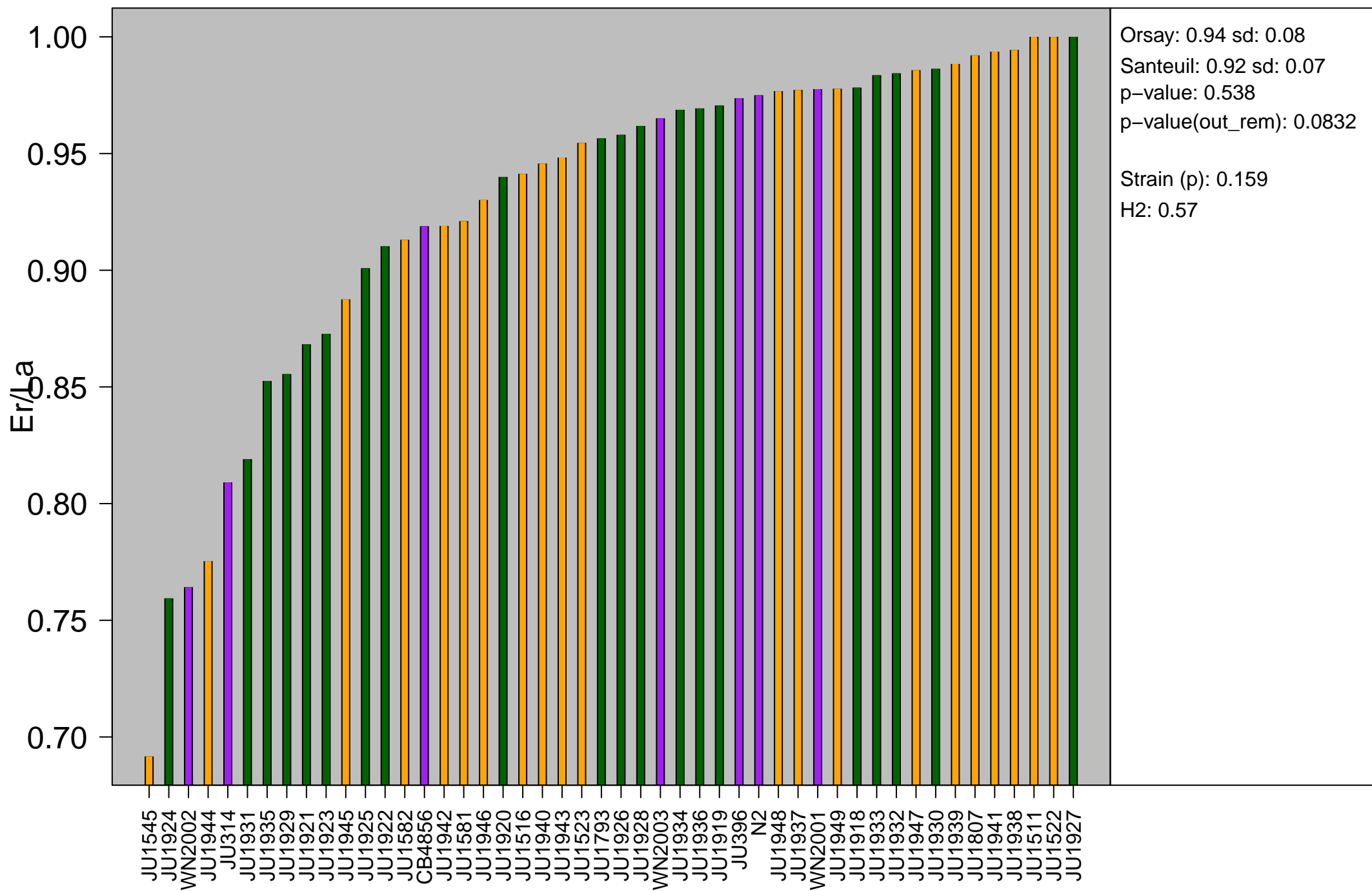

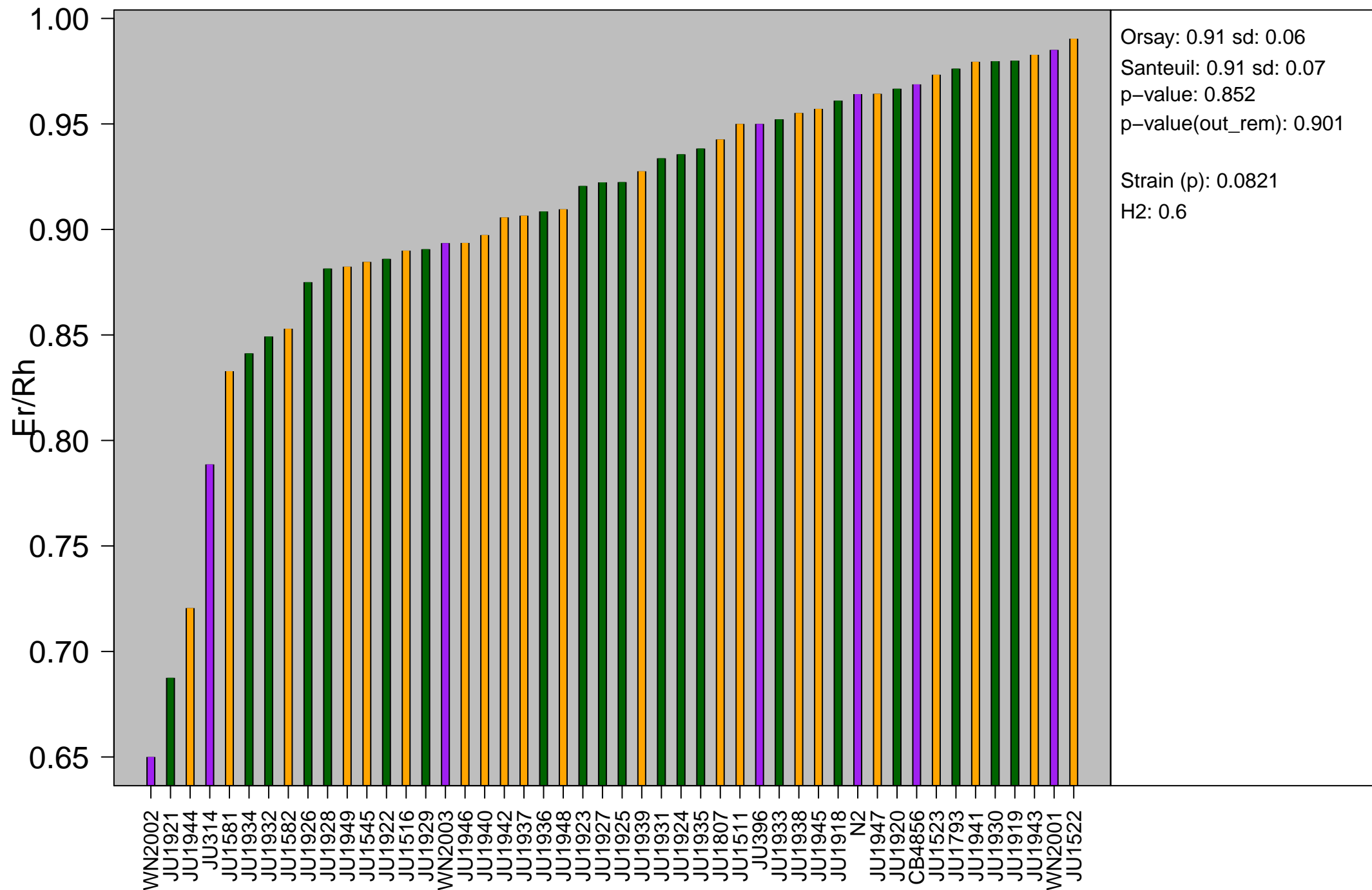

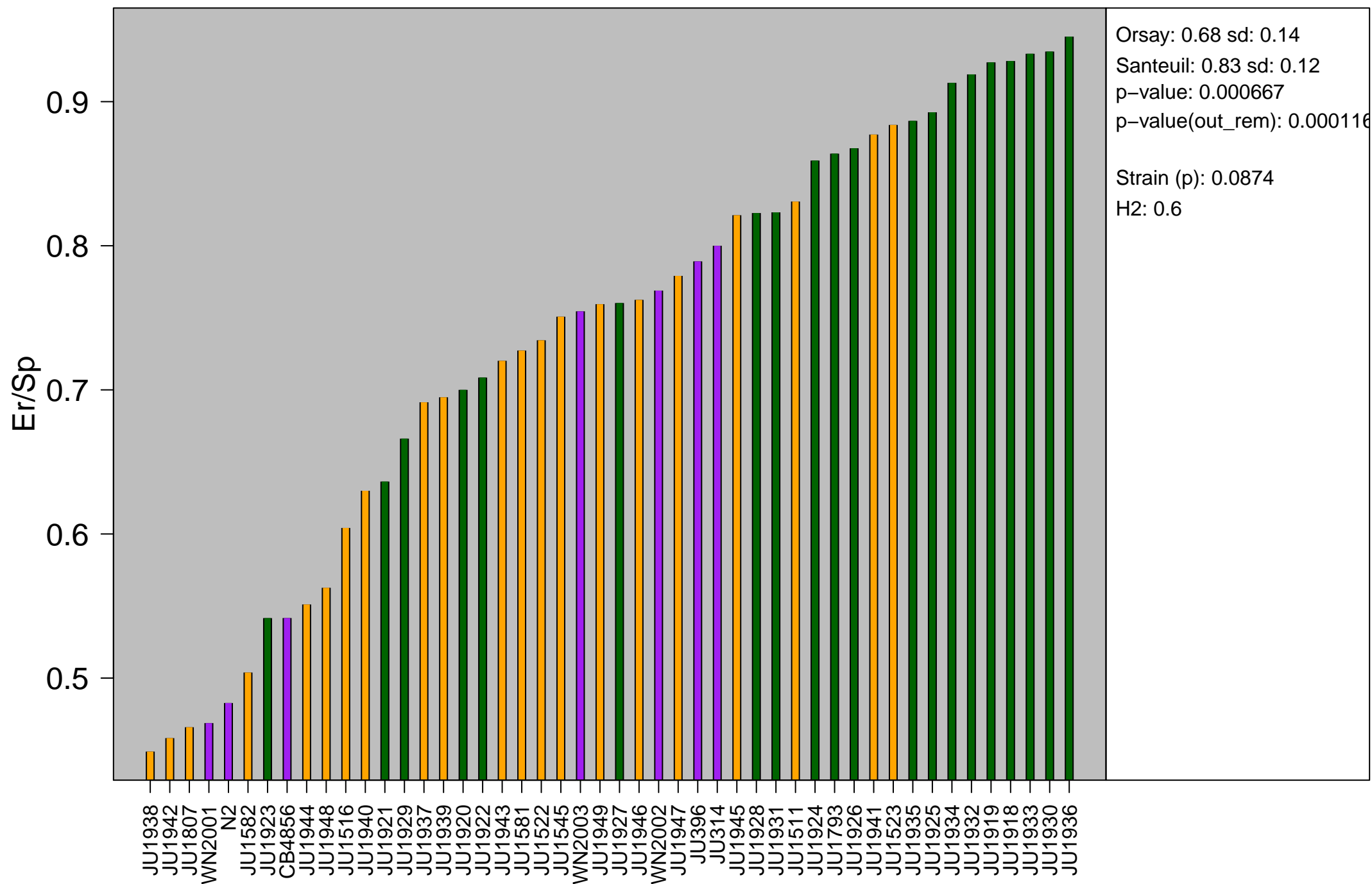

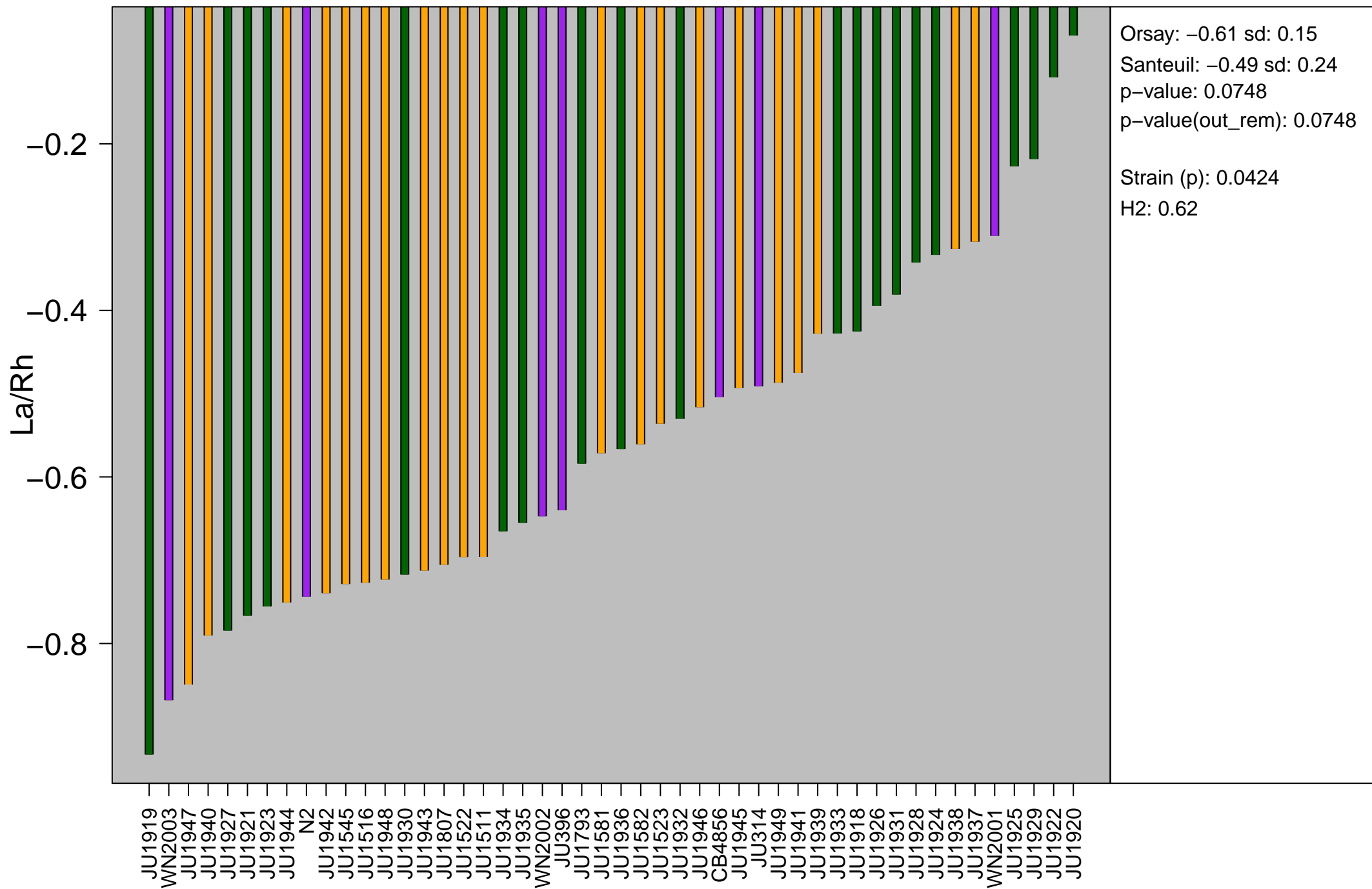

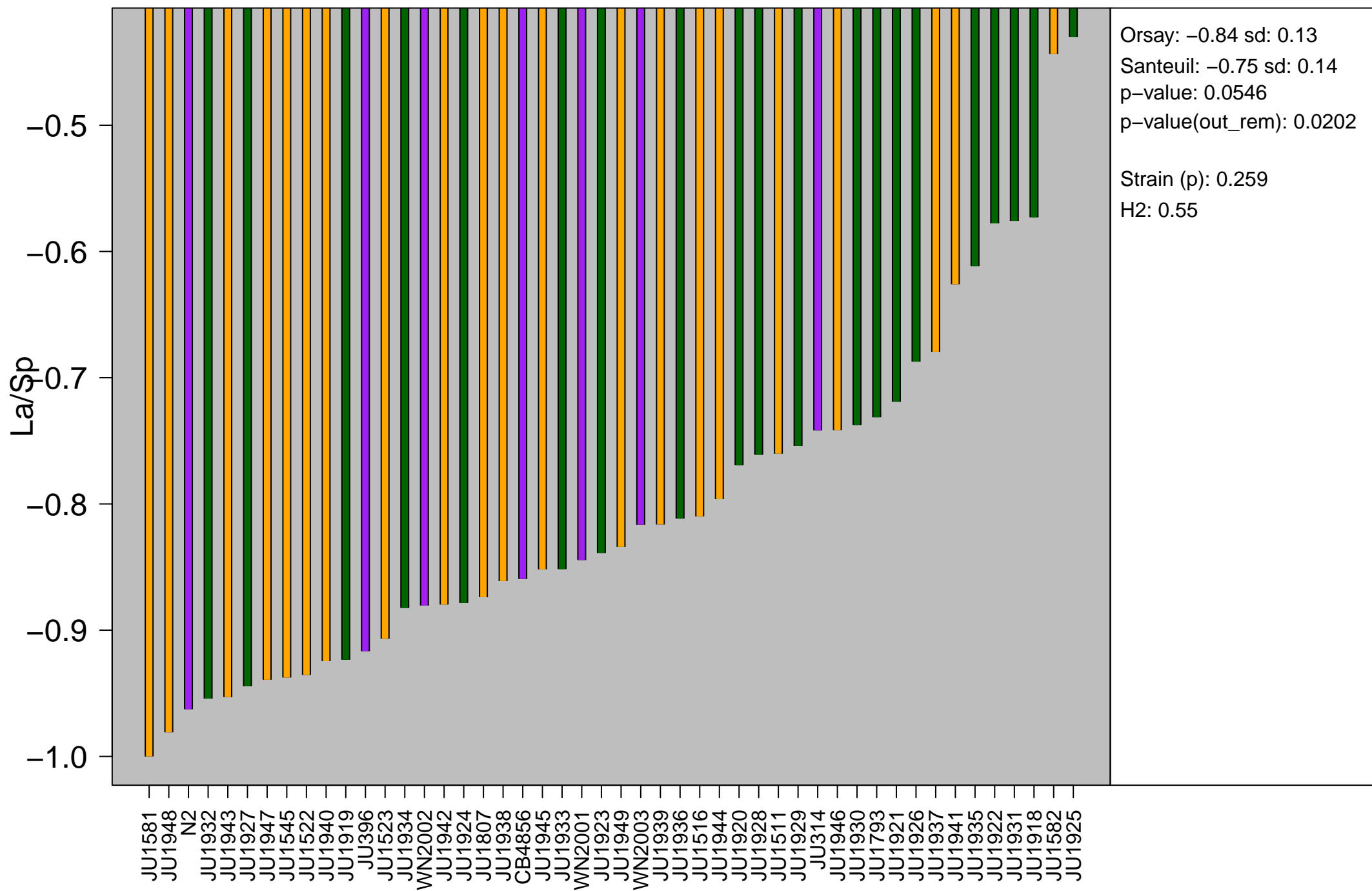

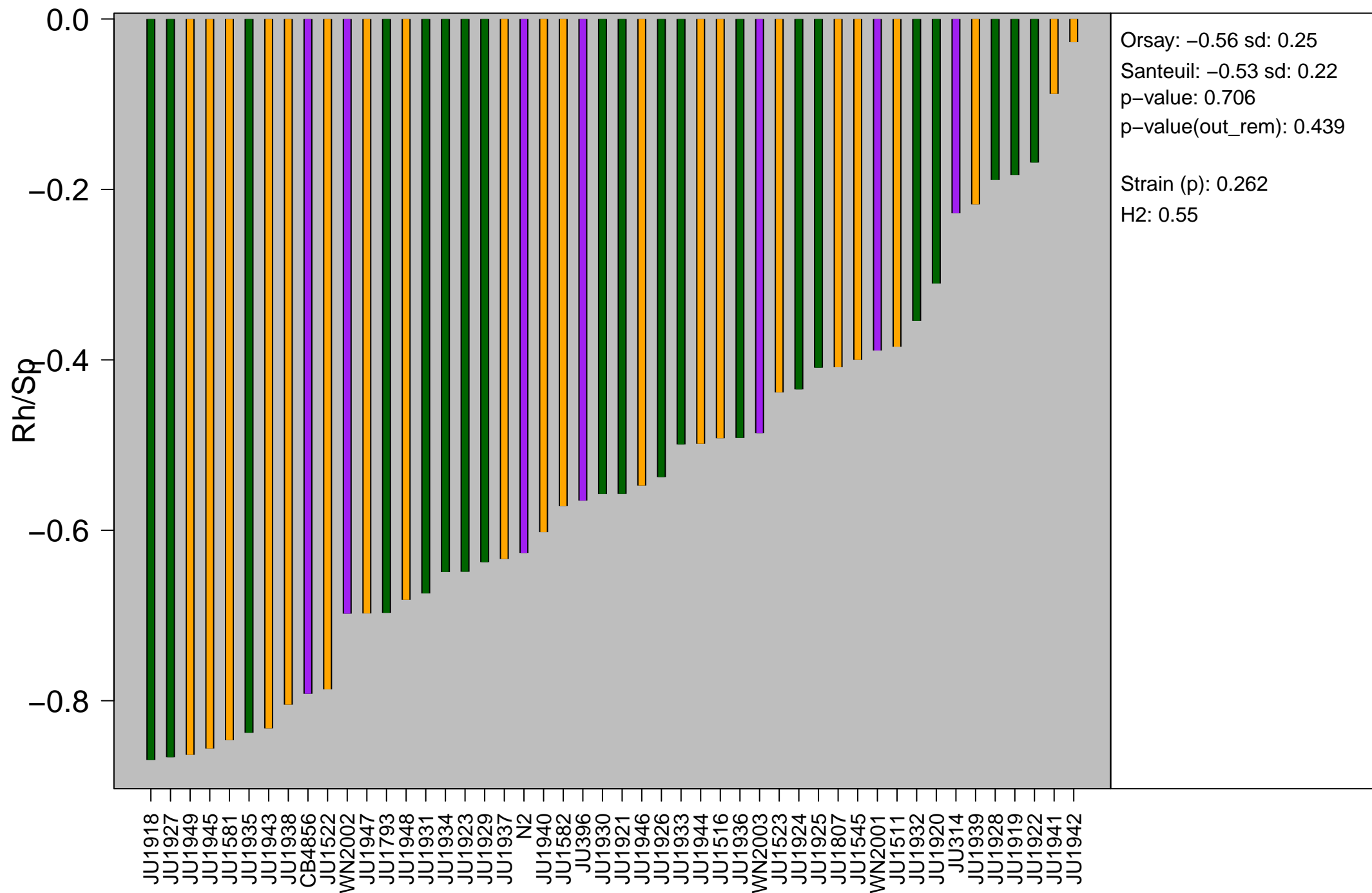

Supplement: Additional file 8 — Phenotypes of the wild isolates. Strains from Orsay are shown in orange, strains from Santeuil in green, and the out-group strains in purple. The right panel shows the statistics, mean and standard deviation (SD) as well as the P-value of the t-test of the phenotypic difference between the Orsay and Santeuil groups with and without outliers removed. When applicable, an ANOVA on strain was performed, and the P-value is shown. Lastly, the heritability (H2) was calculated. Labels on the y-axis refer to the phenotypes described in Table 2. [file 1741-7007-11-93-S8.pdf]
