# Supplementary material for: Gene-environment and protein-degradation signatures characterize genomic and phenotypic diversity in wild Caenorhabditis elegans populations
Source: BMC Biol. 2013 Aug 19;11:93. doi: 10.1186/1741-7007-11-93 (PMC3846632; doi:10.1186/1741-7007-11-93)
Supplement: Additional file 9 — Food preference assay. (A) Set-up of the food preference assay and the calculation of the Choice Index (CI). A, bacterium A; B, bacterium B. (B) Schematic overview of the results of the food preference assay. Green indicates bacteria isolated in Santeuil, orange indicates bacteria isolated in Orsay, purple indicates standard laboratory food OP50. All numbers are percentages and are the average of all strains in the experiment. The percentages near the bacteria indicate the fraction of worms that prefer that particular bacterium when tested together with the bacterium at the opposite end of the line. The percentages in or near the yellow circles indicate the fraction of worms that did not choose between the two bacteria. For example, when offered a choice between Sphingobacterium and Lactococcus lactis, on average 85.7% of all worms of all strains preferred Sphingobacterium, 5.5% preferrred L. lactis, and 8.8% did not make a choice between these bacteria. [file 1741-7007-11-93-S9.pdf]

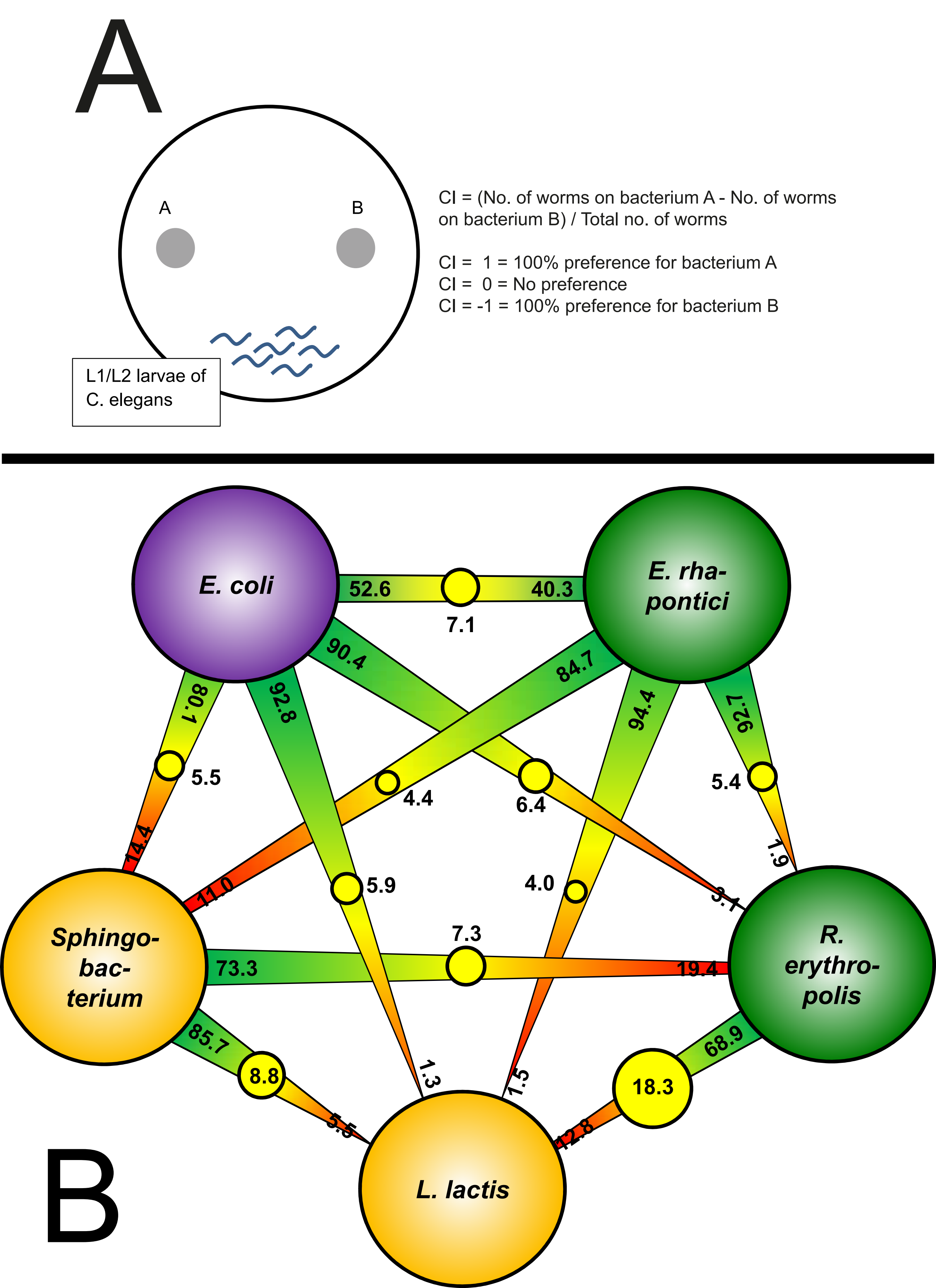
A
A
B
L1/L2 larvae of
C. elegans
CI = (No. of worms on bacterium A - No. of worms
on bacterium B) / Total no. of worms
CI = 1 = 100% preference for bacterium A
CI = 0 = No preference
CI = -1 = 100% preference for bacterium B
B
E. coli
E. rha-
pontici
Sphingo-
bac-
terium
R.
erythro-
polis
L. lactis
52.6
7.1
40.3
90.4
6.4
3.1
84.7
4.4
11.0
80.1
5.5
14.4
92.8
5.9
1.3
94.4
4.0
1.5
92.7
5.4
1.9
73.3
7.3
19.4
85.7
8.8
5.5
68.9
18.3
12.8
