# Supplementary material for: Gene-environment and protein-degradation signatures characterize genomic and phenotypic diversity in wild Caenorhabditis elegans populations
Source: BMC Biol. 2013 Aug 19;11:93. doi: 10.1186/1741-7007-11-93 (PMC3846632; doi:10.1186/1741-7007-11-93)
Supplement: Additional file 10 — Detailed description of Methods. [file 1741-7007-11-93-S10.docx]

**Materials and methods**

**Nematode- and bacterial strains**

The main set of strains of *C. elegans* that was used in this research comprised forty-one new strains that were isolated by Marie-Anne Félix from two different locations in France (Orsay and Santeuil). An out-group was formed that contained three new strains isolated in the Netherlands, two strains previously isolated in France and the canonical strains N2 (Bristol) and CB4856 (Hawaii). See **Additional file 1 (worksheet A)** for more information about these strains.

All strains were routinely maintained on NGM with *Escherichia coli* OP50 as a food source [[1](#_ENREF_1)] for 2-3 generations after which the populations were frozen in -80°C until further use. *E. coli* OP50 was used as a food source in all experiments, except for the population growth experiment in which *Bacillus thuringiensis* NRRL B-18247 and *B. thuringiensis* DSM-350 were used next to *E. coli* OP50 [[2](#_ENREF_2)]. In the food preference experiment, besides *E. coli* OP50, *Erwinia rhapontici* and *Rhodococcus erythropolis* (both isolated from Santeuil), and *Lactococcus lactis* and *Sphingobacterium* sp. (both isolated from Orsay) that were isolated and identified by Marie-Anne Félix and Buck Samuel, were used. The bacteria isolated from Santeuil were not found in Orsay and *vice versa*.

**Genomic DNA analysis: worm culturing, DNA isolation, DNA-microarrays and statistical analysis**

Genotyping was performed with genomic DNA microarrays [[3](#_ENREF_3)]. Populations of mixed stages were freshly inoculated on NGM-plates were used and cultured for 96 hours at 20ºC before sampling.

The microarrays used were *C. elegans* (V2) Gene Expression Microarray 4X44K slides, manufactured by Agilent Technologies (Santa Clara, CA, USA). On these arrays, 43803 60-mer probes are present that were designed based on Wormbase 188, ENSEMBL release 49, UniGene release 37, RefSeq release 27, TIGR release 9 and UCSC cd4 Sanger W170. Labelling of the genomic DNA with Cyanine-3 and Cyanine-5, hybridisation, scanning of the microarrays with an Agilent High Resolution C Scanner and data extraction with Agilent Feature Extraction Software version 10.7 were performed as recommended by Agilent in the ‘Oligonucleotide Array-Based CGH for Genomic DNA Analysis; Enzymatic Labeling for Blood, Cells or Tissues (with a High Throughput option)’ –protocol, version 6.3.

Genomic DNA isolation was not performed according to this protocol because the recommended method (to use a Qiagen DNeasy Blood & Tissue Kit) did not yield the expected amount of DNA, nor did it yield DNA of sufficiently high quality. Instead, the NucleoSpin Tissue Kit from Machery-Nagel (Düren, Germany) was used, with the following modifications: I) after lysis, an RNAse step was performed as optioned in the protocol; II) the second wash step was replaced by a wash step with 80% EtOH; III) elution was done following the third alternative procedure described on page 8 of the manual.

For processing the data of the DNA microarrays the “Limma” package for the “R” environment was used [[4](#_ENREF_4)]. Background correction was done by using the Substract method from the Limma package. Loess within-array normalisation and Scale between-array normalisation were used to process the raw intensity values [[5](#_ENREF_5)]. The obtained normalised intensities were used for further analysis.

Genotypes were compared by calculating, per spot, the ratio of the intensities of each strain with the mean intensity over all strains. The genes with a ratio of >0.5 or < -0.5 were considered the polymorphic genes and were used in further investigations. Principal component analysis (PCA) was done using the polymorphic genes from all strains, except JU1545 for which the DNA-array was of bad quality. The phylogenetic tree was made using the “R” package “Phangorn” [[6](#_ENREF_6)]. The un-rooted neighbour joining (NJ) tree was made from a distance matrix made from the ratios of the polymorphic genes. Linear models were used to calculate the significance of the variation in DNA hybridisation intensities linked to isolation-sites and the identified genetic groups: [log2(intensity) DNA ~ isolation site(s); log2(intensity) DNA ~ genetic group(s)]. The significance thresholds, adjusted for multiple testing, were determined by permutation, for which the same model was used with the spot intensities randomly distributed over the genotypes (the p value which gave a ratio of false positives/true positives < 0.05 was used).

**mRNA analysis: culturing, isolation, RNA-microarrays and statistical analysis**

For the mRNA microarrays, any few males were discarded and only hermaphrodites grown on *E. coli* OP50 were used. Two independent replicates of each strain were analysed. The populations were stage synchronised by bleaching [[7](#_ENREF_7)] and the eggs were placed on NGM-plates and incubated at 20ºC for 47 hours to obtain populations of late L4 larvae. After harvesting the larvae, they were flash frozen in liquid nitrogen and stored at -80°C. For mRNA isolation, an RNEasy Micro Kit from Qiagen (Hilden, Germany) was used, following the ‘Purification of Total RNA from Animal and Human Tissues’ protocol provided with the kit, with modified lysing procedure. In short, to each frozen worm pellet 150 µl RLT buffer, 295 µl RNAse-free water, 800 µg/ml proteinase K and 1% ß-mercaptoethanol were added. This suspension was incubated in a Thermomixer (Eppendorf, Hamburg, Germany) at 55°C and 1200 rpm for one hour or until the sample was clear. After centrifugation, 0.5 volume of 96% ethanol was added to the supernatant. After this, the ‘Two-Color Microarray-Based Gene Expression Analysis; Low Input Quick Amp Labeling’ -protocol, version 6.0 was followed, starting from step 5 as mentioned in this protocol.

The microarrays used were *C. elegans* (V2) Gene Expression Microarray 4X44K slides, manufactured by Agilent (also see section above on *mRNA analysis: worm culturing, isolation and -microarrays*). mRNA isolation, labelling with Cyanine-3 and Cyanine-5, and hybridisation were performed as recommended by Agilent in the protocol mentioned above. The microarrays were scanned using an Agilent High Resolution C Scanner, using the settings as recommended in the above mentioned manual. Data was extracted with the Agilent Feature Extraction Software version 10.5, following manufacturers’ guidelines.

For processing the data of the RNA microarrays the “Limma” package for the “R” environment was used. No background correction of the RNA-array data was performed as recommended [[4](#_ENREF_4)]. For within-array normalisation of the RNA-array data the Loess method was used and for between-array normalisation the Quantile method was used. The obtained normalised intensities were used for further analysis [[5](#_ENREF_5)].

Expression variation was determined by linear models. We explained the variation in intensities by batch, DNA hybridisation, genetic group and genotype. Significance thresholds, adjusted for multiple testing were determined by permutations of all spots on the array. In the permutations the RNA hybridisation intensities were randomly distributed over the genotypes and batches (the p value which gave a ratio of false positives/true positives < 0.05 was used).

**Enrichment analysis**

All enrichment analyses were done using a hyper-geometric test. The number of genes selected by one of the criteria in this paper (for example significantly linked to a genetic group) were compared to the genes with a specific annotation (for example c-type lectin). The chance that a number of genes will be overlapping depends on the total group size, the number of genes selected and the number of genes with a specific annotation. This, together with the number of overlapping genes can be used in a hyper-geometric test to determine enrichment. We considered annotation groups enriched when the overlap was larger than 3 genes and the significance was –log10P > 2.5. The annotation groups were obtained from Wormmart, WS220 release.

**Phenotypic assays**

*Developmental time and generation time*

Eggs were isolated using a standard hypochlorite bleach procedure and allowed to hatch overnight in S-basal at 17°C [[7](#_ENREF_7)]. L1 juveniles were transferred to NGM agar plates seeded with *E. coli* OP50. This moment was taken as the starting point of larval development. The agar used was a mixture of 27.5g nutrient agar (Oxoid – CM0003), 12.5g agar N°1 (Oxoid – LP0011), supplemented with 5 mg cholesterol and 25ml potassium phosphate buffer pH 6 per liter. One batch of L1's was transferred to *E. coli* plates in the morning while a second batch was initiated in the evening.  This parallel setup allowed us to increase time resolution of the observations.  The cultures were incubated at 24°C and observed at regular time intervals. Developmental time is defined as the period between worm inoculation and the moment at which the first worms with open vulva were observed in the culture. Generation time is the period between worm inoculation and the first appearance of eggs in the culture.

*Length and width*

Young gravid worms, grown on *E. coli* OP50 lawns in parallel with the cultures used for developmental and generation time, were rinsed off the plates and stretched by adding a few drops of bleach to the worm suspension. Small aliquots containing a few thousand of worms were loaded into a RapidVue particle analyzer (Beckman Coulter) and cycled through the fluidic system. Morphometric analysis of length and average width of individual worms was performed at a frame rate of 30fps and was ended automatically when 2000 worms were measured. Objects that showed border overlap, out-of-focus, crossing and non-fiber shape were automatically censored. The average volume of the worms in a sample was calculated applying the cylinder formula to the average length and width obtained from the worm population statistics.

*Population growth*

Population growth of *C. elegans* was measured as a proxy for fitness in either presence or absence of pathogenic bacteria. It was examined on 10 cm diameter Petri dishes with peptone-free NGM inoculated with *E. coli* OP50 and either the pathogenic *Bacillus thuringiensis* strain NRRL B-18247 or the non-pathogenic *B. thuringiensis* DSM-350 [[2](#_ENREF_2)]. At the beginning of the experiment, 10 single L4 worms were placed onto the bacterial lawn with a sterilized worm picker. After 96 h (on the fifth day), the number of worms on the plate was counted. The experiment was performed at 20°C. All strains were tested in parallel in randomized order and with neutral coding of plates to avoid observer bias.

*Genetic/strain effect*

For all phenotypes, the number of replicates ranged from 40-120 replicates. ANOVA was used where strain and the identified genetic groups were considered as explaining variables for the variation in the different phenotypes.

*Food preference assay*

Nematodes were age synchronised by bleaching [[7](#_ENREF_7)], the eggs were suspended in 1 mL of S-complete medium in Eppendorf tubes and were allowed to hatch overnight at 20°. Bacteria *Escherichia coli* OP50, *Erwinia rhapontici*, *Rhodococcus erythropolis*, *Lactococcus lactis* and *Sphingobacterium* sp. were maintained on M17 agar plates and cultured in liquid M17 medium for 16 hours at 37°C (*E. coli* OP50) or 28°C (all other strains) before use. The cultures of *E. rhapontici*, *R. erythropolis* and *Sphingobacterium* sp. were then diluted 200X in M9. To test the food preference of the nematodes, 5 µl drops of two different bacterium cultures were placed on NGM agar in each well of a 12 wells plate (**Additional file 9 (panel A)**). These drops only differ in the presence of the different bacteria, but not as to the edia used. A drop with juveniles until stage L2 was added to each well and the plate was incubated overnight at 20°C (for densities of worms see **Additional file 1 (worksheet P)**). Importantly, the juveniles never ate bacteria before the experiment was started, to prevent getting used to *E. coli*. All possible combinations of two bacteria were assayed in duplicate for each *C. elegans* strain, leading to at least six replicates of each combination for each genetic group. After incubation, the worms on each bacterium and the worms that were outside the bacteria were counted and the Choice Index was calculated [[8](#_ENREF_8)]. Subsequently, *t*-tests were performed to determine the significance of the differences in food preference for several groups of *C. elegans* strains.

**Microsatellite analysis**

Population genetic differentiation was assessed using six microsatellite loci (**Additional file 1 (worksheet C)**), which we previously identified to be highly variable for natural and experimental *C. elegans* populations [[9](#_ENREF_9)]. DNA was isolated for each considered *C. elegans* strain by first washing off worms from nematode growth medium plates and then subjecting them to the animal tissue protocol of the Qiagen DNeasy Blood & Tissue Kit (Qiagen Ltd.). PCR amplification was performed in 15 μl reaction volumes, containing GoTaq DNA polymerase (2 U; Promega Ltd.), GoTaq Colorless PCR buffer (Promega Ltd.), 0.25 mM dNTPs, 0.1 mM of the respective primers, and template DNA. The temperature profile consisted of initial denaturation at 95˚C for 2 min; 34 cycles of denaturation at 95˚C for 20 s, annealing at the optimal temperature of each primer pair (**Additional file 1 (worksheet C)**) for 30 s; and extension at 72˚C for 30 s; followed by a final extension step at 72˚C for 10 min. Microsatellite copy numbers were assessed using 5 to 10 µl of the amplification products and capillary electrophoresis with the Megabase Fragment Analysis system, following manufacturer instructions (Amersham Biosciences Corp.). We used Arlequin V 3.5.1.3 (An Integrated Software for Population Genetics Data Analysis) and AMOVA to test for population genetic differentiation and reconstruction of a minimum spanning network.

**References**

1. Sulston JE, Hodgkin J: **Methods**. In: *The nematode Caenorhabditis elegans.* Edited by Wood WB. New York: Cold Spring Harbor Laboratory; 1988: 587-606.

2. Boehnisch C, Wong D, Habig M, Isermann K, Michiels NK, Roeder T, May RC, Schulenburg H: **Protist-type lysozymes of the nematode *Caenorhabditis elegans* contribute to resistance against pathogenic *Bacillus thuringiensis***. *PloS one* 2011, **6**(9):e24619.

3. Flibotte S, Edgley ML, Maydan J, Taylor J, Zapf R, Waterston R, Moerman DG: **Rapid high resolution single nucleotide polymorphism-comparative genome hybridization mapping in *Caenorhabditis elegans***. *Genetics* 2009, **181**(1):33-37.

4. Smyth GK: **Limma: linear models for microarray data**. In: *Bioinformatics and computational biology solutions using R and Bioconductor.* Edited by Gentleman R, Carey VJ, Huber W, Irizarry RA, Dudoit S. New york: Springer Science+Business Media; 2005: 397-420.

5. Smyth GK, Speed T: **Normalization of cDNA microarray data**. *Methods* 2003, **31**(4):265-273.

6. Schliep KP: **phangorn: phylogenetic analysis in R**. *Bioinformatics* 2011, **27**(4):592-593.

7. Emmons SW, Klass MR, Hirsh D: **Analysis of the constancy of DNA sequences during development and evolution of the nematode *Caenorhabditis elegans***. *Proc Natl Acad Sci U S A* 1979, **76**(3):1333-1337.

8. Abada EA, Sung H, Dwivedi M, Park BJ, Lee SK, Ahnn J: ***C. elegans* behavior of preference choice on bacterial food**. *Molecules and cells* 2009, **28**(3):209-213.

9. Haber M, Schungel M, Putz A, Muller S, Hasert B, Schulenburg H: **Evolutionary history of *Caenorhabditis elegans* inferred from microsatellites: evidence for spatial and temporal genetic differentiation and the occurrence of outbreeding**. *Mol Biol Evol* 2005, **22**(1):160-173.
